# High‐Valent Copper Catalysis Enables Regioselective Fluoroarylation of *Gem*‐Difluorinated Cyclopropanes

**DOI:** 10.1002/advs.202401243

**Published:** 2024-03-09

**Authors:** Xiuli Wu, Xiangyu Song, Ying Xia

**Affiliations:** ^1^ West China School of Public Health and West China Fourth Hospital West China‐PUMC C.C. Chen Institute of Health and State Key Laboratory of Biotherapy Sichuan University Chengdu 610041 China

**Keywords:** C─C bond activation, fluoroarylation, *gem*‐difluorinated cyclopropanes, high‐valent copper catalysis, regioselectivity

## Abstract

Transition‐metal (TM) catalyzed reaction of *gem*‐difluorinated cyclopropanes (*gem*‐DFCPs) has drawn much attention recently. The reaction generally occurs via the activation of the distal C─C bond in *gem*‐DFCPs by a low‐valent TM through oxidative addition, eventually producing mono‐fluoro olefins as the coupling products. However, achieving regioselective activation of the proximal C─C bond in *gem*‐DFCPs that overcomes the intrinsic reactivity via TM catalysis remains elusive. Here, a new reaction mode of *gem*‐DFCPs enabled by high‐valent copper catalysis, which allows exclusive activation of the congested proximal C─C bond is presented. The reaction that achieves fluoroarylation of *gem*‐DFCPs uses NFSI (N‐fluorobenzenesulfonimide) as electrophilic fluoro reagent and arenes as the C─H nucleophiles, enabling the synthesis of diverse CF_3_‐containing scaffolds. It is proposed that a high‐valent copper species plays an important role in the regioselective activation of the proximal C─C bond possibly via a σ‐bond metathesis.

## Graphical abstract

A high‐valent copper‐catalyzed regioselective fluoroarylation of *gem‐*DFCPs with simple arenes as the C─H nucleophiles and NFSI as the electrophilic fluororeagent is reported. The activation of the congested proximal C─C bond is proposed via a concerted σ‐bond metathesis with a high‐valent Cu─F species.

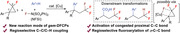

## 1 Introduction

**Scheme 1:**
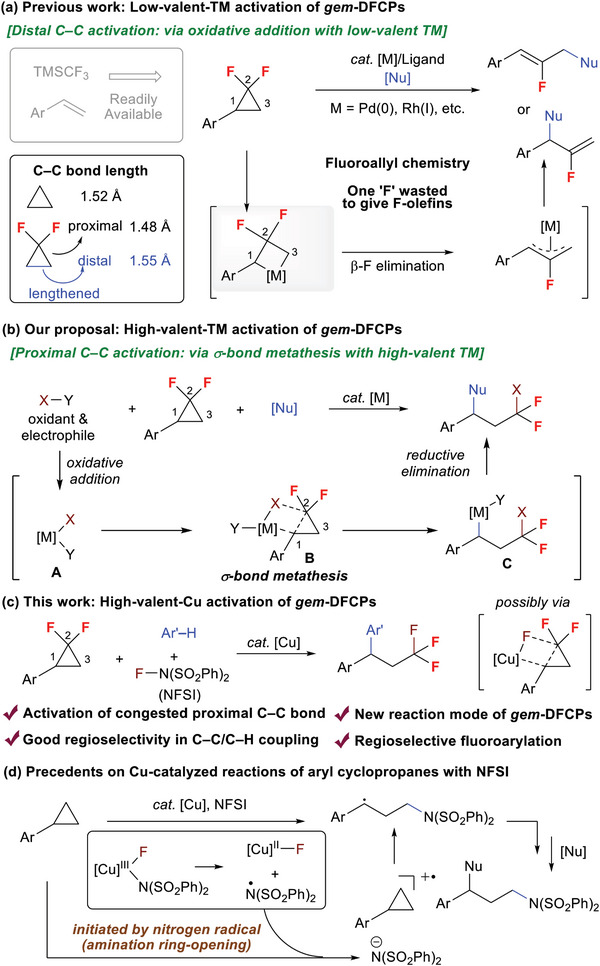
Transition‐metal (TM)‐catalyzed reactions of *gem*‐DFCPs and their background.

Fluorine substitution is very important in modulating the properties of organic molecules by introducing fluorine atoms and/or fluorinated groups.^[^ ^1^ ^]^ As an illustration, fluorination is frequently employed to tune the efficacy and metabolism of drug candidates.^[^ ^1f^ ^]^ Remarkably, one out of every five newly approved small molecule drugs by the FDA in 2023 contain fluorine.^[^ ^1g^ ^]^ Moreover, the inclusion of fluorine atoms into organic materials, like optoelectronic and electronic sensors, can lead to better electron injection efficiency due to the diminished energy levels between the HOMO and LUMO.^[^ ^1a^ ^]^ Consequently, the development of a new strategy that can efficiently incorporate fluorine motifs into organic molecules is therefore in high demand.^[^ ^2^ ^]^ In this context, *gem*‐difluorinated cyclopropanes (*gem*‐DFCPs), owing to their facile availability (for selected reviews and reports on the synthesis of *gem*‐DFCPs, see ref. [3]) and unique reactivity^[^ ^4^ ^]^ (**Scheme** **1**a, left), have emerged as highly versatile building blocks for the synthesis of fluorine‐containing molecules. In recent years, the utilization of transition‐metal (TM) catalysts renders the cross‐coupling reactions of *gem*‐DFCPs with various nucleophiles (for reviews, see ref. [5]; for selected reports on Pd catalysis with linear‐selectivity, see ref. [6]; for selected reports on Pd catalysis with branched‐selectivity, see ref. [7]; for Rh catalysis, see ref. [8]; for Co catalysis, see ref. [9]; for Ni catalysis, see ref. [10]), providing a highly efficient approach for the synthesis of monofluorinated alkenes (instead of monofluorinated alkenes, *gem*‐difluorinated carbocycles and fully‐substituted alkyl vinyl ethers can also be synthesized from *gem*‐DFCPs by Rh‐ or Cu‐catalysis, see ref. [11]). Likely due to the influence of the CF_2_ moiety on the cyclopropyl ring,^[^ ^4a^ ^]^ the lengthened distal C─C bond was selectively cleaved with a low‐valent TM (mainly Pd^0^ and Rh^I^) via oxidative addition, which coupled with nucleophiles through fluoroallyl chemistry to afford the final products with incorporation of one fluorine atom (Scheme 1a, right).^[^ ^6^, ^7^, ^8^ ^]^ To the best of our knowledge, strategy that can activate the congested proximal C─C bond in *gem*‐DFCPs under TM catalysis has not been developed yet (for representative reports on TM‐free reaction of *gem*‐DFCPs, see ref. [12])

Compared with the activation mode of C─C cleavage via oxidative addition with low‐valent TM (for reviews on C─C activation via oxidative addition with low‐valent TM, see ref. [13]), it can be envisaged that a high‐valent TM complex (for selected reviews, see ref. [14]) may be able to activate the C─C bond in *gem*‐DFCPs considering its partial π‐bond nature in cyclopropyl ring. In the hypothesis, the high‐valent TM complex **A**, generated via oxidative addition with an electrophilic reagent, is highly electrophilic and possible to interact with the C─C bond in *gem*‐DFCPs, for example, through a σ‐bond metathesis (**B**) to give a benzyl metal species **C**. The reaction of **C** with a suitable nucleophile via reductive elimination or another process would deliver the C─C difunctionalization product (Scheme 1b).

Based on this concept, we herein disclosed that a high‐valent copper‐catalyzed (for selected reports on high‐valent Cu catalysis, see ref. [15]) regioselective fluoroarylation of *gem*‐DFCPs through proximal C─C bond activation by utilizing NFSI (*N*‐fluorobenzenesulfonimide) as the oxidant and electrophilic fluorine source and simple electron‐rich arenes as the C─H nucleophiles. In this new reaction mode of *gem*‐DFCPs, it was proposed that the regioselective proximal C─C activation is triggered by the high‐valent copper species, other than by low‐valent metal via previously well‐developed oxidative addition process (Scheme 1c). Significantly, this transformation allows for the synthesis of exclusive CF_3_‐containing scaffolds via regioselective fluoroarylation, rather than fluoroallyl compounds via fluoroallyl chemistry, by sequentially introducing a fluorine group and an aryl nucleophile regioselectively. Note that NFSI has been extensively utilized in copper‐catalyzed reactions of simple cyclopropanes.^[^ ^16^ ^]^ In these reactions, the Cu^III^ species undergoes an LMCT process to generate a nitrogen radical, which triggers the ring‐opening process with the nitrogen fragment of NFSI incorporated in the final product (Scheme 1d).

## 2 Results and Discussion

Our investigation began by employing 1‐(2,2‐difluorocyclopropyl)−4‐methylbenzene (**1a**) as the model substrate, anisole as the C─H nucleophile, and NFSI as the electrophilic fluorine reagent to optimize the reaction conditions (**Table** **1**). After systematically optimization, it was found that the designed reaction proceeded smoothly by using 10 mol% Cu(MeCN)_4_PF_6_, 10 mol% [B(neop)]_2_ [bis(neopentyl glycolato)diboron],^[^ ^17^ ^]^ and PhCl (1 m) as the solvent without additional ligand at 80 °C for 3 h. Under the optimized conditions, the reaction produced the fluoroarylation products with a *para*/*ortho* selectivity of 11:1, and the major product **2a** was isolated in a 73% yield (entry 1). The control experiment showed that the reaction didn't work without the participation of Cu(MeCN)_4_PF_6_ (entry 2). In the absence of [B(neop)]_2_, there is a substantial decline in the conversion of *gem*‐DFCP **1a**, resulting in a low yield of the product (entry 3). The utilization of alternative Cu^I^ catalysts, including Cu(MeCN)_4_OTf, Cu(MeCN)_4_BF_4_, Cu(OAc), or CuBr, led to a slight decrease in yields with the same level of regioselectivities (entries 4–7). Furthermore, Cu^II^ salts such as CuBr_2_ and Cu(OTf)_2_ worked in this transformation, but the fluoroarylation product was obtained in decreased yields (entries 8–9). The boron additive has some influence on this reaction (entries 10–13). For instance, replacement of [B(neop)]_2_ with [B(Pin)]_2_ [bis(pinacolato)diboron], [B(OH)_2_]_2_ or PhB(neop) somewhat reduced the yield to 61−68% (entries 10–12). Reducing the loading of [B(neop)]_2_ to 5 mol% slightly decreases the yield (entry 13). As for the solvent effect, Toluene, DCE, and DCM showed similar results compared with PhCl (entries 14–16), but the reaction totally shut down with THF or MeOH as the solvents (entries 17–18). Variation of the reaction concentration from 0.5 to 2 m had a marginal influence on the reaction outcome (entries 19–20). Higher reaction temperature compromised the regioselectivity, whereas lower reaction temperature decreased the reaction efficiency (entries 21–22).

**Table 1:** Optimization of reaction conditions.

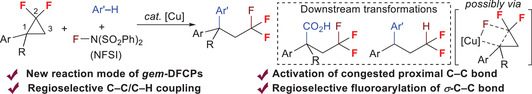			
Entry^a)^	Variations	Ratio (2a:2a′)^b)^	Yield (%)^c)^
1	None	11:1	83 (73)^d)^
2	No Cu(MeCN)_4_PF_6_	‐	0
3	No B[(neop)_2_]_2_	8:1	16
4	Cu(MeCN)_4_OTf	10:1	67
5	Cu(MeCN)_4_BF_4_	10:1	78
6	Cu(OAc)	10:1	63
7	CuBr	9:1	78
8	CuBr_2_	10:1	43
9	Cu(OTf)_2_	10:1	63
10	[B(Pin)]_2_	10:1	68
11	[B(OH)_2_]_2_	10:1	64
12	PhB(neop)	7:1	61
13	5 mol% [B(neop)]_2_	10:1	77
14	Toluene	11:1	75
15	DCE	9:1	85
16	DCM	9:1	81
17	THF	–	0
18	MeOH	–	0
19	0.5 M	10:1	77
20	2 M	10:1	78
21	100 ^o^C	7:1	85
22	60 ^o^C	11:1	65

^a)^ Reaction conditions: **1a** (0.2 mmol), anisole (0.4 mmol), NFSI (0.3 mmol), [Cu] (10 mol%), [B] (10 mol%) in PhCl (200 µL) at 80 °C for 3 h unless otherwise noted;

^b)^ The ratio (*p*:*o* = **2a**:**2a′**) was measured by ^19^F NMR;

^c)^ Total yields of **2a** and **2a′** were determined by ^1^H NMR using 1,1,2,2‐tetrachloroethane as the internal standard;

^d)^ Isolated yield of the major product **2a**.

**Scheme 2:**
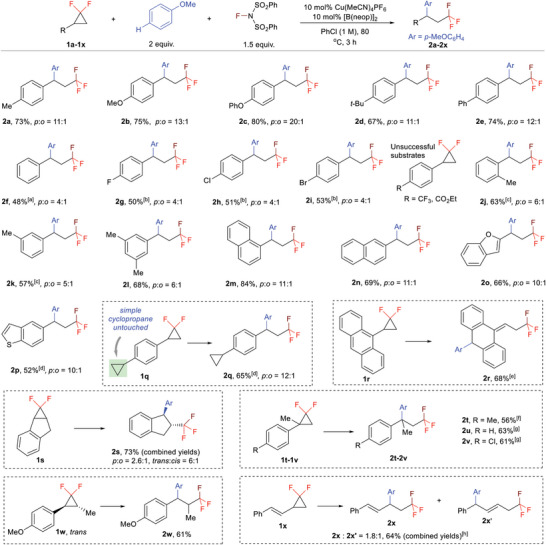
Substrate scope of *gem*‐DFCPs. a) Reaction conditions: **1a‐1x** (0.2 mmol), anisole (0.4 mmol), NFSI (0.3 mmol), [Cu] (10 mol%), [B] (10 mol%) in PhCl (200 µL) at 80 °C for 3 h unless otherwise noted. The *para*/*ortho* selectivity was not given if over 20:1; b) 10 mol% CuBr, 3 equiv. NFSI at 120 °C; c) 2 equiv. NFSI at 100 °C; d) 100 °C; e) 40 °C; f) 10 mol% CuBr, 3 equiv. NFSI in DCM (1 m) at 40 °C for 12 h; g) 10 mol% CuBr, 3 equiv. NFSI in DCM (1 m) at 60 °C for 12 h; h) 50 °C.

With the optimized conditions in hand, we proceeded to explore the scope of the Cu‐catalyzed fluoroarylation reaction. We initially investigated the reaction scope of *gem*‐DFCPs in combination with anisole as the nucleophilic coupling partner (**Scheme** **2**). Aryl *gem*‐DFCPs bearing electron‐donating groups (**2a**–**2d**) and a phenyl group (**2e**) exhibited favorable reactivity, leading to the formation of fluoroarylation products with good yields and *para*‐selectivity. Simple phenyl (**2f**) and electron‐deficient aryl groups (**2g**–**2i**) in *gem*‐DFCPs were tolerated under the optimized reaction conditions, giving the corresponding products in moderate yields with *para*‐selectivity of 4:1. Aryl *gem*‐DFCPs with strong electron‐deficient aryl groups (CF_3_, CO_2_Et) failed to form the desired products. The substrates with *ortho*‐ (**2j**) or *meta*‐substituted aryl moieties (**2k**) displayed satisfactory reactivity, providing the desired products with 63% and 57% yields, respectively. Di‐substituted aryl (**2l**) and naphthyl (**2m**, **2n**) substituted *gem*‐DFCPs work smoothly in this transformation. Note that *gem*‐DFCPs containing oxygen (**2o**) or sulfur (**2p**) heterocycle were also competent substrates in this reaction. An intriguing substrate is 4‐cyclopropyl aryl *gem*‐DFCP, which contains two cyclopropyl rings; it was found that only the *gem*‐difluorinated cyclopropane expressed the fluoroarylation reactivity under the Cu‐ catalyzed conditions, leaving the simple cyclopropyl ring untouched (**2q**). When 9‐anthracenyl *gem*‐DFCP (**1r**) was used as the substrate, an abnormal fluoro arylation product was formed in decent yield, in which the arylation occurs at the *para*‐position of the anthracyl moiety (**2r**). It was found that *gem*‐DFCP derived from indene provided the target product in a total yield of 73% with *para*/*ortho* selectivity of 2.6:1 and *trans*/*cis* selectivity of 6:1 under the optimized reaction conditions (**2s**). It is noteworthy that 1,1‐disubstituted *gem*‐DFCPs show satisfied reactivity toward fluoroarylation, resulting in the formation of CF_3_‐containing scaffolds with an all‐carbon quaternary center (**2t**‐**2v**). Similarly, the use of 1,3‐disubstituted *gem*‐DFCP provides the product with a substituent at the *α*‐position of the CF_3_ moiety (**2w**). Finally, the participation of styryl‐substituted *gem*‐DFCP in the reaction gives a pair of products in 64% total yields with a ratio of 1.8:1 (**2x**, **2x′**), in which a migration of the double bond occurs in the minor product.

**Scheme 3:**
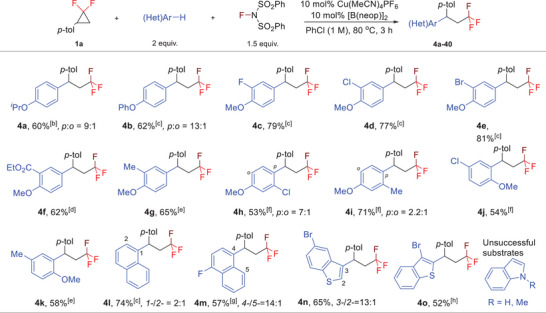
Substrate scope of areas. a) Reaction conditions: **1a** (0.2 mmol), arene (0.4 mmol), NFSI (0.3 mmol), [Cu] (10 mol%), [B] (10 mol%) in PhCl (200 µL) at 80 °C for 3 h unless otherwise noted. The regio‐selectivity was not given if over 20:1; b) 2 equiv. NFSI at 100 °C; c) 100 °C; d) 10 mol% CuBr in CHCl_3_ (1 m) at 100 °C; e) 10 mol% CuBr, 3 equiv. NFSI at 120 °C; f) 10 mol% CuBr, 3 equiv. NFSI at 100 °C; g) 10 mol% CuBr, in DCE (1 m) at 100 °C; h) DCE (0.75 m) at 100 °C.

Subsequently, we evaluated the reactivity of various arenes in our reaction (**Scheme** **3**). Substrates with bulkier alkoxyl (**4a**) and phenoxyl (**4b**) groups exhibit good regioselectivity and decent yields in the fluoroarylation reaction. The reaction of 2‐halogen‐substituted anisoles proceed smoothly, delivering the corresponding products (**4c**‐**4e**) with exclusive *para*‐selectivity. Likewise, anisole derivatives substituted with ester (**4f**) or methyl group (**4g**) at the *ortho*‐position yield the desired products in moderate yields with no other regio isomers observed. When *meta*‐substituted anisole derivatives were used as the substrates, the regio‐selectivity was decreased to some extent (**4h**, **4i**) with moderate to good yields. Note that the aryl C─H functionalization occurs at the *ortho*‐position of the methoxyl group (**4j**, **4k**) when the *para*‐position of the substrates was blocked. In addition, naphthalene derivatives (**4l**, **4m**) are competent nucleophiles in this transformation; while simple naphthalene provides the products with poor regioselectivity, excellent regioselectivity was observed in the reaction of fluoronaphthalene. Finally, we discovered that benzothiophene derivatives could serve as the nucleophiles to participate in the fluoroarylation reaction, in which the C─H functionalization mainly occurs at 3‐position (**4n**) or 2‐position (**4o**) if the 3‐position is substituted; however, more electron‐rich hetereoarenes (indole derivatives) were unsuccessful nucleophiles in this transformation, presumably due to their incompatibility with oxidative conditions.

**Scheme 4:**
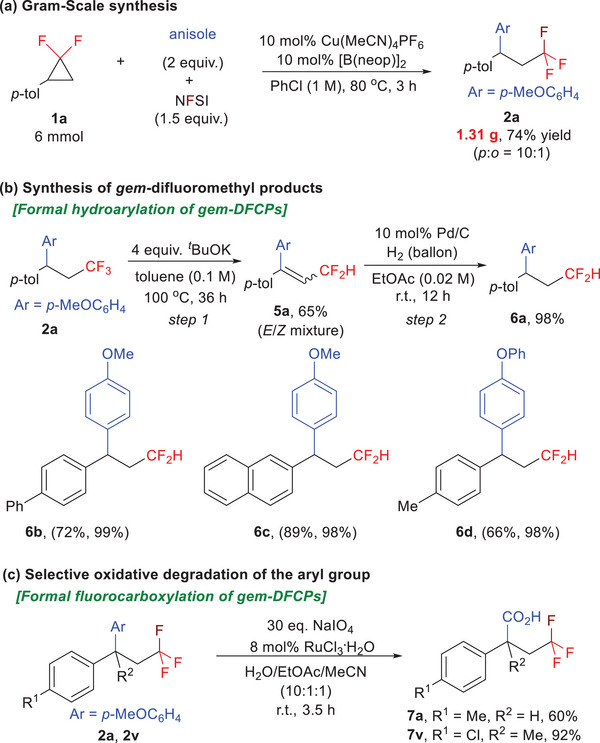
Synthetic applications.

The utility of our reaction was further demonstrated through a gram‐scale synthesis and various synthetic applications (**Scheme** **4**). First, we conducted the model reaction on a gram‐scale, which provides a 74% yield (1.31 g) of the desired product **2a** with good regioselectivity (*p*:*o* = 10:1) (Scheme 4a). Second, the resulting CF_3_ products can be smoothly converted into the corresponding CF_2_‐containing molecules. For example, treatment **2a** with potassium *tert*‐butoxide gives the HF eliminated intermediate **5a**, which then undergoes hydrogenation to deliver the difluoromethyl product **6a** in 98% yield. The two‐step sequence together with our model reaction provides an alternative method to access β, β‐diaryl difluoromethyl scaffolds (**6a‐6d**) (Scheme 4b). Furthermore, the electron‐rich aryl moiety in the products (**2a**, **2v**) can undergo chemoselective oxidative degradation in the presence of RuCl_3_/NaIO_4_, resulting in the formation of the corresponding carboxylic acids (**7a**, **7v**) with yields of 60% and 92%, respectively (Scheme 4c).^[^ ^18^ ^]^ These two types of downstream transformations realized formal hydroarylation and fluorocarboxylation of *gem*‐DFCPs, respectively, which significantly enriched the synthetic applications of *gem*‐DFCPs.

**Scheme 5:**
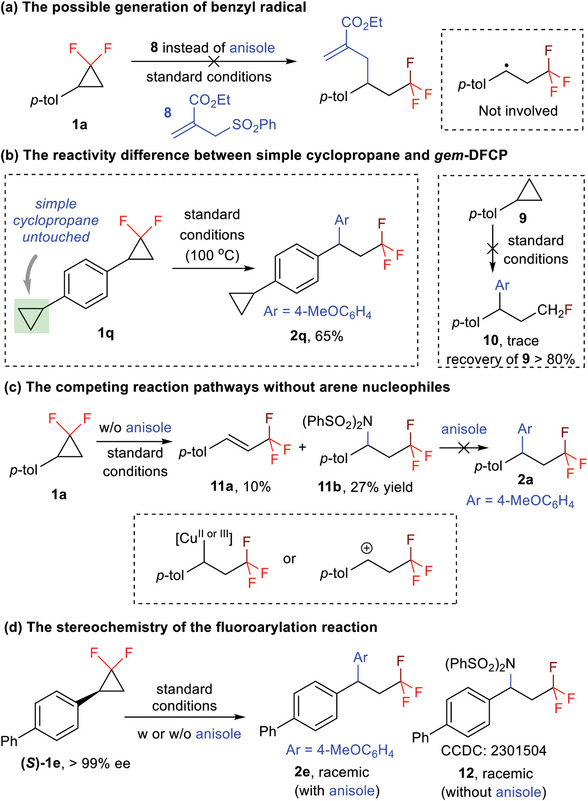
Control experiments.

To better understand the reaction mechanism of the copper‐catalyzed fluoroarylation transformation, a set of control experiments was then carried out. First, compound **8** was used instead of anisole under the copper‐catalyzed conditions, but failed to produce the allylation product. Such a control experiment indicates that a benzyl radical, which was proposed in visible‐light‐promoted radical‐type fluoroallylation reaction of electron‐rich aryl substituted *gem*‐DFCPs,^[^ ^12c^ ^]^ was not involved in this Cu‐catalyzed reaction (**Scheme** **5**a). Second, during the investigation of the reaction scope of *gem*‐DFCPs, it was found that our catalytic system has privileged reactivity on *gem*‐DFCP over simple cyclopropane (**1q** to **2q**). It is indeed that simple arylcyclopropane **9** hardly shows reactivity under the standard reaction conditions, and only a trace amount of fluoroarylation product **10** was obtained with **9** being recovered over 80% (Scheme 5b). Next, we investigated the fate of the model reaction by removing the arene nucleophile, and we detected the formation of eliminated trifluoromethyl product **11a** and fluoroamination product **11b** in 10% and 27% yields, respectively. This result indicates the possible involvement of a high‐valent benzyl‐copper species or a benzyl carbocation intermediate in the reaction, which undergoes elimination, C─N reductive elimination, or Friedel‐Crafts‐type alkylation to give **11a**, **11b,** and **2a**, respectively. Treatment of these two byproducts **11a** and **11b** under the standard reaction conditions failed to produce the original fluoroarylation product (Scheme 5c). Finally, enantiopure *gem*‐DFCP **1e** was used as the substrate to probe the stereochemistry of the arylation process under the standard reactions, and it was found only racemic fluoroarylation product **2e** was obtained. Besides, the corresponding aminoarylation product **12** was also racemic when the reaction was carried out in the absence of anisole. This observation further supports the involvement of the carbocation intermediate in the arylation step (Scheme 5d).

**Scheme 6:**
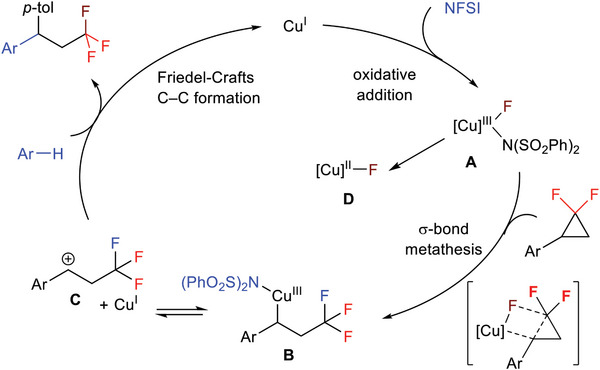
Proposed mechanism.

Based on these experimental observations and previous studies,^[^ ^16^ ^]^ we proposed a simplified mechanism for this reaction (**Scheme** **6**). Initially, Cu(I) is oxidized by NFSI to produce F─Cu(III)─N(PhSO_2_)_2_ species **A**, which is highly electrophilic and may activate the proximal C─C bond in *gem*‐DFCP possibly via a σ‐bond metathesis to form a benzyl─Cu(III) species **B**. Finally, complex **B** is equilibrated with the Cu(I) and benzyl cation species **C**,^[^ ^19^ ^]^ which is then captured by an electron‐rich arene via Friedel‐Crafts process to produce the fluoroarylation product. Meanwhile, it is also possible that the F─Cu(III)─N(PhSO_2_)_2_ species **A** undergoes comproportionation with the Cu(I) or releases a nitrogen radical to form a Cu(II)─F species **D**, which then triggers the C─C bond activation. Nevertheless, in addition to the proposed σ‐bond metathesis, we cannot rule out the possibility of a stepwise mechanism involving SET process to cleave the C─C bond (see[Supplementary-material advs7754-supitem-0001]Supporting Information for more details), and further inverstigations are required to better understand the details of the reaction mechanism.

## 3 Conclusion

In conclusion, we have developed a high‐valent copper‐catalyzed regioselective fluoroarylation of *gem‐*DFCPs with electron‐rich arenes as the C─H nucleophiles and NFSI as the electrophilic fluoro reagent. It is proposed that the regioselective congested proximal C─C bond activation proceeds via a concerted σ‐bond metathesis with a high‐valent Cu─F species accompanying the C─F bond formation, and the arylation occurs through a Friedel–Crafts process with the benzyl carbocation intermediate that is generated from the high‐valent benzyl‐copper species. The reaction expresses a broad substrate scope with respect to both *gem‐*DFCPs and simple arenes, enabling the synthesis of trifluoromethylated products to a great diversity. Furthermore, the successful gram‐scale synthesis and downstream transformations further validate the applicability of this methodology for accessing organofluorine molecules from *gem*‐DFCPs. This new reaction model not only broadens the synthetic applications of *gem*‐DFCPs in organic synthesis, but also provides new opportunities for exploring high‐valent TM in C─C bond activation.

## Conflict of Interest

The authors declare no conflict of interest.

## Supporting information

Supporting Information

## Data Availability

The data that support the findings of this study are available in the supplementary material of this article.
