# Supplementary material for: High‐Valent Copper Catalysis Enables Regioselective Fluoroarylation of Gem‐Difluorinated Cyclopropanes
Source: Adv Sci (Weinh). 2024 Mar 9;11(18):2401243. doi: 10.1002/advs.202401243 (PMC11095216; doi:10.1002/advs.202401243)

ADVANCED SCIENCE
Open Access

Supporting Information

for *Adv. Sci.*, DOI 10.1002/advs.202401243

High-Valent Copper Catalysis Enables Regioselective Fluoroarylation of *Gem*-Difluorinated Cyclopropanes

*Xiuli Wu, Xiangyu Song and Ying Xia**

Supporting Information

High-Valent Copper Catalysis Enables Regioselective Fluoroarylation of *gem*-Difluorinated Cyclopropanes

Xiuli Wu[a], Xiangyu Song[a] and Ying Xia[a]*

[a]***West China School of Public Health and West China Fourth Hospital, West China-PUMC C.C. Chen Institute of Health, and State Key Laboratory of Biotherapy, Sichuan University, Chengdu 610041, China***

Email: xiayingscu@scu.edu.cn

CONTENTS

1. General Information 1

2. Synthesis and Information of Substrates 2

3. General Experimental Procedures 5

4. Synthetic Applications 24

5. Experimental Mechanistic Investigations 30

6. Proposed Mechanism 38

7. References 40

8. NMR Spectra 41

1. General Information

All reagents and solvents are commercially available and used without further purification, unless specified noted. Reactions were conducted in a nitrogen atmosphere glovebox (Vigor). Heating reactions were carried out using a suitable pie-block with a heating magnetic stirrer. NMR spectra were recorded using a JEOL JNM-ECZ400S spectrometer (400 MHz for ^{1}H, 100 MHz for ^{13}C, 376 MHz for ^{19}F) at ambient in $CDCl_3$ with tetramethylsilane (TMS) as the internal standard. Chemical shifts were recorded in ppm relative to tetramethylsilane (TMS) (δ = 0.00 ppm), with the solvent resonance as an internal standard ($CDCl_3$: δ = 7.26 ppm for ^{1}H NMR or 77.00 for ^{13}C NMR). The coupling constant (*J*) was expressed in hertz (Hz). The following abbreviations were used to explain the multiplicities: s = singlet, d = doublet, t = triplet, q = quartet, m = multiplet. Gas chromatography mass spectrometry (GC-MS) analysis was performed using an Agilent Technologies 5975C instrument. High-resolution mass spectrometry employed a Q-Exactive Focus (Thermo Fisher Scientific), which combines quadrupole precursor ion selection and a high-resolution accurate-mass (HR/AM) Orbitrap mass analyzer. Enantiomeric excesses (ee) were determined by High Performance Liquid Chromatography (HPLC) analysis on Agilent HPLC 1260 with Daicel chiral columns. Melting points were determined using a digital melting point apparatus (JHX-4). Flash column chromatography purification for products was utilized with silica gel (200-300 mesh, Haiyang, Qingdao).

2. Synthesis and Information of Substrates

2.1 Synthesis of *gem*-Difluorinated Cyclopropanes (*gem*-DFCPs)

The structures of the *gem*-DFCPs used in this study were listed in **Table S1** and were prepared following a known procedure.[1-12]

Table S1. ***gem*-DFCPs (1a-1x) used in this work.**

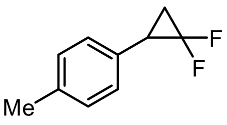

1a

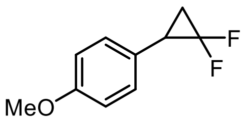

1b

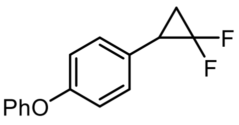

1c

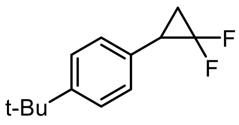

1d

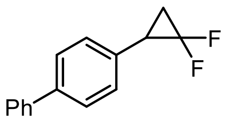

1e

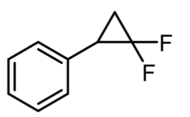

1f

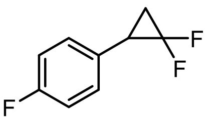

1g

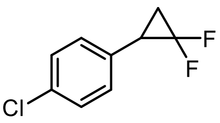

1h

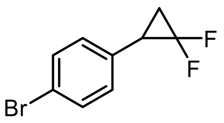

1i

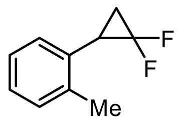

1g

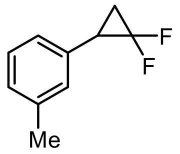

1k

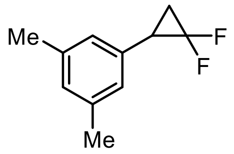

1l

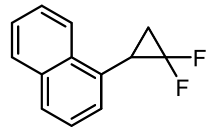

1m

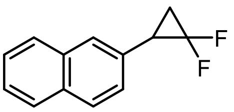

1n

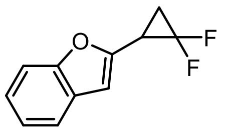

1o

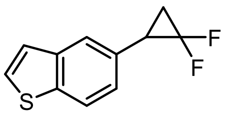

1p

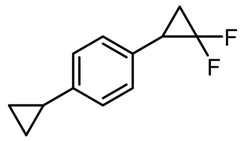

1q

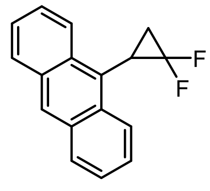

1r

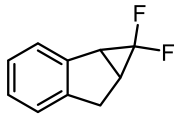

1s

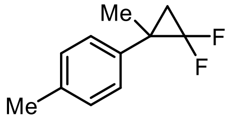

1t

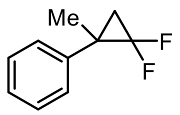

1u

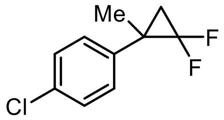

1v

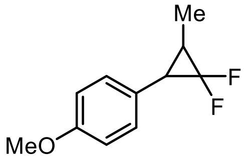

1w

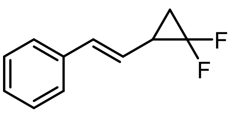

1x

<u>**The procedure for the preparation of *gem*-DFCPs:**</u>

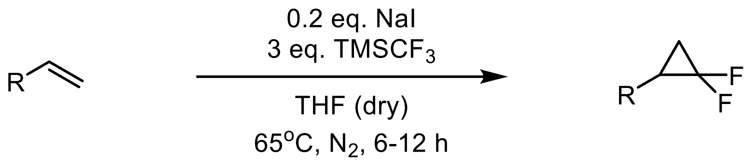

A mixture of NaI (0.15 g, 0.2 eq.) and corresponding alkenes (5.0 mmol, 1 eq.) in anhydrous THF (10 mL) was heat to 65 °C under nitrogen atmosphere. Then, $TMSCF_3$ (1.85 mL, 3 eq.) was added

dropwise over 3-5 h at this temperature. The reaction mixture was stirred at 65 °C for 6-12 h until completion. Afterward, the reaction mixture was concentrated, and the residue was carefully poured into a solution of saturated sodium sulfite (20 mL) and extracted with EtOAc (20 mL×3). The organic phase was then dried using anhydrous Na_2SO_4 and concentrated under vacuum. The resulting residue was purified by silica gel column chromatography (200-300 mesh) to obtain corresponding *gem*-DFCPs (**1a**-**1x**). Among them, **1a**-**1q** and **1s**-**1x** are literature reported compounds, the spectral data match the literature report.[1-9] *gem*-DFCPs **1r** is new compound, which is characterized as follows.

9-(2,2-difluorocyclopropyl)anthracene (1r)

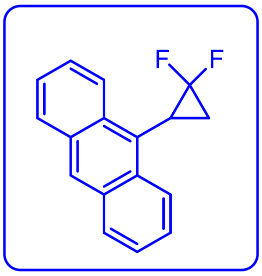

Following the general procedure, isolated yield of the product = 34% (432 mg); green-yellow solid; m.p.: 102.1-104.4 °C; R_f = 0.8 (PE). **^{1}H NMR** (400 MHz, $CDCl_3$) δ 8.44 (s, 1H), 8.34 (d, *J* = 8.8 Hz, 2H), 8.01 (d, *J* = 8.4 Hz, 2H), 7.55 (ddd, *J* = 8.9, 6.5, 1.4 Hz, 2H), 7.48 (ddd, *J* = 7.8, 6.5, 1.2 Hz, 2H), 3.39 – 3.30 (m, 1H), 2.45 – 3.36 (m, 1H), 1.76 – 1.68 (m, 1H). **^{13}C NMR** (101 MHz, $CDCl_3$) δ 131.5, 131.4, 131.3, 129.0, 127.8, 125.9, 125.2, 125.0, 113.2 (t, *J* = 284.0 Hz), 22.6 (t, *J* = 11.4 Hz), 19.2 (t, *J* = 10.8 Hz). **^{19}F NMR** (376 MHz, $CDCl_3$) δ -127.00 – -127.99 (m), -134.00 (ddd, *J* = 151.4, 12.8, 4.3 Hz). The HRMS was not satisfied, and the result of LRMS was obtained for this compound. **LRMS** (EI) m/z: 254 (M^+, 52), 239 (4), 233 (100), 202 (23), 117 (8), 101(10), 75 (2).

2.2 Arene Nucleophiles

Arene nucleophiles are commercially available reagents and listed in **Table S2**.

Table S2. Arenes (3a-3o) used in this work.

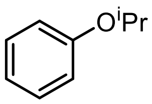

3a

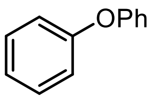

3b

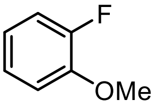

3c

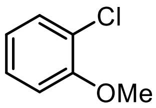

3d

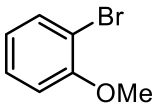

3e

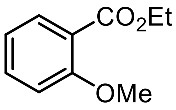

3f

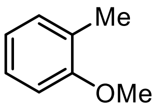

3g

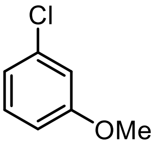

3h

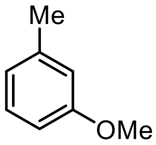

3i

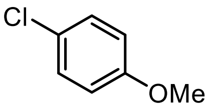

3j

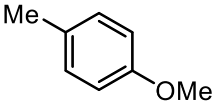

3k

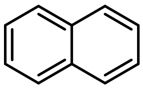

3l

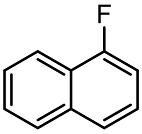

3m

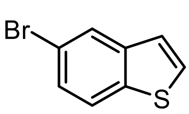

3n

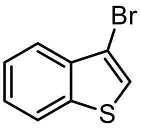

3o

3. General Experimental Procedures

3.1 General procedure:

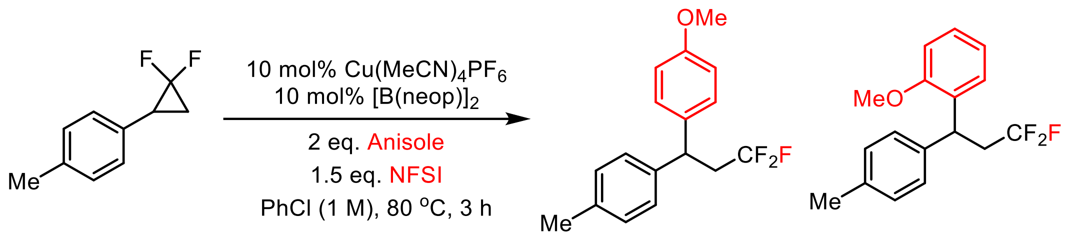

1a
0.2 mmol

2a
major

2a'
minor

A 1.5 mL vial equipped with stir bar was charged with **1a** (0.2 mmol), **anisole** (0.4 mmol, 2 eq.), **NFSI** (0.3 mmol, 1.5 eq.), $Cu(MeCN)_4PF_6$ (0.02 mmol, 0.1 eq.) and $[B(neop)]_2$ (0.02 mmol, 0.1 eq.) in dry PhCl (0.2 mL). Next, the 1.5 mL vial was sealed and removed from the glove box. After stirring at 80 °C for 3 h, the reaction was completed. Then, the reaction mixture was analyzed by ^{19}F NMR and ^{1}H NMR to measure the regioselectivity if necessary, and the product was isolated through chromatography on a silica gel column to give the product.

3.2 Characterization data of the products

1-methoxy-4-(3,3,3-trifluoro-1-(p-tolyl)propyl)benzene (2a)

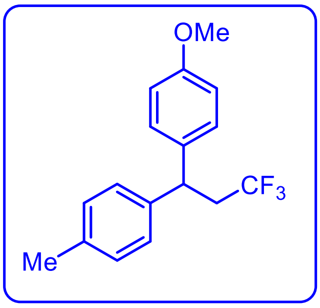

Following the general procedure, the reaction mixture was analyzed by ^{19}F NMR giving a ratio of 11:1 (*para/ortho*). Isolated yield of the major product = 73% (43.1 mg); colorless oil; R_f = 0.5 (PE: EtOAc =100:1). **^{1}H NMR** (400 MHz, $CDCl_3$) δ 7.12 (d, J = 8.8 Hz, 2H), 7.10 – 7.06 (m, 4H), 6.80 (d, J = 8.8 Hz, 2H), 4.23 (t, J = 7.4 Hz, 1H), 3.70 (s, 3H), 2.82 (qd, J = 10.5, 7.4 Hz, 2H), 2.26 (s, 3H). **^{13}C NMR** (101 MHz, $CDCl_3$) δ 158.3, 140.2, 136.2, 135.1, 129.3, 128.4, 127.2, 126.5 (q, J = 277.5 Hz), 114.0, 55.1, 43.8 (q, J = 2.8 Hz), 39.7 (q, J = 27.1 Hz), 20.9. **^{19}F NMR** (376 MHz, $CDCl_3$) δ -63.5 (t, J = 10.2 Hz). **HRMS** (ESI, *m/z*): $[M+Na]^+$ Calcd for $C_{17}H_{17}F_3NaO^+$ 371.1124, found 371.1123.

1-methoxy-2-(3,3,3-trifluoro-1-(p-tolyl)propyl)benzene (2a')

Following the general procedure, Isolated yield of the product = 6.6% (3.9 mg); colorless oil; R_f = 0.6

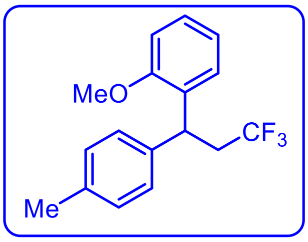

(PE: EtOAc =100:1). **^{1}H NMR** (400 MHz, $CDCl_3$) δ 7.20 – 7.13 (m, 4H), 7.08 (d, *J* = 8.0 Hz, 2H), 6.89 (td, *J* = 7.5, 1.2 Hz, 1H), 6.83 (dd, *J* = 8.1, 1.2 Hz, 1H), 4.71 (t, *J* = 7.3 Hz, 1H), 3.80 (s, 3H), 2.96 – 2.77 (m, 2H), 2.29 (s, 3H). **^{13}C NMR** (101 MHz, $CDCl_3$) δ 156.6, 139.3, 136.0, 131.4, 129.0, 127.9, 127.8, 127.7, 126.7 (q, *J* = 279.5 Hz), 120.6, 110.9, 55.4, 38.6 (q, *J* = 27.1 Hz), 38.2 (q, *J* = 2.8 Hz), 21.0. **^{19}F NMR** (376 MHz, $CDCl_3$) δ -63.90 (t, *J* = 10.8 Hz). **HRMS** (ESI, *m/z*): $[M+Na]^+$ Calcd for $C_{17}H_{17}F_3NaO^+$ 371.1124, found 371.1129.

4,4'-(3,3,3-trifluoropropane-1,1-diyl)bis(methoxybenzene) (2b)

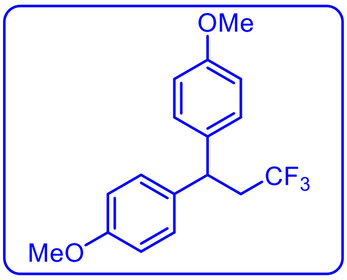

Following the general procedure, the reaction mixture was analyzed by ^{19}F NMR giving a ratio of 13:1 (*para/ortho*). Isolated yield of the major product = 75% (92.5 mg); colorless oil; R_f = 0.2 (PE: EtOAc =100:1). **^{1}H NMR** (400 MHz, $CDCl_3$) δ 7.13 (d, *J* = 8.7 Hz, 4H), 6.82 (d, *J* = 8.7 Hz, 4H), 4.23 (t, *J* = 7.4 Hz, 1H), 3.75 (s, 6H), 2.82 (qd, *J* = 10.5, 7.4 Hz, 2H). **^{13}C NMR** (101 MHz, $CDCl_3$) δ 158.2, 135.2, 128.3, 126.4 (q, *J* = 278.0 Hz), 114.0, 55.1, 43.4 (q, *J* = 2.0 Hz), 39.8 (q, *J* = 27.1 Hz). **^{19}F NMR** (376 MHz, $CDCl_3$) δ -63.47 (t, *J* = 10.7 Hz). **HRMS** (ESI, *m/z*): $[M+Na]^+$ Calcd for $C_{17}H_{17}F_3NaO_2^+$ 333.1073, found 333.1069.

1-methoxy-4-(3,3,3-trifluoro-1-(4-phenoxyphenyl)propyl)benzene (2c)

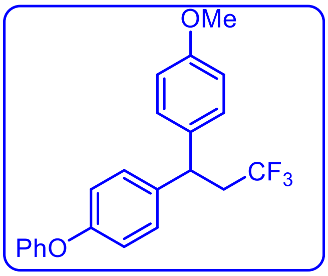

Following the general procedure, the reaction mixture was analyzed by ^{19}F NMR giving a ratio of 20:1 (*para/ortho*). Isolated yield of the major product = 80% (59.9 mg); colorless oil; R_f = 0.2 (PE: EtOAc =100:1). **^{1}H NMR** (400 MHz, $CDCl_3$) δ 7.31 – 7.27 (m, 2H), 7.17 – 7.13 (m, 4H), 7.09 – 7.04 (m, 1H), 6.98 – 6.95 (m, 2H), 6.92 (d, *J* = 8.7 Hz, 2H), 6.83 (d, *J* = 8.7 Hz, 2H), 4.26 (t, *J* = 7.4 Hz, 1H), 3.74 (s, 3H), 2.83 (qd, *J* = 10.4, 7.5 Hz, 2H). **^{13}C NMR** (101 MHz, $CDCl_3$) δ 158.3, 157.1, 155.9, 137.9, 134.9, 129.7, 128.6, 128.4, 126.4 (q, *J* = 277.7 Hz), 123.2, 118.9, 118.8, 114.1, 55.1, 43.6 (q, *J* = 2.8 Hz), 39.8 (q, *J* = 27.1 Hz). **^{19}F NMR** (376 MHz, $CDCl_3$) δ -63.40 (t, *J* = 10.3 Hz). **HRMS** (ESI, *m/z*): $[M+Na]^+$ Calcd for $C_{22}H_{19}F_3NaO_2^+$ 395.1229, found 395.1233.

1-(tert-butyl)-4-(3,3,3-trifluoro-1-(4-methoxyphenyl)propyl)benzene (2d)

Following the general procedure, the reaction mixture was analyzed by ^{19}F NMR giving a ratio of 11:1 (*para/ortho*). Isolated yield of the major product = 67% (45.2 mg); colorless oil; R_f = 0.5 (PE: EtOAc

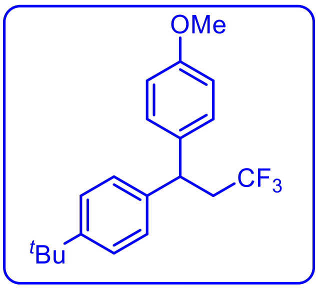

=100:1). **^{1}H NMR** (400 MHz, $CDCl_3$) δ 7.29 (d, *J* = 8.4 Hz, 2H), 7.17 – 7.13 (m, 4H), 6.82 (d, *J* = 8.7 Hz, 2H), 4.24 (t, *J* = 7.3 Hz, 1H), 3.74 (s, 3H), 2.89 – 2.79 (m, 2H), 1.27 (s, 9H). **^{13}C NMR** (101 MHz, $CDCl_3$) δ 158.3, 149.4, 140.2, 135.0, 128.5, 126.9, 125.5, 126.5 (q, *J* = 277.6 Hz), 114.0, 55.1, 43.8 (q, *J* = 2.8 Hz), 39.8 (q, *J* = 27.1 Hz), 34.3, 31.3. **^{19}F NMR** (376 MHz, $CDCl_3$) δ -63.52 (t, *J* = 10.7 Hz). **HRMS** (ESI, *m/z*): $[M+Na]^+$ Calcd for $C_{20}H_{23}F_3NaO^+$ 359.1593, found 359.1590.

4-(3,3,3-trifluoro-1-(4-methoxyphenyl)propyl)-1,1'-biphenyl (2e)

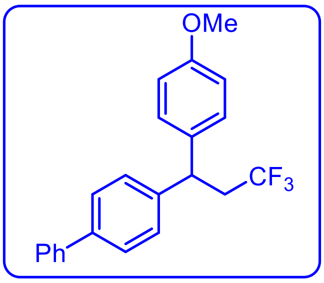

Following the general procedure, the reaction mixture was analyzed by ^{19}F NMR giving a ratio of 12:1 (*para/ortho*). Isolated yield of the major product = 74% (52.7 mg); white solid; m.p.: 91.6-93.7 °C; R_f = 0.3 (PE: EtOAc =100:1). **^{1}H NMR** (400 MHz, $CDCl_3$) δ 7.55 – 7.50 (m, 4H), 7.42 – 7.38 (m, 2H), 7.33 – 7.27 (m, 3H), 7.18 (d, *J* = 8.7 Hz, 2H), 6.84 (d, *J* = 8.7 Hz, 2H), 4.31 (t, *J* = 7.4 Hz, 1H), 3.74 (s, 3H), 2.88 (qd, *J* = 10.4, 7.3 Hz, 2H). **^{13}C NMR** (101 MHz, $CDCl_3$) δ 158.4, 142.2, 140.6, 139.6, 134.8, 128.7, 128.5, 127.7, 127.4, 127.2, 127.0, 126.4 (q, *J* = 277.8 Hz),114.1, 55.2, 43.9 (q, *J* = 3.0 Hz), 39.7 (q, *J* = 27.3 Hz). **^{19}F NMR** (376 MHz, $CDCl_3$) δ -63.48 (t, *J* = 10.8 Hz). **HRMS** (ESI, *m/z*): $[M+Na]^+$ Calcd for $C_{22}H_{19}F_3NaO^+$ 379.1280, found 379.1276.

1-methoxy-4-(3,3,3-trifluoro-1-phenylpropyl)benzene (2f)

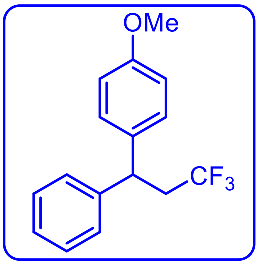

Following the general procedure (except the reaction was carried out with 10 mol% CuBr, 3eq. NFSI at 120 °C), the reaction mixture was analyzed by ^{19}F NMR giving a ratio of 4:1 (*para/ortho*). Isolated yield of the major product = 48% (26.9 mg); colorless oil; R_f = 0.4 (PE: EtOAc =100:1). **^{1}H NMR** (400 MHz, $CDCl_3$) δ 7.30 – 7.26 (m, 2H), 7.23 – 7.18 (m, 3H), 7.15 (d, *J* = 8.7 Hz, 2H), 6.82 (d, *J* = 8.8 Hz, 2H), 4.27 (t, *J* = 7.4 Hz, 1H), 3.74 (s, 3H), 2.85 (qd, *J* = 10.4, 7.4 Hz, 2H). **^{13}C NMR** (101 MHz, $CDCl_3$) δ 158.3, 143.1, 134.9, 128.7, 128.4, 127.4, 126.7, 126.4 (q, *J* = 277.9 Hz), 114.0, 55.2, 44.2 (q, *J* = 2.8 Hz), 39.7 (q, *J* = 27.2 Hz). **^{19}F NMR** (376 MHz, $CDCl_3$) δ -63.52 (t, *J* = 10.2 Hz). **HRMS** (ESI, *m/z*): $[M+Na]^+$ Calcd for $C_{16}H_{15}F_3NaO^+$ 303.0967, found 303.0970.

1-fluoro-4-(3,3,3-trifluoro-1-(4-methoxyphenyl)propyl)benzene (2g)

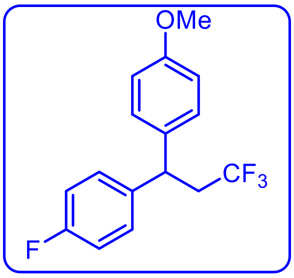

Following the general procedure (except the reaction was carried out with 10 mol% CuBr, 3 eq. NFSI at 120 °C), the reaction mixture was analyzed by ^{19}F NMR giving a ratio of 4:1 (*para/ortho*). Isolated yield of the major product = 50% (27.0 mg); colorless oil; R_f = 0.4 (PE: EtOAc =100:1). **^{1}H NMR** (400 MHz, $CDCl_3$) δ 7.20 – 7.11 (m, 2H), 7.13 (dd, *J* = 8.8, 2.2 Hz, 2H), 7.00 – 6.95 (m, 2H), 6.84 (dd, *J* = 8.8, 2.4 Hz, 2H), 4.27 (t, *J* = 7.0 Hz, 1H), 3.77 (s, 3H), 2.88 – 2.78 (m, 2H). **^{13}C NMR** (101 MHz, $CDCl_3$) δ 161.6 (d, *J* = 245.3 Hz), 158.4, 138.8 (d, *J* = 3.3 Hz), 134.6, 128.9 (d, *J* = 7.9 Hz), 128.3, 126.3 (q, *J* = 277.7 Hz), 115.5 (d, *J* = 21.3 Hz), 114.1, 55.2, 43.5 (q, *J* = 2.8 Hz), 39.8 (q, *J* = 27.3 Hz). **^{19}F NMR** (376 MHz, $CDCl_3$) δ -63.49 (t, *J* = 10.2 Hz), -116.03 – -116.11 (m). The HRMS was not satisfied, and the result of LRMS was obtained for this compound. **LRMS** (EI) m/z: 298 (M+, 36), 299 (7), 215 (100), 183 (20), 171(29), 107(4), 77 (2).

1-chloro-4-(3,3,3-trifluoro-1-(4-methoxyphenyl)propyl)benzene (2h)

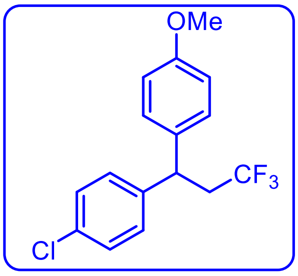

Following the general procedure (except the reaction was carried out with 10 mol% CuBr, 3 eq. NFSI at 120 °C), the reaction mixture was analyzed by ^{19}F NMR giving a ratio of 4:1 (*para/ortho*). Isolated yield of the major product = 51% (32.0 mg); colorless oil; R_f = 0.4 (PE: EtOAc =100:1). **^{1}H NMR** (400 MHz, $CDCl_3$) δ 7.26 (d, *J* = 8.6 Hz, 2H), 7.15 (d, *J* = 8.4 Hz, 2H), 7.12 (d, *J* = 8.7 Hz, 2H), 6.83 (d, *J* = 8.7 Hz, 2H), 4.25 (t, *J* = 7.4 Hz, 1H), 3.77 (s, 3H), 2.83 (qd, *J* = 10.3, 7.4 Hz, 2H). **^{13}C NMR** (101 MHz, $CDCl_3$) δ 158.5, 141.6, 134.3, 132.5, 128.81, 128.75, 128.3, 126.3 (q, *J* = 277.6 Hz), 114.2, 55.2, 43.6 (q, *J* = 2.7 Hz), 39.6 (q, *J* = 27.4 Hz). **^{19}F NMR** (376 MHz, $CDCl_3$) δ -63.49 (d, *J* = 10.2 Hz). The HRMS was not satisfied, and the result of LRMS was obtained for this compound. **LRMS** (EI) m/z: 314 (M+, 34), 316 (34), 231 (100), 196 (28), 181 (19), 153 (29), 77 (3).

1-bromo-4-(3,3,3-trifluoro-1-(4-methoxyphenyl)propyl)benzene (2i)

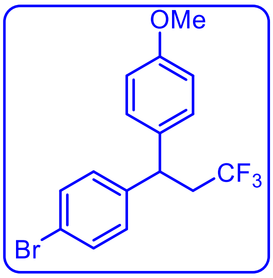

Following the general procedure (except the reaction was carried out with 10 mol% CuBr, 3 eq. NFSI at 120 °C), the reaction mixture was analyzed by ^{19}F NMR giving a ratio of 4:1 (*para/ortho*). Isolated yield of the major product = 53% (38.0 mg); colorless oil; R_f = 0.3 (PE: EtOAc =100:1). **^{1}H NMR** (400 MHz, $CDCl_3$) δ 7.39 (d, *J* = 8.5 Hz, 2H), 7.11 – 7.07 (m, 4H), 6.82 (d, *J* = 8.8 Hz, 2H), 4.23 (t, *J* = 7.4 Hz, 1H), 3.74 (s, 3H), 2.81 (qd, *J* = 10.4, 7.4 Hz, 2H). **^{13}C NMR** (101 MHz, $CDCl_3$) δ 158.5, 142.1, 134.2, 131.7, 129.1, 128.3, 126.3 (q, *J* = 277.8 Hz), 120.6, 114.1, 55.2, 43.7 (q,

J = 2.6 Hz), 39.4 (q, J = 27.4 Hz). **^{19}F NMR** (376 MHz, $CDCl_3$) δ -63.41 (t, J = 10.1 Hz). The HRMS was not satisfied, and the result of LRMS was obtained for this compound. **LRMS** (EI) m/z: 358 (M+, 34), 360 (34), 275 (100), 196 (32), 181 (26), 153 (38), 77 (4).

1-methyl-2-(3,3,3-trifluoro-1-(4-methoxyphenyl)propyl)benzene (2j)

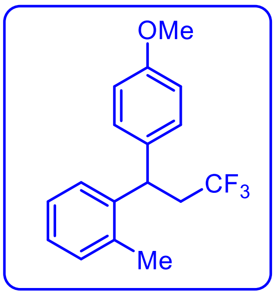

Following the general procedure (except the reaction was carried out with 2 eq. NFSI at 100 °C), the reaction mixture was analyzed by ^{19}F NMR giving a ratio of 6:1 (*para/ortho*). Isolated yield of the major product = 63% (37.2 mg); colorless oil; R_f = 0.3 (PE: EtOAc =100:1). **^{1}H NMR** (400 MHz, $CDCl_3$) δ 7.25 – 7.17 (m, 2H), 7.14 – 7.11 (m, 4H), 6.81 (d, J = 8.7 Hz, 2H), 4.50 (t, J = 7.3 Hz, 1H), 3.74 (s, 3H), 2.88 – 2.78 (m, 2H), 2.31 (s, 3H). **^{13}C NMR** (101 MHz, $CDCl_3$) δ 158.2, 141.0, 135.7, 134.2, 130.9, 128.9, 126.6, 126.5 (q, J = 277.8 Hz), 126.20, 126.16, 113.9, 55.1, 39.9 (q, J = 27.1 Hz), 39.6 (q, J = 2.8 Hz), 19.7. **^{19}F NMR** (376 MHz, $CDCl_3$) δ -63.57 (t, J = 10.3 Hz). **HRMS** (ESI, *m/z*): $[M+Na]^+$ Calcd for $C_{17}H_{17}F_3NaO^+$ 317.1124, found 317.1121.

1-methyl-3-(3,3,3-trifluoro-1-(4-methoxyphenyl)propyl)benzene (2k)

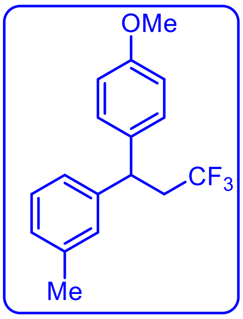

Following the general procedure (except the reaction was carried out with 2 eq. NFSI at 100 °C), the reaction mixture was analyzed by ^{19}F NMR giving a ratio of 5:1 (*para/ortho*). Isolated yield of the major product = 57% (33.7 mg); colorless oil; R_f = 0.3 (PE: EtOAc =100:1). **^{1}H NMR** (400 MHz, $CDCl_3$) δ 7.18 – 7.13 (m, 3H), 7.02 – 6.98 (m, 3H), 6.81 (d, J = 8.7 Hz, 2H), 4.23 (t, J = 7.4 Hz, 1H), 3.73 (s, 3H), 2.84 (qd, J = 10.5, 7.3 Hz, 2H), 2.29 (s, 3H). **^{13}C NMR** (101 MHz, $CDCl_3$) δ 158.3, 143.1, 138.3, 135.0, 128.5, 128.4, 128.2, 127.5, 126.5 (q, J = 277.8 Hz), 124.3, 114.0, 55.1, 44.2 (q, J = 2.6 Hz), 39.7 (q, J = 27.3 Hz), 21.4. **^{19}F NMR** (376 MHz, $CDCl_3$) δ -63.49 (t, J = 10.8 Hz). **HRMS** (ESI, *m/z*): $[M+Na]^+$ Calcd for $C_{17}H_{17}F_3NaO^+$ 317.1124, found 317.1122.

1,3-dimethyl-5-(3,3,3-trifluoro-1-(4-methoxyphenyl)propyl)benzene (2l)

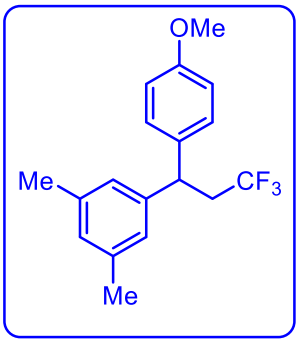

Following the general procedure, the reaction mixture was analyzed by ^{19}F NMR giving a ratio of 6:1 (*para/ortho*). Isolated yield of the major product = 68% (42.1 mg); colorless oil; R_f = 0.3 (PE: EtOAc =100:1). **^{1}H NMR** (400 MHz, $CDCl_3$) δ 7.15 (d, J = 8.7 Hz, 2H), 6.82 (s, 3H), 6.81 (d, J = 8.7 Hz, 2H), 4.18 (t, J = 7.4 Hz, 1H), 3.73 (s, 3H), 2.82 (qd, J = 10.5, 7.4 Hz, 2H), 2.25 (s, 6H). **^{13}C NMR** (101 MHz, $CDCl_3$) δ 158.2, 143.1, 138.1, 135.1,

128.41, 128.36, 125.13 (d, *J* = 277.7 Hz), 125.11, 114.0, 55.1, 44.1 (q, *J* = 2.8 Hz), 39.7 (q, *J* = 27.1 Hz), 21.3. **^{19}F NMR** (376 MHz, $CDCl_3$) δ -63.51 (t, *J* = 10.3 Hz). **HRMS** (ESI, *m/z*): $[M+Na]^+$ Calcd for $C_{18}H_{19}F_3NaO^+$ 331.1280, found 331.1277.

1-(3,3,3-trifluoro-1-(4-methoxyphenyl)propyl)naphthalene (2m)

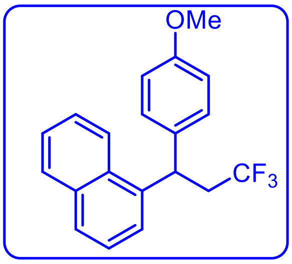

Following the general procedure, the reaction mixture was analyzed by ^{19}F NMR giving a ratio of 11:1 (*para/ortho*). Isolated yield of the major product = 84% (55.7 mg); colorless oil; R_f = 0.4 (PE: EtOAc =100:1). **^{1}H NMR** (400 MHz, $CDCl_3$) δ 8.13 – 8.10 (m, 1H), 7.83 – 7.81 (m, 1H), 7.74 – 7.72 (m, 1H), 7.50 – 7.41 (m, 3H), 7.37 – 7.31 (m, 1H), 7.21 (d, *J* = 8.7 Hz, 2H), 6.79 (d, *J* = 8.8 Hz, 2H), 5.12 (t, *J* = 7.2 Hz, 1H), 3.70 (s, 3H), 3.03 – 2.90 (m, 2H). **^{13}C NMR** (101 MHz, $CDCl_3$) δ 158.3, 138.6, 134.2, 134.1, 131.0, 129.0, 128.9, 127.6, 126.5 (q, *J* = 277.9 Hz), 126.4, 125.6, 125.2, 124.2, 123.1, 113.9, 55.1, 39.9 (q, *J* = 27.2 Hz), 39.3 (q, *J* = 3.1 Hz). **^{19}F NMR** (376 MHz, $CDCl_3$) δ -63.28 (t, *J* = 10.8 Hz). **HRMS** (ESI, *m/z*): $[M+Na]^+$ Calcd for $C_{20}H_{17}F_3NaO^+$ 353.1124, found 353.1125.

2-(3,3,3-trifluoro-1-(4-methoxyphenyl)propyl)naphthalene (2n)

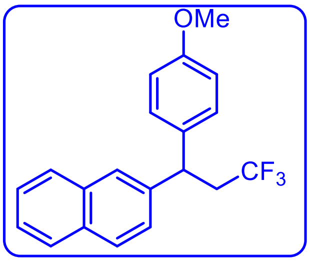

Following the general procedure, the reaction mixture was analyzed by ^{19}F NMR giving a ratio of 11:1 (*para/ortho*). Isolated yield of the major product = 69% (45.6 mg); colorless oil; R_f = 0.4 (PE: EtOAc =100:1). **^{1}H NMR** (400 MHz, $CDCl_3$) δ 7.79 – 7.73 (m, 3H), 7.67 (s, 1H), 7.47 – 7.39 (m, 2H), 7.30 (dd, *J* = 8.5, 1.9 Hz, 1H), 7.20 – 7.17 (m, 2H), 6.82 (d, *J* = 8.7 Hz, 2H), 4.44 (t, *J* = 7.4 Hz, 1H), 3.73 (s, 3H), 3.05 – 2.86 (m, 2H). **^{13}C NMR** (101 MHz, $CDCl_3$) δ 158.4, 140.5, 134.7, 133.4, 132.2, 128.6, 128.5, 127.7, 127.6, 126.5 (d, *J* = 277.8 Hz), 126.2, 125.9, 125.8, 125.5, 114.0, 55.1, 44.2 (q, *J* = 3.0 Hz), 39.5 (q, *J* = 27.3 Hz). **^{19}F NMR** (376 MHz, $CDCl_3$) δ -63.30 (t, *J* = 10.3 Hz). **HRMS** (ESI, *m/z*): $[M+Na]^+$ Calcd for $C_{20}H_{17}F_3NaO^+$ 353.1124, found 353.1122.

2-(3,3,3-trifluoro-1-(4-methoxyphenyl)propyl)benzofuran (2o)

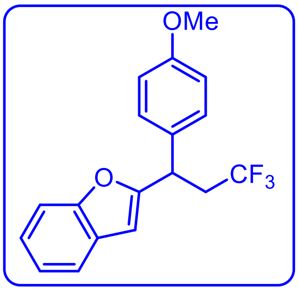

Following the general procedure. the reaction mixture was analyzed by ^{19}F NMR giving a ratio of 10:1 (*para/ortho*). Isolated yield of the major product = 66% (42.2 mg); colorless oil; R_f = 0.3 (PE: EtOAc =100:1). **^{1}H NMR** (400 MHz, $CDCl_3$) δ 7.45 (dd, *J* = 7.6, 1.7 Hz, 1H), 7.40 (dd, *J* = 8.0, 1.7 Hz, 1H), 7.23 (d, *J* = 8.7 Hz, 2H), 7.20 – 7.14 (m, 2H), 6.85 (d, *J* = 8.7 Hz, 2H), 6.39

(s, 1H), 4.40 (t, J = 7.3 Hz, 1H), 3.74 (s, 3H), 3.08 (dqd, J = 15.0, 10.5, 6.8 Hz, 1H), 2.74 (dqd, J = 15.0, 10.3, 7.8 Hz, 1H). **^{13}C NMR** (101 MHz, $CDCl_3$) δ 158.9, 158.2, 154.8, 131.6, 128.8, 128.3, 126.1 (q, J = 277.6 Hz), 123.9, 122.7, 120.7, 114.2, 111.0, 103.1, 55.2, 39.0 (q, J = 3.1 Hz), 38.4 (q, J = 27.9 Hz). **^{19}F NMR** (376 MHz, $CDCl_3$) δ -63.96 (t, J = 10.2 Hz). **HRMS** (ESI, *m/z*): $[M+Na]^+$ Calcd for $C_{18}H_{15}F_3NaO_2^+$ 343.0916, found 343.0916.

5-(3,3,3-trifluoro-1-(4-methoxyphenyl)propyl)benzo[b]thiophene (2p)

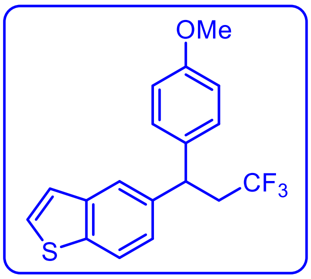

Following the general procedure (except the reaction was carried out at 100 °C), the reaction mixture was analyzed by ^{19}F NMR giving a ratio of 10:1 (*para/ortho*). Isolated yield of the major product = 52% (35.1 mg); colorless oil; R_f = 0.2 (PE: EtOAc =100:1). **^{1}H NMR** (400 MHz, $CDCl_3$) δ 7.77 (d, J = 8.4 Hz, 1H), 7.67 (d, J = 1.9 Hz, 1H), 7.39 (d, J = 5.5 Hz, 1H), 7.26 (dd, J = 5.5, 0.8 Hz, 1H), 7.21 – 7.17 (m, 3H), 6.83 (d, J = 8.8 Hz, 2H), 4.41 (t, J = 7.4 Hz, 1H), 3.74 (s, 3H), 2.92 (qd, J = 10.4, 7.4 Hz, 2H). **^{13}C NMR** (101 MHz, $CDCl_3$) δ 158.3, 139.9, 139.4, 138.1, 135.0, 128.4, 127.0, δ 126.5 (q, J = 277.7 Hz), 124.1, 123.8, 122.7, 122.0, 114.1, 55.2, 44.1 (q, J = 2.8 Hz), 39.8 (q, J = 27.3 Hz). **^{19}F NMR** (376 MHz, $CDCl_3$) δ -63.35 (t, J = 10.2 Hz). **HRMS** (ESI, *m/z*): $[M+Na]^+$ Calcd for $C_{18}H_{15}F_3NaOS^+$ 359.0688, found 359.0685.

1-cyclopropyl-4-(3,3,3-trifluoro-1-(4-methoxyphenyl)propyl)benzene (2q)

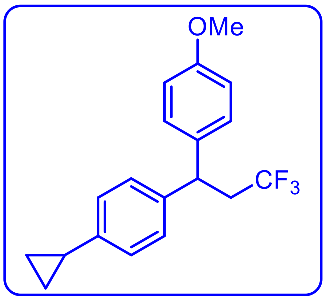

Following the general procedure (except the reaction was carried out at 100 °C), the reaction mixture was analyzed by ^{19}F NMR giving a ratio of 12:1 (*para/ortho*). Isolated yield of the major product = 65% (41.8 mg); colorless oil; R_f = 0.3 (PE: EtOAc =100:1). **^{1}H NMR** (400 MHz, $CDCl_3$) δ 7.13 (d, J = 8.7 Hz, 2H), 7.10 (d, J = 8.2 Hz, 2H), 6.98 (d, J = 8.3 Hz, 2H), 6.81 (d, J = 8.8 Hz, 2H), 4.23 (t, J = 7.4 Hz, 1H), 3.73 (s, 3H), 2.82 (qd, J = 10.5, 7.4 Hz, 2H), 1.84 – 1.80 (m, 1H), 0.93 – 0.88 (m, 2H), 0.65 – 0.60 (m, 2H). **^{13}C NMR** (101 MHz, $CDCl_3$) δ 158.3, 142.4, 140.2, 135.1, 128.4, 127.2, 126.5 (q, J = 277.6 Hz), 125.9, 114.0, 55.1, 43.8 (q, J = 2.7 Hz), 39.7 (q, J = 27.2 Hz), 14.9, 9.1. **^{19}F NMR** (376 MHz, $CDCl_3$) δ -63.48 (t, J = 10.7 Hz). **HRMS** (ESI, *m/z*): $[M+Na]^+$ Calcd for $C_{19}H_{19}F_3NaO^+$ 343.1280, found 343.1282.

9-(4-methoxyphenyl)-10-(3,3,3-trifluoropropylidene)-4a,8a,9,9a,10,10a-hexahydroanthracene (2r)

Following the general procedure (except the reaction was carried out at 40 °C). Isolated yield of the

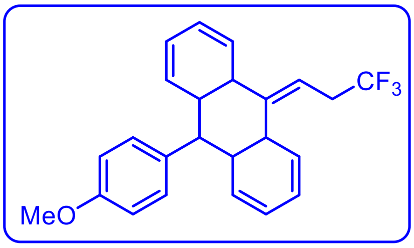

major product = 68% (51.6 mg); colorless oil; R_f = 0.2 (PE: EtOAc =100:1). **^{1}H NMR** (400 MHz, $CDCl_3$) δ 7.64 – 7.62 (m, 1H), 7.47 – 7.45 (m, 1H), 7.43 – 7.41 (m, 1H), 7.37 – 7.28 (m, 5H), 7.09 (d, *J* = 8.8 Hz, 2H), 6.79 (d, *J* = 8.8 Hz, 2H), 6.08 (dd, *J* = 8.2, 6.5 Hz, 1H), 5.21 (s, 1H), 3.75 (s, 3H), 3.51 – 3.39 (m, 2H). **^{13}C NMR** (101 MHz, $CDCl_3$) δ 158.2, 141.1, 140.9, 138.6, 138.0, 136.0, 133.5, 128.7, 128.4, 128.0, 127.60, 127.55, 127.1, 127.0, 126.2, 124.1, 123.5 (d, *J* = 277.0 Hz), 115.2 (q, *J* = 3.8 Hz), 113.9, 55.1, 51.4, 34.8 (q, *J* = 29.5 Hz). **^{19}F NMR** (376 MHz, $CDCl_3$) δ -65.61 (t, *J* = 10.4 Hz). The HRMS was not satisfied, and the result of LRMS was obtained for this compound. **LRMS** (EI) m/z: 380 (M+, 100), 349 (20), 297 (56), 273 (32), 233 (16), 203 (56), 77 (2).

Trans-1-(4-methoxyphenyl)-2-(trifluoromethyl)-2,3-dihydro-1H-indene (2s)

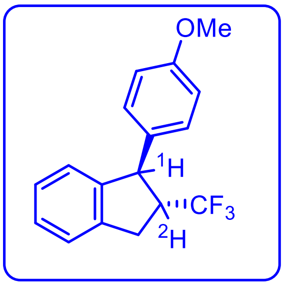

Following the general procedure, the reaction mixture was analyzed by ^{19}F NMR giving a ratio of 2.6:1 (*para/ortho*) and 6:1 (dr). Isolated yield of the para products = 53% (31.0 mg); white soild; m.p.: 85.4-86.3 °C; R_f = 0.3 (PE: EtOAc =100:1). **^{1}H NMR** (400 MHz, $CDCl_3$) δ 7.28 – 7.25 (m, 1H), 7.23 – 7.19 (m, 1H), 7.18 – 7.13 (m, 1H), 7.08 (d, *J* = 8.7 Hz, 2H), 6.89 – 6.84 (m, 3H), 4.50 (d, *J* = 7.8 Hz, 1H), 3.78 (s, 3H), 3.33 – 3.26 (m, 1H), 3.21 – 3.07 (m, 2H). **^{13}C NMR** (101 MHz, $CDCl_3$) δ 158.6, 144.8, 140.2, 135.3, 129.3, 127.4, 127.3, 126.8 (q, *J* = 278.2), 125.2, 124.2, 114.0, 55.2, 52.4 (q, *J* = 26.1 Hz), 51.2 (q, *J* = 2.5 Hz), 32.2 (q, *J* = 2.8 Hz). **^{19}F NMR** (376 MHz, $CDCl_3$) δ -69.90 (d, *J* = 8.6 Hz). **HRMS** (ESI, *m/z*): $[M+Na]^+$ Calcd for $C_{17}H_{15}F_3NaO^+$ 315.0967, found 315.0970. The structure was analyzed through HMQC, which indicates that the ^{1}H and ^{2}H atoms are in a trans position.

HMQC spectrum of 2s

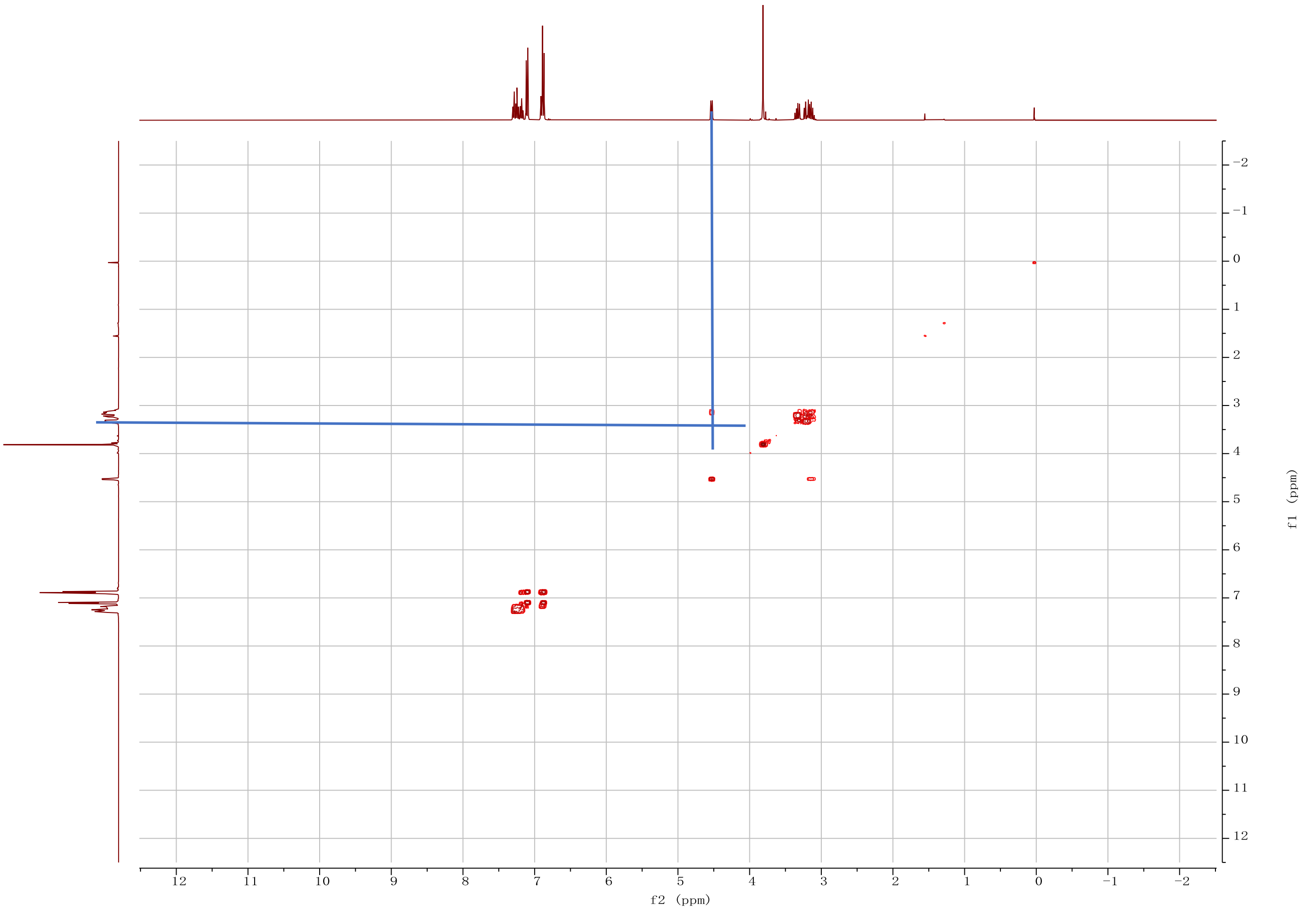

Trans-1-(2-methoxyphenyl)-2-(trifluoromethyl)-2,3-dihydro-1H-indene (2s')

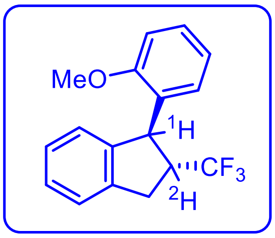

Isolated yield of the ortho products = 20% (11.7 mg); colorless oil; R_f = 0.4 (PE: EtOAc =100:1). **^{1}H NMR** (400 MHz, $CDCl_3$) δ 7.26 – 7.21 (m, 2H), 7.20 – 7.17 (m, 1H), 7.14 – 7.10 (m, 1H), 6.96 (dd, *J* = 7.5, 1.8 Hz, 1H), 6.91 – 6.86 (m, 3H), 4.94 (d, *J* = 6.7 Hz, 1H), 3.74 (s, 3H), 3.35 – 3.15 (m, 3H). **^{13}C NMR** (101 MHz, $CDCl_3$) δ 157.4, 144.7, 140.4, 131.3, 129.2, 128.2, 128.1 (q, *J* = 278.2 Hz), 127.0, 126.9, 124.5, 124.1, 120.7, 111.1, 55.5, 50.3 (q, *J* = 26.4 Hz), 45.9, 32.4 (q, *J* = 2.9 Hz). **^{19}F NMR** (376 MHz, $CDCl_3$) δ -70.71 (d, *J* = 8.6 Hz). **HRMS** (ESI, *m/z*): $[M+Na]^+$ Calcd for $C_{17}H_{15}F_3NaO^+$ 315.0967, found 315.0968. The structure was analyzed through HMQC, which indicates that the ^{1}H and ^{2}H atoms are in a trans position.

HMQC spectrum of 2s'

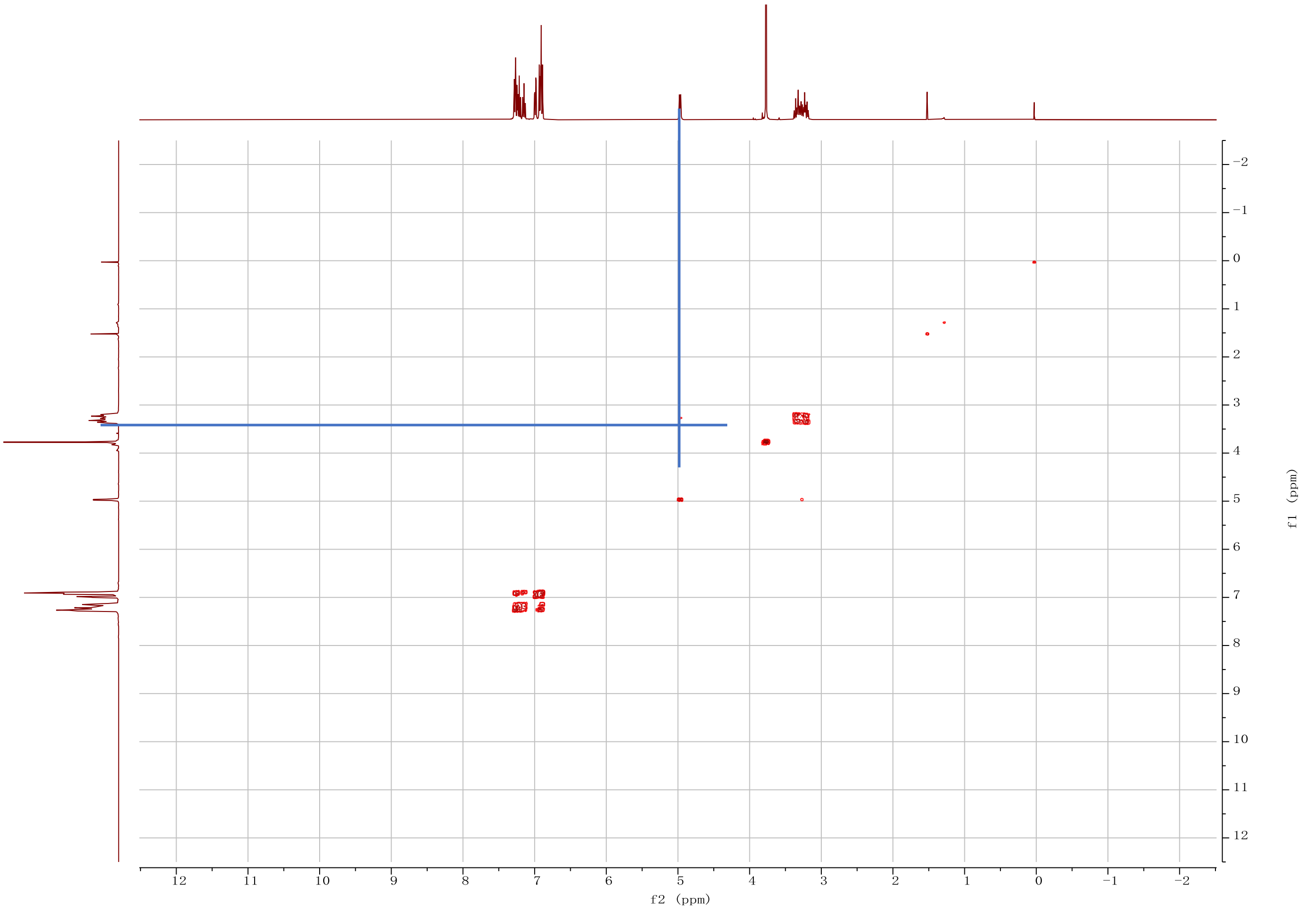

1-methoxy-4-(4,4,4-trifluoro-2-(p-tolyl)butan-2-yl)benzene (2t)

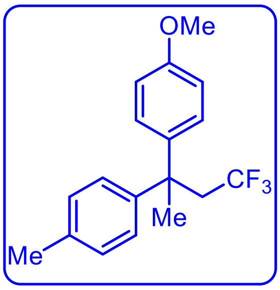

Following the general procedure (except the reaction was carried out with 10 mol% CuBr, 3 eq. NFSI in DCM (1M) at 40 °C for 12 h). Isolated yield of the major product = 56% (34.5 mg); colorless oil; R_f = 0.4 (PE: EtOAc =100:1). **^{1}H NMR** (400 MHz, $CDCl_3$) δ 7.11 – 7.05 (m, 6H), 6.81 (d, *J* = 8.9 Hz, 2H), 3.78 (s, 3H), 2.97 (q, *J* = 10.9 Hz, 2H), 2.31 (s, 3H), 1.79 (q, *J* = 1.3 Hz, 3H). **^{13}C NMR** (101 MHz, $CDCl_3$) δ 157.8, 145.1, 139.8, 135.8, 128.8, 127.9, 126.64, 126.57 (q, *J* = 279.1 Hz), 113.4, 55.1, 44.6 (q, *J* = 25.7 Hz), 42.8, 27.7, 20.8. **^{19}F NMR** (376 MHz, $CDCl_3$) δ -58.22 (t, *J* = 10.9 Hz). **HRMS** (ESI, *m/z*): $[M+Na]^+$ Calcd for $C_{18}H_{19}F_3NaO^+$ 331.1280, found 331.1278.

1-methoxy-4-(4,4,4-trifluoro-2-phenylbutan-2-yl)benzene (2u)

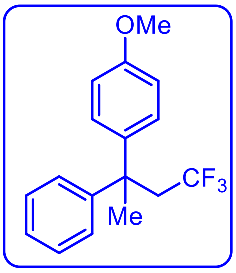

Following the general procedure (except the reaction was carried out with 10 mol% CuBr, 3 eq. NFSI in DCM (1M) at 60 °C for 12 h). Isolated yield of the major product = 63% (37.0 mg); colorless oil; R_f = 0.4 (PE: EtOAc =100:1). **^{1}H NMR** (400 MHz, $CDCl_3$) δ 7.30 – 7.25 (m, 2H), δ 7.21 – 7.16 (m, 3H), 7.09 (d, *J* = 8.9 Hz, 2H), 6.81 (d, *J* = 8.9 Hz, 2H), 3.77 (s, 3H), 2.99 (qd, *J* = 10.9, 1.2 Hz, 2H), 1.81 (q, *J* = 1.4 Hz, 3H). **^{13}C NMR** (101 MHz, $CDCl_3$) δ 157.8, 148.0, 139.7, 128.1, 128.0, 127.5 (q,

J = 277.8 Hz), 126.8, 126.2, 113.4, 55.1, 44.6 (q, J = 25.8 Hz), 43.2, 27.7 (d, J = 2.2 Hz). **^{19}F NMR** (376 MHz, $CDCl_3$) δ -58.21 (t, J = 10.8 Hz). **^{19}F NMR** (376 MHz, $CDCl_3$) δ -58.22 (t, J = 10.9 Hz). **HRMS** (ESI, *m/z*): $[M+Na]^+$ Calcd for $C_{17}H_{17}F_3NaO^+$ 317.1124, found 317.1123.

1-chloro-4-(4,4,4-trifluoro-2-(4-methoxyphenyl)butan-2-yl)benzene (2v)

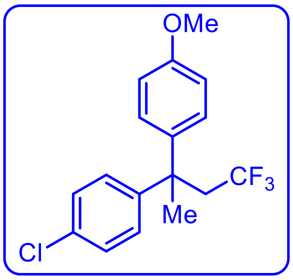

Following the general procedure (except the reaction was carried out with 10 mol% CuBr, 3 eq. NFSI in DCM (1M) at 60 °C for 12 h). Isolated yield of the major product = 61% (40.1 mg); colorless oil; R_f = 0.4 (PE: EtOAc =100:1). **^{1}H NMR** (400 MHz, $CDCl_3$) δ 7.24 (d, J = 8.7 Hz, 2H), 7.11 (d, J = 8.7 Hz, 2H), 7.06 (d, J = 8.9 Hz, 2H), 6.81 (d, J = 8.9 Hz, 2H), 3.77 (s, 3H), 2.95 (q, J = 10.8 Hz, 2H), 1.78 (s, 3H). **^{13}C NMR** (101 MHz, $CDCl_3$) δ 158.0, 146.4, 139.3, 132.1, 128.3, 128.2, 127.8, 126.4 (q, J = 278.9 Hz), 113.6, 55.2, 44.6 (q, J = 26.0 Hz), 42.9, 27.7 (d, J = 2.1 Hz). **^{19}F NMR** (376 MHz, $CDCl_3$) δ -58.24 (t, J = 10.8 Hz). **HRMS** (ESI, *m/z*): $[M+Na]^+$ Calcd for $C_{17}H_{16}{}^{35}ClF_3NaO^+$ 351.0734, found 351.0734.

4,4'-(3,3,3-trifluoro-2-methylpropane-1,1-diyl)bis(methoxybenzene) (2w)

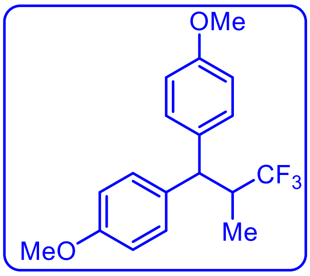

Following the general procedure, the reaction mixture was analyzed by ^{19}F NMR giving a ratio of 11:1 (*para/ortho*). Isolated yield of the major product = 61% (39.7 mg); colorless oil; R_f = 0.2 (PE: EtOAc =100:1). **^{1}H NMR** (400 MHz, $CDCl_3$) δ 7.19 (d, J = 8.7 Hz, 2H), 7.14 (d, J = 8.7 Hz, 2H), 6.82 – 6.78 (m, 4H), 3.96 (d, J = 10.0 Hz, 1H), 3.73 (s, 3H), 3.72 (s, 3H), 3.08 (dq, J = 15.6, 7.8, 7.3 Hz, 1H), 1.05 (d, J = 7.0 Hz, 3H). **^{13}C NMR** (101 MHz, $CDCl_3$) δ 158.2, 158.0, 135.2, 134.4, 129.1, 128.5, 128.1 (q, J = 281.1 Hz), 114.0, 113.8, 55.1 (d, J = 4.9 Hz), 50.8, 41.8 (q, J = 24.3 Hz), 12.9 (q, J = 3.2 Hz). **^{19}F NMR** (376 MHz, $CDCl_3$) δ -68.27 (d, J = 7.3 Hz). **HRMS** (ESI, *m/z*): $[M+Na]^+$ Calcd for $C_{18}H_{19}F_3NaO_2{}^+$ 347.1229, found 347.1232.

(E)-1-methoxy-4-(5,5,5-trifluoro-1-phenylpent-1-en-3-yl)benzene (2x)

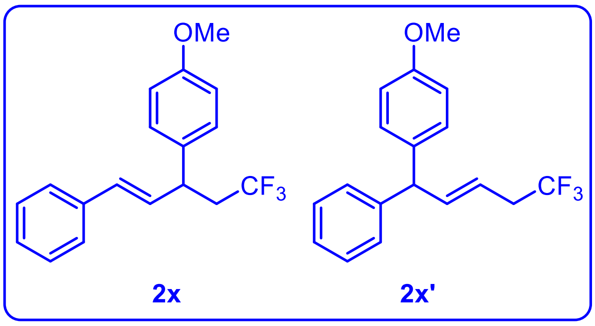

Following the general procedure (except the reaction was carried out at 50 °C), the reaction mixture was analyzed by ^{19}F NMR giving a ratio of 1.8:1 (***2x/2x'***). Isolated yield of the two isomers = 64% (39.1 mg); colorless oil; R_f = 0.3 (PE: EtOAc =100:1). **^{1}H NMR** (400 MHz, $CDCl_3$) δ 7.34 – 7.26 (m, 4H), 7.16 (d, J

= 8.7 Hz, 2H), 6.88 (d, J = 8.7 Hz, 2H), 6.40 (d, J = 15.9 Hz, 1H), 6.29 (dd, J = 15.8, 7.4 Hz, 1H), 3.82 (t, J = 7.3 Hz, 1H), 3.79 (s, 3H), 2.69 – 2.50 (m, 2H). **^{13}C NMR** (101 MHz, $CDCl_3$) δ 158.5, 136.9, 134.0, 131.5, 130.3, 128.5, 128.3, 127.5, 126.4 (q, J = 277.8 Hz), 126.3, 114.2, 55.2, 42.1 (q, J = 2.8 Hz), 39.8 (q, J = 26.9 Hz). **^{19}F NMR** (376 MHz, $CDCl_3$) δ -63.23 (t, J = 10.2 Hz). **HRMS** (ESI, *m/z*): $[M+Na]^+$ Calcd for $C_{18}H_{17}F_3NaO^+$ 329.1124, found 329.1120.

1-isopropoxy-4-(3,3,3-trifluoro-1-(p-tolyl)propyl)benzene (4a)

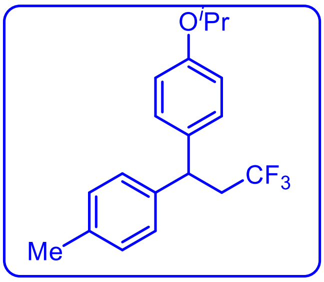

Following the general procedure (except the reaction was carried out with 2 eq. NFSI at 100 °C), the reaction mixture was analyzed by ^{19}F NMR giving a ratio of 9:1 (*para/ortho*). Isolated yield of the major product = 60% (38.7 mg); colorless oil; R_f = 0.3 (PE: EtOAc =100:1). **^{1}H NMR** (400 MHz, $CDCl_3$) δ 7.12 – 7.07 (m, 6H), 6.79 (d, J = 8.7 Hz, 2H), 4.47 (hept, J = 6.1 Hz, 1H), 4.22 (t, J = 7.4 Hz, 1H), 2.82 (m, 2H), 2.28 (s, 3H), 1.28 (d, J = 6.1 Hz, 6H). **^{13}C NMR** (101 MHz, $CDCl_3$) δ 156.6, 140.2, 136.2, 134.9, 129.3, 128.3, 127.2, 126.5 (q, J = 277.8 Hz), 115.9, 69.7, 43.8 (q, J = 2.8 Hz), 39.7 (q, J = 27.1 Hz), 22.0, 20.9. **^{19}F NMR** (376 MHz, $CDCl_3$) δ -63.49 (t, J = 10.5 Hz). **HRMS** (ESI, *m/z*): $[M+Na]^+$ Calcd for $C_{19}H_{21}F_3NaO^+$ 345.1437, found 345.1435.

1-methyl-4-(3,3,3-trifluoro-1-(4-phenoxyphenyl)propyl)benzene (4b)

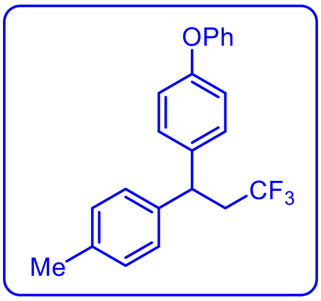

Following the general procedure (except the reaction was carried out at 100 °C), the reaction mixture was analyzed by ^{19}F NMR giving a ratio of 13:1 (*para/ortho*). Isolated yield of the major product = 62% (44.2 mg); colorless oil; R_f = 0.4 (PE: EtOAc =100:1). **^{1}H NMR** (400 MHz, $CDCl_3$) δ 7.32 – 7.28 (m, 2H), 7.18 (d, J = 8.6 Hz, 2H), 7.15 – 7.06 (m, 5H), 6.97 (d, J = 8.7 Hz, 2H), 6.92 (d, J = 8.7 Hz, 2H), 4.27 (t, J = 7.4 Hz, 1H), 2.90 – 2.81 (m, 2H), 2.30 (s, 3H). **^{13}C NMR** (101 MHz, $CDCl_3$) δ 157.1, 155.9, 139.8, 137.8, 136.4, 129.7, 129.4, 128.7, 127.2, 126.4 (q, J = 277.8 Hz), 123.2, 118.94, 118.86, 44.0 (q, J = 2.9 Hz), 39.7 (q, J = 27.1 Hz), 21.0. **^{19}F NMR** (376 MHz, $CDCl_3$) δ -63.50 (t, J = 10.7 Hz). **HRMS** (ESI, *m/z*): $[M+Na]^+$ Calcd for $C_{22}H_{19}F_3NaO^+$ 379.1280, found 379.1277.

2-fluoro-1-methoxy-4-(3,3,3-trifluoro-1-(p-tolyl)propyl)benzene (4c)

Following the general procedure (except the reaction was carried out at 100 °C). Isolated yield of the

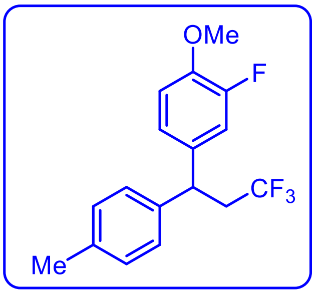

major product = 79% (49.4 mg); colorless oil; R_f = 0.2 (PE: EtOAc =100:1). **^{1}H NMR** (400 MHz, $CDCl_3$) δ 7.12 – 7.07 (m, 4H), 6.97 – 6.91 (m, 2H), 6.89 – 6.82 (m, 1H), 4.21 (t, *J* = 7.4 Hz, 1H), 3.82 (s, 3H), 2.81 (qd, *J* = 10.4, 7.4 Hz, 2H), 2.29 (s, 3H). **^{13}C NMR** (101 MHz, $CDCl_3$) δ 152.3 (d, *J* = 246.1 Hz), 146.3 (d, *J* = 10.9 Hz), 139.5, 136.6, 136.1 (d, *J* = 5.5 Hz), 129.5, 127.1, 126.3 (q, *J* = 277.7 Hz), 123.0 (d, *J* = 3.5 Hz), 115.2 (d, *J* = 18.8 Hz), 113.4 (d, *J* = 2.3 Hz), 56.2, 43.7, 39.5 (q, *J* = 27.4 Hz), 20.9. **^{19}F NMR** (376 MHz, $CDCl_3$) δ -63.51 (t, *J* = 10.3 Hz), -134.35 (dd, *J* = 12.2, 8.8 Hz). **HRMS** (ESI, *m/z*): $[M+Na]^+$ Calcd for $C_{17}H_{16}F_4NaO^+$ 335.1029, found 335.1030.

2-chloro-1-methoxy-4-(3,3,3-trifluoro-1-(p-tolyl)propyl)benzene (4d)

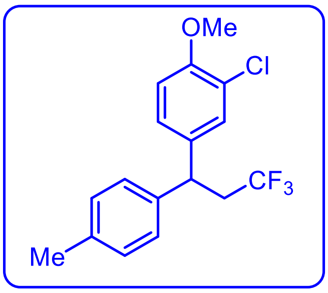

Following the general procedure (except the reaction was carried out at 100 °C). Isolated yield of the major product = 77% (50.3 mg); colorless oil; R_f = 0.2 (PE: EtOAc =100:1). **^{1}H NMR** (400 MHz, $CDCl_3$) δ 7.24 – 7.23 (m, 1H), 7.10 – 7.07 (m, 5H), 6.84 (d, *J* = 8.5 Hz, 1H), 4.21 (t, *J* = 7.4 Hz, 1H), 3.84 (s, 3H), 2.82 (qd, *J* = 10.4, 7.1 Hz, 2H), 2.30 (s, 3H). **^{13}C NMR** (101 MHz, $CDCl_3$) δ 153.7, 139.4, 136.6, 136.2, 129.5, 129.2, 127.1, 126.6, 126.3 (q, *J* = 278.8 Hz), 122.5, 112.1, 56.1, 43.6 (q, *J* = 3.0 Hz), 39.5 (q, *J* = 27.4 Hz), 20.9. **^{19}F NMR** (376 MHz, $CDCl_3$) δ -63.50 (t, *J* = 10.7 Hz). **HRMS** (ESI, *m/z*): $[M+Na]^+$ Calcd for $C_{17}H_{16}{}^{35}ClF_3NaO^+$ 351.0734, found 351.0738.

2-bromo-1-methoxy-4-(3,3,3-trifluoro-1-(p-tolyl)propyl)benzene (4e)

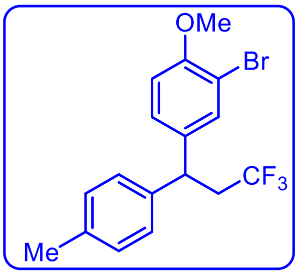

Following the general procedure (except the reaction was carried out at 100 °C). Isolated yield of the major product = 81% (60.3 mg); colorless oil; R_f = 0.2 (PE: EtOAc =100:1). **^{1}H NMR** (400 MHz, $CDCl_3$) δ 7.41 (d, *J* = 2.3 Hz, 1H), 7.13 (dd, *J* = 8.5, 2.3 Hz, 1H), 7.11 – 7.08 (m, 4H), 6.80 (d, *J* = 8.5 Hz, 1H), 4.21 (t, *J* = 7.4 Hz, 1H), 3.83 (s, 3H), 2.82 (qd, *J* = 10.4, 7.1 Hz, 2H), 2.30 (s, 3H). **^{13}C NMR** (101 MHz, $CDCl_3$) δ 154.6, 139.4, 136.61, 136.57, 132.2, 129.5, 127.4, 127.1, 126.3 (q, *J* = 277.7 Hz), 111.9, 111.7, 56.1, 43.5 (q, *J* = 2.9 Hz), 39.5 (q, *J* = 27.4 Hz), 20.9. **^{19}F NMR** (376 MHz, $CDCl_3$) δ -63.46 (t, *J* = 10.8 Hz). **HRMS** (ESI, *m/z*): $[M+Na]^+$ Calcd for $C_{17}H_{16}{}^{79}BrF_3NaO^+$ 395.0229, found 395.0227.

Ethyl 2-methoxy-5-(3,3,3-trifluoro-1-(p-tolyl)propyl)benzoate (4f)

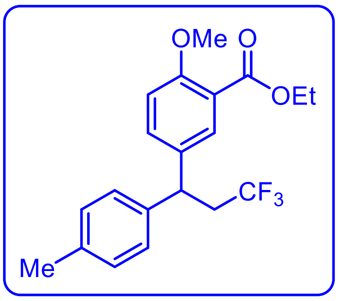

Following the general procedure (except the reaction was carried out with 10 mol% CuBr, $CHCl_3$ (1 M) at 100 °C). Isolated yield of the major product = 62% (45.4 mg); colorless oil; R_f = 0.5 (PE: EtOAc =100:1). **^{1}H NMR** (400 MHz, $CDCl_3$) δ 7.68 (d, J = 2.5 Hz, 1H), 7.31 (dd, J = 8.7, 2.5 Hz, 1H), 7.11 (s, 4H), 6.89 (d, J = 8.6 Hz, 1H), 4.35 (q, J = 7.1 Hz, 2H), 4.27 (t, J = 7.4 Hz, 1H), 3.84 (s, 3H), 2.86 (qd, J = 10.4, 7.4 Hz, 2H), 2.29 (s, 3H), 1.37 (t, J = 7.1 Hz, 3H). **^{13}C NMR** (101 MHz, $CDCl_3$) δ 166.1, 157.8, 139.5, 136.5, 134.6, 132.2, 130.3, 129.4, 127.2, 126.3 (q, J = 277.6 Hz), 120.4, 112.4, 60.9, 56.0, 43.6 (q, J = 2.7 Hz), 39.5 (q, J = 27.4 Hz), 20.9, 14.2. **^{19}F NMR** (376 MHz, $CDCl_3$) δ -63.47 (t, J = 10.7 Hz). **HRMS** (ESI, *m/z*): $[M+Na]^+$ Calcd for $C_{20}H_{21}F_3NaO_3^+$ 389.1335, found 389.1333.

1-methoxy-2-methyl-4-(3,3,3-trifluoro-1-(p-tolyl)propyl)benzene (4g)

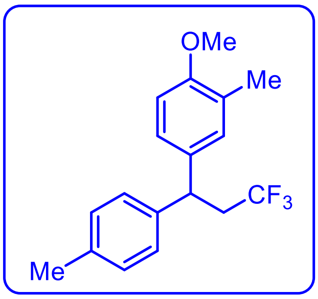

Following the general procedure (except the reaction was carried out with 10 mol% CuBr, 3 eq. NFSI at 120 °C). Isolated yield of the major product = 65% (32.0 mg); colorless oil; R_f = 0.4 (PE: EtOAc =100:1). **^{1}H NMR** (400 MHz, $CDCl_3$) δ 7.13 – 7.07 (m, 4H), 7.02 – 6.98 (m, 2H), 6.72 (d, J = 8.2 Hz, 1H), 4.20 (t, J = 7.4 Hz, 1H), 3.75 (s, 3H), 2.83 (qd, J = 10.5, 7.4 Hz, 2H), 2.28 (s, 3H), 2.17 (s, 3H). **^{13}C NMR** (101 MHz, $CDCl_3$) δ 156.5, 140.4, 136.2, 134.7, 129.7, 129.3, 127.2, 126.8, 126.5 (q, J = 277.7 Hz), 125.4, 109.8, 55.2, 43.9 (q, J = 2.8 Hz), 39.7 (q, J = 27.1 Hz), 20.9, 16.3. **^{19}F NMR** (376 MHz, $CDCl_3$) δ -63.47 (t, J = 10.2 Hz). **HRMS** (ESI, *m/z*): $[M+Na]^+$ Calcd for $C_{18}H_{19}F_3NaO^+$ 331.1280, found 331.1283.

2-chloro-4-methoxy-1-(3,3,3-trifluoro-1-(p-tolyl)propyl)benzene (4h)

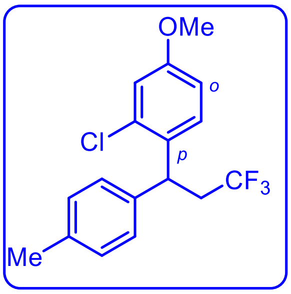

Following the general procedure (except the reaction was carried out with 10 mol% CuBr, 3 eq. NFSI at 100 °C), the reaction mixture was analyzed by ^{19}F NMR giving a ratio of 7:1 (*para/ortho*). Isolated yield of the major product = 53% (34.8 mg); colorless oil; R_f = 0.2 (PE: EtOAc =100:1). **^{1}H NMR** (400 MHz, $CDCl_3$) δ 7.16 – 7.08 (m, 5H), 6.89 (d, J = 2.7 Hz, 1H), 6.77 (dd, J = 8.7, 2.7 Hz, 1H), 4.81 (t, J = 7.4 Hz, 1H), 3.73 (s, 3H), 2.87 – 2.77 (m, 2H), 2.29 (s, 3H). **^{13}C NMR** (101 MHz, $CDCl_3$) δ 158.6, 138.6, 136.5, 134.0, 132.3, 129.3, 128.8, 127.5, 126.3 (q, J = 277.8 Hz), 115.0, 113.3, 55.4, 39.7 (q, J = 2.8 Hz), 39.0 (q, J = 27.5 Hz), 20.9. **^{19}F NMR** (376 MHz, $CDCl_3$) δ -63.70 (t, J = 10.2 Hz). **HRMS** (ESI, *m/z*): $[M+Na]^+$ Calcd for $C_{17}H_{16}{}^{35}ClF_3NaO^+$

351.0734, found 351.0737. The structure confirmed through the HMBC and the similar structure was also observed in the literature.[9]

HMBC spectrum of 4h

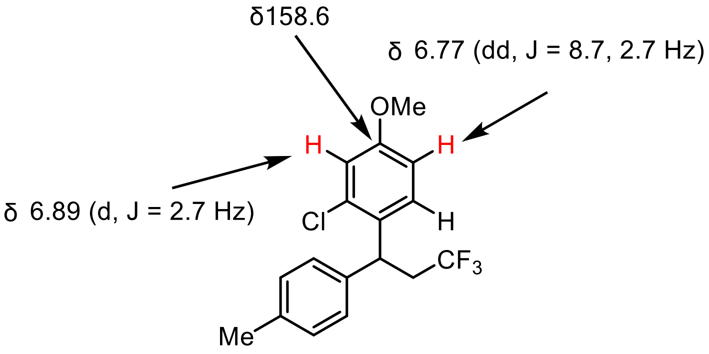

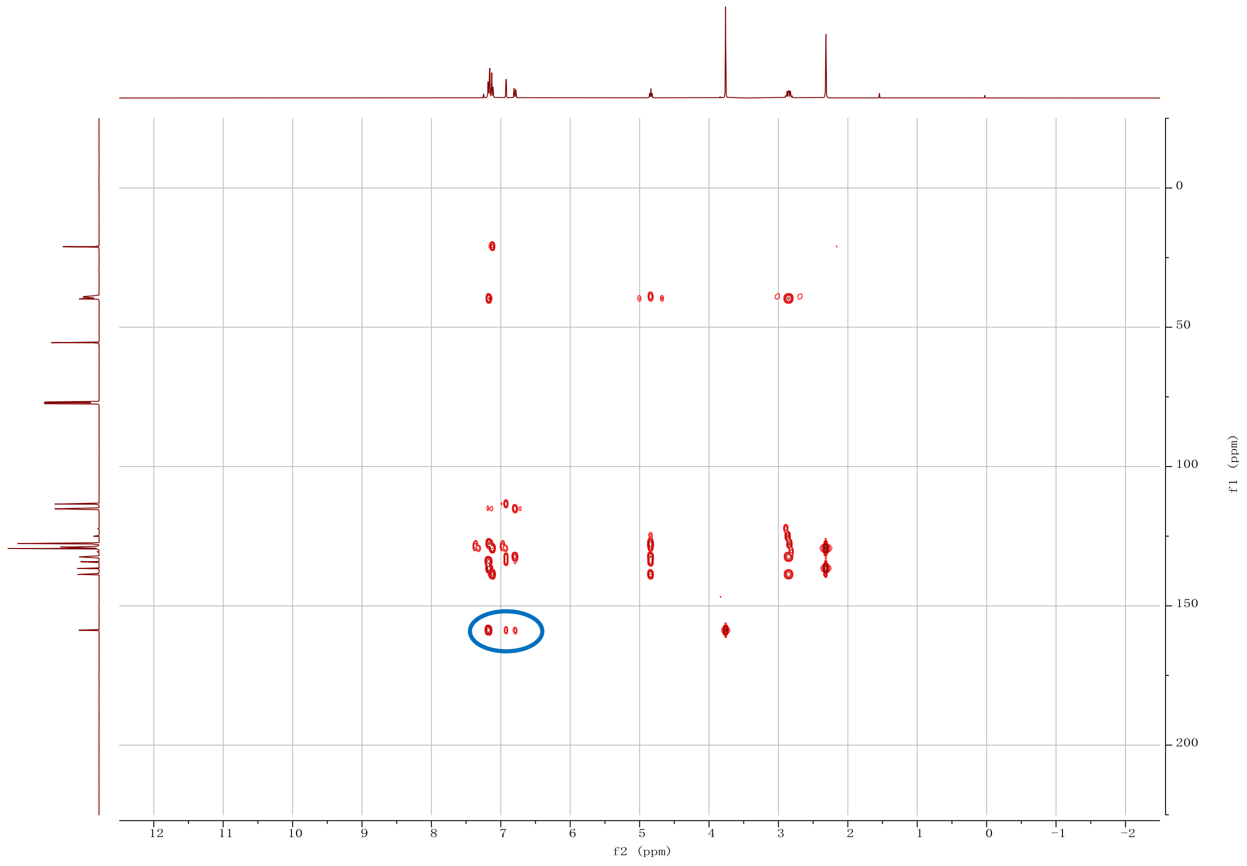

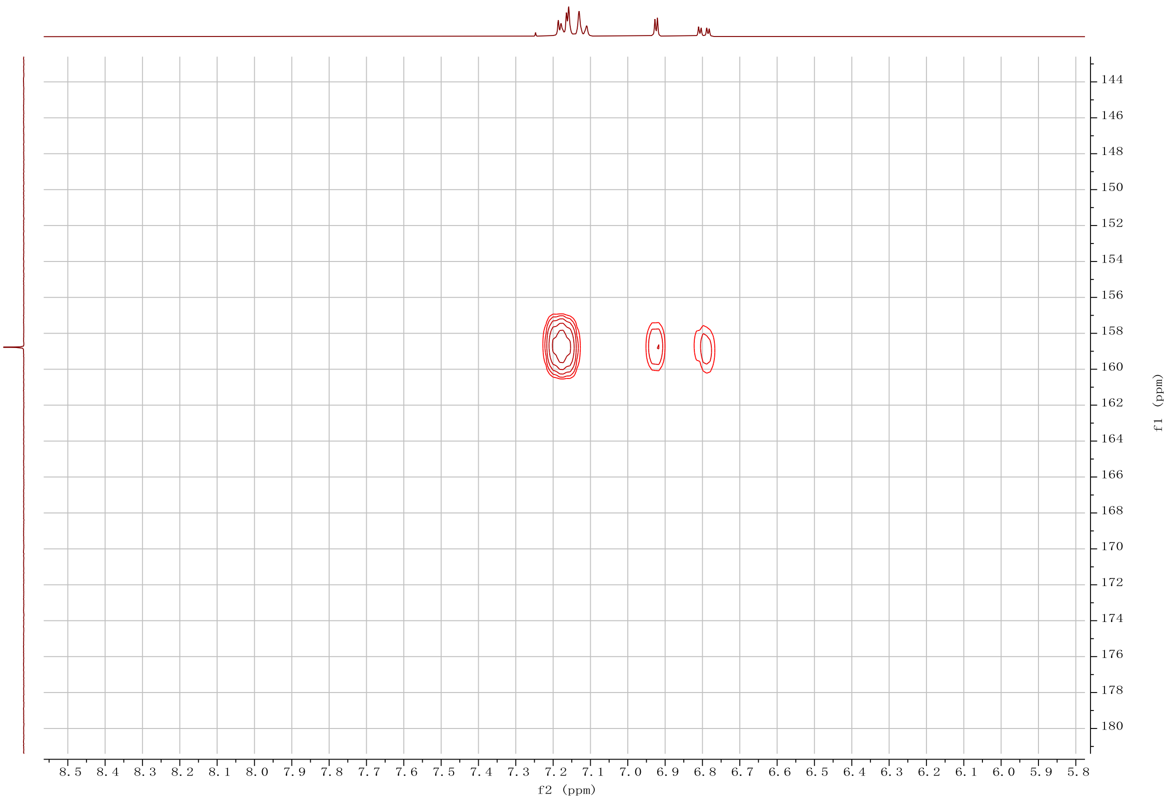

4-methoxy-2-methyl-1-(3,3,3-trifluoro-1-(p-tolyl)propyl)benzene (4i)

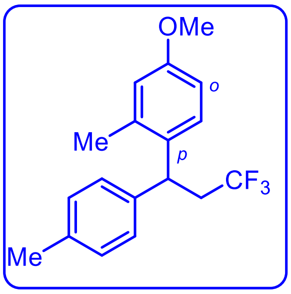

Following the general procedure (except the reaction was carried out with 10 mol% CuBr, 3eq. NFSI at 100 °C), the reaction mixture was analyzed by ^{19}F NMR giving a ratio of 2.2:1 (*para/ortho*). Isolated yield of the major product = 49 % (30.2 mg); colorless oil; R_f = 0.4 (PE: EtOAc =100:1). **^{1}H NMR** (400 MHz, $CDCl_3$)·δ 7.14 (d, *J* = 8.5 Hz, 1H), 7.07 (s, 4H), 6.73 (dd, *J* = 8.5, 2.9 Hz, 1H), 6.68 (d, *J* = 2.8 Hz, 1H), 4.45 (t, *J* = 7.3 Hz, 1H), 3.75 (s, 3H), 2.88 – 2.72 (m, 2H), 2.28 (s, 3H), 2.28 (s, 3H). **^{13}C NMR** (101 MHz, $CDCl_3$) δ 158.0, 139.6, 137.1, 136.1, 133.2, 129.2, 127.6, 127.3, 126.5 (q, *J* = 277.8 Hz), 116.3, 111.3, 55.1, 39.9 (q, *J* = 27.0 Hz), 39.4 (q, *J* = 2.8 Hz), 20.9, 20.0. **^{19}F NMR** (376 MHz, $CDCl_3$) δ -63.63 (t, *J* = 10.7 Hz). **HRMS** (ESI, *m/z*): $[M+Na]^+$ Calcd for $C_{18}H_{19}F_3NaO^+$ 331.1280, found 331.1282.

2-methoxy-4-methyl-1-(3,3,3-trifluoro-1-(p-tolyl)propyl)benzene (4i')

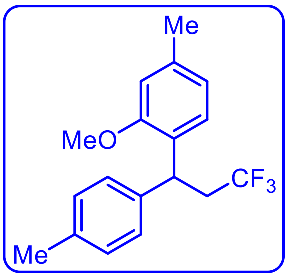

Isolated yield of the ortho product = 22% (13.6 mg); colorless oil; R_f = 0.3 (PE: EtOAc =100:1). **^{1}H NMR** (400 MHz, $CDCl_3$) δ 7.16 (d, *J* = 8.1 Hz, 2H), 7.07 (d, *J* = 8.1 Hz, 2H), 7.01 (d, *J* = 7.7 Hz, 1H), 6.70 (d, *J* = 7.6 Hz, 1H), 6.65 (s, 1H), 4.66 (t, *J* = 7.3 Hz, 1H), 3.78 (s, 3H), 2.92 – 2.76 (m, 2H), 2.29 (s, 3H), 2.28 (s, 3H). **^{13}C NMR** (101 MHz, $CDCl_3$) δ 156.4, 139.6, 137.7, 135.9, 129.0, 128.8, 128.5, 127.7, 126.7 (q, *J* = 277.9 Hz), 121.1, 111.9, 55.4, 38.5 (q, *J* = 27.1 Hz), 37.8 (q, *J* = 3.0 Hz), 21.4, 21.0. **^{19}F NMR** (376 MHz, $CDCl_3$) δ -63.88 (d, *J* = 10.3 Hz). **HRMS** (ESI, *m/z*): $[M+Na]^+$ Calcd for $C_{18}H_{19}F_3NaO^+$ 331.1280, found 331.1279.

4-chloro-1-methoxy-2-(3,3,3-trifluoro-1-(p-tolyl)propyl)benzene (4j)

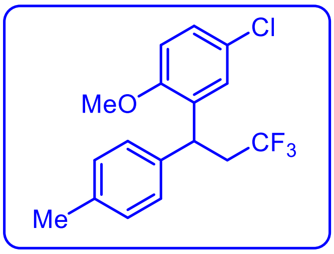

Following the general procedure (except the reaction was carried out with 10 mol% CuBr, 3 eq. NFSI at 100 °C). Isolated yield of the major product = 54% (35.7 mg); colorless oil; R_f = 0.4 (PE: EtOAc =100:1). **^{1}H NMR** (400 MHz, $CDCl_3$) δ 7.16 – 7.09 (m, 6H), 6.75 (d, *J* = 8.6 Hz, 1H), 4.67 (t, *J* = 7.3 Hz, 1H), 3.79 (s, 3H), 2.93– 2.74 (m, 2H), 2.31 (s, 3H). **^{13}C NMR** (101 MHz, $CDCl_3$) δ 155.2, 138.5, 136.4, 133.2, 129.2, 127.9, 127.6, 127.5, 126.5 (q, *J* = 277.6 Hz), 125.5, 112.2, 55.7, 38.3 (q, *J* = 27.5 Hz), 37.9 (q, *J* = 2.9 Hz), 21.0. **^{19}F NMR** (376 MHz, $CDCl_3$) δ -63.90 (t, *J* = 10.2 Hz). **HRMS** (ESI, *m/z*): $[M+Na]^+$ Calcd for $C_{17}H_{16}{}^{35}ClF_3NaO^+$ 351.0734, found 351.0736.

1-methoxy-4-methyl-2-(3,3,3-trifluoro-1-(p-tolyl)propyl)benzene (4k)

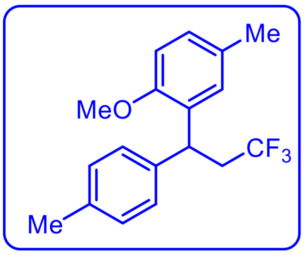

Following the general procedure (except the reaction was carried out with 10 mol% CuBr, 3 eq. NFSI at 120 °C). Isolated yield of the major product = 58% (35.5 mg); colorless oil; R_f = 0.4 (PE: EtOAc =100:1). **^{1}H NMR** (400 MHz, $CDCl_3$) δ 7.17 (d, *J* = 8.2 Hz, 2H), 7.07 (d, *J* = 7.9 Hz, 2H), 6.97 – 6.93 (m, 2H), 6.72 (d, *J* = 8.1 Hz, 1H), 4.66 (t, *J* = 7.3 Hz, 1H), 3.75 (s, 3H), 2.92 – 2.79 (m, 2H), 2.28 (s, 3H), 2.24 (s, 3H). **^{13}C NMR** (101 MHz, $CDCl_3$) δ 154.5, 139.5, 135.9, 131.2, 129.7, 129.0, 128.6, 128.1, 127.7, 126.7 (q, *J* = 277.7 Hz), 111.0, 55.5, 38.5 (q, *J* = 27.1 Hz), 38.1 (q, *J* = 3.1 Hz), 21.0, 20.6. **^{19}F NMR** (376 MHz, $CDCl_3$) δ -63.86 (t, *J* = 10.1 Hz). **HRMS** (ESI, *m/z*): $[M+Na]^+$ Calcd for $C_{18}H_{19}F_3NaO^+$ 331.1280, found 331.1280.

1-(3,3,3-trifluoro-1-(p-tolyl)propyl)naphthalene and 2-(3,3,3-trifluoro-1-(p-

tolyl)propyl)naphthalene (4l)

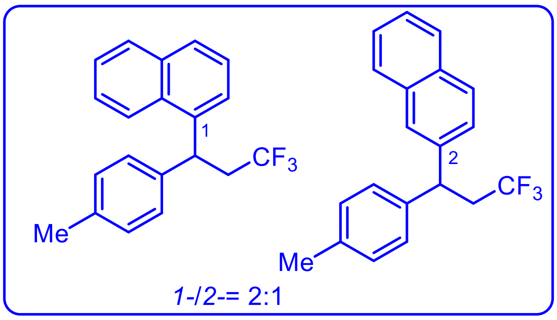

Following the general procedure (except the reaction was carried out at 100 °C), the reaction mixture was analyzed by ^{19}F NMR giving a ratio of 2:1 (*1-/2-*). Isolated yield of the two isomers = 74% (46.4 mg); colorless oil; R_f = 0.5 (PE: EtOAc =100:1). **^{1}H NMR** (400 MHz, $CDCl_3$) δ 8.14 – 8.11 (m, 1H), 7.81 (dd, *J* = 8.0, 1.6 Hz, 1H), 7.78 – 7.71 (m, 1H), 7.50 – 7.40 (m, 3H), 7.36 (dd, *J* = 7.3, 1.4 Hz, 1H), 7.19 (d, *J* = 8.2 Hz, 2H), 7.09 – 7.05 (m, 2H), 5.13 (t, *J* = 7.2 Hz, 1H), 3.04 – 2.89 (m, 2H), 2.25 (s, 3H). **^{13}C NMR** (101 MHz, $CDCl_3$) δ 139.1, 138.5, 136.4, 134.1, 131.0, 129.3, 129.0, 127.7, 127.4, 126.6 (q, *J* = 277.8 Hz), 126.4, 125.6, 125.2, 124.3, 123.1, 39.8 (q, *J* = 27.0 Hz), 39.7 (q, *J* = 3.1 Hz), 20.9. **^{19}F NMR** (376 MHz, $CDCl_3$) δ -63.31 (t, *J* = 10.7 Hz). **HRMS** (ESI, *m/z*): $[M+Na]^+$ Calcd for $C_{20}H_{17}F_3Na^+$ 337.1175, found 337.1174.

1-fluoro-4-(3,3,3-trifluoro-1-(p-tolyl)propyl)naphthalene (4m)

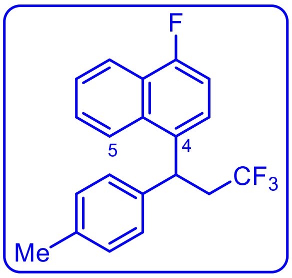

Following the general procedure (except the reaction was carried out with 10 mol% CuBr, DCE (1 M) at 100 °C), the reaction mixture was analyzed by ^{19}F NMR giving a ratio of 14:1 (*4-/5-*). Isolated yield of the two isomers = 57% (38.1 mg); colorless oil; R_f = 0.5 (PE: EtOAc =100:1). **^{1}H NMR** (400 MHz, $CDCl_3$) δ 8.13 (dd, *J* = 3.9, 1.9 Hz, 1H), 8.11 (dd, *J* = 4.0, 1.9 Hz, 1H), 7.56 – 7.48 (m, 2H), 7.29 (dd, *J* = 8.1, 5.3 Hz, 1H), 7.18 (d, *J* = 8.1 Hz, 2H), 7.11 (d, *J* = 8.1 Hz, 1H), 7.08 (d, *J* = 8.0 Hz, 2H), 5.07 (t, *J* = 7.2 Hz, 1H), 3.04 – 2.89 (m, 2H), 2.27 (s, 3H). **^{13}C NMR** (101 MHz, $CDCl_3$) δ 158.0 (d, *J* = 251.8 Hz), 139.0, 136.6, 134.4 (d, *J* = 4.5 Hz), 132.3 (d, *J* = 4.3 Hz), 129.4, 127.6, 127.3, 126.0 (d, *J* = 2.1 Hz), 124.3 (d, *J* = 13.2 Hz), 124.2 (d, *J* = 5.9 Hz), 123.2 (d, *J* = 2.8 Hz), 121.4 (d, *J* = 6.2 Hz), 108.6 (d, *J* = 20.1 Hz), 39.8 (q, *J* = 27.4 Hz), 39.5 (q, *J* = 2.9 Hz), 20.9. **^{19}F NMR** (376 MHz, $CDCl_3$) δ -63.37 (t, *J* = 10.1 Hz), -124.00 (dd, *J* = 10.1, 4.6 Hz). **HRMS** (ESI, *m/z*): $[M+Na]^+$ Calcd for $C_{20}H_{16}F_4Na^+$ 355.1080, found 355.1075. This structure is similar to that reported in the known literature.[13]

5-bromo-3-(3,3,3-trifluoro-1-(p-tolyl)propyl)benzo[b]thiophene (4n)

Following the general procedure, the reaction mixture was analyzed by ^{19}F NMR giving a ratio of 13:1

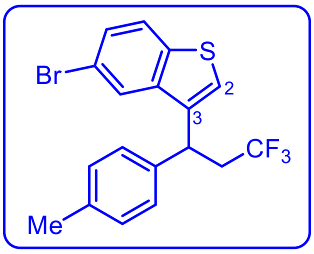

(*3-/2-*). Isolated yield of the two isomers = 65% (51.1 mg); colorless oil; R_f = 0.5 (PE: EtOAc =100:1). **^{1}H NMR** (400 MHz, $CDCl_3$) δ 7.78 (d, *J* = 1.9 Hz, 1H), 7.64 (d, *J* = 8.5 Hz, 1H), 7.38 (dd, *J* = 8.5, 1.9 Hz, 1H), 7.22 (s, 1H), 7.16 (d, *J* = 8.2 Hz, 2H), 7.10 (d, *J* = 8.1 Hz, 2H), 4.58 (t, *J* = 7.3 Hz, 1H), 3.02 – 2.80 (m, 2H), 2.28 (s, 3H). **^{13}C NMR** (101 MHz, $CDCl_3$) δ 139.5, 139.2, 137.6, 137.0, 136.7, 129.6, 127.6, 127.4, 126.2 (q, *J* = 277.8 Hz), 124.7, 124.2, 123.6, 118.4, 39.7 (q, *J* = 27.6 Hz), 38.7 (q, *J* = 2.9 Hz), 21.0. **^{19}F NMR** (376 MHz, $CDCl_3$) δ -63.38 (t, *J* = 10.2 Hz). **HRMS** (ESI, *m/z*): $[M+Na]^+$ Calcd for $C_{18}H_{14}{}^{79}BrF_3NaS^+$ 420.9844, found 420.9839.

3-bromo-2-(3,3,3-trifluoro-1-(p-tolyl)propyl)benzo[b]thiophene (4o)

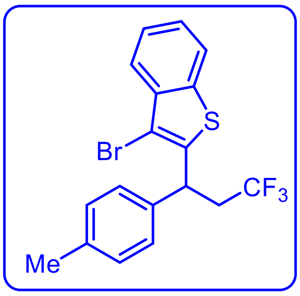

Following the general procedure (except the reaction was carried out in DCE (0.75 M) at 100 °C). Isolated yield of the two isomers = 52% (41.6 mg); colorless oil; R_f = 0.7 (PE: EtOAc =100:1). **^{1}H NMR** (400 MHz, $CDCl_3$) δ 7.74 (dt, *J* = 8.0, 1.0 Hz, 1H), 7.68 (dt, *J* = 8.0, 0.9 Hz, 1H), 7.38 (ddd, *J* = 8.1, 7.2, 1.1 Hz, 1H), 7.32 – 7.28 (m, 3H), 7.14 (d, *J* = 7.6 Hz, 2H), 5.00 (t, *J* = 7.3 Hz, 1H), 3.02 – 2.90 (m, 2H), 2.30 (s, 3H). **^{13}C NMR** (101 MHz, $CDCl_3$) δ 141.8, 138.0, 137.4, 137.2, 137.0, 129.6, 127.3, 125.9 (q, *J* = 277.8 Hz), 125.3, 125.2, 123.2, 122.4, 106.3, 40.1 (q, *J* = 3.1 Hz), 39.6 (q, *J* = 28.1 Hz), 21.0. **^{19}F NMR** (376 MHz, $CDCl_3$) δ -63.90 (d, *J* = 10.1 Hz). **HRMS** (ESI, *m/z*): $[M+Na]^+$ Calcd for $C_{18}H_{14}{}^{79}BrF_3NaS^+$ 420.9844, found 420.9846.

4. Synthetic Applications

4.1 Gram-scale synthesis

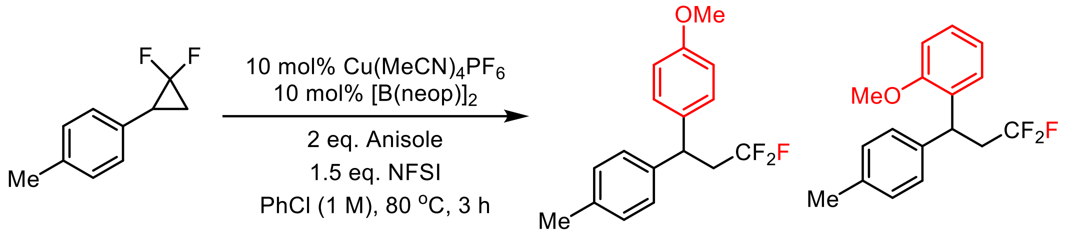

1a
6 mmol

2a
major

2a'
minor

A 100 mL round bottom flask with a stirring bar, was charged with **1a** (6 mmol), **anisole** (12 mmol, 2 eq.), **NFSI** (9 mmol, 1.5 eq.), $Cu(MeCN)_4PF_6$ (0.6 mmol, 0.1 eq.) and $[B(neop)]_2$ (0.6 mmol, 0.1 eq.) in dry PhCl (6 mL). Next, the round bottom flask was sealed, removed from the glove box and equipped with a nitrogen balloon. After stirring at 80 °C for 3 h, the reaction was completed. Then, the reaction mixture was analyzed by ^{19}F NMR and ^{1}H NMR to measure the regioselectivity (**2a**: **2a'**= 10:1), and the product was isolated through chromatography on a silica gel column to give the major product **2a** in 74% yield (1.31 g).

Note: The reaction is highly exothermic, and the reaction flask was required to be equipped with a nitrogen balloon to avoid explosion risk.

4.2 Synthesis of *gem*-difluoromethyl product

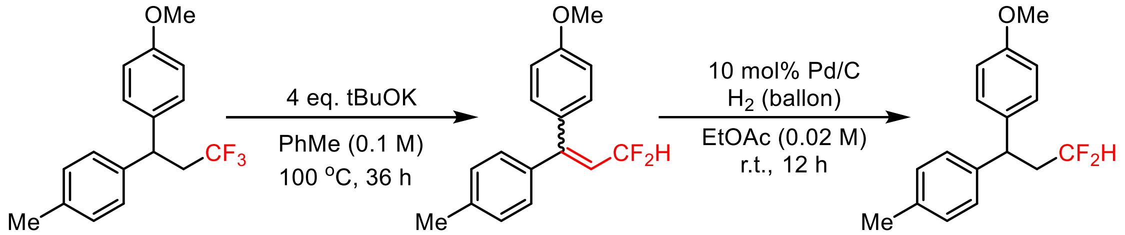

2a **5** **6**

A 4 mL vial equipped with stir bar was charged with (*Z*)-1-(2-fluoro-3-phenylallyl)-4-methoxybenzene (**2a**) (0.05 mmol, 1.0 eq.) and tBuOK (0.2 mmol, 4 eq.) in PhMe (0.5 mL). Next, the 4 mL vial was sealed and removed from the glove box. After stirring at 100 °C for 36 h, the reaction was completed. the reaction solution was concentrated and purified by silica gel column chromatography (200-300 mesh) to obtain **5**. Next step, a 25 mL round bottom flask equipped with a stirring bar, was charged with Pd/C (21.2 mg, 40 wt%, 10 mol%). After the flask was evacuated and filled with H_2 (three cycles), **5** (0.1 mmol, 1 eq.) and EtOAc (3 mL) was added and the reaction mixture

was stirred 25 °C for 12 hours under a H_2 atmosphere (H_2 balloon). The reaction mixture was filtered through a pad of celite and concentrated. Purification by column chromatography on silica gel afforded the desired product **6**.

4.3 Selective oxidative degradation of the aryl group

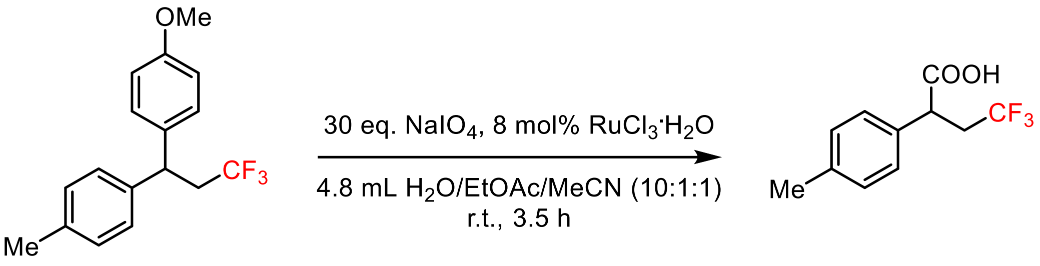

2a, 0.1 mmol **7a**, 60%

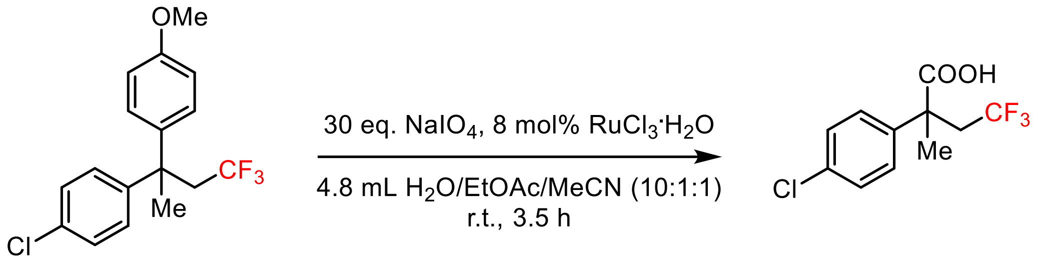

2w, 0.1 mmol **7w**, 92%

Following the literature reported procedure[14], under air atmosphere, a 20 mL vial equipped with stir bar was charge with **2a** or **2w** (0.1 mmol, 1 eq.), H_2O (4 mL), EtOAc (0.4mL), and MeCN (0.4 mL). To this solution was added $NaIO_4$ (6 mmol, 30 eq.) and $RuCl_3{\cdot}H_2O$ (0.08 mmol, 0.8 eq.) at 25 °C for 3.5 h. After completion of the reaction, DCM was added, and the aqueous and organic layers were separated. The aqueous layer was extracted with DCM (10 mL X 3), washed with brine, dried over $MgSO_4$ filtered and concentrated. The residue was purified by column chromatography on silica gel to afforded the desired product **7a** (60% yield) or **7w** (92% yield).

4.4 Characterization data of the products

1-(3,3-difluoro-1-(4-methoxyphenyl)prop-1-en-1-yl)-4-methylbenzene (Z/E mixture)(5a)

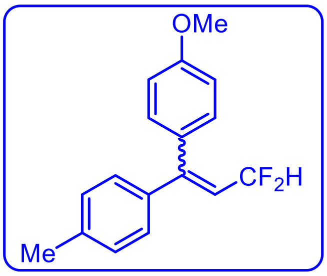

Following the general procedure. Isolated yield = 65% (8.9 mg); colorless oil; R_f = 0.5 (PE: EtOAc =100:1). **^{1}H NMR** (400 MHz, $CDCl_3$) δ 7.22 – 7.18 (m, 2H), 7.15 (d, J = 8.8 Hz, 3H), 7.11 (d, J = 8.1 Hz, 1H), 6.92 (d, J = 8.7 Hz, 1H), 6.84 (d, J = 8.9 Hz, 1H), 6.19 – 5.86 (m, 2H), 3.85 – 3.81 (s, 3H), 2.40 – 2.36 (s, 3H). **^{13}C NMR** (101 MHz, $CDCl_3$) δ 160.3, 159.8, 150.6 – 149.9 (m), 139.0, 138.5, 137.6, 134.5, 132.7, 131.2, 129.7, 129.4, 129.0, 128.1, 118.7 (t, J =

26.5 Hz), 114.1 (t, J = 228.8 Hz), 114.0 (t, J = 228.8 Hz), 113.7, 113.7, 55.3, 21.24, 21.17. **^{19}F NMR** (376 MHz, $CDCl_3$) δ -105.70 – -106.07 (m). **HRMS** (ESI, *m/z*): $[M+Na]^+$ Calcd for $C_{17}H_{16}F_2NaO^+$ 297.1061, found 297.1063.

4-(3,3-difluoro-1-(4-methoxyphenyl)prop-1-en-1-yl)-1,1'-biphenyl (Z/E mixture) (5b)

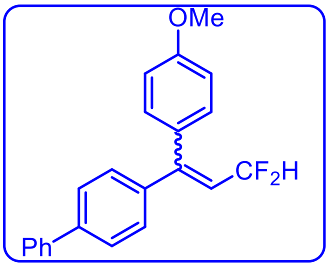

Following the general procedure. Isolated yield = 72% (12.1 mg); colorless oil; R_f = 0.4 (PE: EtOAc =100:1). **^{1}H NMR** (400 MHz, $CDCl_3$) δ 7.63 – 7.57 (m, 3H), 7.55 (d, J = 8.5 Hz, 1H), 7.48 – 7.41 (m, 2H), 7.39 – 7.33 (m, 2H), 7.29 (d, J = 8.3 Hz, 1H), 7.25 (d, J = 8.9 Hz, 1H), 7.19 (d, J = 8.8 Hz, 1H), 6.94 (d, J = 8.8 Hz, 1H), 6.86 (d, J = 8.9 Hz, 1H), 6.23 – 5.92 (m, 2H), 3.85 – 3.81 (s, 3H). **^{13}C NMR** (101 MHz, $CDCl_3$) δ 160.4, 160.0, 150.6 – 149.4 (m), 141.8, 141.4, 140.3, 139.3, 136.3, 132.4, 131.2, 130.3, 129.4, 128.9, 128.8, 128.6, 127.7, 127.6, 127.1, 127.0, 119.4 (t, J = 26.5 Hz), 118.4 (t, J = 26.6 Hz), 114.02 (t, J = 228.9 Hz), 113.93 (t, J = 229.2 Hz), 113.82, 113.77, 55.3. **^{19}F NMR** (376 MHz, $CDCl_3$) δ -105.79 – -106.00 (m). **HRMS** (ESI, *m/z*): $[M+Na]^+$ Calcd for $C_{22}H_{18}F_2NaO^+$ 359.1218, found 359.1220.

2-(3,3-difluoro-1-(4-methoxyphenyl)prop-1-en-1-yl)naphthalene (Z/E mixture) (5c)

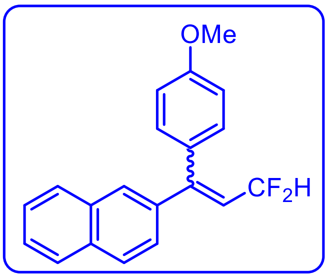

Following the general procedure. Isolated yield = 89% (13.9 mg); colorless oil; R_f = 0.4 (PE: EtOAc =100:1). **^{1}H NMR** (400 MHz, $CDCl_3$) δ 7.89 – 7.81 (m, 2H), 7.78 – 7.76 (m, 1H), 7.68 (s, 1H), 7.55 – 7.53 (m, 1H), 7.50 – 7.43 (m, 2H), 7.27 – 7.20 (m, 2H), 6.96 (d, J = 8.8 Hz, 1H), 6.85 (d, J = 9.0 Hz, 1H), 6.26 – 5.89 (m, 2H), 3.86 – 3.81 (s, 3H). **^{13}C NMR** (101 MHz, $CDCl_3$) δ 160.4, 160.0, 150.2 (dt, J = 32.8, 12.8 Hz), 137.8, 134.8, 133.4, 133.1, 133.0, 132.9, 132.3, 131.3, 129.4, 129.1, 128.4, 128.1 (t, J = 10.4 Hz), 127.7, 127.6, 127.3, 126.7 (t, J = 5.2 Hz), 126.4, 125.4, 120.0 (t, J = 26.5 Hz), 118.6 (t, J = 26.6 Hz), 114.1 (t, J = 229.1 Hz), 113.9 (t, J = 229.2 Hz), 113.8, 113.8, 55.3. **^{19}F NMR** (376 MHz, $CDCl_3$) δ -105.74 – -105.96 (m). **HRMS** (ESI, *m/z*): $[M+Na]^+$ Calcd for $C_{20}H_{16}F_2NaO^+$ 333.1061, found 333.1063.

1-(3,3-difluoro-1-(4-phenoxyphenyl)prop-1-en-1-yl)-4-methylbenzene(Z/E mixture) (5d)

Following the general procedure. Isolated yield = 66% (11.2 mg); colorless oil; R_f = 0.5 (PE: EtOAc

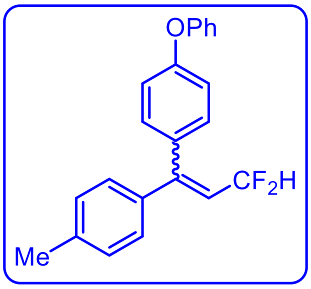

=100:1). **¹H NMR** (400 MHz, $CDCl_3$) δ 7.40 – 7.33 (m, 2H), 7.24 – 7.07 (m, 8H), 7.04 – 7.00 (m, 2H), 6.93 (d, *J* = 8.8 Hz, 1H), 6.21 – 5.87 (m, 2H), 2.39 – 2.36 (s, 3H). **¹³C NMR** (101 MHz, $CDCl_3$) δ 158.3, 158.0, 156.5, 156.3, 149.9 (d, *J* = 12.8 Hz), 139.2, 138.7, 137.4, 134.9, 134.2, 131.9, 131.4, 129.9, 129.8, 129.7, 129.5, 129.1, 128.0, 123.9, 123.8, 119.6, 119.4, 118.9 (t, *J* = 26.8 Hz), 118.1, 118.0, 114.0 (t, *J* = 229.0 Hz), 113.9 (t, *J* = 229.0 Hz), 21.24, 21.18. **¹⁹F NMR** (376 MHz, $CDCl_3$) δ -105.97 (dd, *J* = 55.8, 7.8 Hz), -106.18 (dd, *J* = 56.0, 7.6 Hz). The HRMS was not satisfied, and the result of LRMS was obtained for this compound. **LRMS** (EI) m/z: 338 (M+, 65), 273 (100), 180 (18), 165 (26), 115(2), 107(4), 77 (3).

1-(3,3-difluoro-1-(4-methoxyphenyl)propyl)-4-methylbenzene (6a)

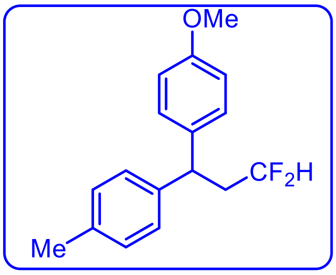

Following the general procedure. Isolated yield = 98% (27.1 mg); colorless oil; R_f = 0.5 (PE: EtOAc =100:1). **¹H NMR** (400 MHz, $CDCl_3$) δ 7.14 (d, *J* = 8.7 Hz, 2H), 7.10 (s, 4H), 6.83 (d, *J* = 8.6 Hz, 2H), 5.56 (tt, *J* = 56.8, 5.1 Hz, 1H), 4.09 (t, *J* = 8.2 Hz, 1H), 3.76 (s, 3H), 2.58 – 2.45 (m, 2H), 2.30 (s, 3H). **¹³C NMR** (101 MHz, $CDCl_3$) δ 158.2, 140.2, 136.3, 135.2, 129.4, 128.5, 127.3, 116.7 (t, *J* = 238.7 Hz), 114.1, 55.2, 44.0 (t, *J* = 6.0 Hz), 39.9 (t, *J* = 21.2 Hz), 20.9. **¹⁹F NMR** (376 MHz, $CDCl_3$) δ -116.89 (dt, *J* = 56.4, 15.9 Hz). **HRMS** (ESI, *m/z*): $[M+Na]^+$ Calcd for $C_{17}H_{18}F_2NaO^+$ 299.1218, found 299.1223.

4-(3,3-difluoro-1-(4-methoxyphenyl)propyl)-1,1'-biphenyl (6b)

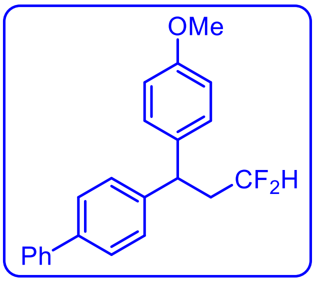

Following the general procedure. Isolated yield = 99% (33.5 mg); colorless oil; R_f = 0.4 (PE: EtOAc =100:1). **¹H NMR** (400 MHz, $CDCl_3$) δ 7.56 – 7.51 (m, 4H), 7.43 – 7.39 (m, 2H), 7.34 – 7.28 (m, 3H), 7.18 (d, *J* = 8.7 Hz, 2H), 6.85 (d, *J* = 8.7 Hz, 2H), 5.61 (tt, *J* = 56.7, 5.0 Hz, 1H), 4.17 (t, *J* = 8.2 Hz, 1H), 3.77 (s, 3H), 2.63 – 2.52 (m, 2H). **¹³C NMR** (101 MHz, $CDCl_3$) δ 158.4, 142.3, 140.6, 139.6, 134.8, 128.7, 128.6, 127.9, 127.5, 127.2, 127.0, 116.6 (t, *J* = 238.8 Hz), 114.2, 55.2, 44.1 (t, *J* = 6.0 Hz), 39.9 (t, *J* = 21.2 Hz). **¹⁹F NMR** (376 MHz, $CDCl_3$) δ -116.86 (dt, *J* = 56.7, 16.0 Hz). **HRMS** (ESI, *m/z*): $[M+Na]^+$ Calcd for $C_{22}H_{20}F_2NaO^+$ 361.1374, found 361.1373.

2-(3,3-difluoro-1-(4-methoxyphenyl)propyl)naphthalene (6c)

Following the general procedure. Isolated yield = 98% (36.7 mg); colorless oil; R_f = 0.4 (PE: EtOAc

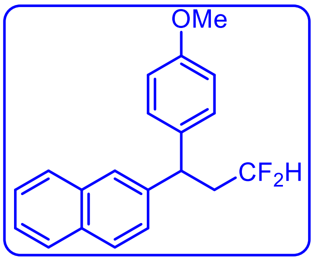

=100:1). **^{1}H NMR** (400 MHz, $CDCl_3$) δ 7.72 – 7.65 (m, 3H), 7.60 (s, 1H), 7.40 – 7.32 (m, 2H), 7.20 (dd, J = 8.5, 1.9 Hz, 1H), 7.09 (d, J = 8.7 Hz, 2H), 6.75 (d, J = 8.8 Hz, 2H), 5.52 (tt, J = 56.7, 5.1 Hz, 1H), 4.20 (t, J = 8.1 Hz, 1H), 3.66 (s, 3H), 2.62 – 2.49 (m, 2H). **^{13}C NMR** (101 MHz, $CDCl_3$) δ 158.4, 140.6, 134.7, 133.4, 132.3, 128.7, 128.5, 127.7, 127.6, 126.2, 126.1, 125.8, 125.5, 116.7 (t, J = 238.8 Hz), 114.1, 55.2, 44.5 (t, J = 6.0 Hz), 39.7 (t, J = 21.3 Hz). **^{19}F NMR** (376 MHz, $CDCl_3$) δ -116.75 (dt, J = 56.8, 15.7 Hz). **HRMS** (ESI, *m/z*): $[M+Na]^+$ Calcd for $C_{20}H_{18}F_2NaO^+$ 335.1218, found 335.1220.

1-(3,3-difluoro-1-(4-phenoxyphenyl)propyl)-4-methylbenzene (6d)

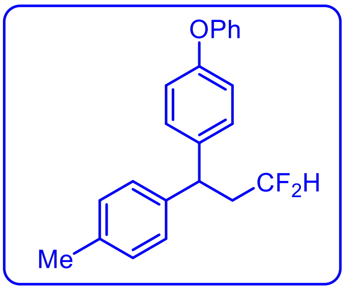

Following the general procedure. Isolated yield = 98% (33.0 mg); colorless oil; R_f = 0.5 (PE: EtOAc =100:1). **^{1}H NMR** (400 MHz, $CDCl_3$) δ 7.34 – 7.30 (m, 2H), 7.18 (d, J = 8.6 Hz, 2H), 7.13 (s, 4H), 7.11 – 7.07 (m, 1H), 6.99 – 6.92 (m, 2H), 6.93 (d, J = 8.7 Hz, 2H), 5.59 (tt, J = 56.7, 5.0 Hz, 1H), 4.13 (t, J = 8.2 Hz, 1H), 2.60 – 2.49 (m, 2H), 2.31 (s, 3H). **^{13}C NMR** (101 MHz, $CDCl_3$) δ 157.1, 155.9, 139.9, 138.0, 136.5, 129.7, 129.5, 128.7, 127.4, 123.3, 119.0, 118.9, 116.6 (d, J = 238.8 Hz), 44.2 (t, J = 6.0 Hz), 39.9 (t, J = 21.3 Hz), 21.0. **^{19}F NMR** (376 MHz, $CDCl_3$) δ -116.88 (dtd, J = 56.8, 15.8, 15.4, 7.5 Hz). **HRMS** (ESI, *m/z*): $[M+Na]^+$ Calcd for $C_{22}H_{20}F_2NaO^+$ 361.1374, found 361.1376.

4,4,4-trifluoro-2-(p-tolyl)butanoic acid (7a)

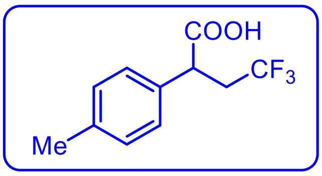

Following the general procedure. Isolated yield = 60% (14.0 mg); colorless oil; R_f = 0.5 (PE: EtOAc =10:1). **^{1}H NMR** (400 MHz, $CDCl_3$) δ 9.30 (s, 1H), 7.20 – 7.14 (m, 4H), 3.86 (dd, J = 8.6, 5.3 Hz, 1H), 3.05 (dqd, J = 14.9, 10.4, 8.6 Hz, 1H), 2.48 (ddq, J = 21.0, 10.3, 5.3 Hz, 1H), 2.33 (s, 3H). **^{13}C NMR** (101 MHz, $CDCl_3$) δ 178.0, 138.2, 133.3, 129.8, 127.5, 125.9 (q, J = 277.0 Hz), 44.8 (q, J = 2.9 Hz), 36.8 (q, J = 28.8 Hz), 21.0. **^{19}F NMR** (376 MHz, $CDCl_3$) δ -65.16 (t, J = 10.2 Hz). The HRMS was not satisfied, and the result of LRMS was obtained for this compound. **LRMS** (EI) m/z: 232 (M+, 56), 187 (100), 147 (9), 123 (100), 105 (12), 91 (10), 77 (8).

2-(4-chlorophenyl)-4,4,4-trifluoro-2-methylbutanoic acid (7w)

Following the general procedure. Isolated yield = 92% (24.6 mg); colorless oil; R_f = 0.5 (PE: EtOAc

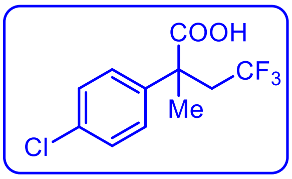

=10:1). **^{1}H NMR** (400 MHz, $CDCl_3$) δ 11.02 (s, 1H), 7.35 – 7.30 (m, 4H), 3.07 (dq, J = 15.3, 11.0 Hz, 1H), 2.67 (dq, J = 15.3, 10.6 Hz, 1H), 1.74 (s, 3H). **^{13}C NMR** (101 MHz, $CDCl_3$) δ 180.5, 139.0, 133.9, 129.0, 127.2, 125.7 (q, J = 278.5 Hz), 46.5 (d, J = 2.3 Hz), 42.1 (q, J = 27.8 Hz), 21.0. **^{19}F NMR** (376 MHz, $CDCl_3$) δ -59.59 (t, J = 10.8 Hz). HRMS was not satisfied, and the result of LRMS was obtained for this compound. **LRMS** (EI) m/z: 266 (M+, 38), 268 (13), 221 (100), 187 (7), 157 (57), 102 (7), 77 (7).

5. Experimental Mechanistic Investigations

The structures of the compound **8**[15], **9**[16], **11a**[17], **12a**[17] and ***S*-1e**[18] used in this study were prepared following known procedures.

5.1 The possible generation of benzyl radical

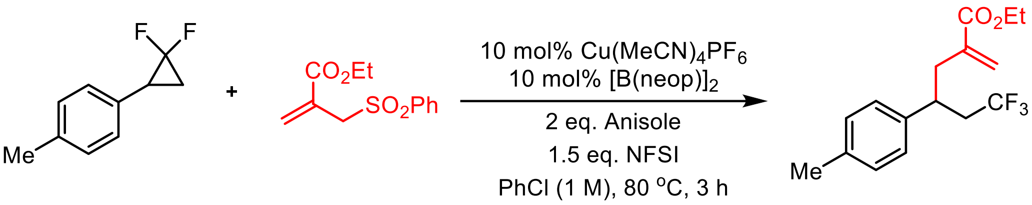

1a **8** no product

Following the general procedure: A 1.5 mL vial equipped with stir bar was charged with **1a** (0.2 mmol), **8** (0.4 mmol, 2 eq.), **NFSI** (0.3 mmol, 1.5 eq.), $Cu(MeCN)_4PF_6$ (0.02 mmol, 0.1 eq.) and $[B(neop)]_2$ (0.02 mmol, 0.1 eq.) in dry PhCl (0.2 mL). Next, the 1.5 mL vial was sealed and removed from the glove box. After stirring at 80 °C for 3 h, the reaction was completed. Then, the reaction mixture was analyzed by GCMS, which showed that there was no allylation product was obtained. This observation suggest that the reaction may not proceed through radical intermediate.

5.2 The reactivity different between simple cyclopropane and *gem*-DFCP

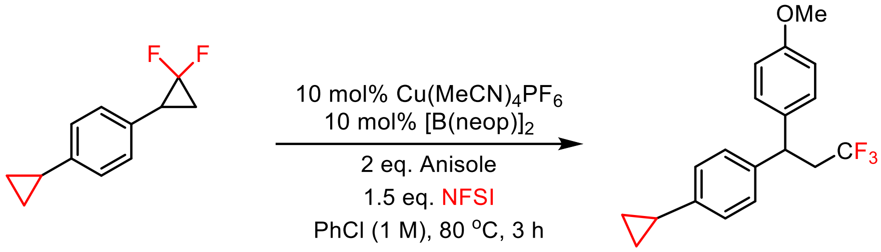

1q **2q**, 65% yield

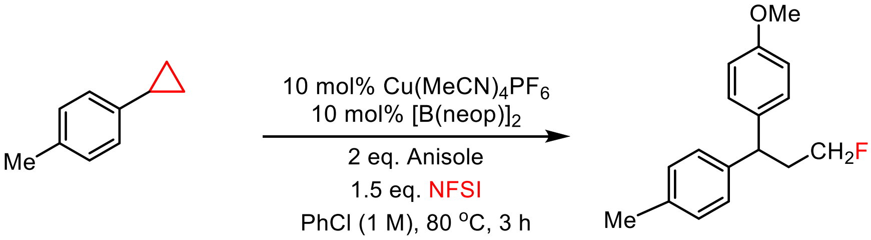

9 **10**, trace
recovery of **9** > 80%

Following the general procedure: A 1.5 mL vial equipped with stir bar was charged with **1q** or **9** (0.2 mmol), **anisole** (0.4 mmol, 2 eq.), **NFSI** (0.3 mmol, 1.5 eq.), $Cu(MeCN)_4PF_6$ (0.02 mmol, 0.1 eq.) and $[B(neop)]_2$ (0.02 mmol, 0.1 eq.) in dry PhCl (0.2 mL). Next, the 1.5 mL vial was sealed and removed from the glove box. After stirring at 80 °C for 3 h, the reaction was completed. The results

showed that our catalytic system has privileged reactivity on *gem*-DFCP over simple cyclopropane (**1q** to **2q**). It is indeed that simple arylcyclopropane **9** hardly shows reactivity under the standard reaction conditions, and only trace amount of fluoroarylation product **10** was obtained with **9** being recovered over 80%. Obviously, these results showed that the catalytic mechanism of this reaction system is different from that of conventional Cu catalyzed reaction of arylcyclopropane system.

5.3 The competing reaction pathways without arene nucleophiles

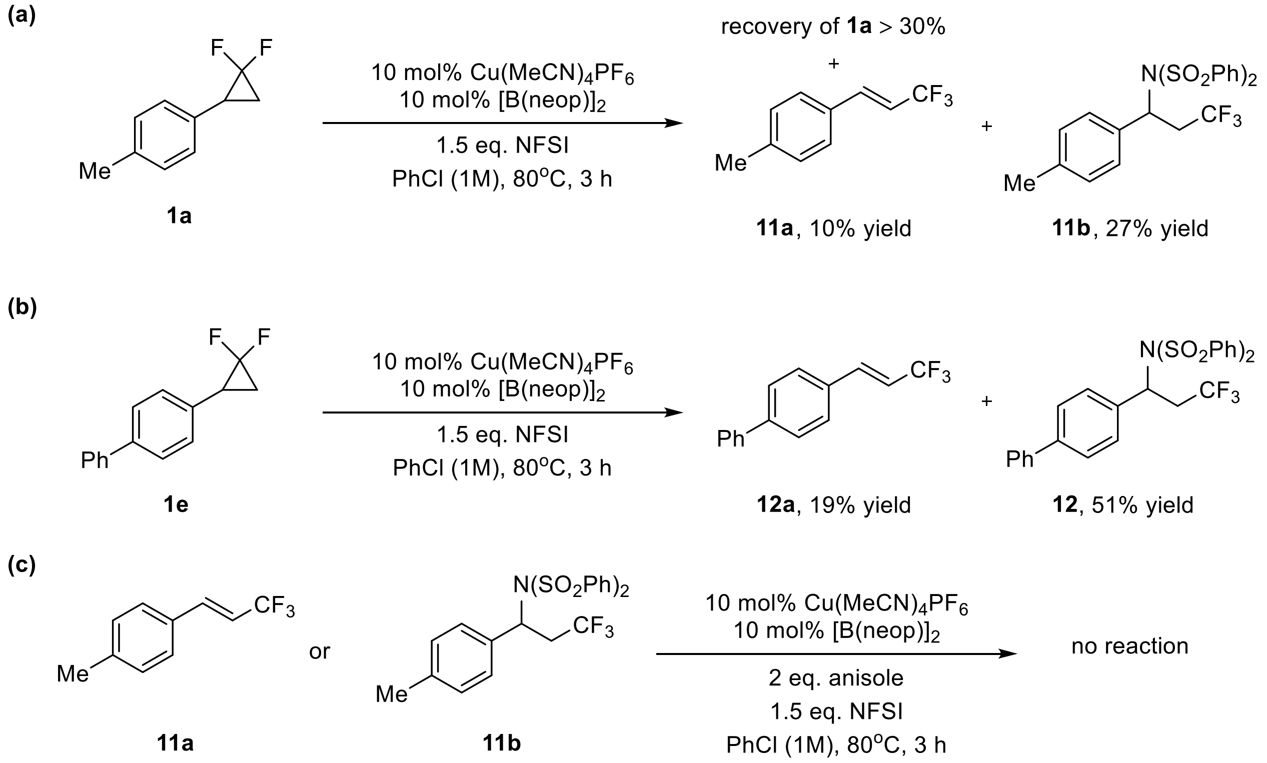

Following the general procedure: A 1.5 mL vial equipped with stir bar was charged with **1a** or **1e** (0.2 mmol), **NFSI** (0.3 mmol, 1.5 eq.), $Cu(MeCN)_4PF_6$ (0.02 mmol, 0.1 eq.) and $[B(neop)]_2$ (0.02 mmol, 0.1 eq.) in dry PhCl (0.2 mL). Next, the 1.5 mL vial was sealed and removed from the glove box. After stirring at 80 °C for 3 h, the reaction was completed. The yields of compounds (**11a-12**) were confirmed by F-NMR. Purification by column chromatography on silica gel afforded the product **11a**, **11b**, **12a** and **12**, in which **11a** and **12a** was literature reported compounds, the spectral data match the literature report.[17] **11b** and **12** were new compounds, which were characterized as follows. Subsequently, the introduction of substrates **11a** and **11b** into the standard reaction did not result in any observable reaction, and both **11a** and **11b** remained unchanged throughout the process. This outcome suggests the possibility of the formation of carbocation intermediate. In addition, the appearance of **11b** and **12** indicates the possible involvement of reductive elimination process of Cu(III)

species in the reaction. An alternative mechanism is that nitrogen nucleophilic attacks the benzyl carbocation produced by the dissociation of Cu(III) species.

N-(phenylsulfonyl)-N-(3,3,3-trifluoro-1-(p-tolyl)propyl)benzenesulfonamide (11b)

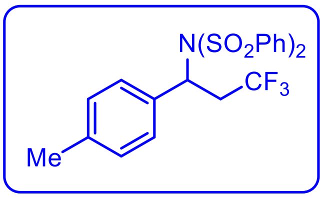

white solid; m.p.: 155.3-157.1 °C; R_f = 0.5 (PE: EtOAc =20:1). **^{1}H NMR** (400 MHz, $CDCl_3$) δ 8.16 (s, 2H), 7.61 – 7.08 (m, 12H), 5.81 (dd, *J* = 11.0, 2.5 Hz, 1H), 3.77 – 3.63 (m, 1H), 2.40 – 2.20 (m, 4H). **^{13}C NMR** (101 MHz, $CDCl_3$) δ 139.8, 138.7, 133.8, 130.8, 129.2, 128.9, 128.1, 125.0 (q, *J* = 277.5 Hz), 57.7 (q, *J* = 3.4 Hz), 37.2 (q, *J* = 28.5 Hz), 21.0. **^{19}F NMR** (376 MHz, $CDCl_3$) δ -63.97 (t, *J* = 10.2 Hz). **HRMS** (ESI, *m/z*): $[M+H]^+$ Calcd for $C_{22}H_{21}F_3NO_4S_2^+$ 484.0859, found 484.0862.

N-(1-([1,1'-biphenyl]-4-yl)-3,3,3-trifluoropropyl)-N-(phenylsulfonyl)benzenesulfonamide (12)

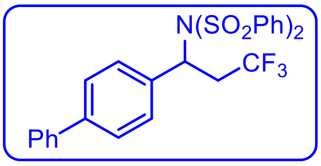

white solid; m.p.: 147.9-149.2 °C; R_f = 0.4 (PE: EtOAc =20:1). **^{1}H NMR** (400 MHz, $CDCl_3$) δ 8.20 – 7.63 (m, 4H), 7.62 – 7.57 (m, 5H), 7.52 – 7.48 (m, 3H), 7.48 – 7.45 (m, 2H), 7.40 – 7.36 (m, 2H), 7.32 – 7.11 (m, 3H), 5.91 (dd, *J* = 10.9, 2.5 Hz, 1H), 3.82 – 3.68 (m, 1H), 2.37 (dqd, *J* = 15.8, 10.8, 2.5 Hz, 1H). **^{13}C NMR** (101 MHz, $CDCl_3$) δ 141.7, 140.2, 134.3, 133.4, 132.8, 129.7, 128.9, 128.2, 127.7, 127.0, 126.9, 125.0 (q, *J* = 277.5 Hz), 57.5 (q, *J* = 3.5 Hz), 37.2 (q, *J* = 28.5 Hz). **^{19}F NMR** (376 MHz, $CDCl_3$) δ -63.97 (t, *J* = 10.2 Hz). **HRMS** (ESI, *m/z*): $[M+NH_4]^+$ Calcd for $C_{27}H_{26}F_3N_2O_4S_2^+$ 563.1280, found 563.1279.

X-ray Crystallographic Data of 12

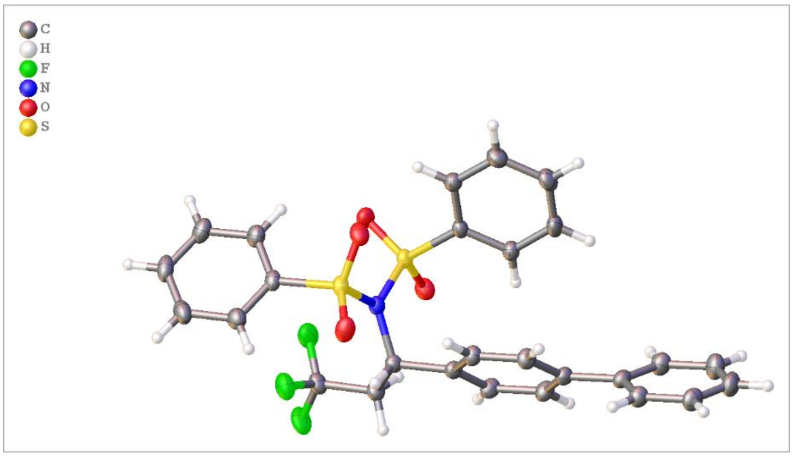

Identification code	**12**
Empirical formula	$C_{27}H_{22}F_3NO_4S_2$
Formula weight	545.57

Temperature/K	150.0
Crystal system	triclinic
Space group	P-1
a/Å	10.220(2)
b/Å	11.062(3)
c/Å	11.219(3)
α/°	107.399(9)
β/°	96.657(8)
γ/°	95.172(8)
Volume/Å^3	1191.7(5)
Z	2
ρ_{calc}g/cm^3	1.520
μ/mm^{-1}	0.284
F(000)	564.0
Crystal size/mm^3	0.36 × 0.27 × 0.22
Radiation	MoKα (λ = 0.71073)
2Θ range for data collection/	3.848 to 55.04
Index ranges	-13 ≤ h ≤ 13, -14 ≤ k ≤ 14, -14 ≤ l ≤ 14
Reflections collected	52711
Independent reflections	5480 [R_{int} = 0.0553, R_{sigma} = 0.0301]
Data/restraints/parameters	5480/0/334
Goodness-of-fit on F^2	1.025
Final R indexes [I>=2σ (I)]	R_1 = 0.0327, wR_2 = 0.0840

Final R indexes [all data] R_1 = 0.0417, wR_2 = 0.0883

Largest diff. peak/hole / e Å^{-3} 0.33/-0.37

5.4 The stereochemistry of the fluoroarylation reaction

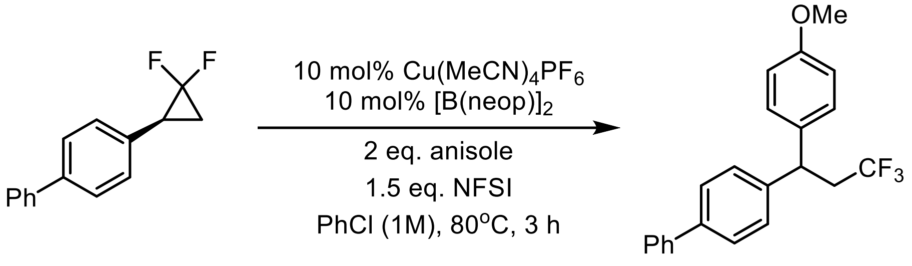

(*S*)-1e, > 99% ee   racemic **2e**

Following the general procedure: A 1.5 mL vial equipped with stir bar was charged with **(*S*)-1e** (0.2 mmol), **anisole** (0.4 mmol, 2 eq.), **NFSI** (0.3 mmol, 1.5 eq.), $Cu(MeCN)_4PF_6$ (0.02 mmol, 0.1 eq.) and $[B(neop)]_2$ (0.02 mmol, 0.1 eq.) in dry PhCl (0.2 mL). Next, the 1.5 mL vial was sealed and removed from the glove box. After stirring at 80 °C for 3 h, the reaction was completed. Then, the reaction mixture was analyzed HPLC find that the product **2e** was racemic. This observation further supports the involvement of carbocation intermediate in the arylation step.

Note: The ee value of **(*S*)-1e** used in this study was > 99%. Chiral HPLC (Chiralpak AD-H, Hexane:i-PrOH = 100:0, 1 mL/min, 254 nm, t_{major} = 20.0 min, t_{minor}= 13.0 min).

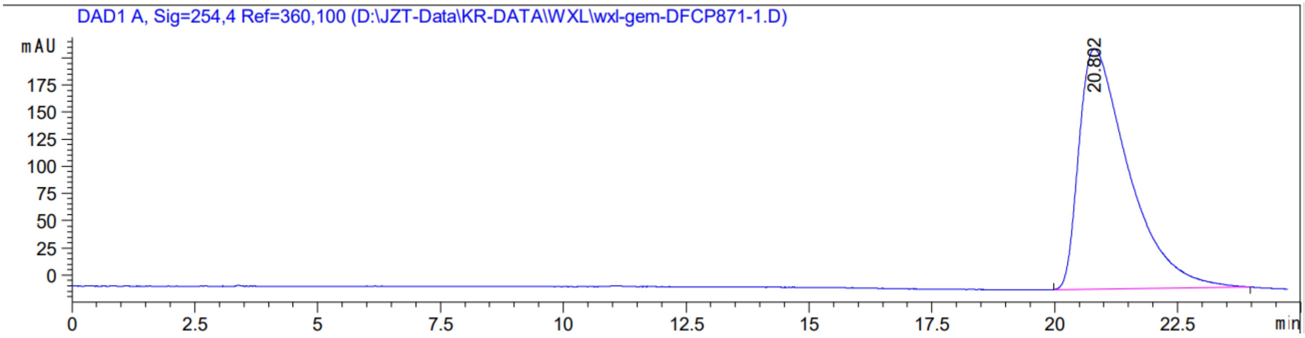

Signal 1: DAD1 A, Sig=254,4 Ref=360,100

Peak #	RetTime [min]	Type	Width [min]	Area [mAU*s]	Height [mAU]	Area %
1	20.802	BB	0.9700	1.55660e4	221.47029	100.0000

<u>The racemic 1e:</u>

Chiral HPLC (Chiralpak AD-H, Hexane:i-PrOH = 100:0, 1 mL/min, 254 nm, t_{major} = 20.0 min, t_{minor}= 13.0 min).

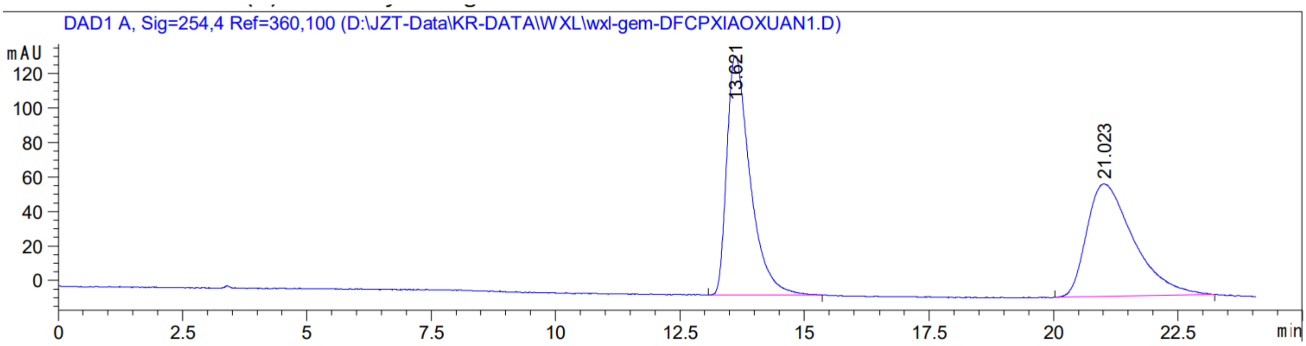

Signal 1: DAD1 A, Sig=254,4 Ref=360,100

Peak #	RetTime [min]	Type	Width [min]	Area [mAU*s]	Height [mAU]	Area %
1	13.621	BB	0.4795	4519.67920	138.94356	51.3873
2	21.023	BB	0.7772	4275.64209	65.35727	48.6127

<u>**The racemic 2e**</u>： Chiral HPLC (Chiralpak OJ-H, Hexane:i-PrOH = 95:5, 1 mL/min, 254 nm, t_{major} = 37.0 min, t_{minor}= 29.5 min).

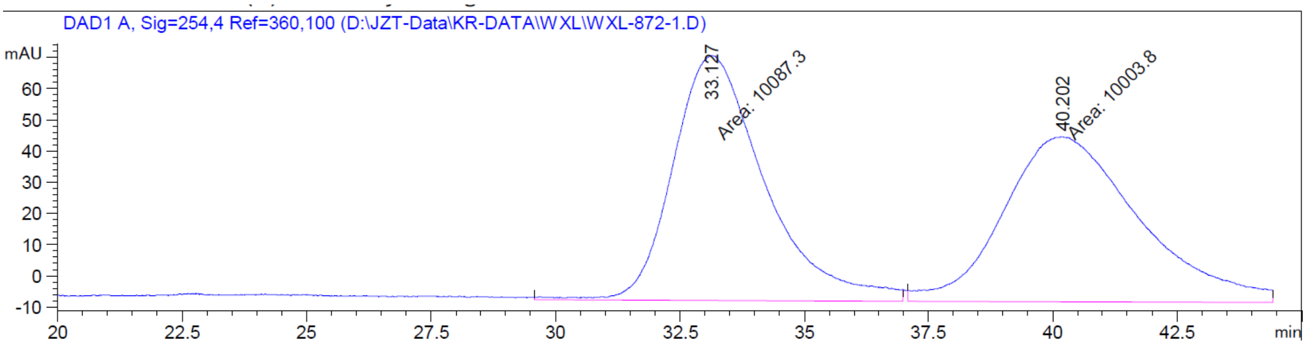

Signal 1: DAD1 A, Sig=254,4 Ref=360,100

Peak #	RetTime [min]	Type	Width [min]	Area [mAU*s]	Height [mAU]	Area %
1	33.127	MM	2.1422	1.00873e4	78.48005	50.2077
2	40.202	MM	3.1537	1.00038e4	52.86886	49.7923

5.5 The stereochemistry of the fluoroamination reaction

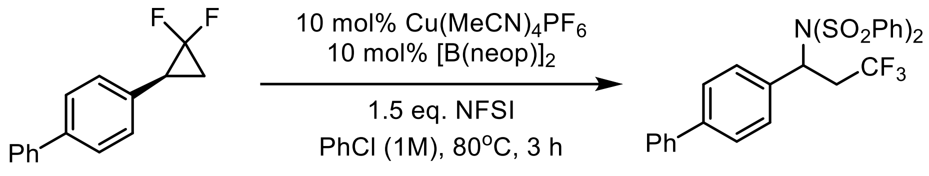

(*S*)-1e, > 99% ee racemic **12**

Following the general procedure: A 1.5 mL vial equipped with stir bar was charged with **(*S*)-1e** (0.2 mmol), **NFSI** (0.3 mmol, 1.5 eq.), $Cu(MeCN)_4PF_6$ (0.02 mmol, 0.1 eq.) and $[B(neop)]_2$ (0.02 mmol, 0.1 eq.) in dry PhCl (0.2 mL). Next, the 1.5 mL vial was sealed and removed from the glove box. After stirring at 80 °C for 3 h, the reaction was completed. Then, the reaction mixture was analyzed by HPLC and it was found that the product **12** was racemic. This observation indicates that the Cu(III) species can reversibly dissociate into Cu(I) and carbocation intermediates, which can be attacked by the dissociated nucleophilic amino species to afford racemic **12**.

<u>The racemic 12</u>： Chiral HPLC (Chiralpak OD-H, Hexane : i-PrOH = 95:5, 1 mL/min, 254 nm, t_{major} = 9.8 min, t_{minor} = 8.9 min).

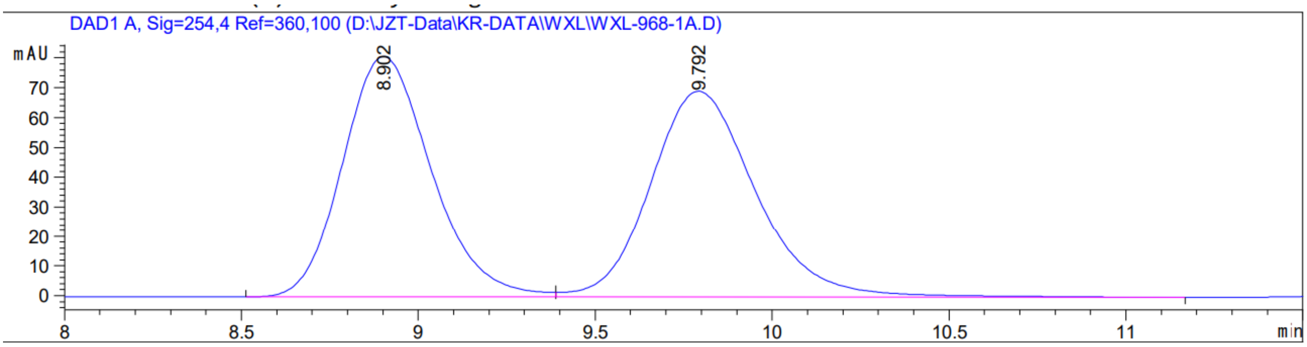

Signal 1: DAD1 A, Sig=254,4 Ref=360,100

Peak #	RetTime [min]	Type	Width [min]	Area [mAU*s]	Height [mAU]	Area %
1	8.902	BV	0.2698	1413.99841	80.92760	49.4557
2	9.792	VB	0.3192	1445.12231	69.32471	50.5443

5.6 The role of the $[B(neop)]_2$ additive in this reaction

$[B(neop)]_2$ additive was indeed found to be important for our reaction. While the exact role was not clear, there might be some possibilities. On one hand, the potential comproportionation between the activated Cu(III) and unactivated Cu(I) could lead to the formation of Cu(II) species. If the Cu(II) is an off-cycle species, the catalytic efficiency of Cu could be decreased. In this context, boron

additives could potentially reduce the Cu(II) to give Cu(I) that facilitates the reincorporation of more copper into the catalytic cycle. On the other hand, boron additives might serve as special Lewis acids to stabilize high-valent copper species through some interaction between the boron center and the F, N or O atoms on the ligands of the copper. Nevertheless, further investigations are required to better understand the details of the effects of $[B(neop)]_2$ additive.

6. Proposed Mechanism

6.1 C–C activation via σ-bond metathesis

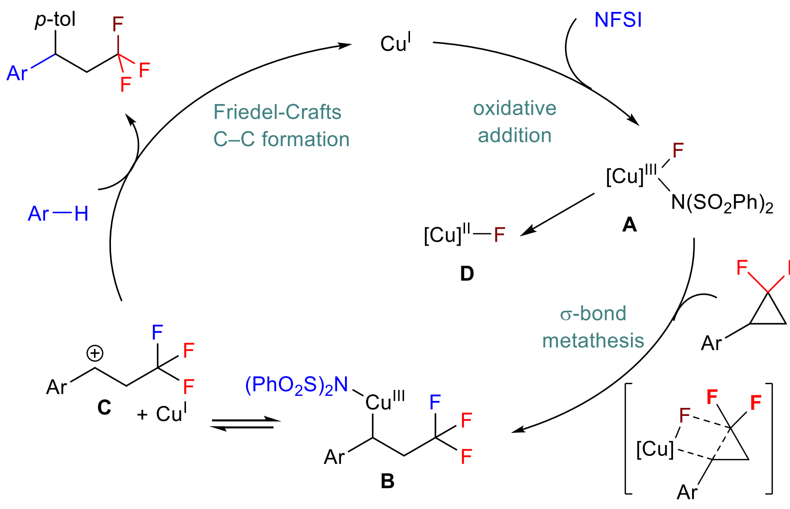

Initially, Cu(I) is oxidized by NFSI to produce F–Cu(III)–N(PhSO_2)$_2$ species **A**, which is highly electrophilic and may activate the proximal C–C bond in *gem*-DFCP possibly via a σ-bond metathesis to form a benzyl–Cu(III) species **C**. Finally, complex **C** is equilibrated with the Cu(I) and benzyl cation species, which is then captured by an electron-rich arene via Friedel-Crafts process to produce the fluoroarylation product. Meanwhile, it is also possible that the F–Cu(III)–N(PhSO_2)$_2$ species **A** undergoes comproportionation with the Cu(I) or releases a nitrogen radical to form a Cu(II)–F species **D**, which then triggers the C–C bond activation.

6.2 C–C activation via stepwise mechanism involving SET process

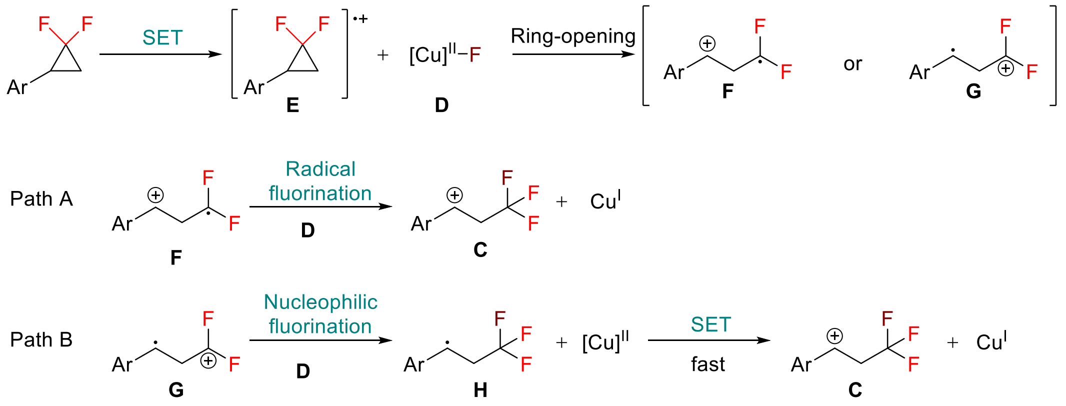

As suggested by the reviewers, while the final formation of a benzylic cation **C** is well supported by the control experiments, the way to go to it is less conclusive. In addition to the proposed σ-bond metathesis, we cannot rule out the possibility of a stepwise mechanism involving SET process to cleave the C–C bond that eventually forms the benzylic carbocation **C**. In this situation, F–Cu(III)–N(PhSO_2)$_2$

species **A** may react with the *gem*-DFCP to give a Cu(II)–F species **D** and a *gem*-DFCP radical cation **E**. The radical cation may be less stable, and open up to form another radical cation with a radical in either benzylic position or between the two fluorines (**F** or **G**). Then, the species **F** converts to benzyl cation species **C** through radical fluorination (**path A**). On the other hand, the species **G** may convert to benzyl radical **H** through nucleophilic fluorination followed by another fast SET process to give the benzylic carbocation **C** (**path B**). Nevertheless, further inverstigations are required to better understand the details of the reaction mechanism.

7. References

(1) F. Wang, T. Luo, J. Hu, Y. Wang, H. S. Krishnan, P. V. Jog; S. K. Ganesh, G. K. Prakash, G. A. Olah, *Angew. Chem., Int. Ed.* **2011**, *50*, 7153−7157; *Angew. Chem*. **2011**, *123*, 7291–7295.

(2) Z.-T. Jiang, J. Huang, Y. Zeng, F. Hu, Y. Xia, *Angew. Chem., Int. Ed*. **2021**, *60*, 10626–10631; *Angew. Chem*. **2021**, *133*, 10720–10725.

(3) A. Suliman, E. M. Ahmed, T. Gong, Y. Fu, *Chem. Commun*. **2021**, *57*, 6400–6403.

(4) Z. Fu, J. Zhu, S. Guo, A. Lin, *Chem. Commun.* **2021**, *57*, 1262–1265.

(5) J. Ni, B. Nishonov, A. Pardaev, A. Zhang, *J. Org. Chem.* **2019**, *84*, 13646–13654.

(6) P. S. Nosik, S. V. Ryabukhin, O. O. Grygorenko, D. M. Volochnyuk, *Adv. Synth. Catal*., **2018**, *360*, 4104–4114.

(7) J. Xu, E. A. Ahmed, B. Xiao, Q.-Q. Lu, Y.-L. Wang, C. G. Yu, Y. Fu, *Angew. Chem., Int. Ed*. **2015**, *54*, 8231–8235; *Angew.Chem*. **2015**, *127,* 8349–8353.

(8) Y. Zeng, Y. Xia. *Angew. Chem. Int. Ed*. **2023**, e202307129; *Angew. Chem*. **2023**,*135,* e202307129.

(9) X. Wu, Y. Zeng, Z.-T. Jiang, Y. Zhu, L. Xie, Y. Xia, *Org. Lett*. **2022**, *24*, 8429−8434.

(10) Y. Zeng, H. Yang, J. Du, Q. Huang, G. Huang, Y. Xia, *Chem. Sci.,* **2022**, *13*, 12419−12425.

(11) L. Lv, C.-J. Li, *Angew. Chem., Int. Ed*. **2021**, *60,* 13098–13104; *Angew.Chem*. **2021**,*133*,13208–13214.

(12) K. Oshiro, Y. Morimoto, H. Amii, *Synthesis,* **2010**, *12*, 2080−2084.

(13) L. Wu, F. Wang, X. Wan, D. Wang, P. Chen, G. Liu, *J. Am. Chem. Soc*. **2017**, *139*, 2904–2907.

(14) M. T. Nunez, V. S. Martin, *J. Org. Chem*. **1990**, *55*, 1928–1932.

(15) H. Liu, Y. Li, D.-X. Wang, M.-M. Sun, C. Feng, *Org. Lett*. **2020**, *22*, 8681−8686.

(16) S. Yang, L. Wang, H. Zhang, C. Liu, L. Zhang, X. Wang, G. Zhang, Y. Li, Q. Zhang, *ACS Catal*. **2019**, *9*, 716−721.

(17) X.-P. Wang, J.-H. Lin, C.-P. Zhang, J.-C. Xiao, X. Zheng, *Beilstein J. Org. Chem*. **2013**, *9,* 2635–2640.

(18) Z.-T. Jiang, Z. Chen, Y. Zeng, J.-L. Shi, Y. Xia, *Org. Lett*. **2022**, *24*, 6176−6181.

8. NMR Spectra

^{1}H NMR (400 MHz, $CDCl_3$) spectrum of 1r

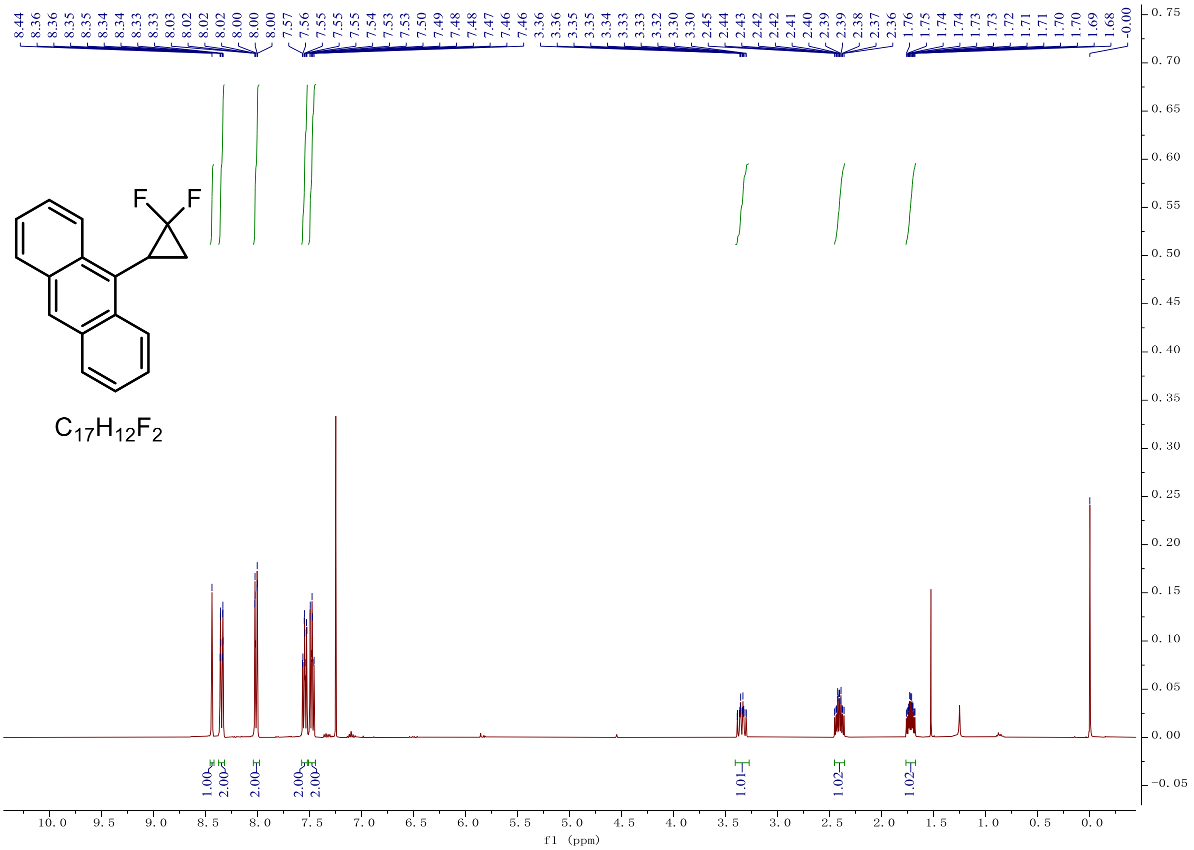

^{13}C NMR (101 MHz, $CDCl_3$) spectrum of 1r

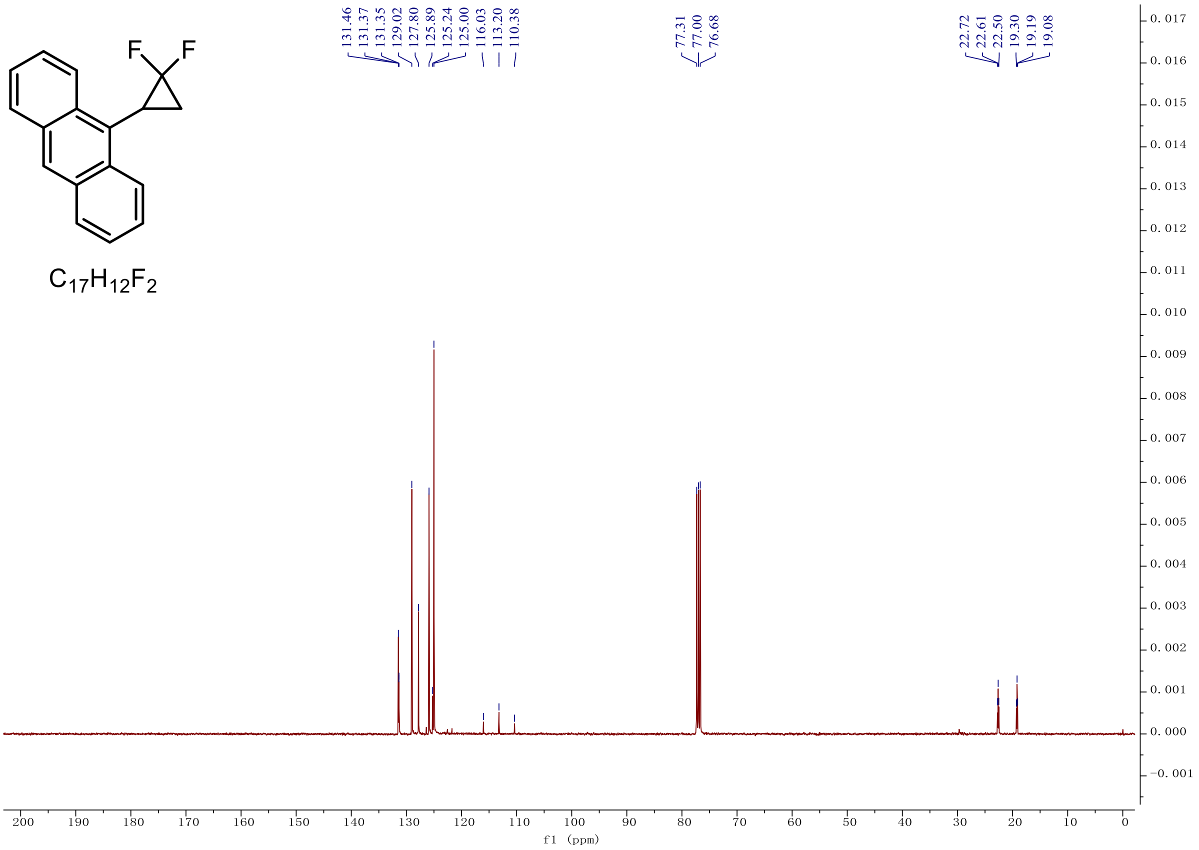

^{19}F NMR (376 MHz, CDCl$_3$) spectrum of 1r

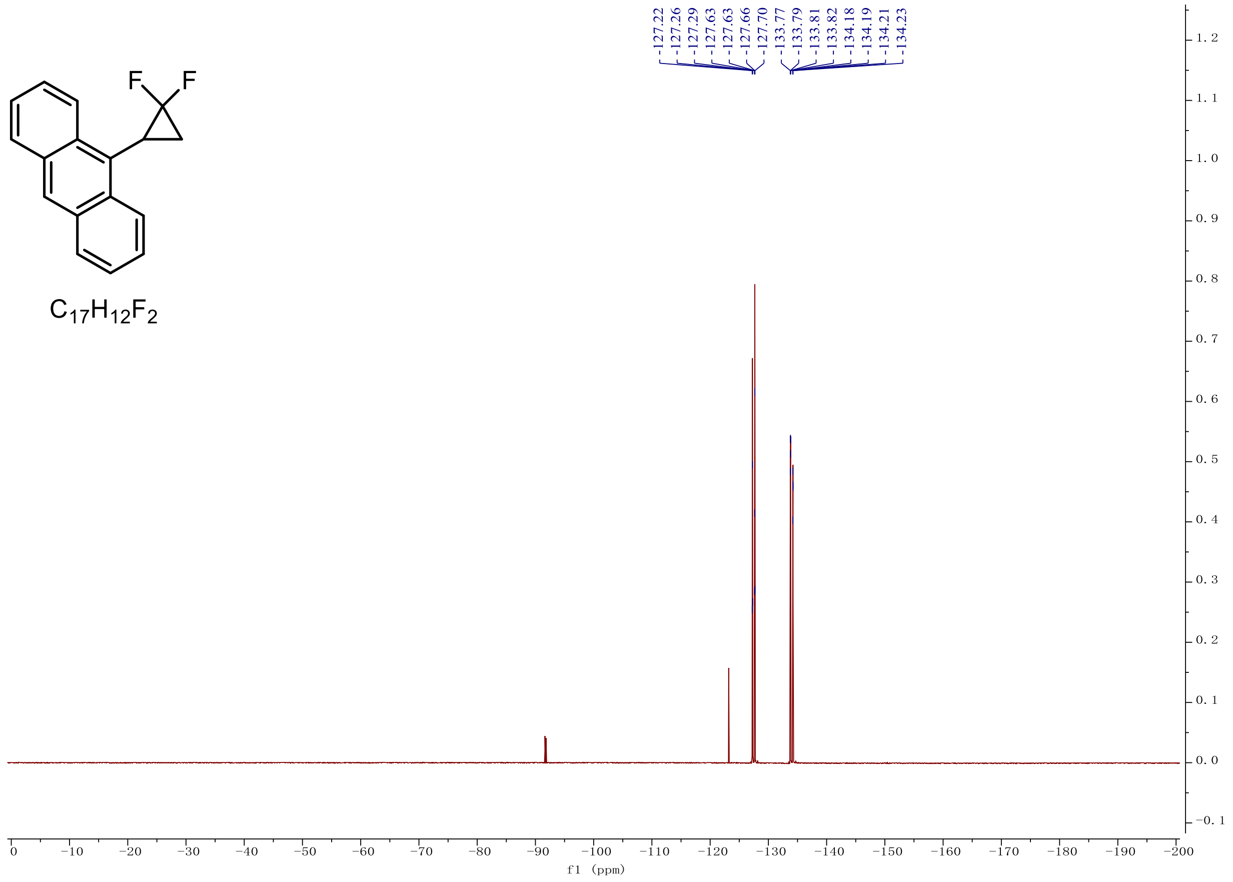

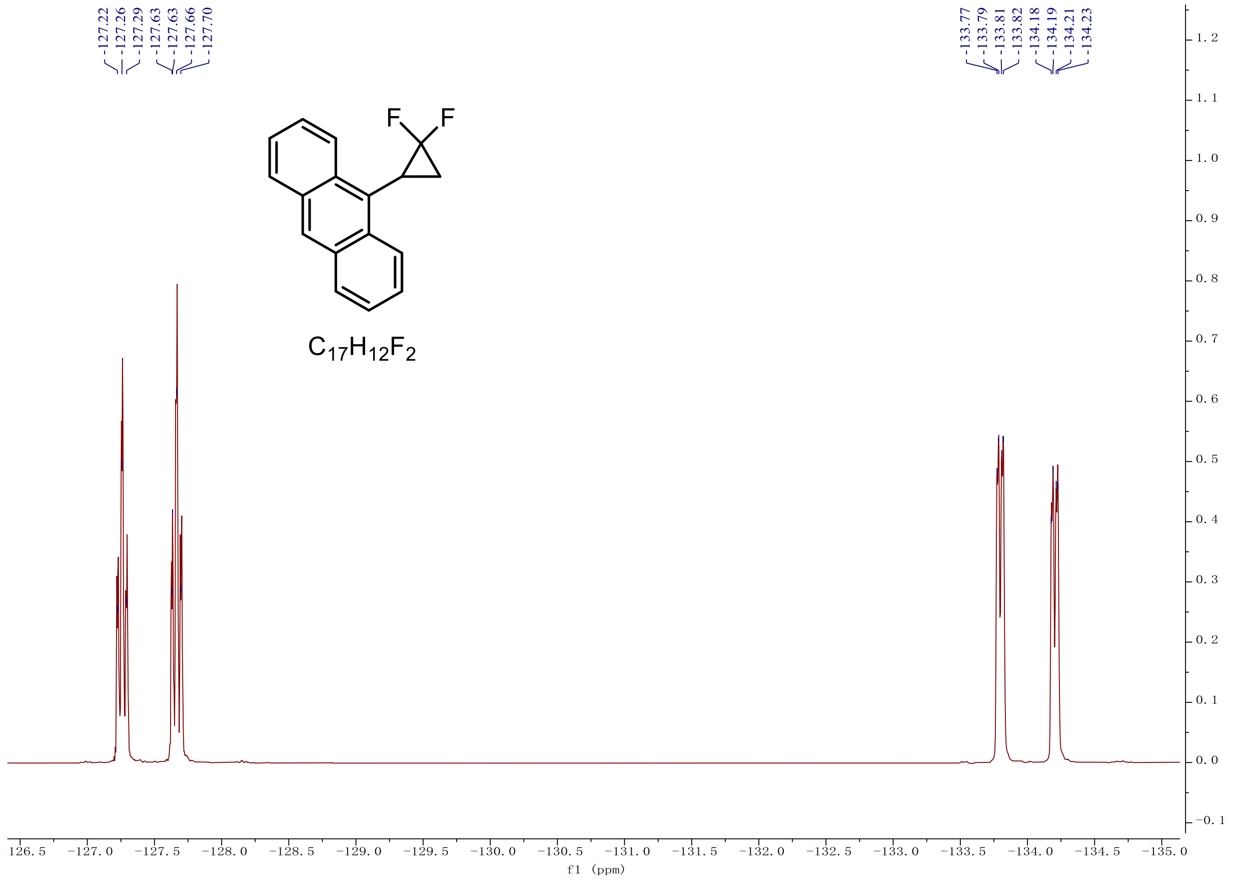

NMR (400 MHz, CDCl3) spectrum of 2a

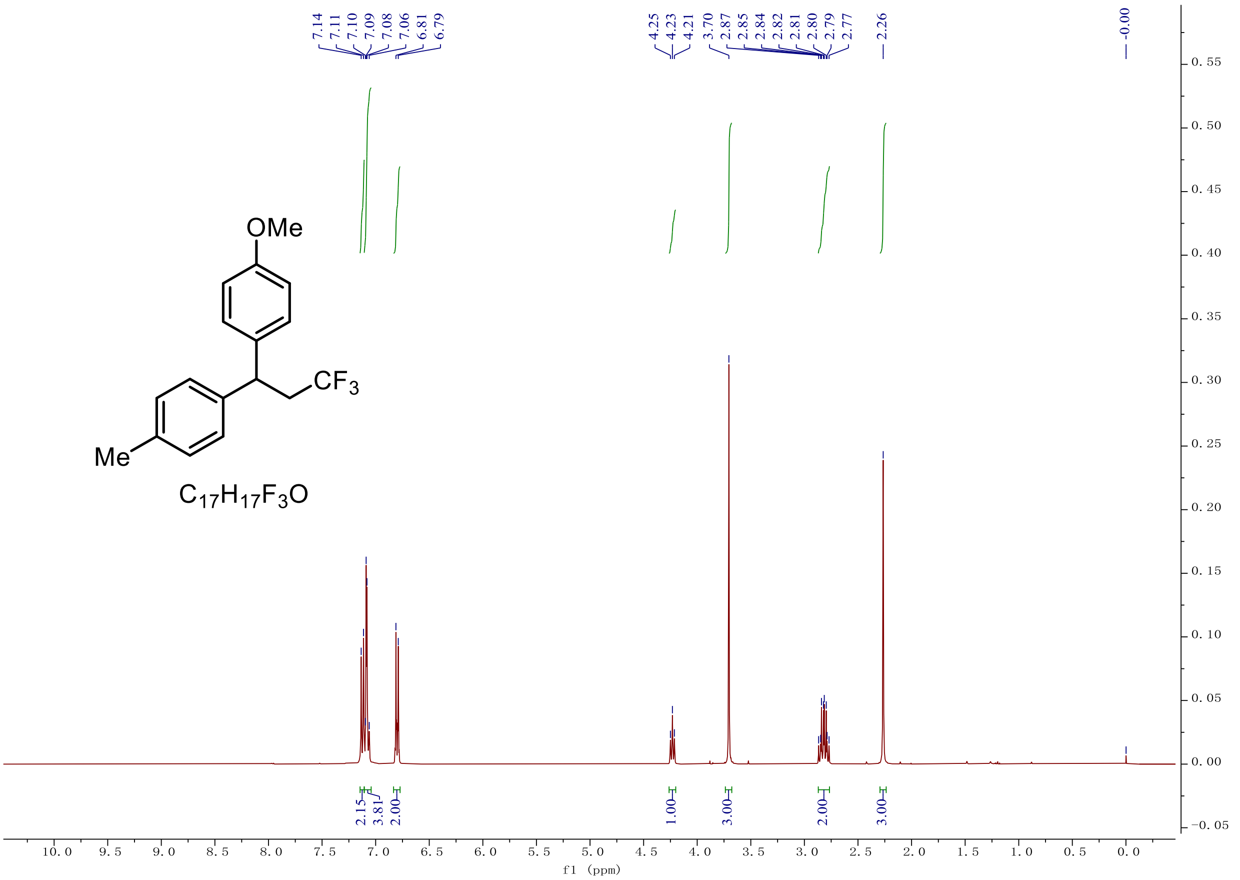

^{13}C NMR (101 MHz, CDCl3) spectrum of 2a

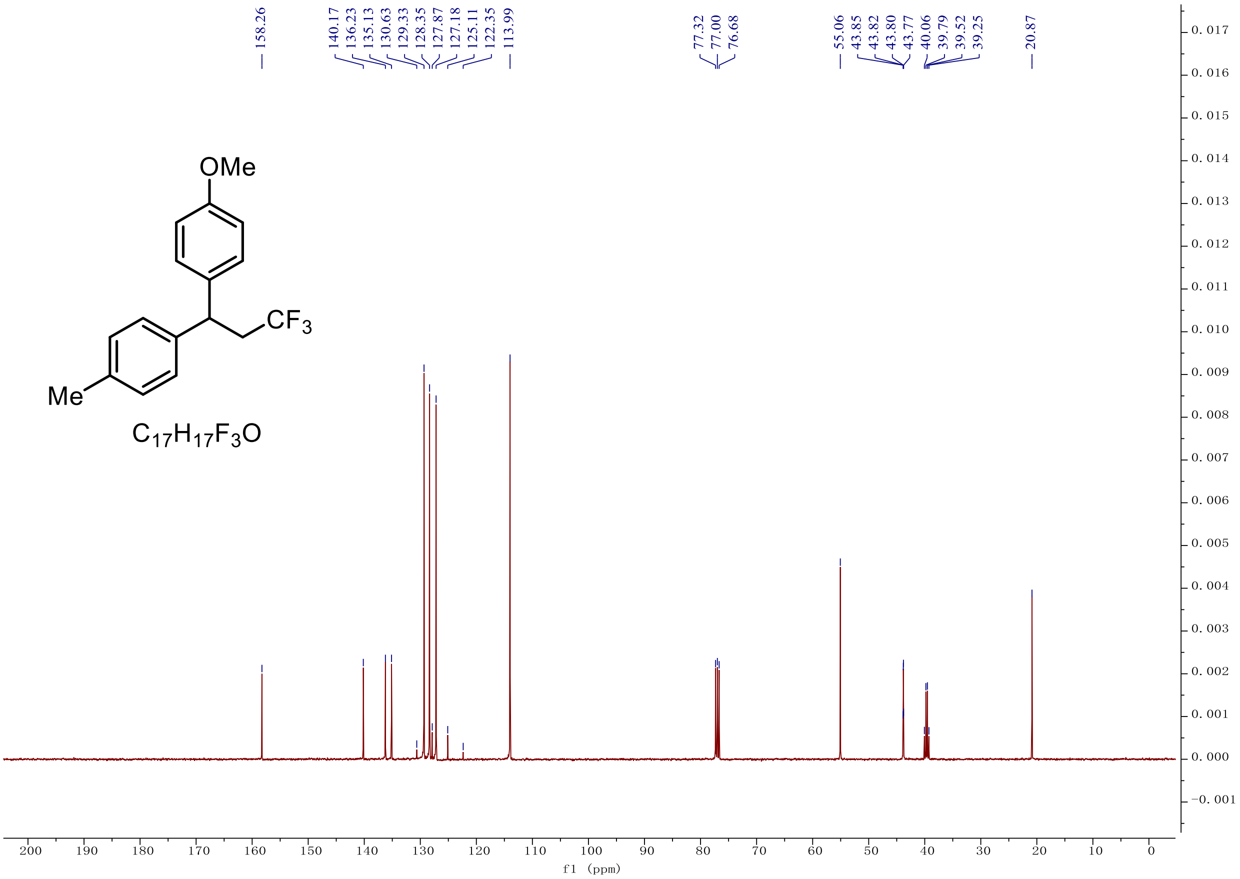

^{19}F NMR (376 MHz, $CDCl_3$) spectrum of 2a

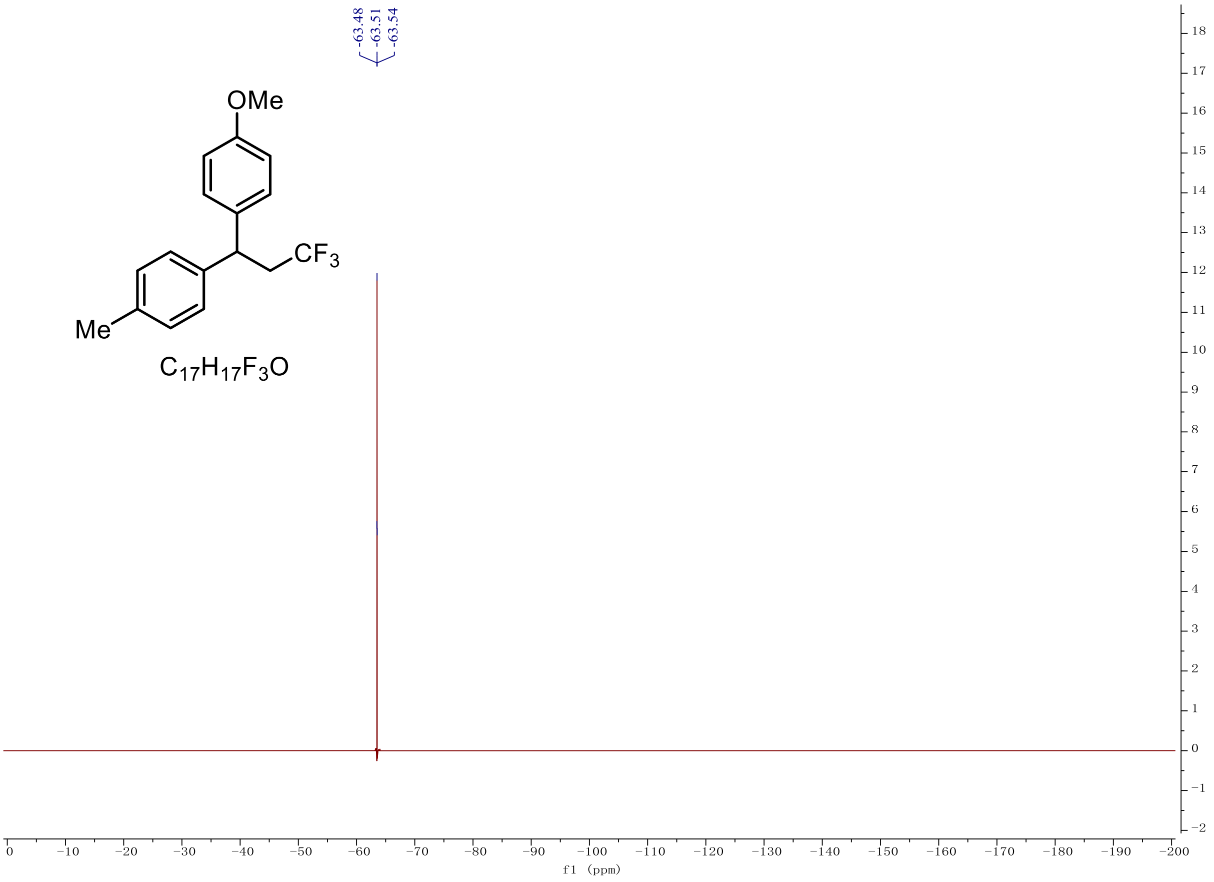

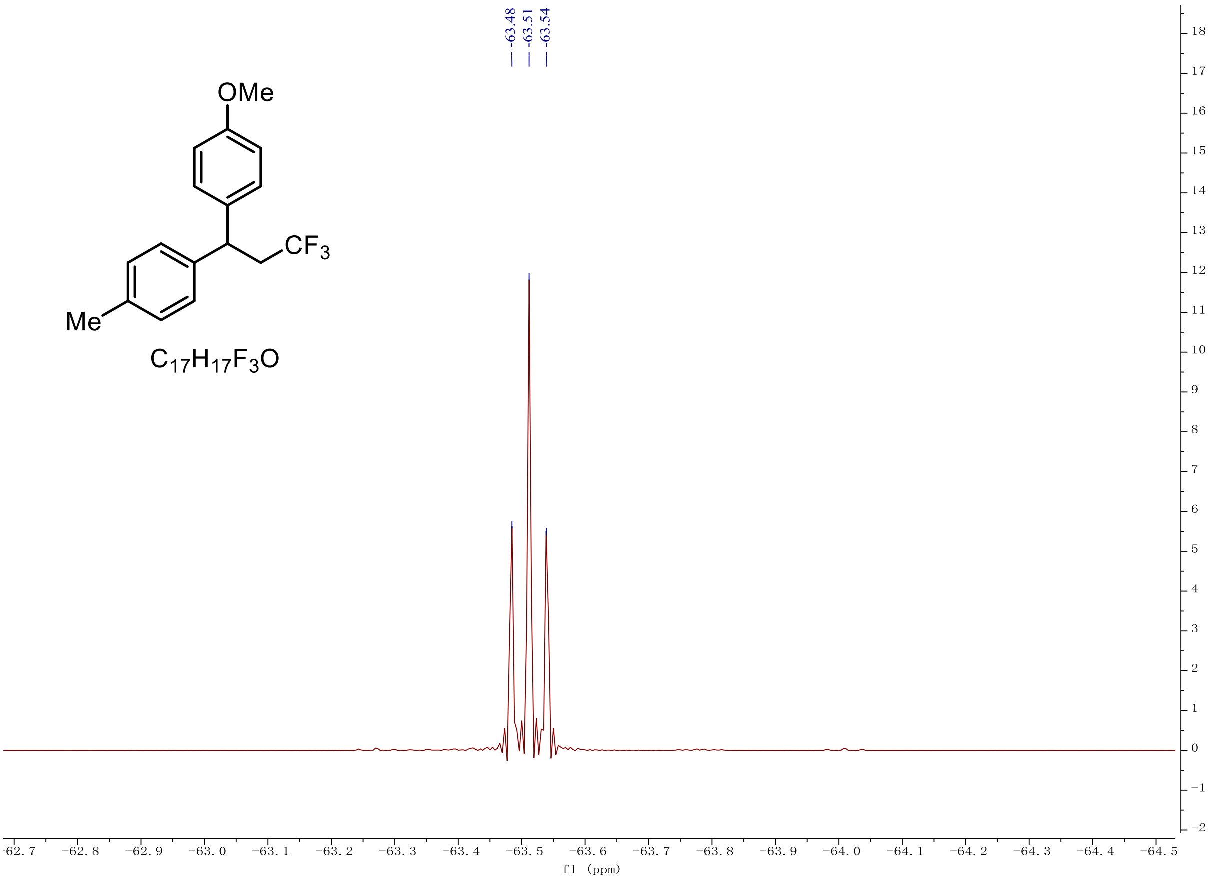

^{1}H NMR (400 MHz, CDCl3) spectrum of 2a'

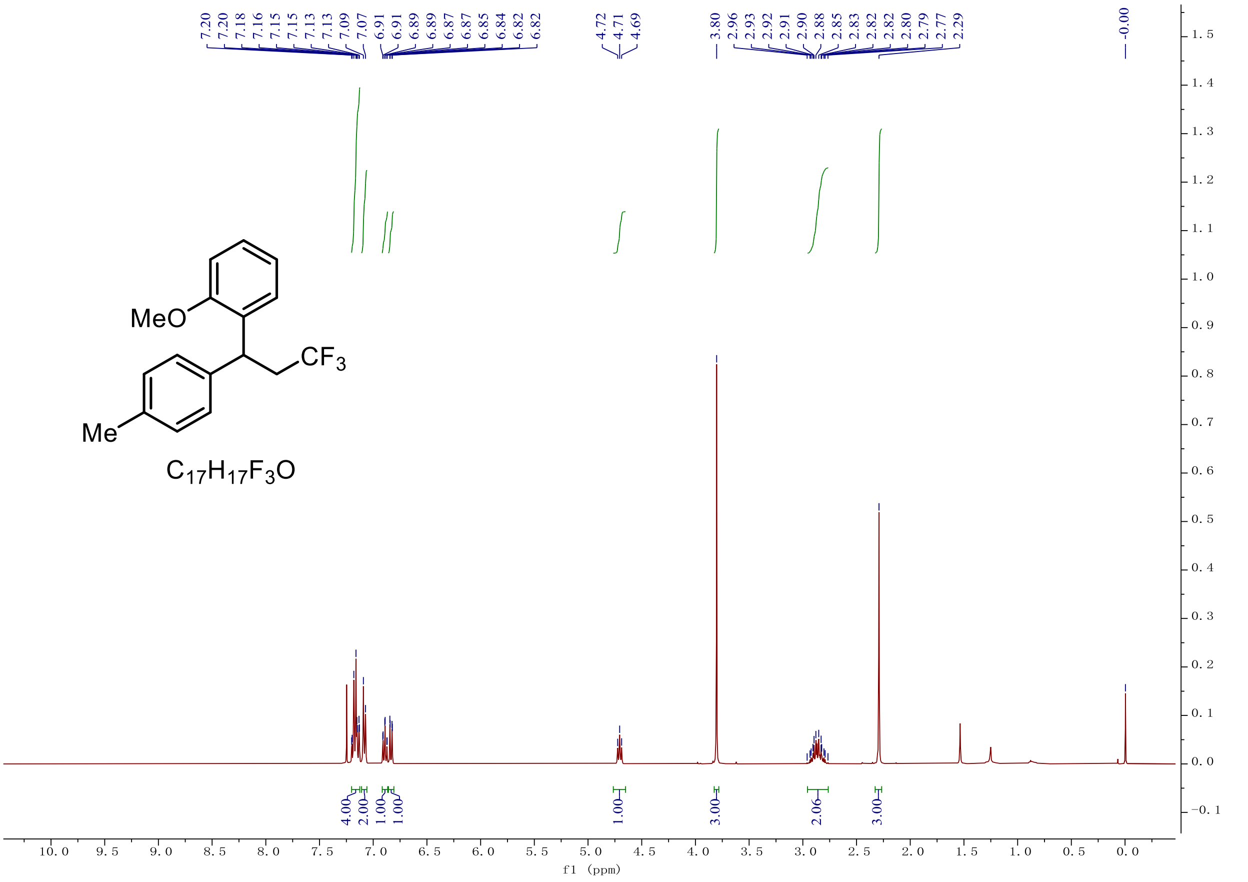

^{13}C NMR (101 MHz, CDCl3) spectrum of 2a'

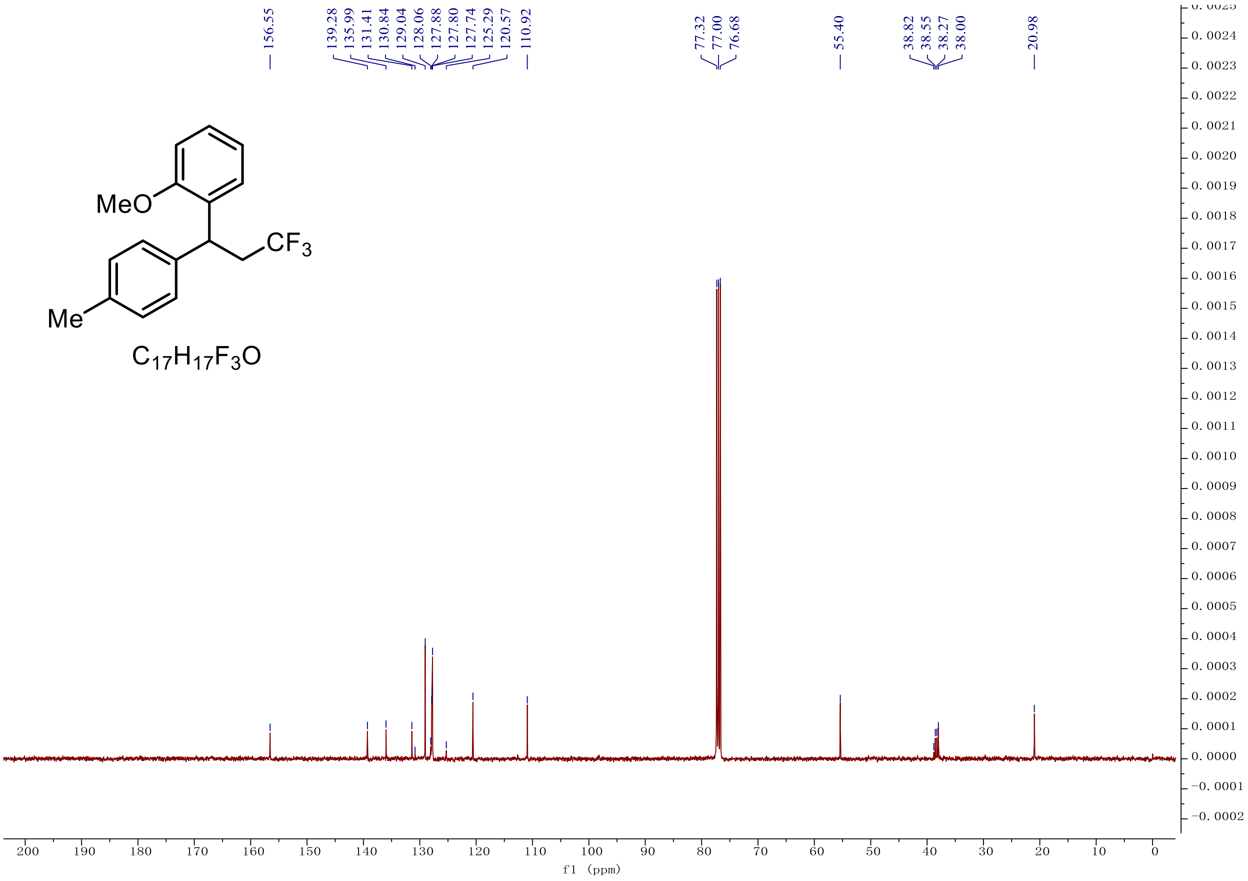

^{19}F NMR (376 MHz, $CDCl_3$) spectrum of 2a’

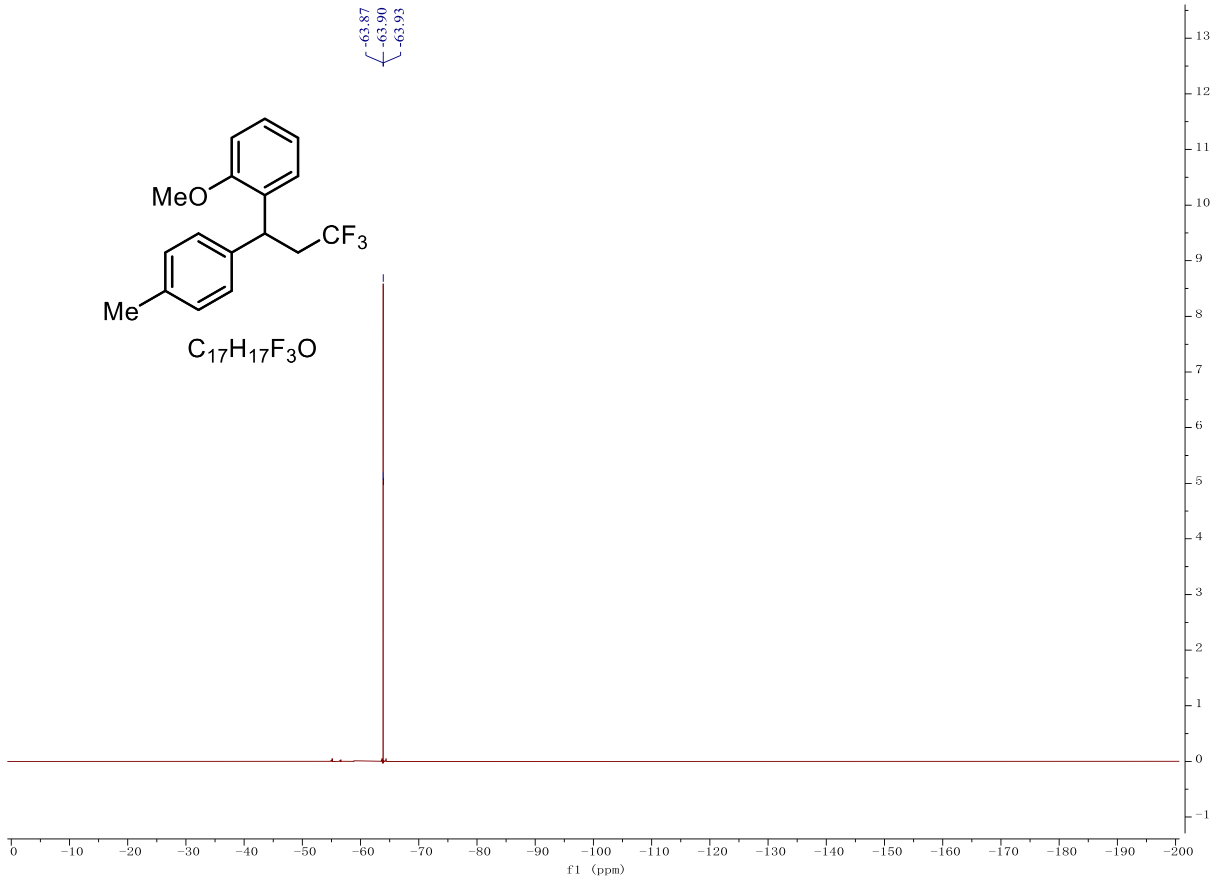

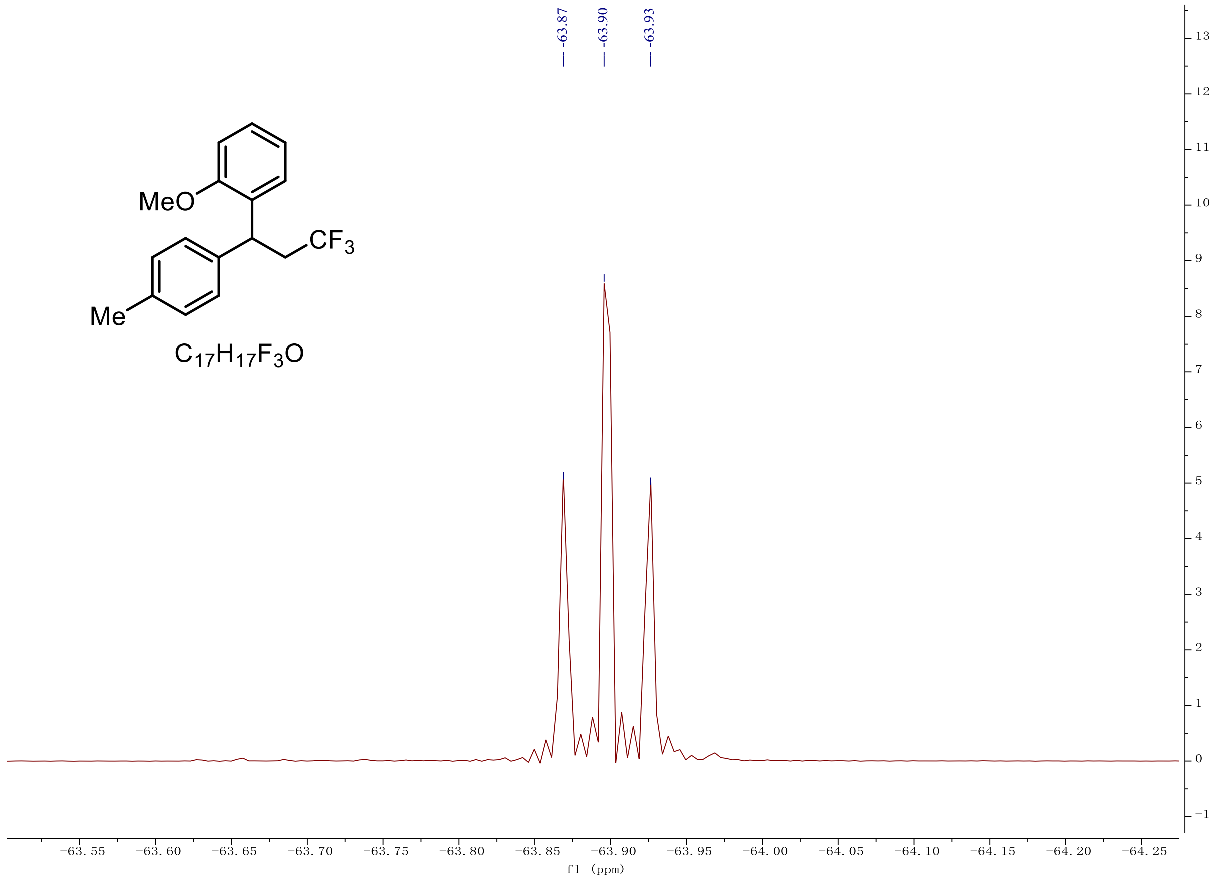

^{1}H NMR (400 MHz, $CDCl_3$) spectrum of 2b

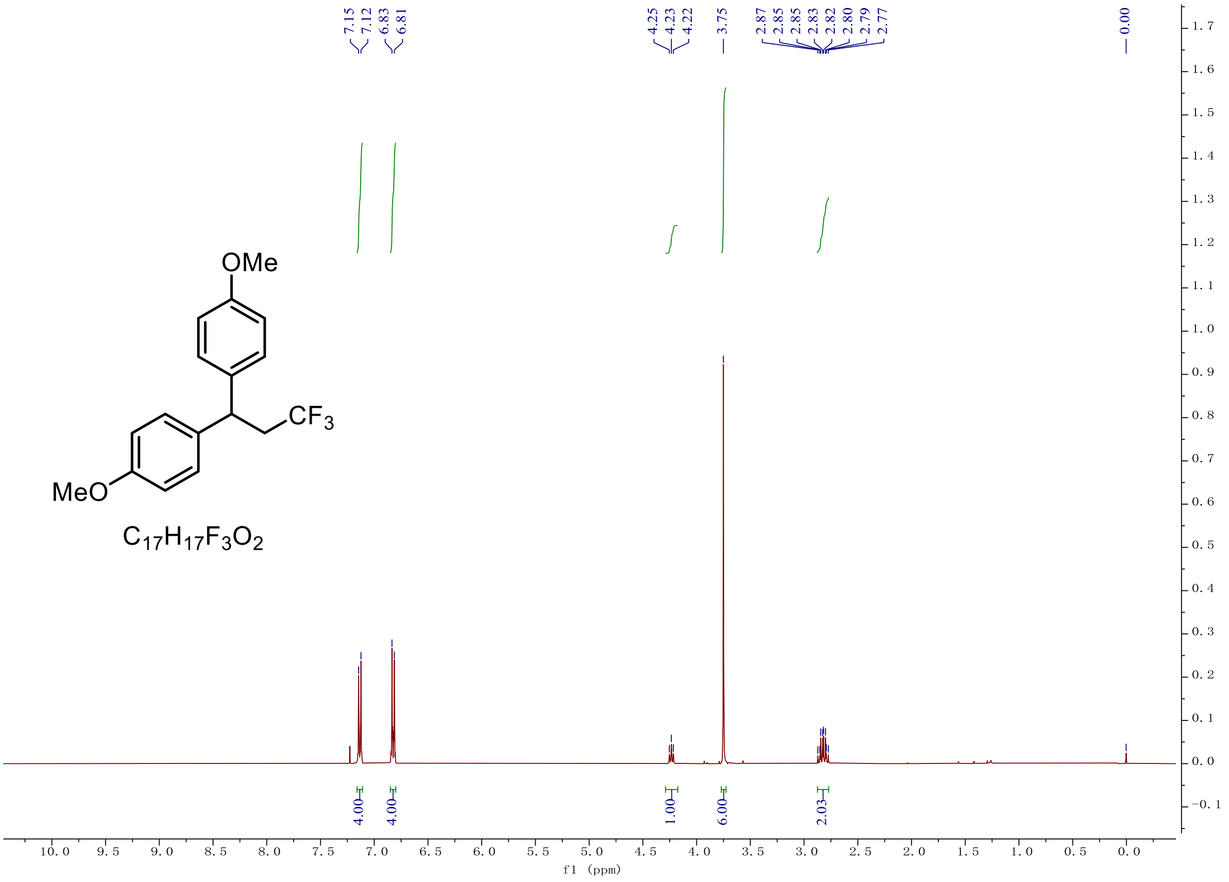

^{13}C NMR (101 MHz, $CDCl_3$) spectrum of 2b

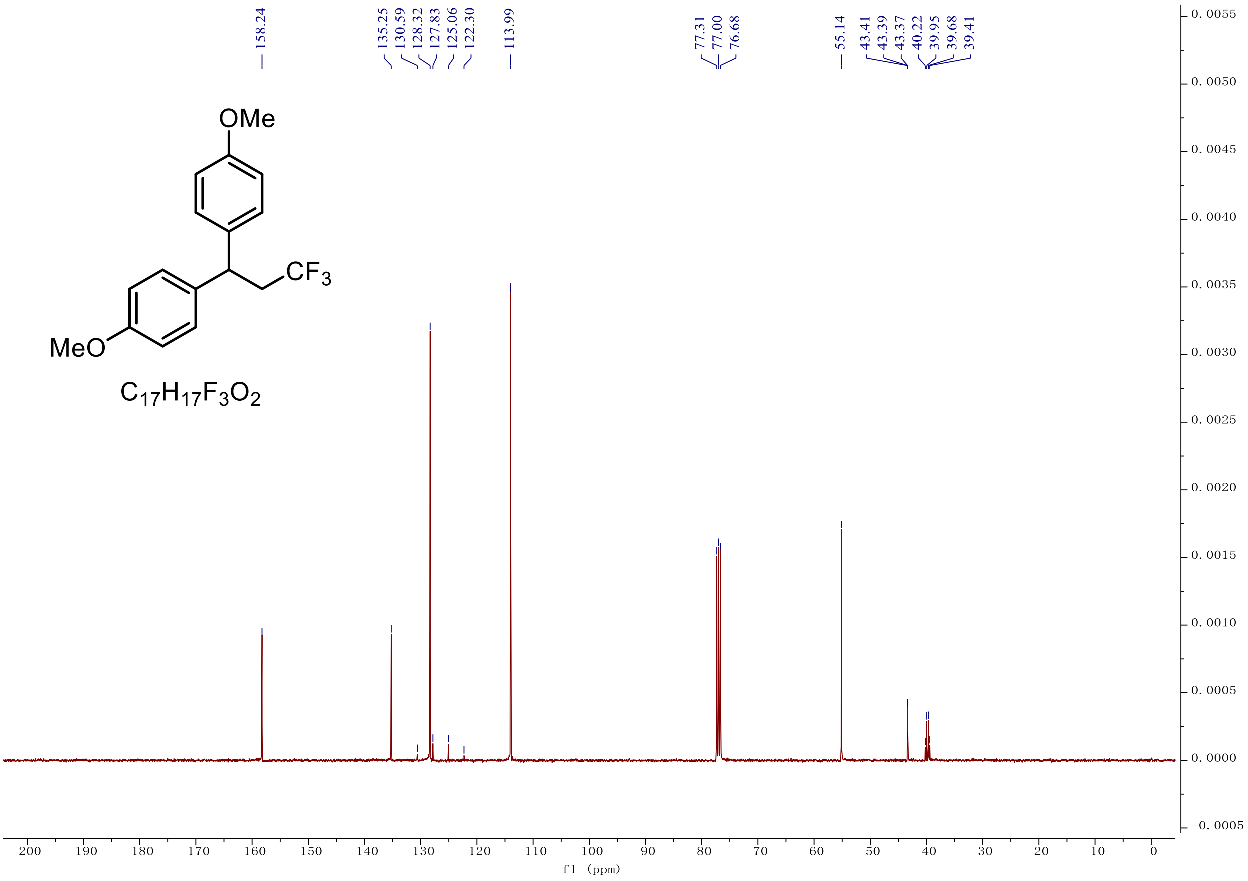

^{19}F NMR (376 MHz, $CDCl_3$) spectrum of 2b

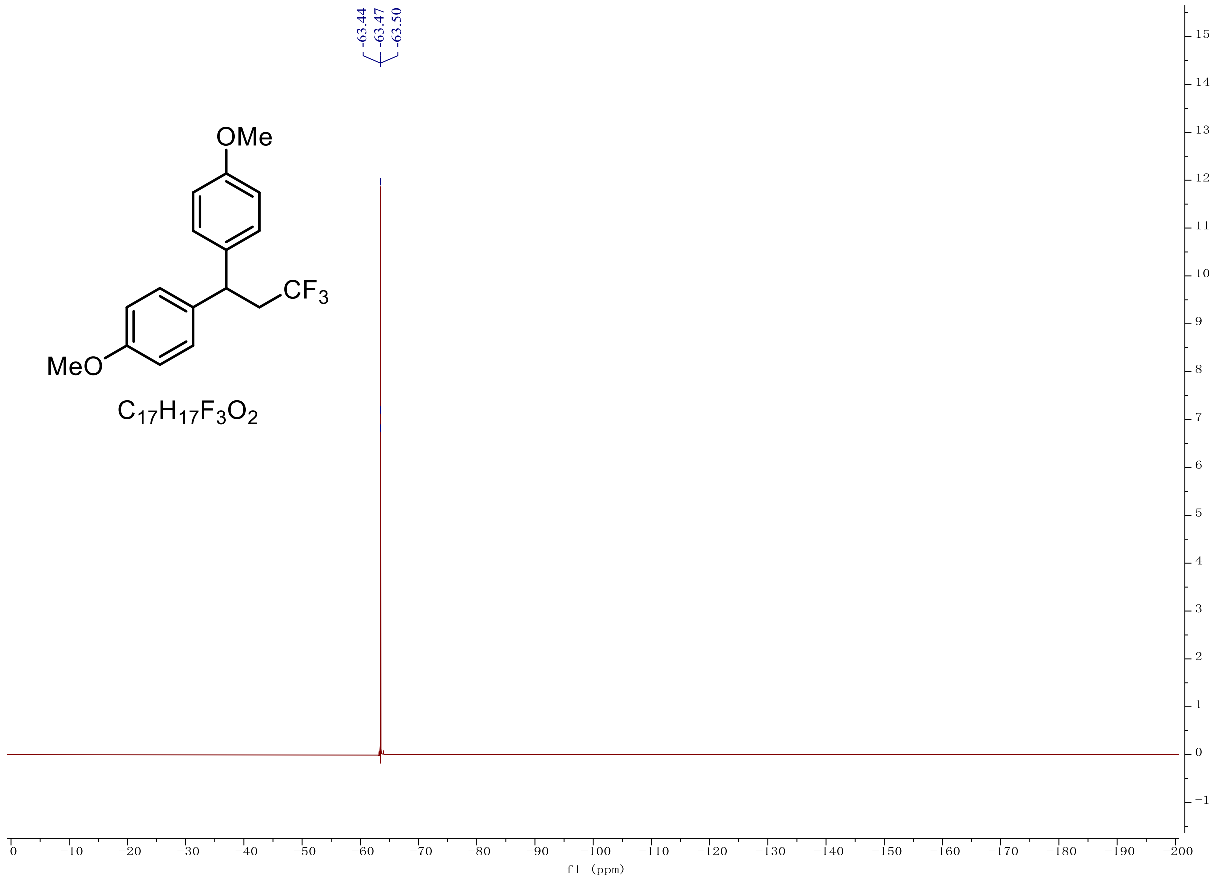

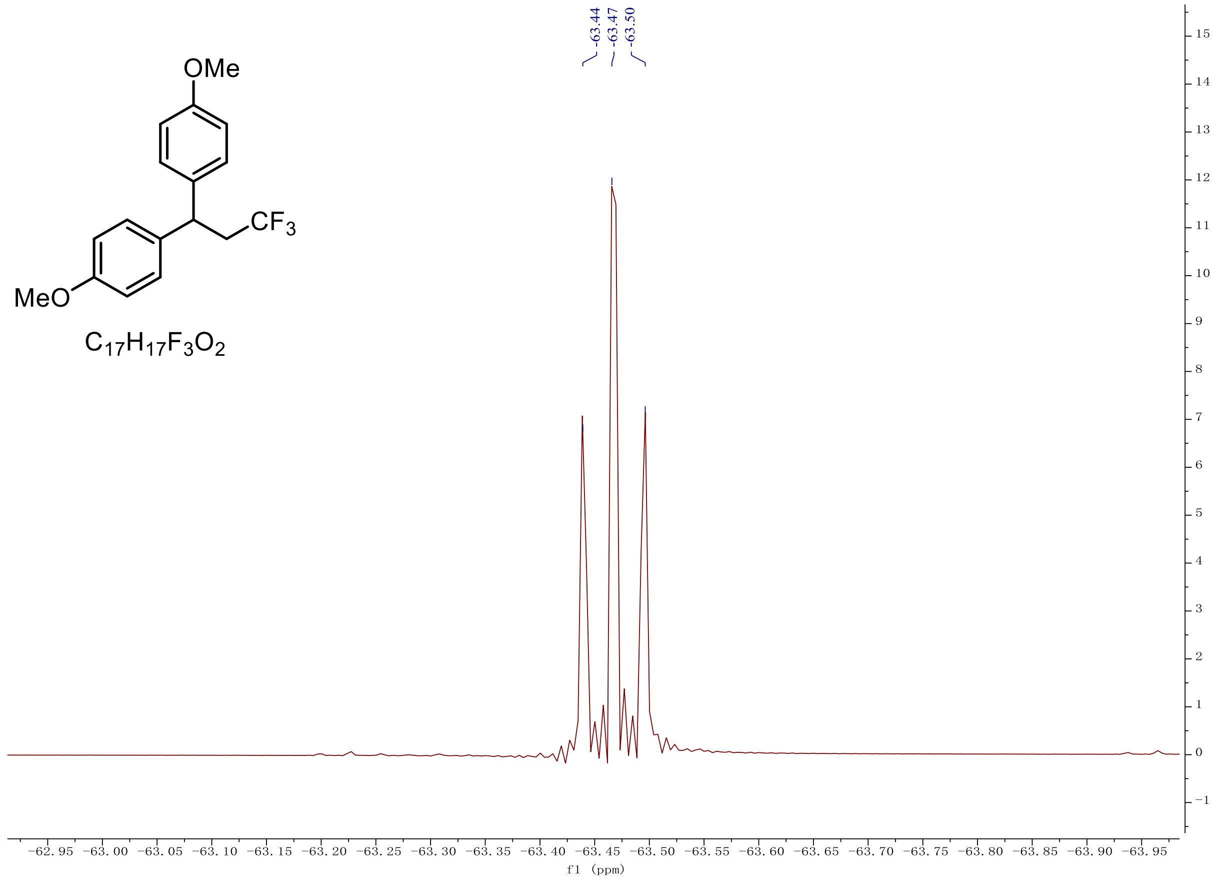

^{1}H NMR (400 MHz, $CDCl_3$) spectrum of 2c

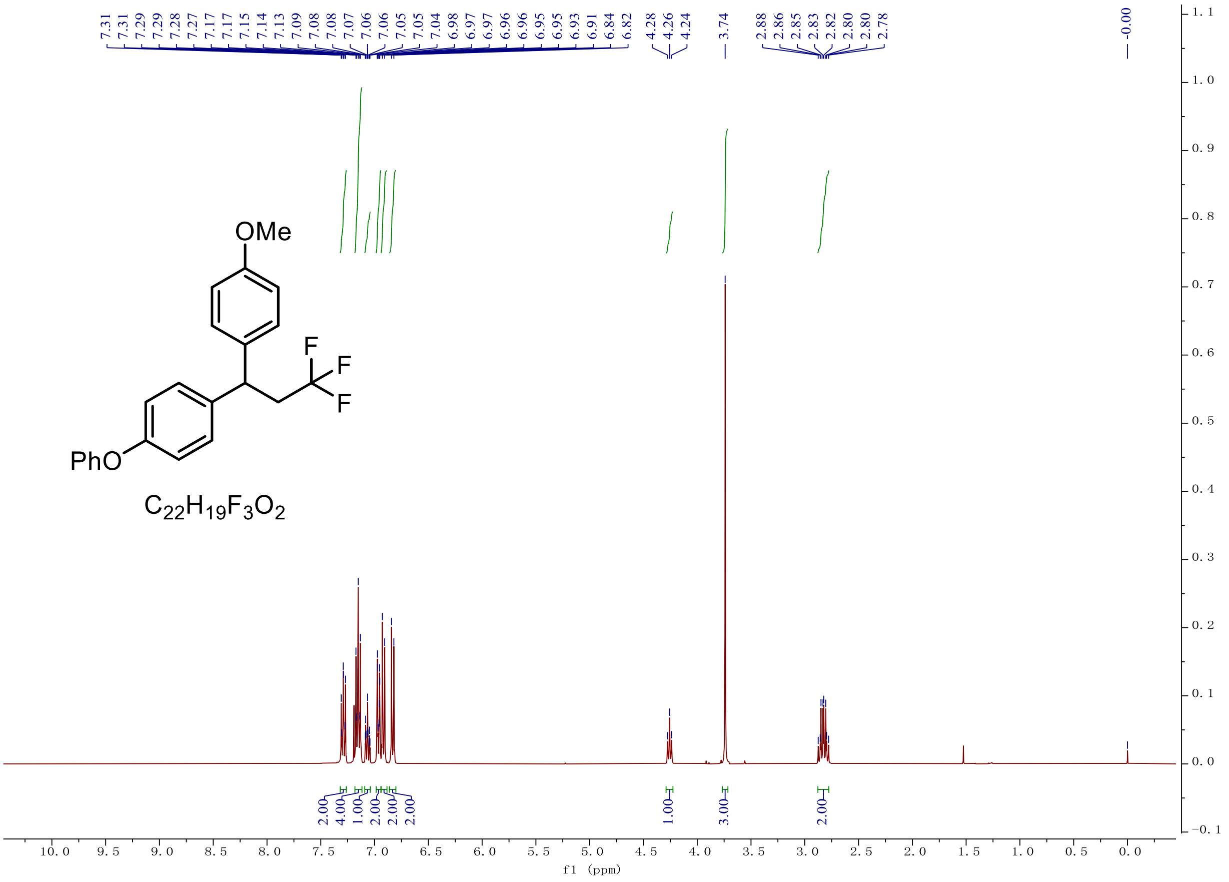

^{13}C NMR (101 MHz, $CDCl_3$) spectrum of 2c

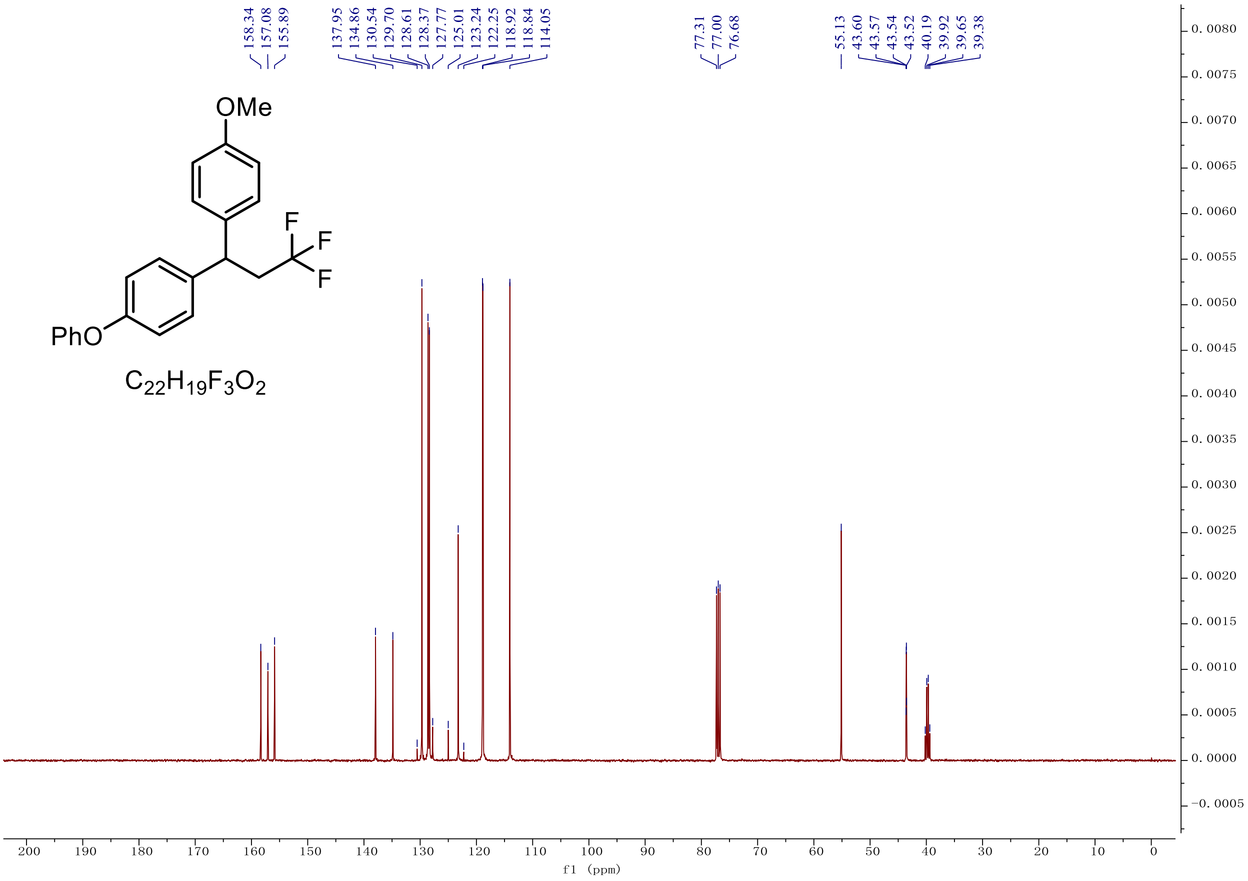

^{19}F NMR (376 MHz, $CDCl_3$) spectrum of 2c

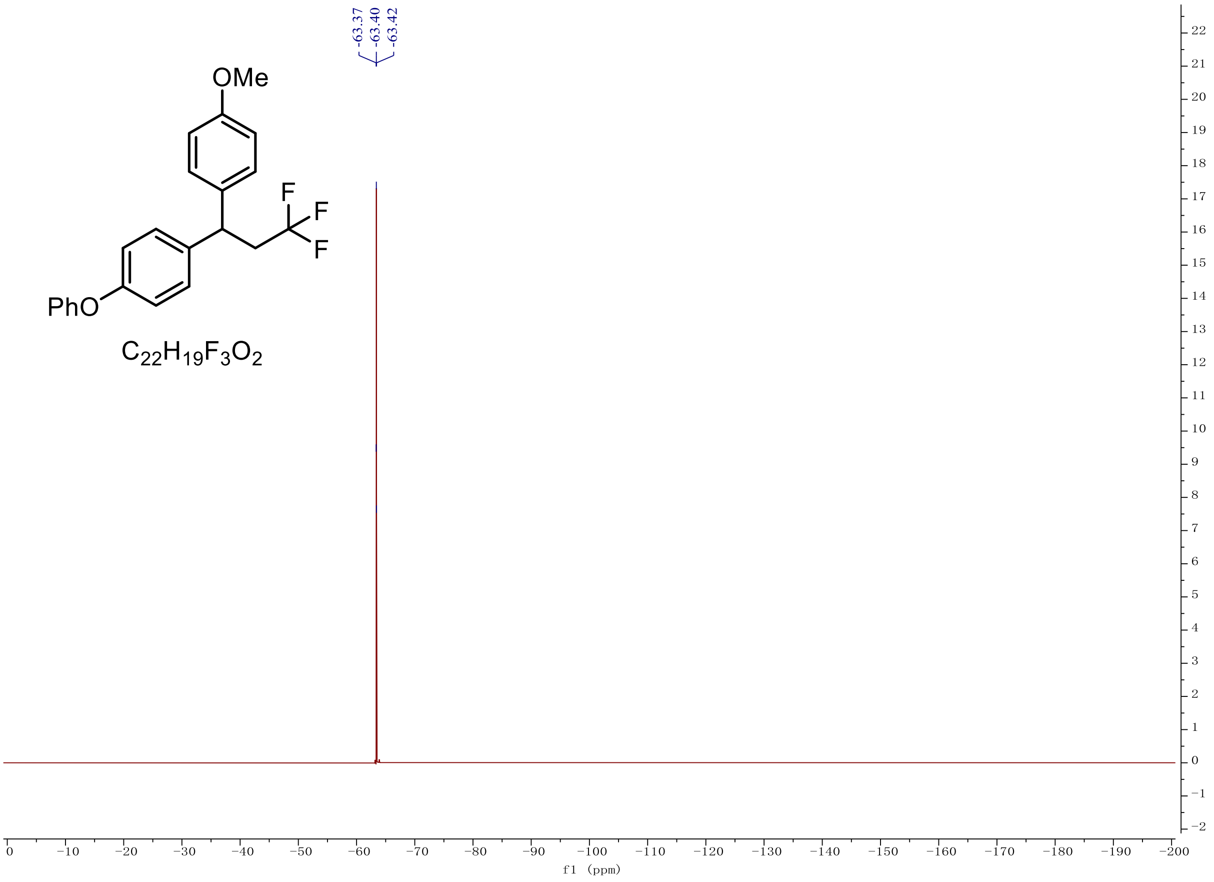

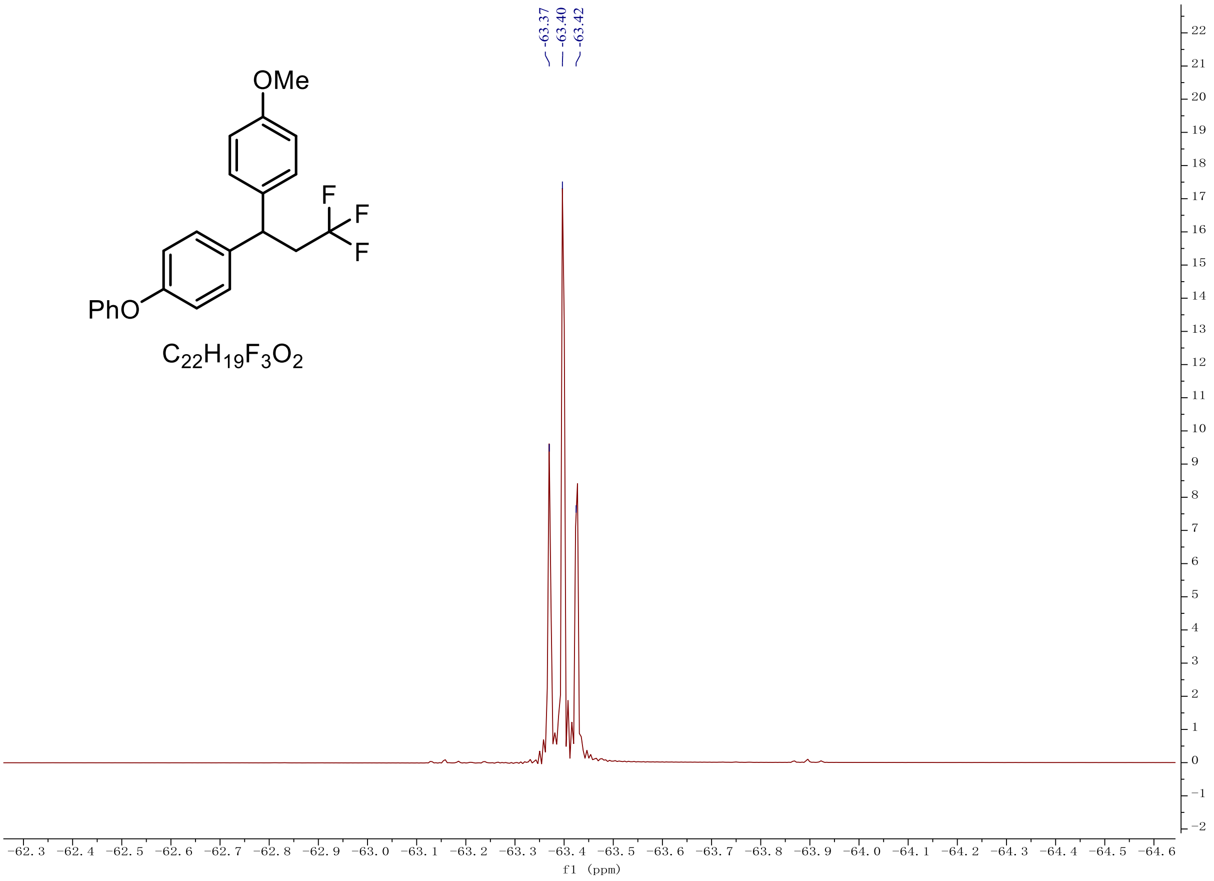

^{1}H NMR (400 MHz, $CDCl_3$) spectrum of 2d

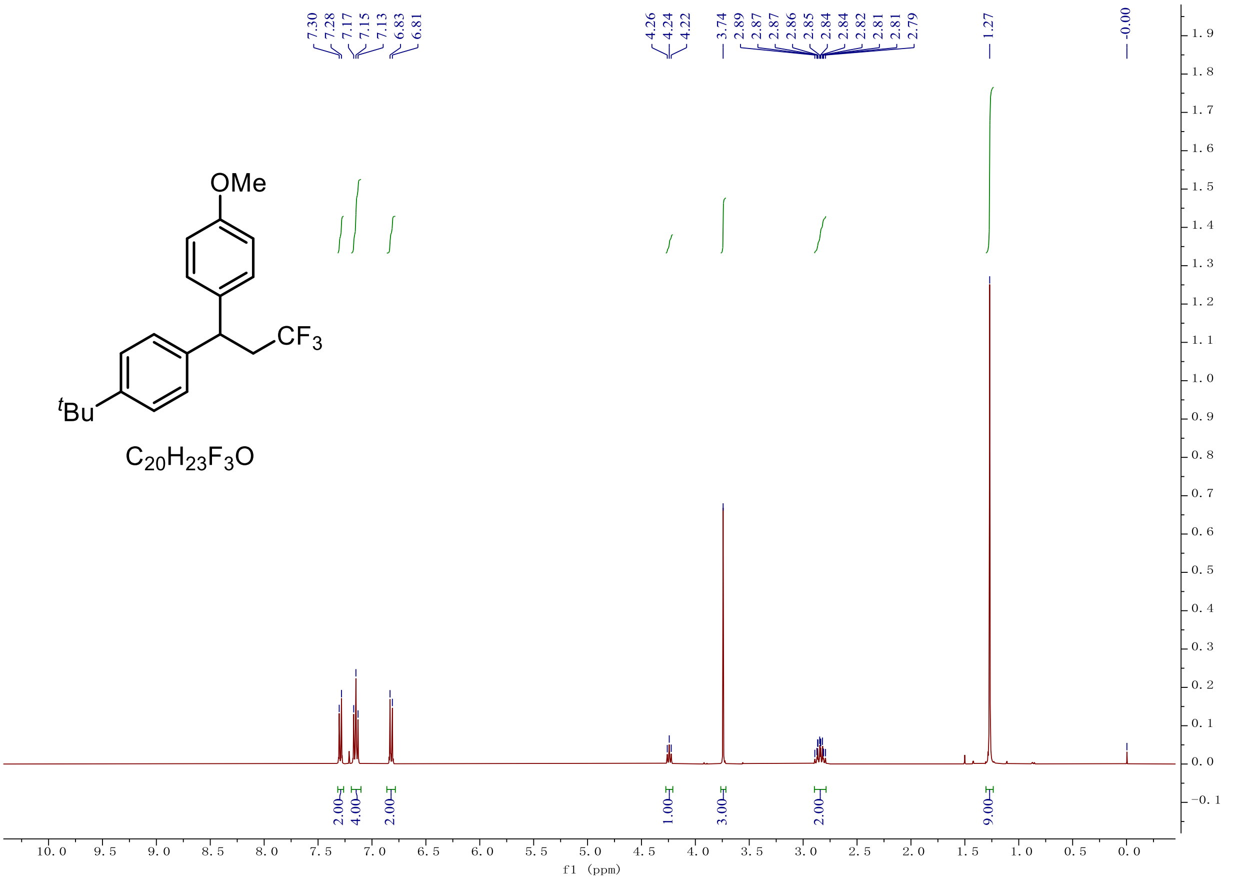

^{13}C NMR (101 MHz, $CDCl_3$) spectrum of 2d

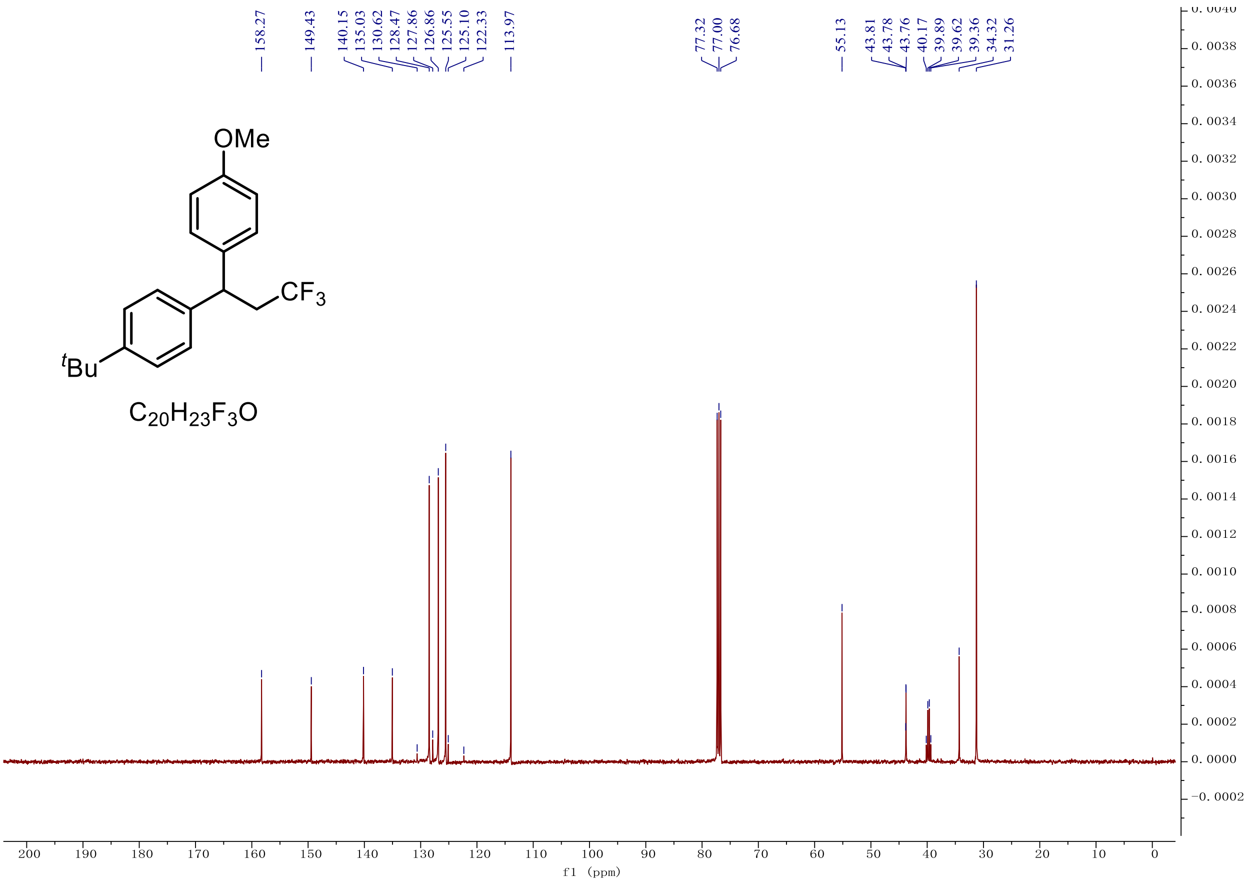

^{19}F NMR (376 MHz, $CDCl_3$) spectrum of 2d

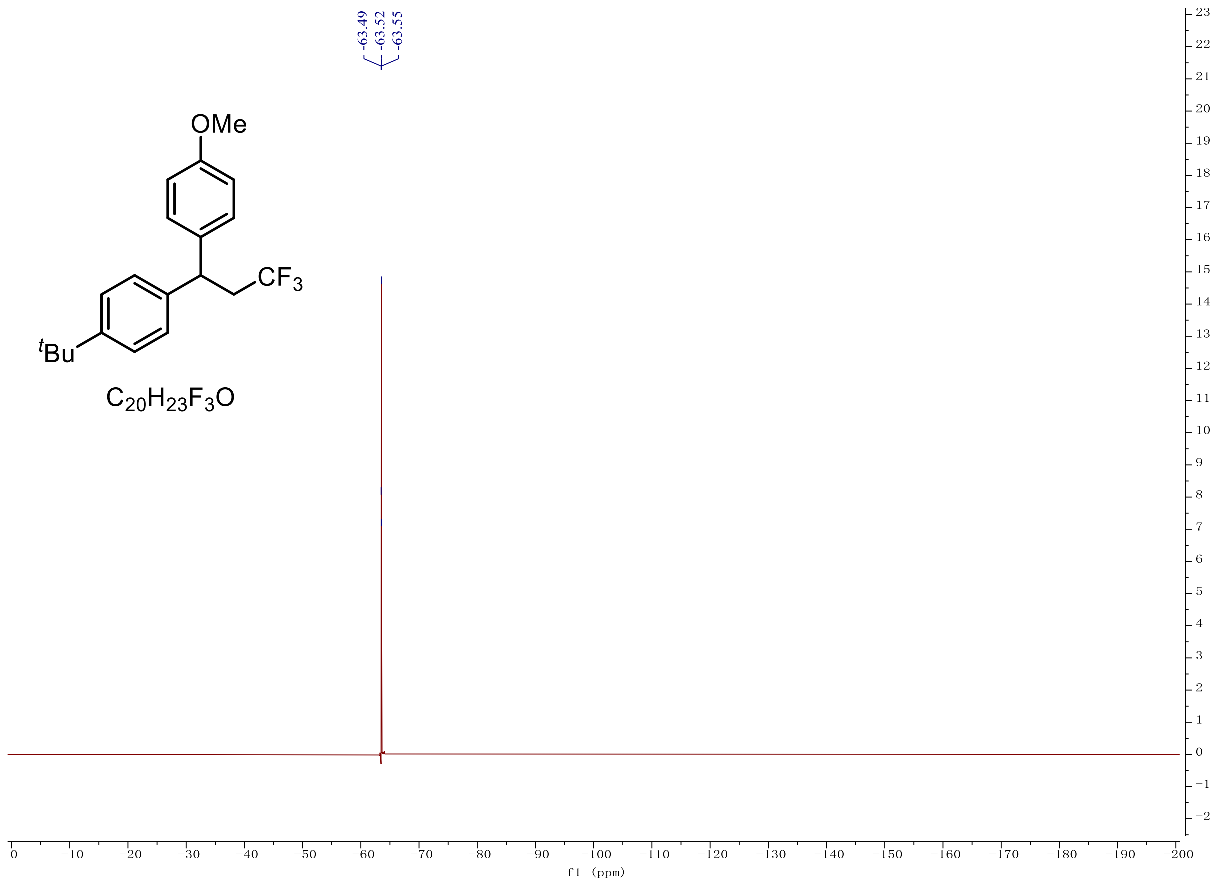

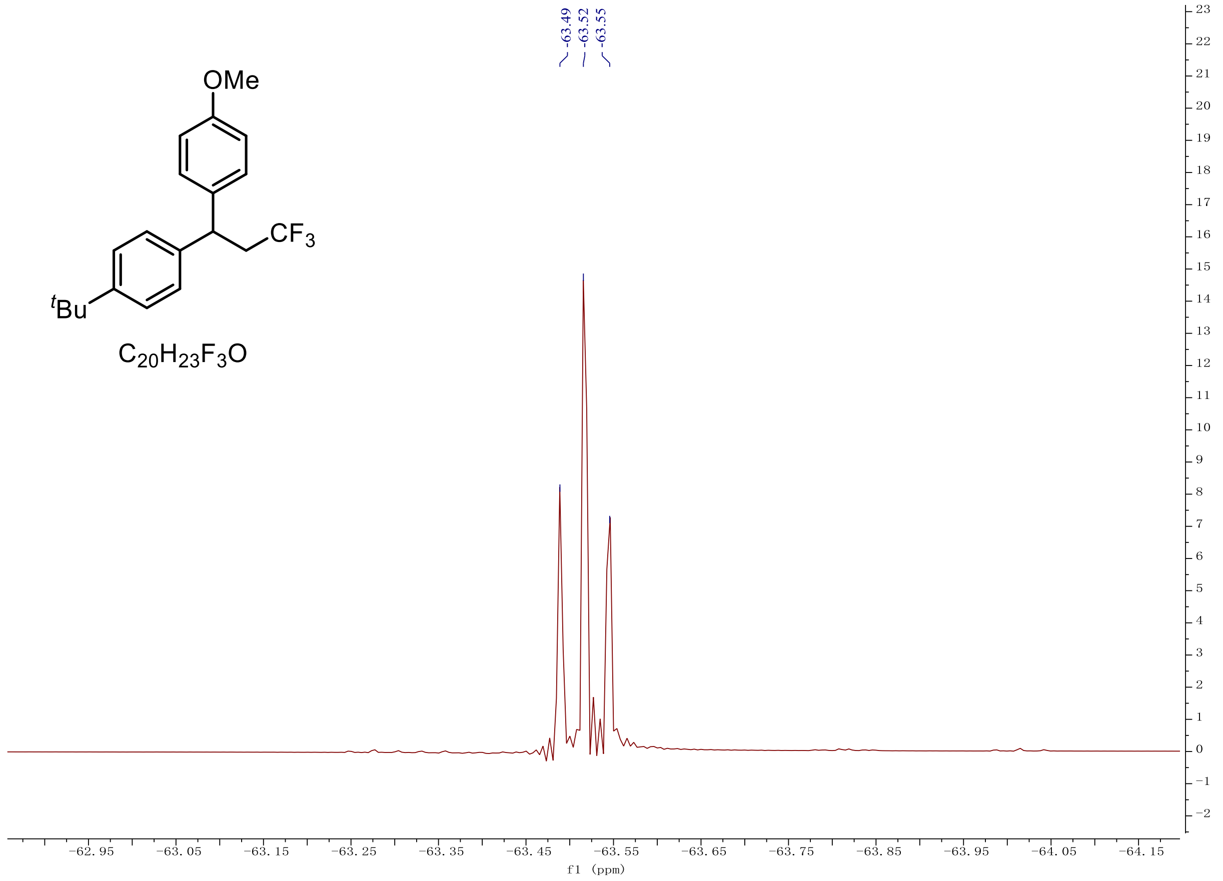

^{1}H NMR (400 MHz, $CDCl_3$) spectrum of 2e

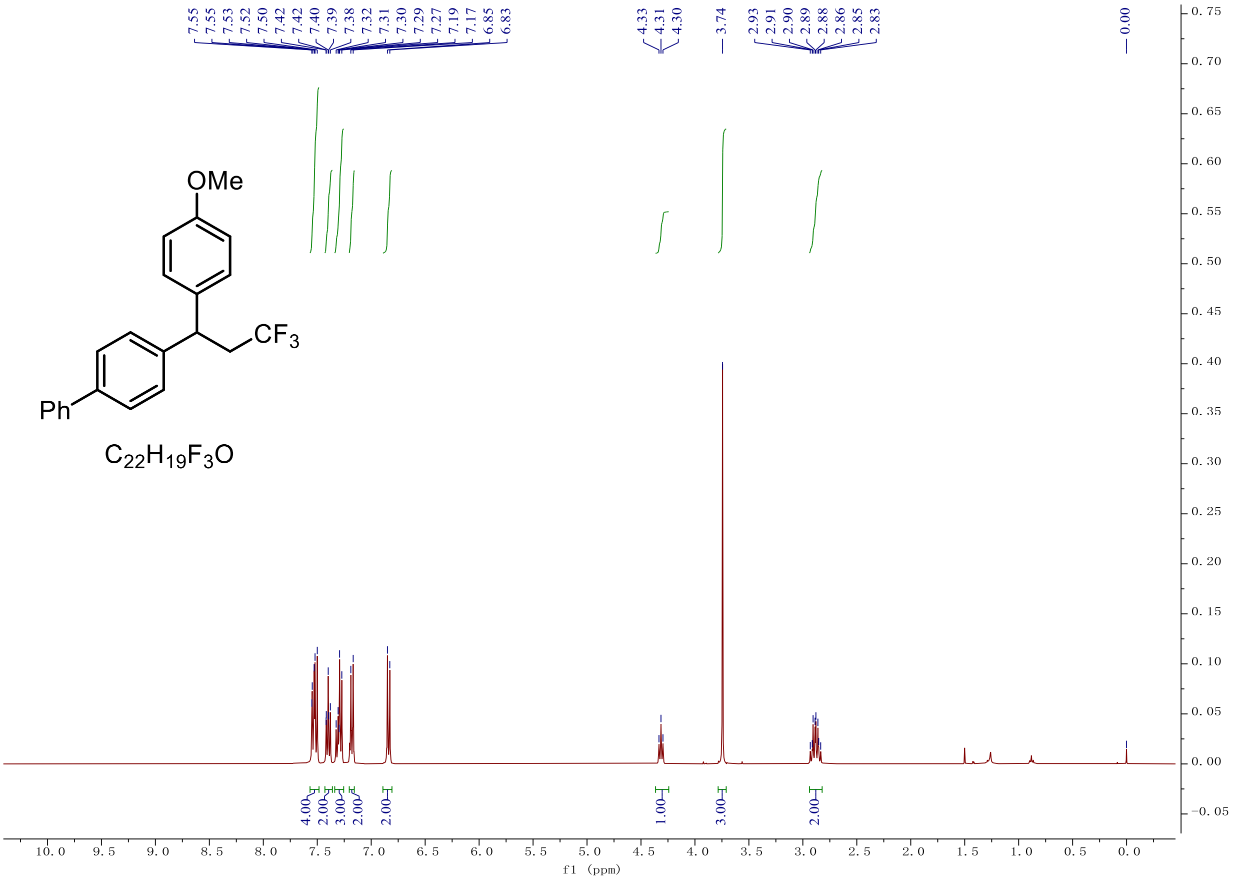

^{13}C NMR (101 MHz, $CDCl_3$) spectrum of 2e

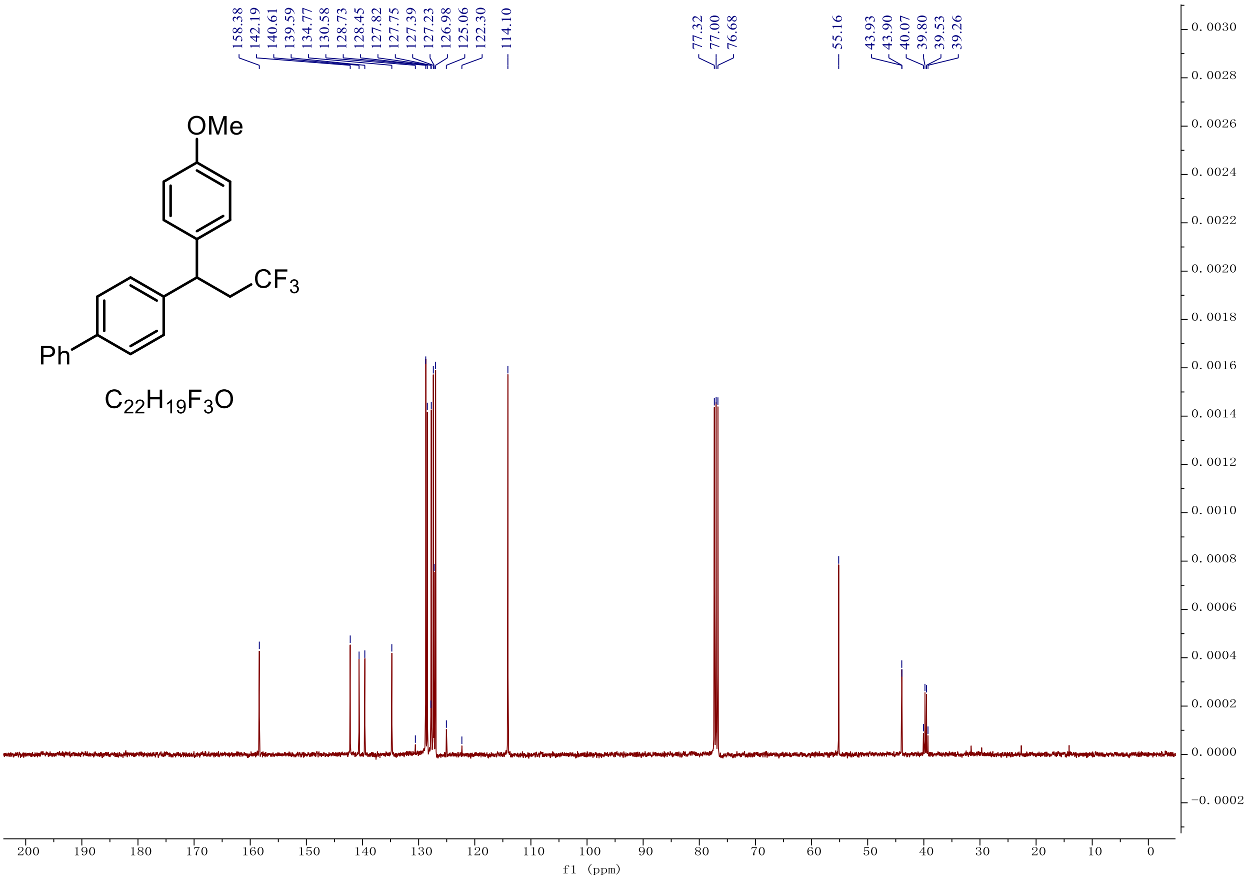

^{19}F NMR (376 MHz, CDCl3) spectrum of 2e

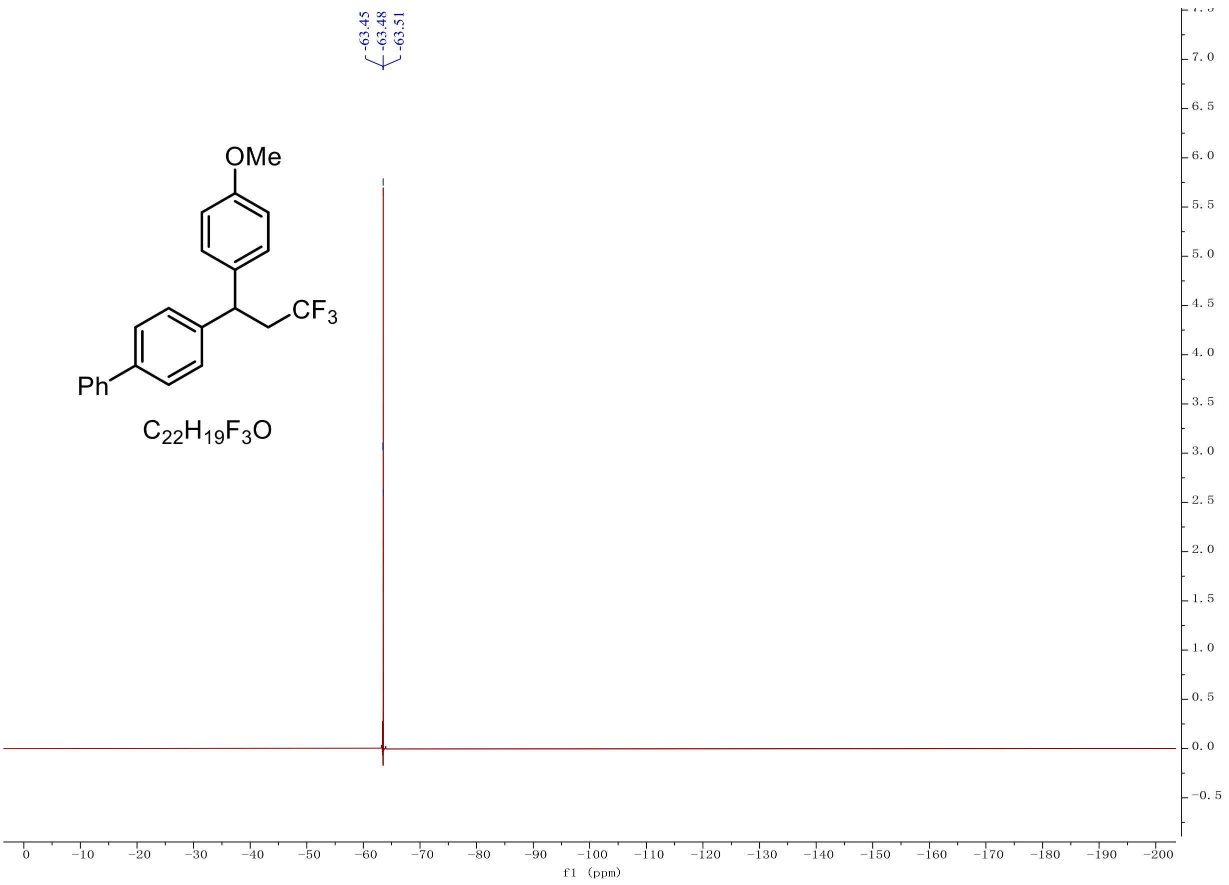

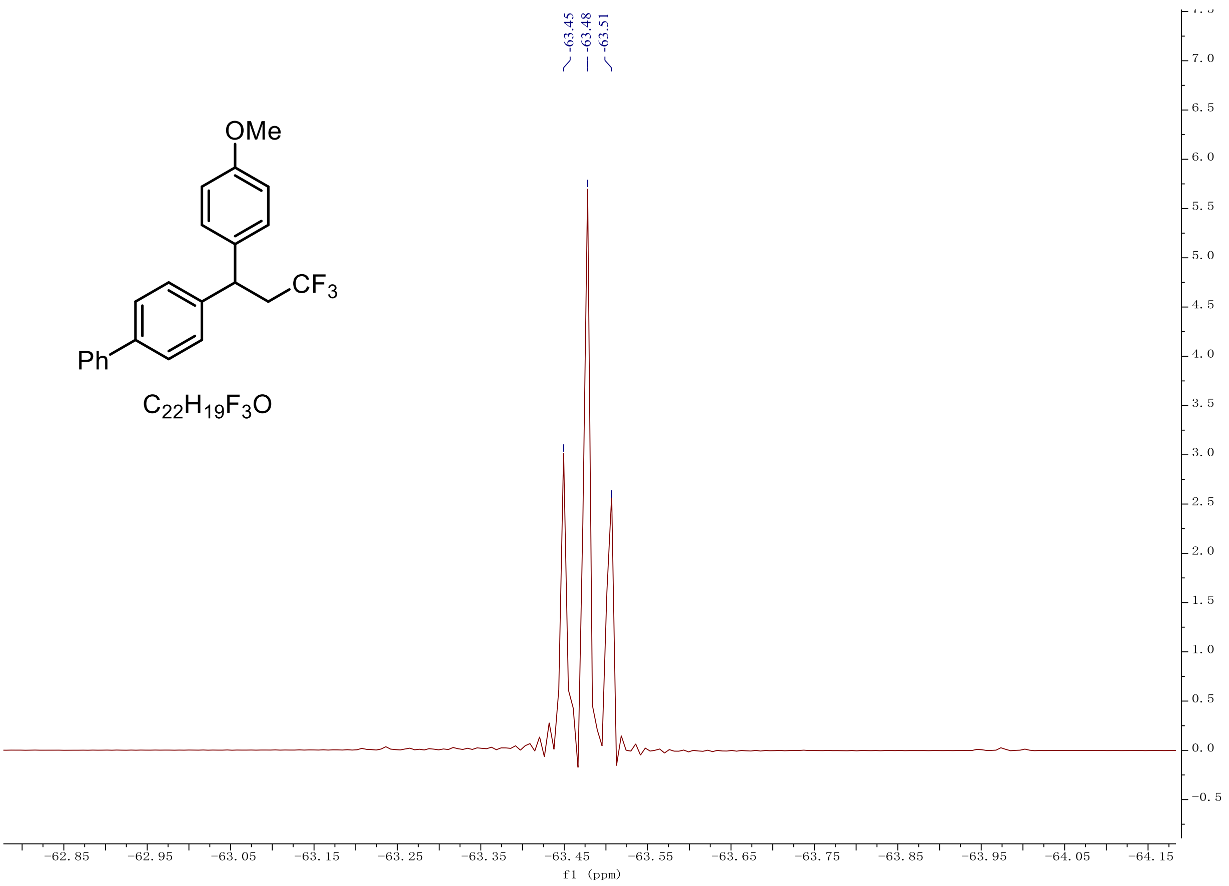

^{1}H NMR (400 MHz, $CDCl_3$) spectrum of 2f

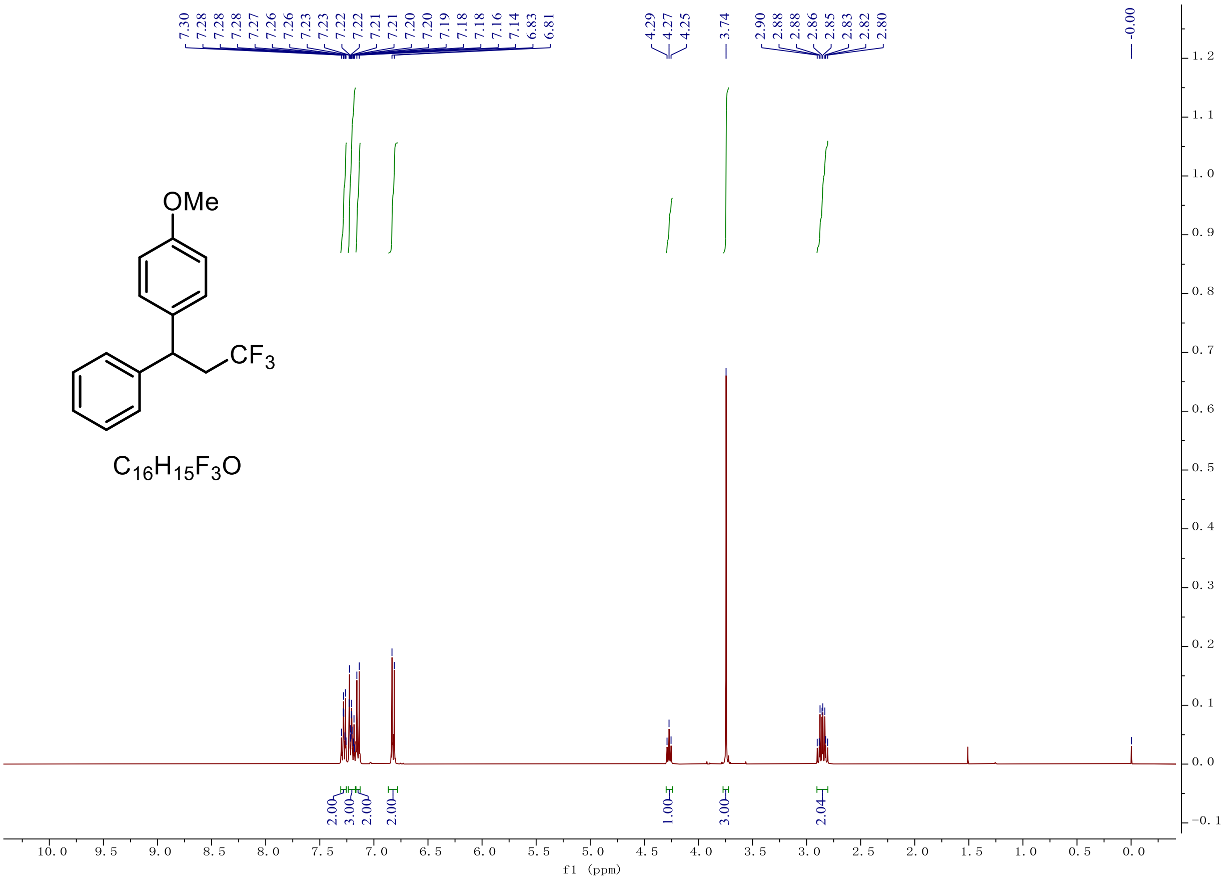

^{13}C NMR (101 MHz, $CDCl_3$) spectrum of 2f

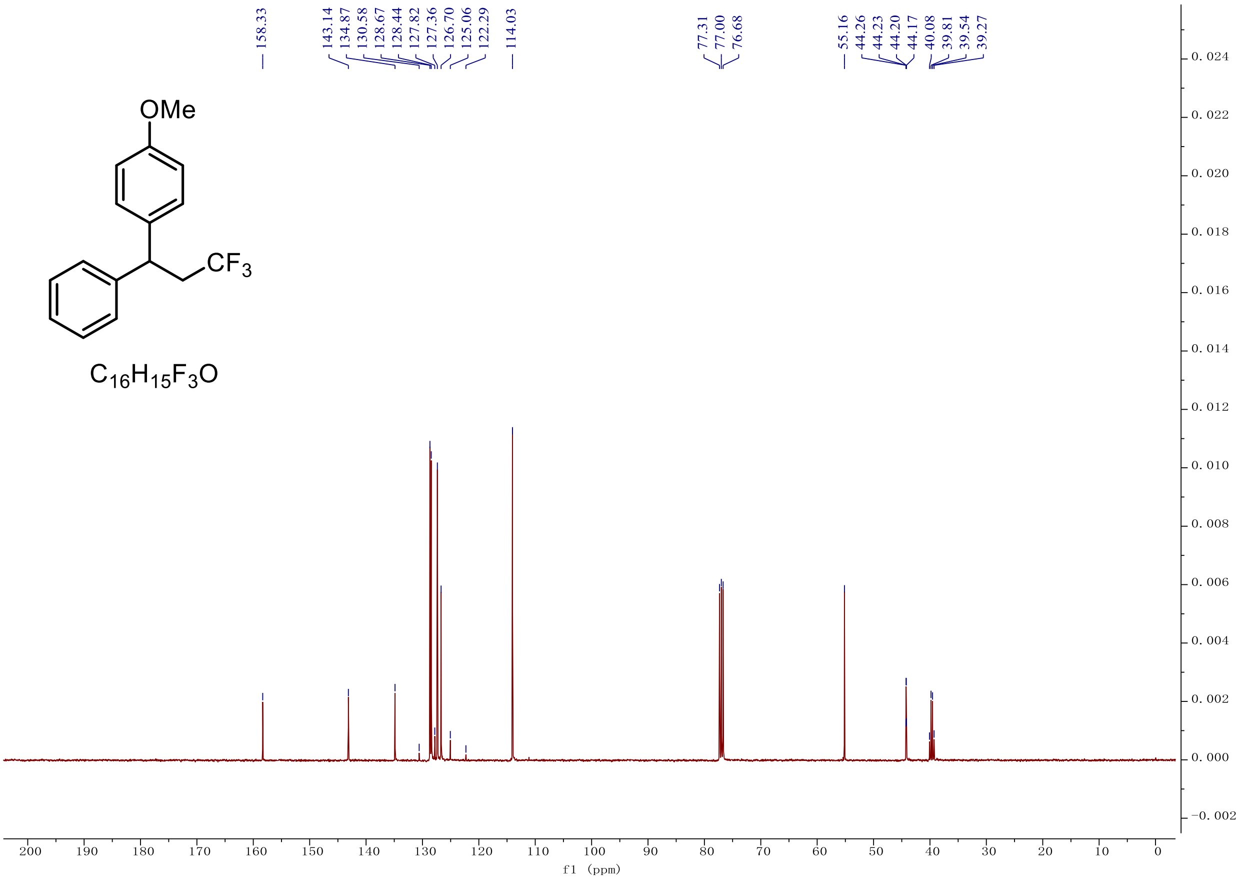

^{19}F NMR (376 MHz, $CDCl_3$) spectrum of 2f

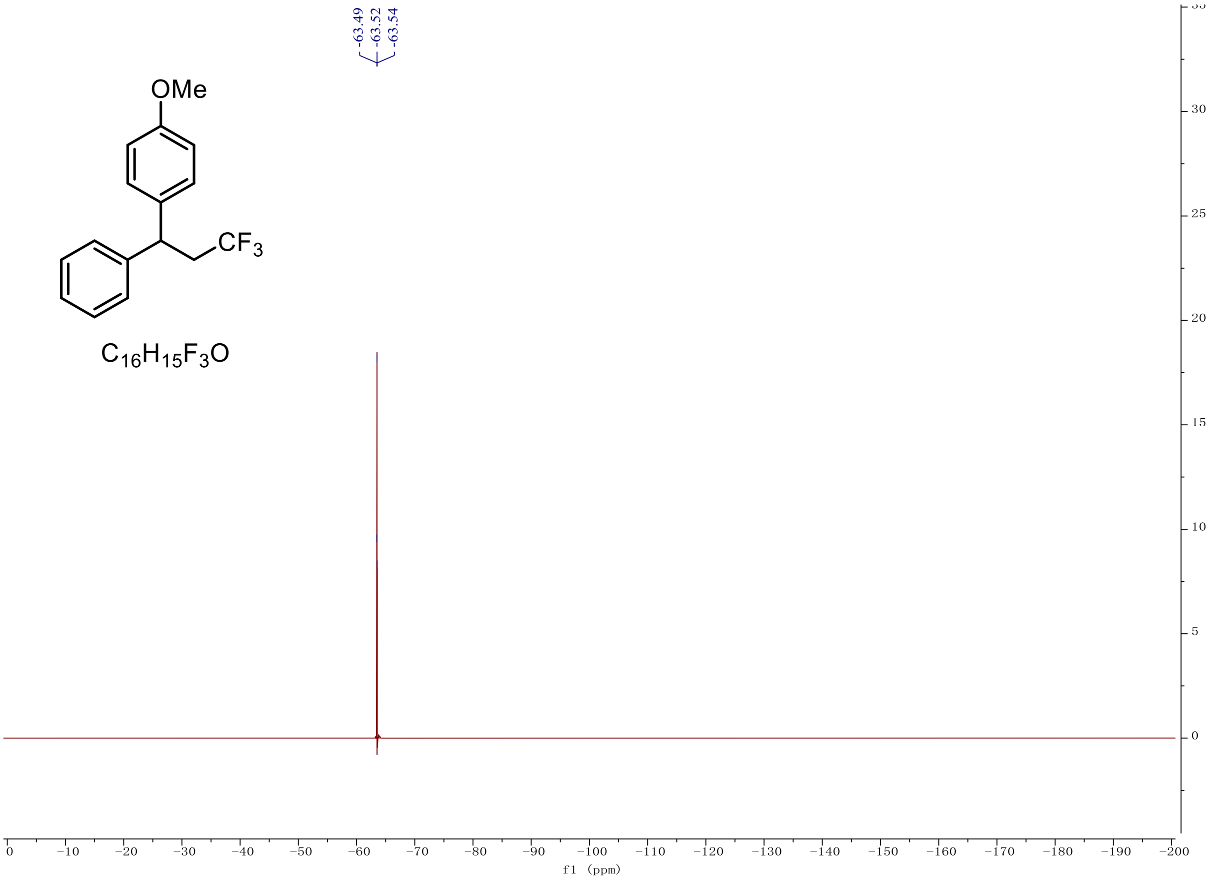

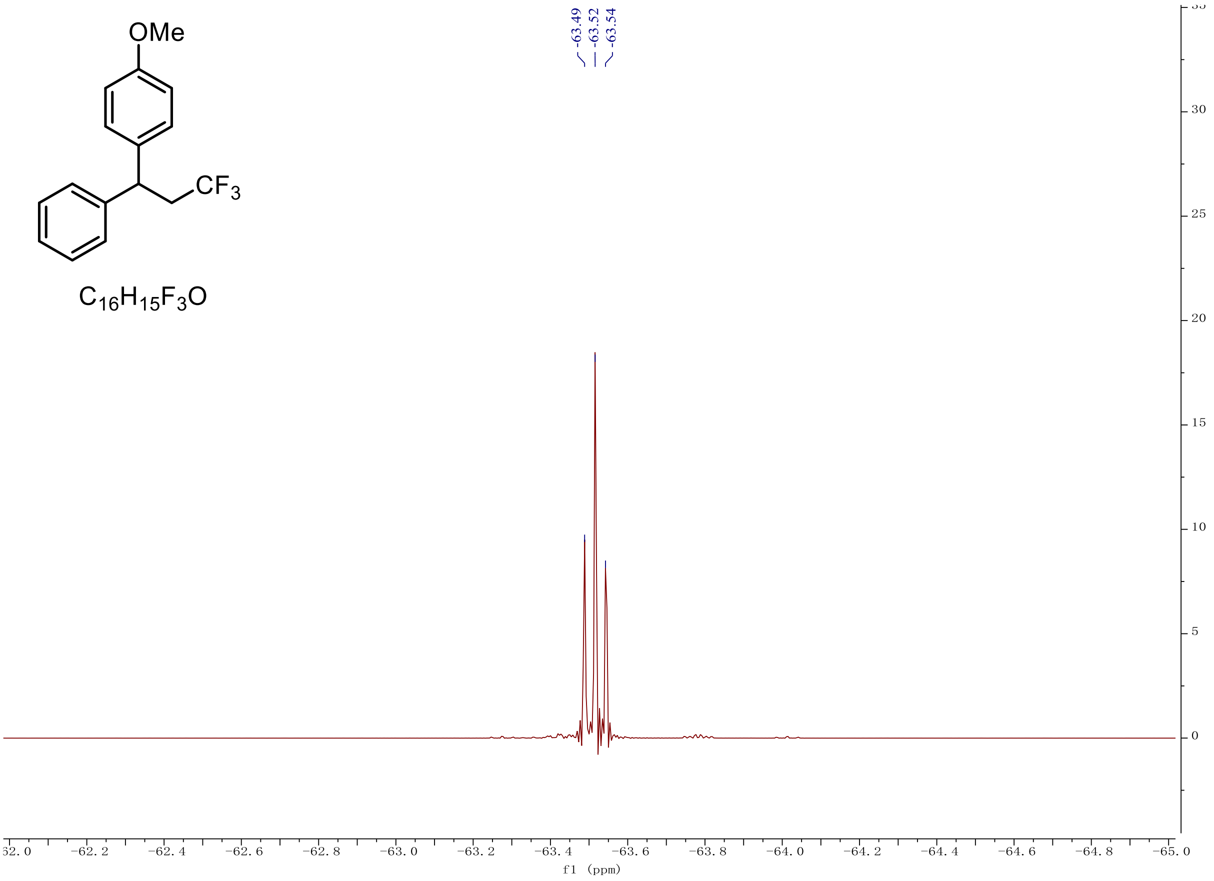

^{1}H NMR (400 MHz, CDCl3) spectrum of 2g

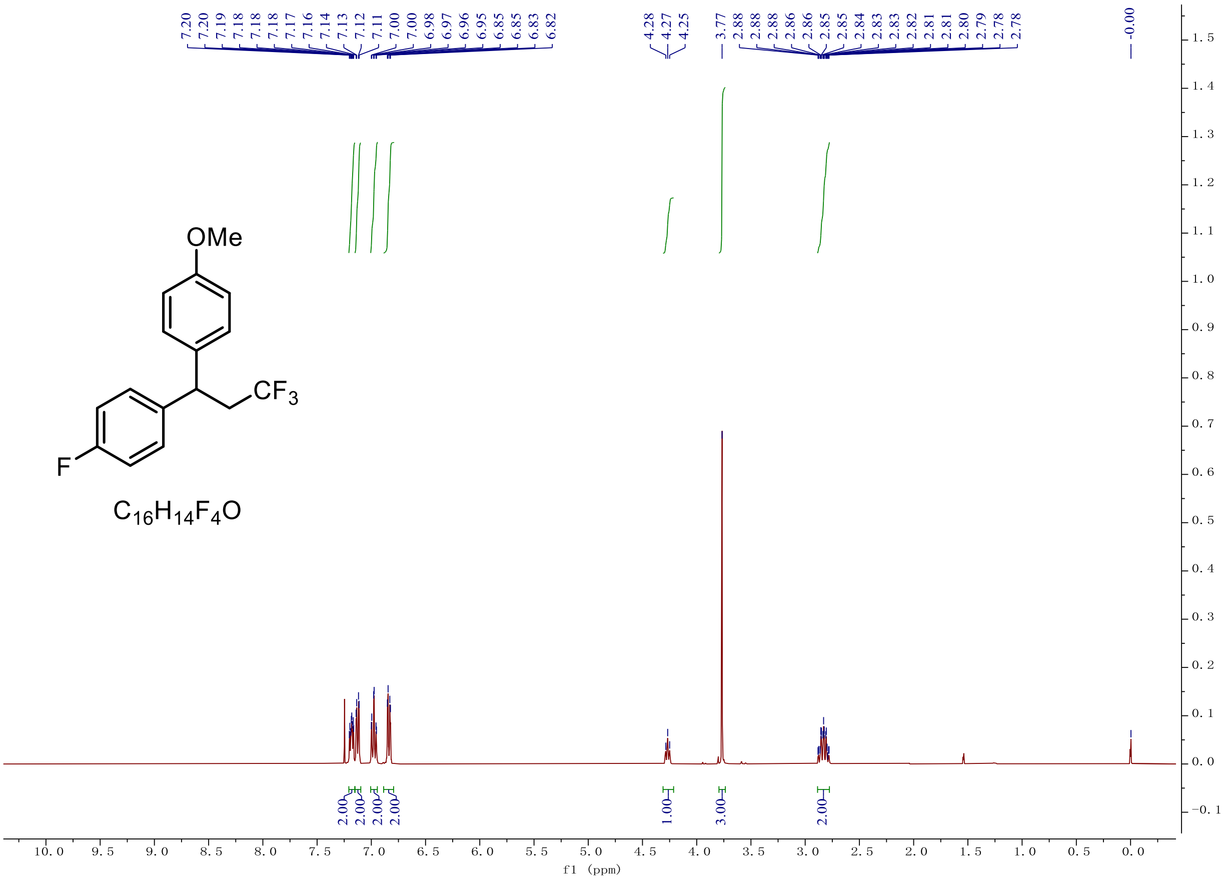

^{13}C NMR (101 MHz, CDCl3) spectrum of 2g

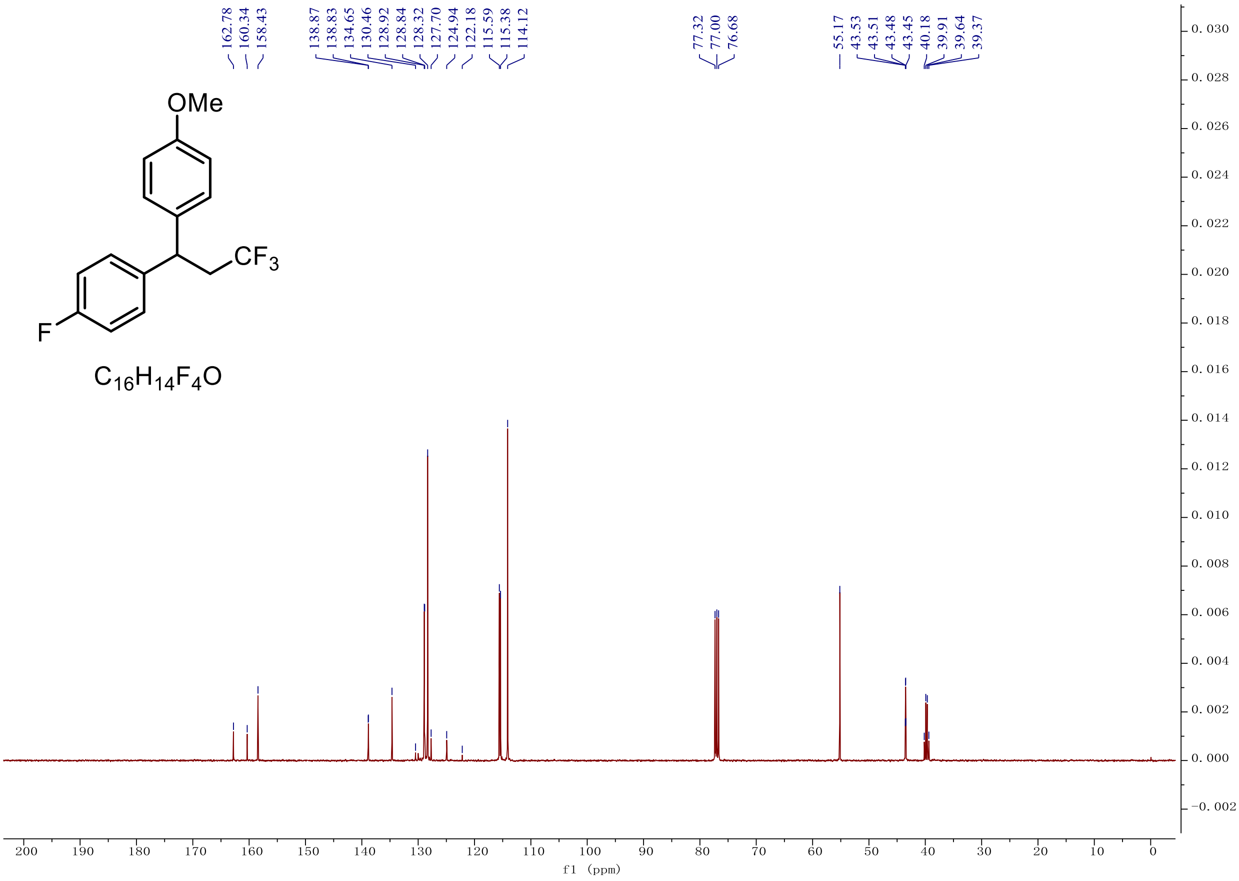

^{19}F NMR (376 MHz, $CDCl_3$) spectrum of 2g

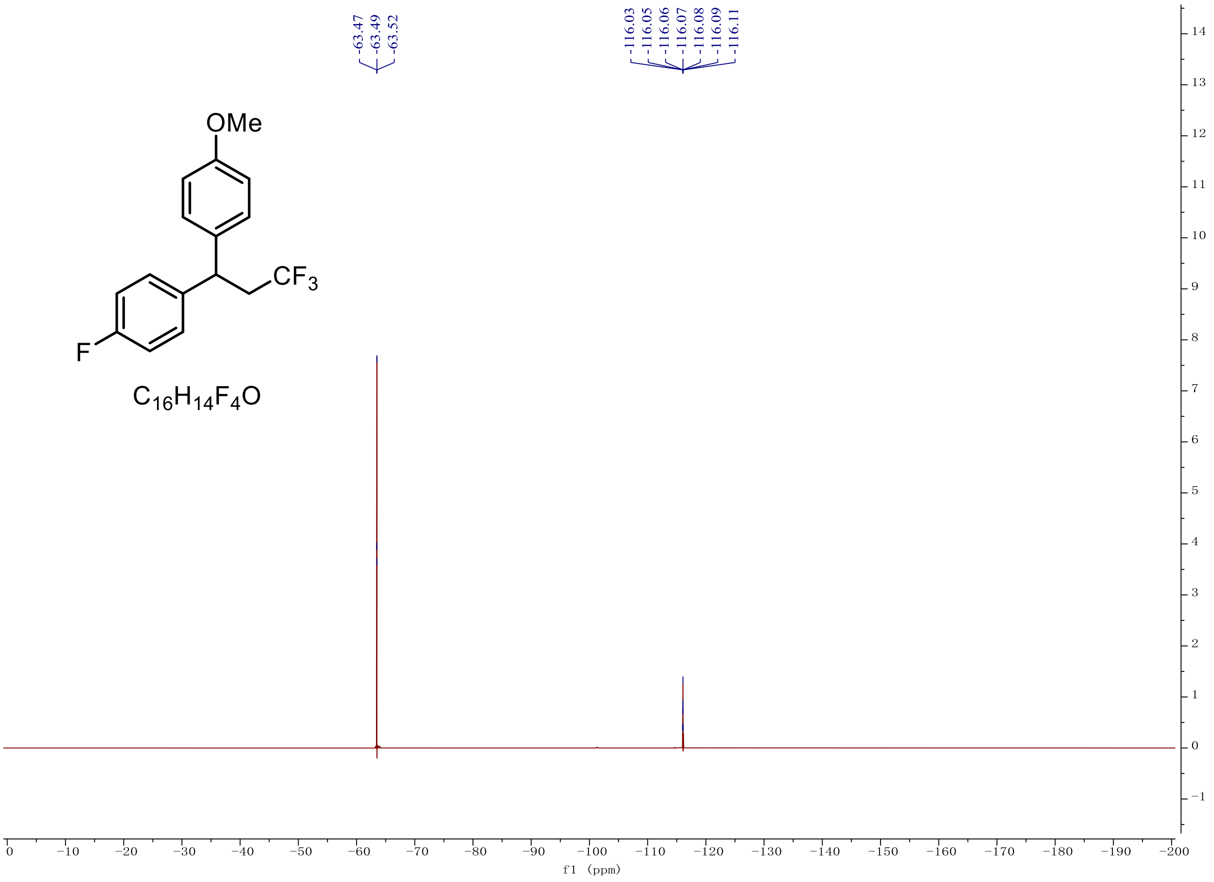

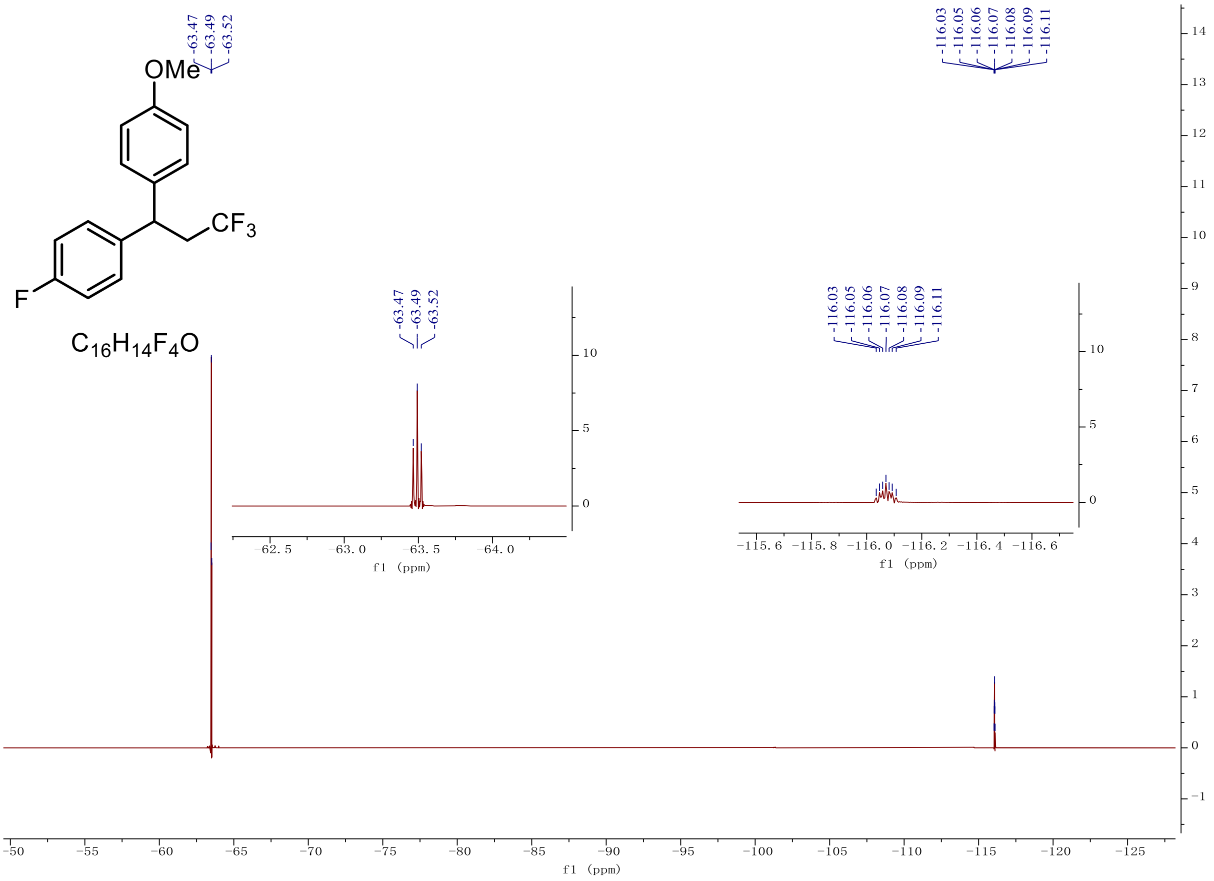

^{1}H NMR (400 MHz, $CDCl_3$) spectrum of 2h

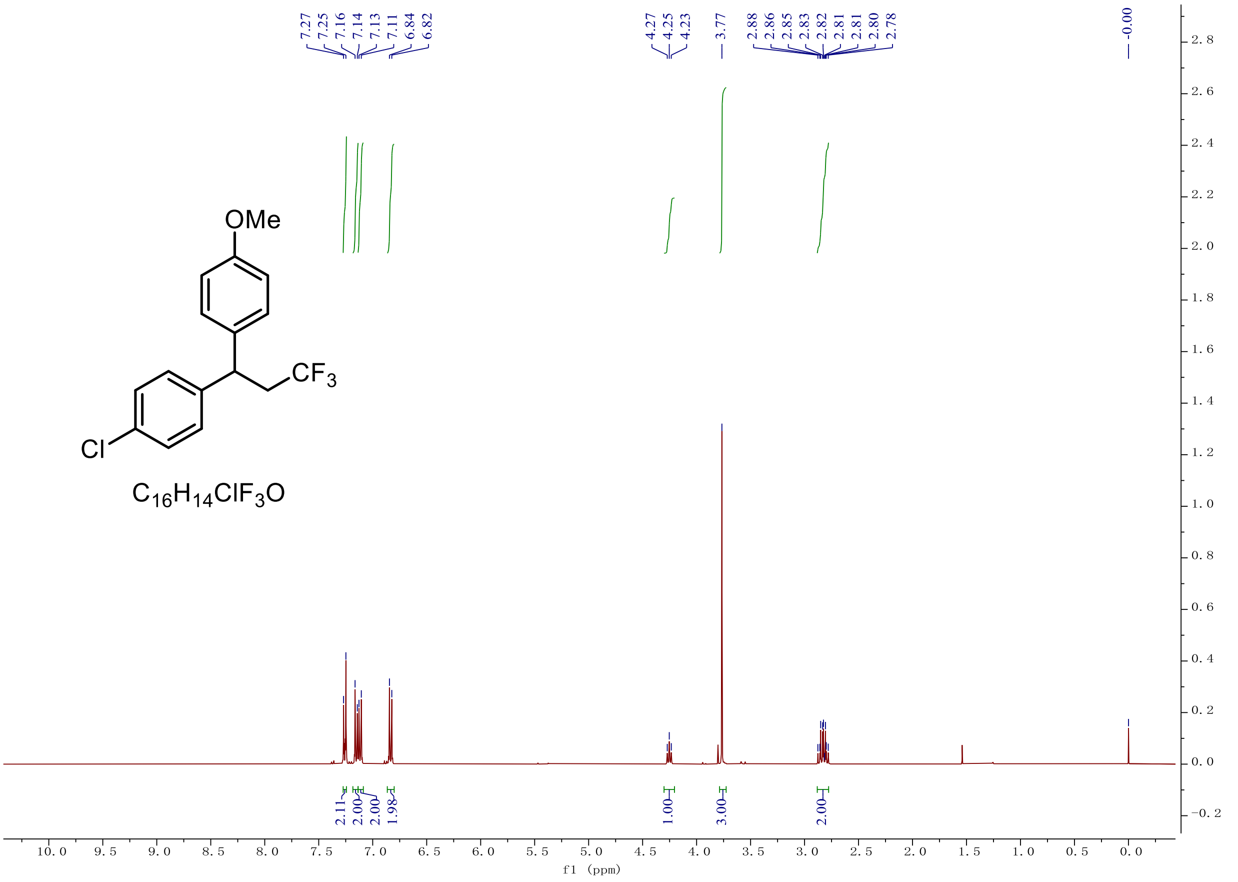

^{13}C NMR (101 MHz, $CDCl_3$) spectrum of 2h

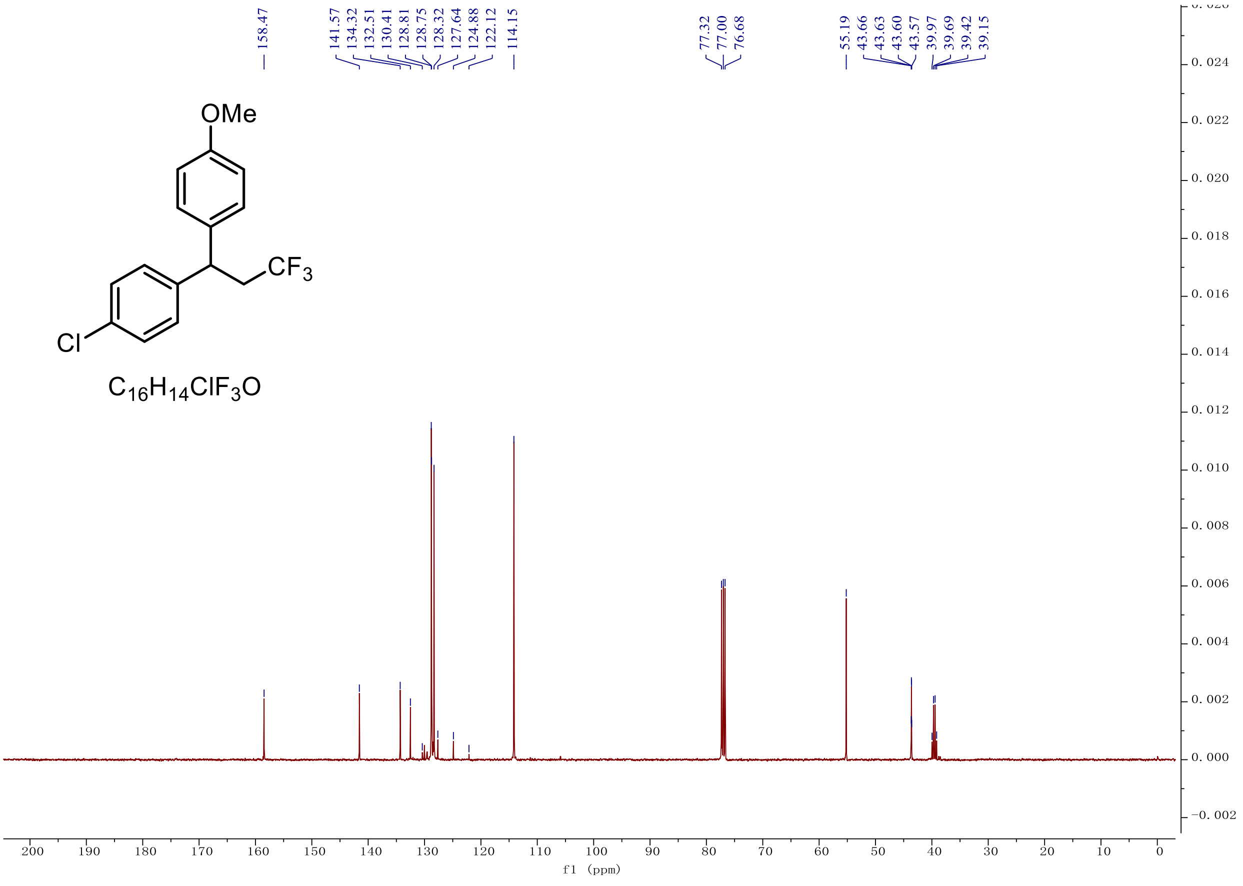

^{19}F NMR (376 MHz, $CDCl_3$) spectrum of 2h

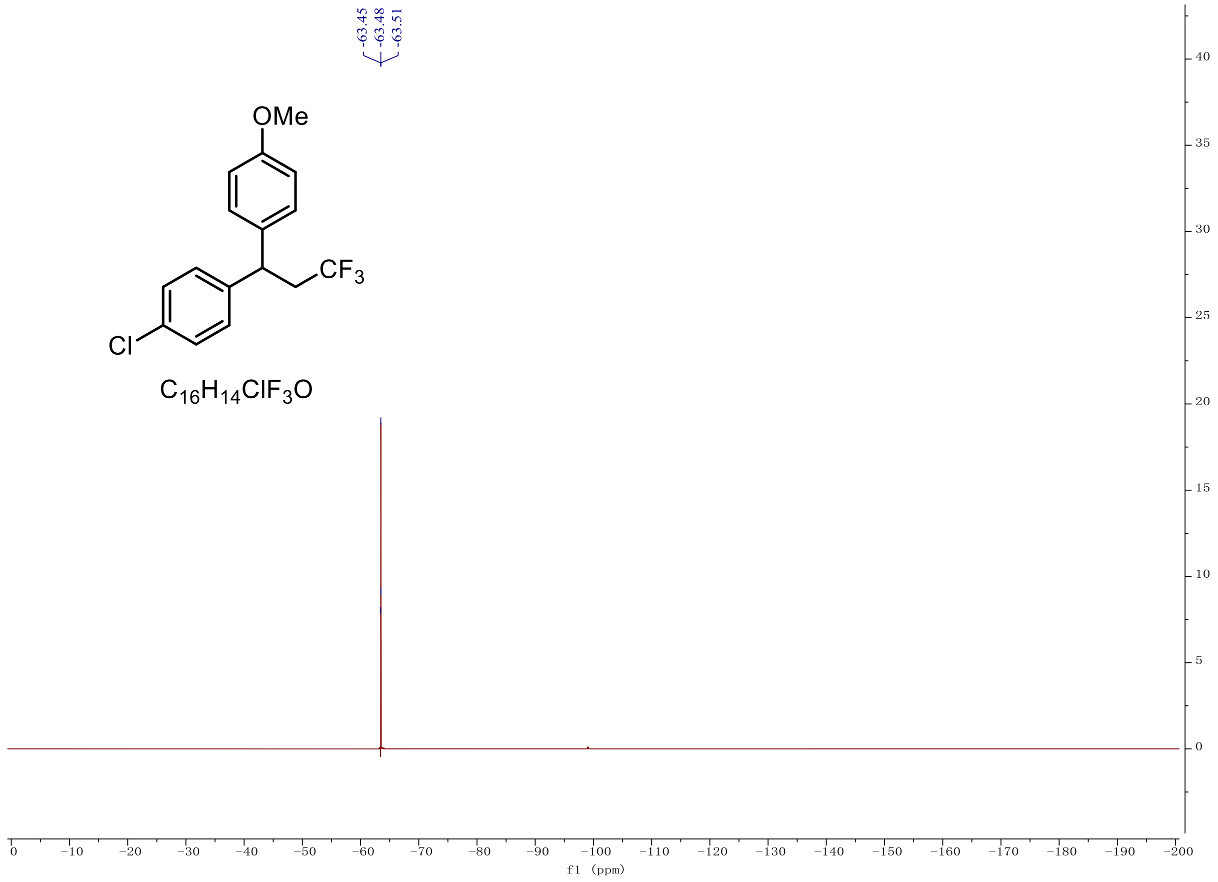

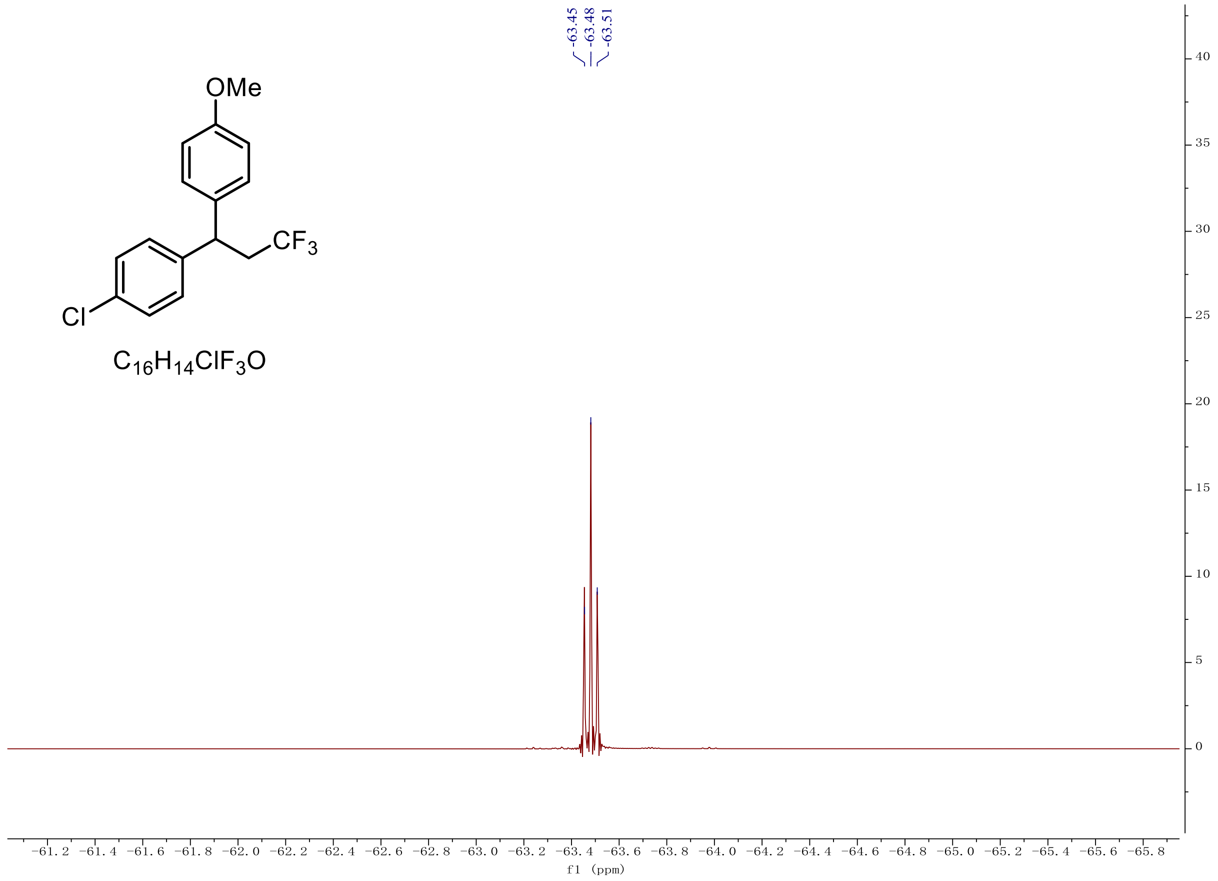

^{1}H NMR (400 MHz, $CDCl_3$) spectrum of 2i

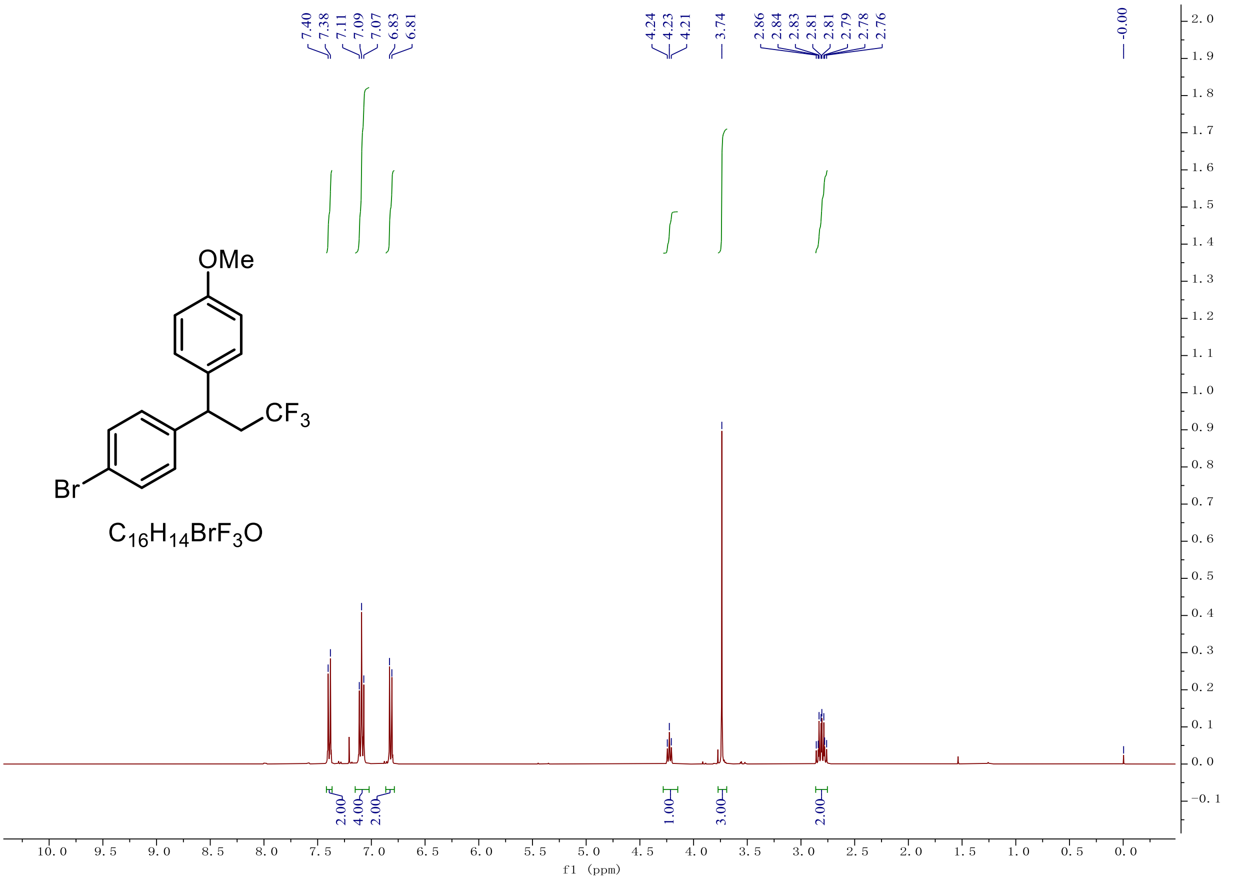

^{13}C NMR (101 MHz, $CDCl_3$) spectrum of 2i

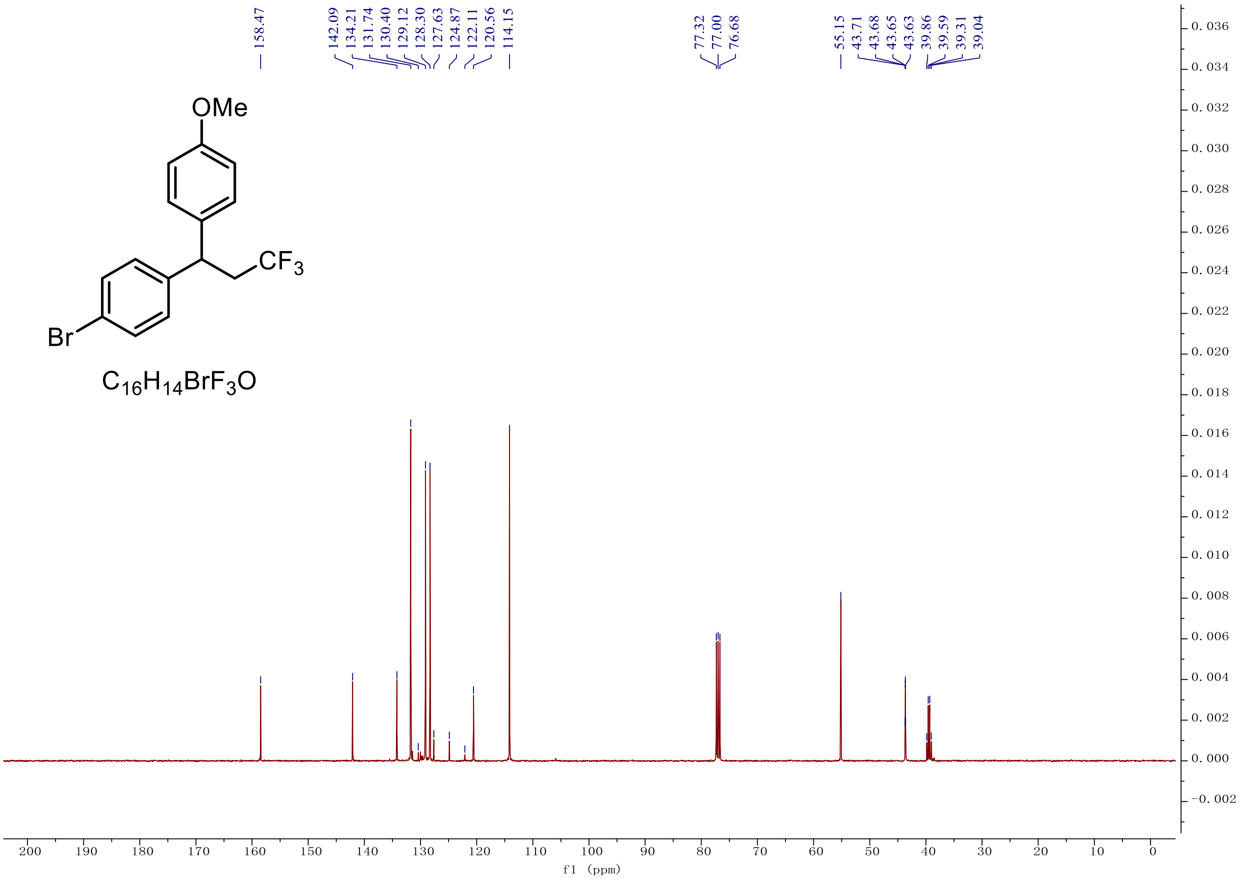

^{19}F NMR (376 MHz, CDCl₃) spectrum of 2i

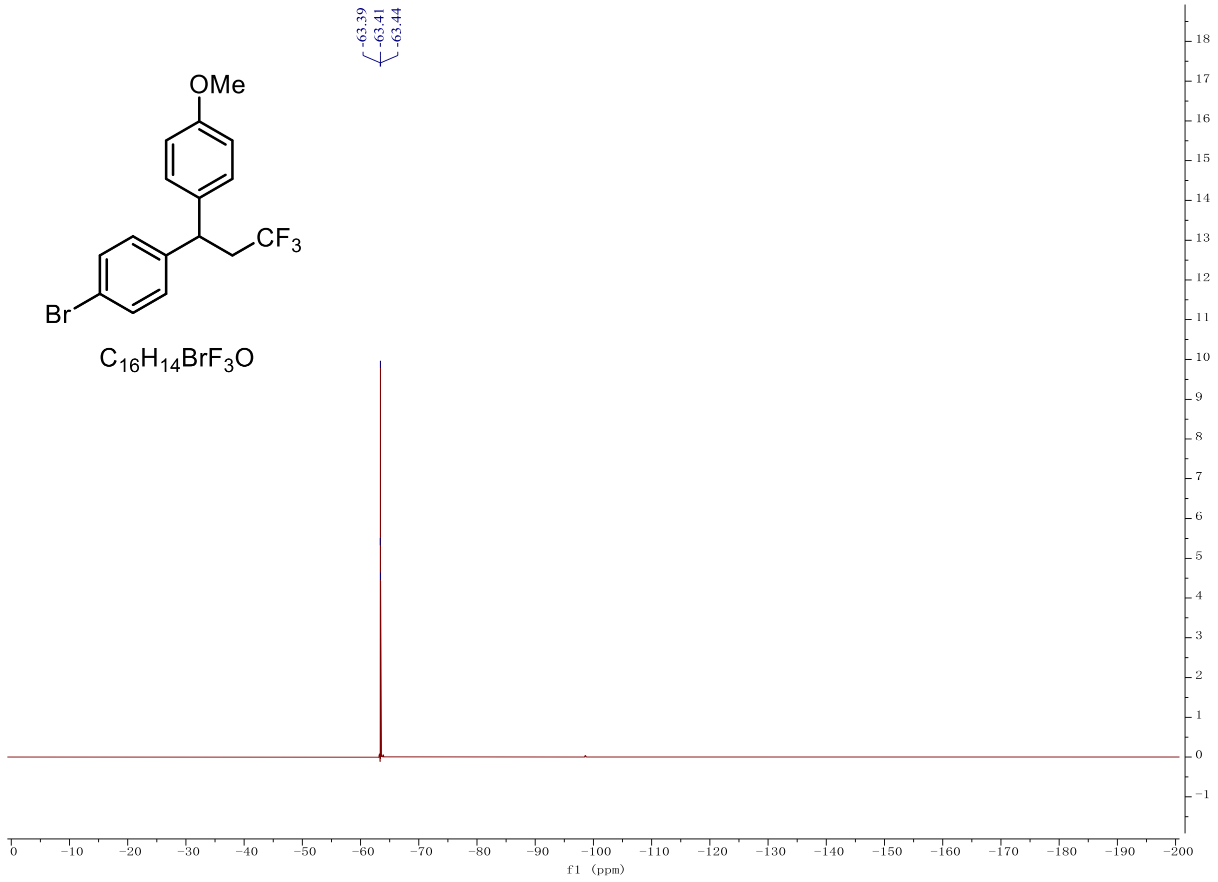

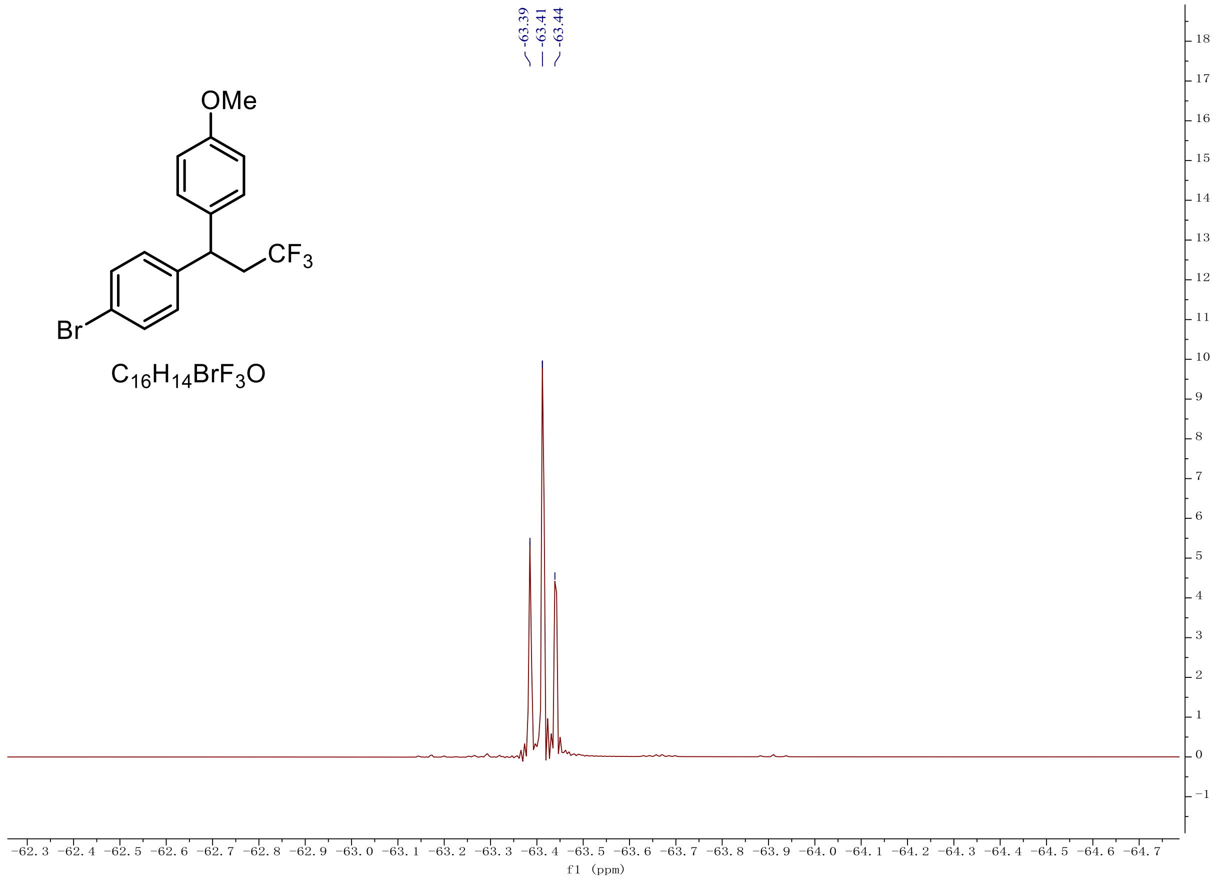

^{1}H NMR (400 MHz, CDCl3) spectrum of 2j

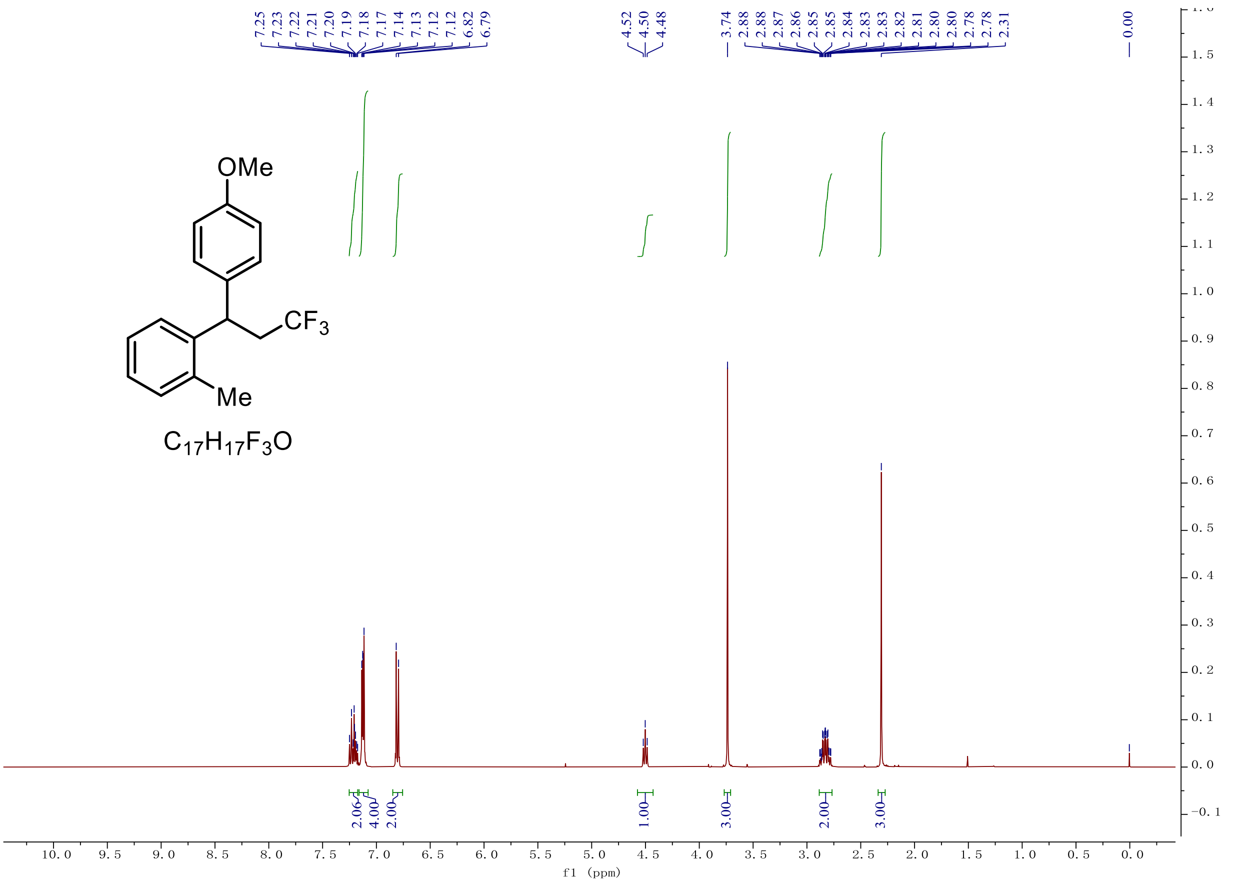

^{13}C NMR (101 MHz, CDCl3) spectrum of 2j

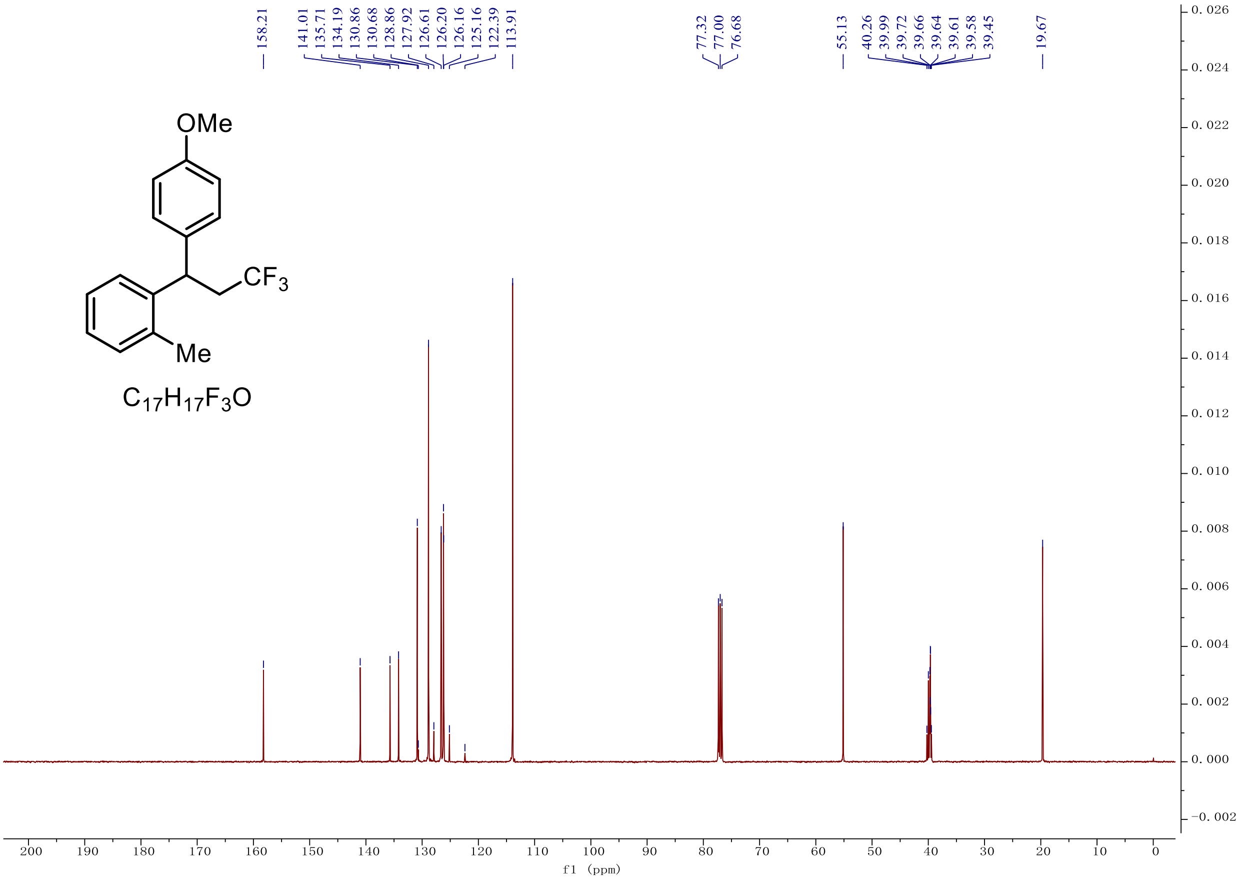

^{19}F NMR (376 MHz, $CDCl_3$) spectrum of 2j

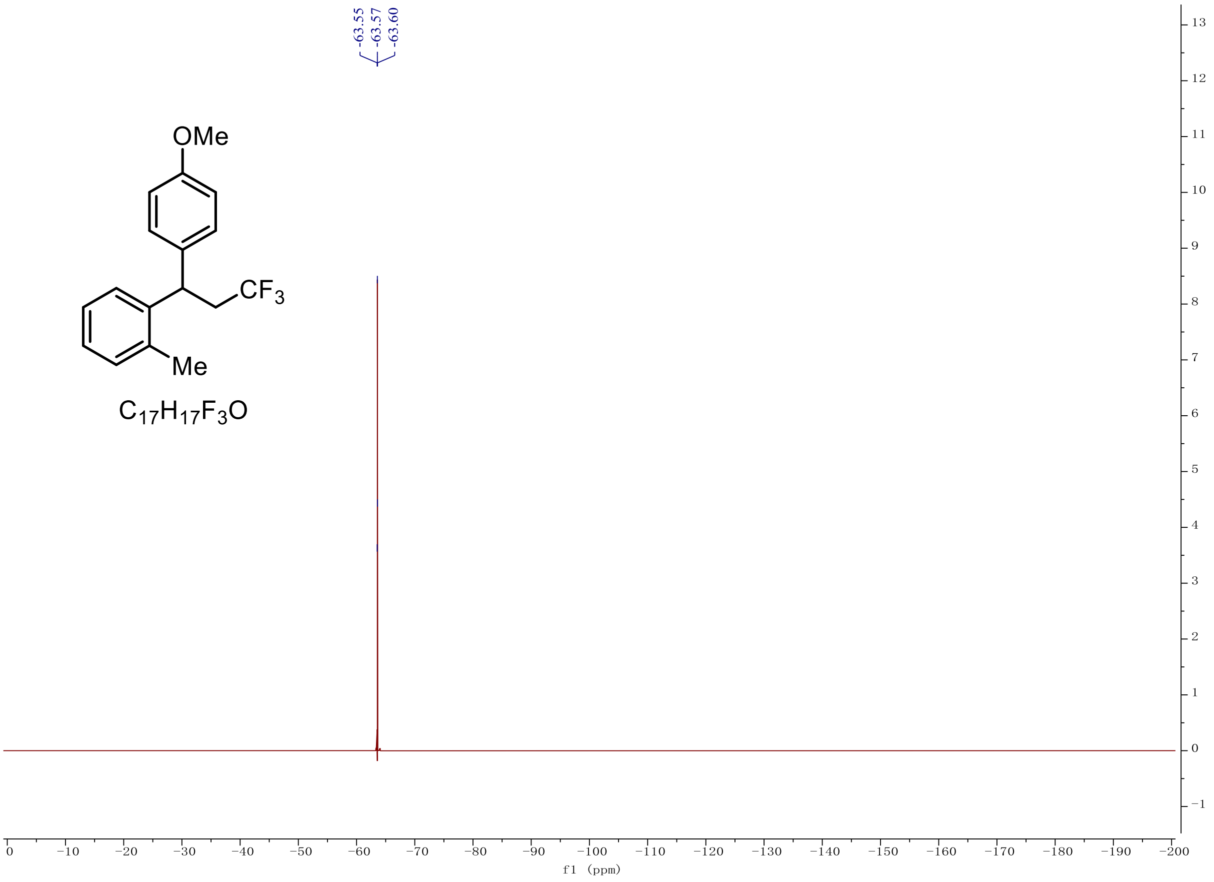

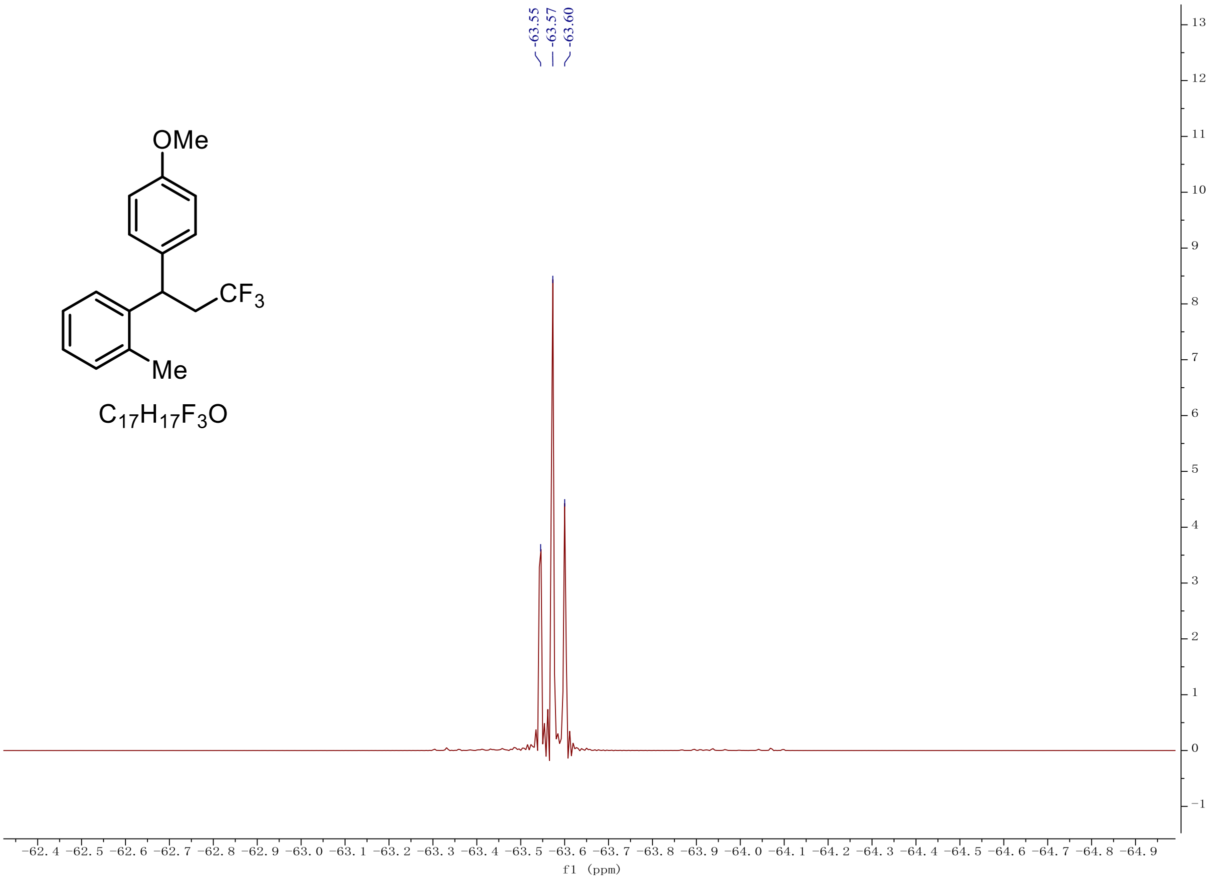

^{1}H NMR (400 MHz, $CDCl_3$) spectrum of 2k

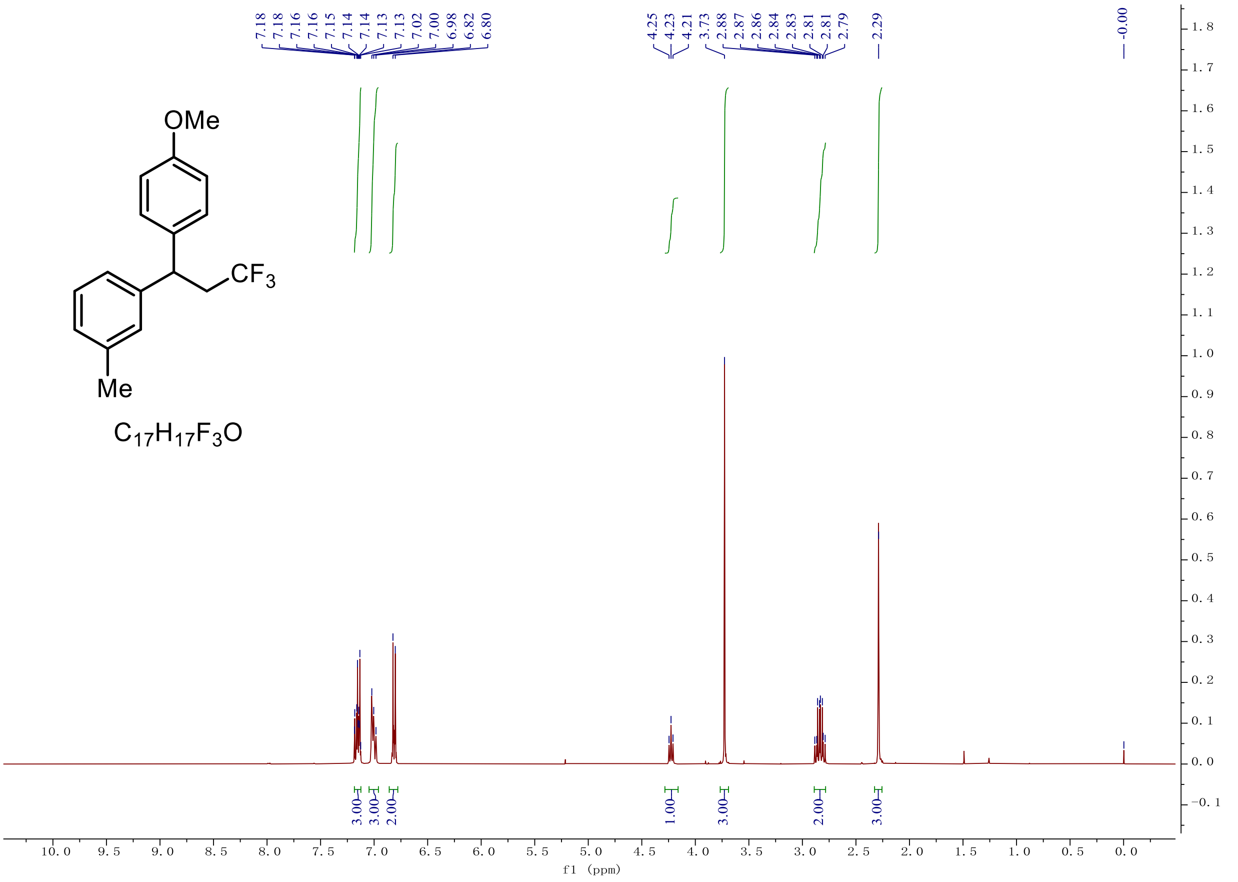

^{13}C NMR (101 MHz, $CDCl_3$) spectrum of 2k

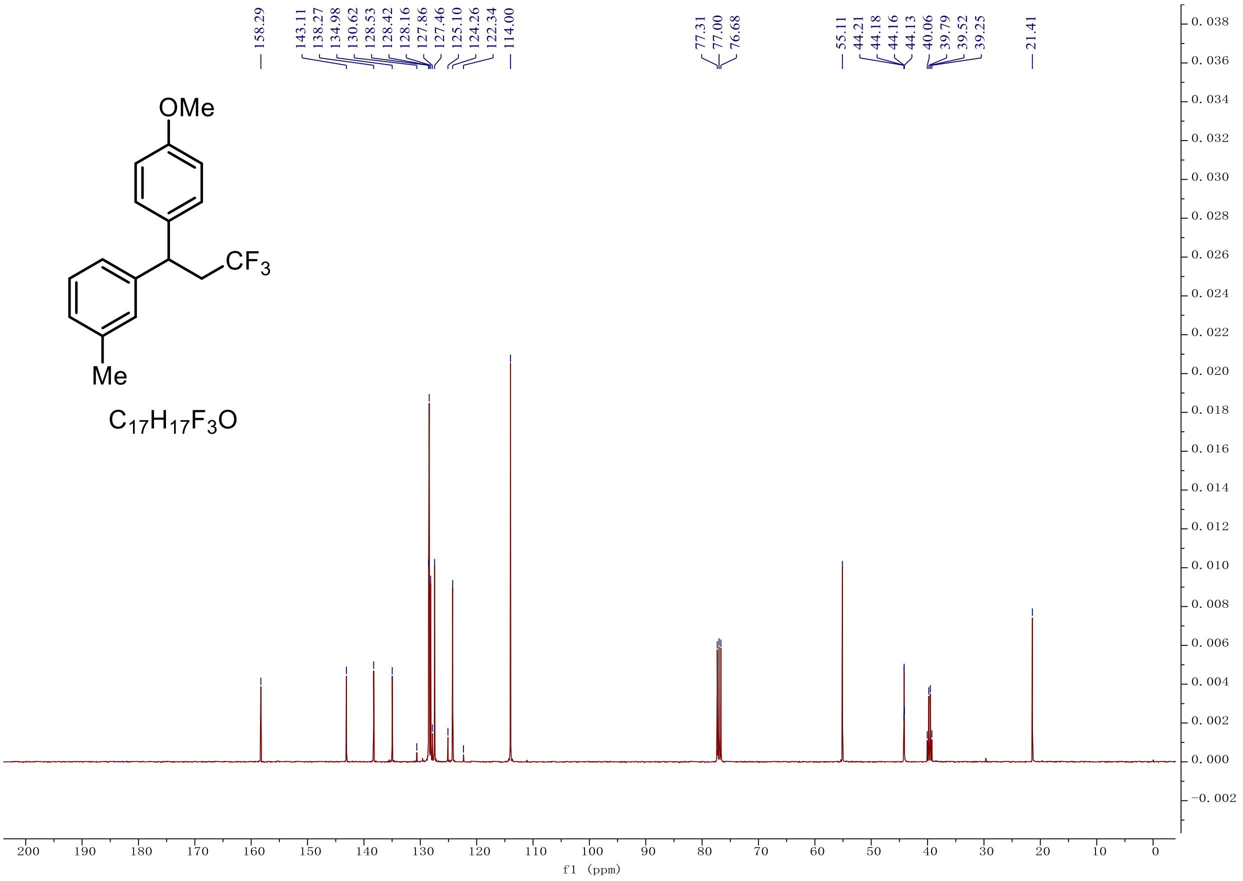

^{19}F NMR (376 MHz, $CDCl_3$) spectrum of 2k

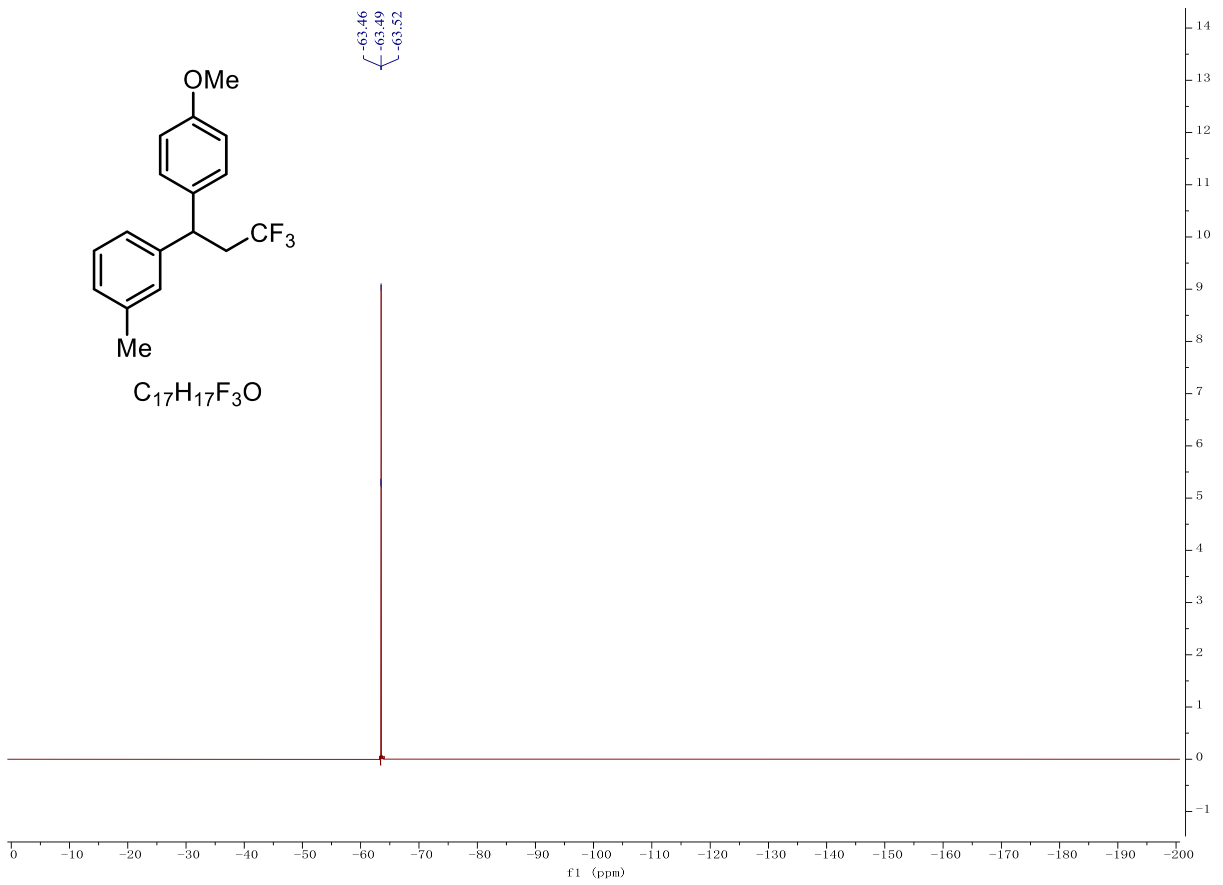

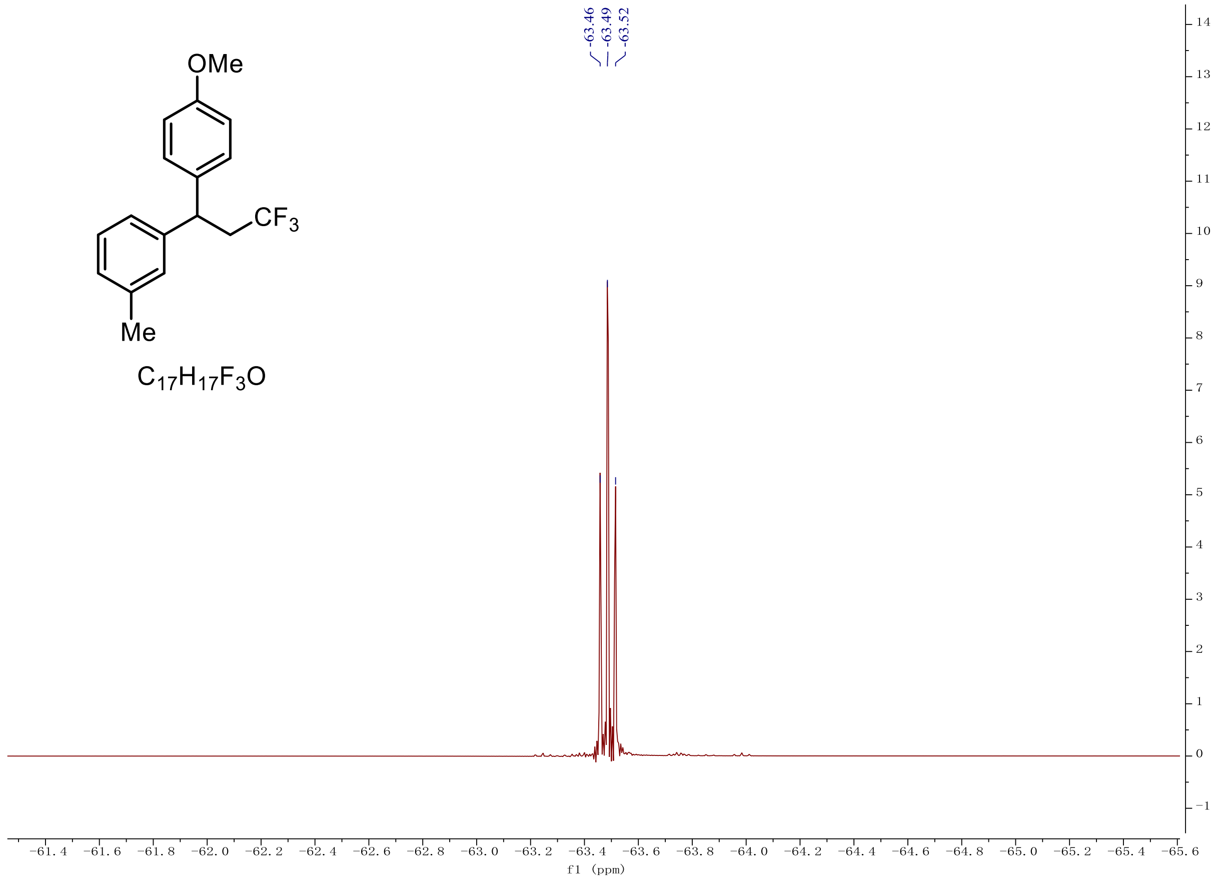

^{1}H NMR (400 MHz, CDCl3) spectrum of 2l

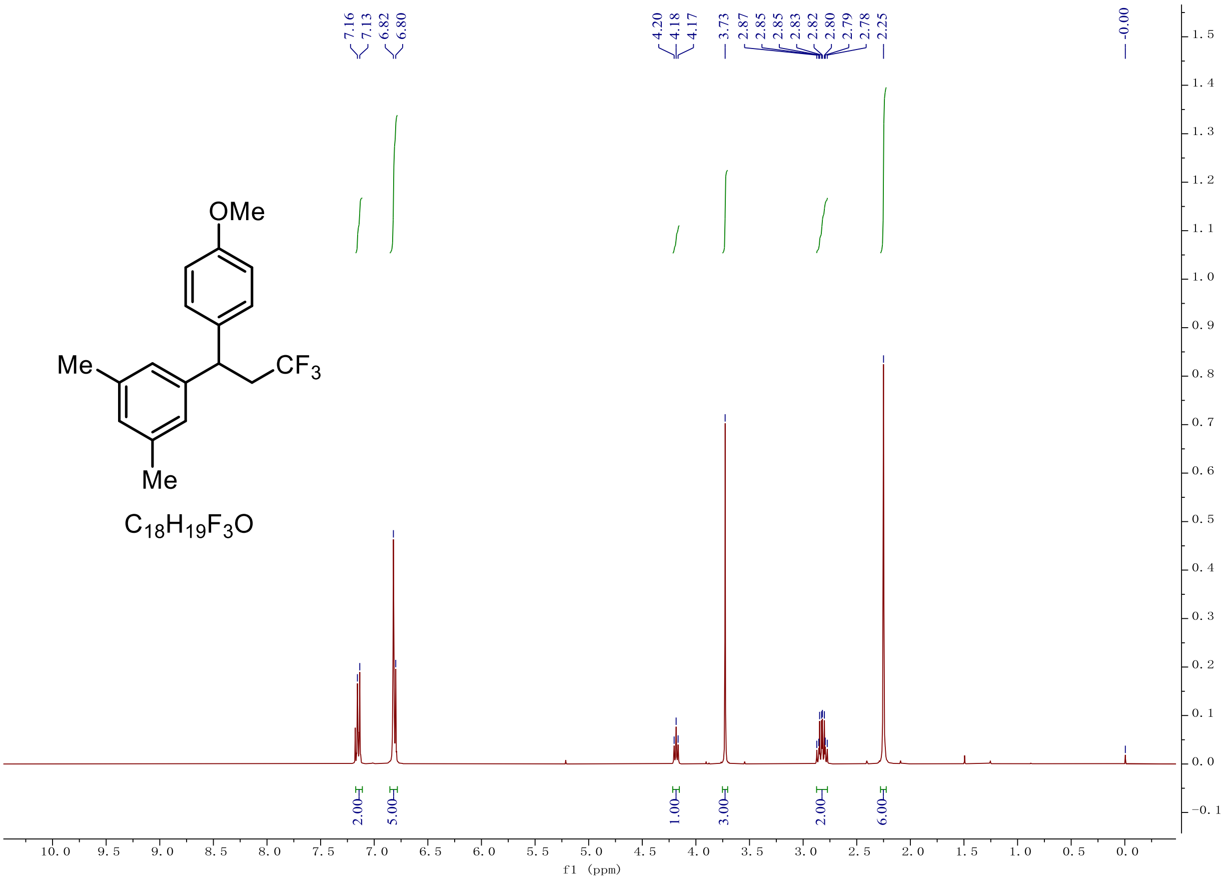

^{13}C NMR (101 MHz, CDCl3) spectrum of 2l

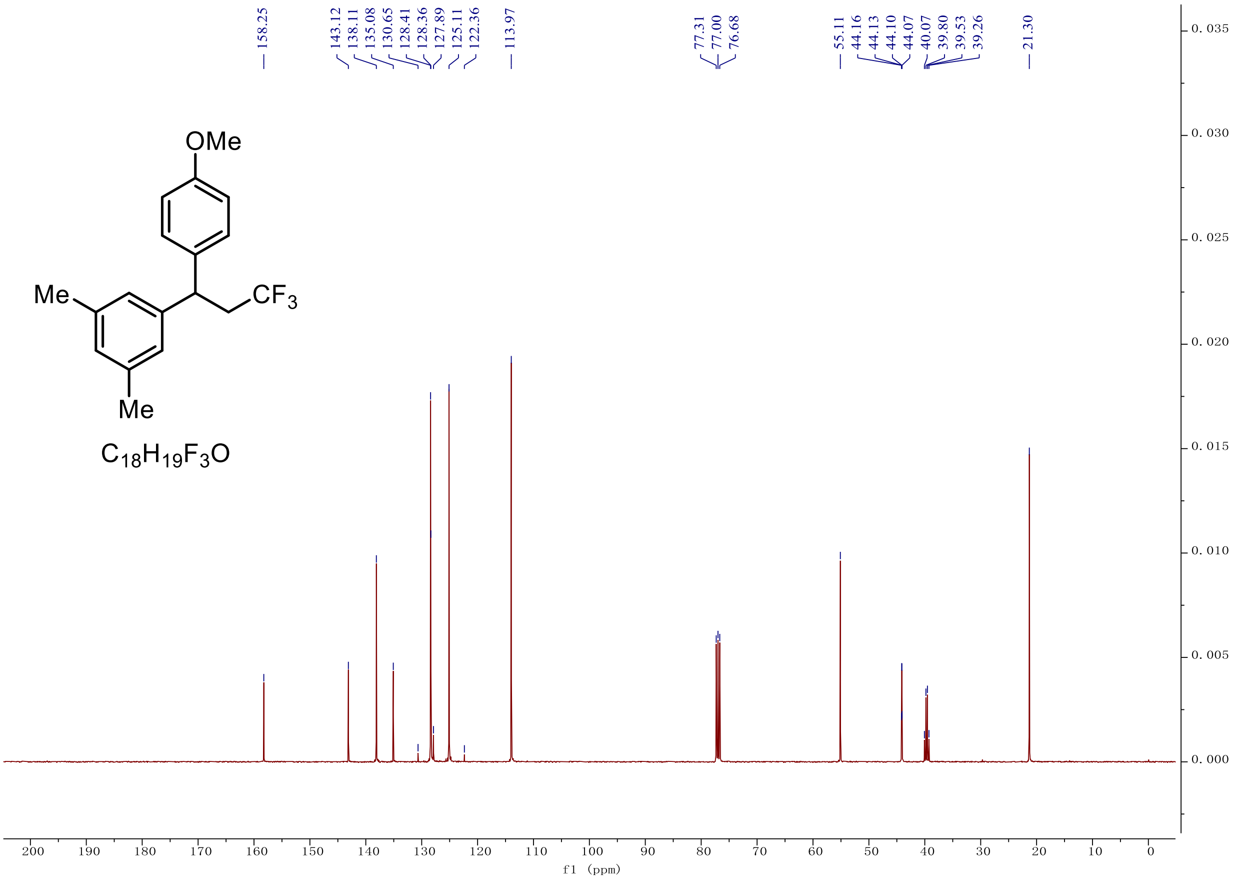

^{19}F NMR (376 MHz, CDCl3) spectrum of 2l

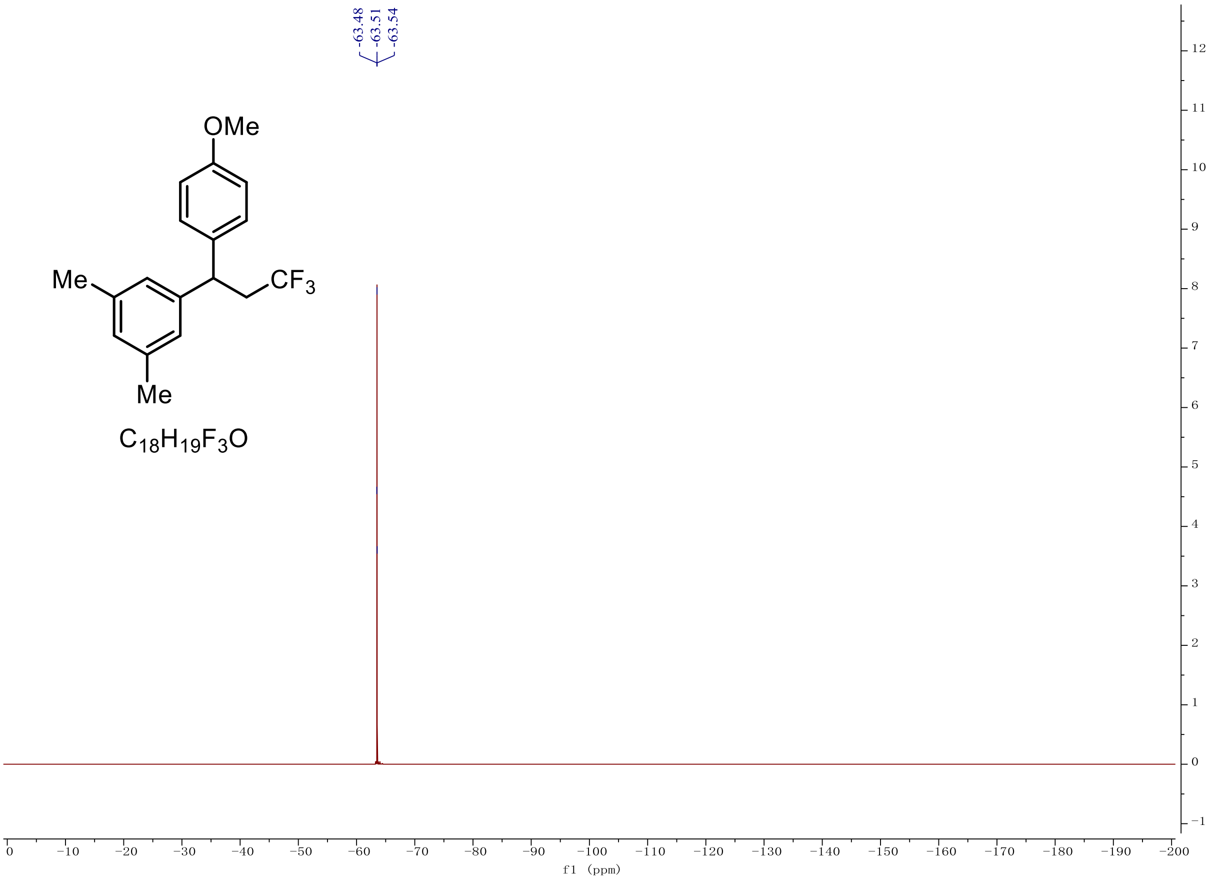

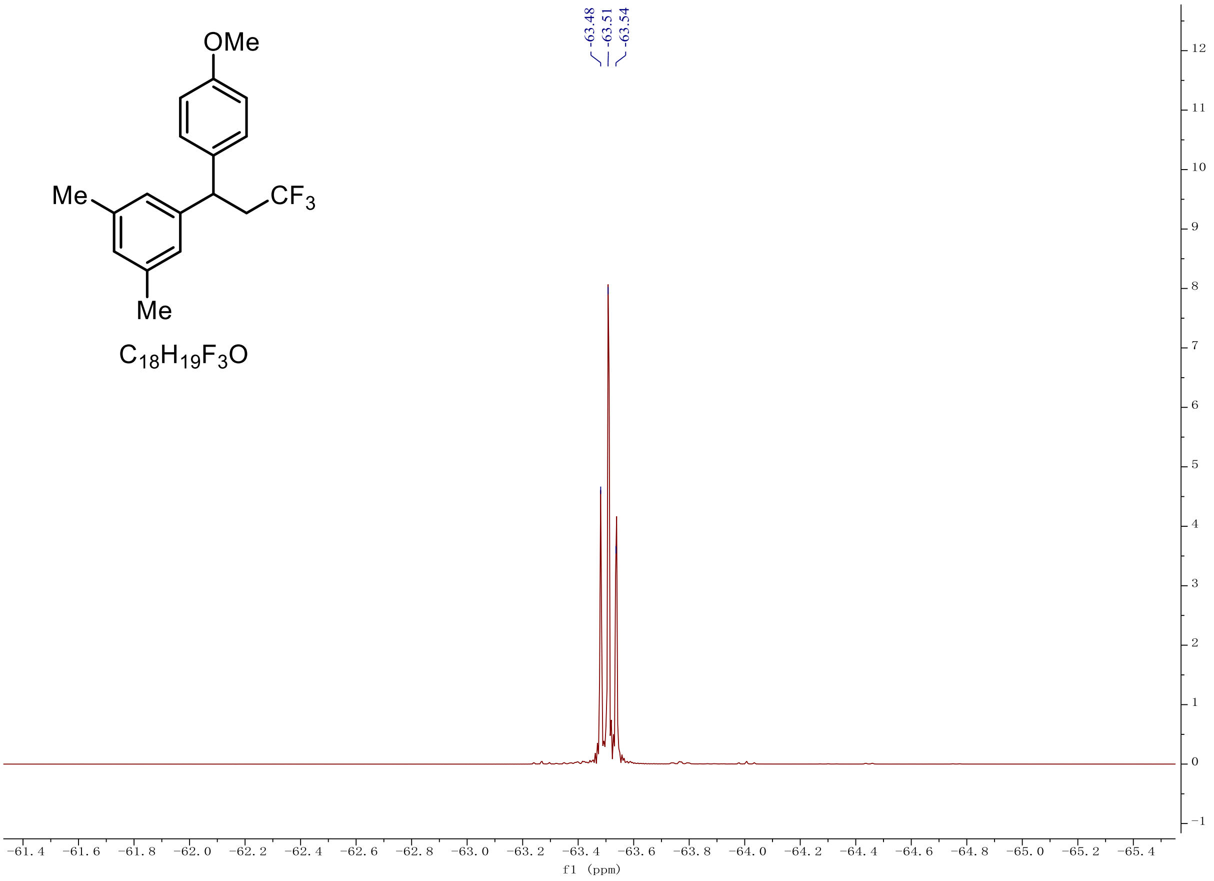

^{1}H NMR (400 MHz, CDCl3) spectrum of 2m

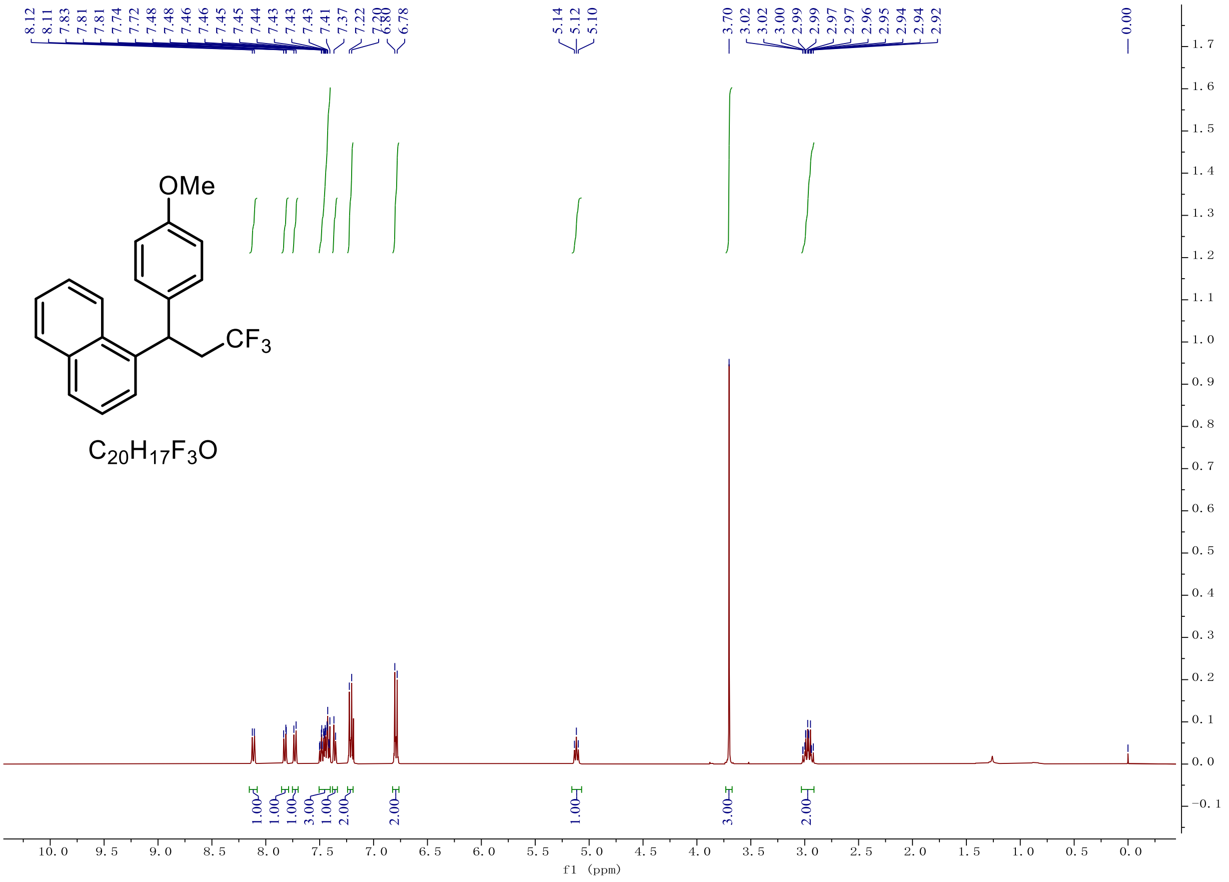

^{13}C NMR (101 MHz, CDCl3) spectrum of 2m

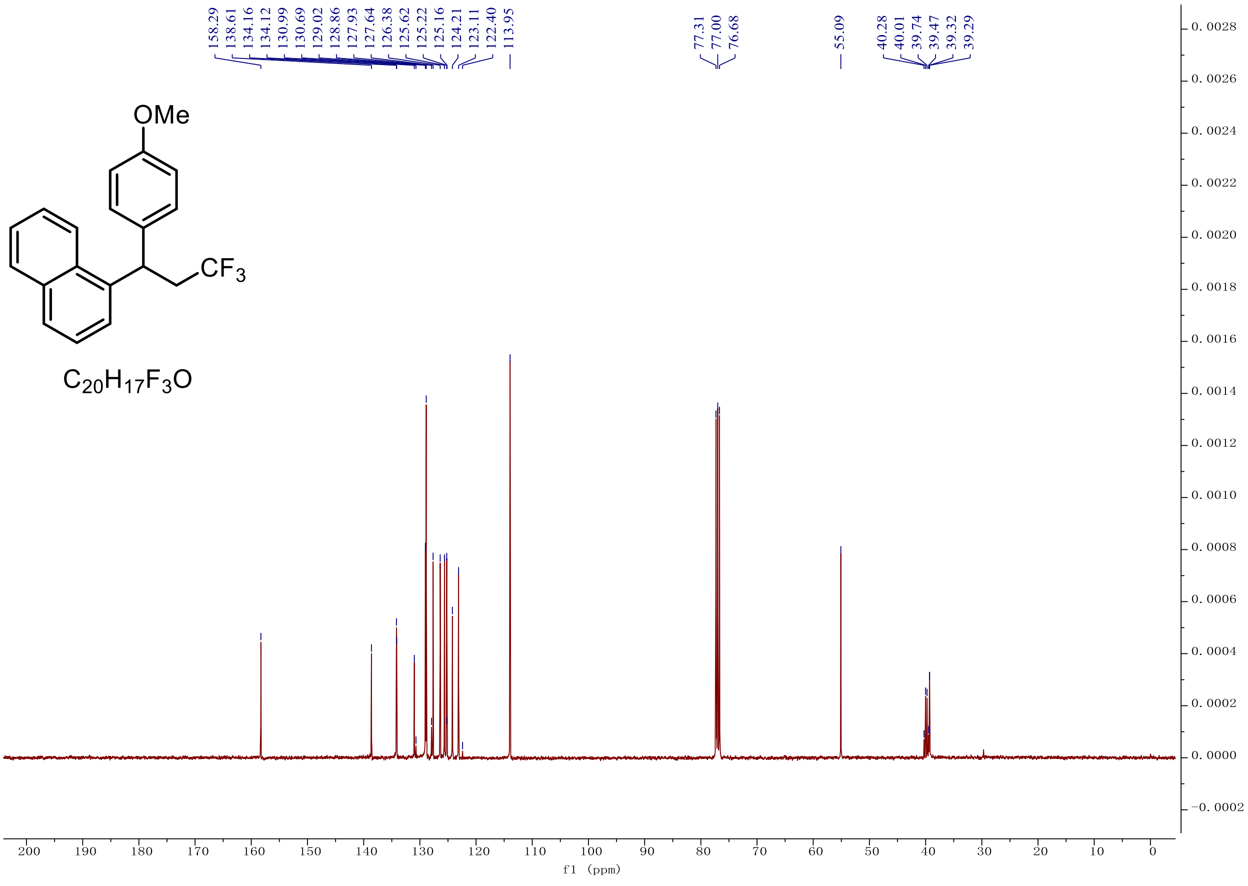

^{19}F NMR (376 MHz, $CDCl_3$) spectrum of 2m

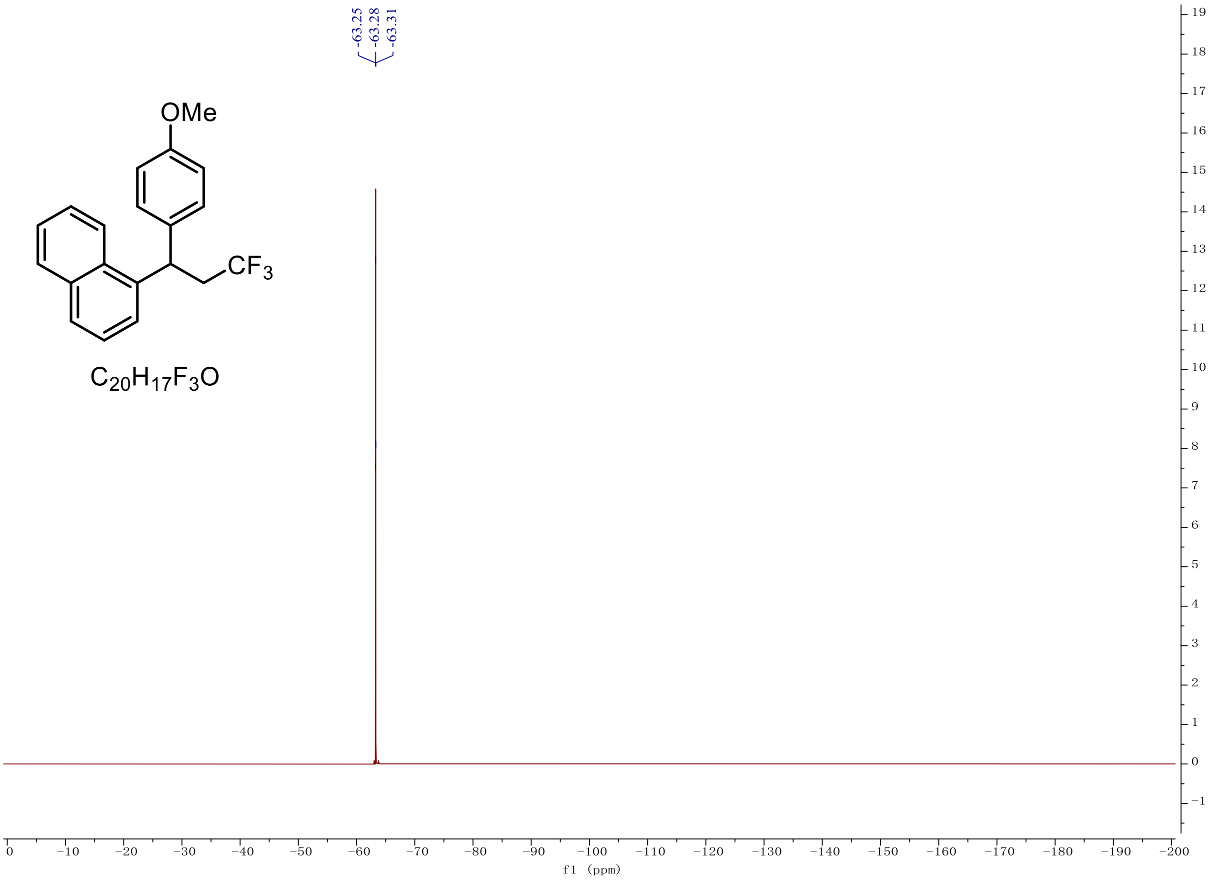

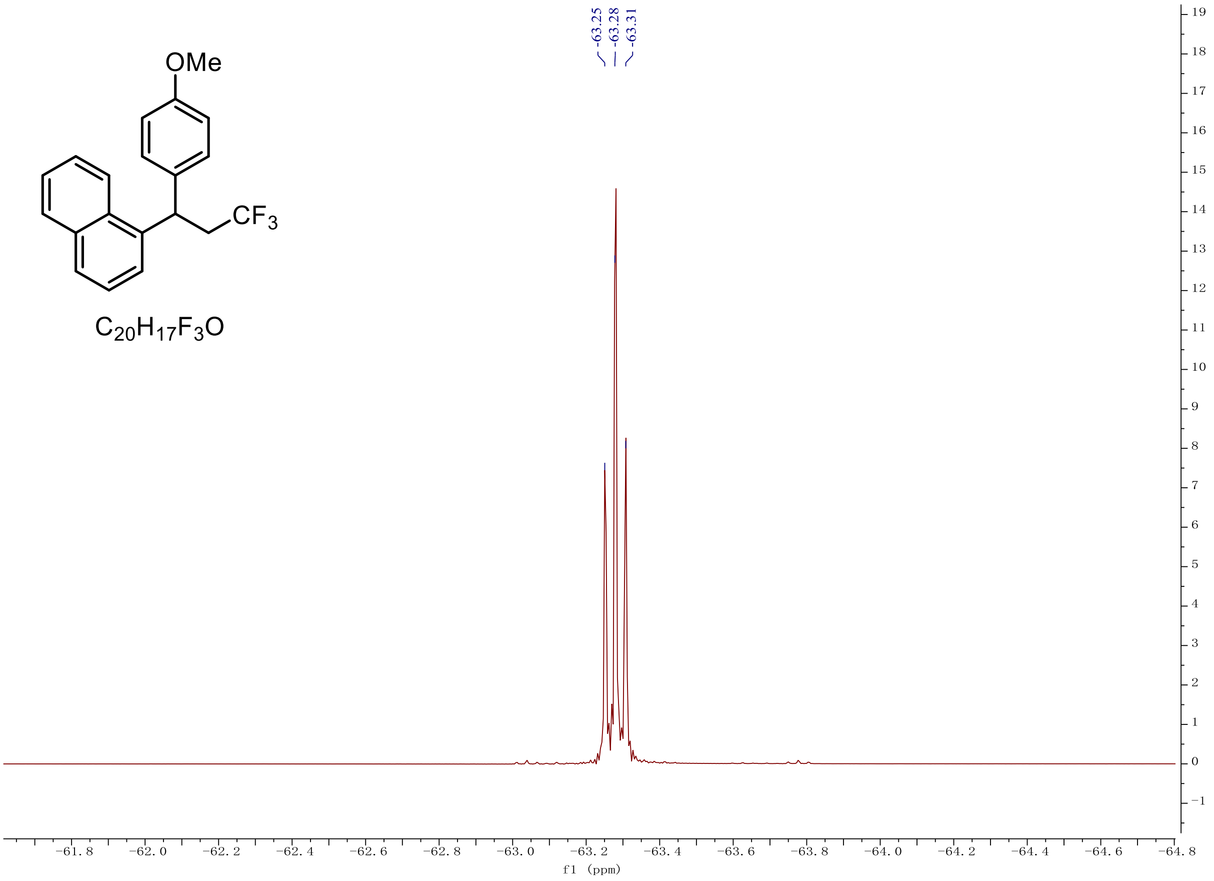

^{1}H NMR (400 MHz, CDCl3) spectrum of 2n

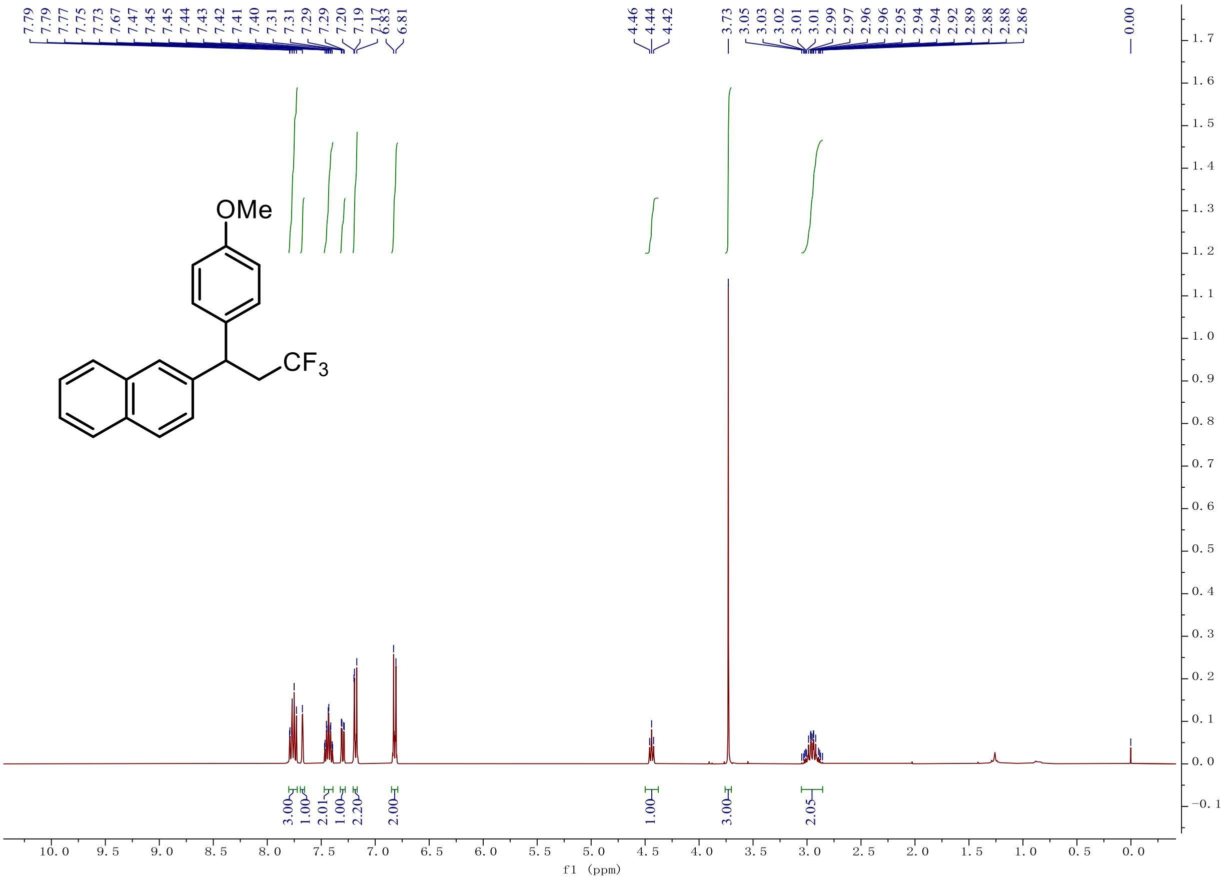

^{13}C NMR (101 MHz, CDCl3) spectrum of 2n

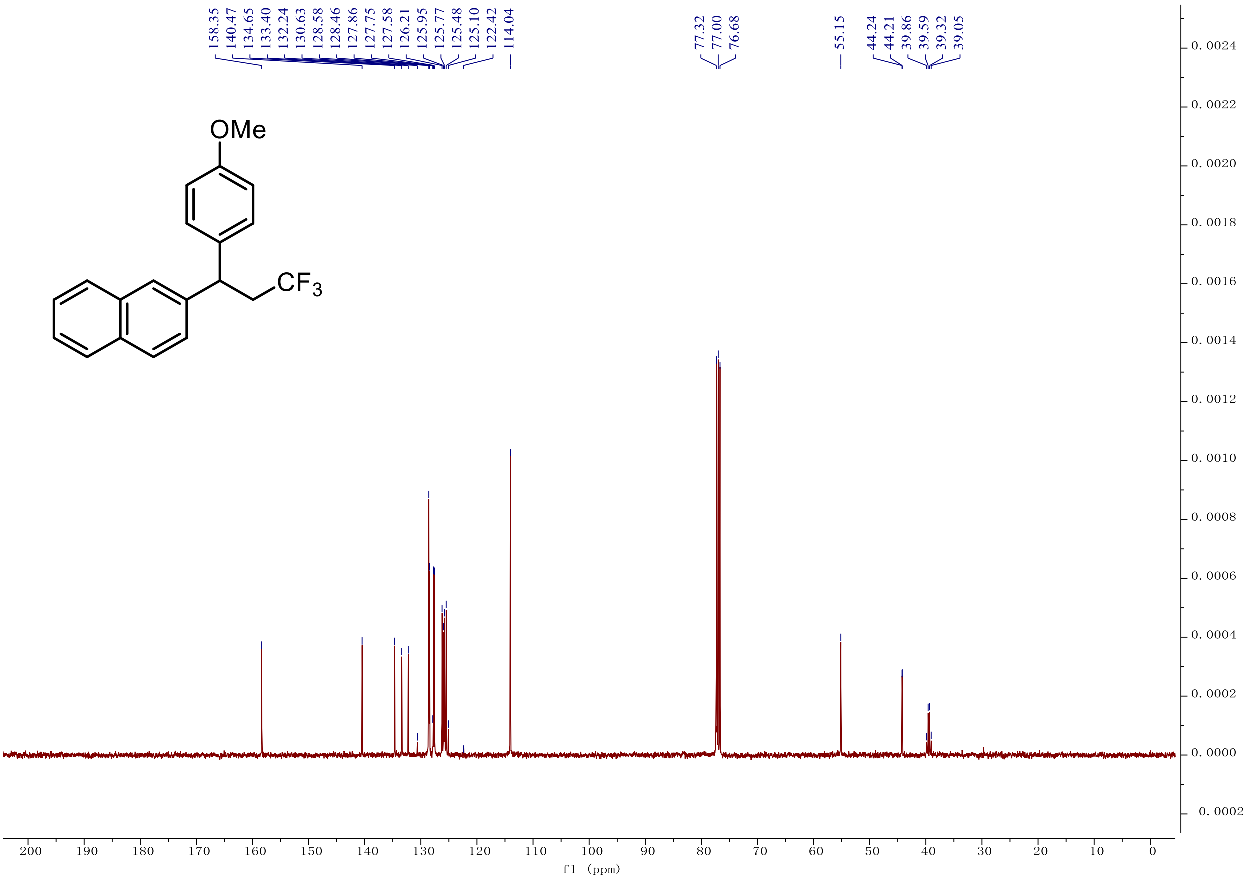

^{19}F NMR (376 MHz, $CDCl_3$) spectrum of 2n

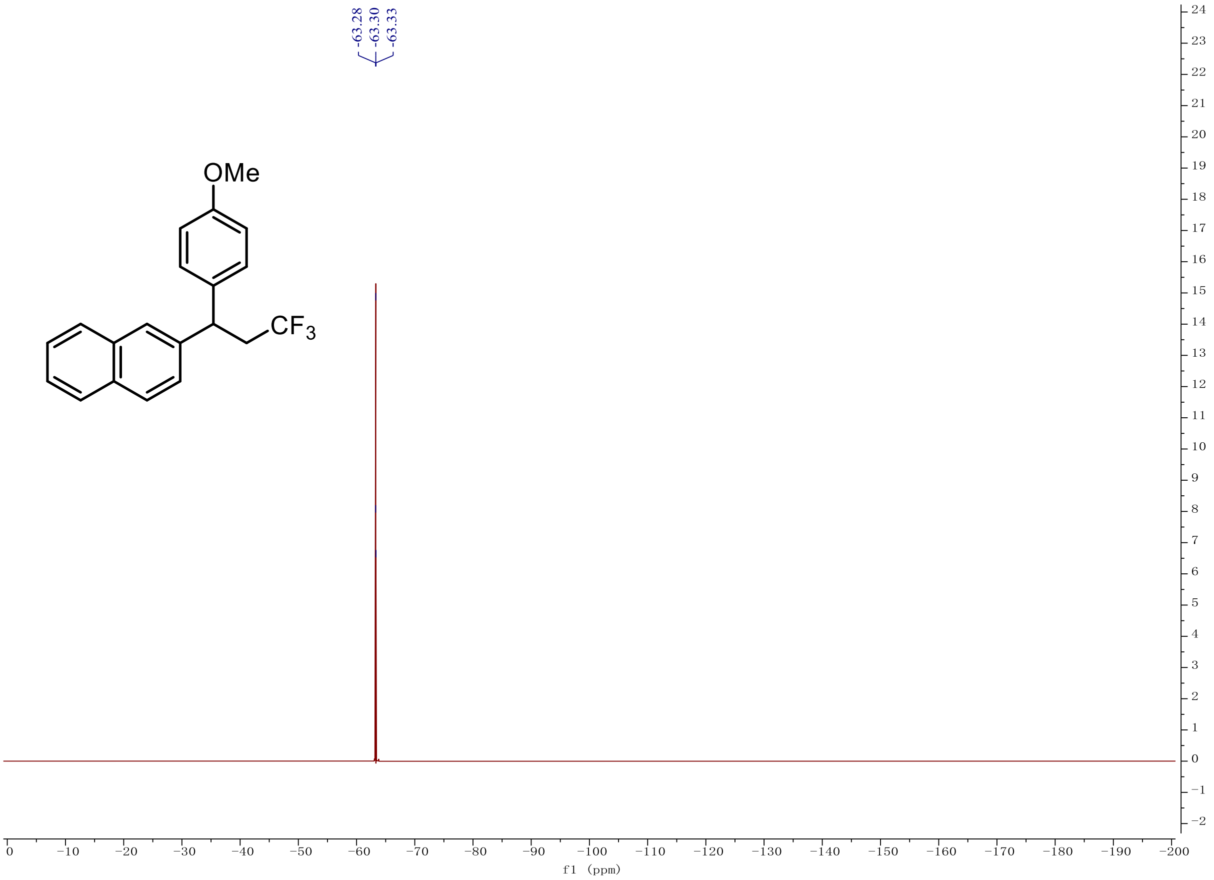

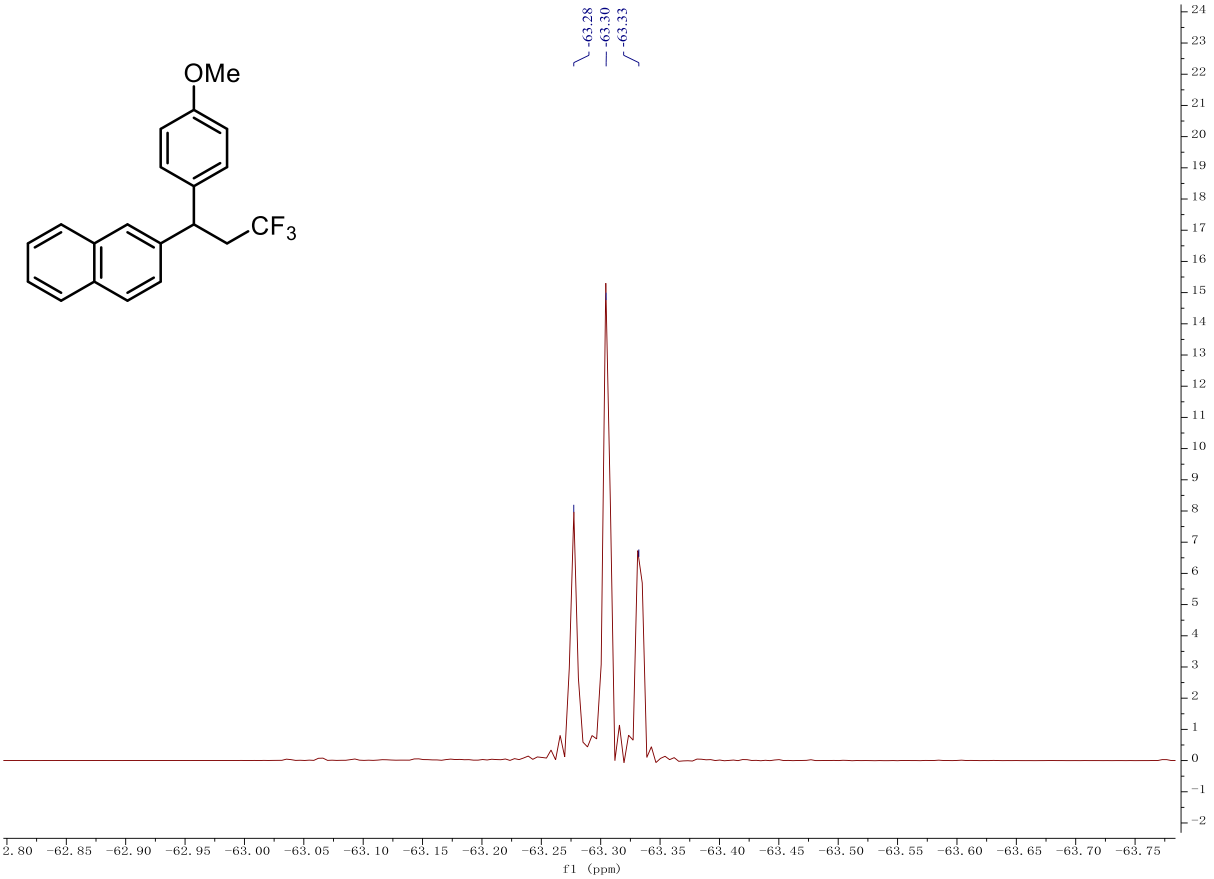

^{1}H NMR (400 MHz, $CDCl_3$) spectrum of 2o

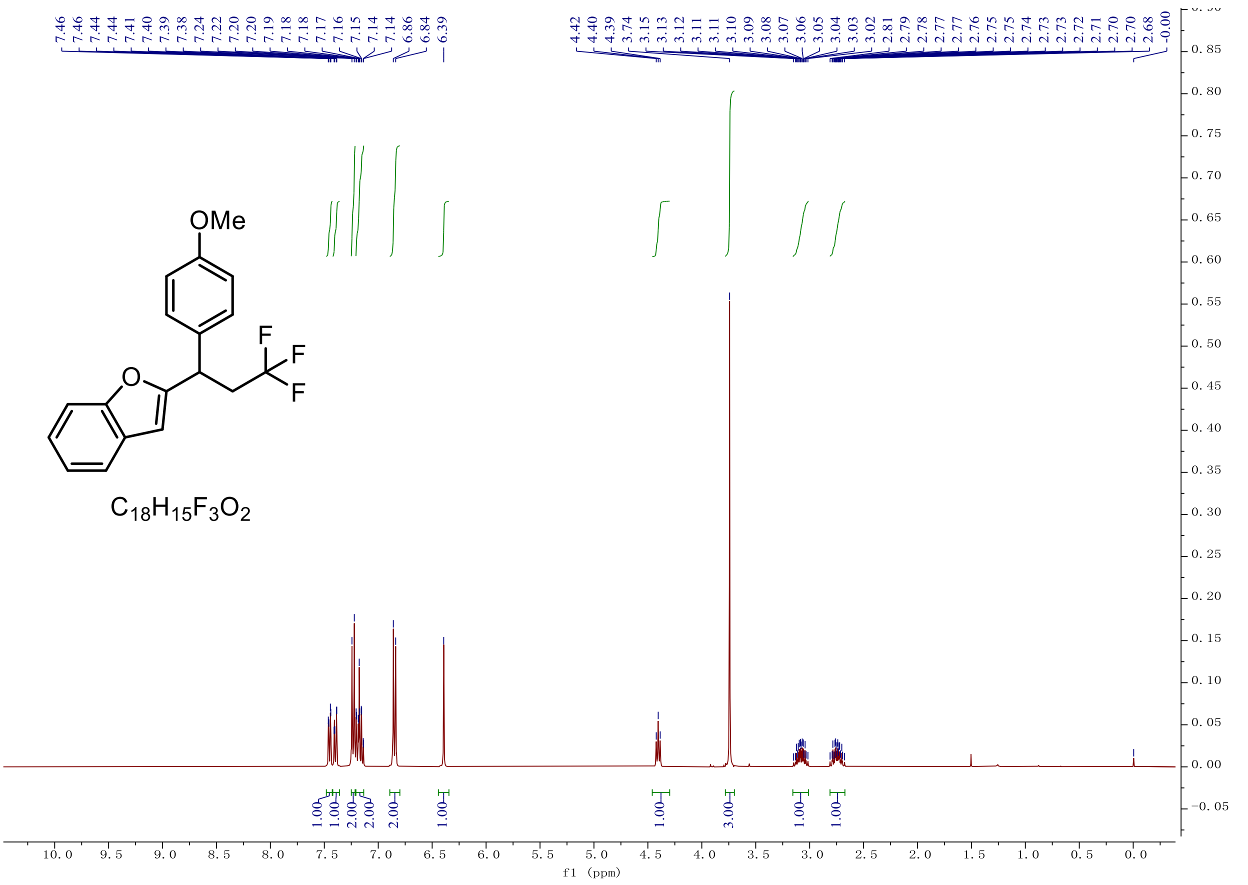

^{13}C NMR (101 MHz, $CDCl_3$) spectrum of 2o

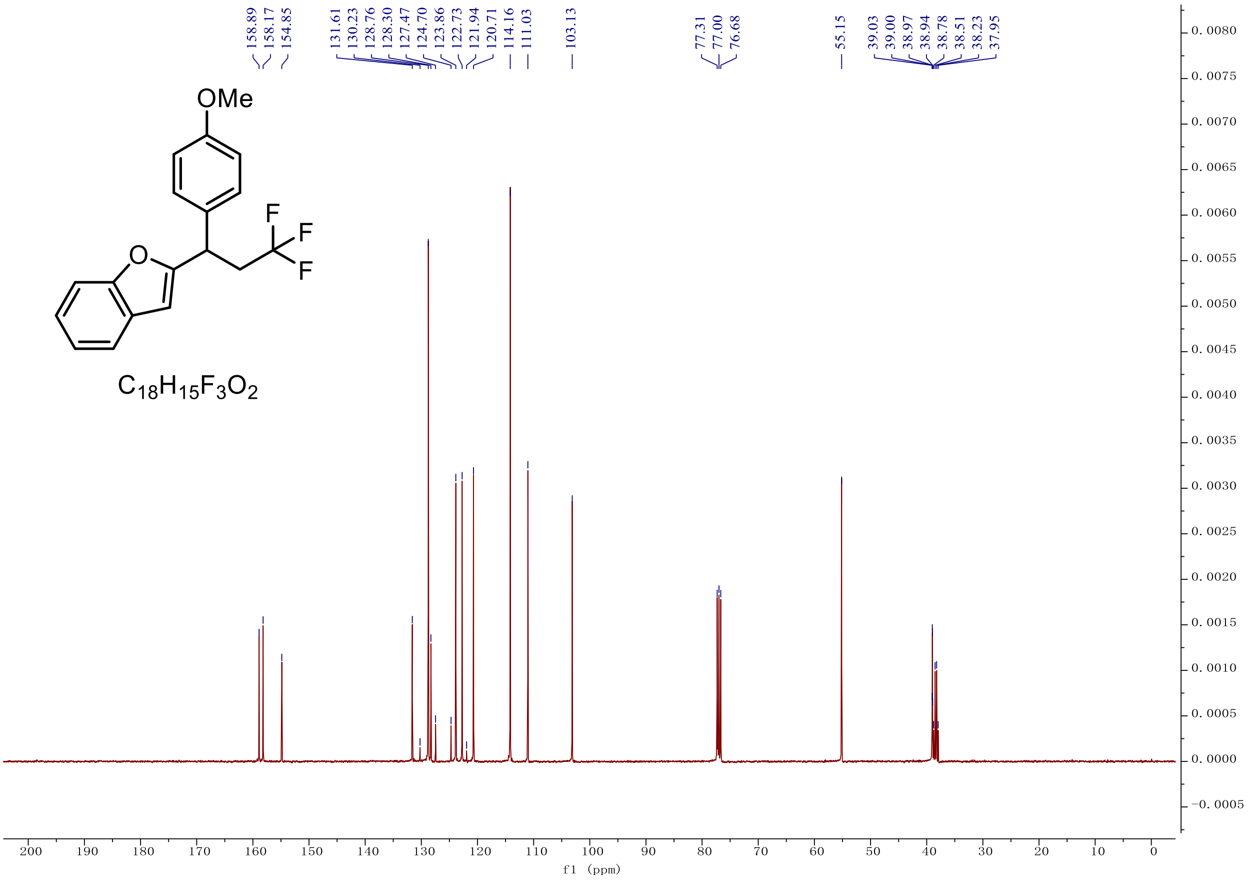

^{19}F NMR (376 MHz, CDCl3) spectrum of 2o

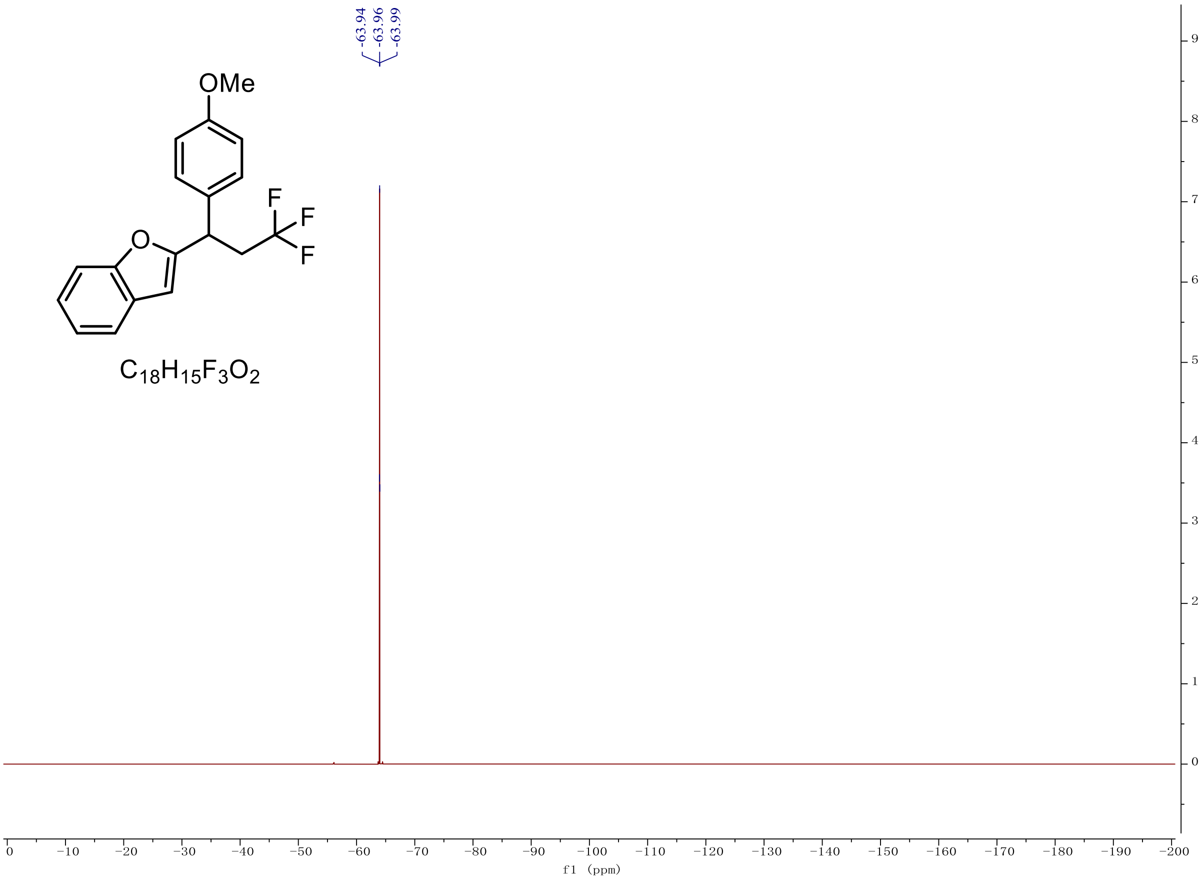

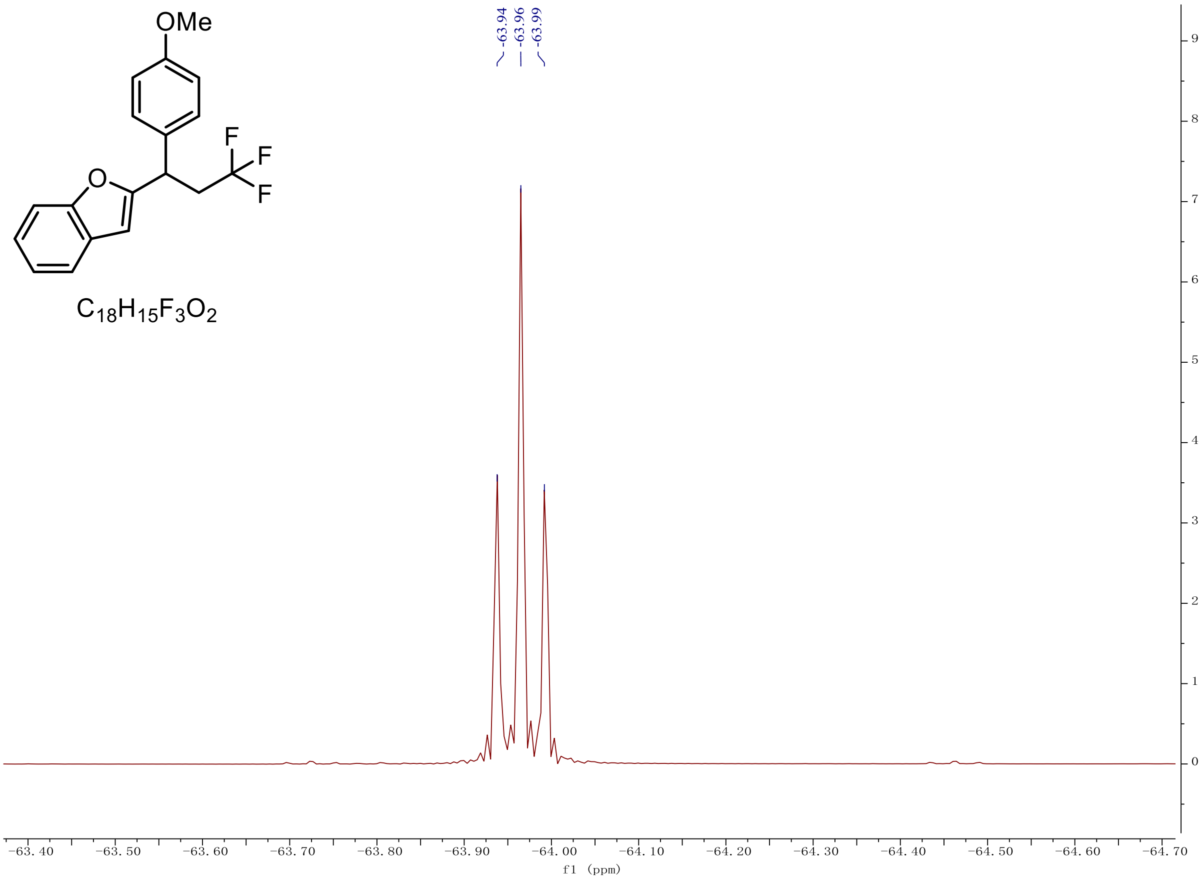

^{1}H NMR (400 MHz, $CDCl_3$) spectrum of 2p

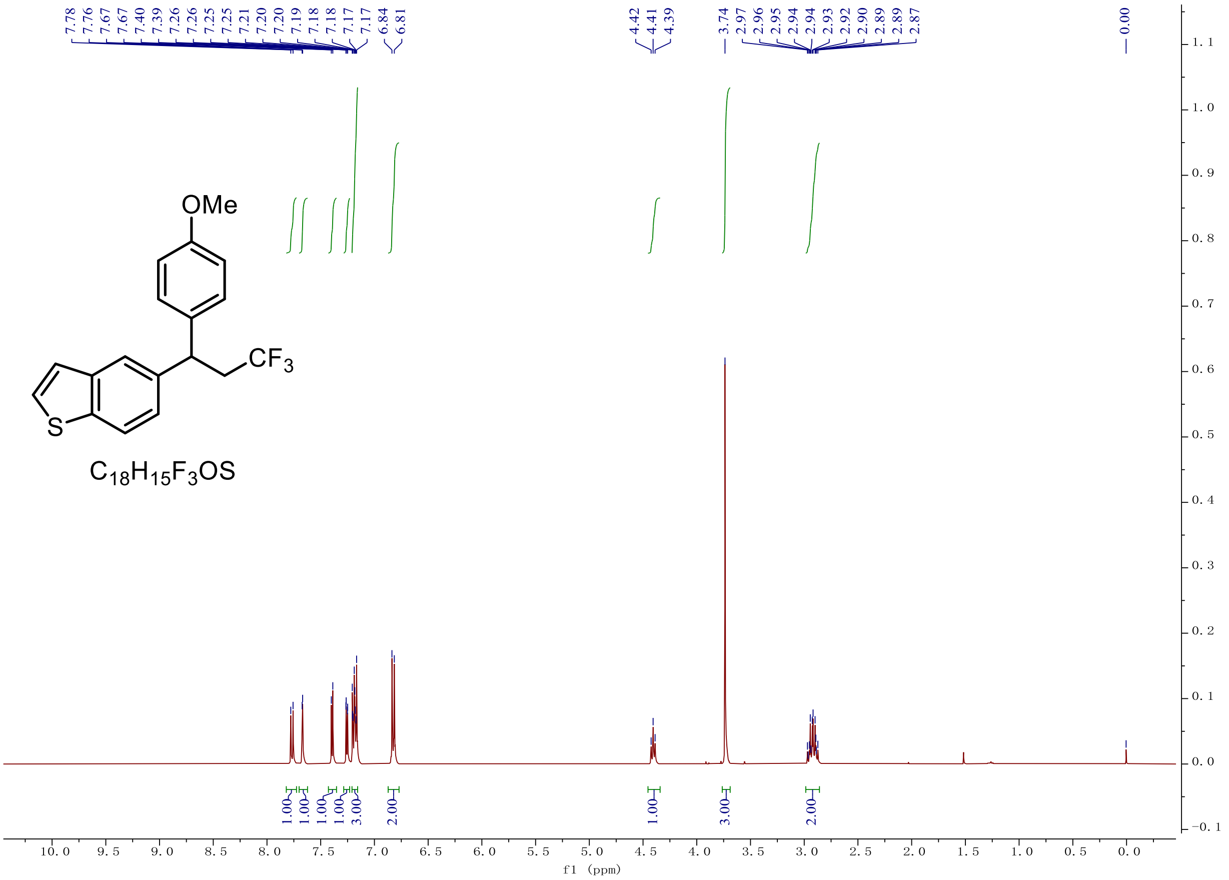

^{13}C NMR (101 MHz, $CDCl_3$) spectrum of 2p

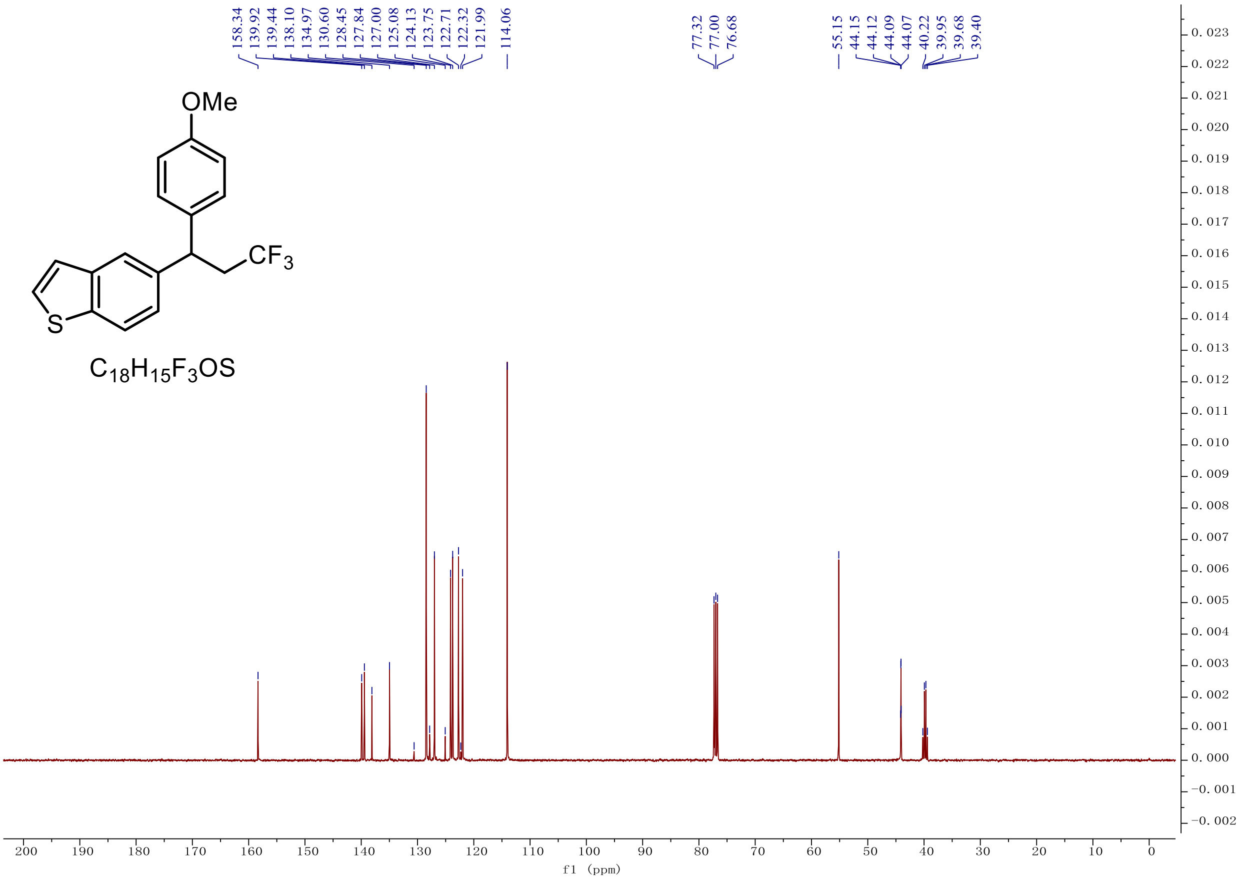

^{19}F NMR (376 MHz, $CDCl_3$) spectrum of 2p

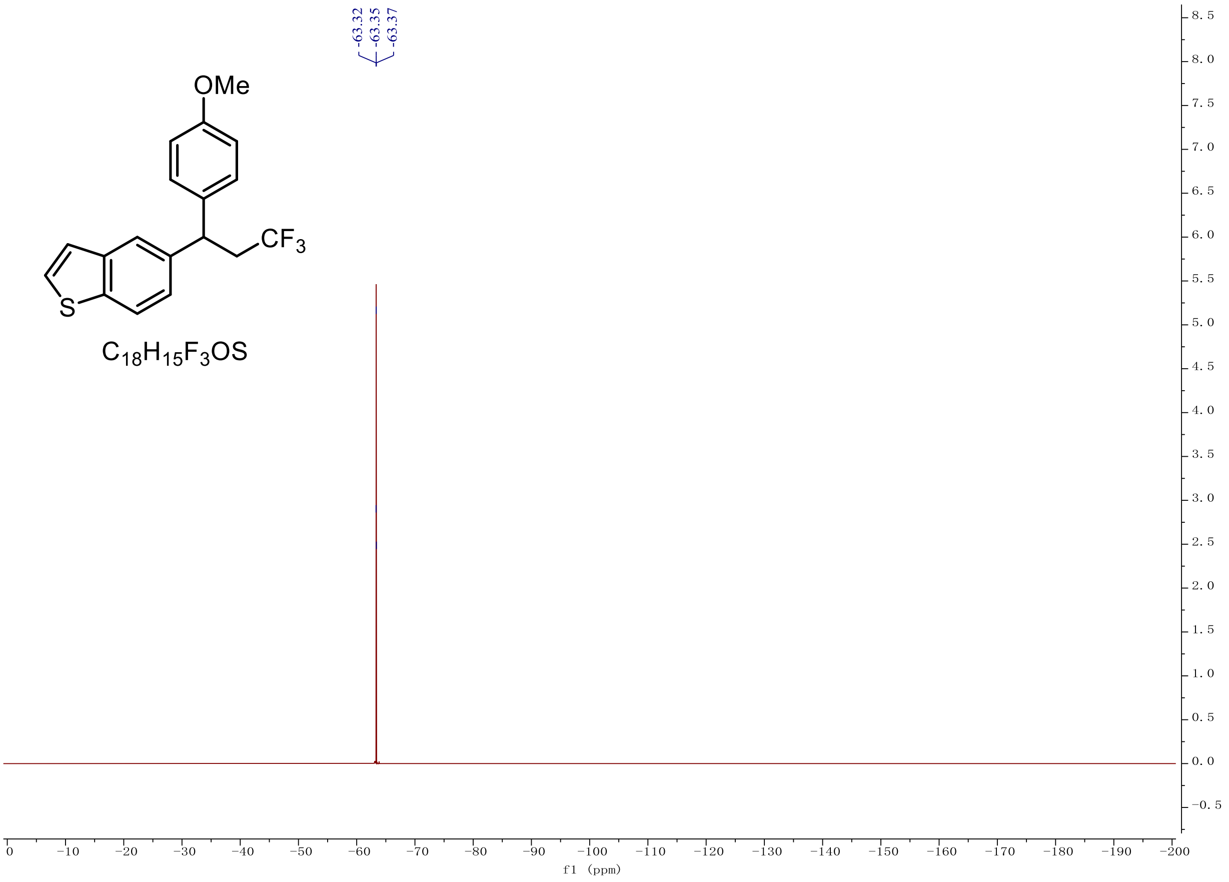

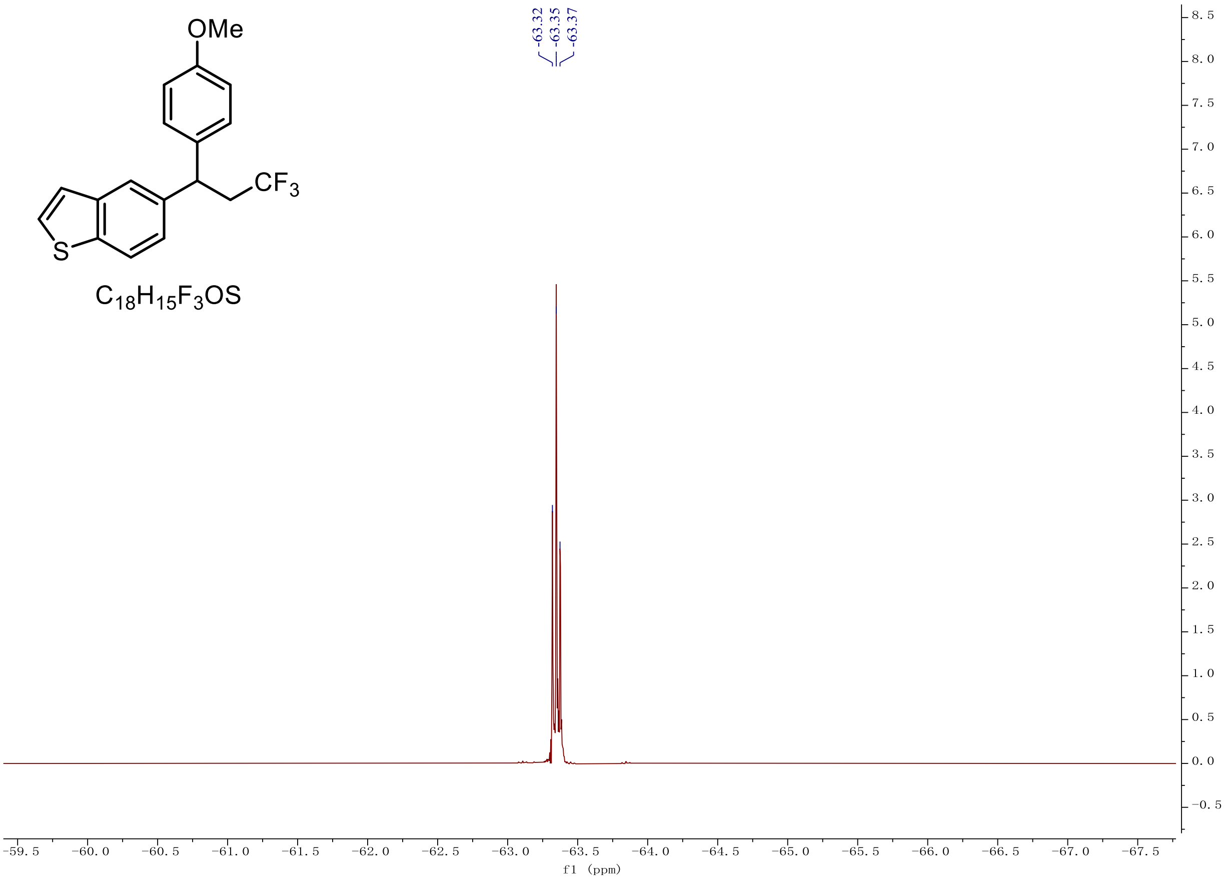

^{1}H NMR (400 MHz, CDCl3) spectrum of 2q

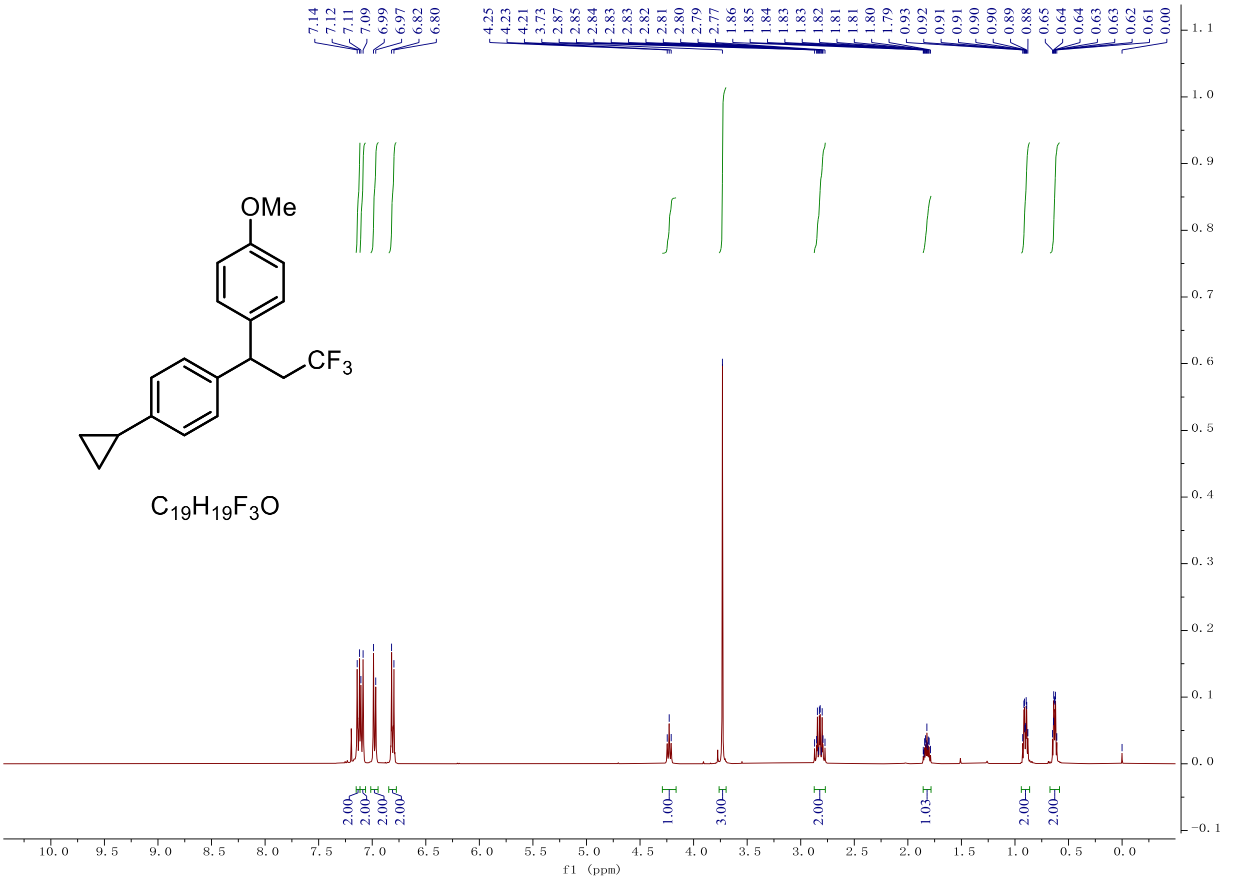

^{13}C NMR (101 MHz, CDCl3) spectrum of 2q

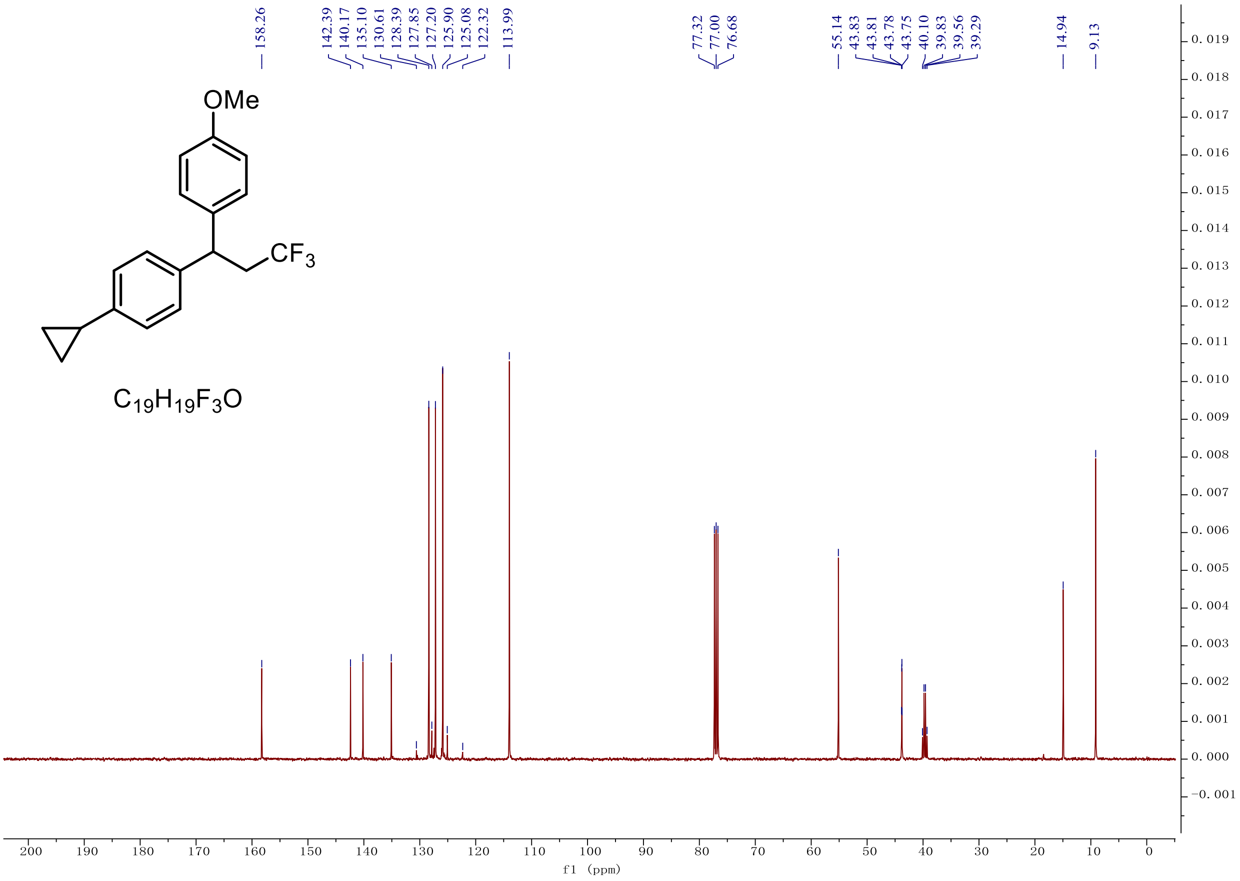

^{19}F NMR (376 MHz, CDCl3) spectrum of 2q

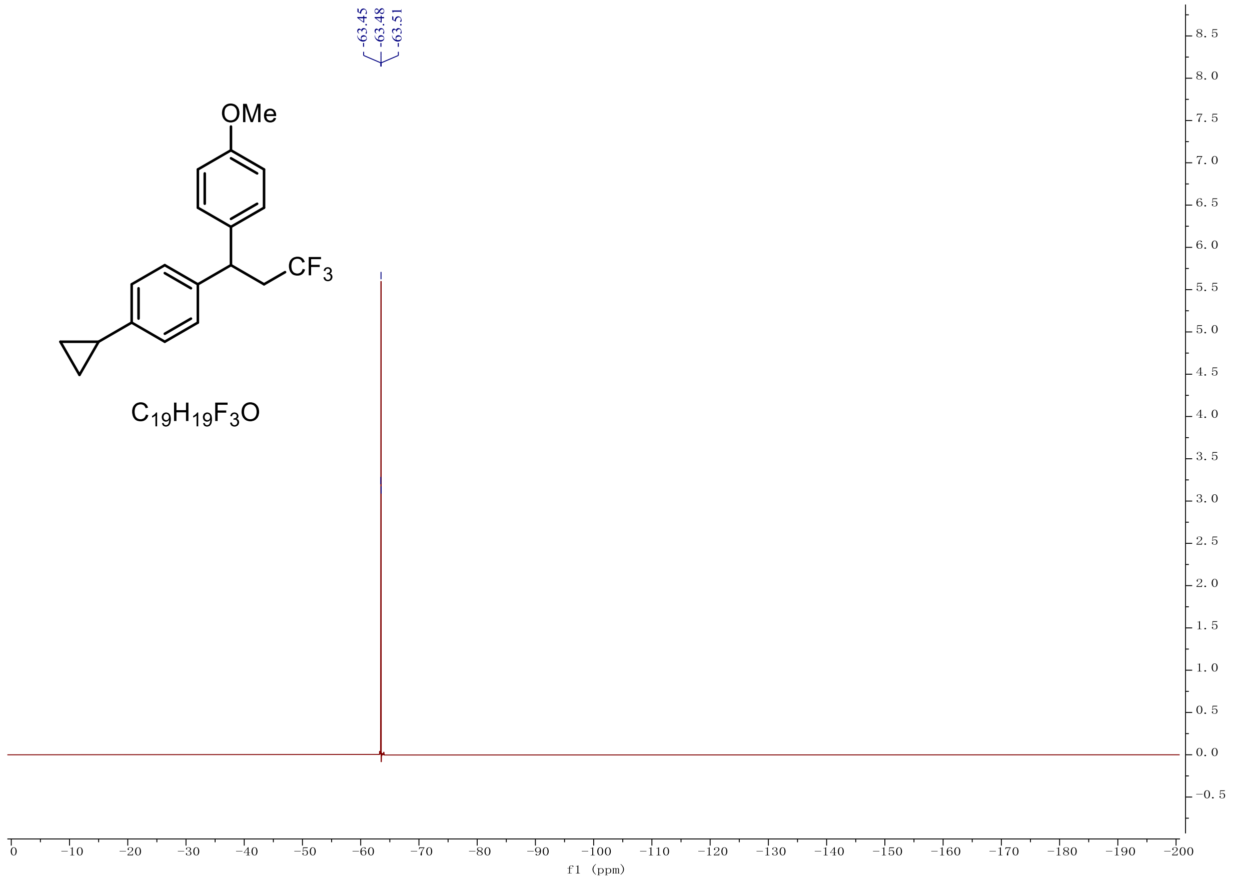

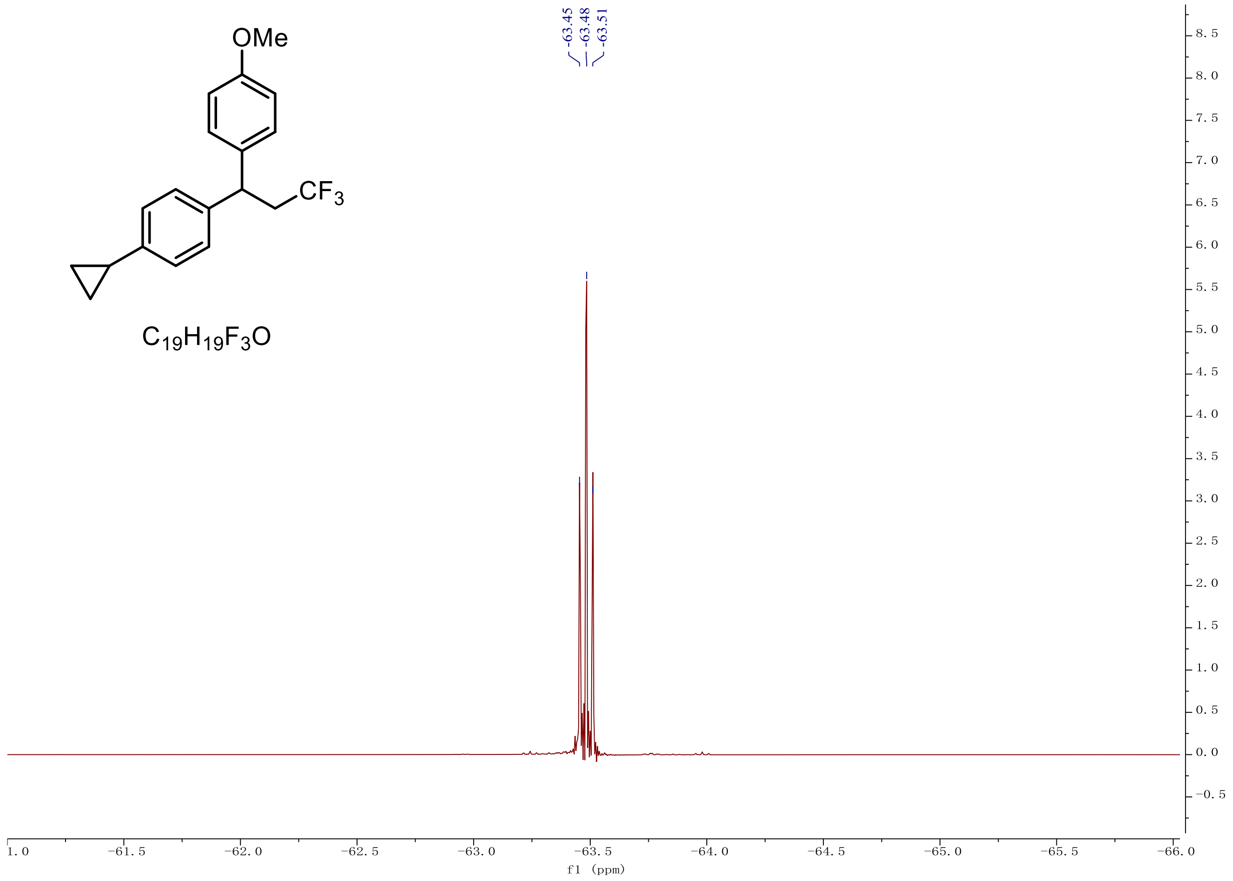

^{1}H NMR (400 MHz, $CDCl_3$) spectrum of 2r

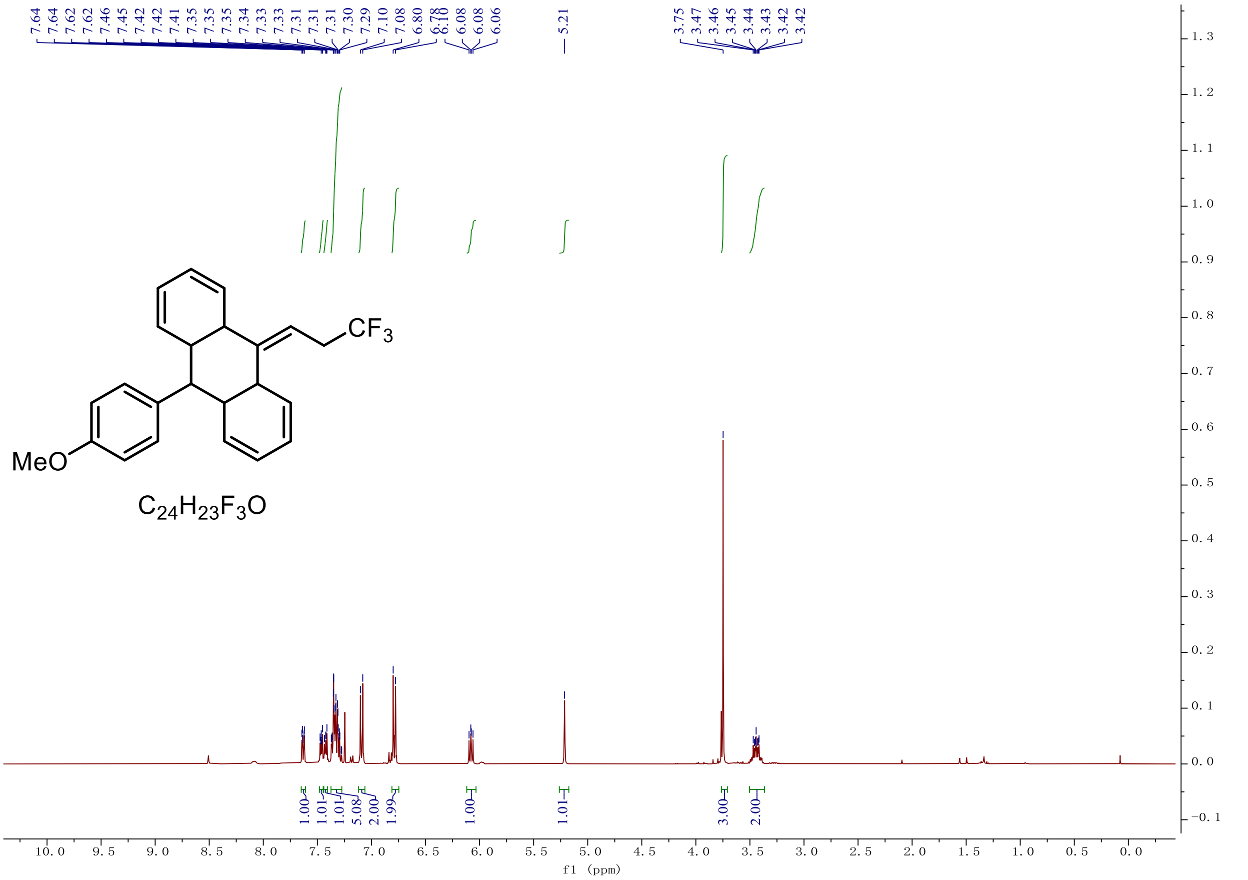

^{13}C NMR (101 MHz, $CDCl_3$) spectrum of 2r

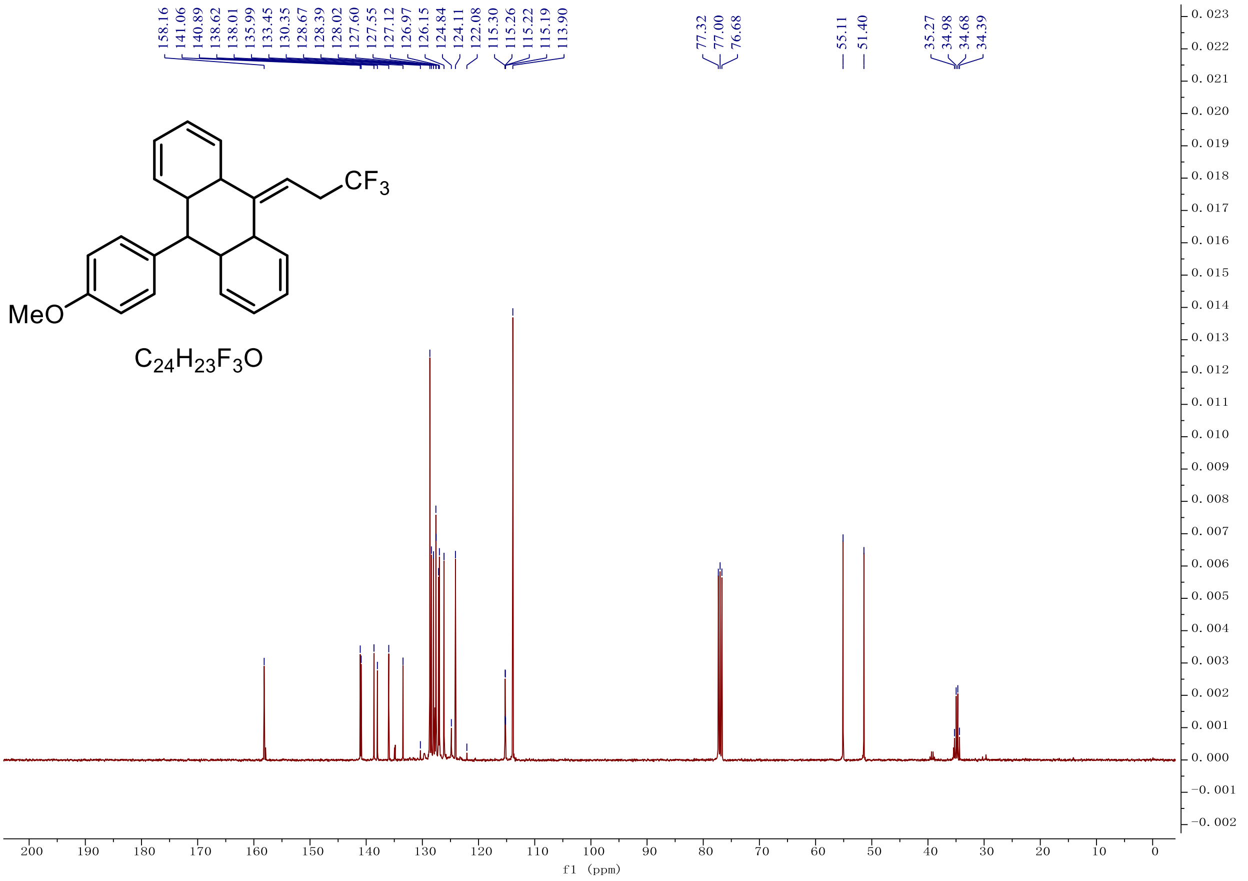

^{19}F NMR (376 MHz, $CDCl_3$) spectrum of 2r

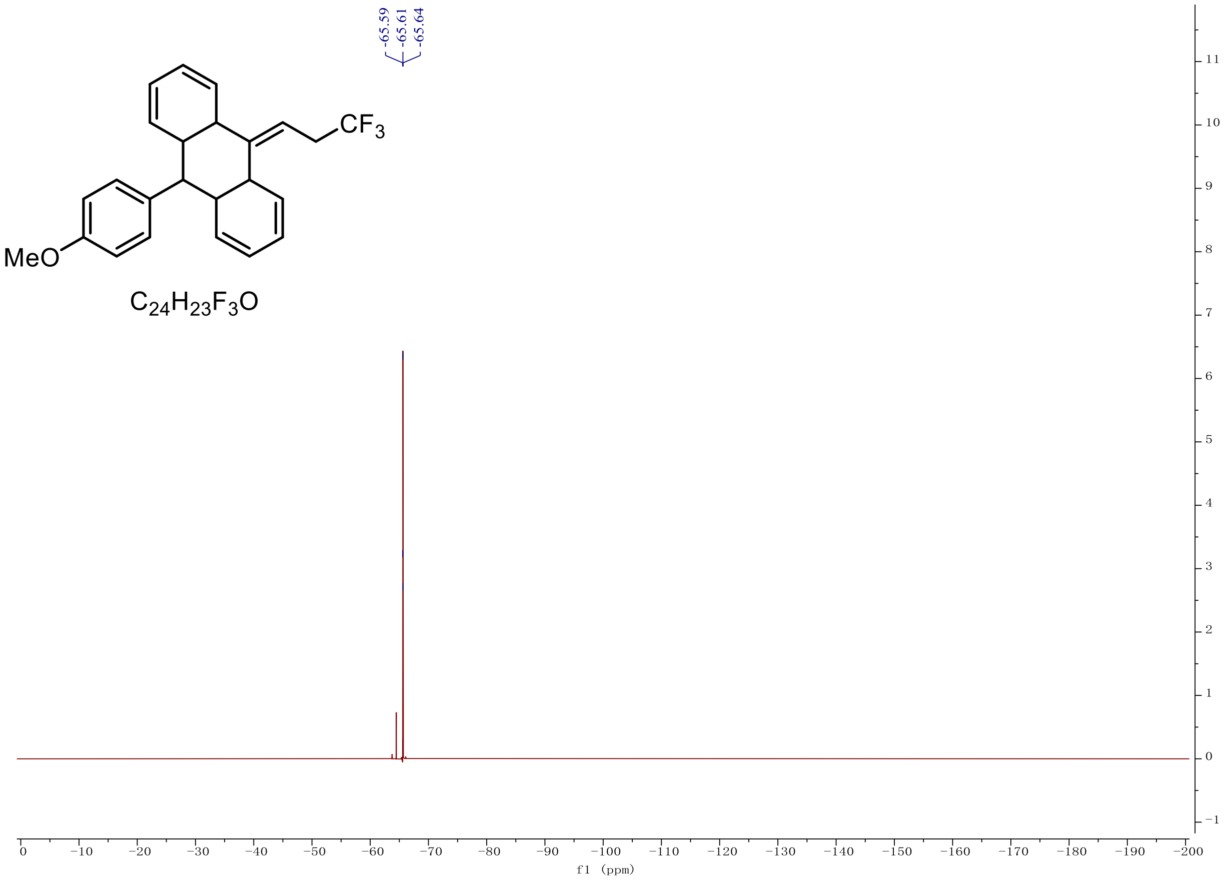

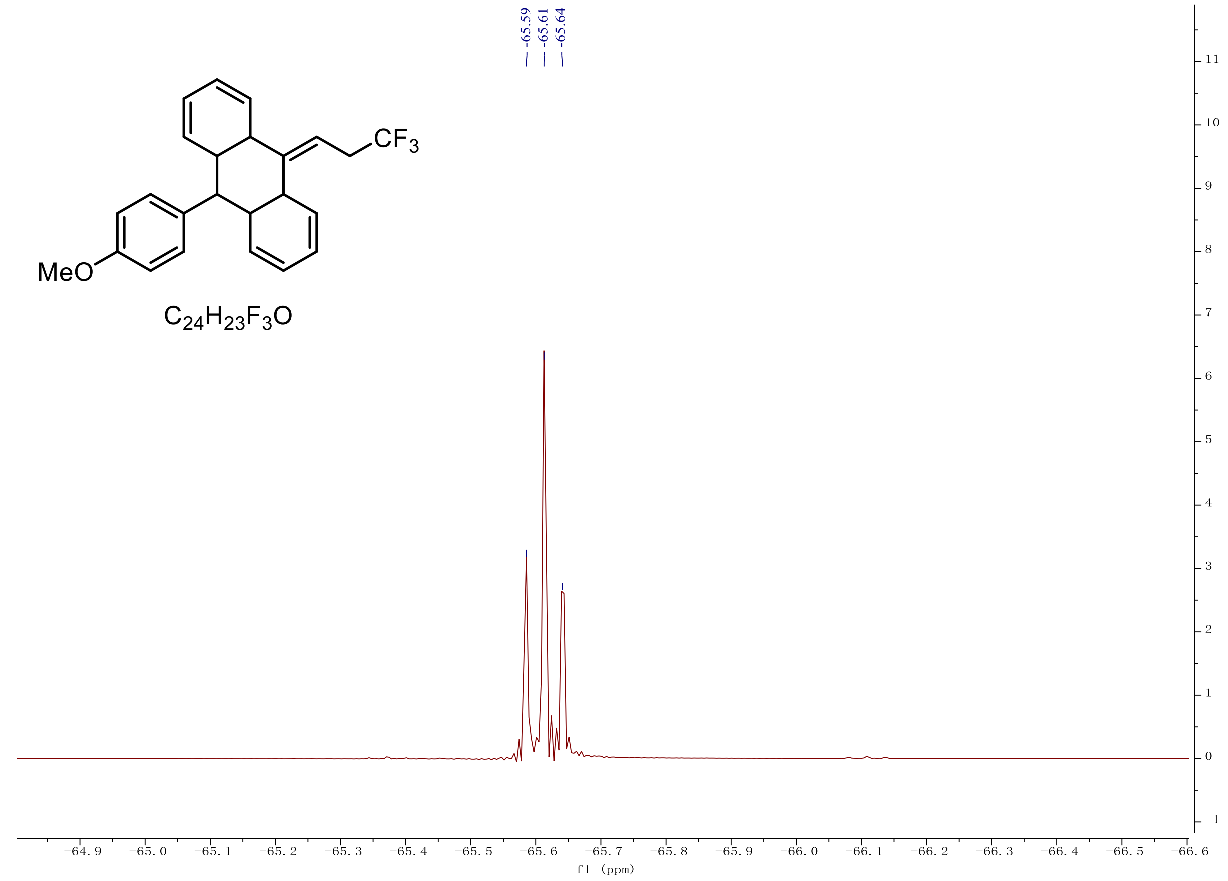

^{1}H NMR (400 MHz, $CDCl_3$) spectrum of 2s

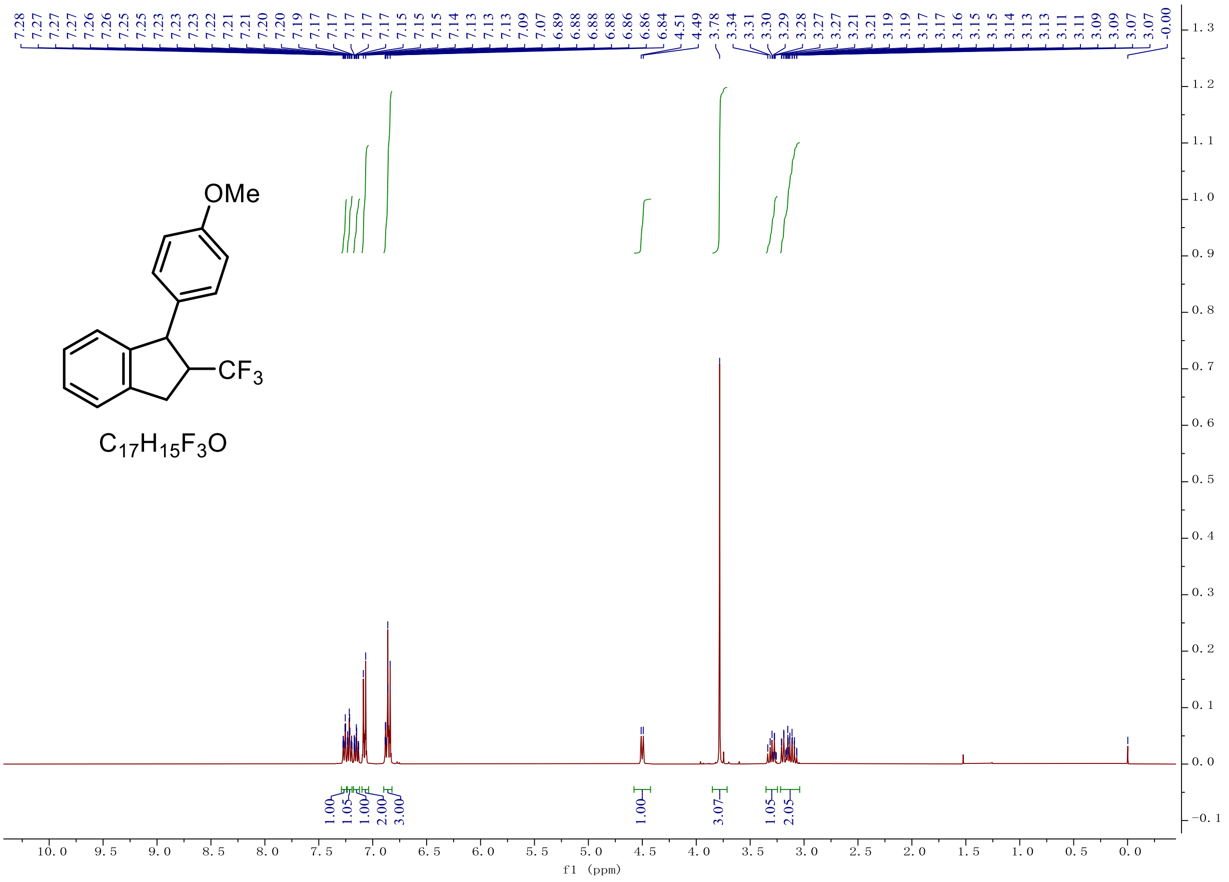

^{13}C NMR (101 MHz, $CDCl_3$) spectrum of 2s

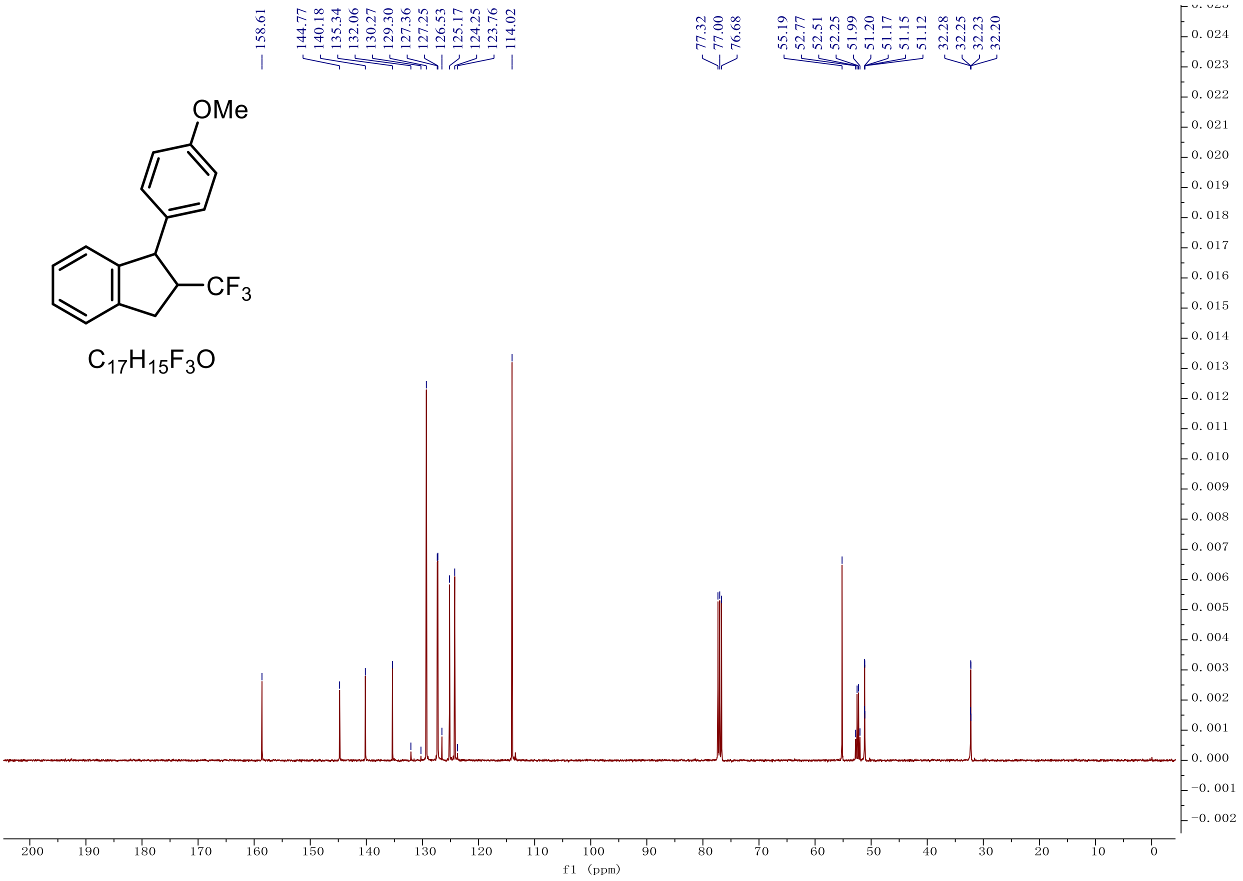

^{19}F NMR (376 MHz, $CDCl_3$) spectrum of 2s

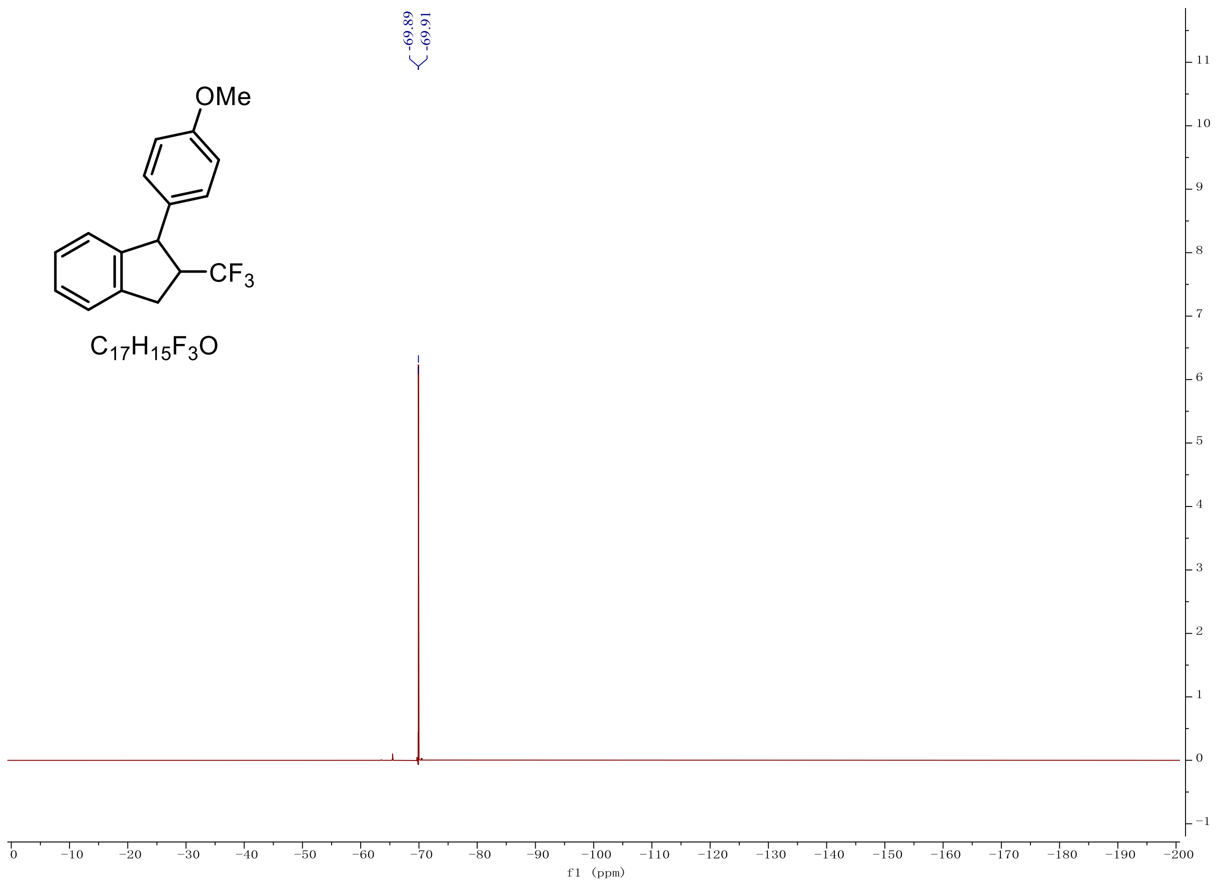

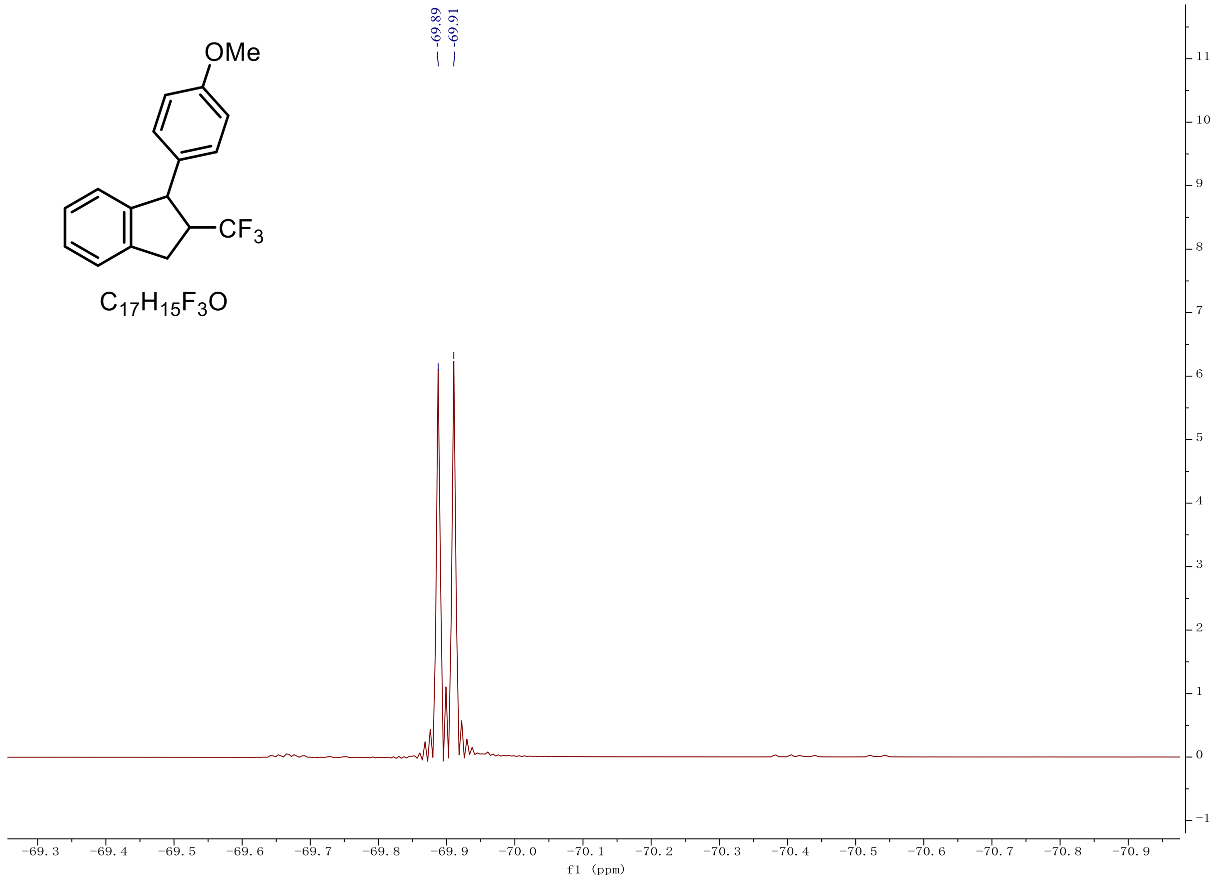

^{1}H NMR (400 MHz, $CDCl_3$) spectrum of 2s’

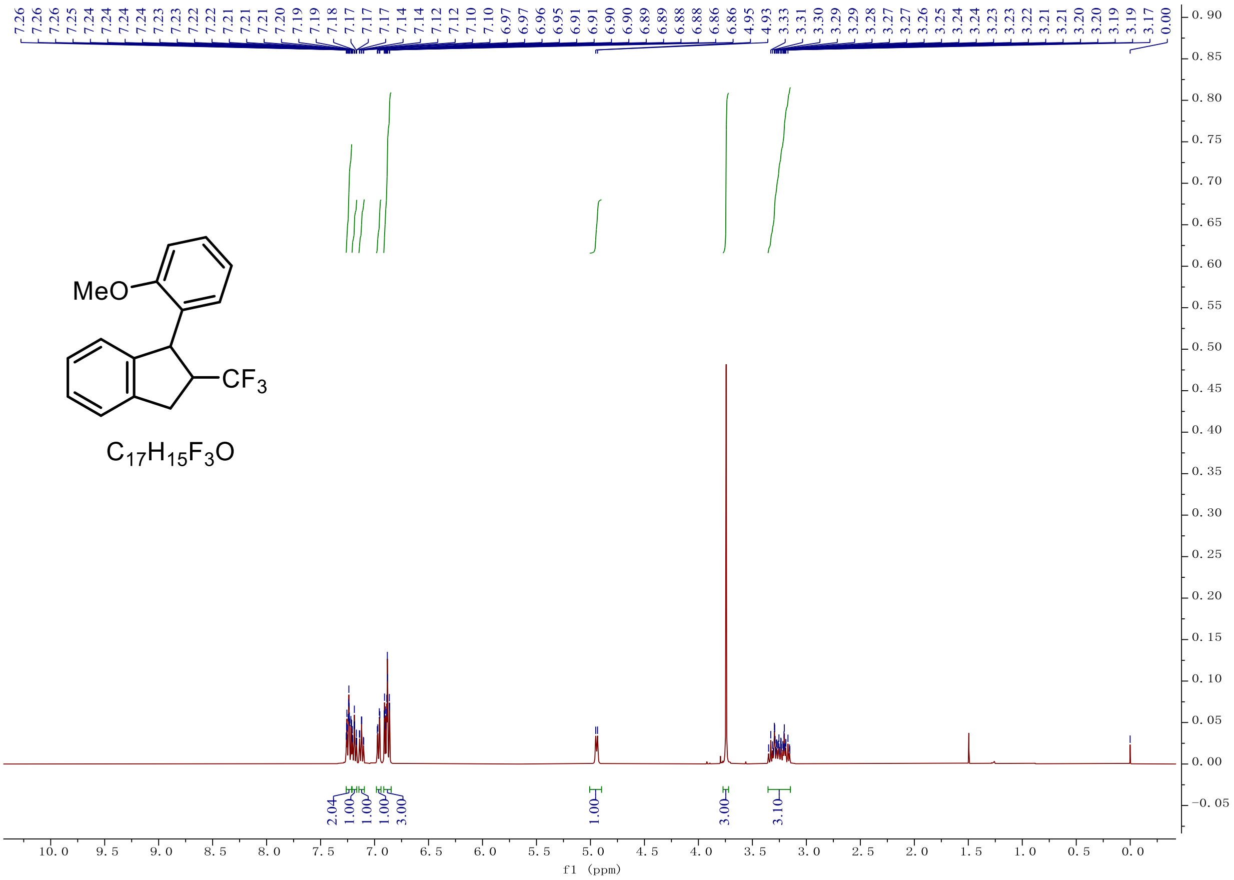

^{13}C NMR (101 MHz, $CDCl_3$) spectrum of 2s’

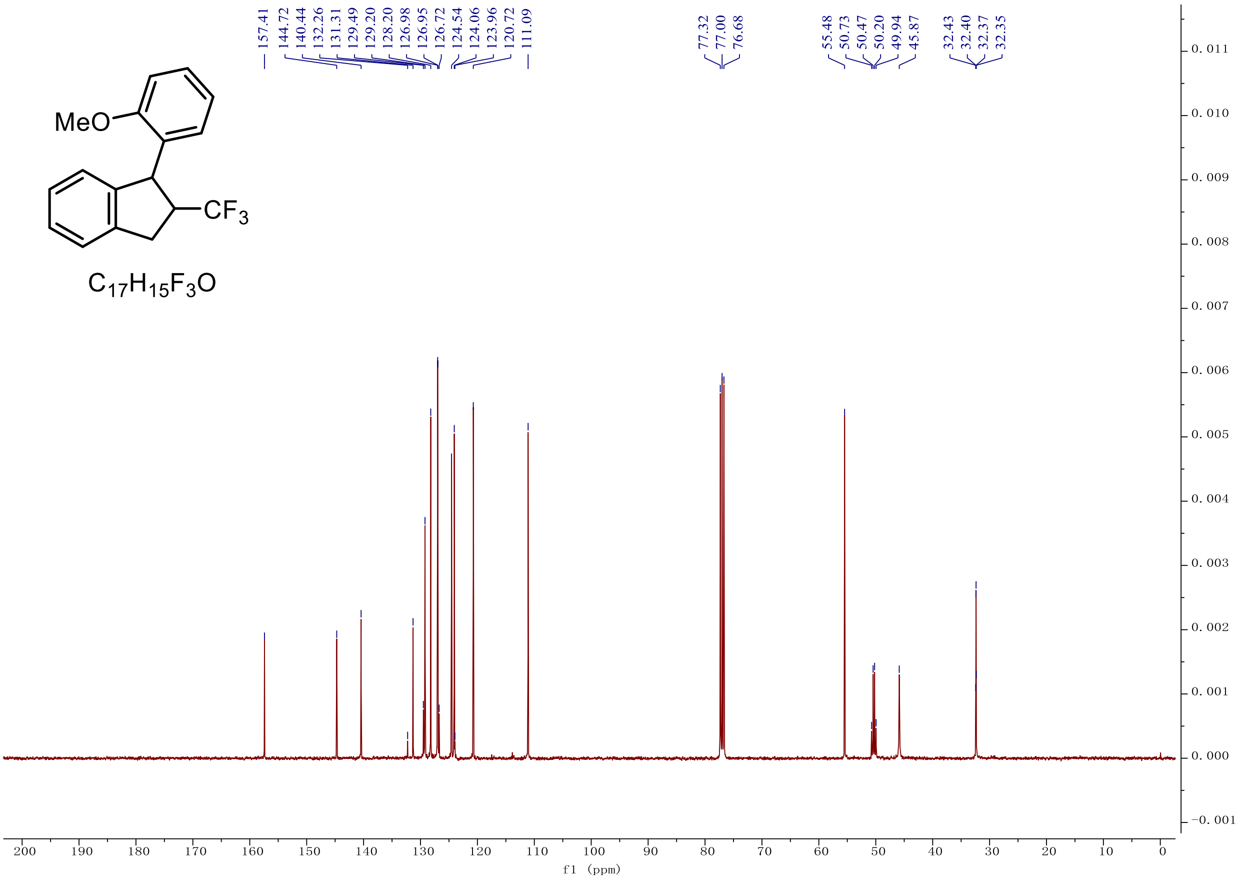

^{19}F NMR (376 MHz, $CDCl_3$) spectrum of 2s'

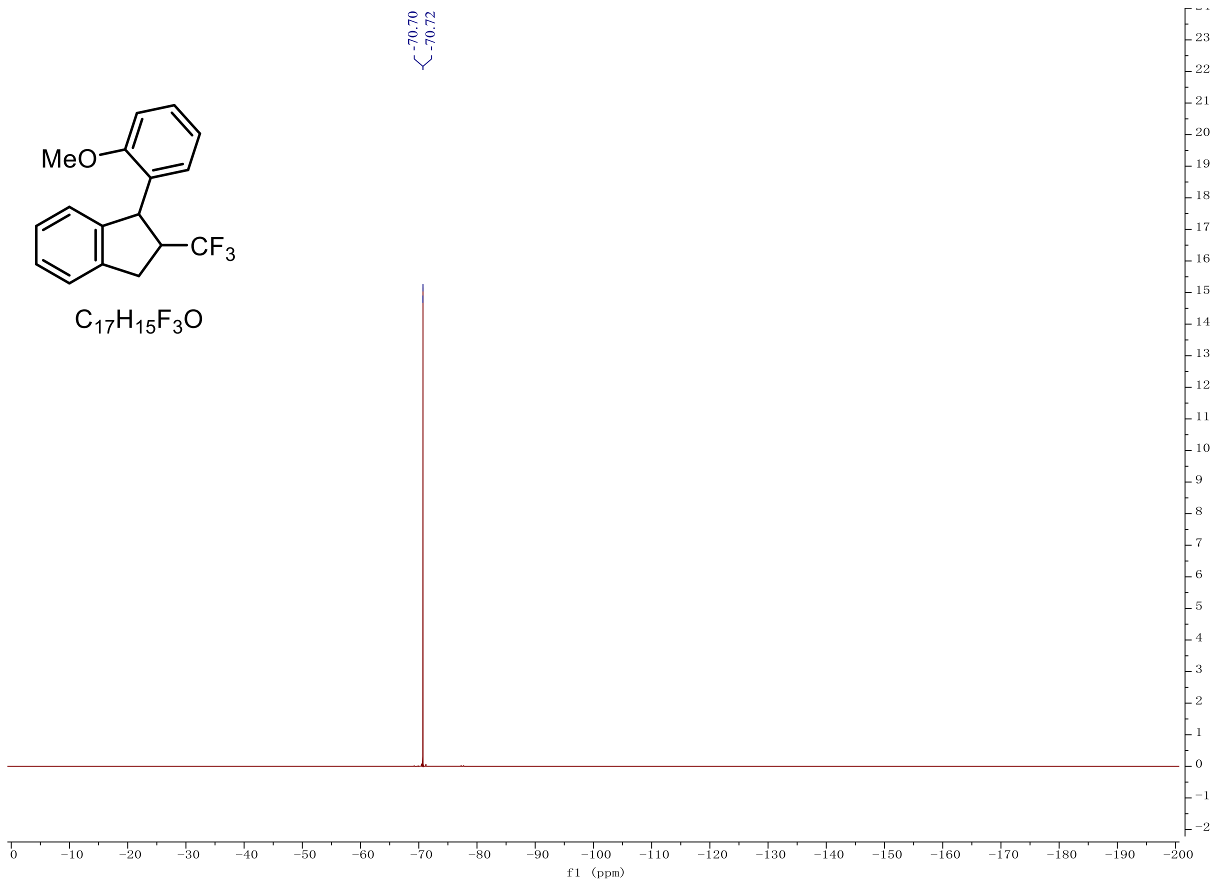

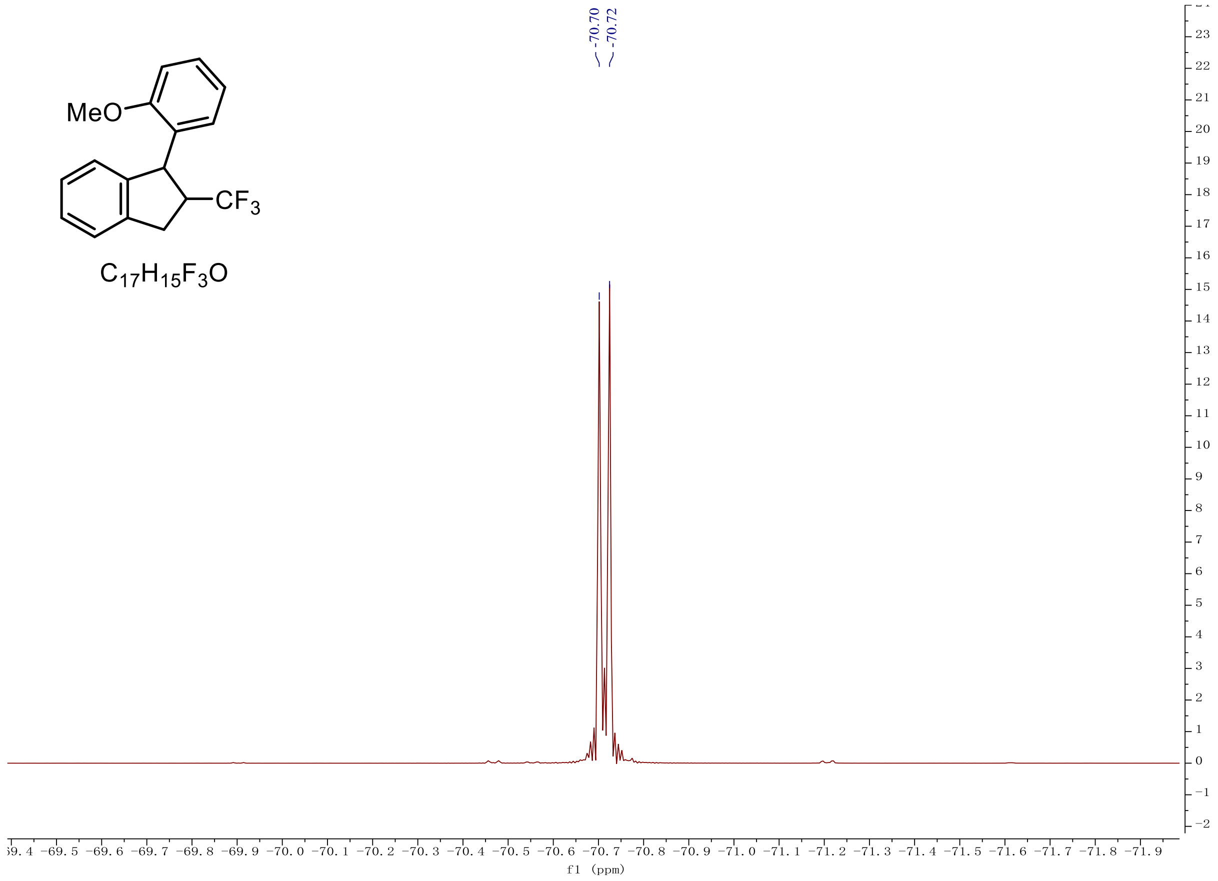

^{1}H NMR (400 MHz, $CDCl_3$) spectrum of 2t

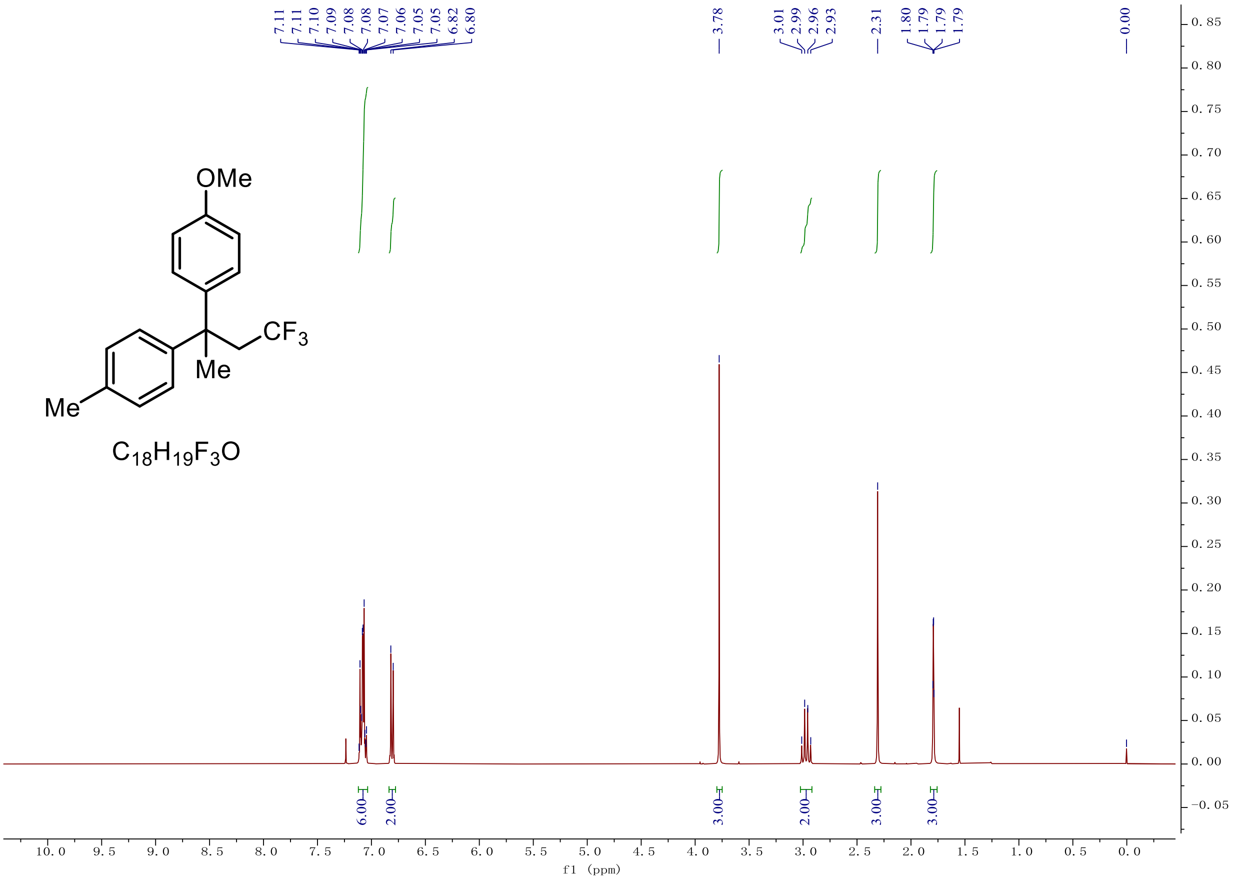

^{13}C NMR (101 MHz, $CDCl_3$) spectrum of 2t

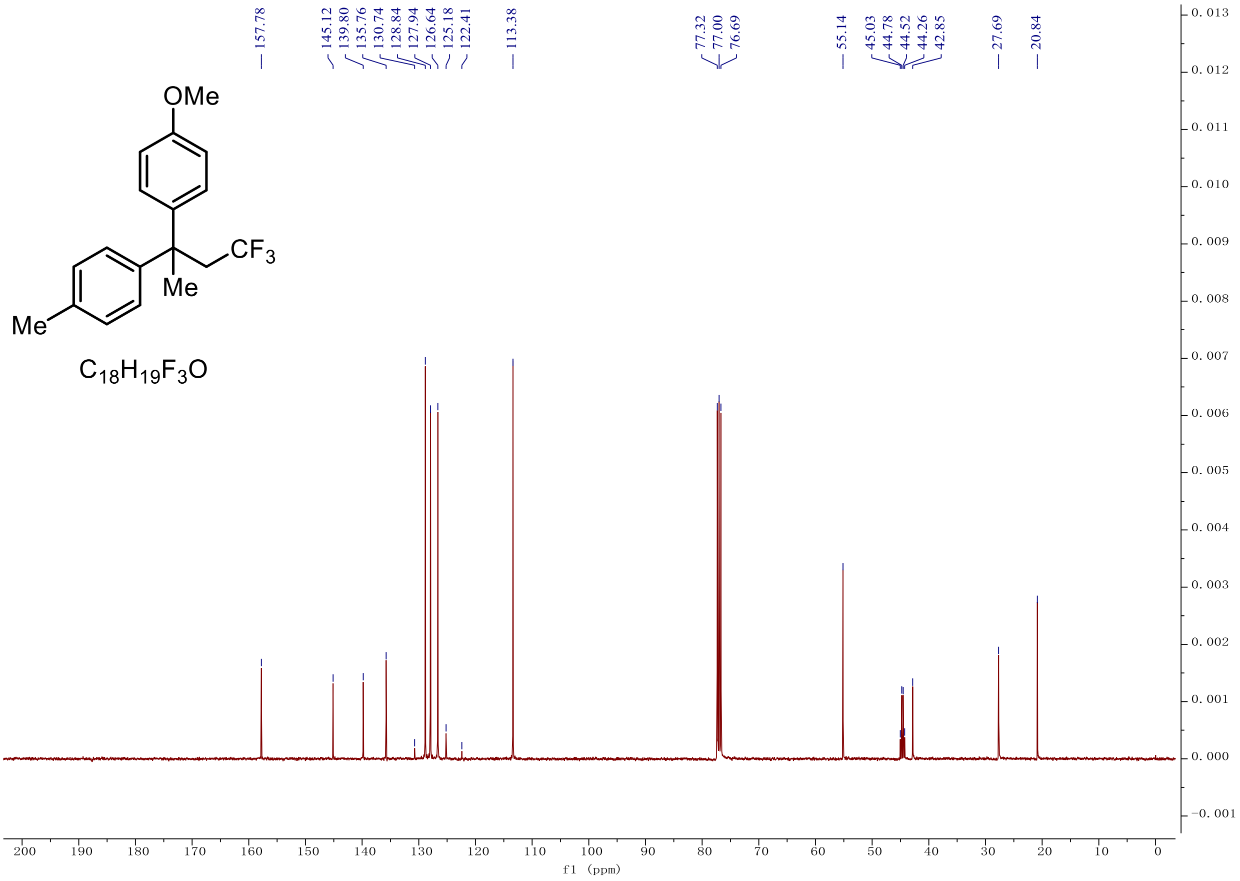

^{19}F NMR (376 MHz, $CDCl_3$) spectrum of 2t

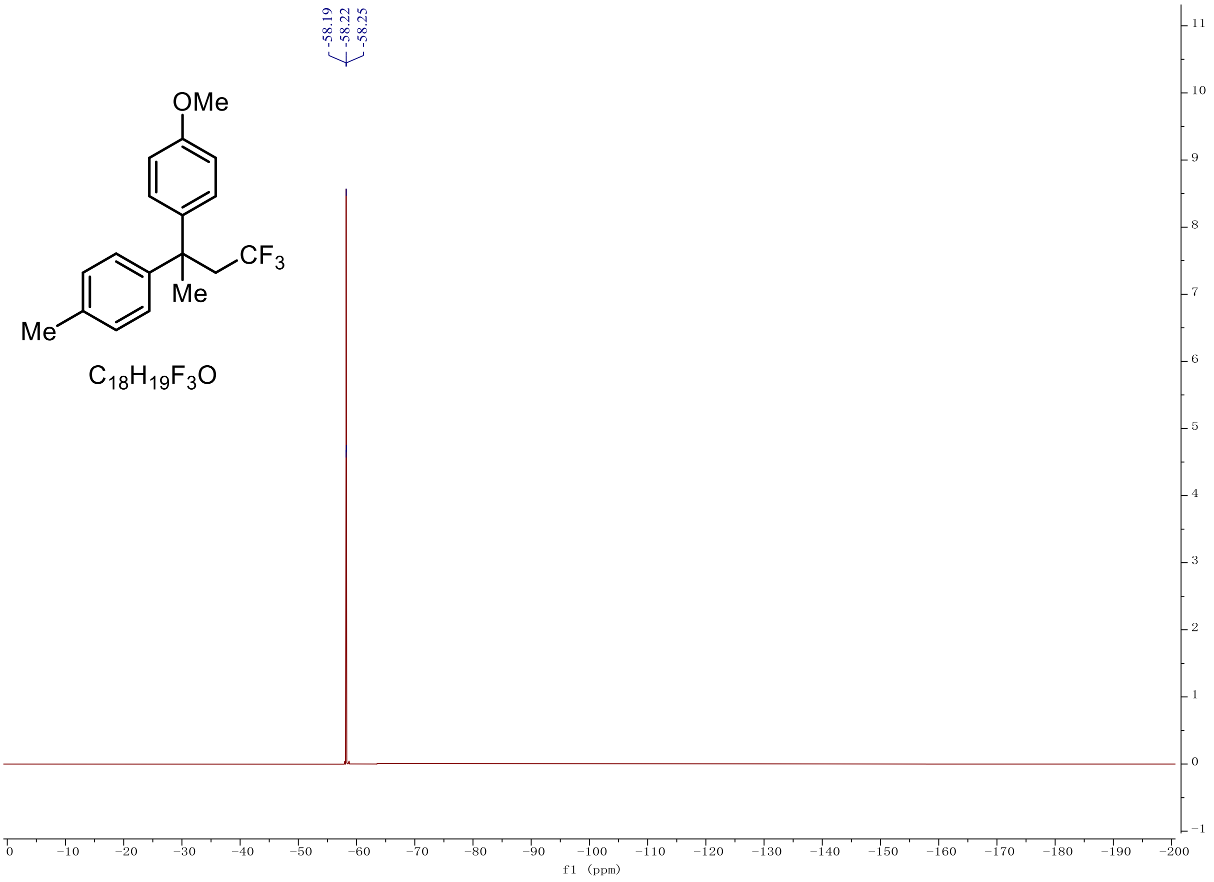

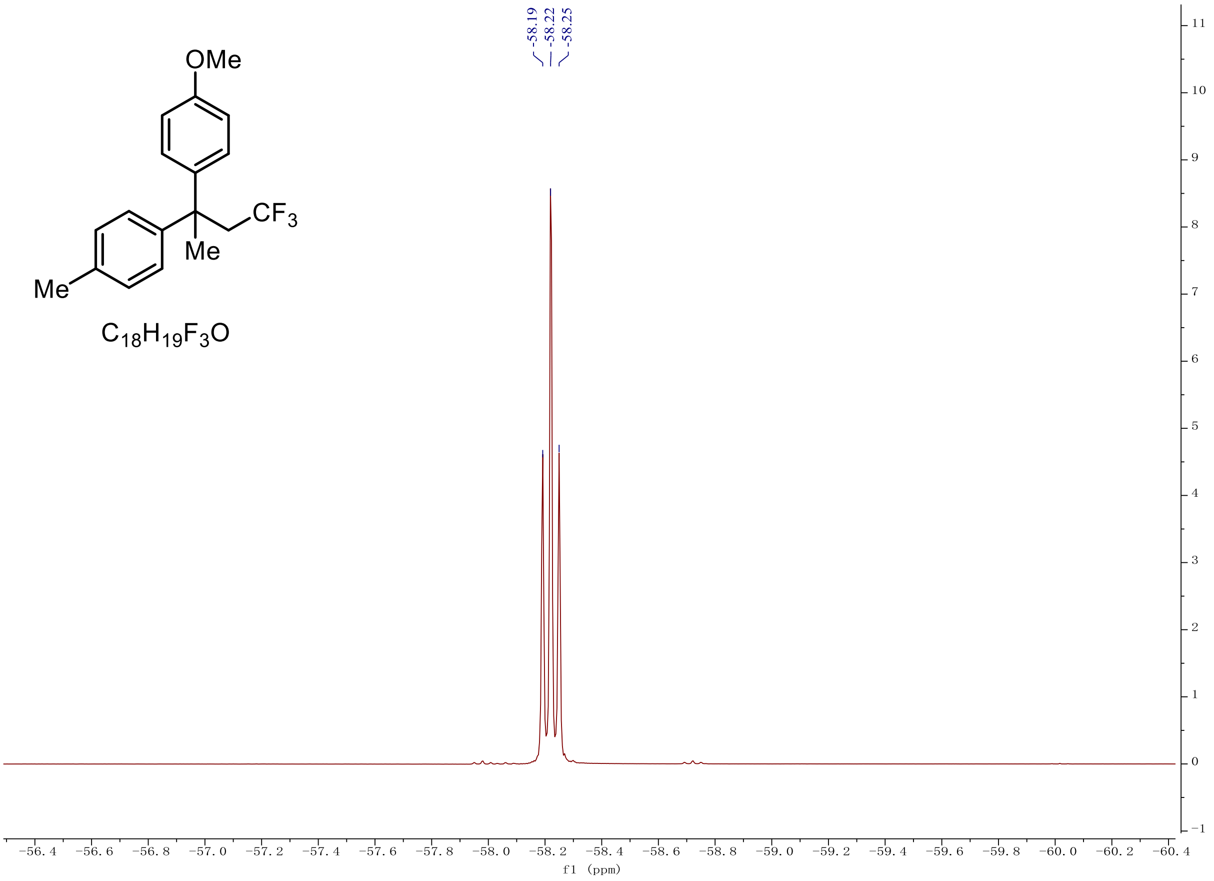

^{1}H NMR (400 MHz, $CDCl_3$) spectrum of 2u

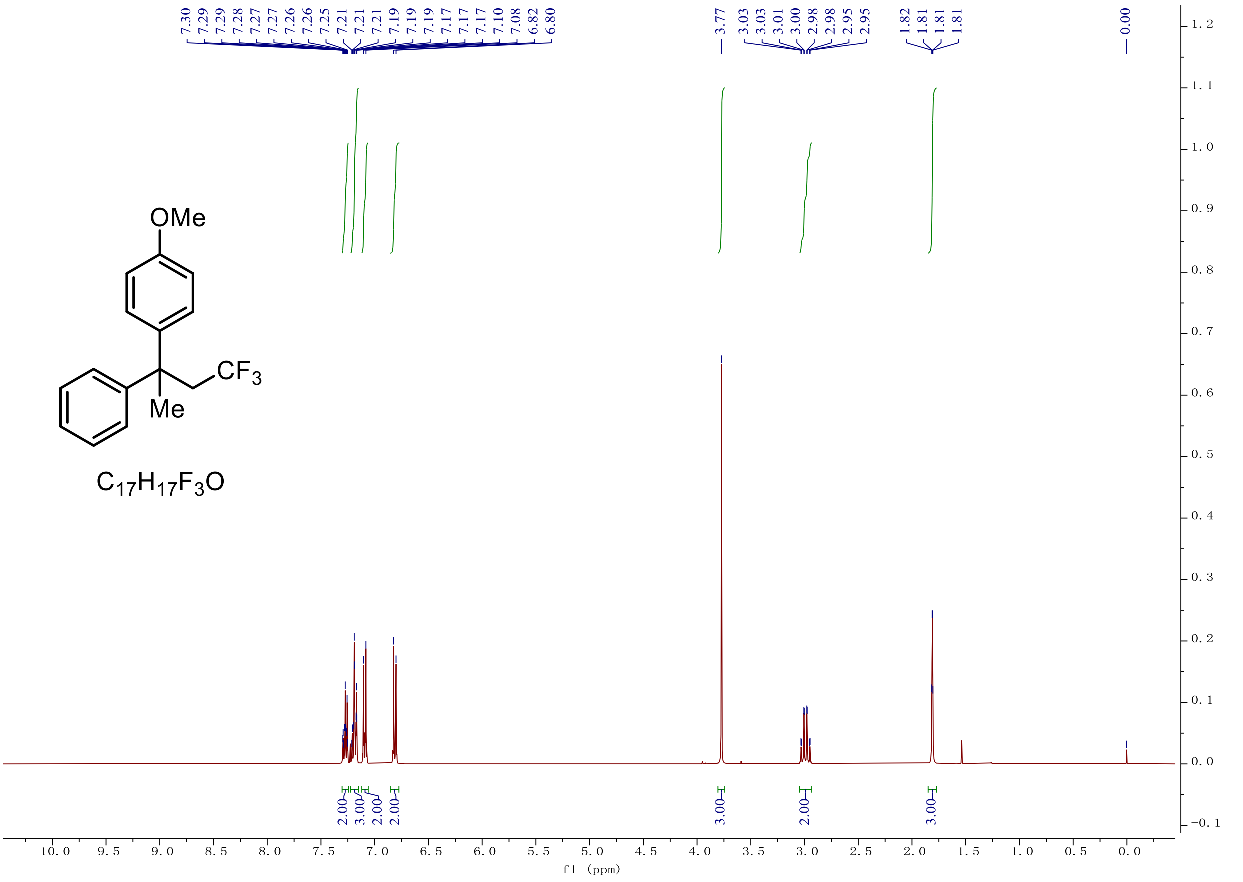

^{13}C NMR (101 MHz, $CDCl_3$) spectrum of 2u

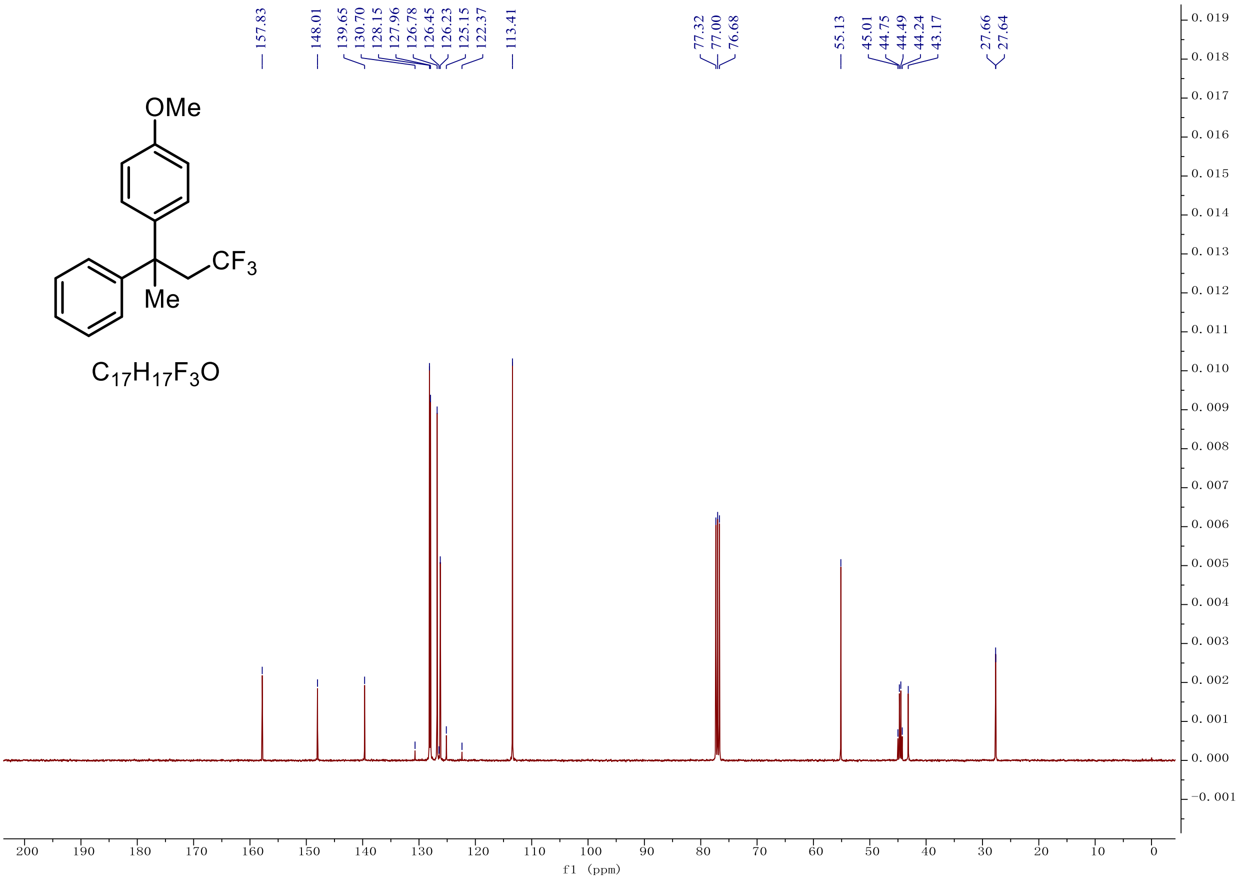

^{19}F NMR (376 MHz, CDCl$_3$) spectrum of 2u

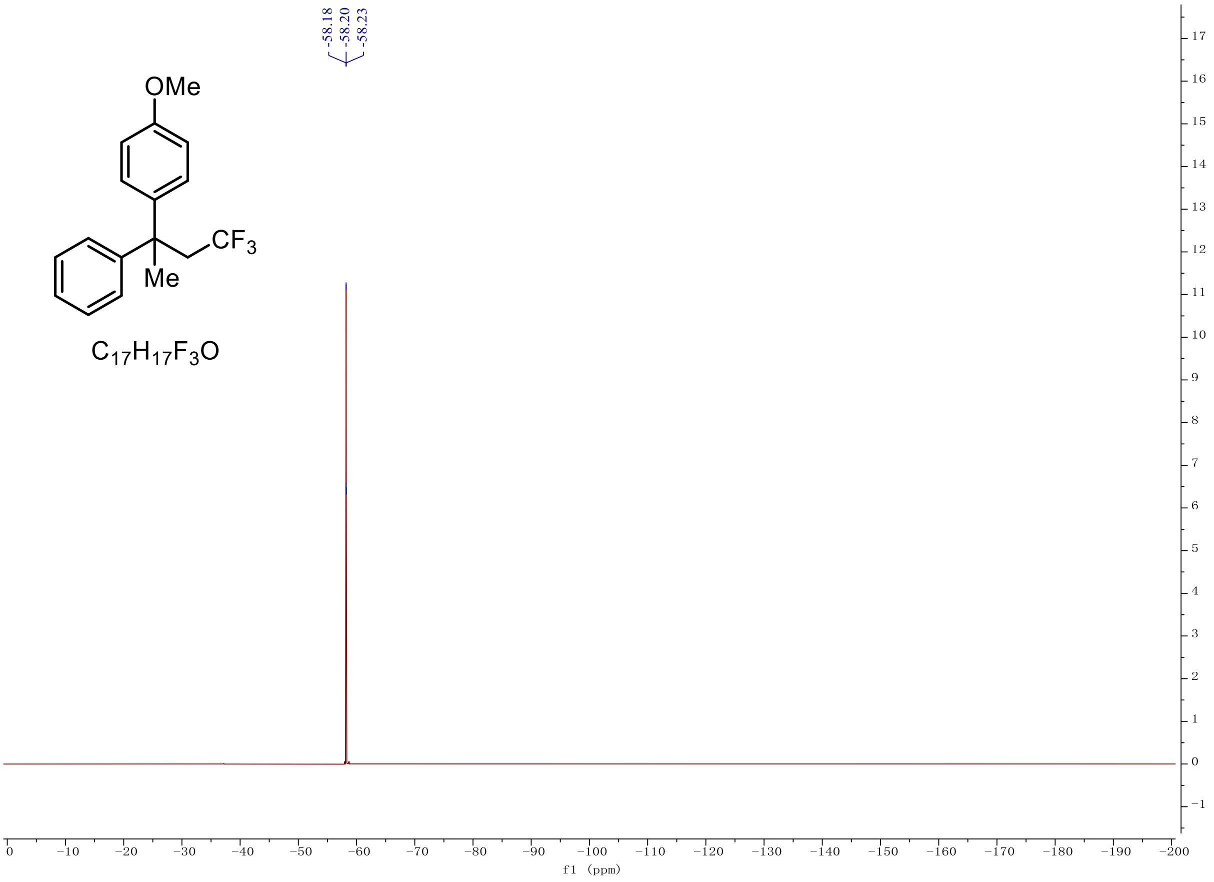

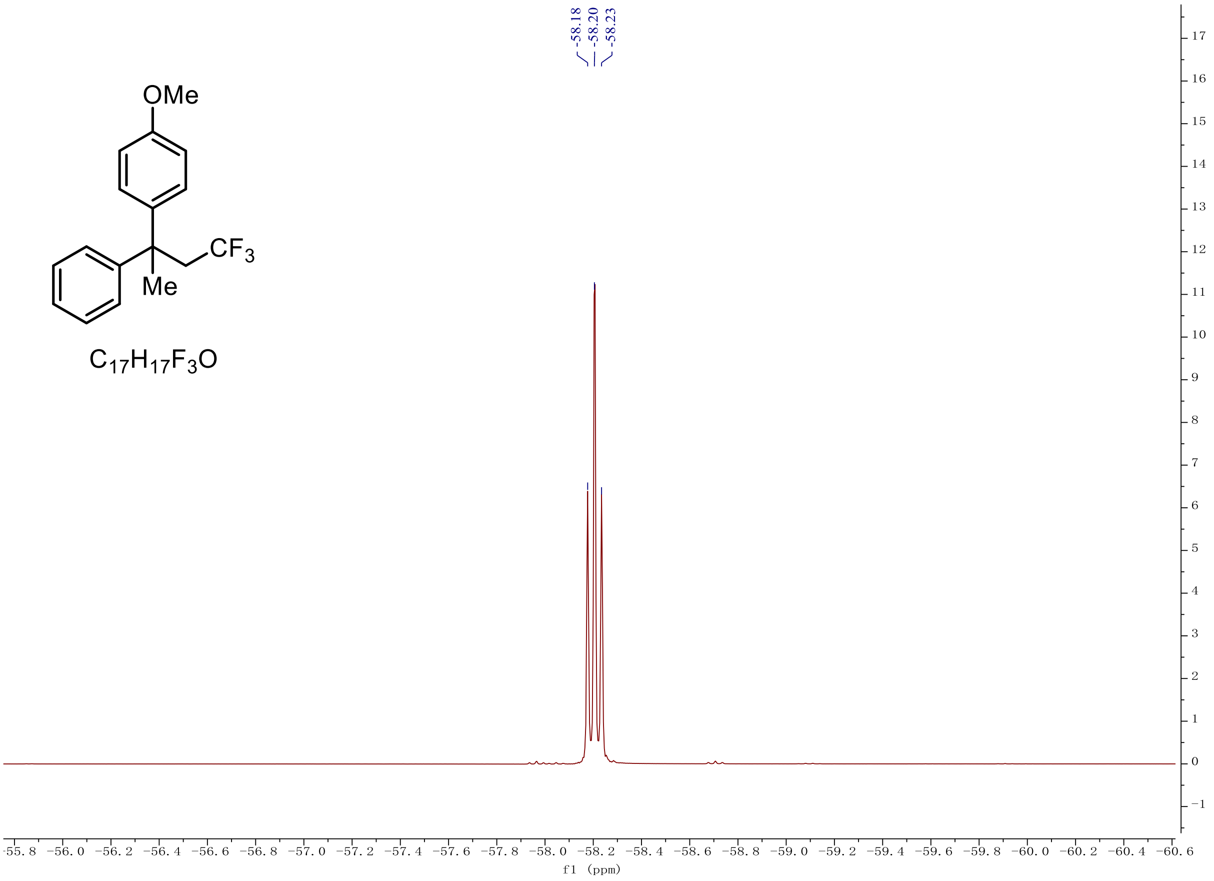

^{1}H NMR (400 MHz, $CDCl_3$) spectrum of 2v

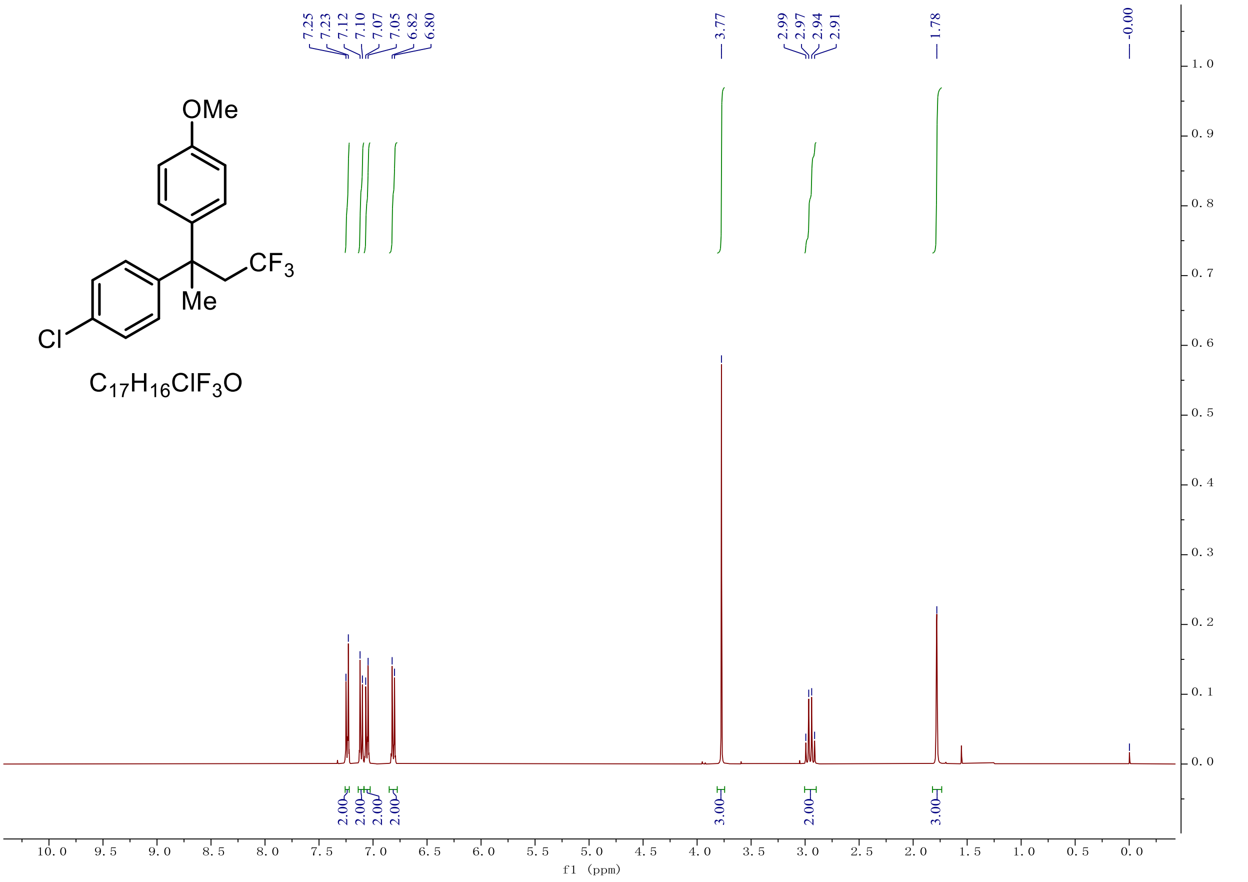

^{13}C NMR (101 MHz, $CDCl_3$) spectrum of 2v

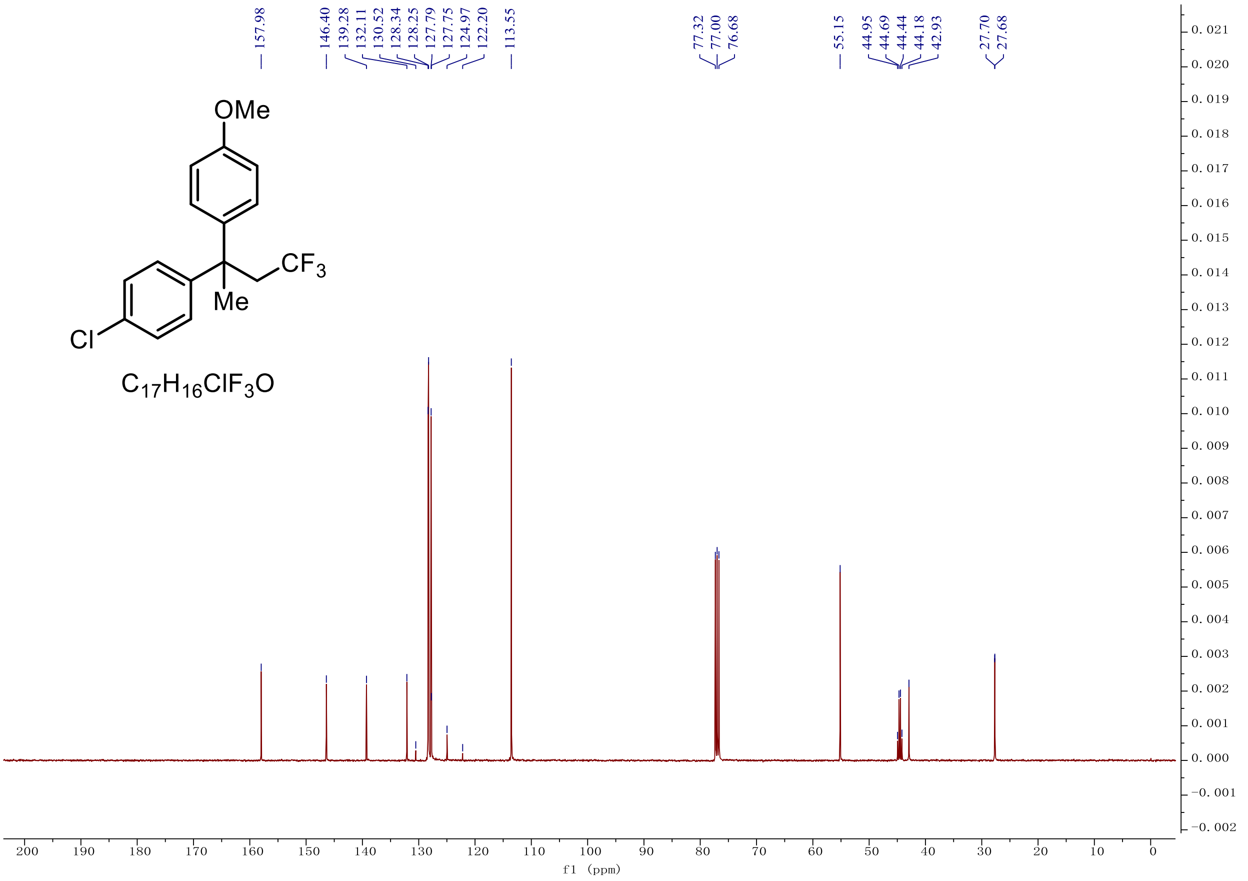

^{19}F NMR (376 MHz, CDCl3) spectrum of 2v

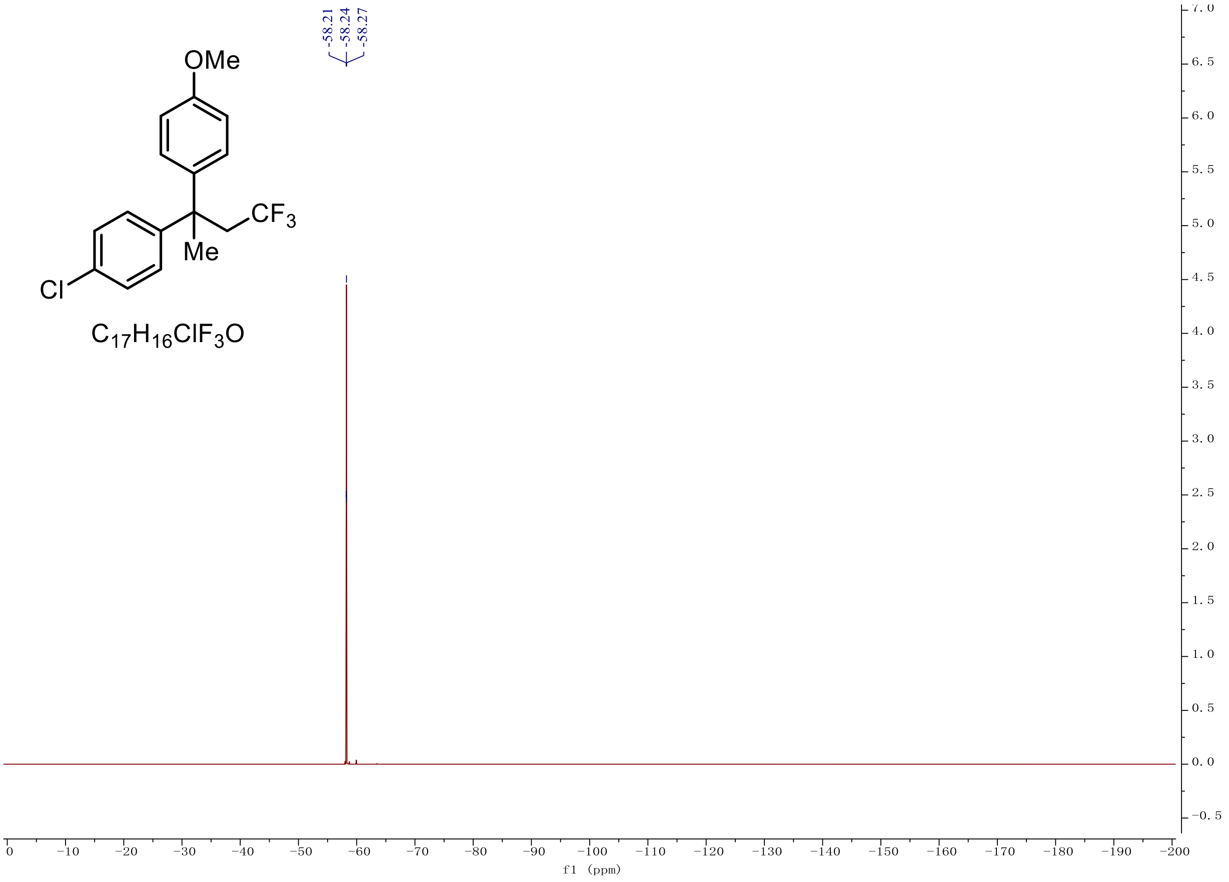

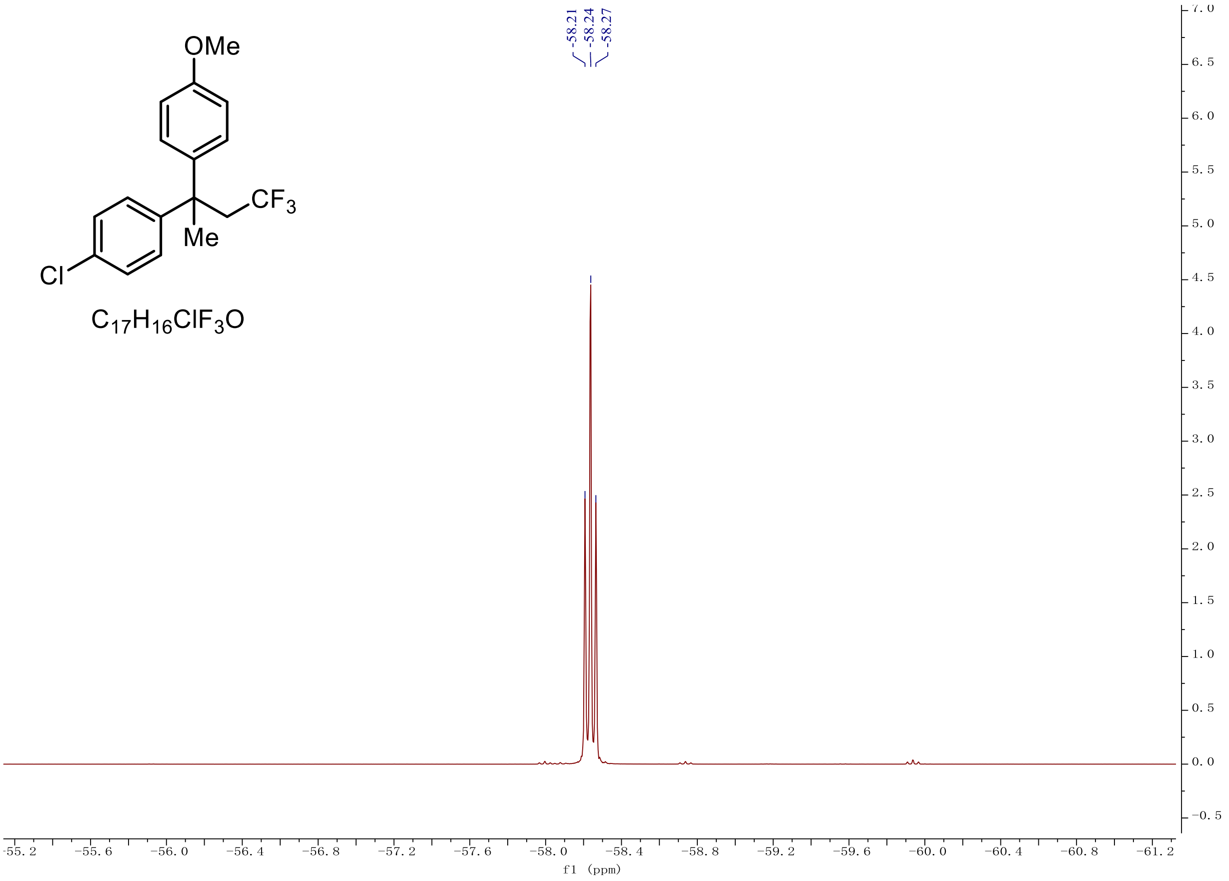

^{1}H NMR (400 MHz, $CDCl_3$) spectrum of 2w

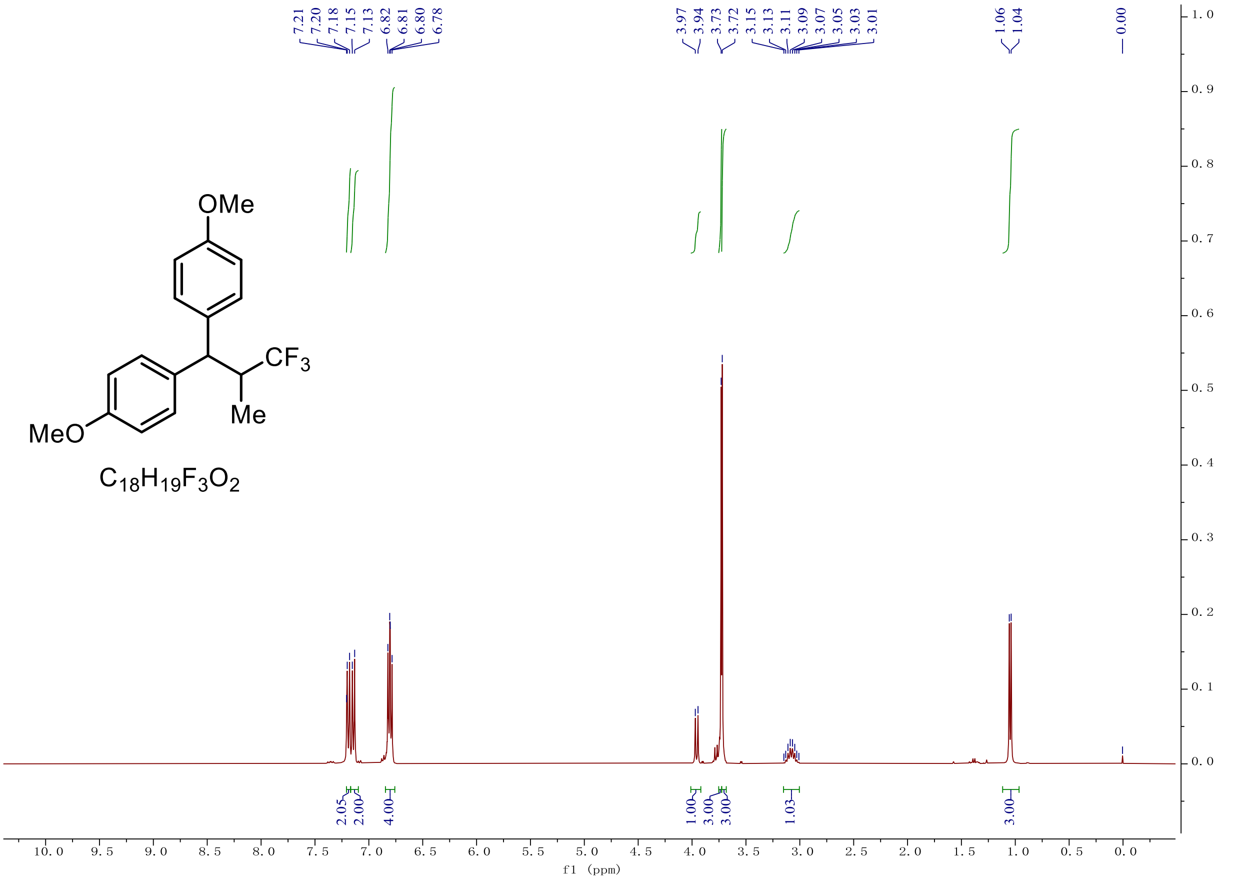

^{13}C NMR (101 MHz, $CDCl_3$) spectrum of 2w

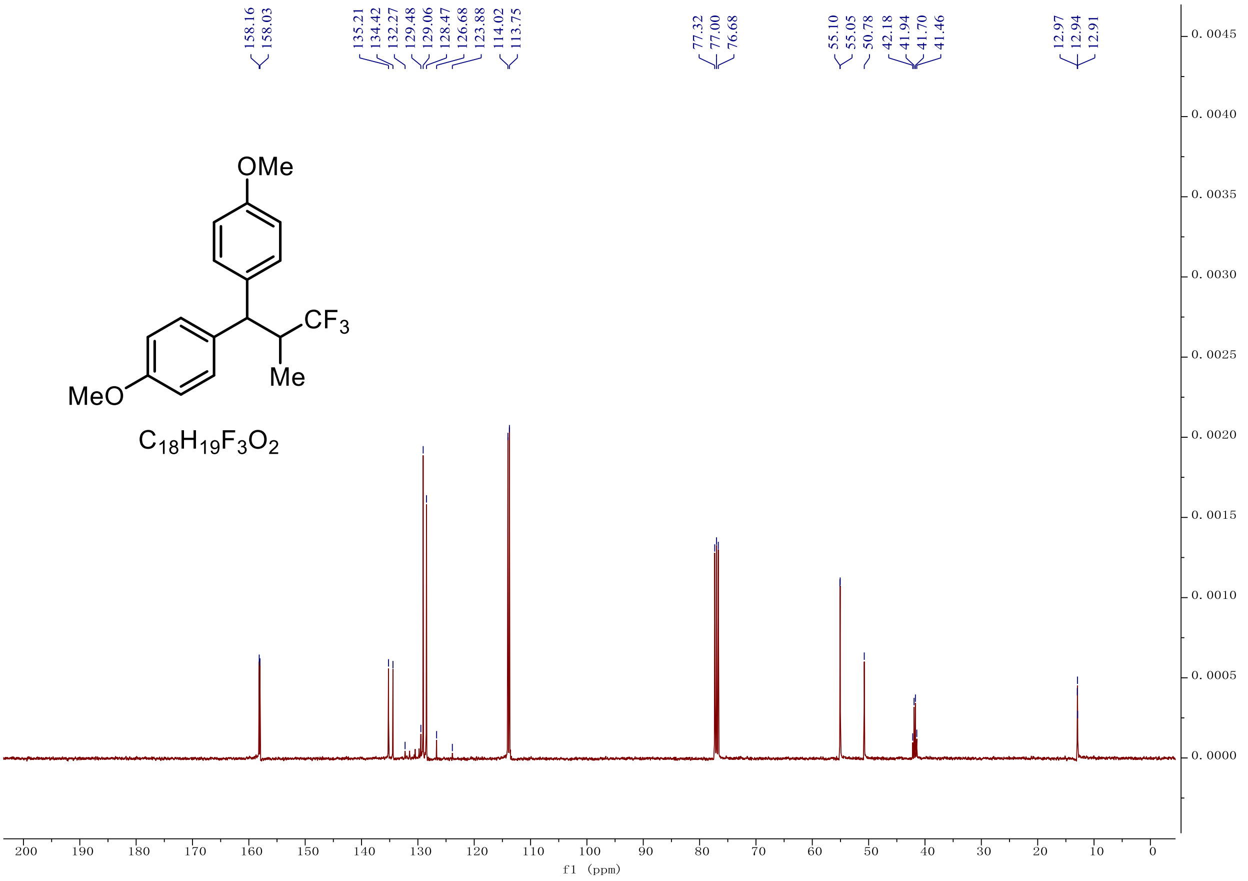

^{19}F NMR (376 MHz, $CDCl_3$) spectrum of 2w

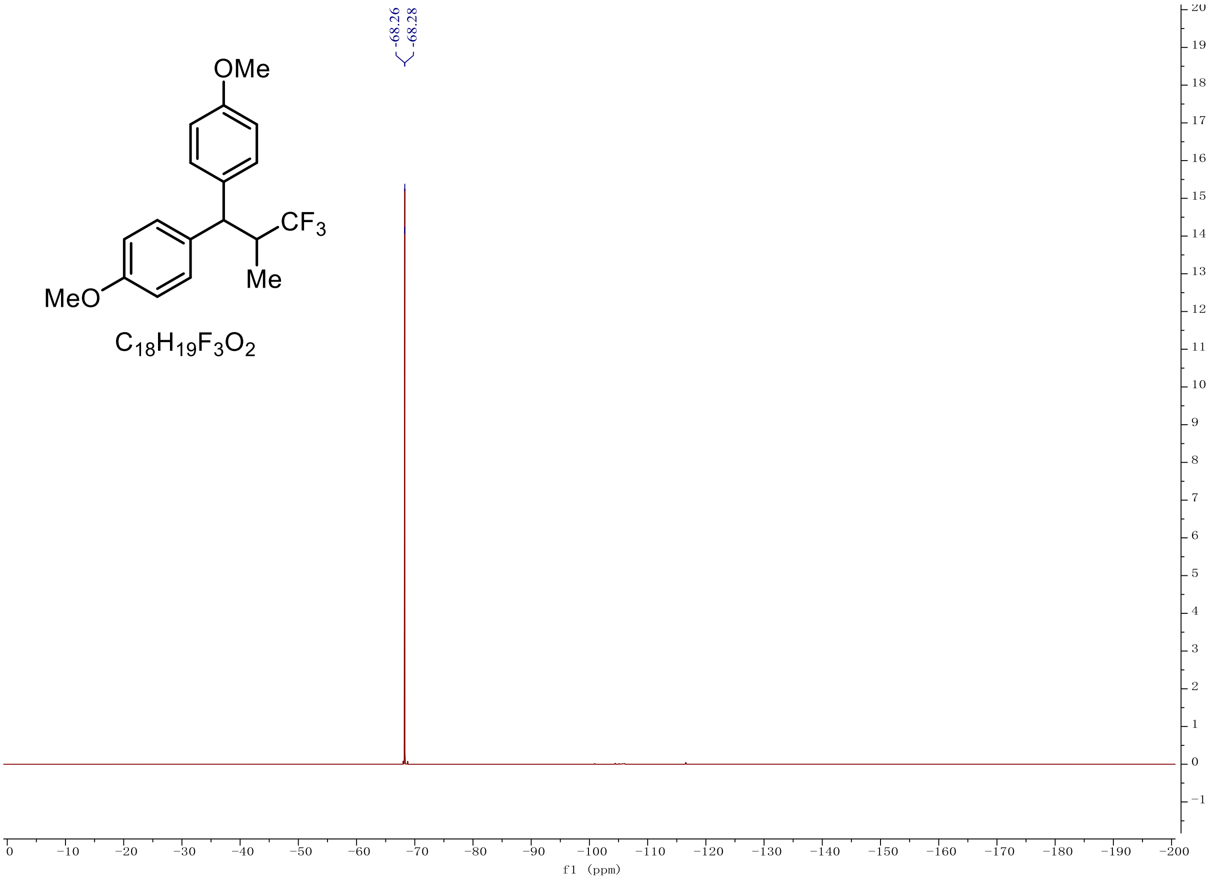

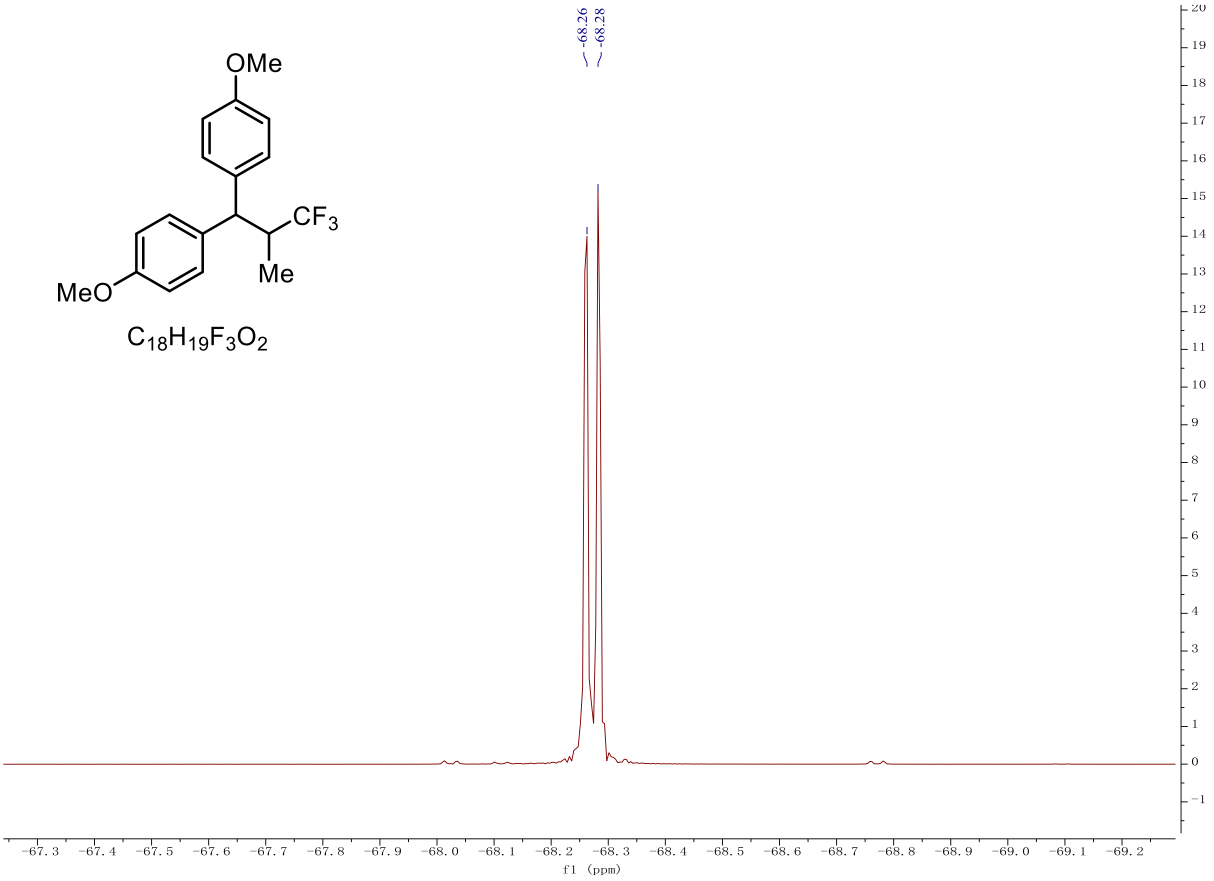

^{1}H NMR (400 MHz, $CDCl_3$) spectrum of 2x

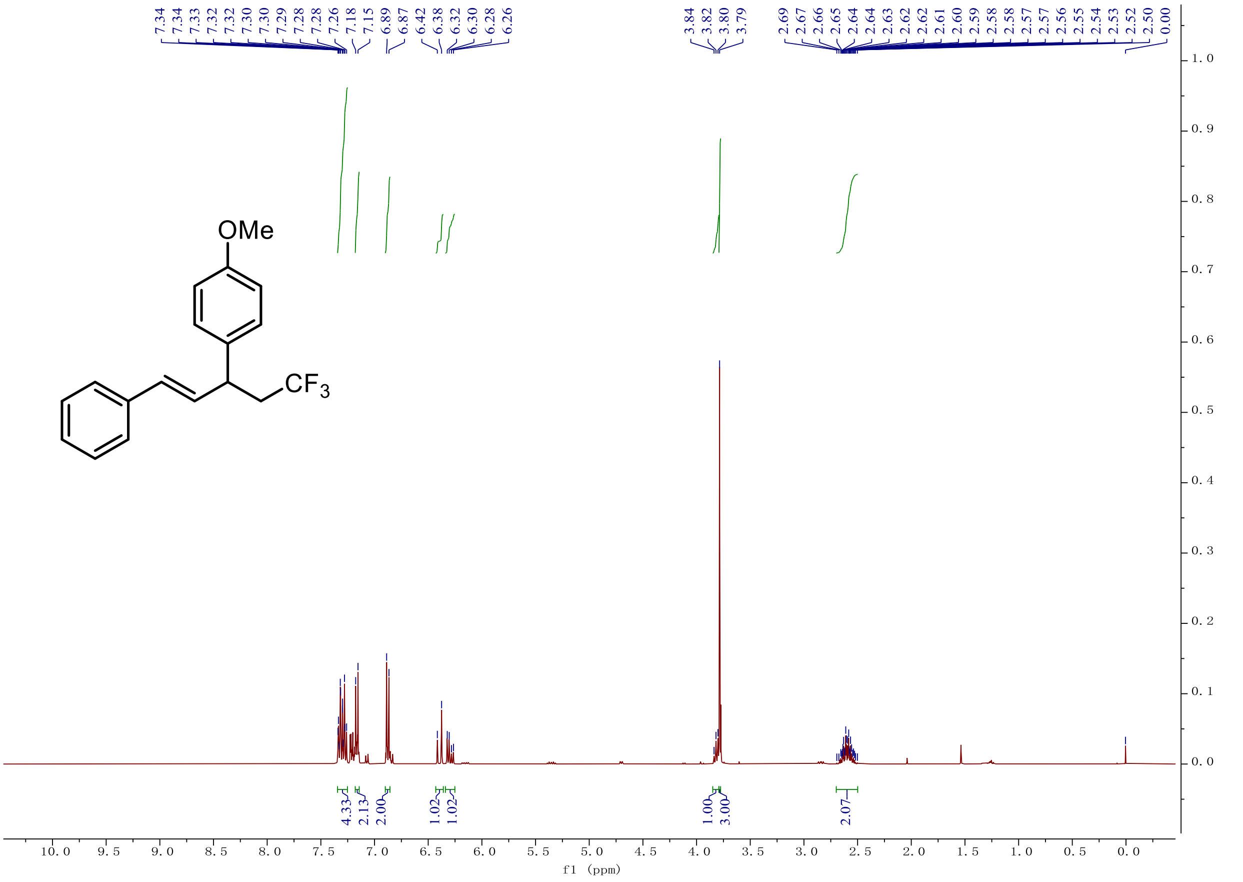

^{13}C NMR (101 MHz, $CDCl_3$) spectrum of 2x

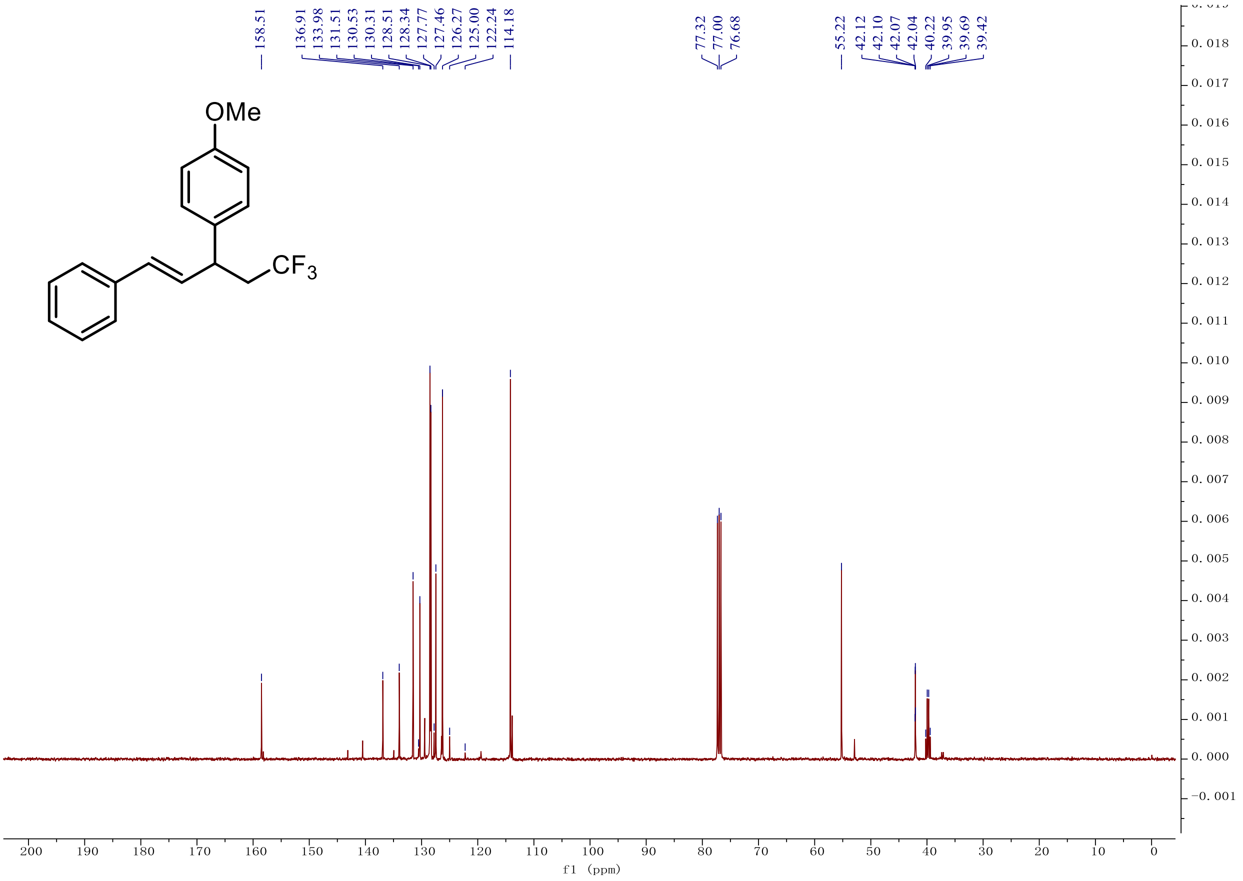

^{19}F NMR (376 MHz, $CDCl_3$) spectrum of 2x

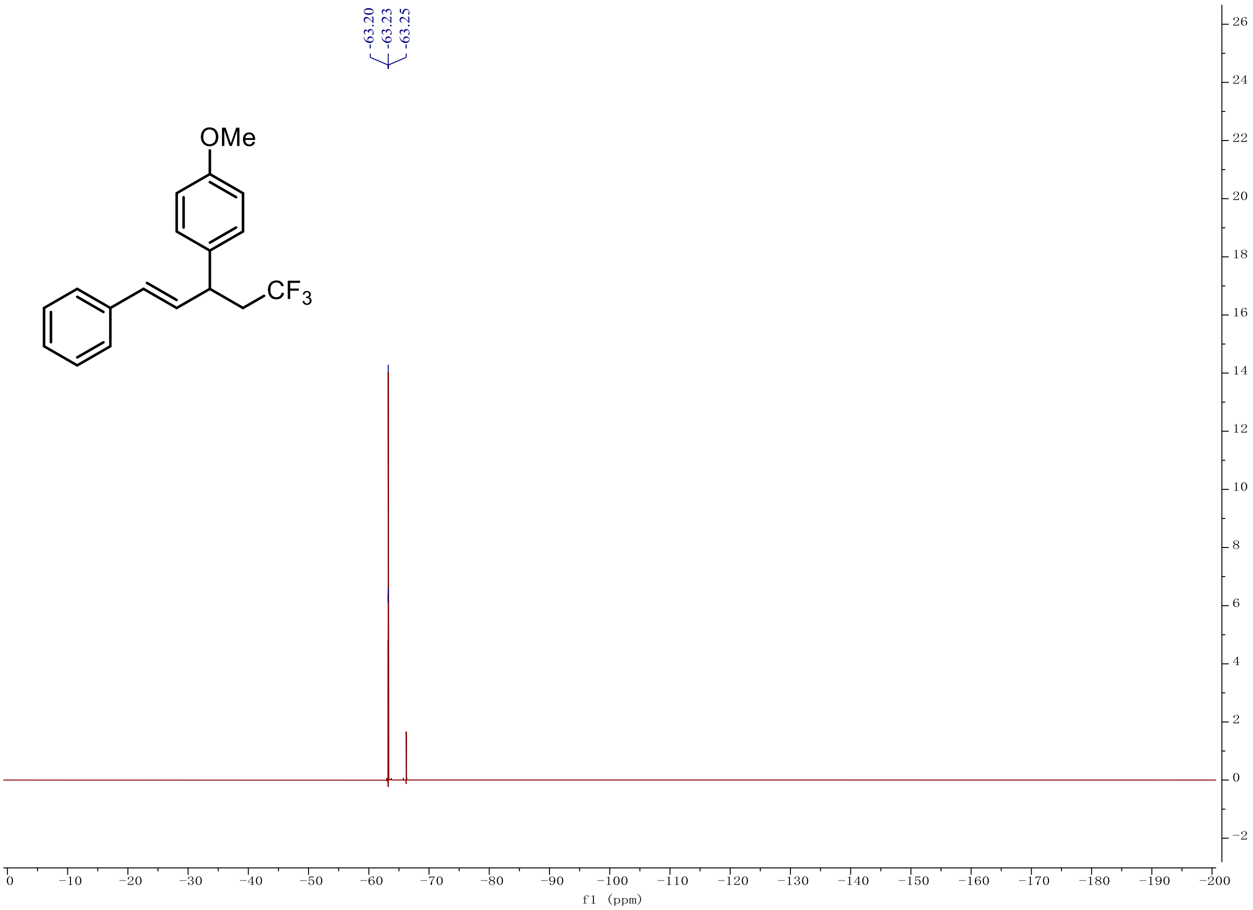

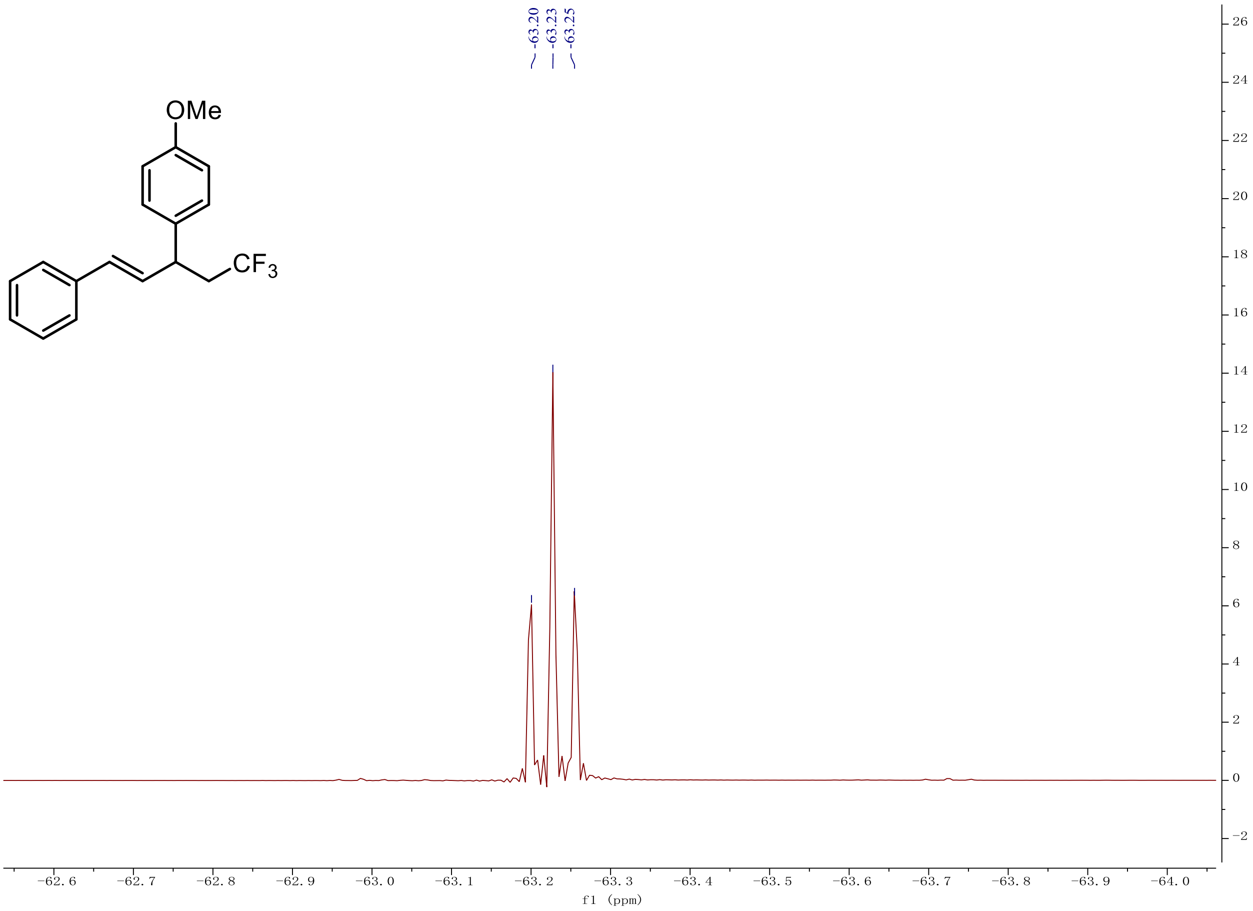

^{1}H NMR (400 MHz, $CDCl_3$) spectrum of 4a

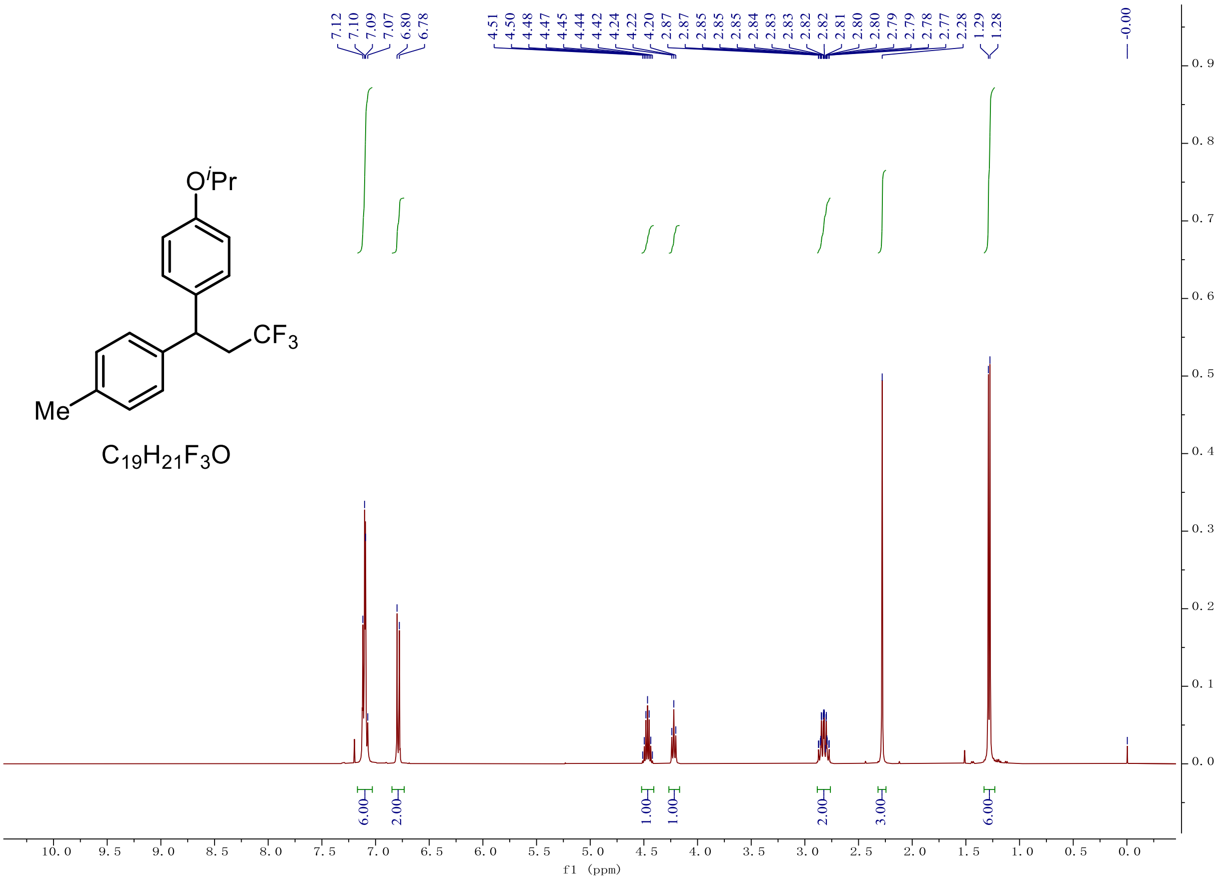

^{13}C NMR (101 MHz, $CDCl_3$) spectrum of 4a

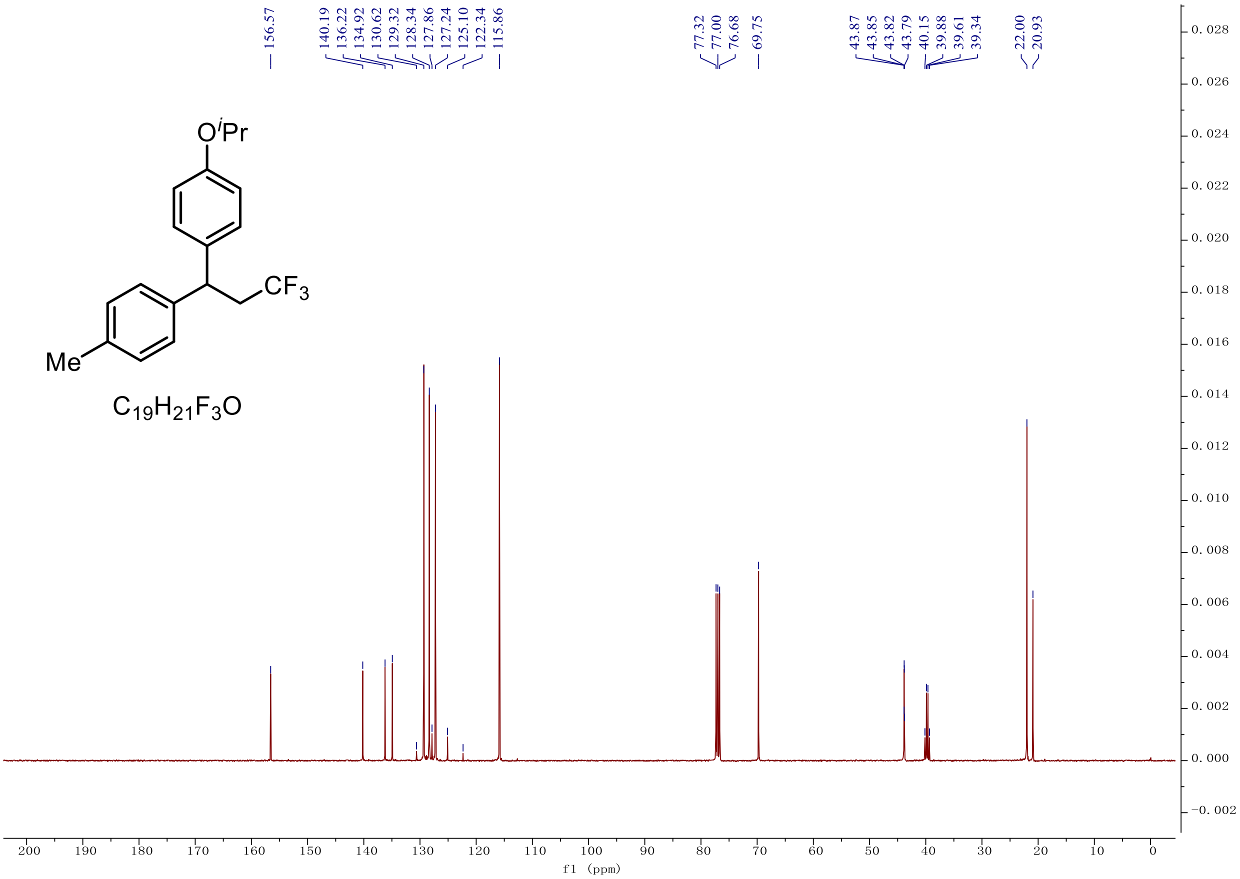

^{19}F NMR (376 MHz, $CDCl_3$) spectrum of 4a

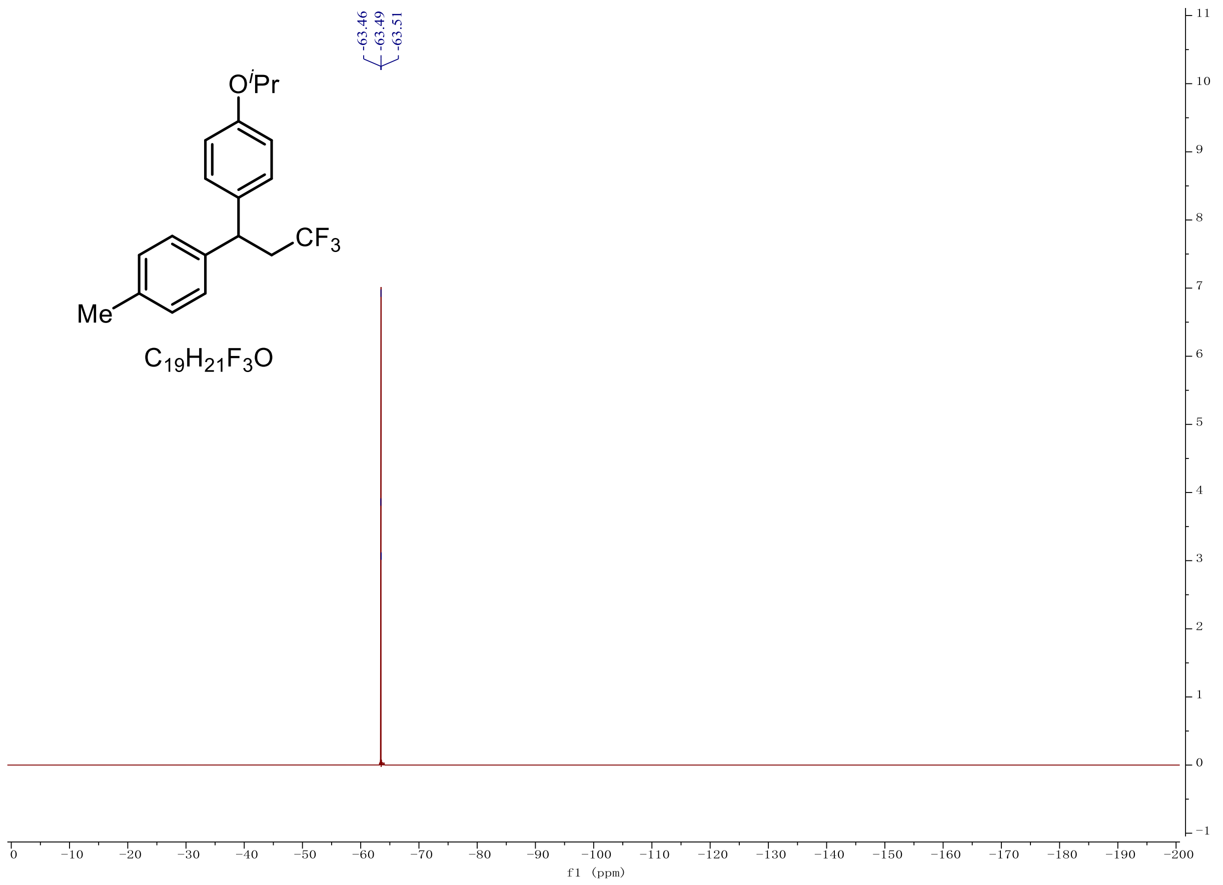

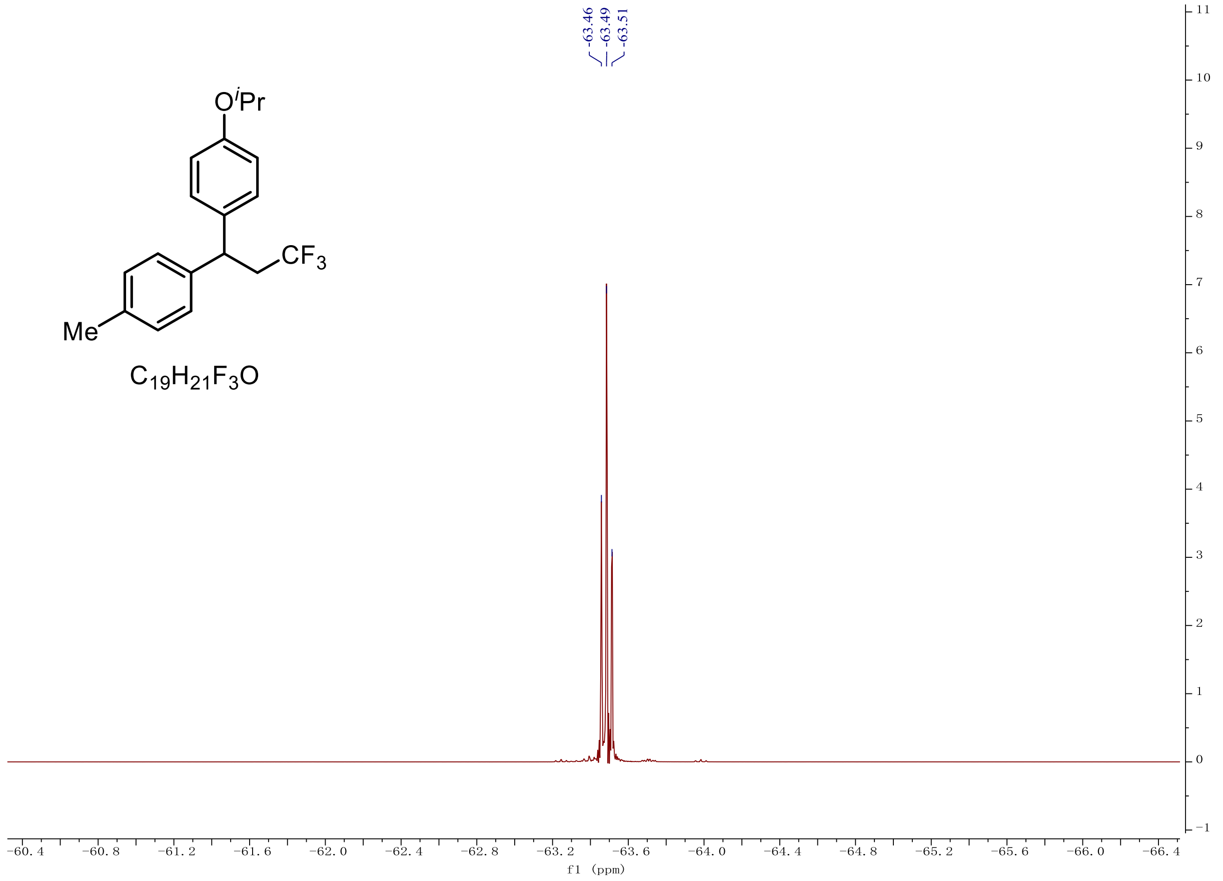

^{1}H NMR (400 MHz, CDCl$_3$) spectrum of 4b

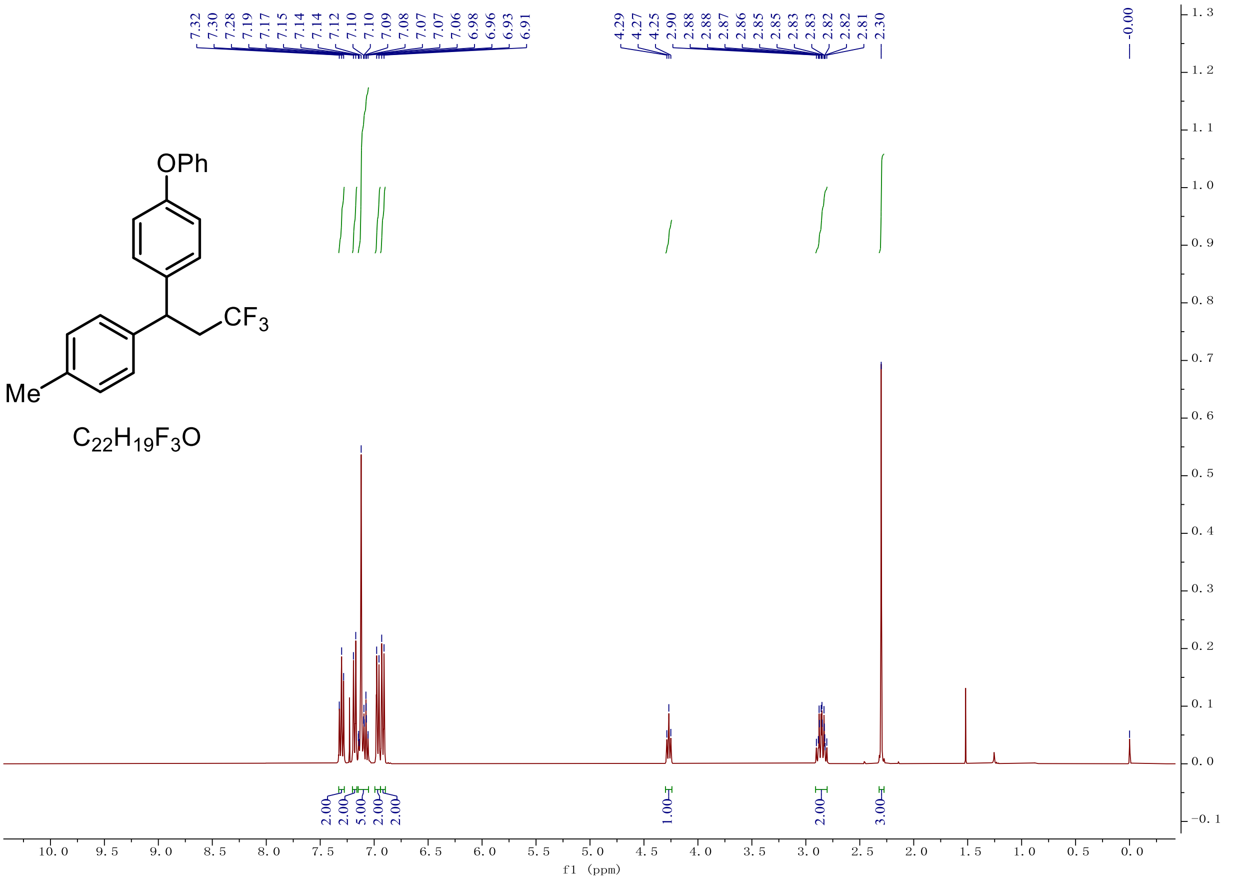

^{13}C NMR (101 MHz, CDCl$_3$) spectrum of 4b

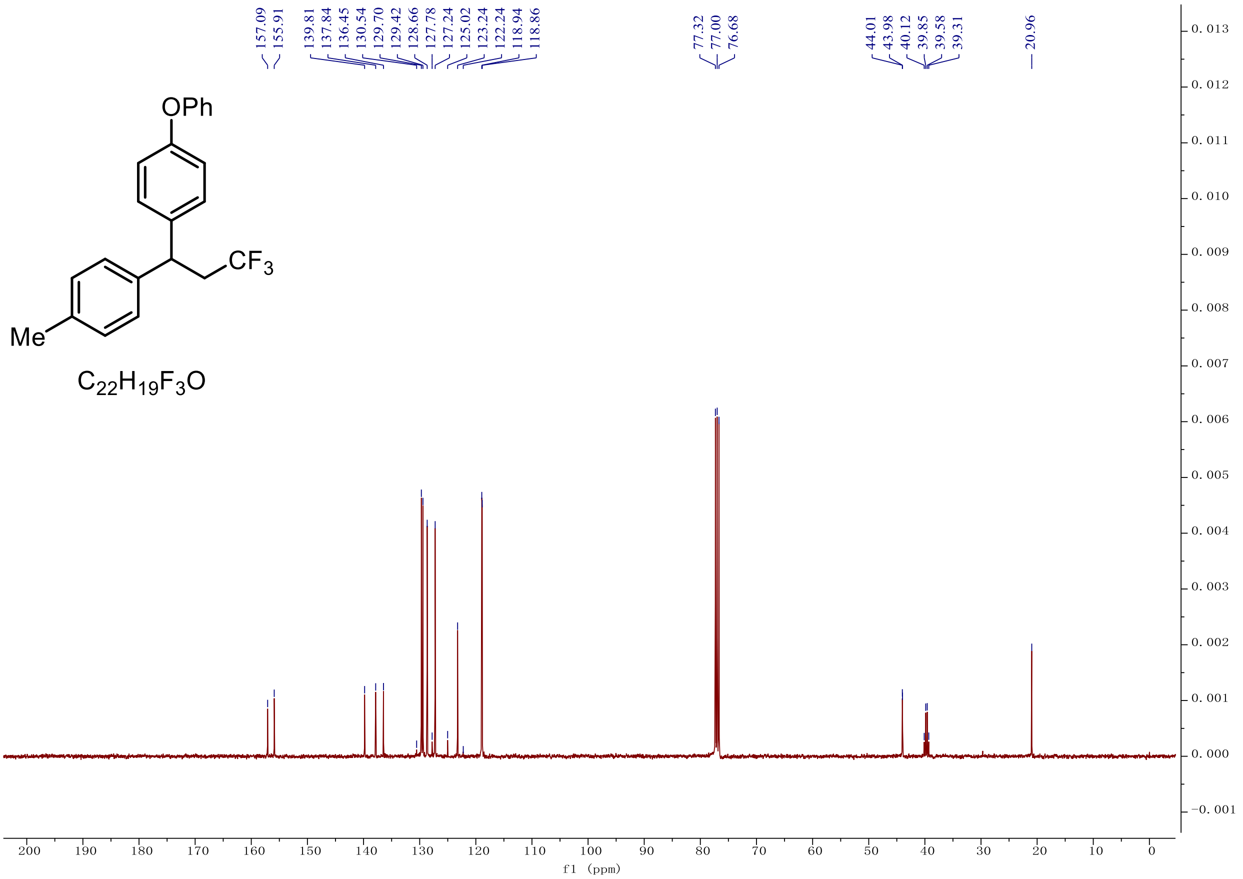

^{19}F NMR (376 MHz, CDCl3) spectrum of 4b

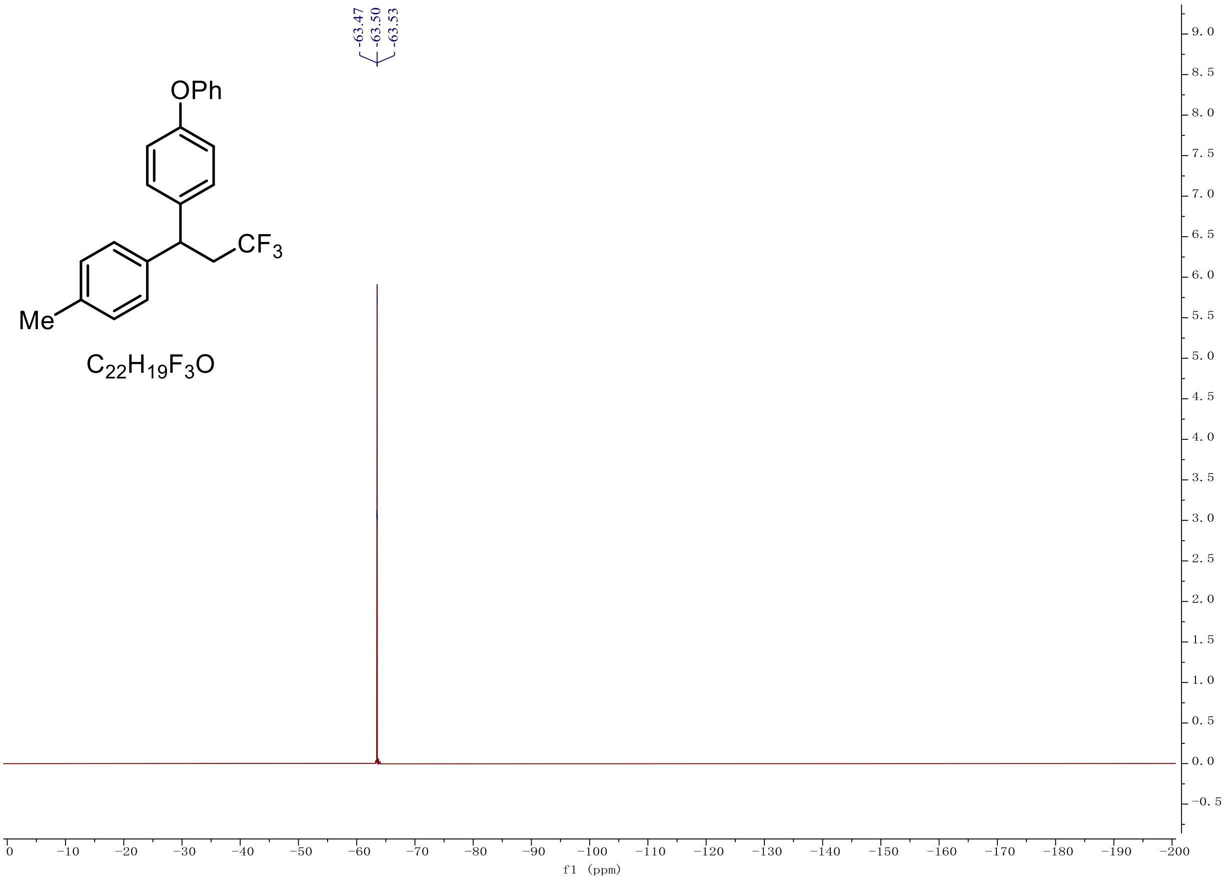

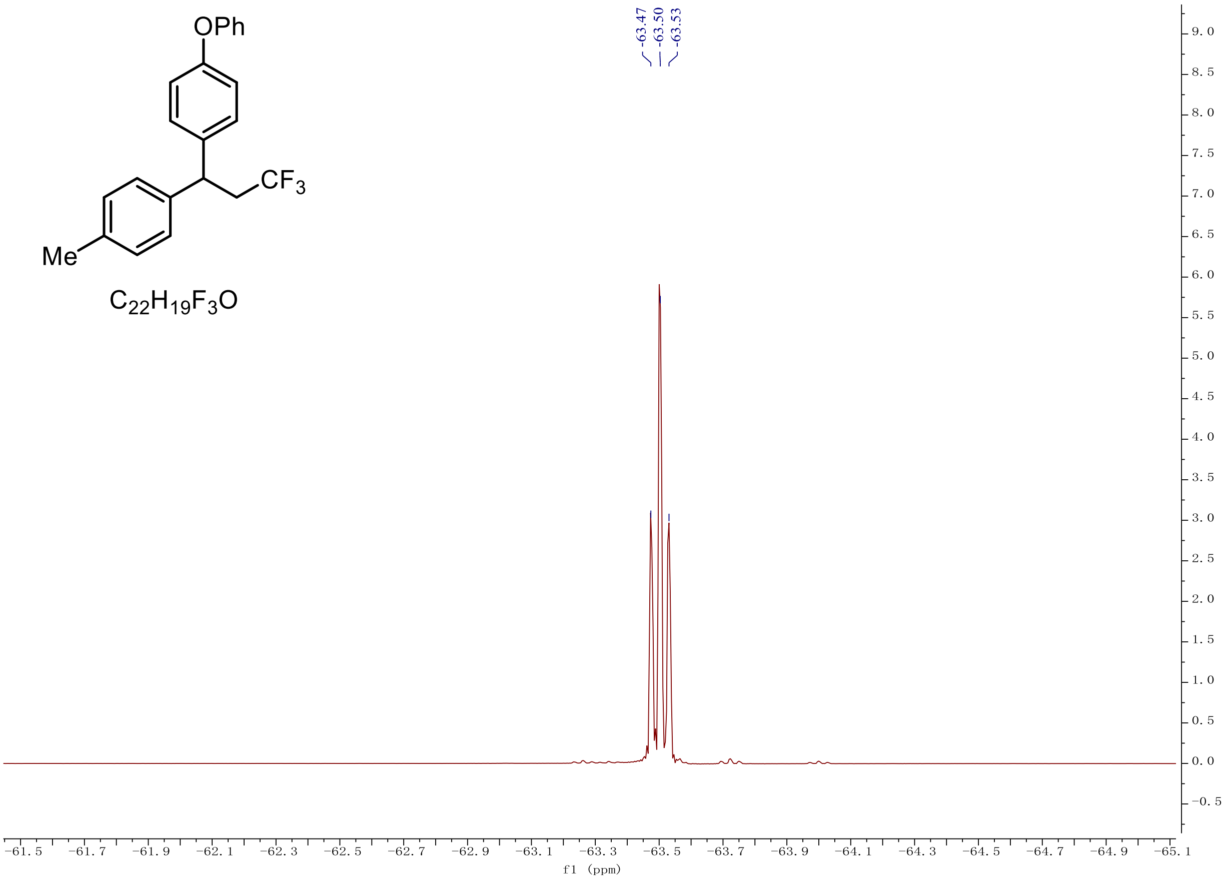

^{1}H NMR (400 MHz, $CDCl_3$) spectrum of 4c

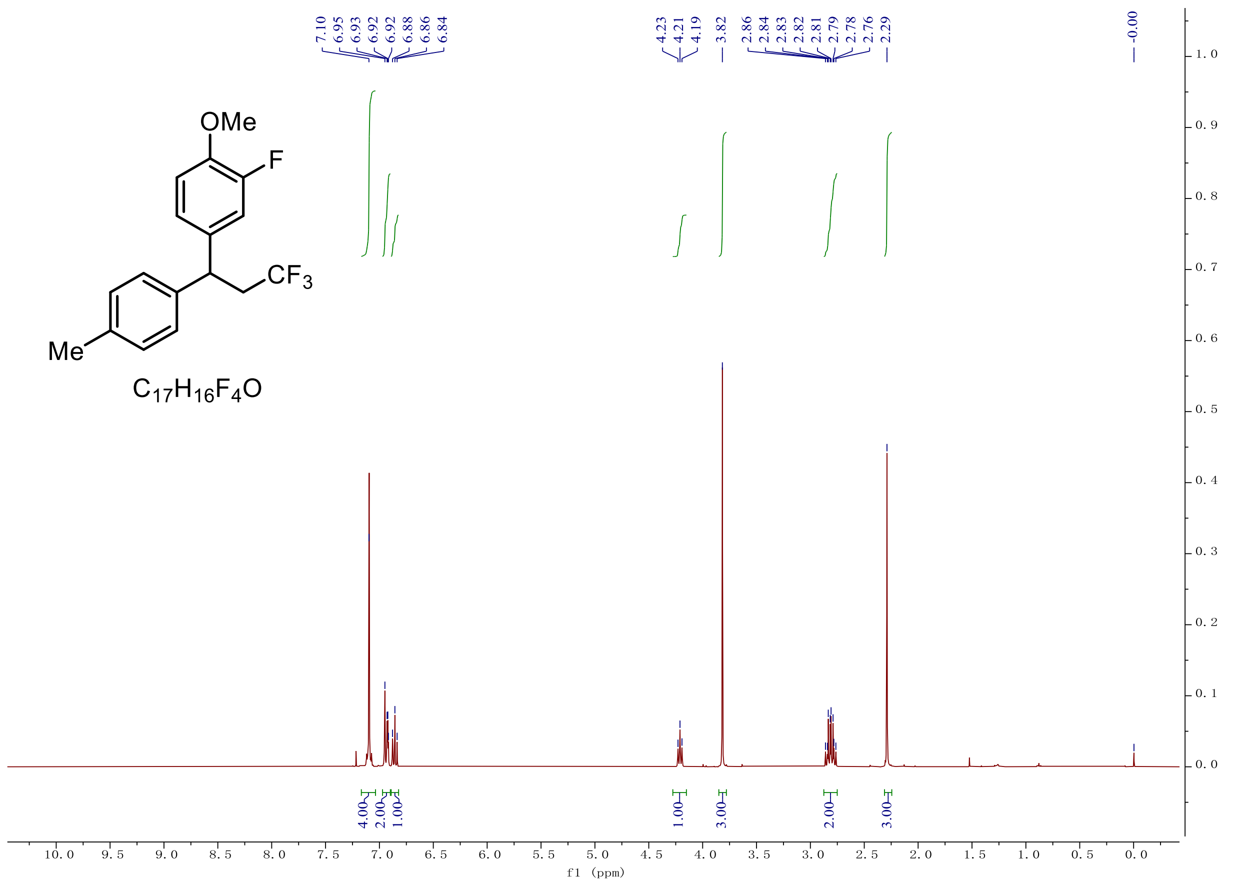

^{13}C NMR (101 MHz, $CDCl_3$) spectrum of 4c

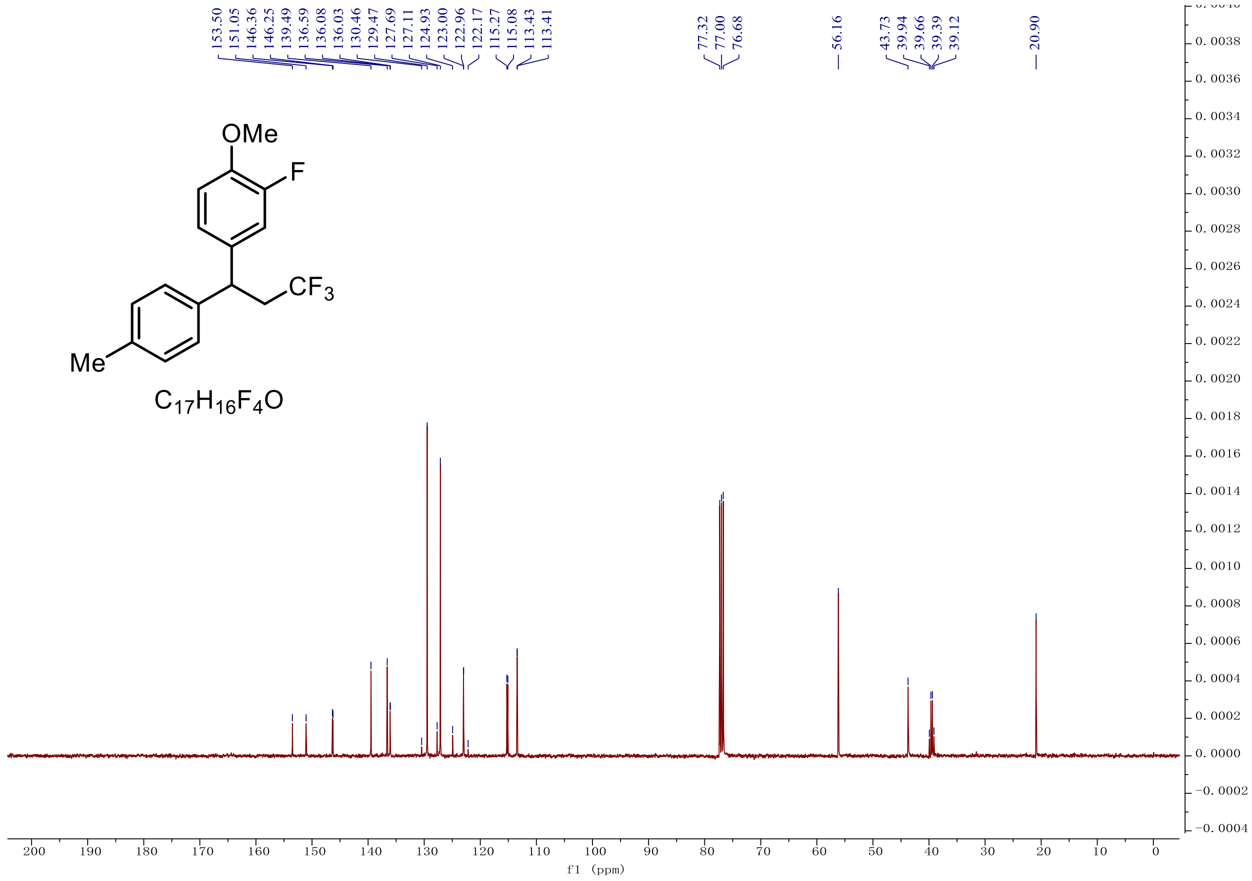

^{19}F NMR (376 MHz, CDCl3) spectrum of 4c

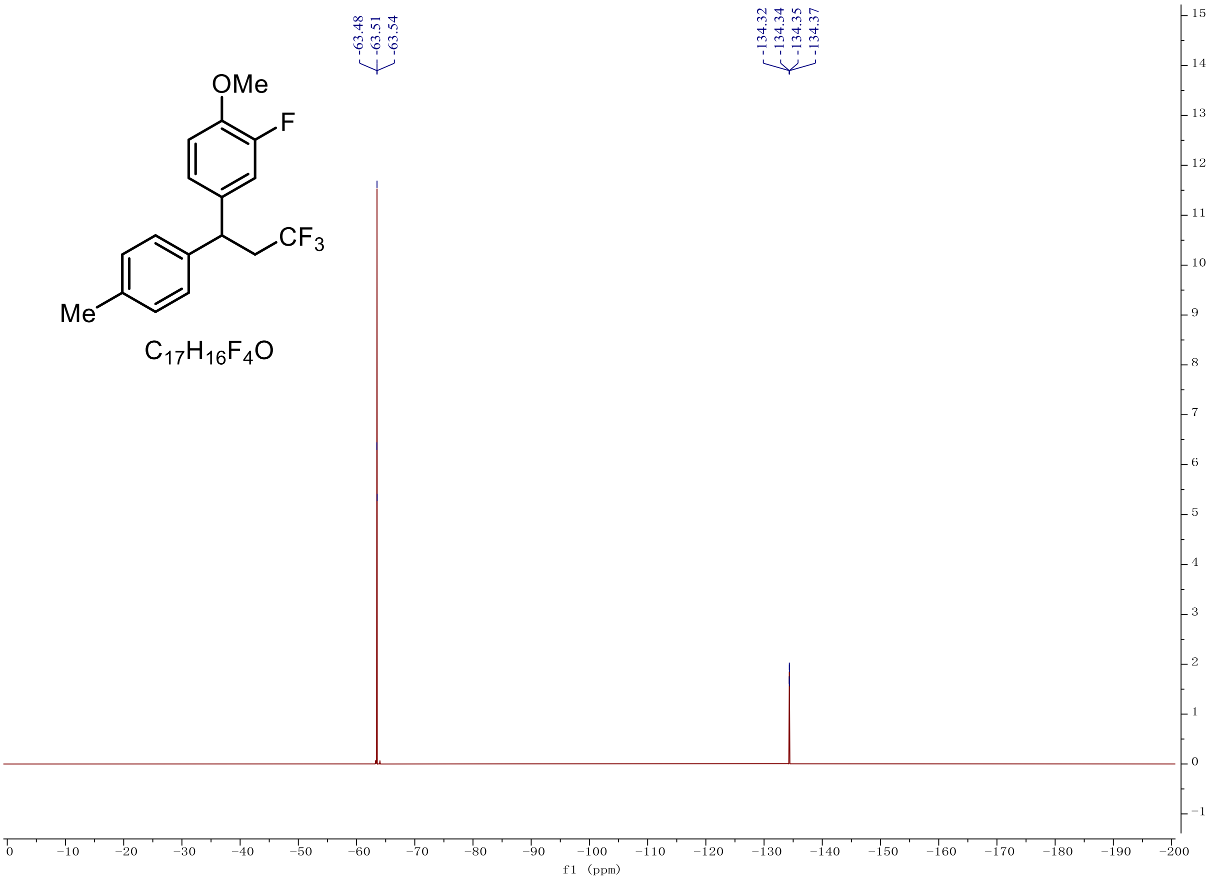

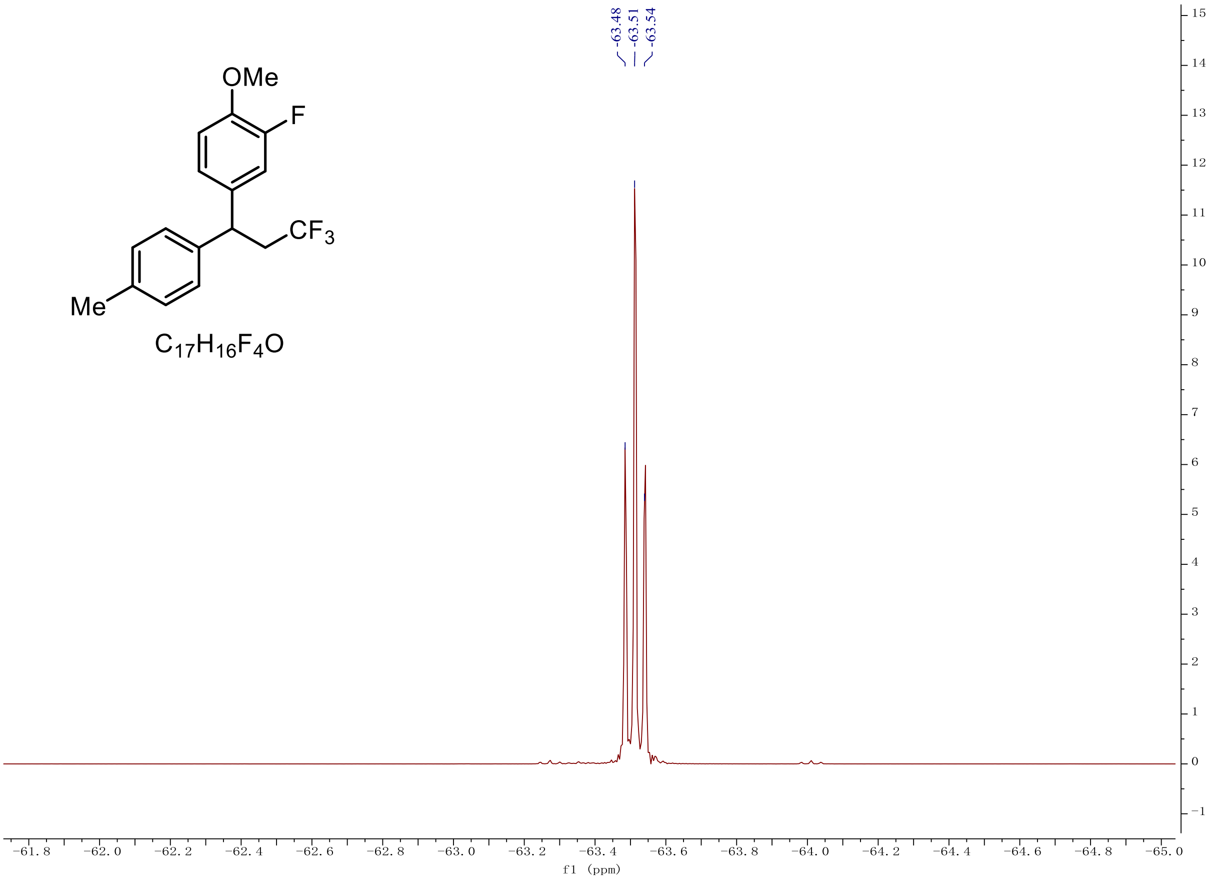

^{1}H NMR (400 MHz, $CDCl_3$) spectrum of 4d

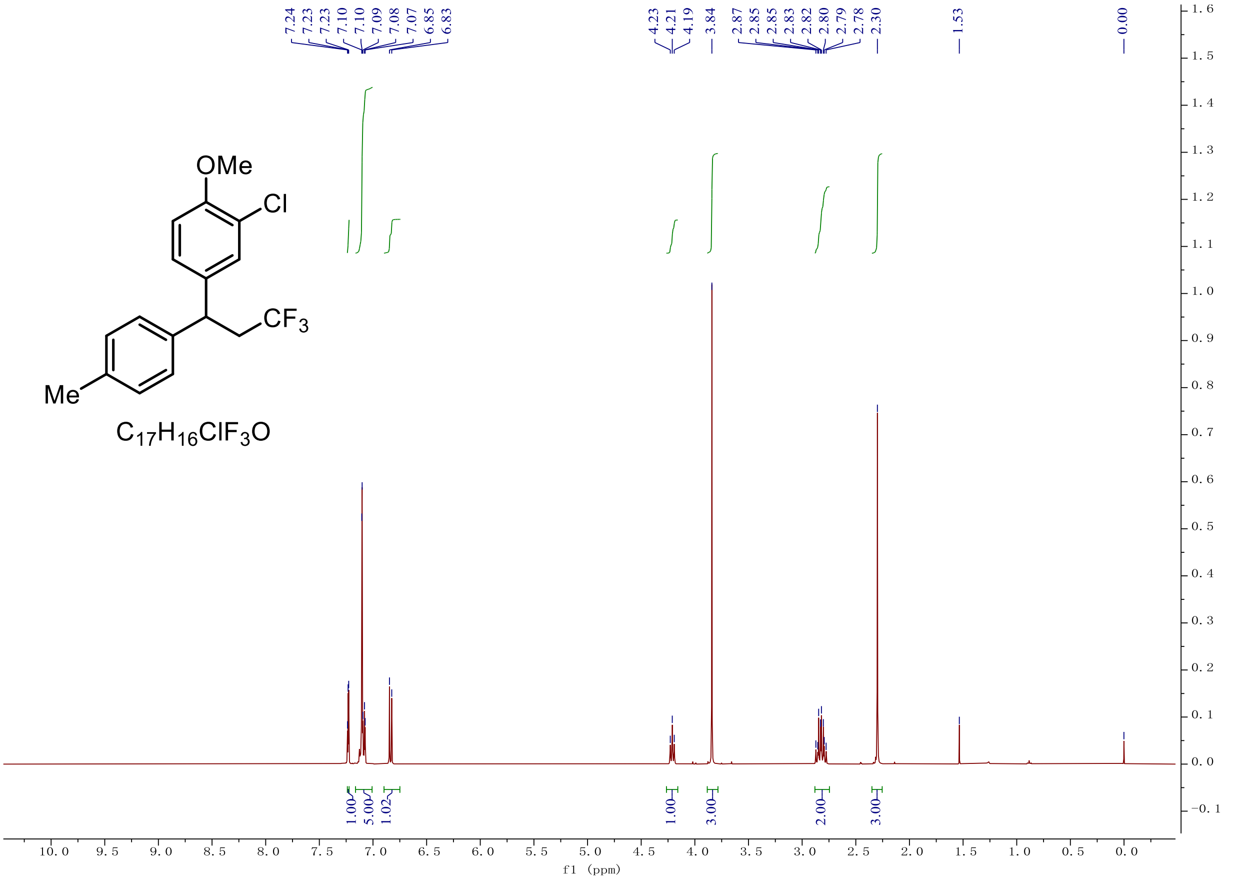

^{13}C NMR (101 MHz, $CDCl_3$) spectrum of 4d

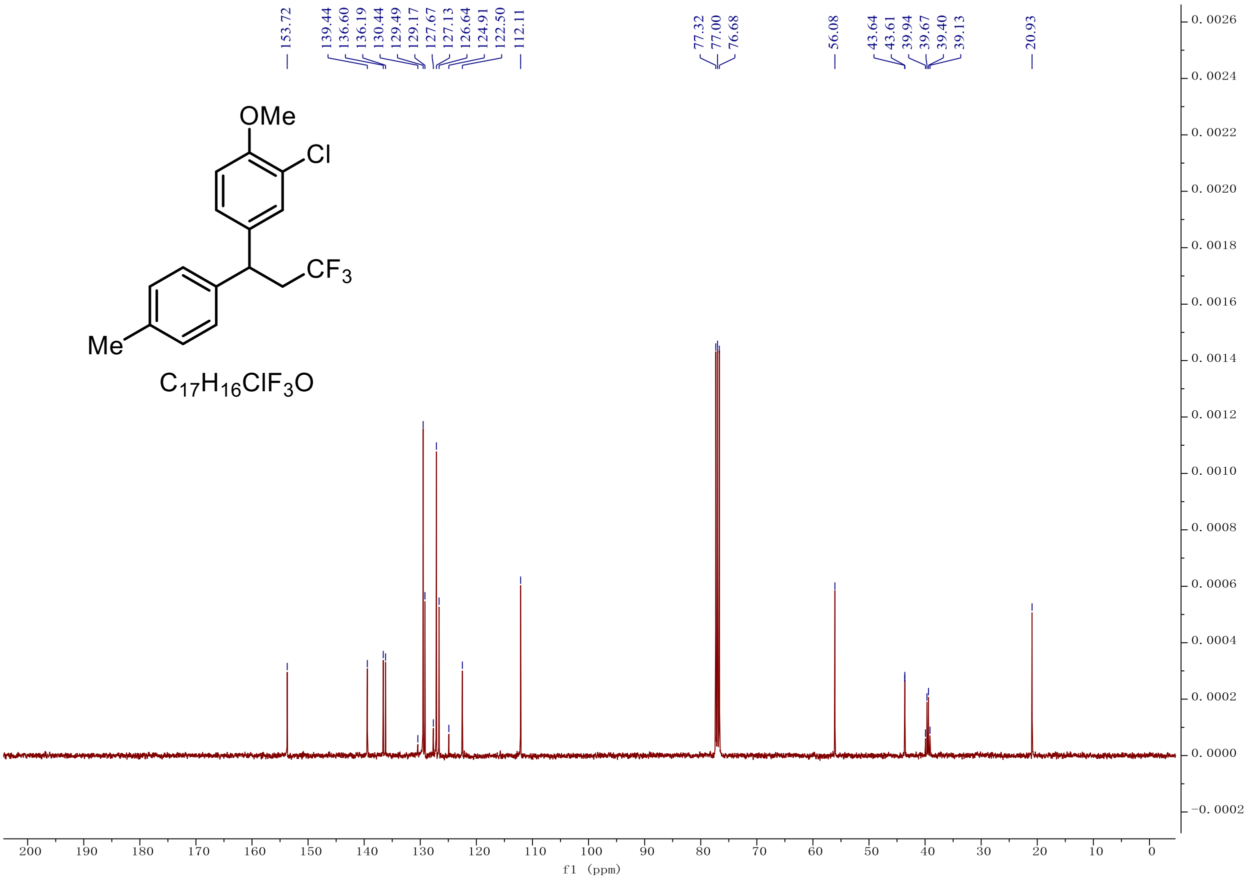

^{19}F NMR (376 MHz, CDCl3) spectrum of 4d

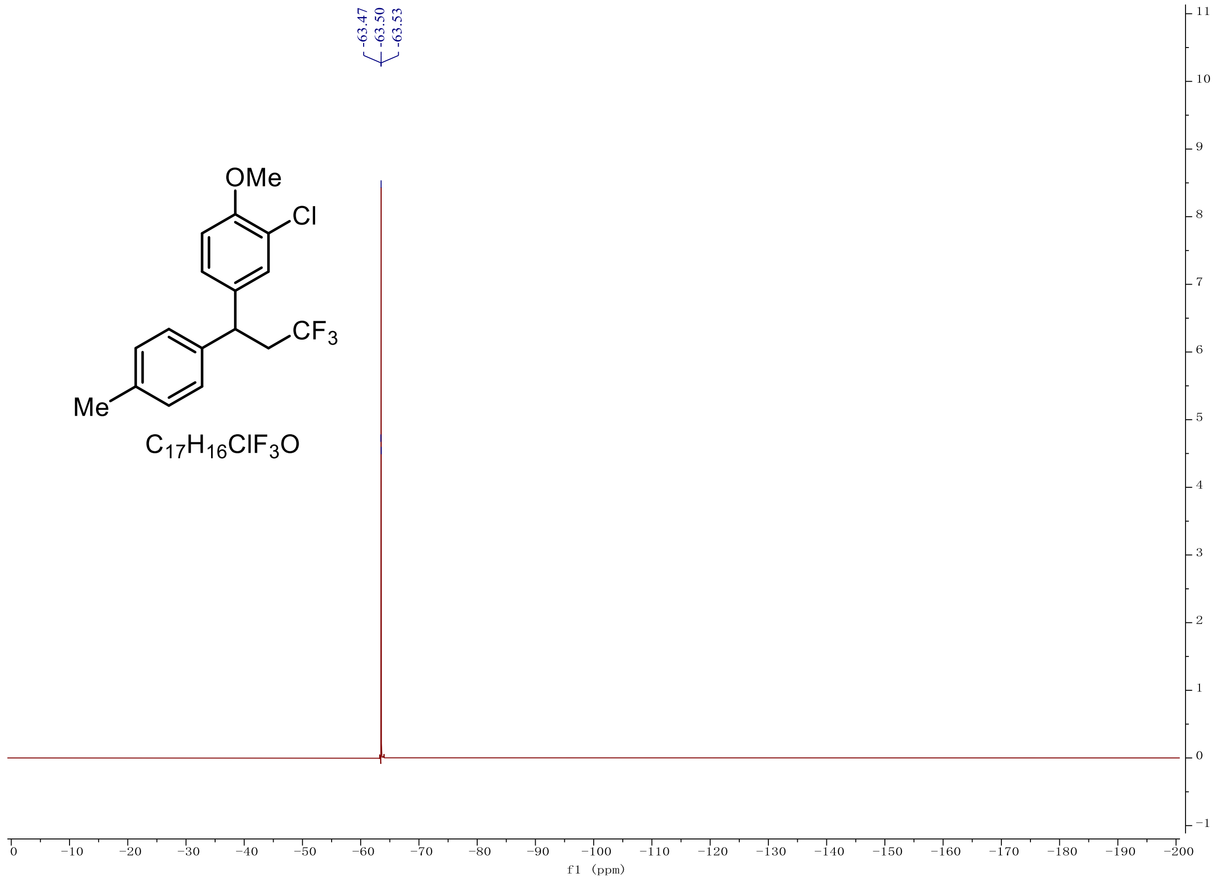

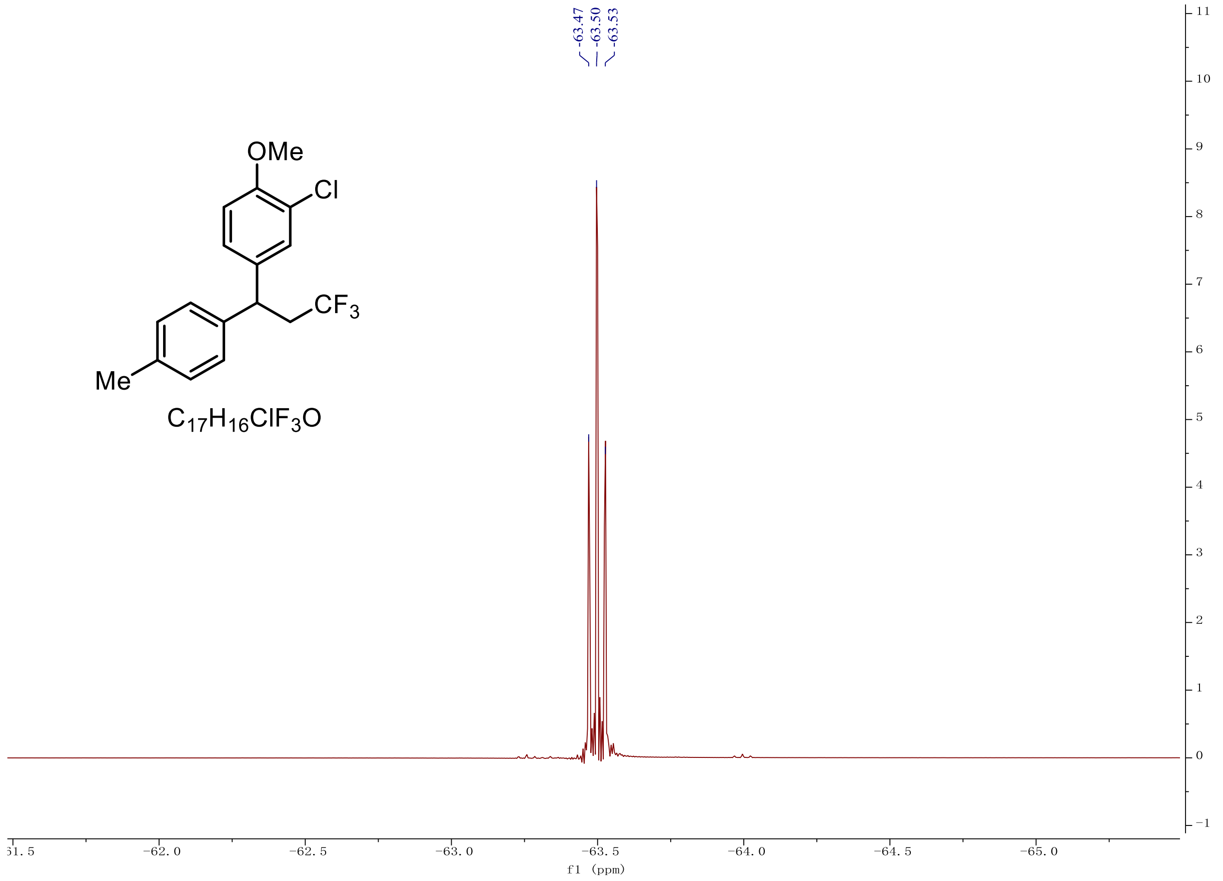

^{1}H NMR (400 MHz, $CDCl_3$) spectrum of 4e

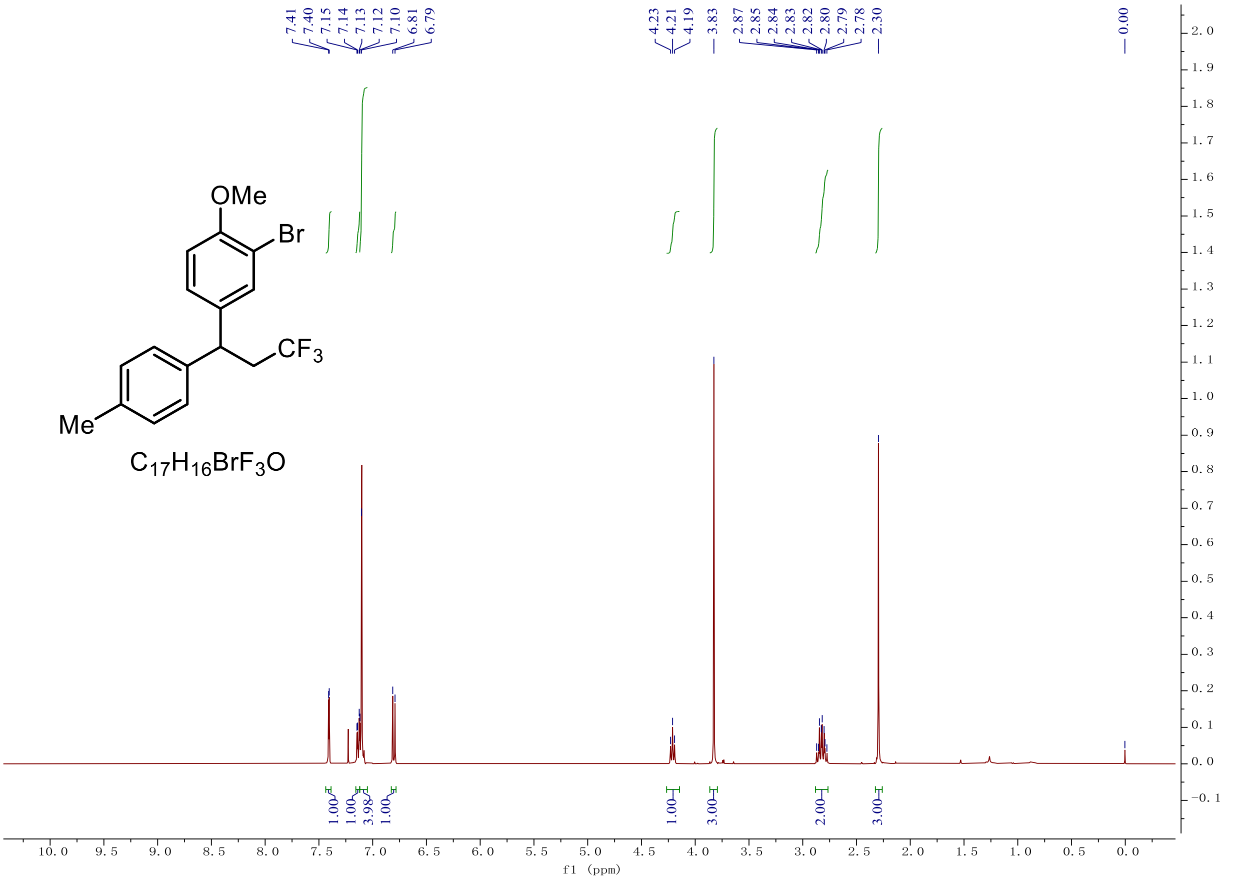

^{13}C NMR (101 MHz, $CDCl_3$) spectrum of 4e

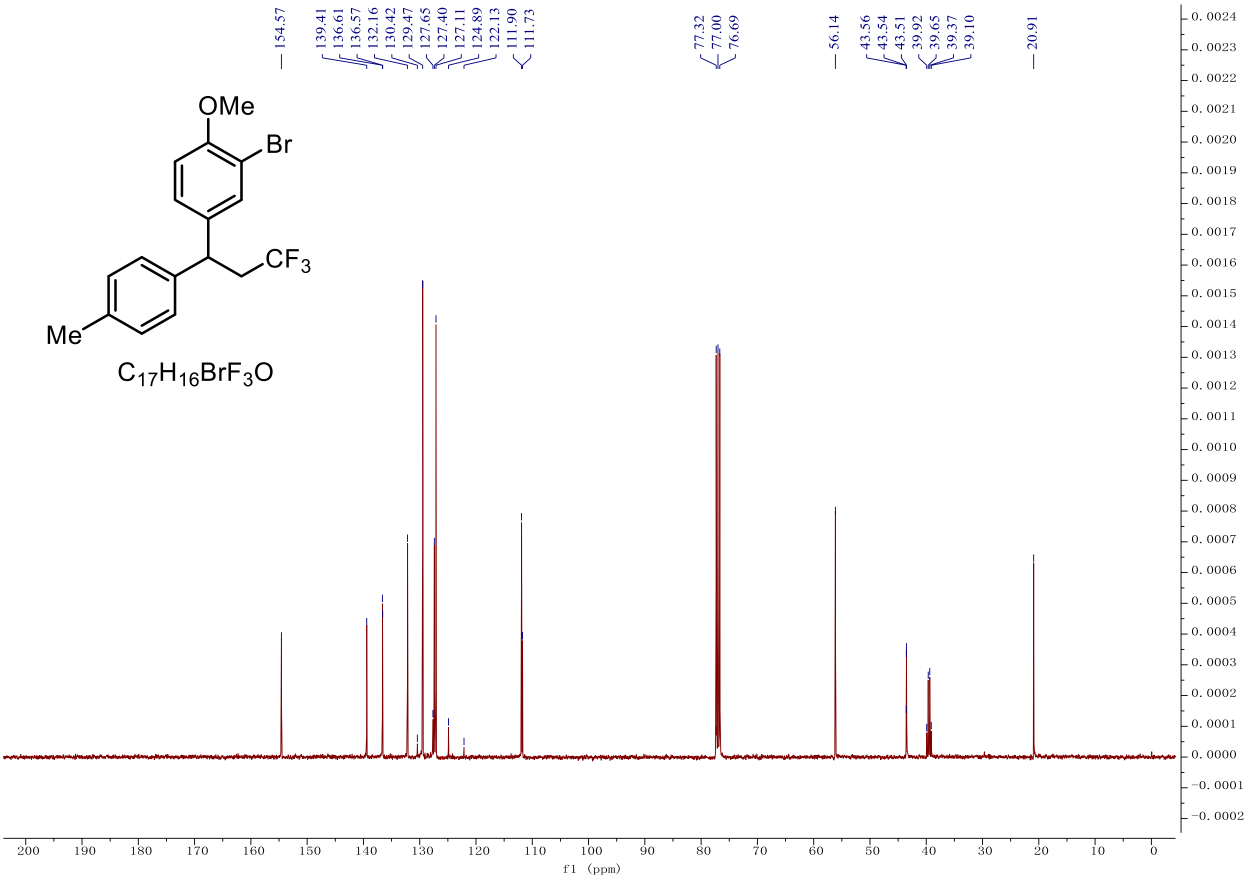

^{19}F NMR (376 MHz, CDCl3) spectrum of 4e

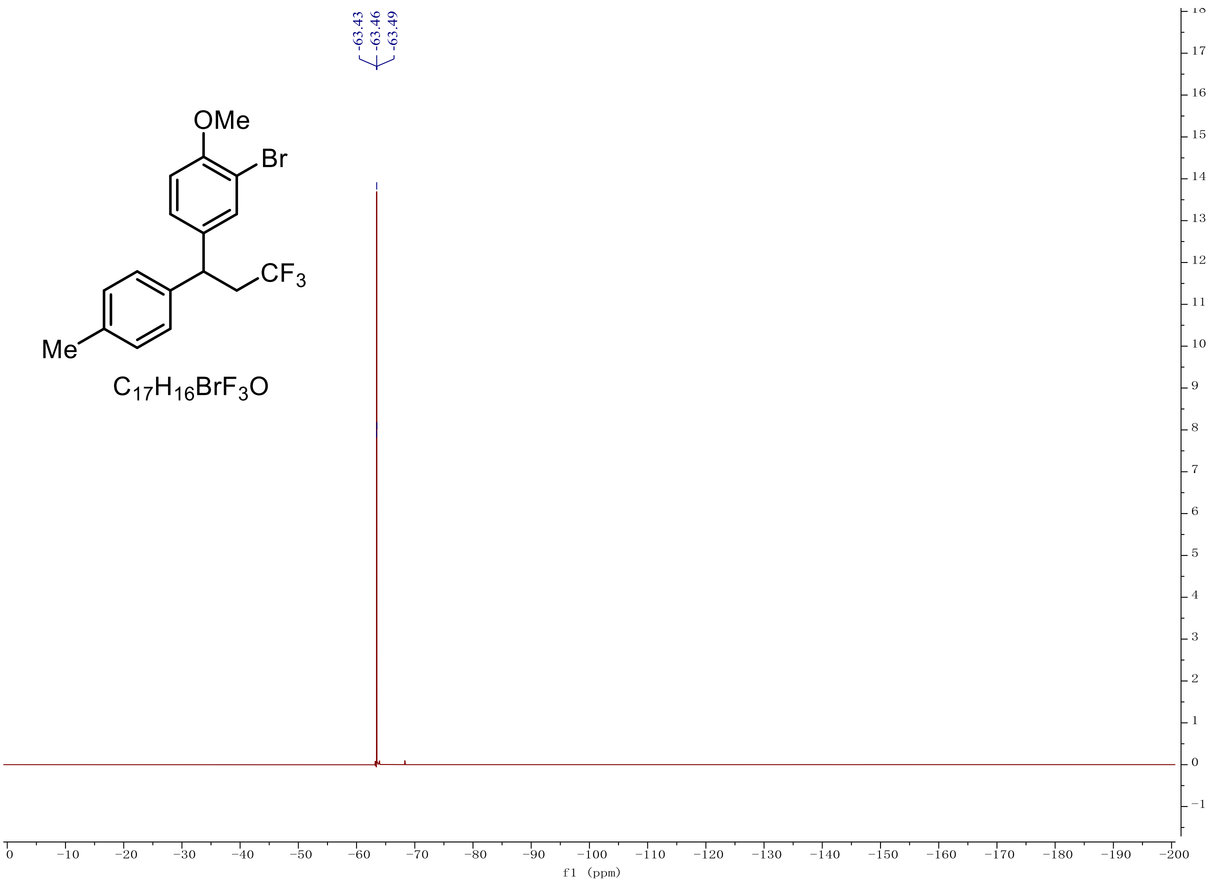

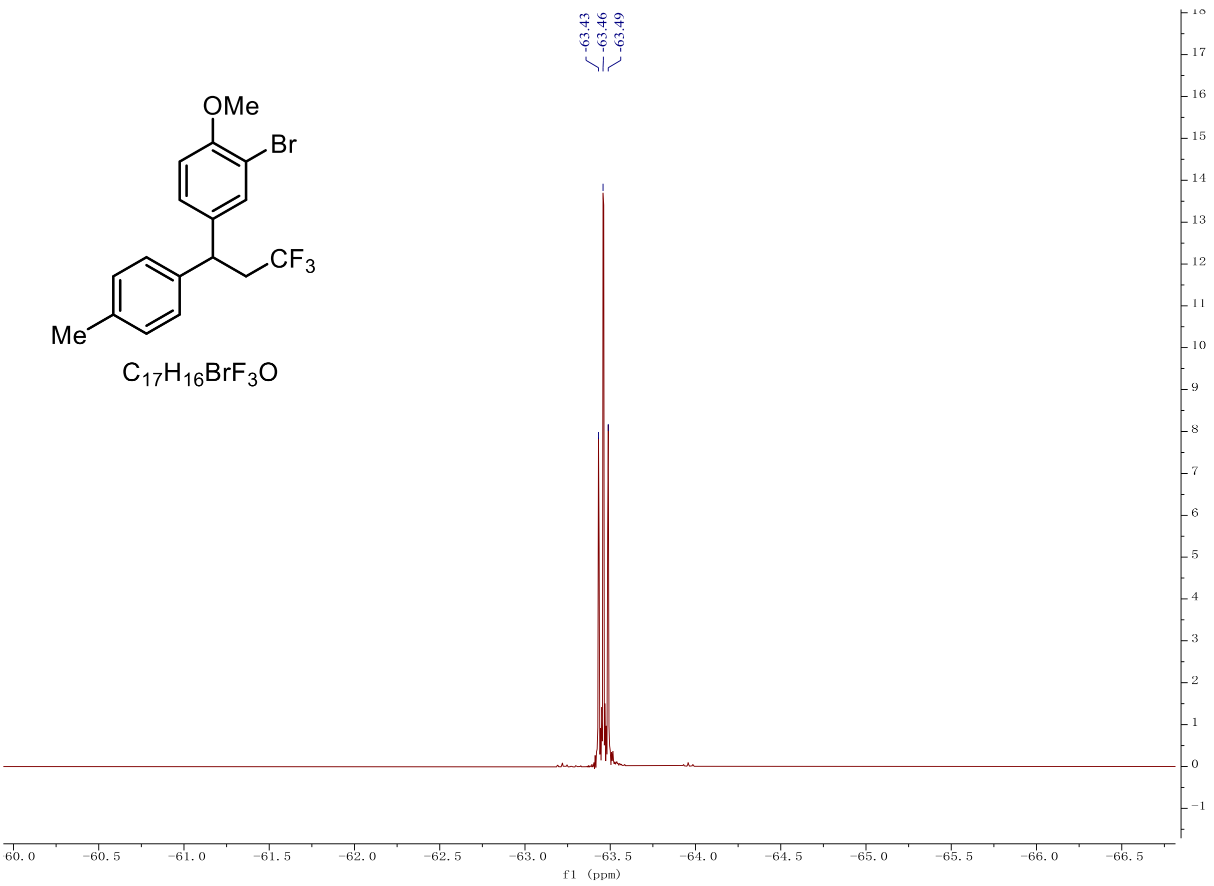

^{1}H NMR (400 MHz, $CDCl_3$) spectrum of 4f

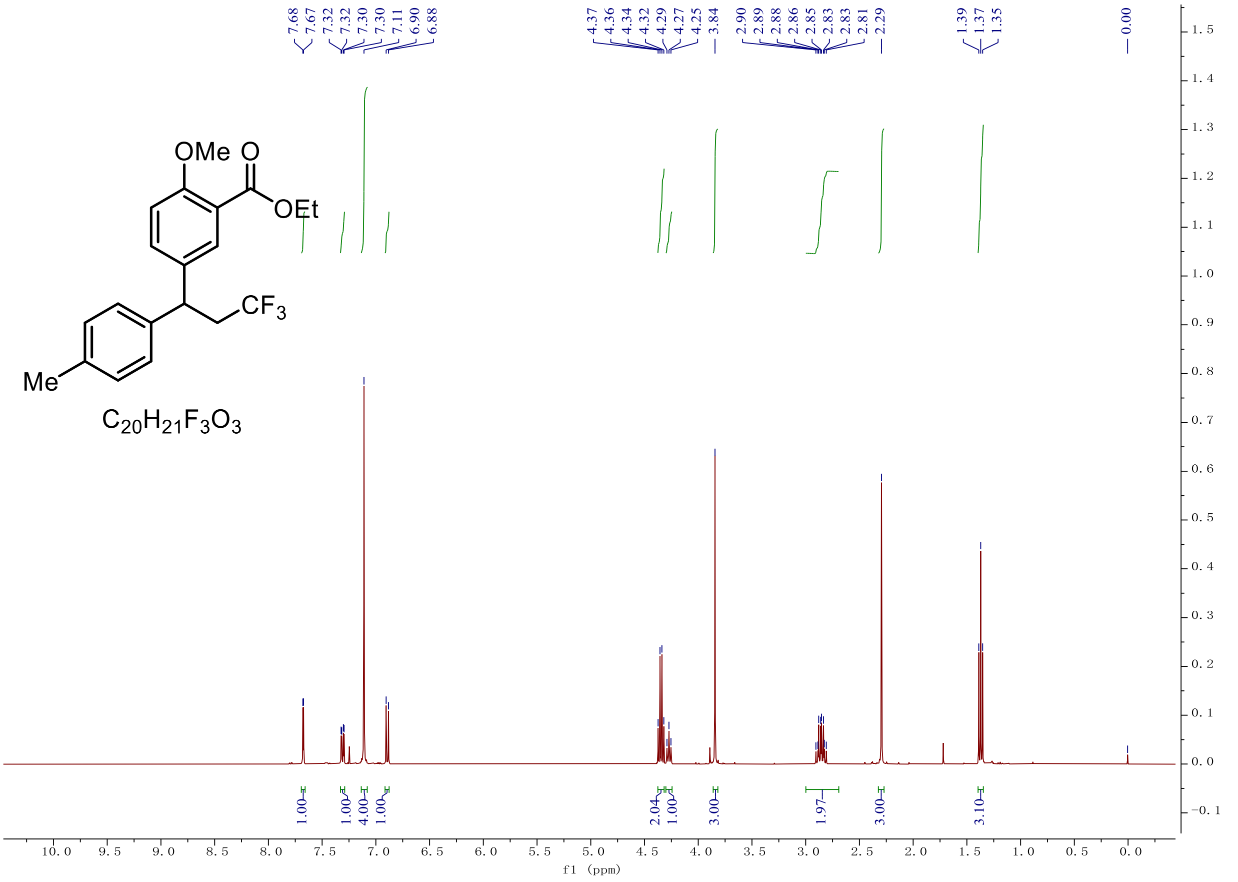

^{13}C NMR (101 MHz, $CDCl_3$) spectrum of 4f

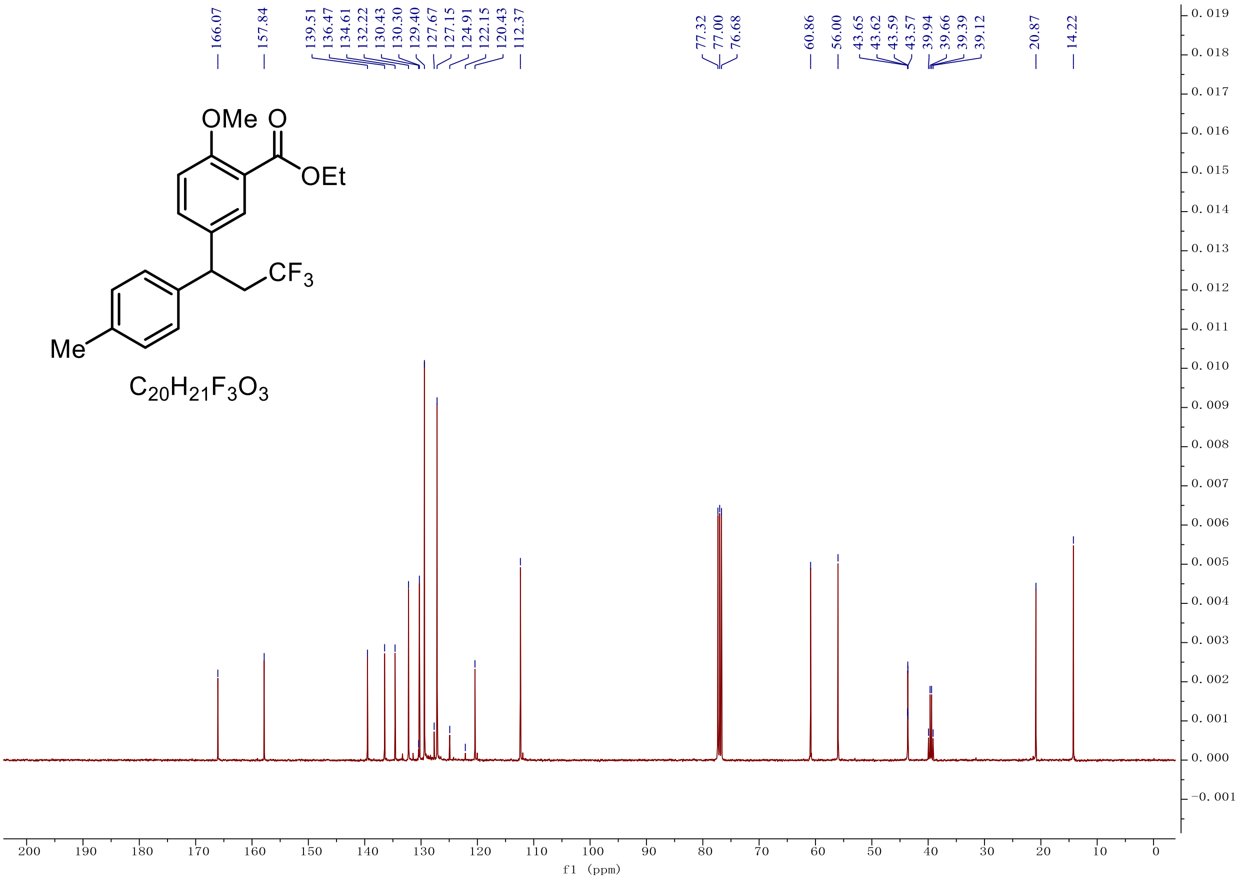

^{19}F NMR (376 MHz, $CDCl_3$) spectrum of 4f

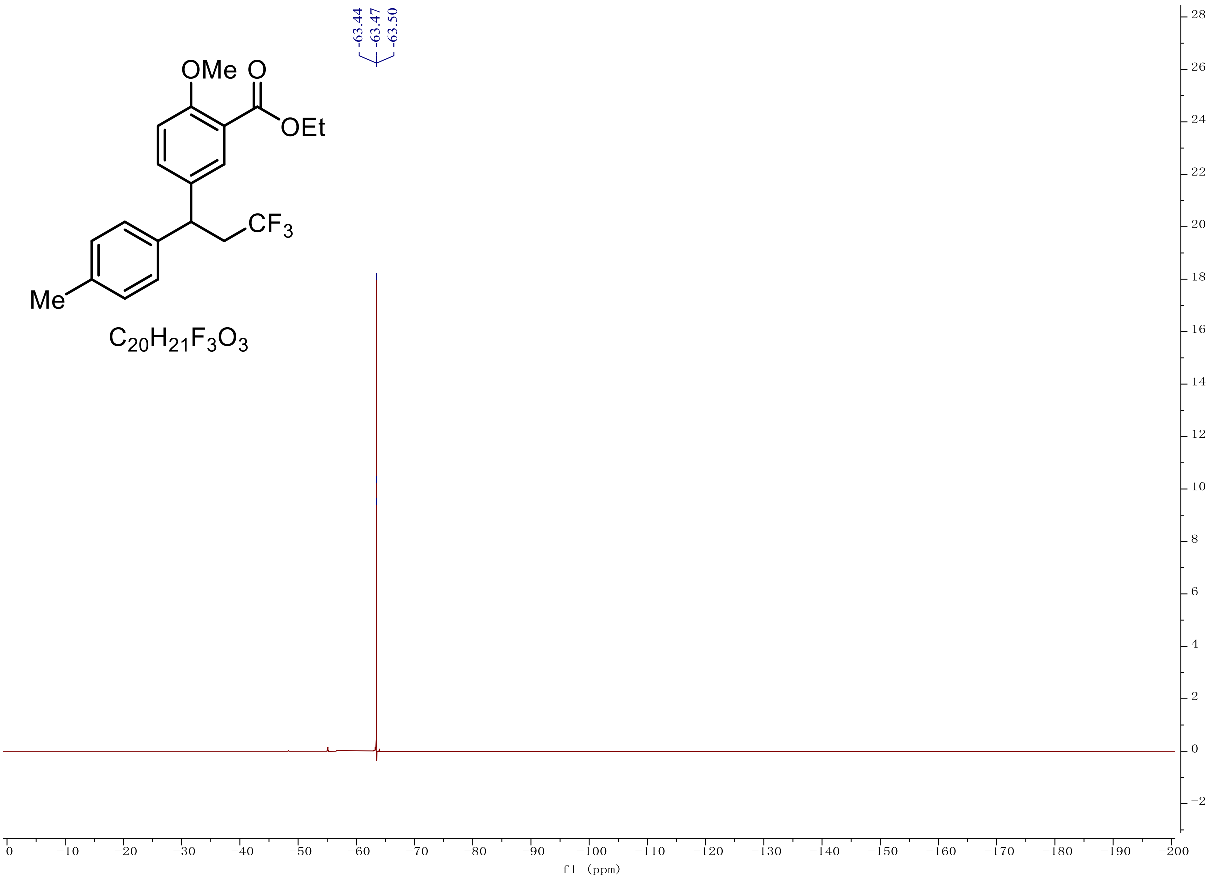

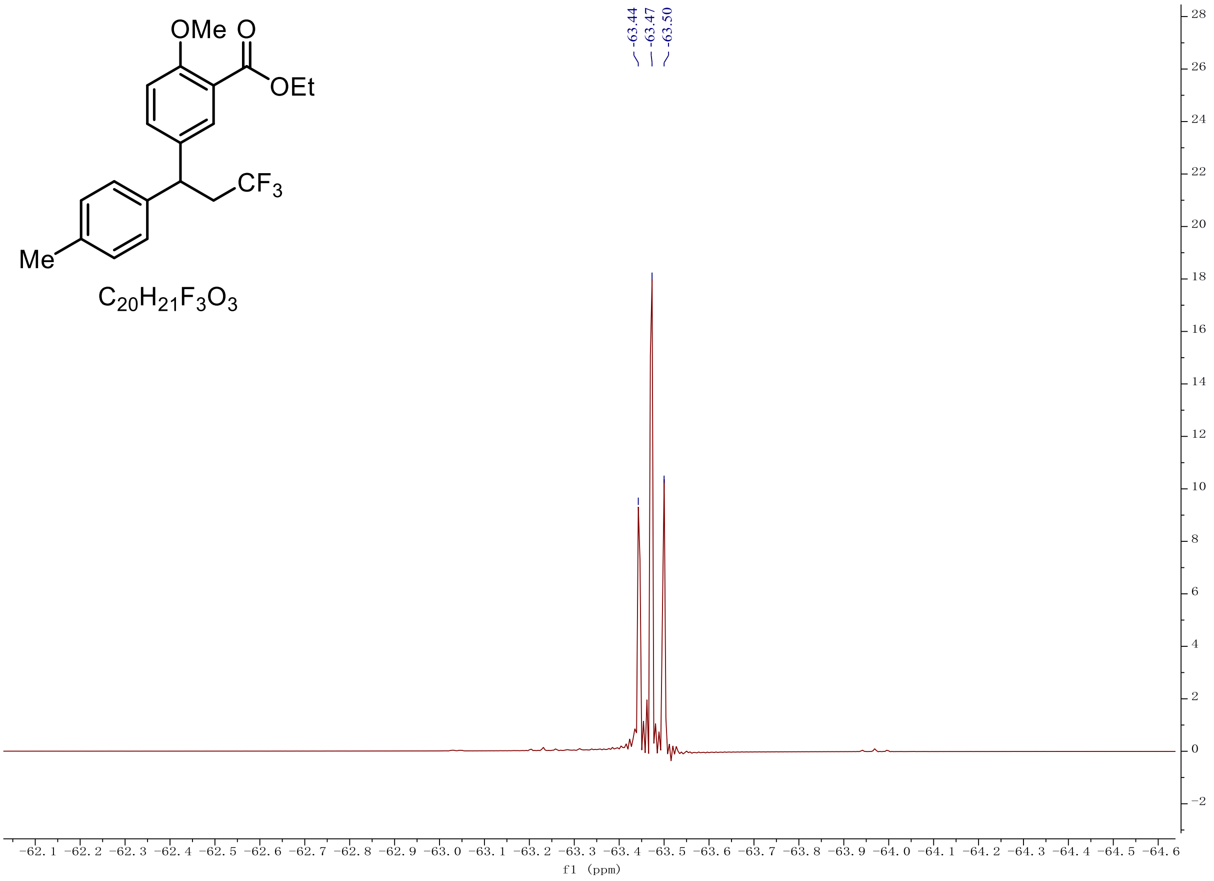

^{1}H NMR (400 MHz, $CDCl_3$) spectrum of 4g

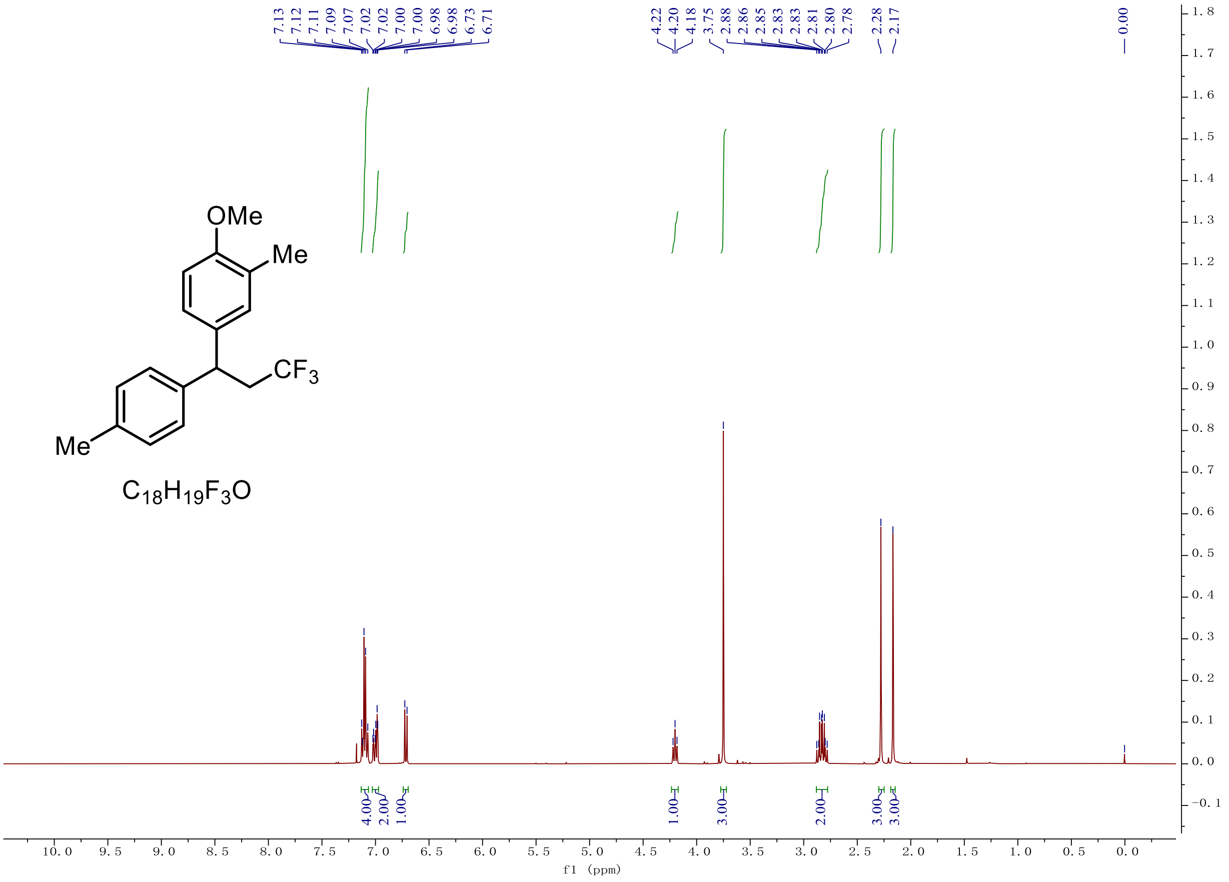

^{13}C NMR (101 MHz, $CDCl_3$) spectrum of 4g

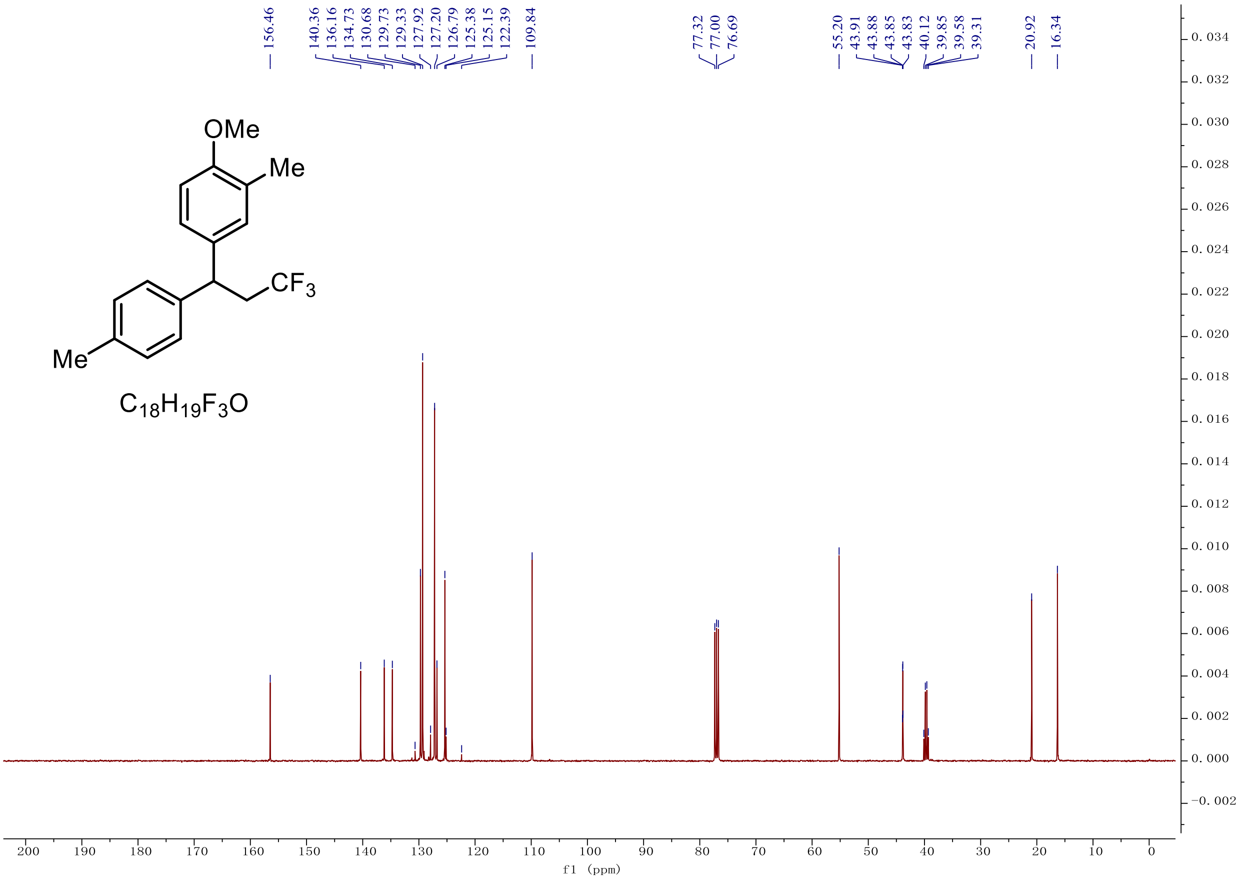

^{19}F NMR (376 MHz, CDCl3) spectrum of 4g

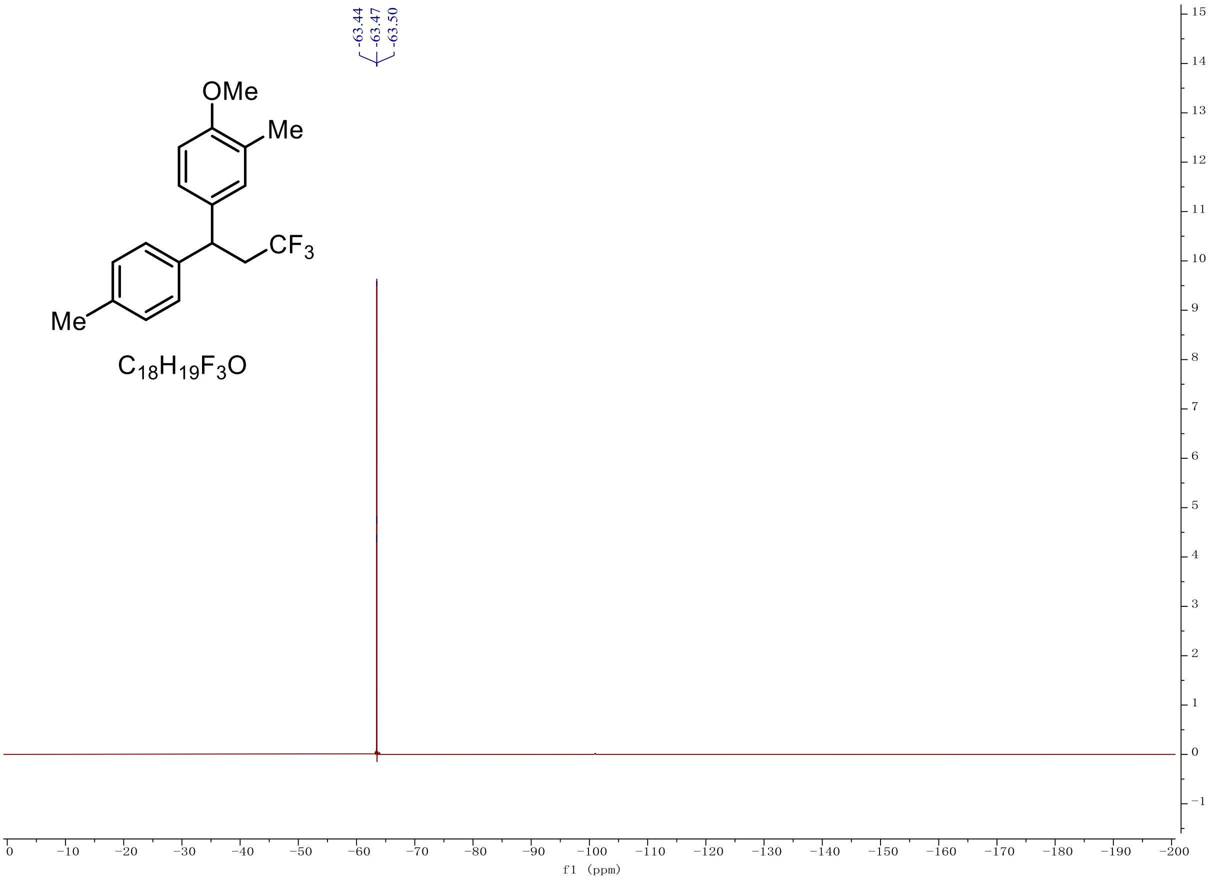

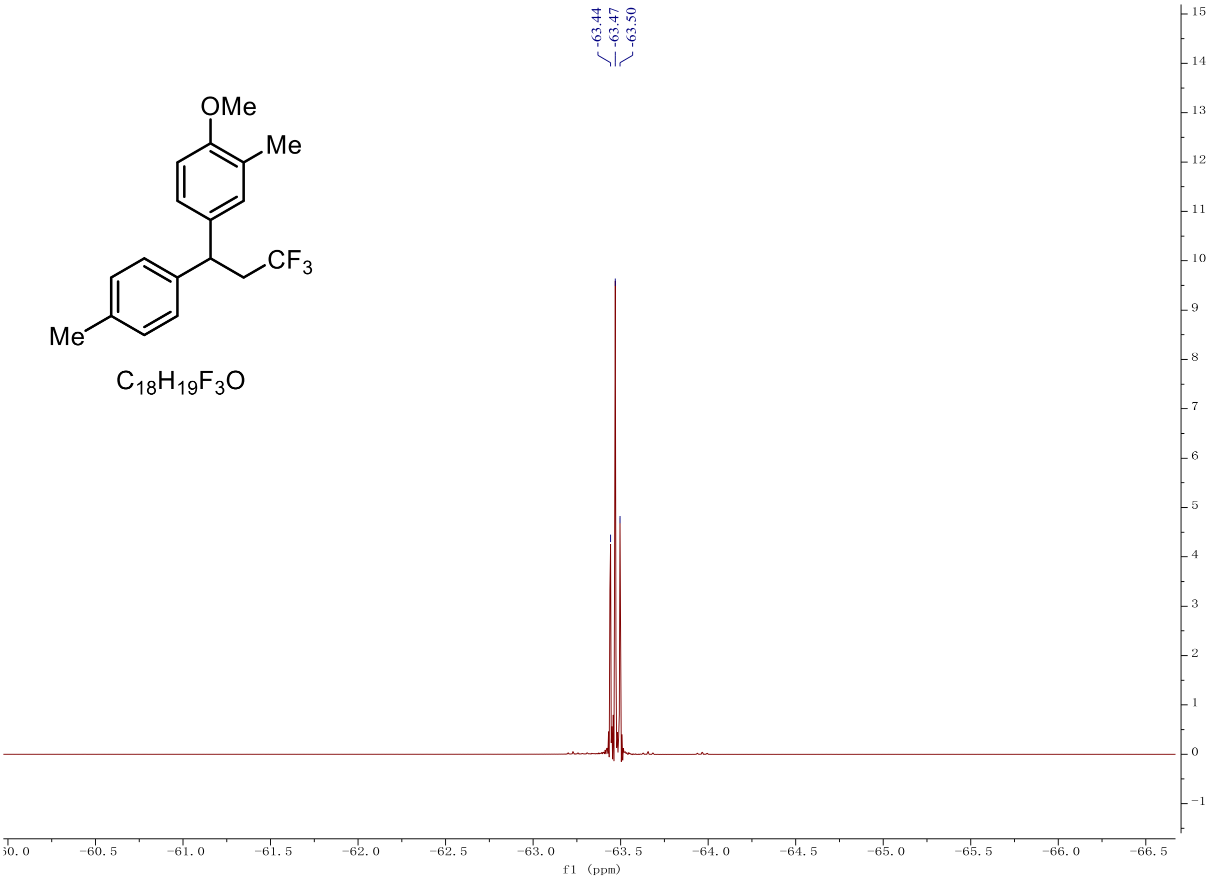

^{1}H NMR (400 MHz, $CDCl_3$) spectrum of 4h

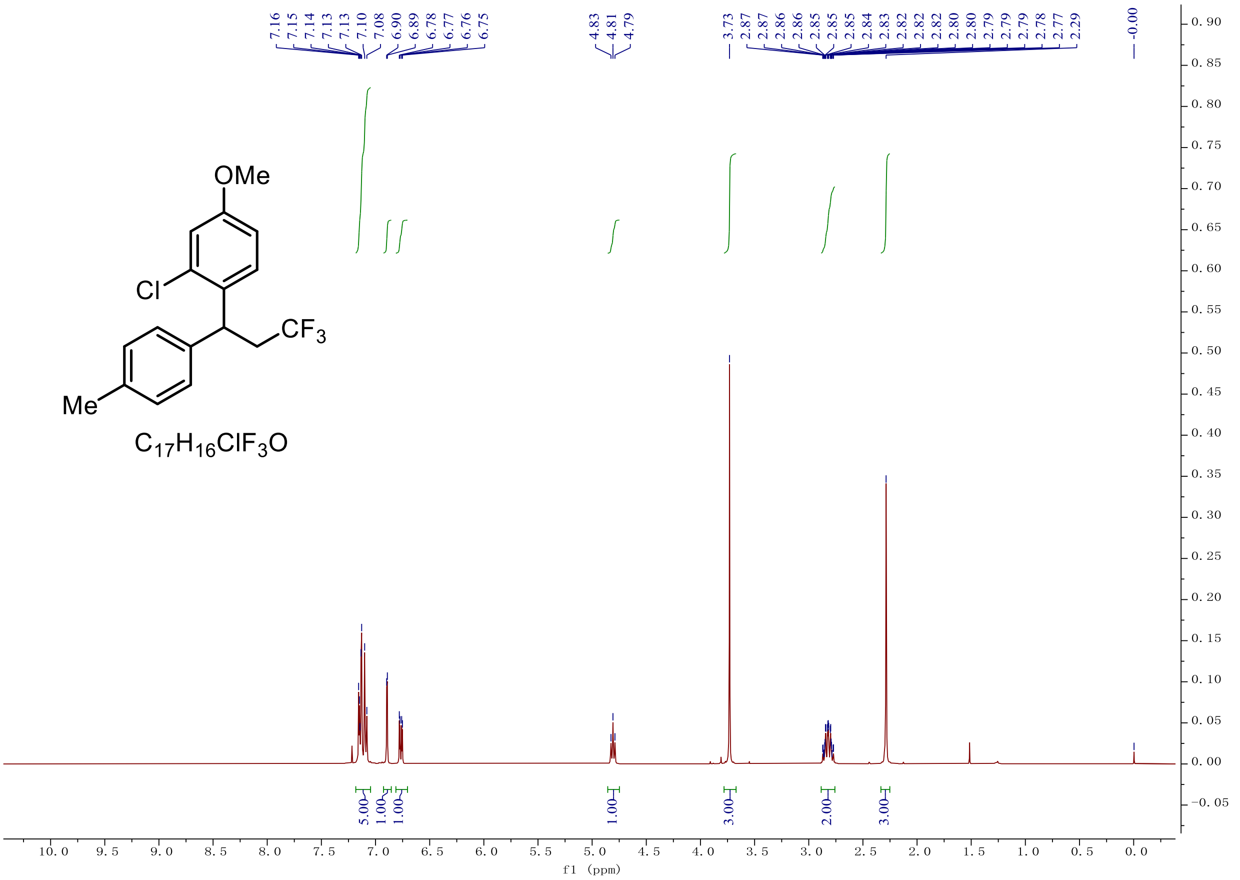

^{13}C NMR (101 MHz, $CDCl_3$) spectrum of 4h

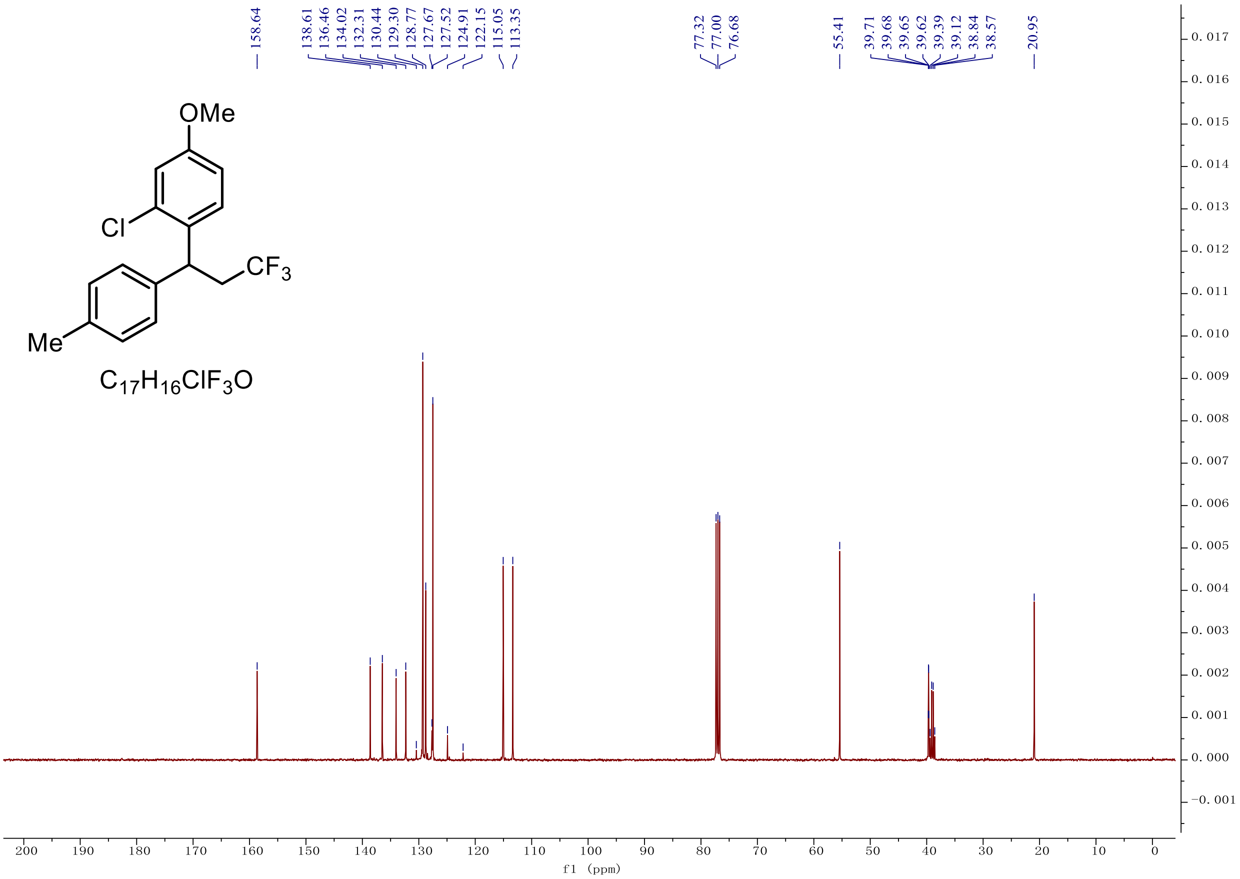

^{19}F NMR (376 MHz, $CDCl_3$) spectrum of 4h

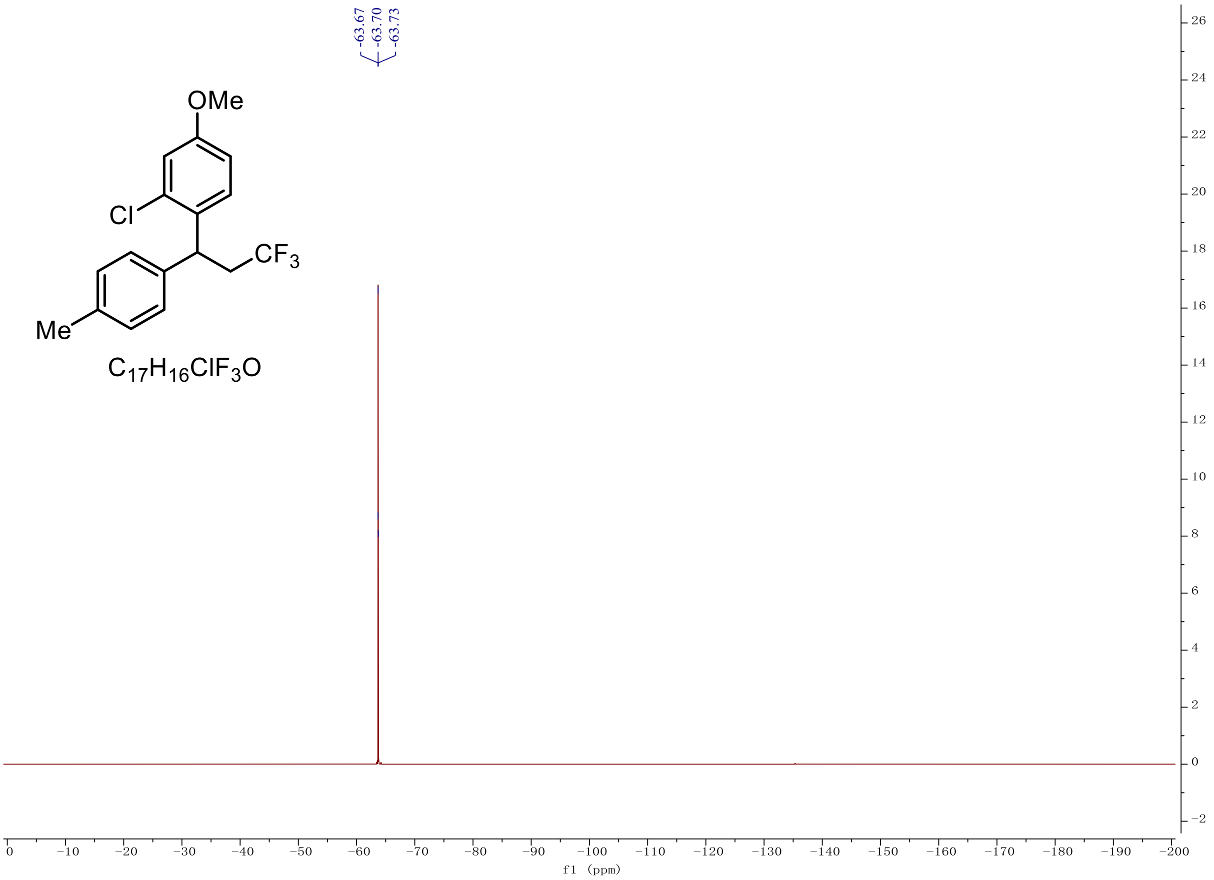

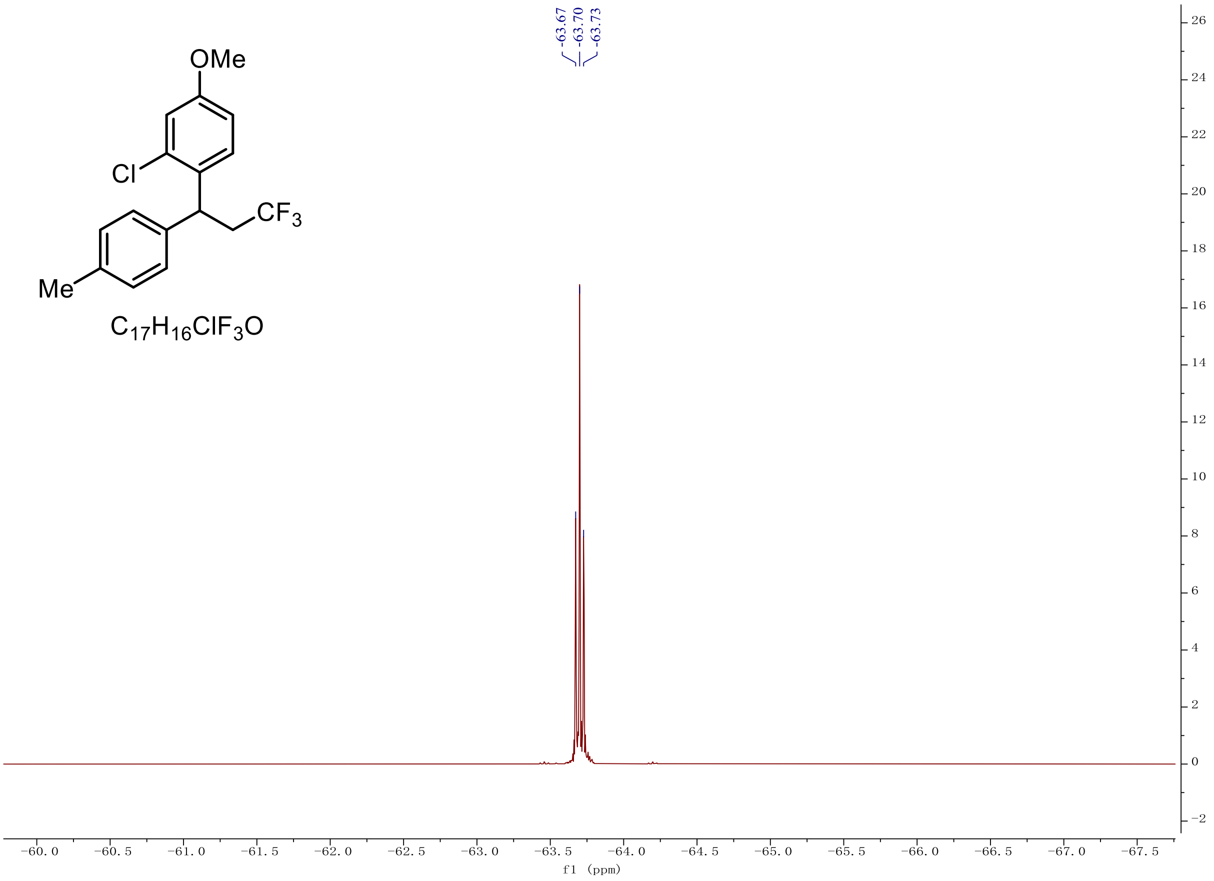

^{1}H NMR (400 MHz, $CDCl_3$) spectrum of 4i

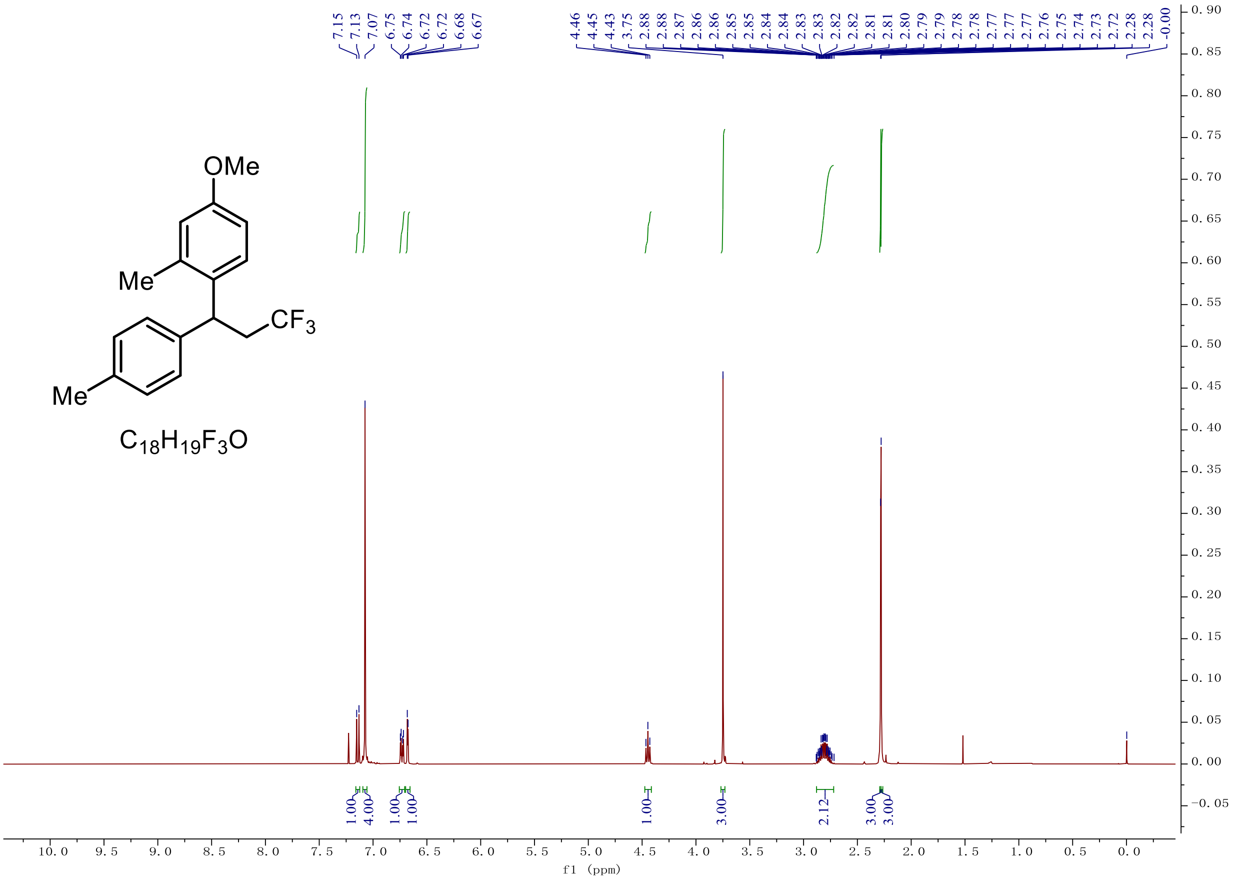

^{13}C NMR (101 MHz, $CDCl_3$) spectrum of 4i

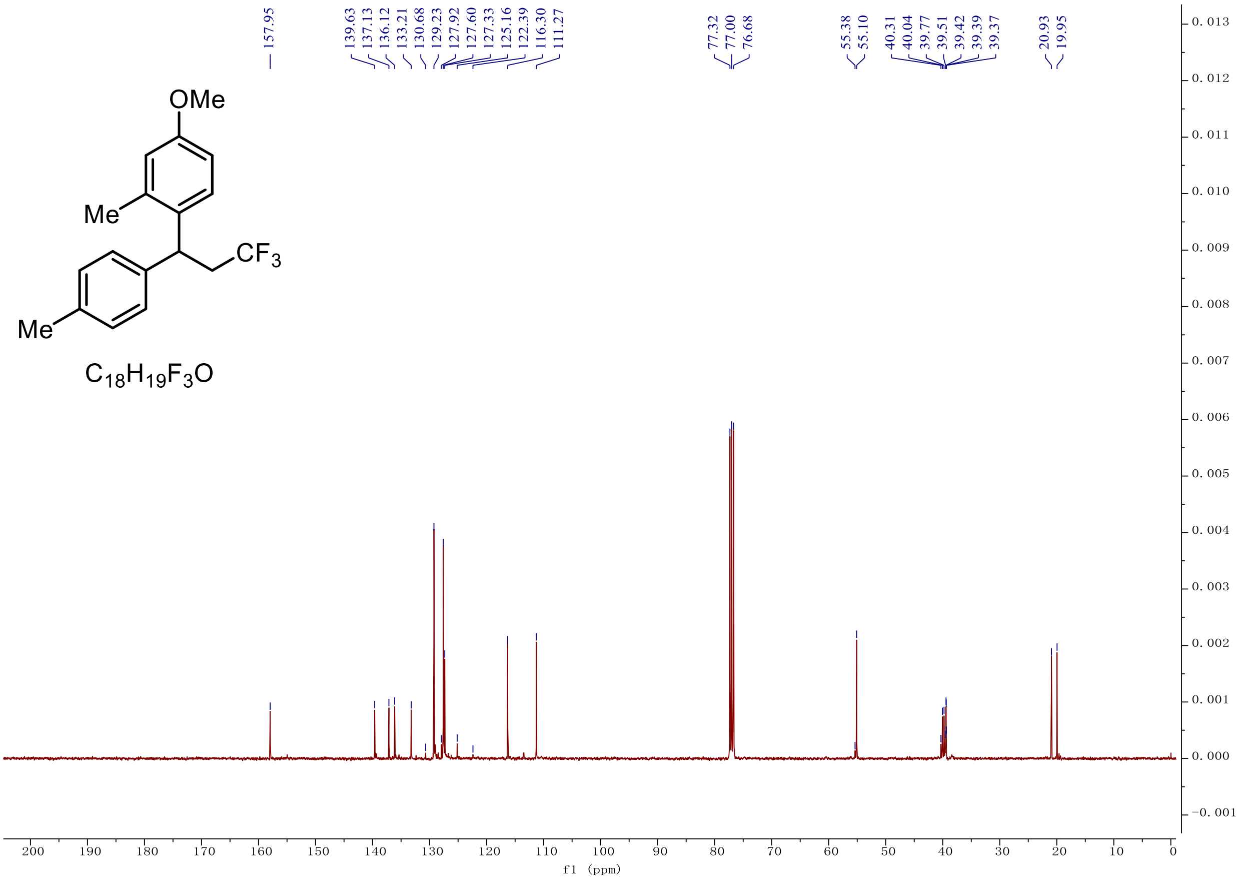

^{19}F NMR (376 MHz, CDCl3) spectrum of 4i

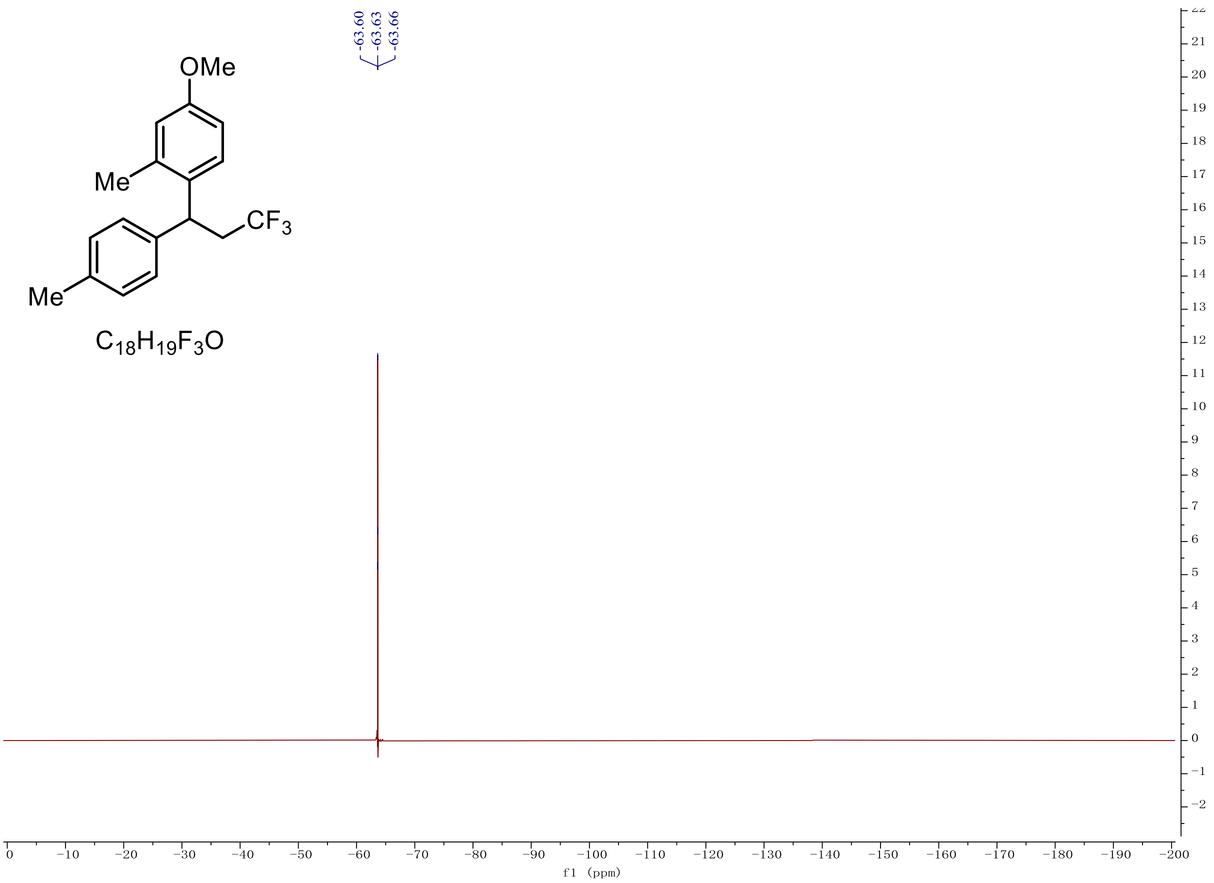

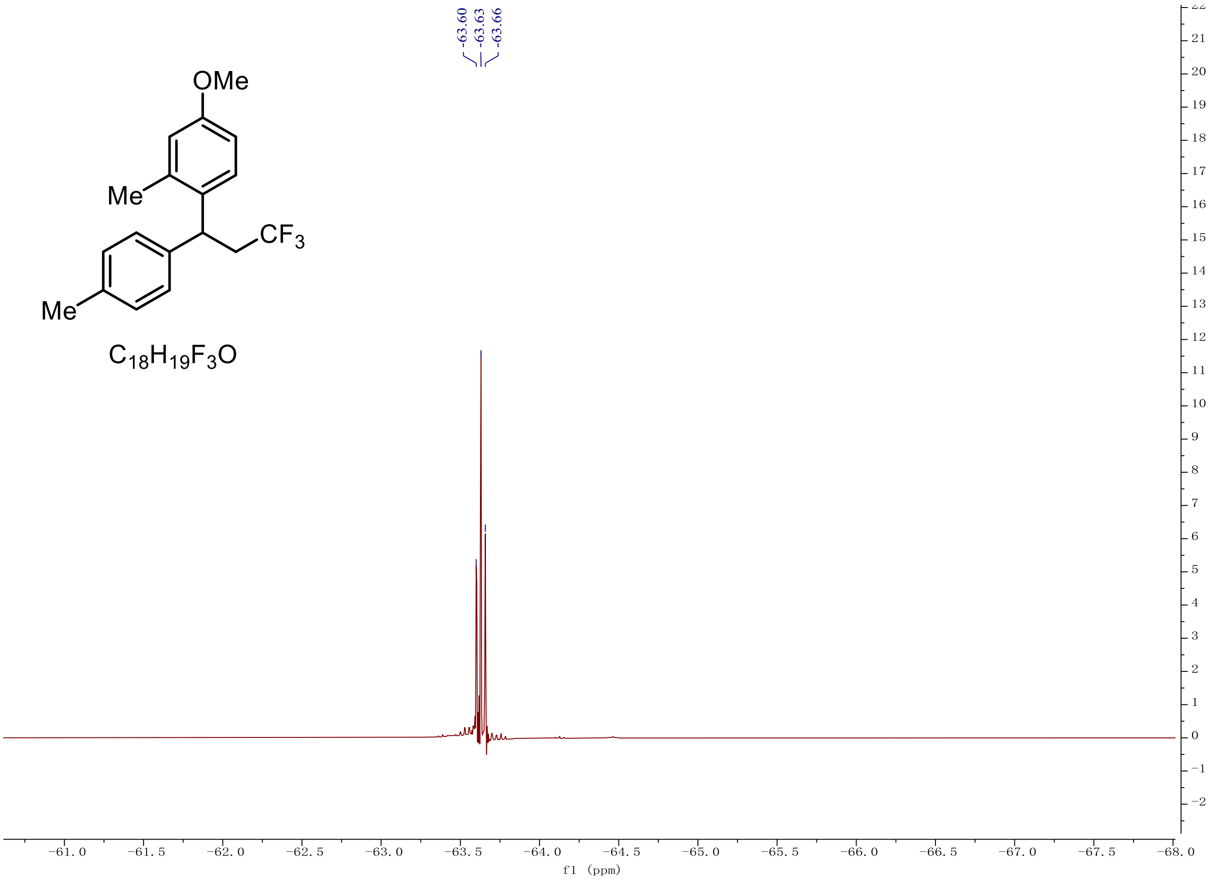

^{1}H NMR (400 MHz, CDCl3) spectrum of 4i'

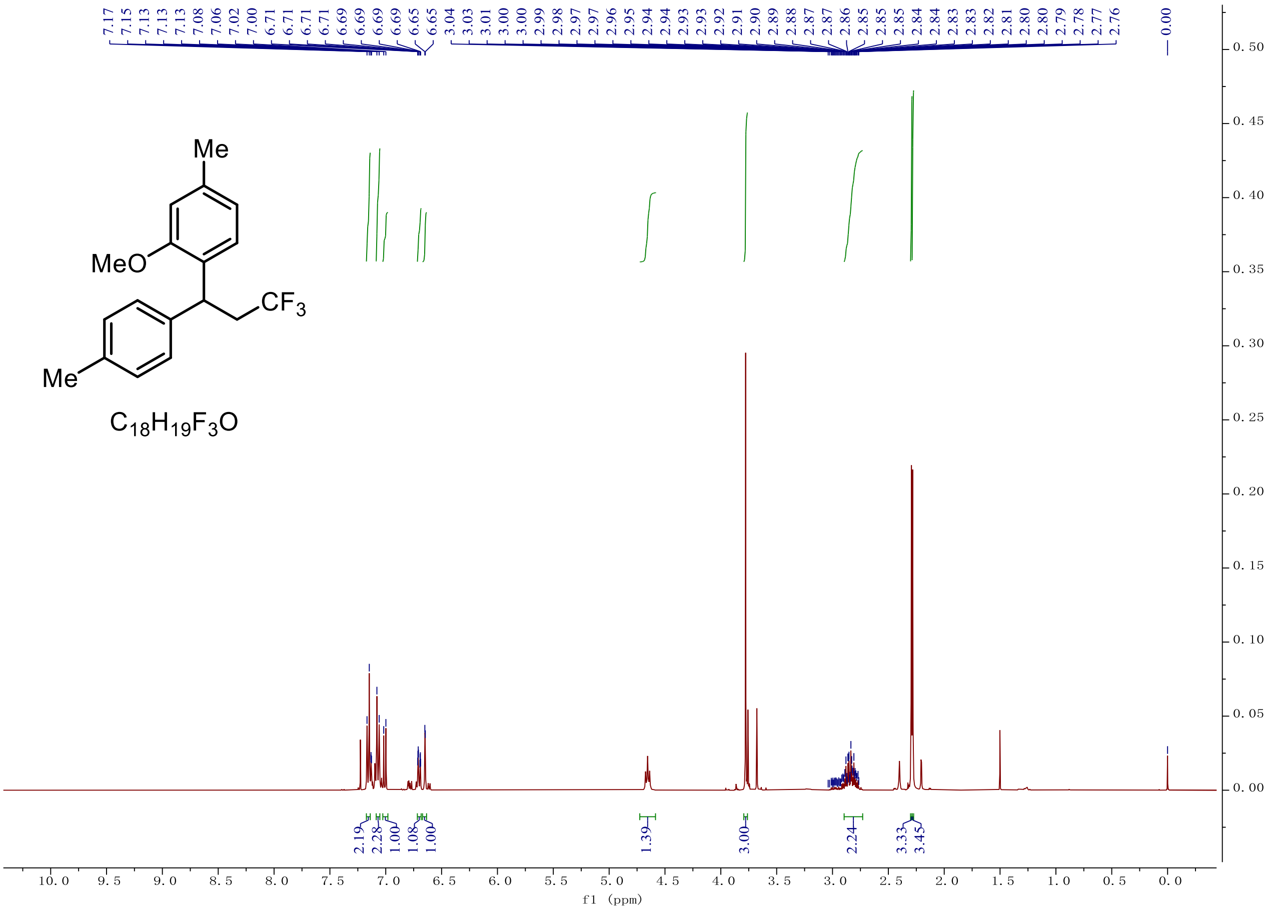

^{13}C NMR (101 MHz, CDCl3) spectrum of 4i'

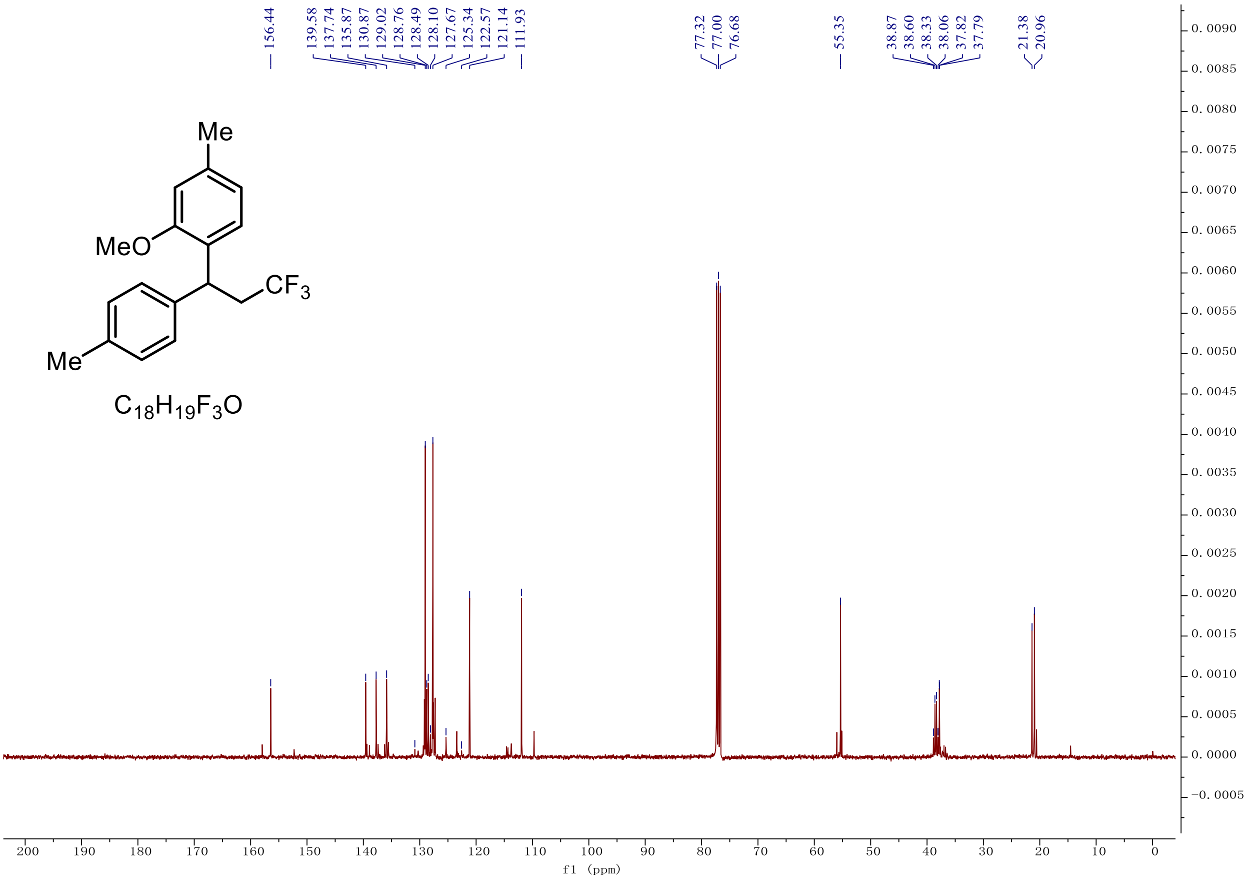

^{19}F NMR (376 MHz, $CDCl_3$) spectrum of 4i'

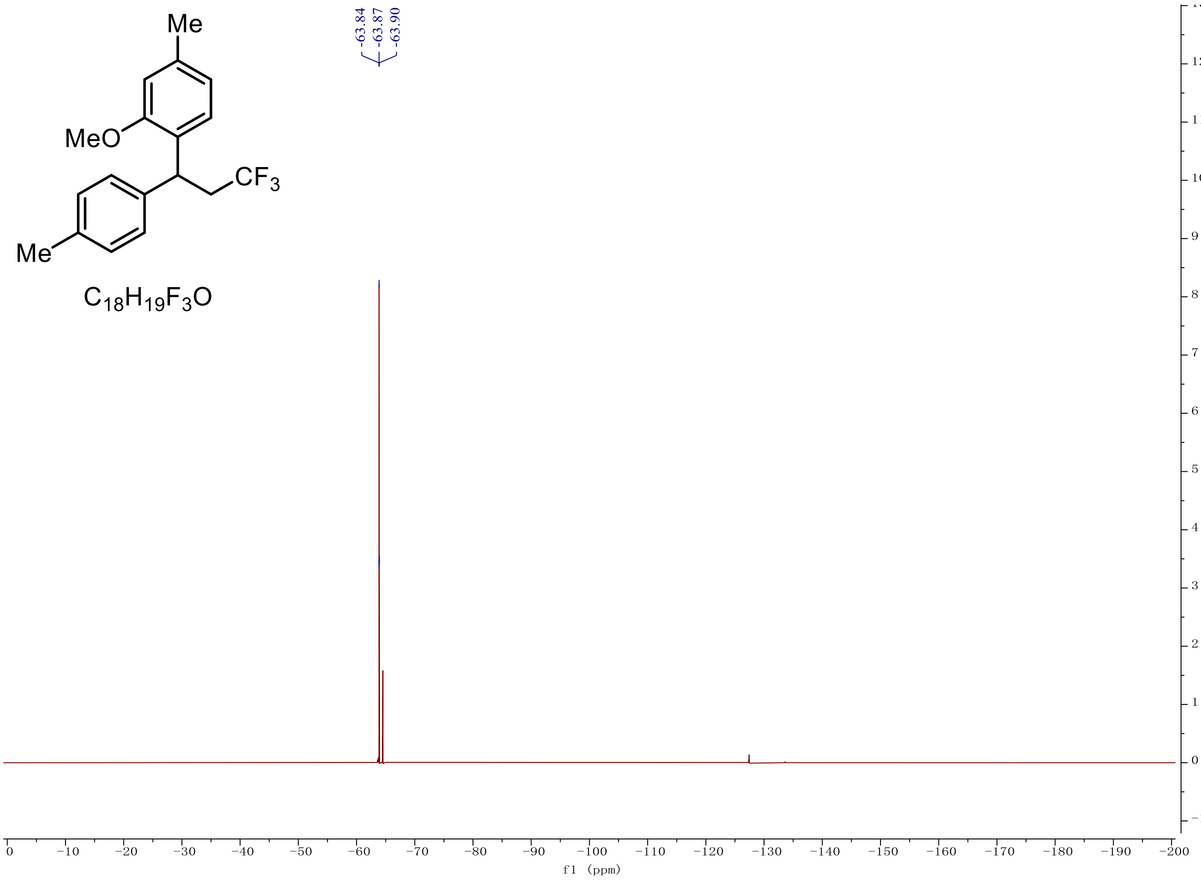

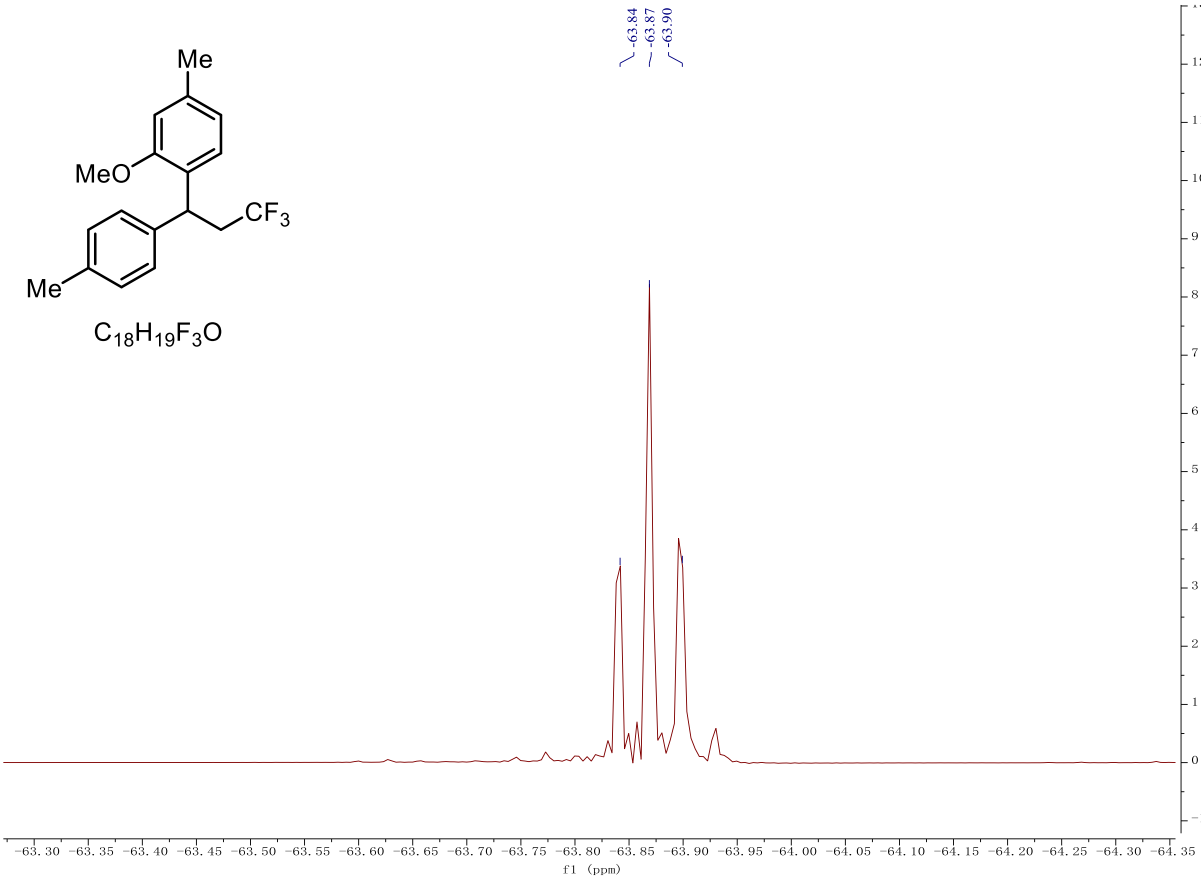

^{1}H NMR (400 MHz, CDCl3) spectrum of 4j

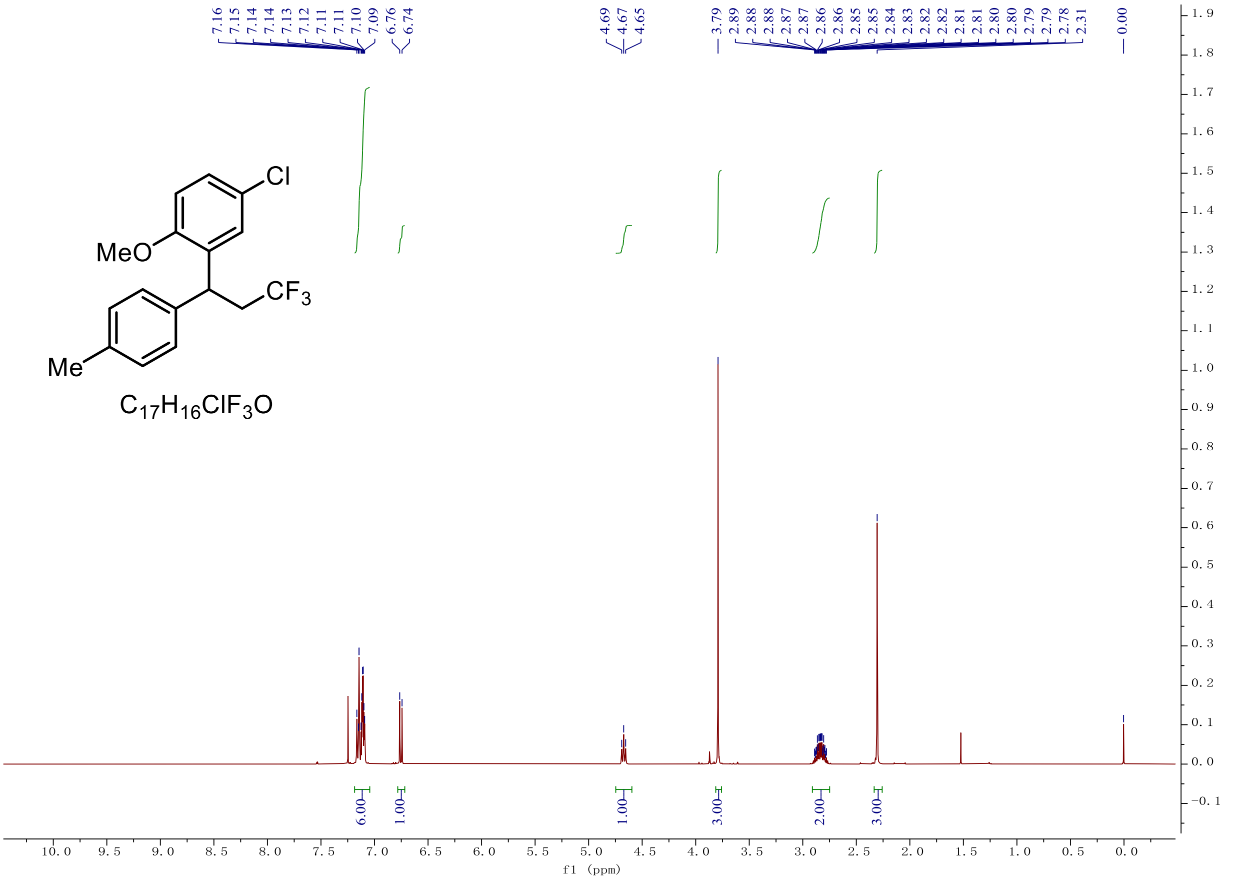

^{13}C NMR (101 MHz, CDCl3) spectrum of 4j

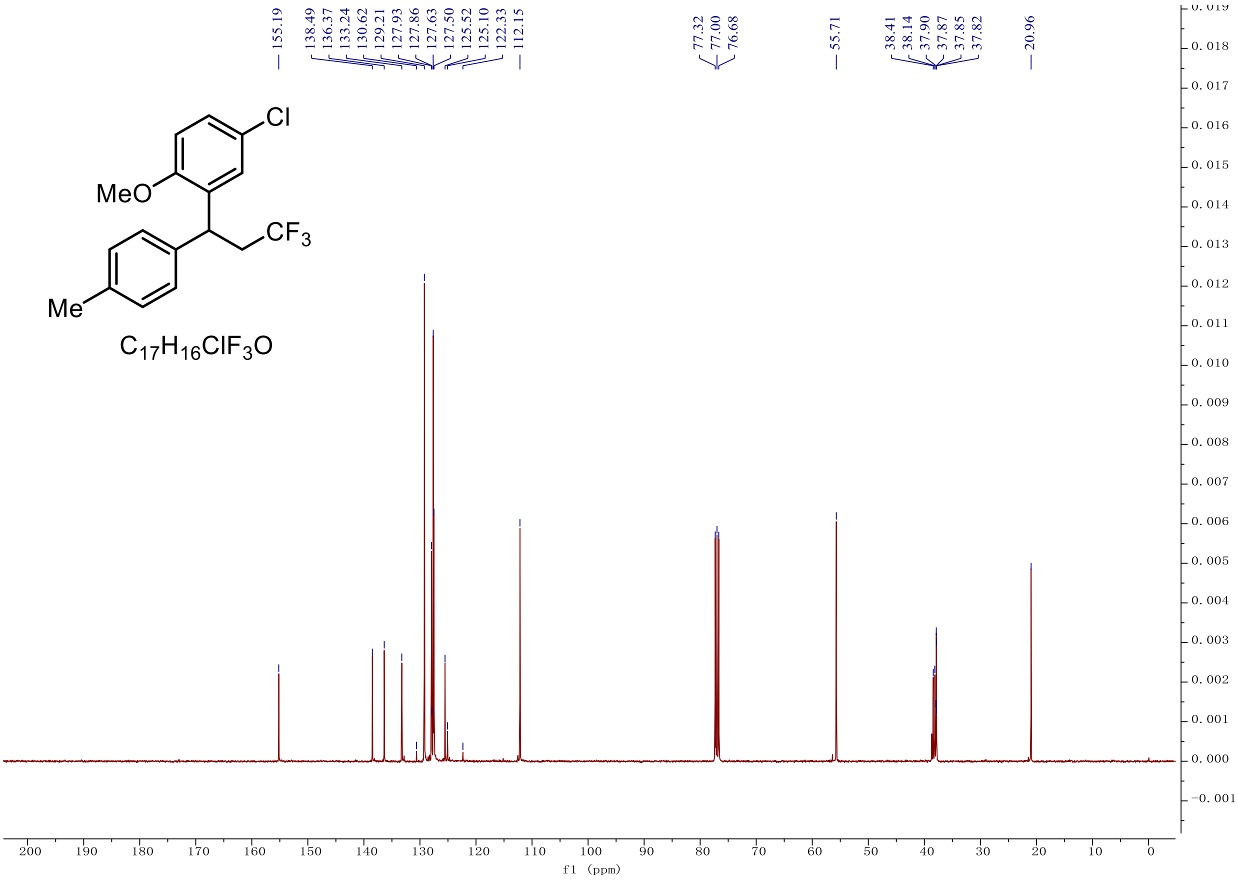

^{19}F NMR (376 MHz, CDCl₃) spectrum of 4j

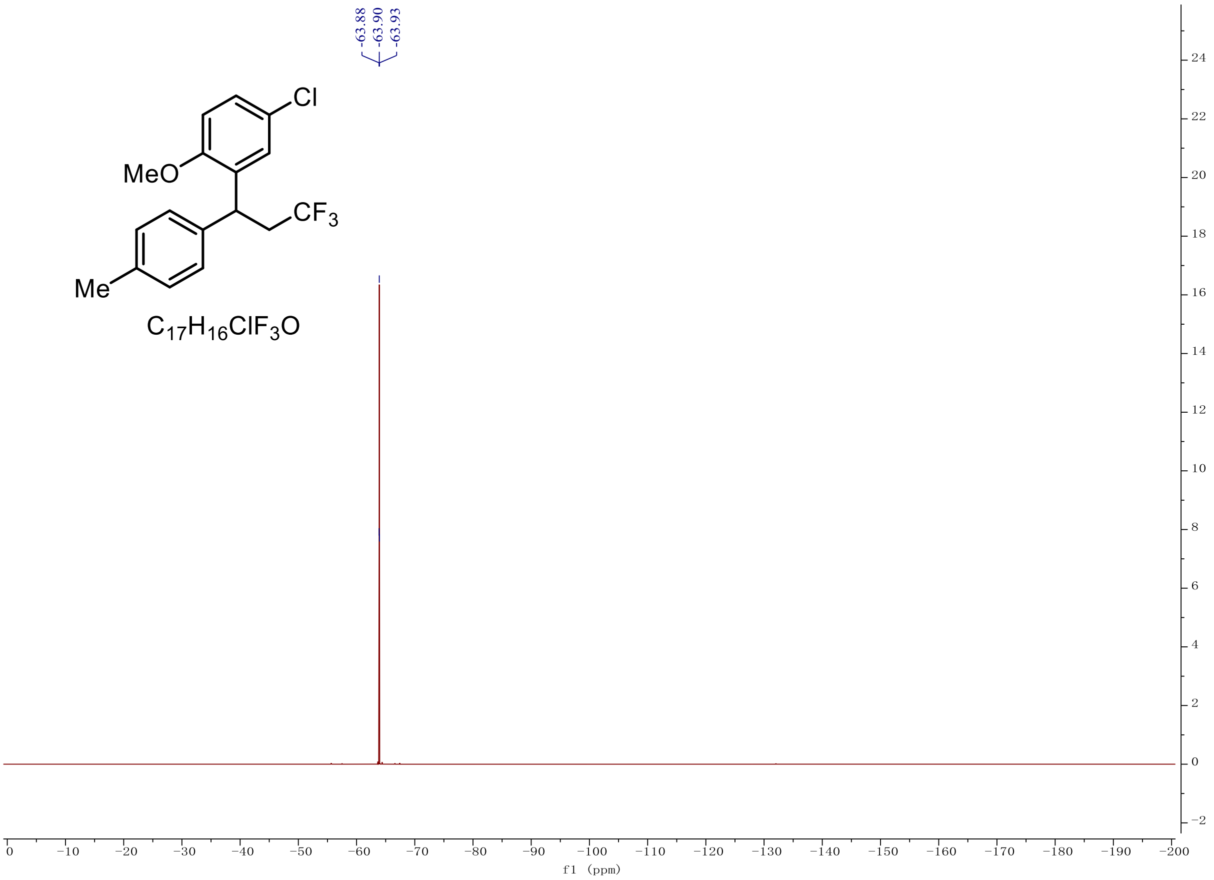

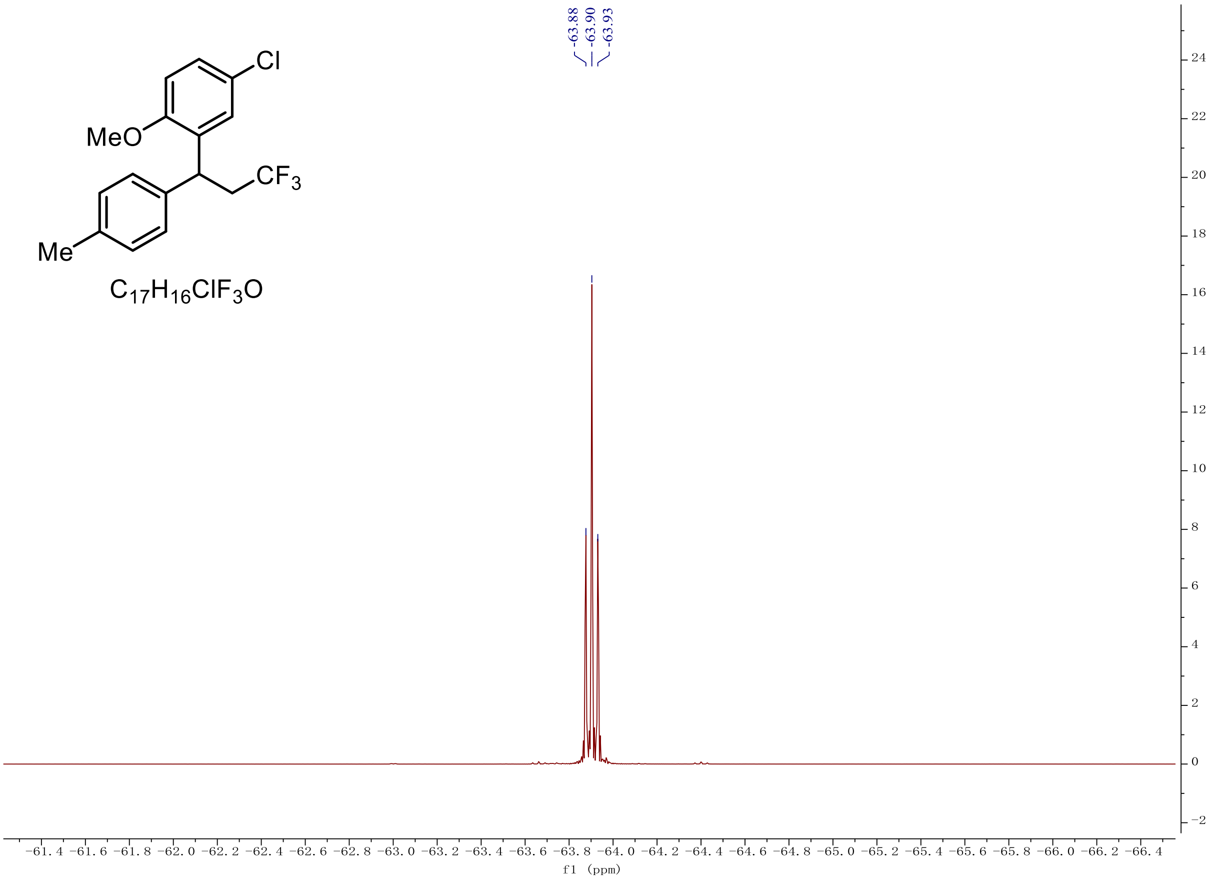

^{1}H NMR (400 MHz, CDCl$_3$) spectrum of 4k

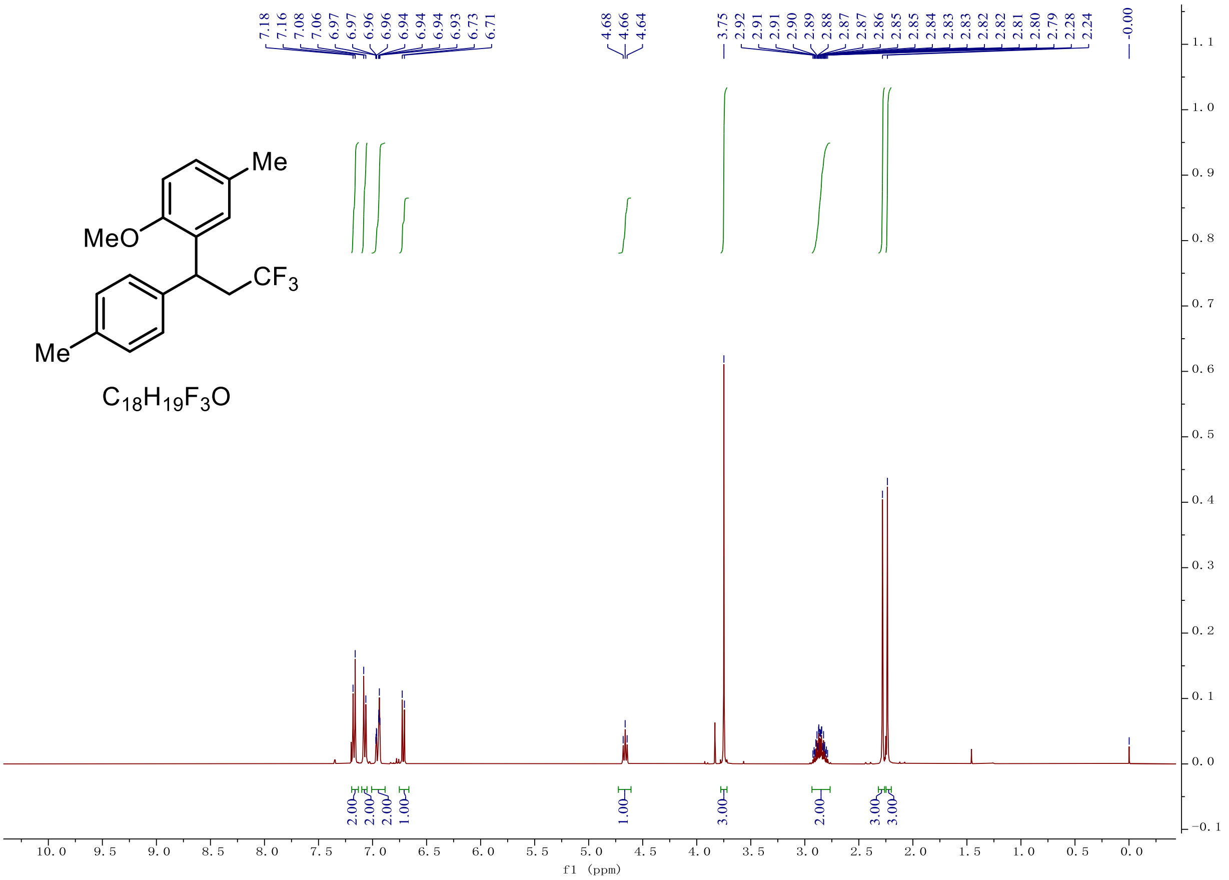

^{13}C NMR (101 MHz, CDCl$_3$) spectrum of 4k

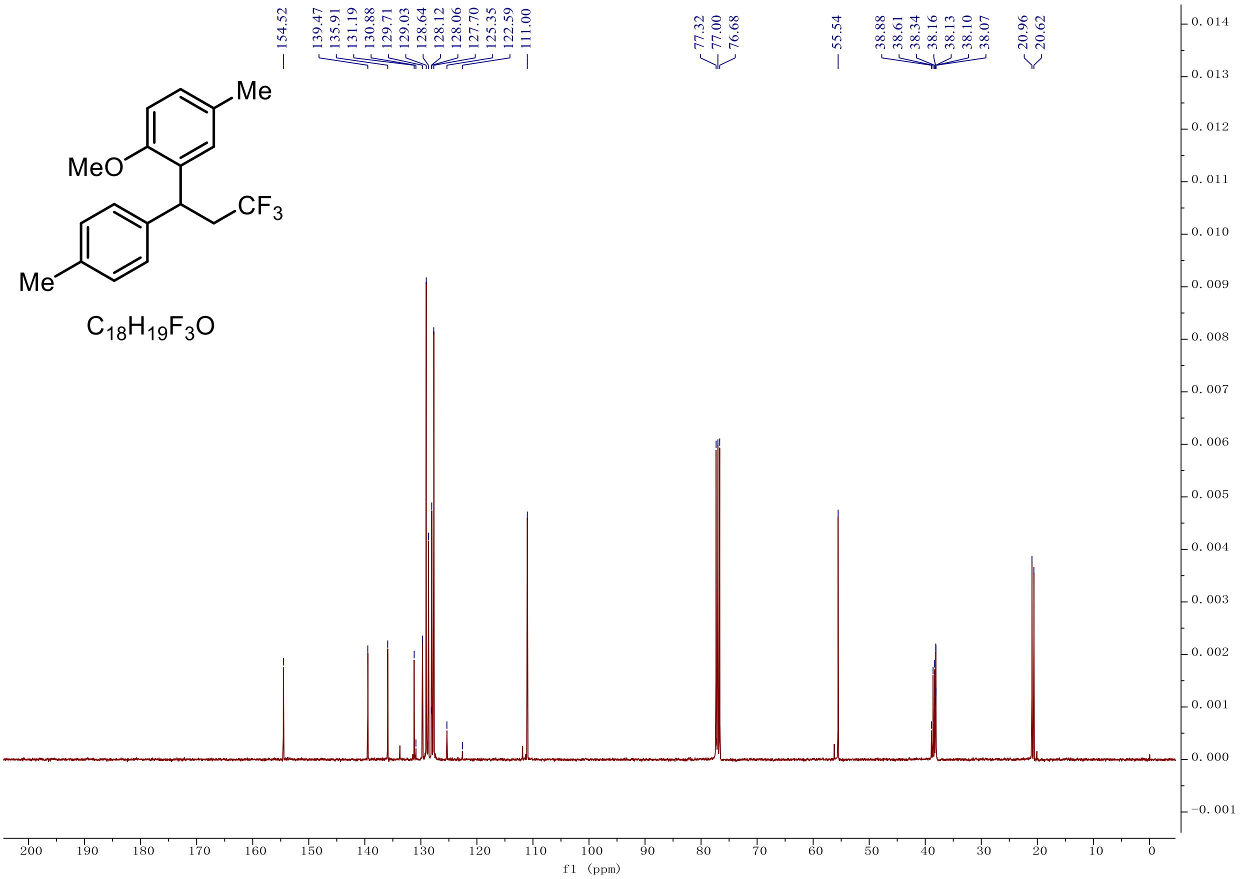

^{19}F NMR (376 MHz, $CDCl_3$) spectrum of 4k

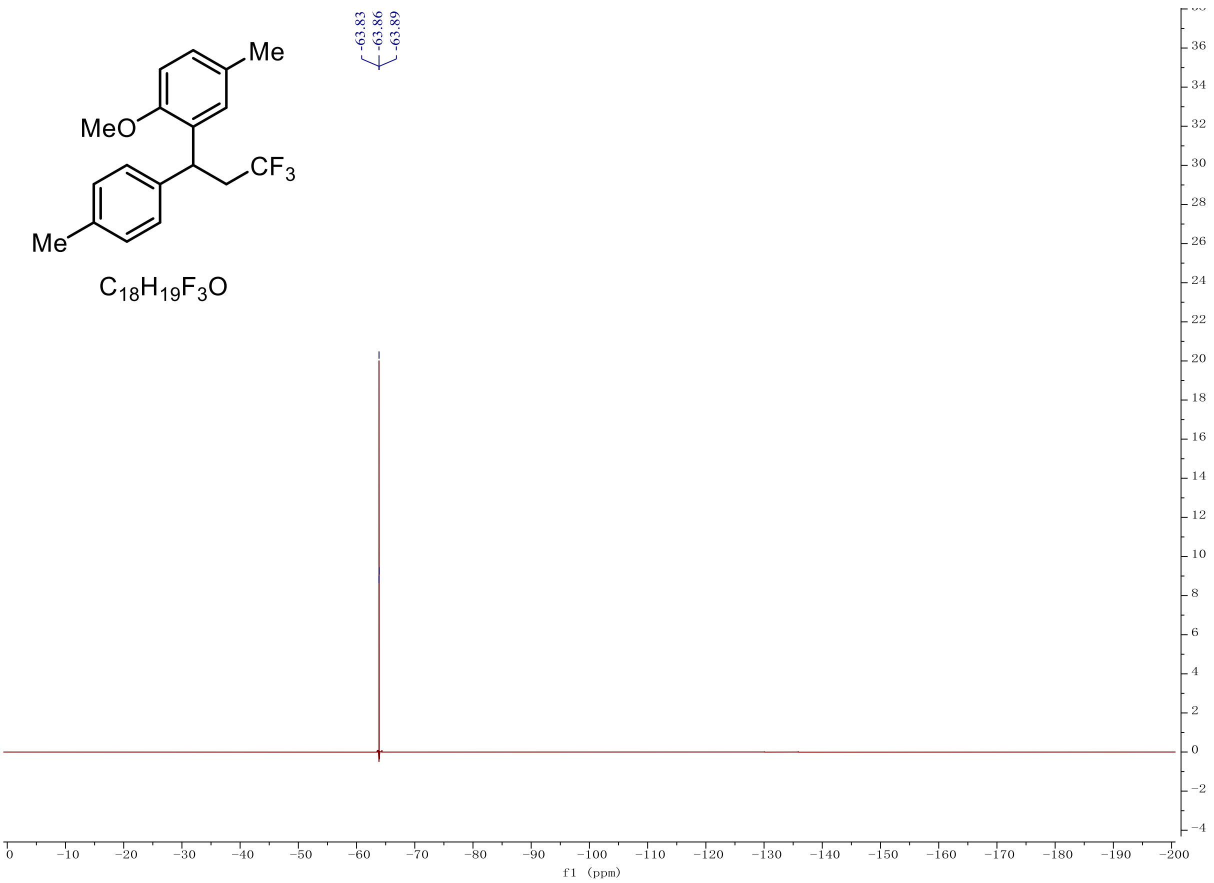

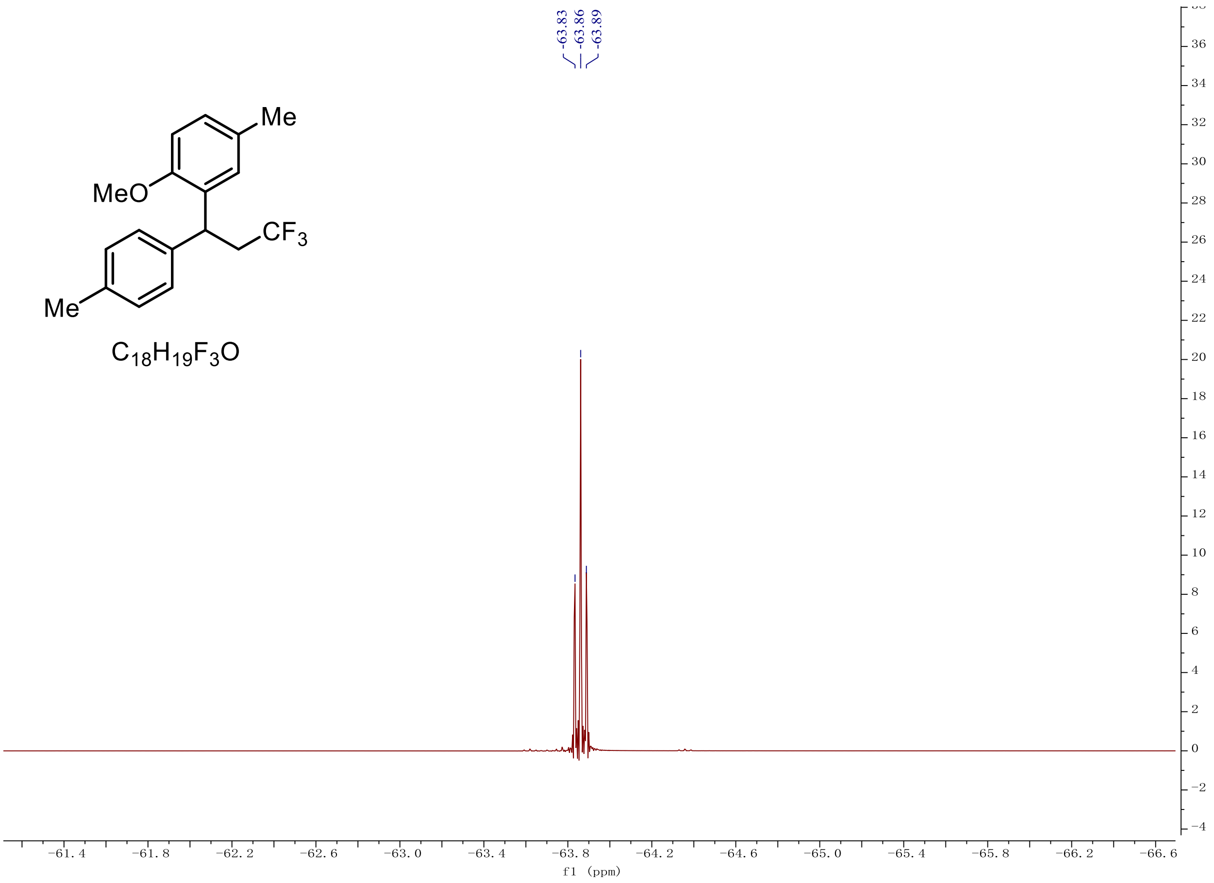

^{1}H NMR (400 MHz, $CDCl_3$) spectrum of 4l

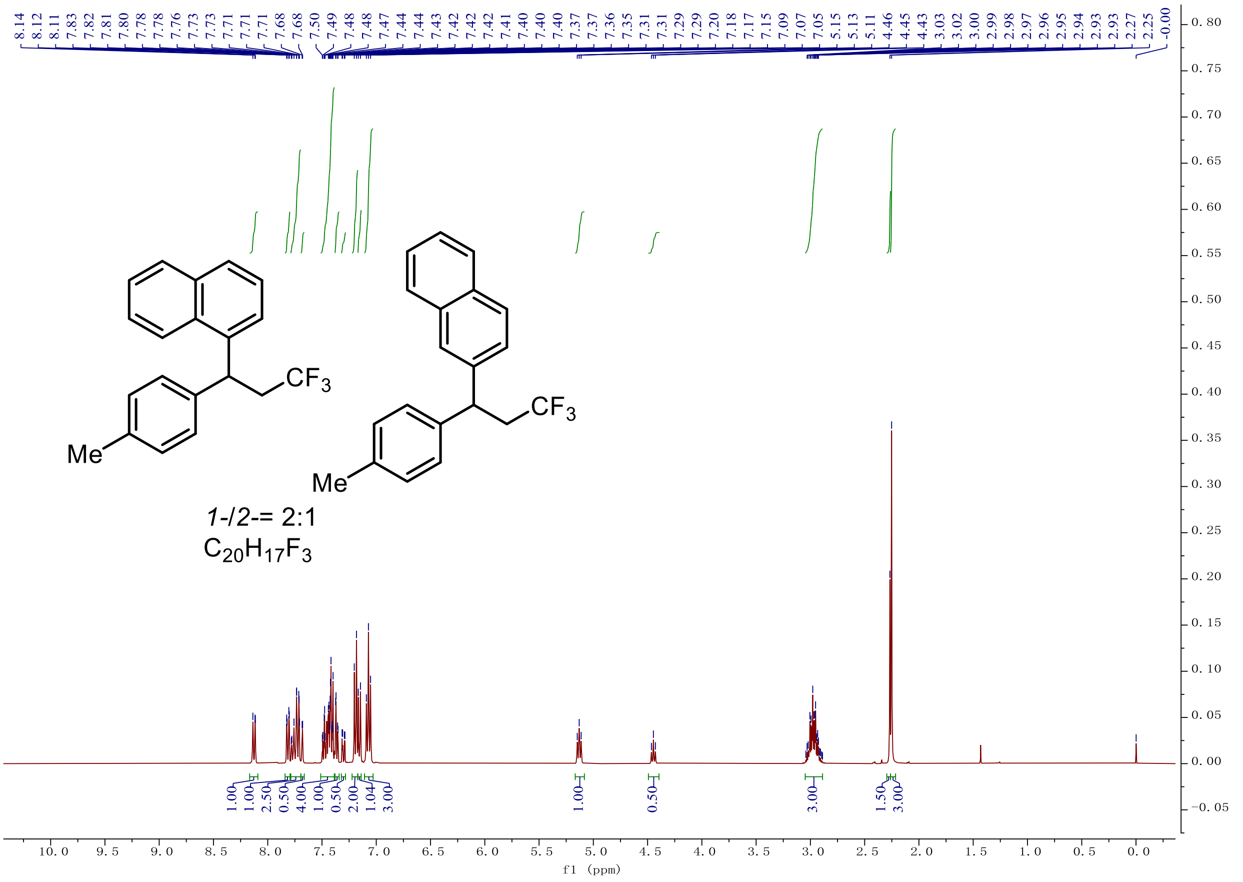

^{13}C NMR (101 MHz, $CDCl_3$) spectrum of 4l

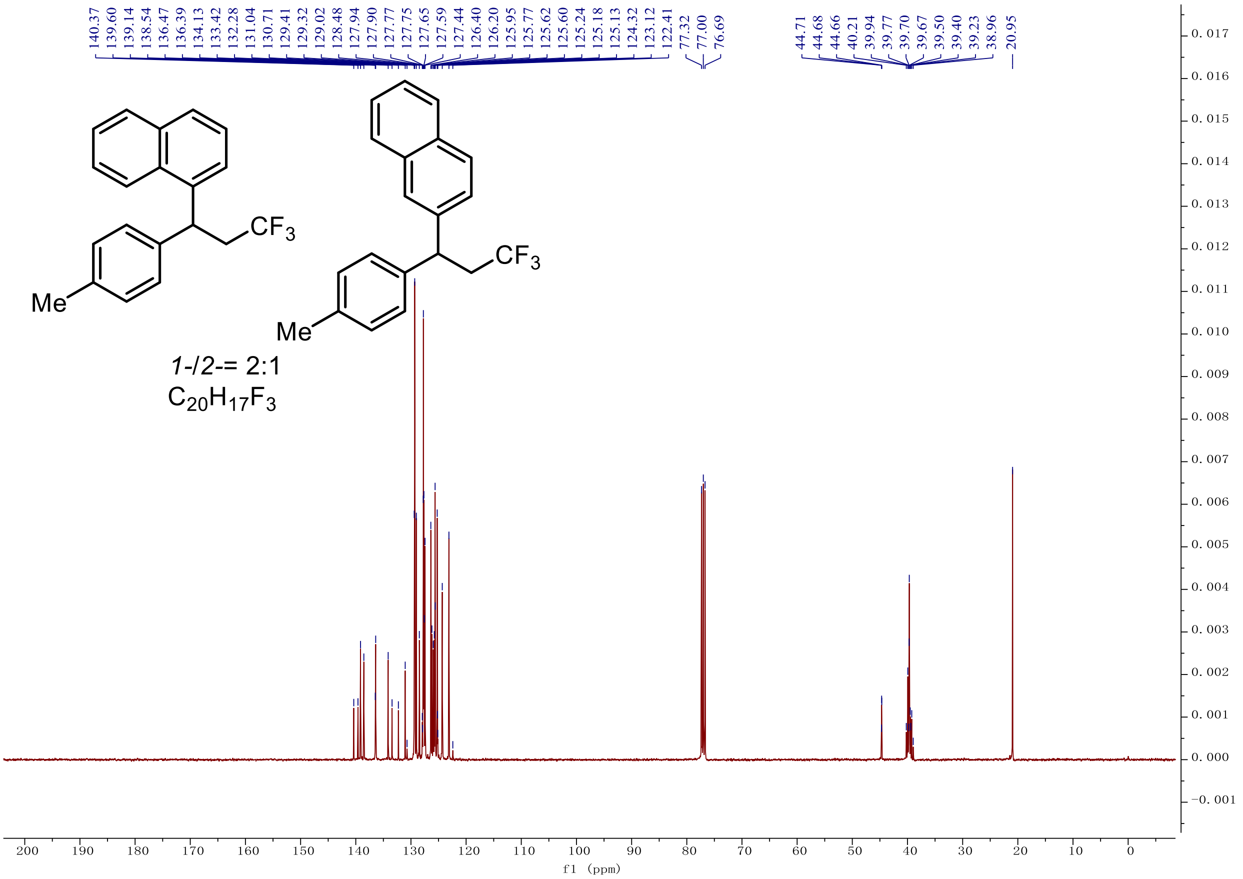

^{19}F NMR (376 MHz, $CDCl_3$) spectrum of 4l

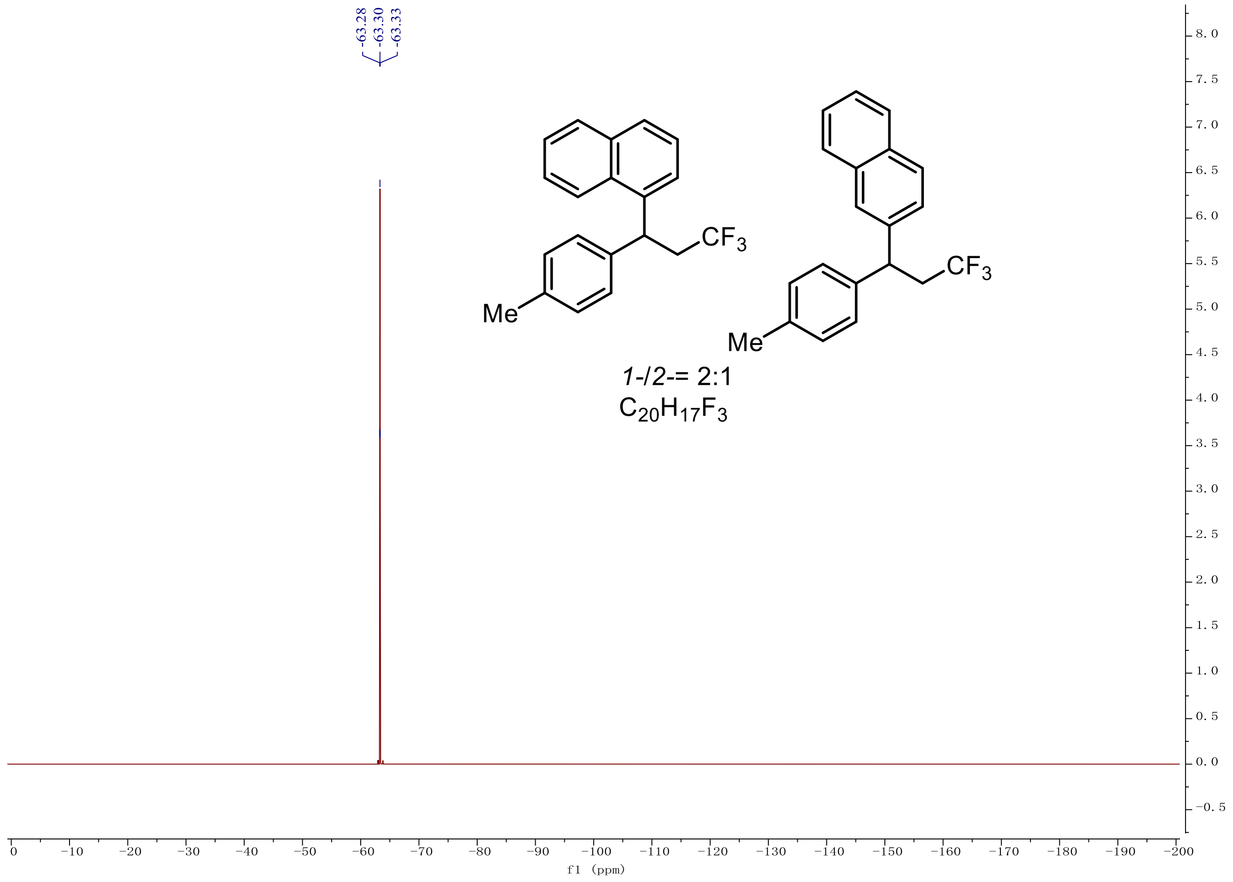

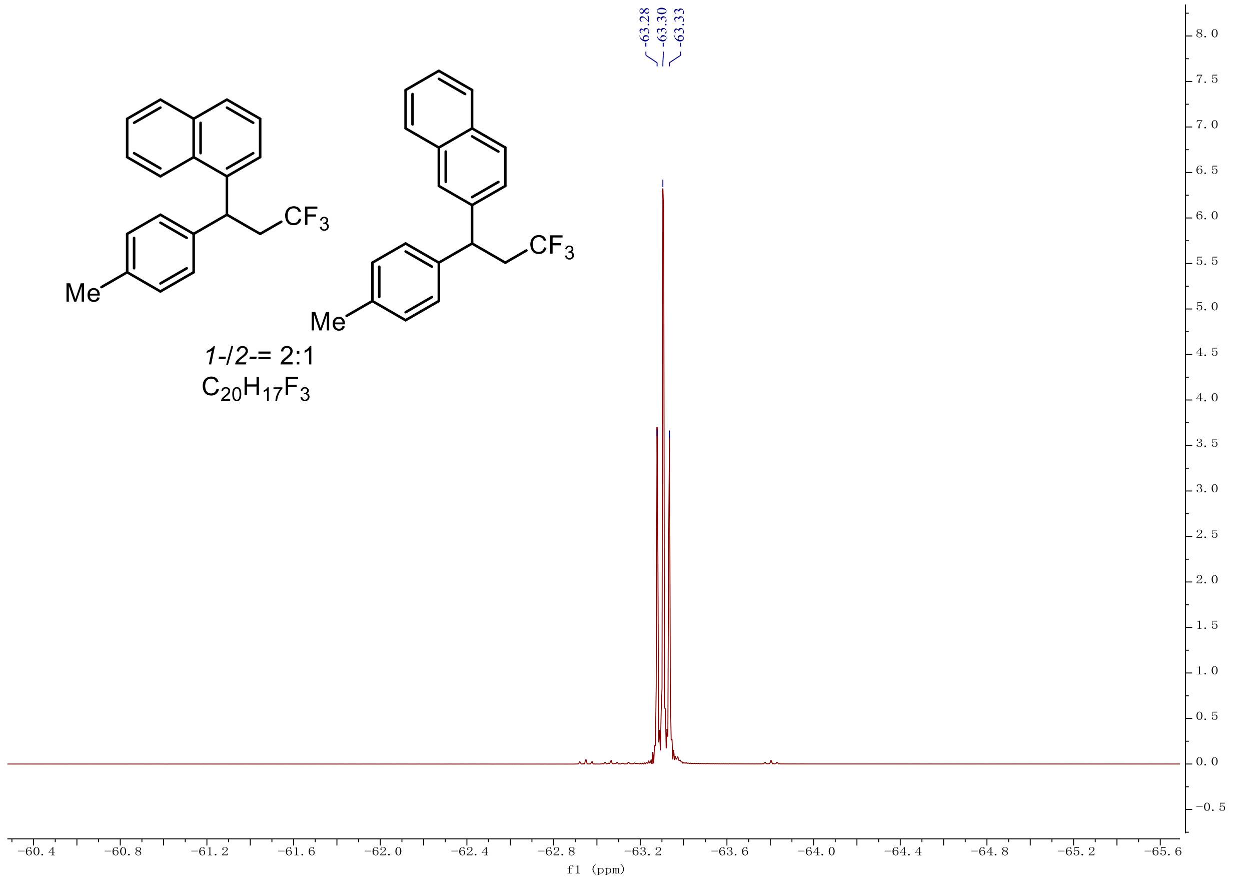

^{1}H NMR (400 MHz, CDCl3) spectrum of 4m

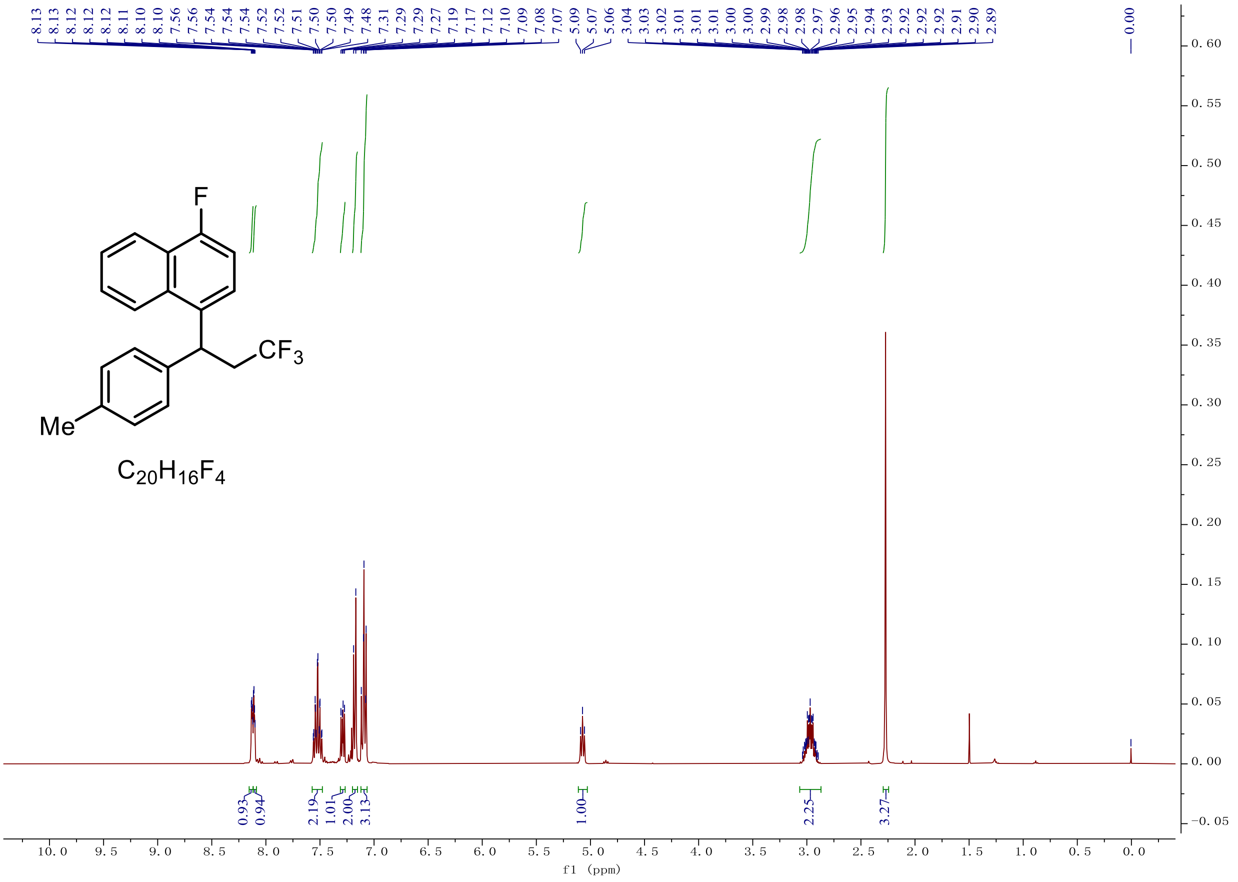

^{13}C NMR (101 MHz, CDCl3) spectrum of 4m

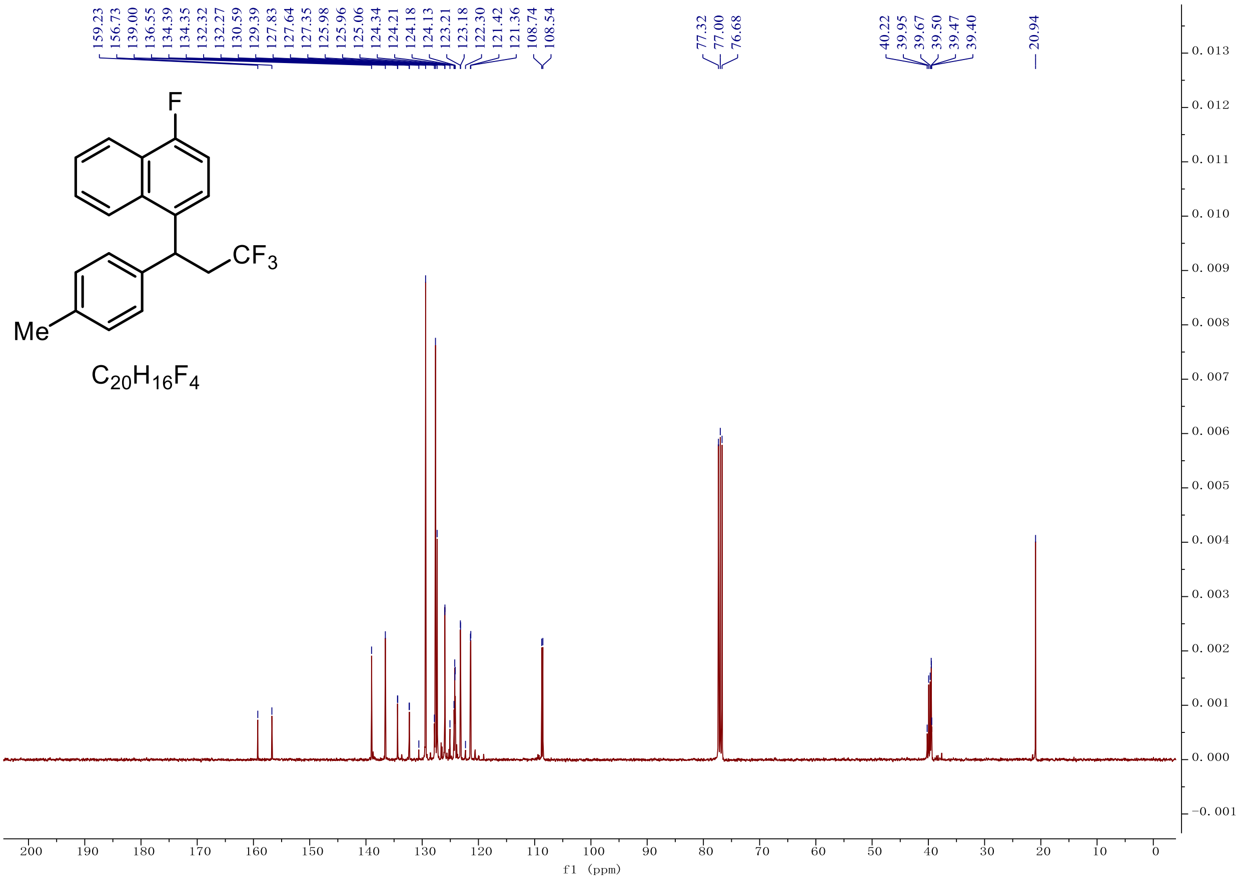

^{19}F NMR (376 MHz, CDCl3) spectrum of 4m

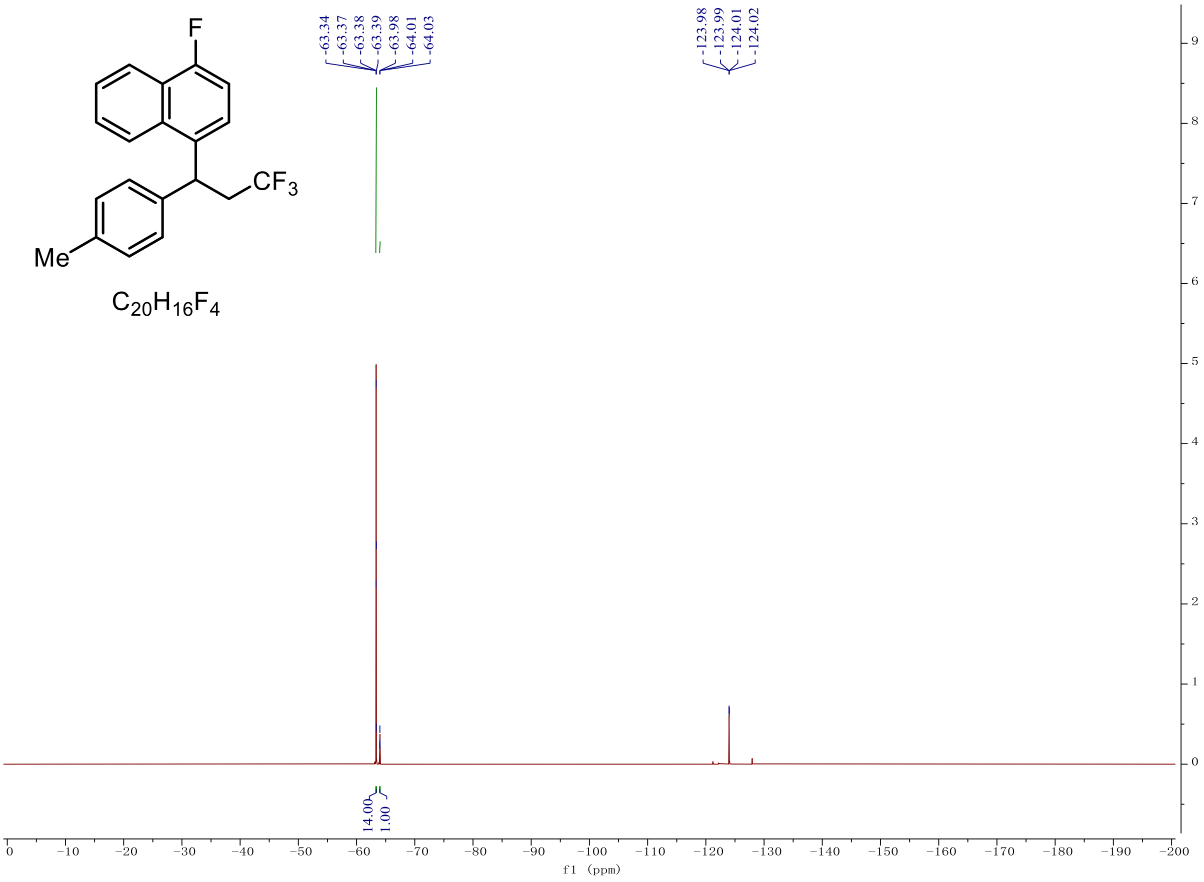

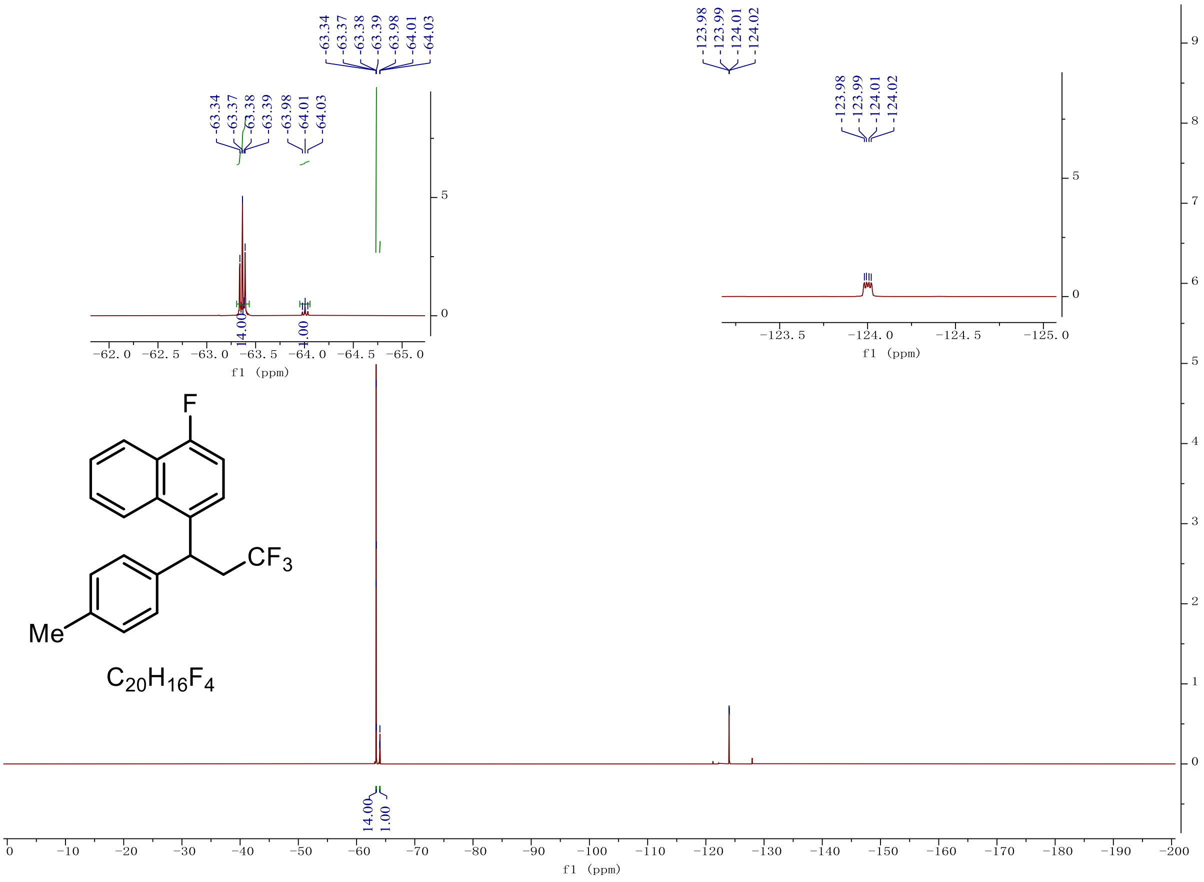

^{1}H NMR (400 MHz, $CDCl_3$) spectrum of 4n

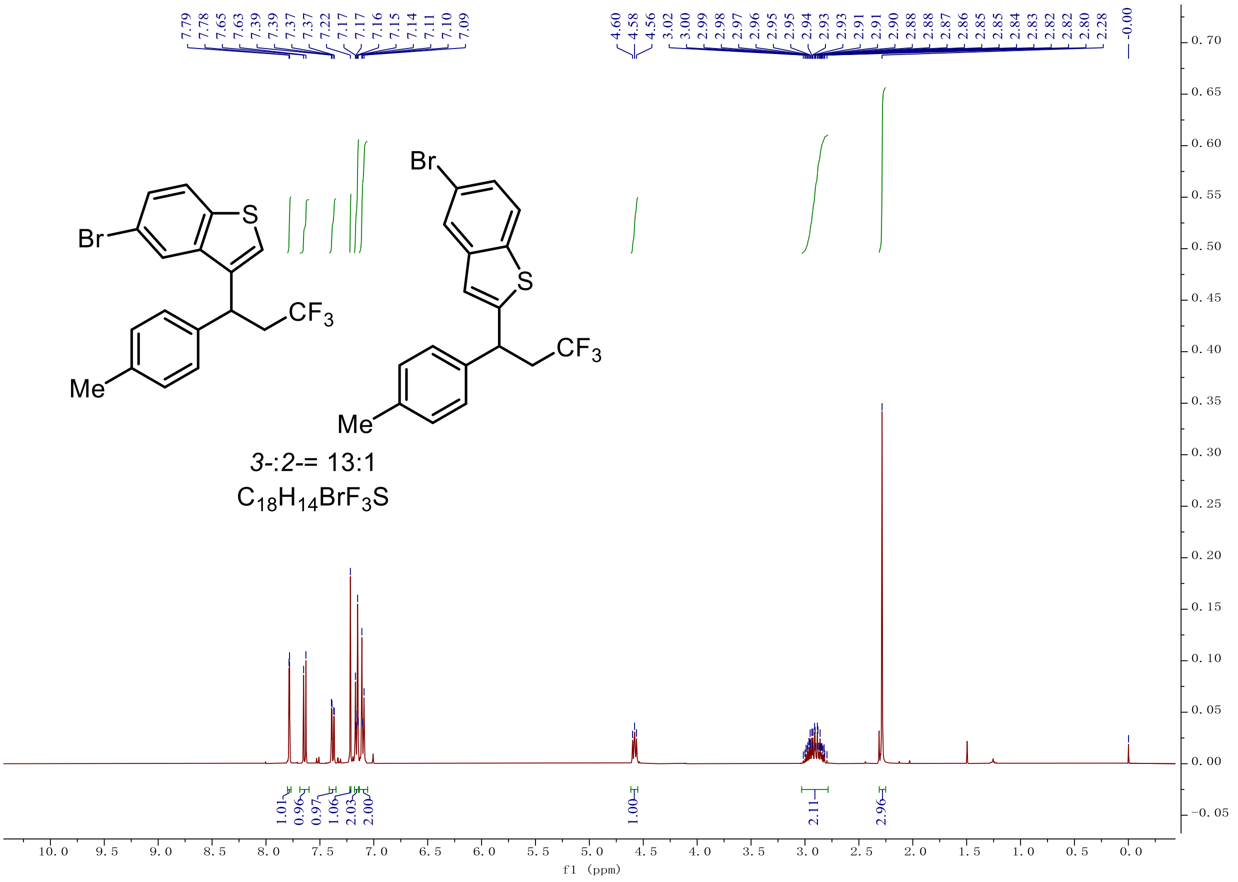

^{13}C NMR (101 MHz, $CDCl_3$) spectrum of 4n

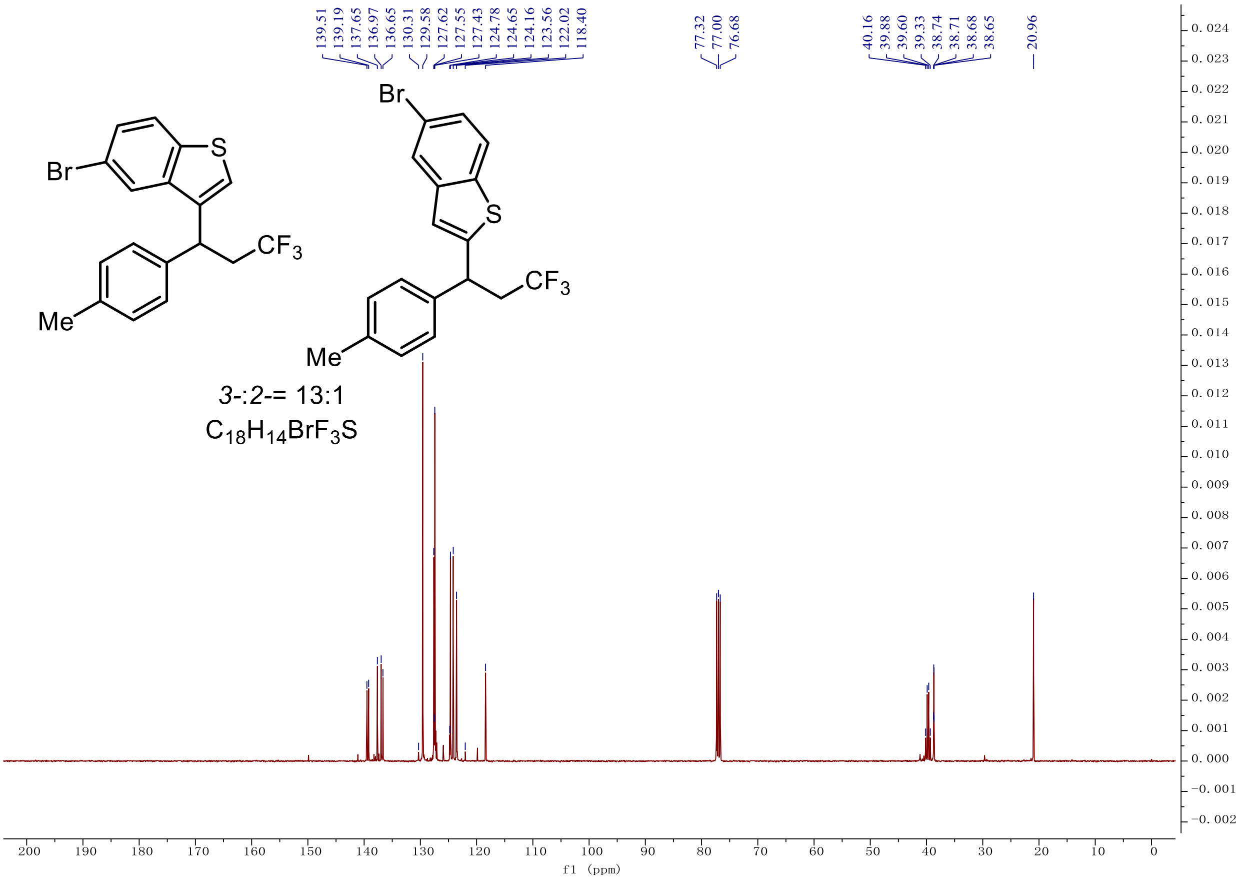

^{19}F NMR (376 MHz, CDCl3) spectrum of 4n

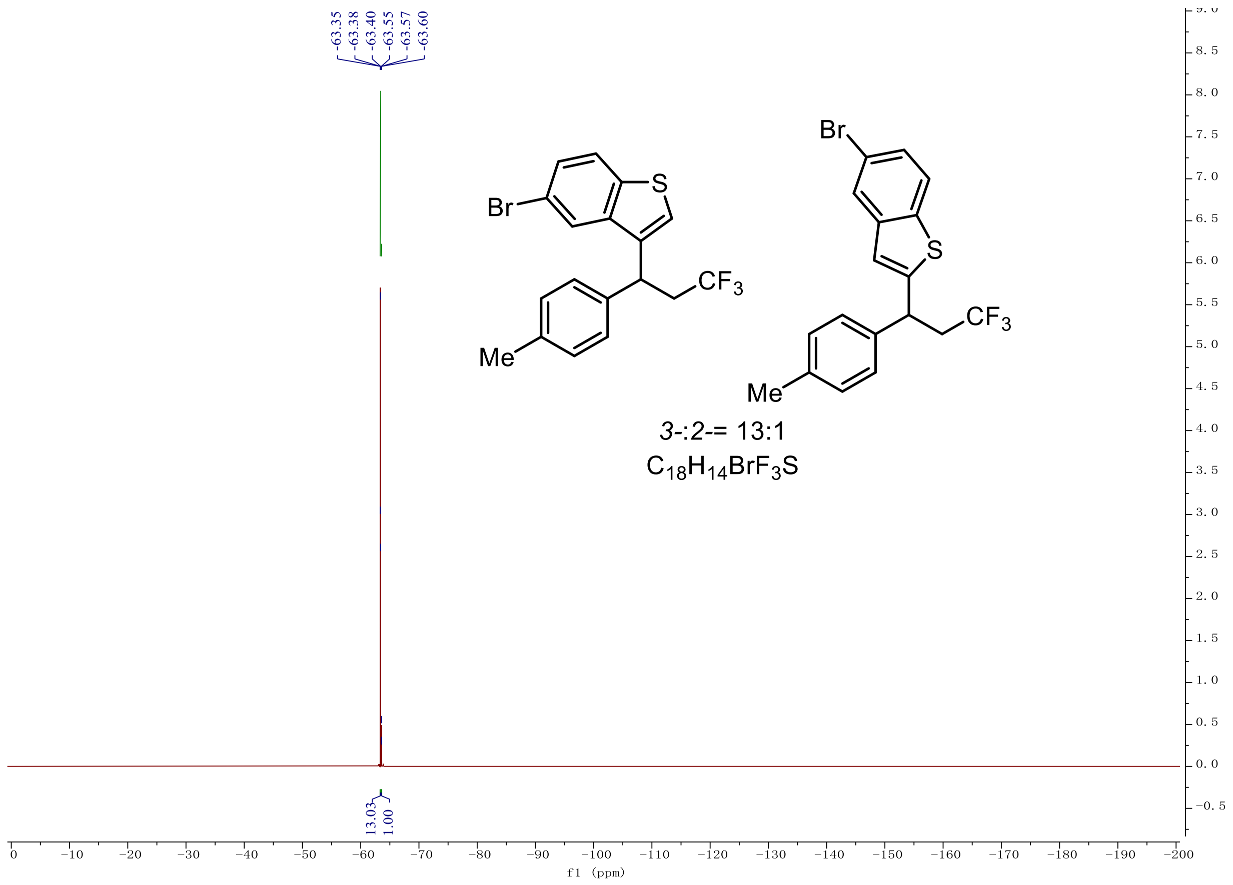

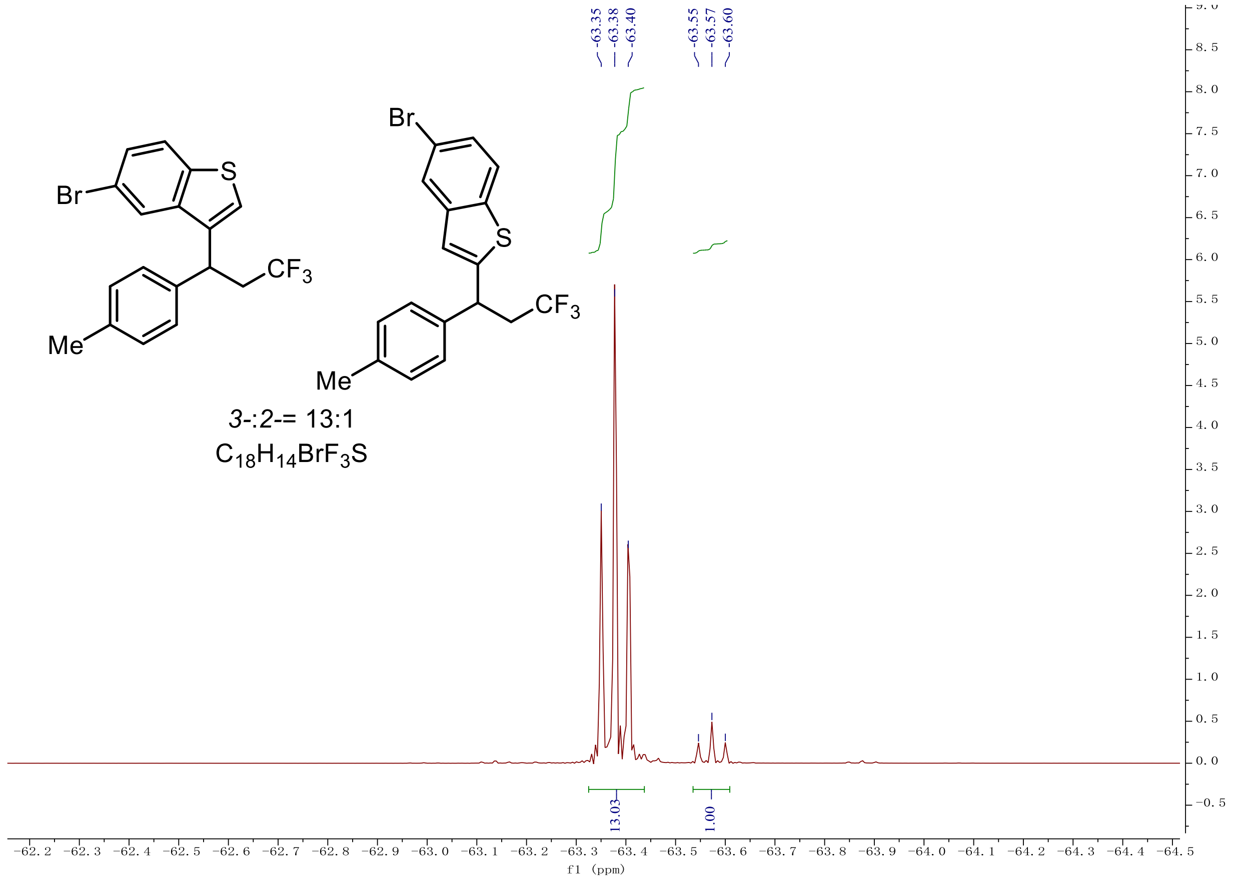

^{1}H NMR (400 MHz, $CDCl_3$) spectrum of 4o

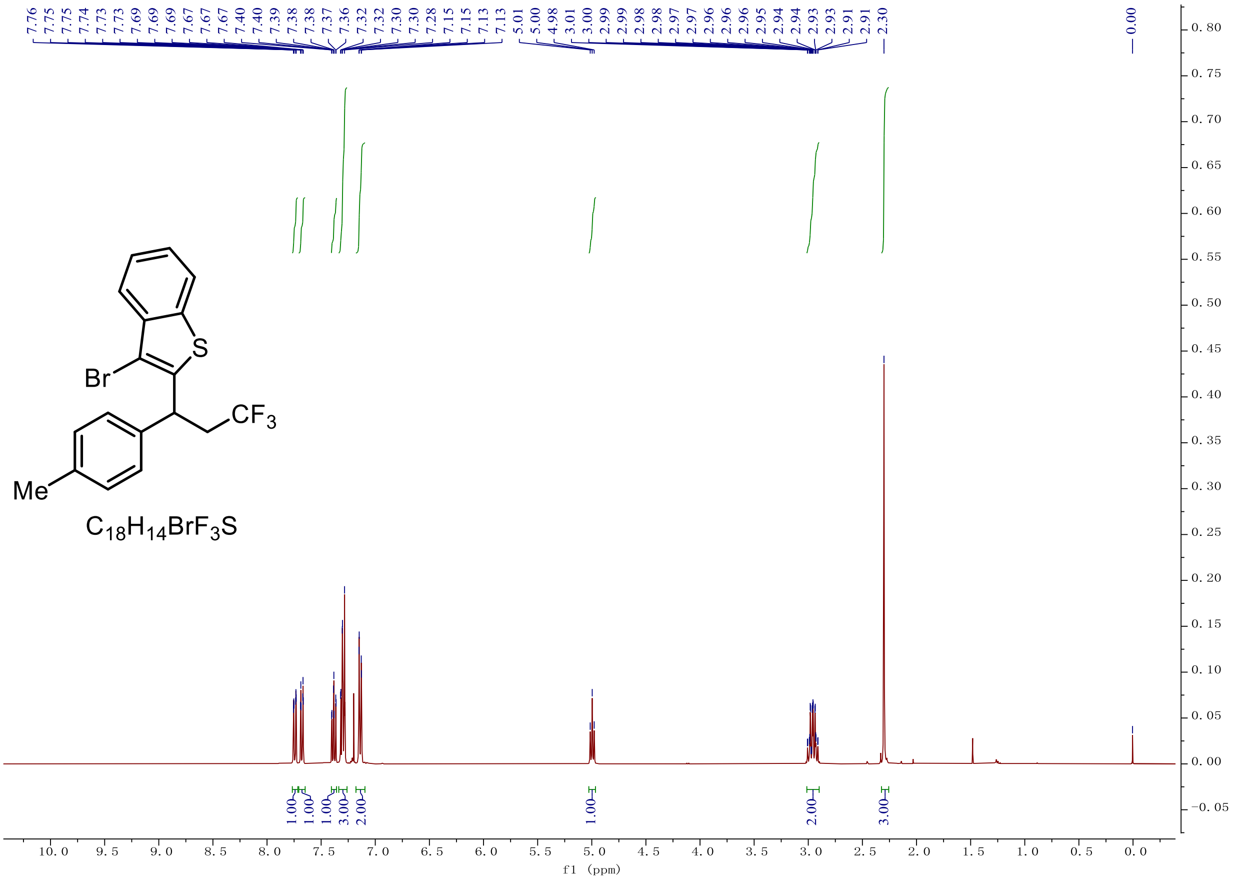

^{13}C NMR (101 MHz, $CDCl_3$) spectrum of 4o

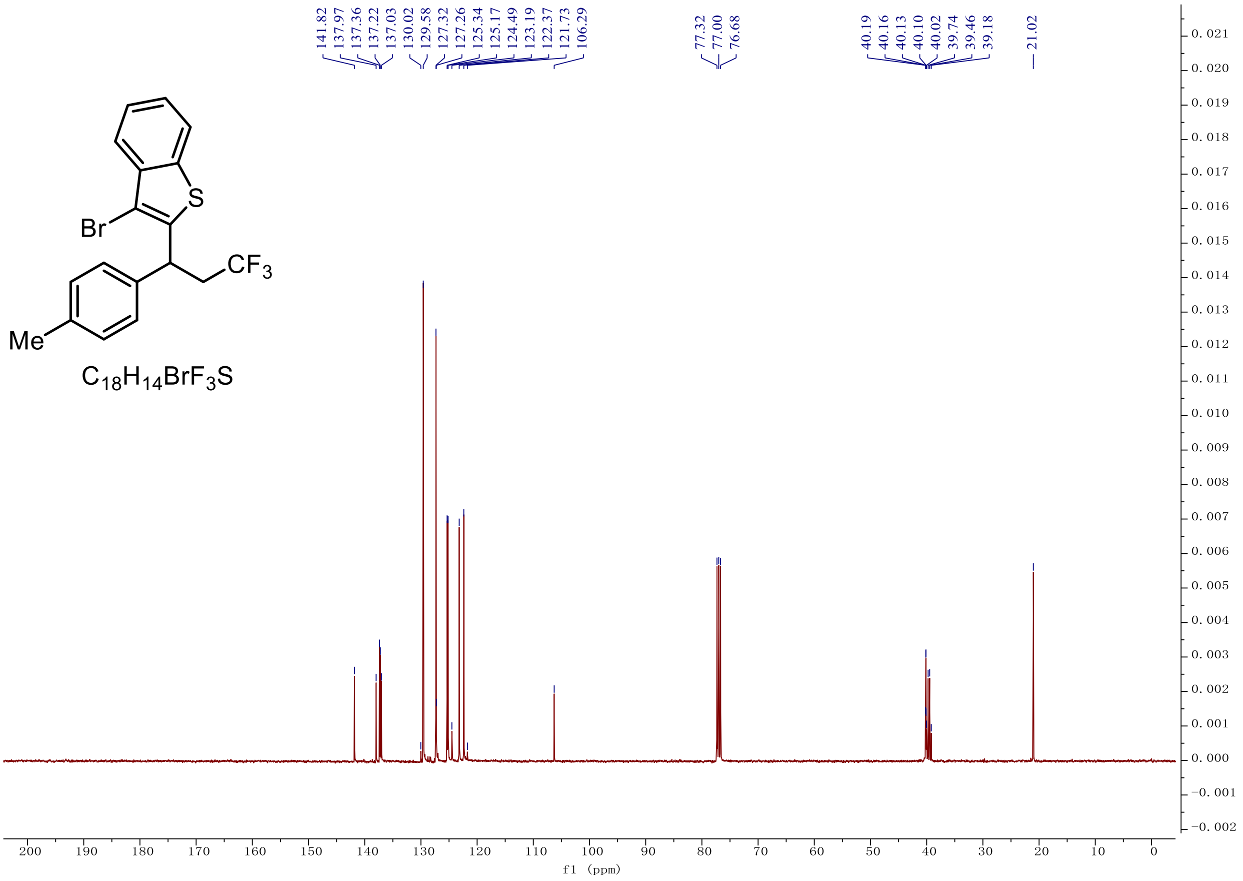

^{19}F NMR (376 MHz, $CDCl_3$) spectrum of 4o

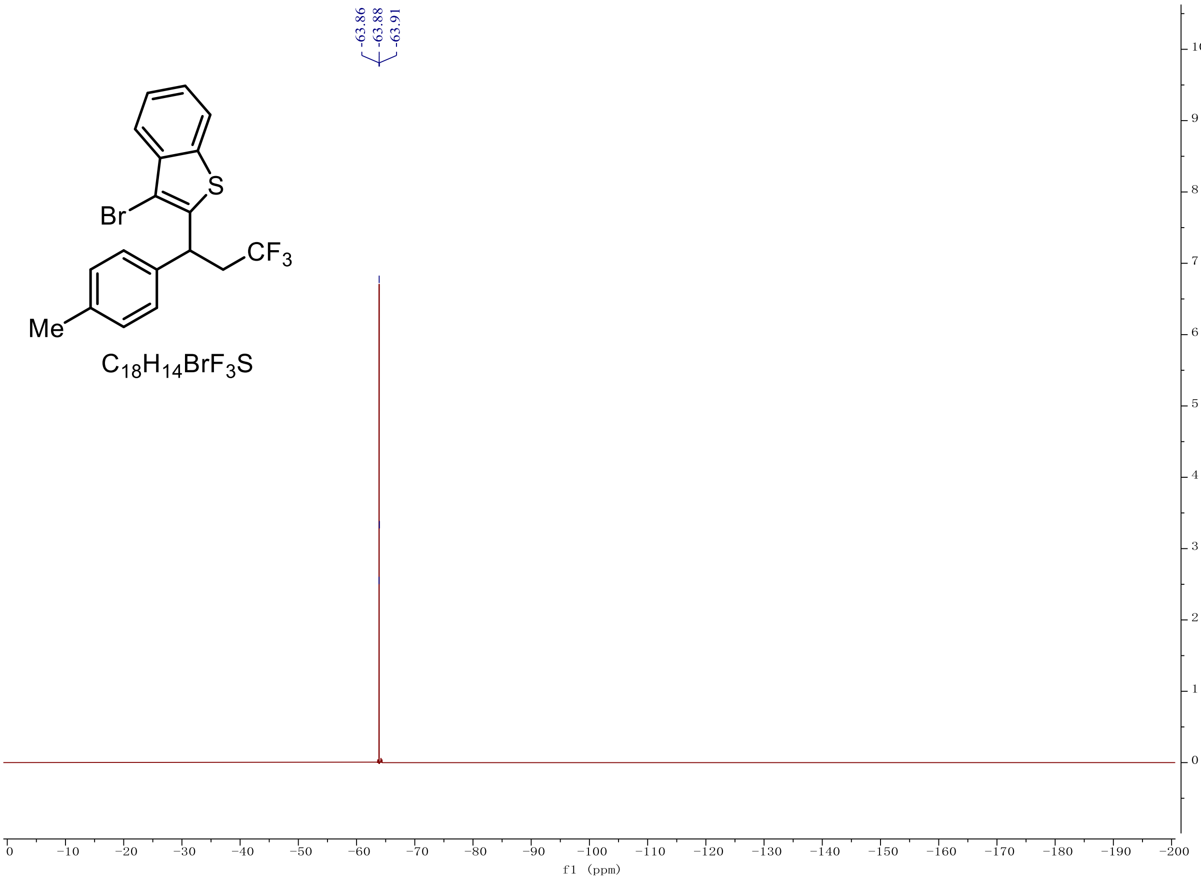

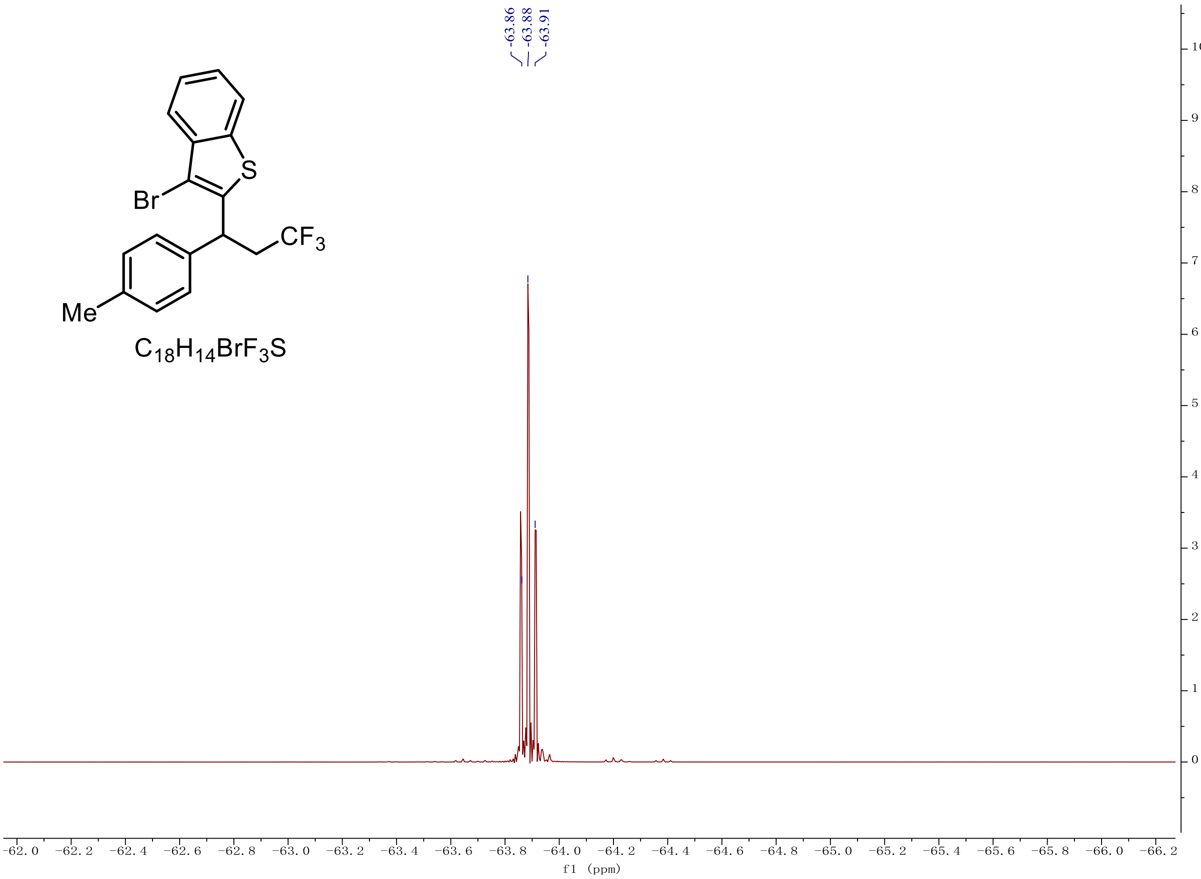

^{1}H NMR (400 MHz, CDCl$_3$) spectrum of 5a

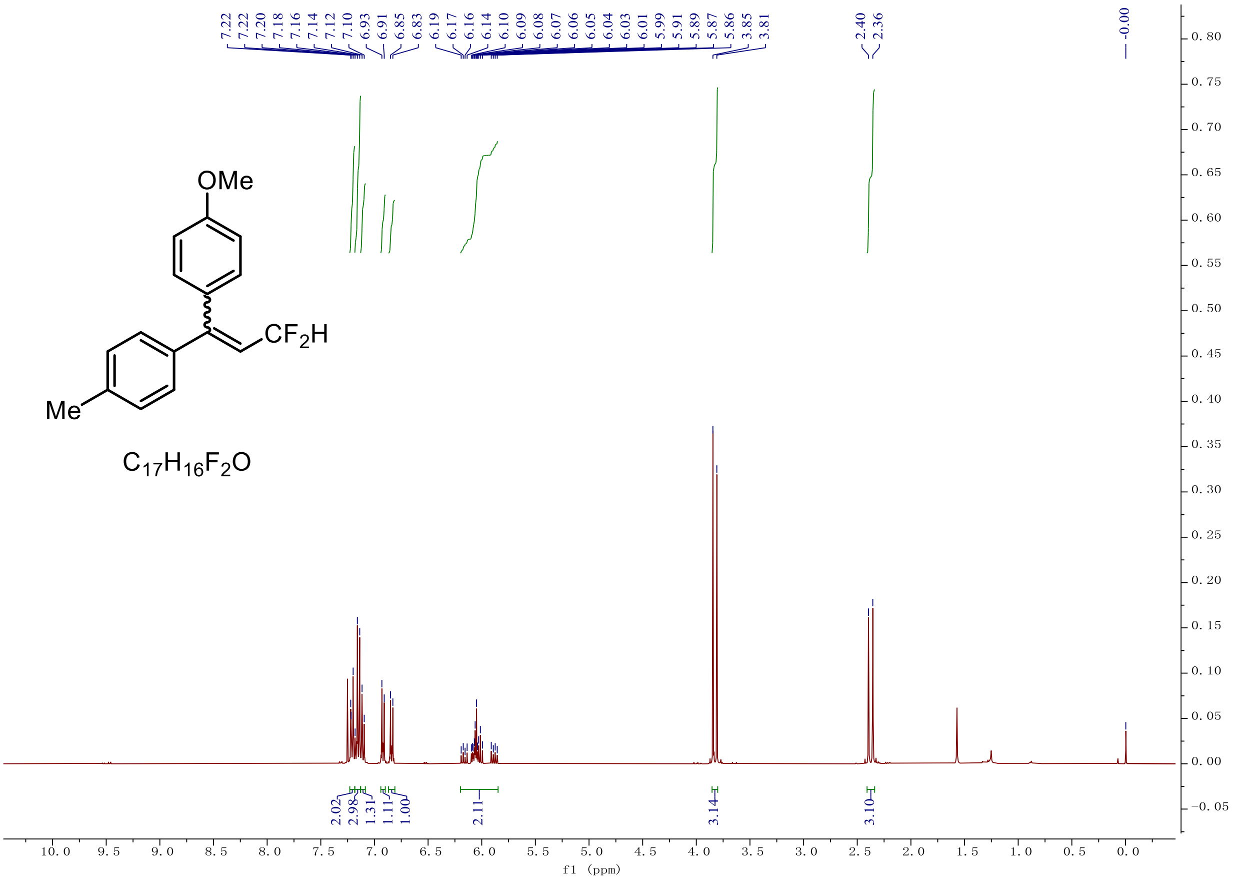

^{13}C NMR (101 MHz, CDCl$_3$) spectrum of 5a

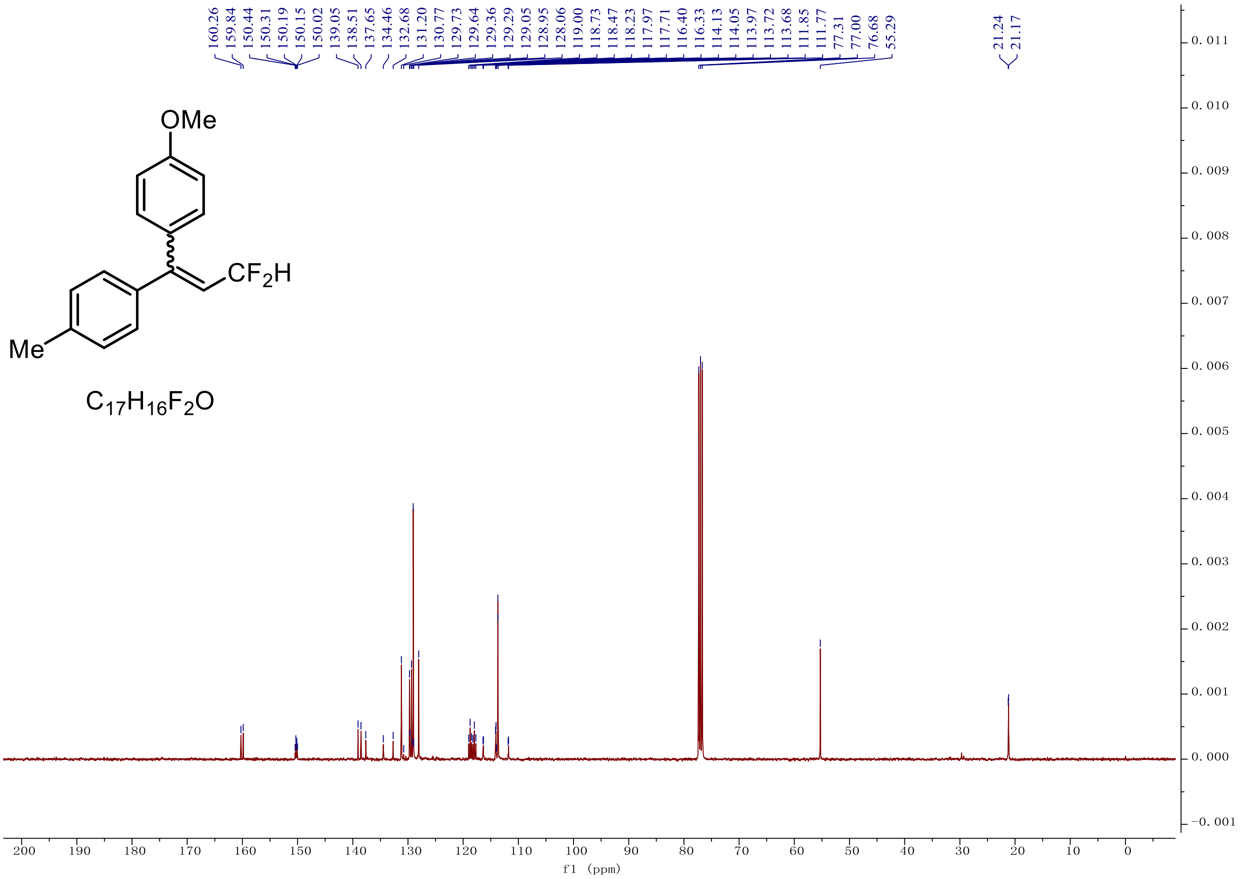

^{19}F NMR (376 MHz, $CDCl_3$) spectrum of 5a

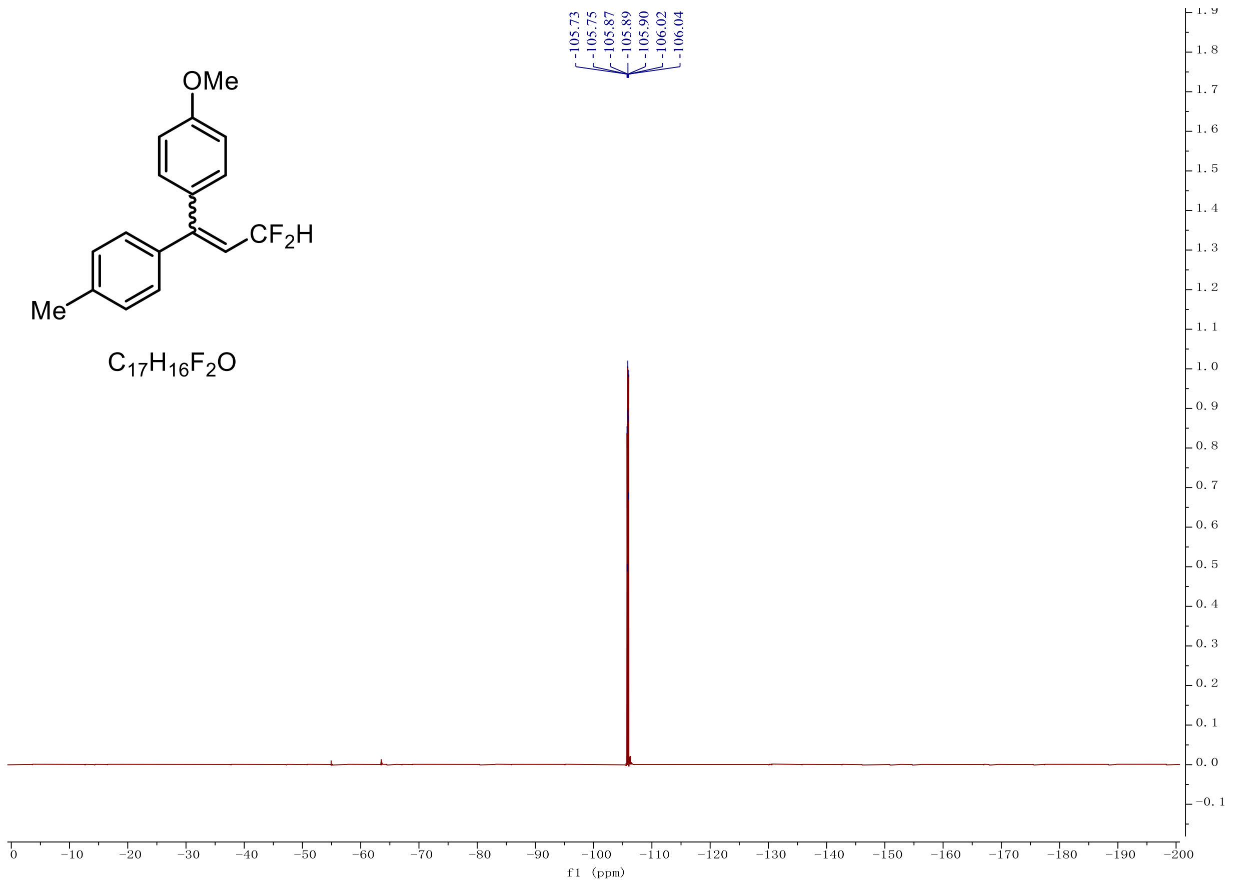

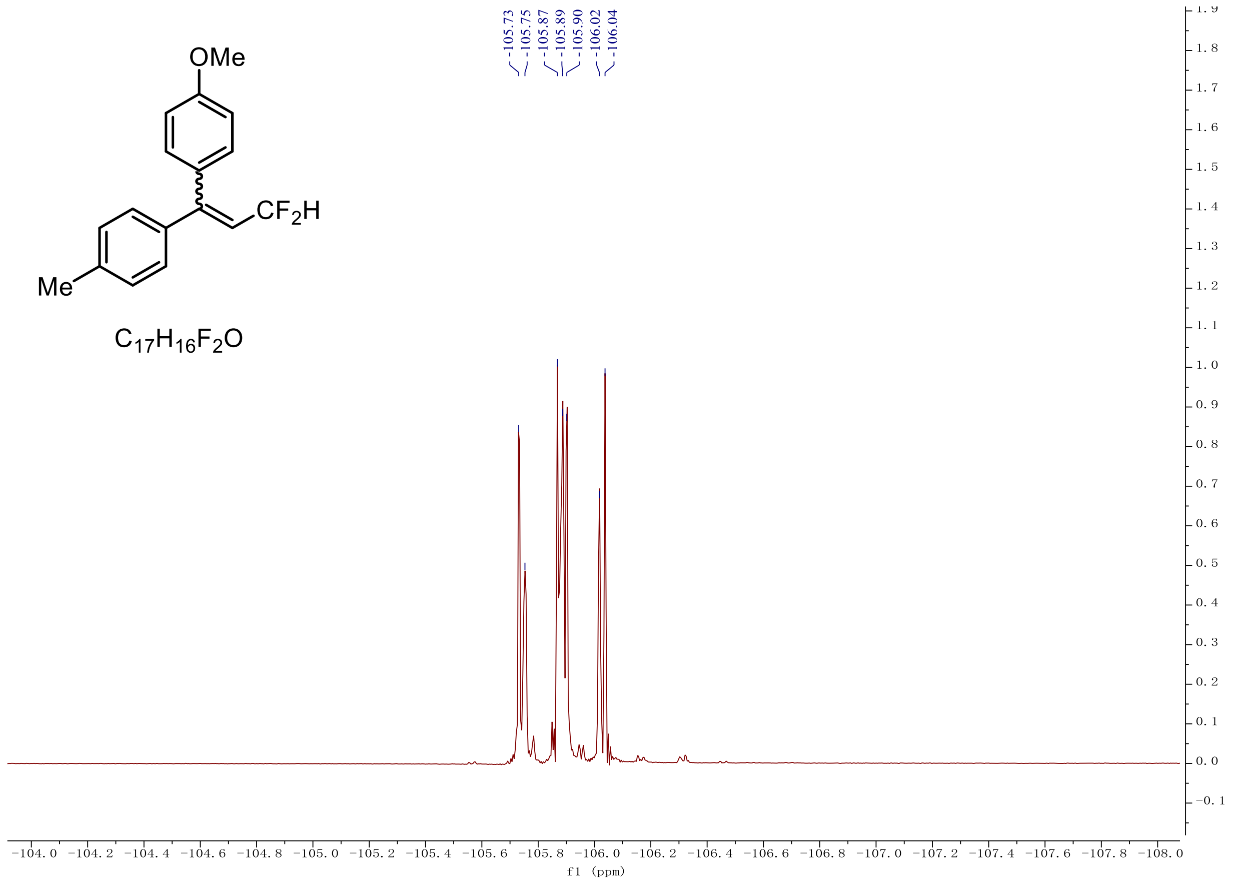

^{1}H NMR (400 MHz, $CDCl_3$) spectrum of 5b

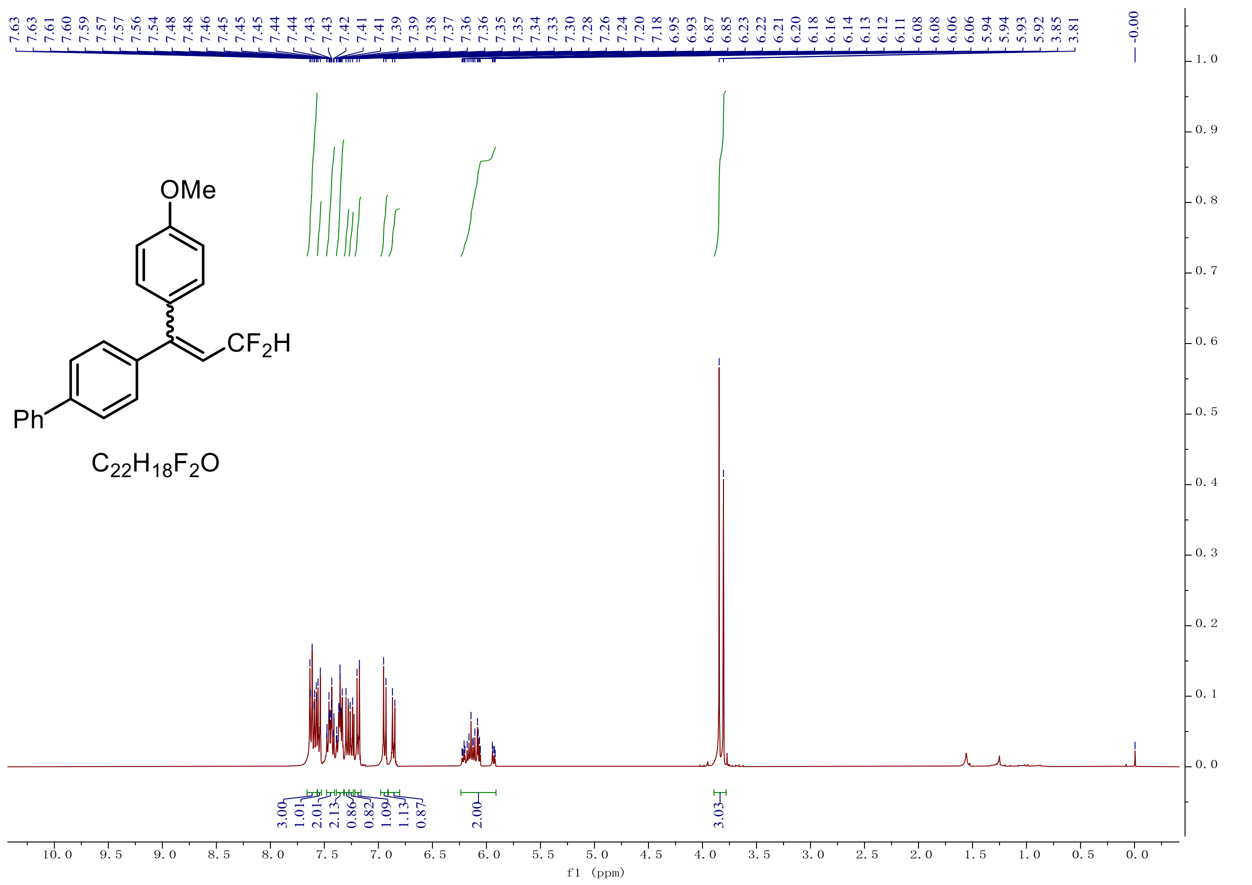

^{13}C NMR (101 MHz, $CDCl_3$) spectrum of 5b

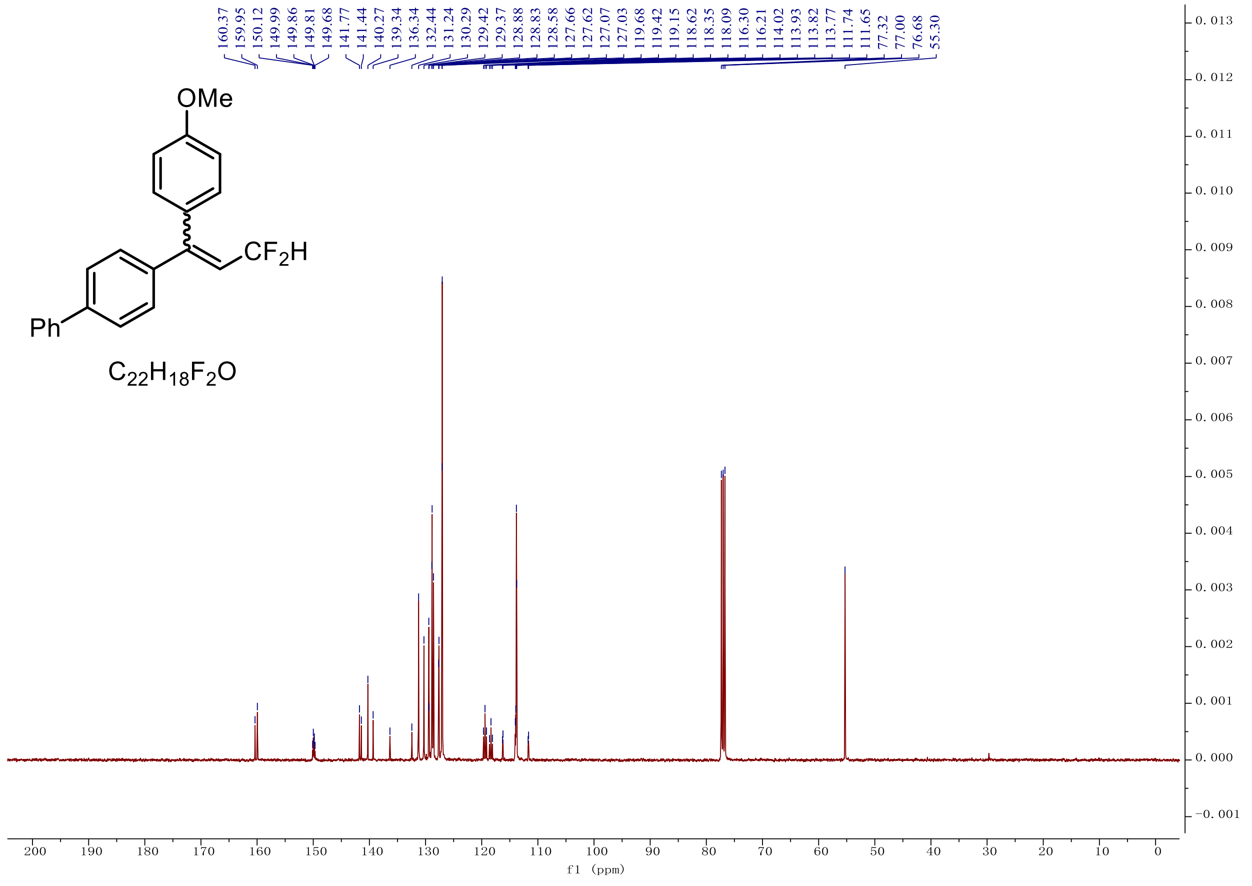

^{19}F NMR (376 MHz, $CDCl_3$) spectrum of 5b

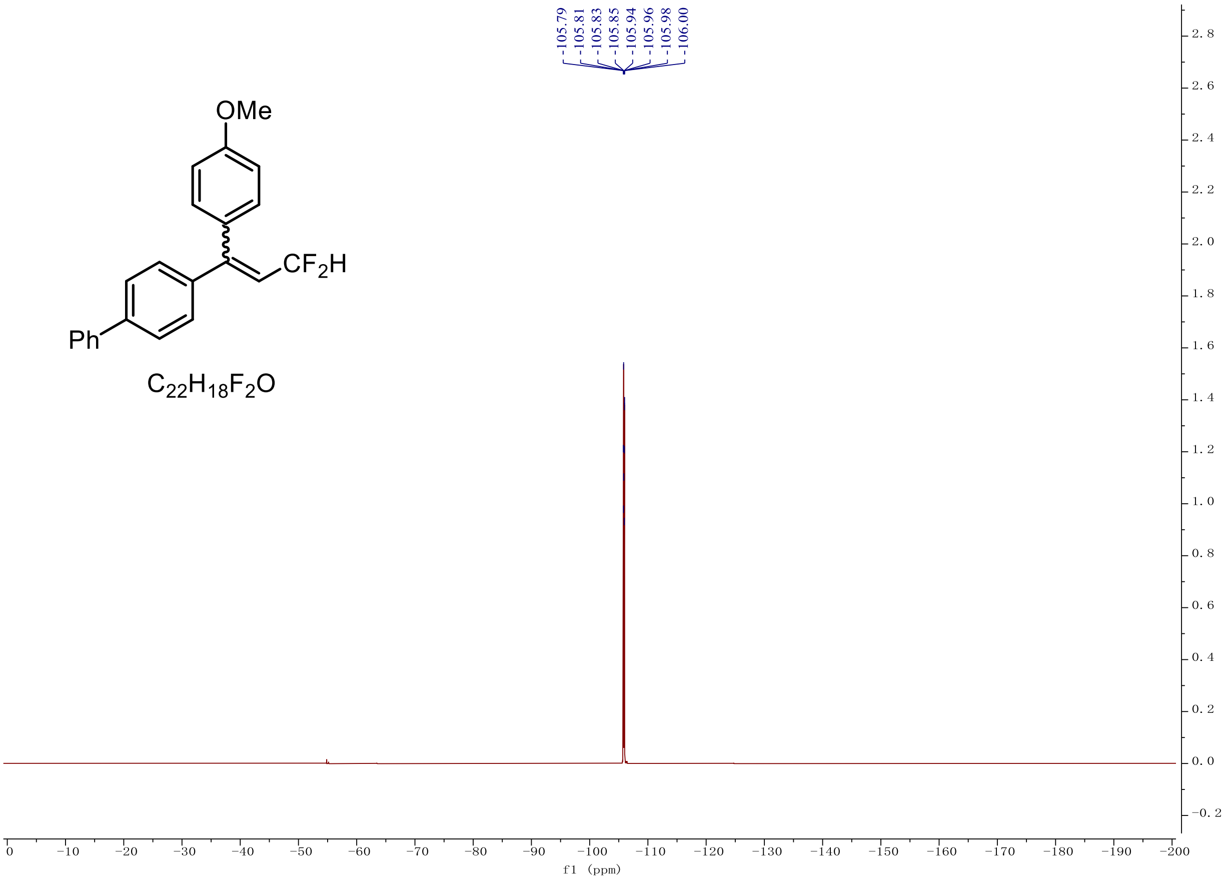

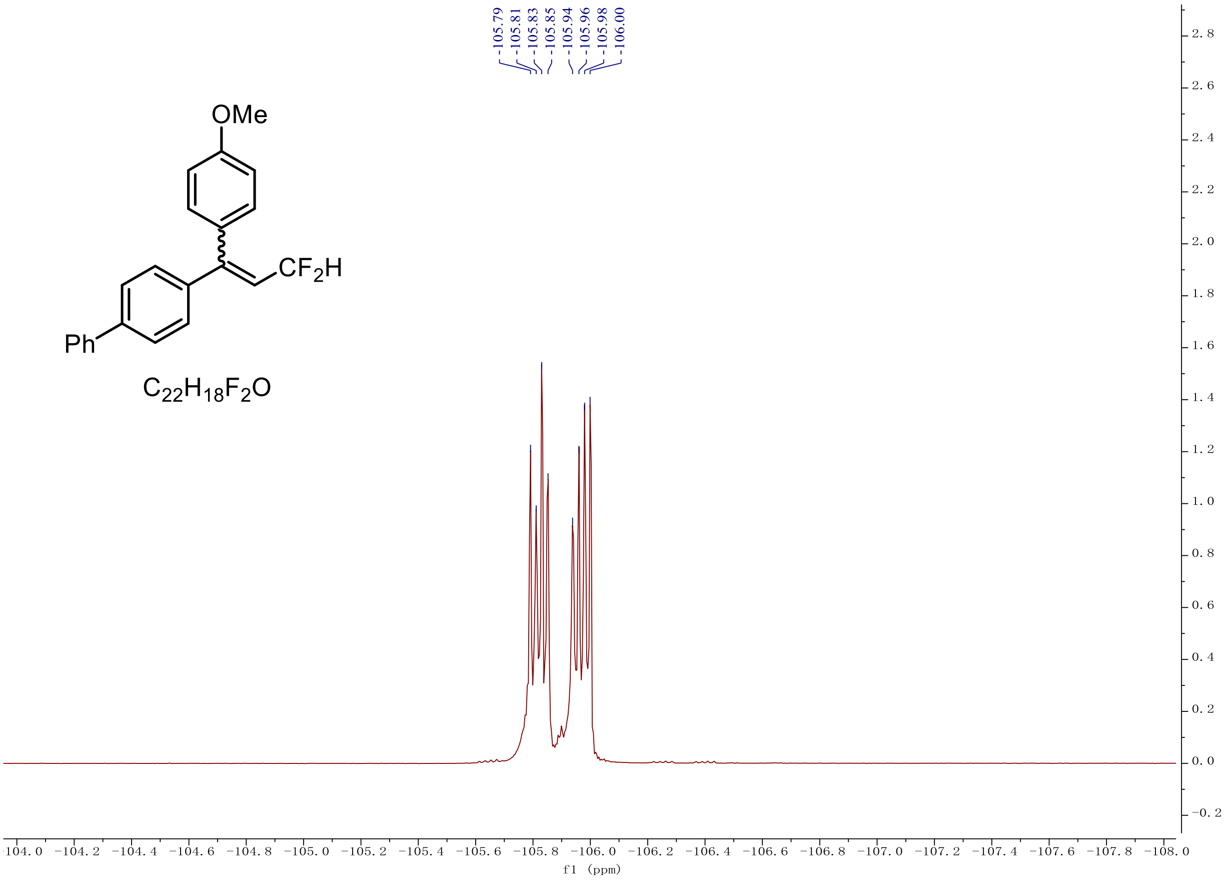

^{1}H NMR (400 MHz, $CDCl_3$) spectrum of 5c

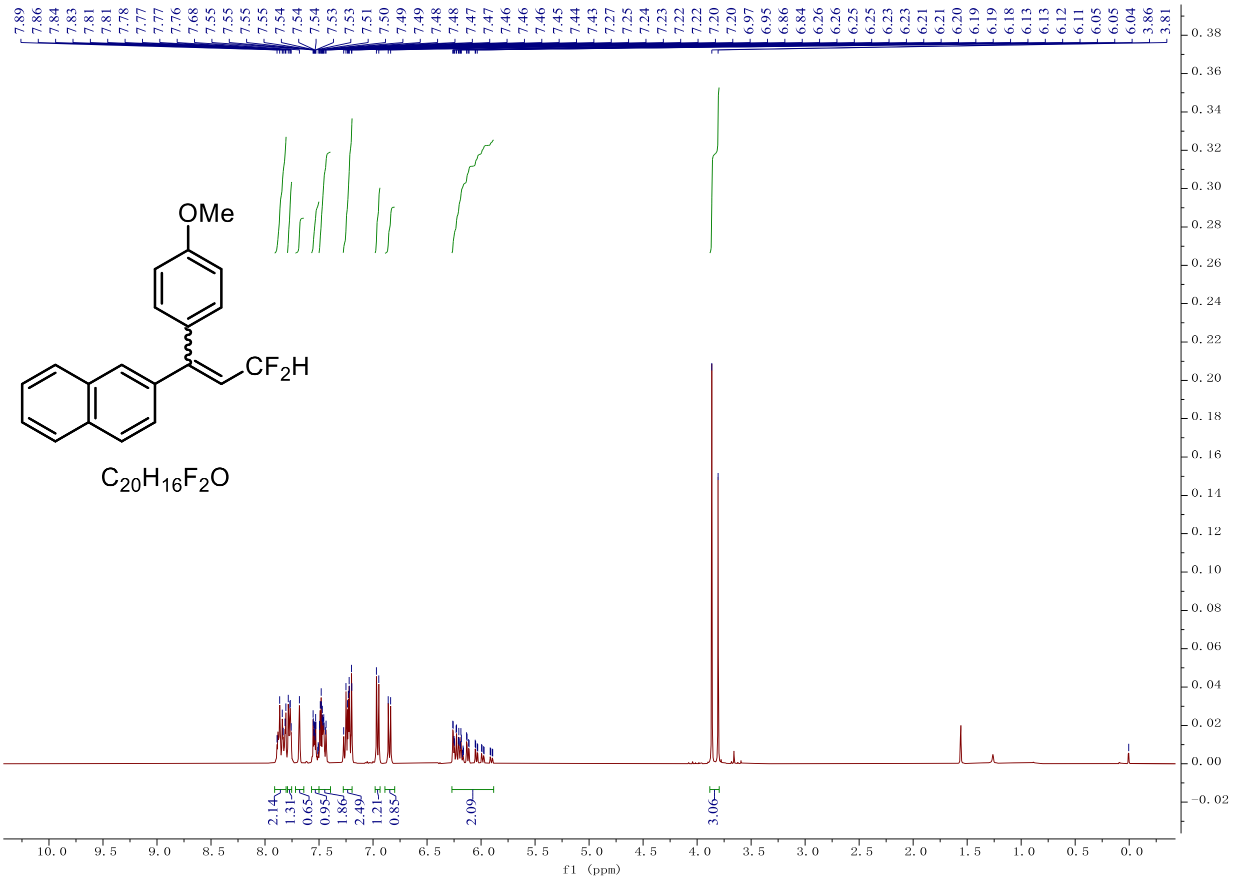

^{13}C NMR (101 MHz, $CDCl_3$) spectrum of 5c

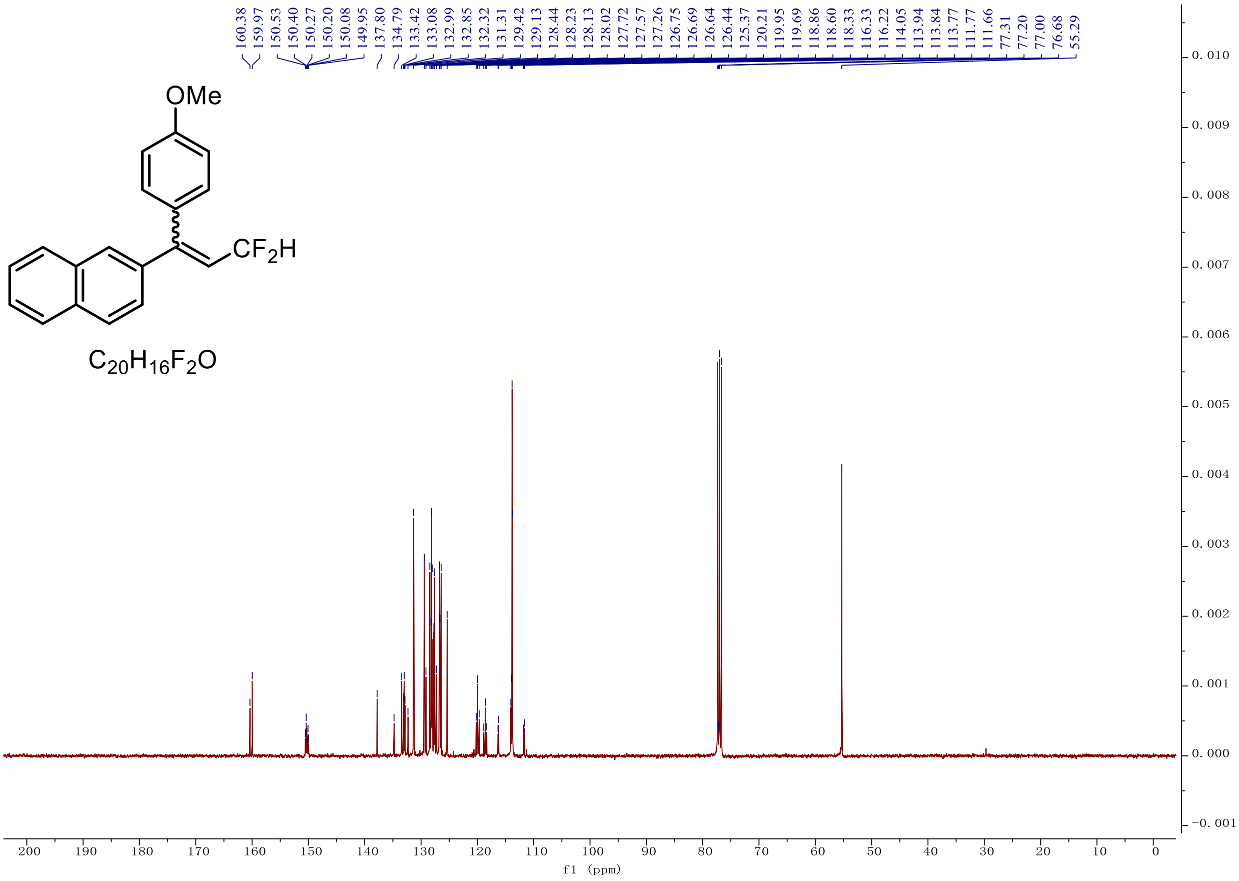

^{19}F NMR (376 MHz, $CDCl_3$) spectrum of 5c

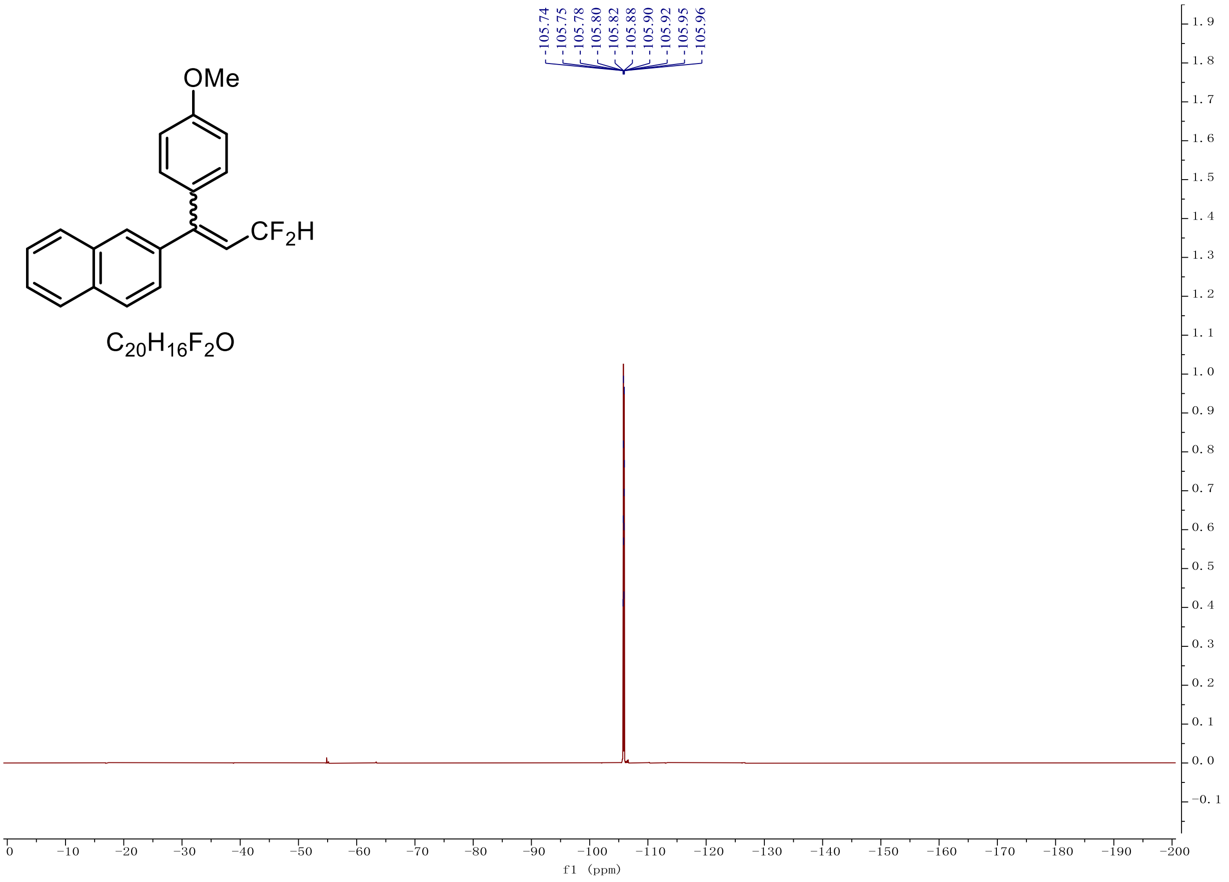

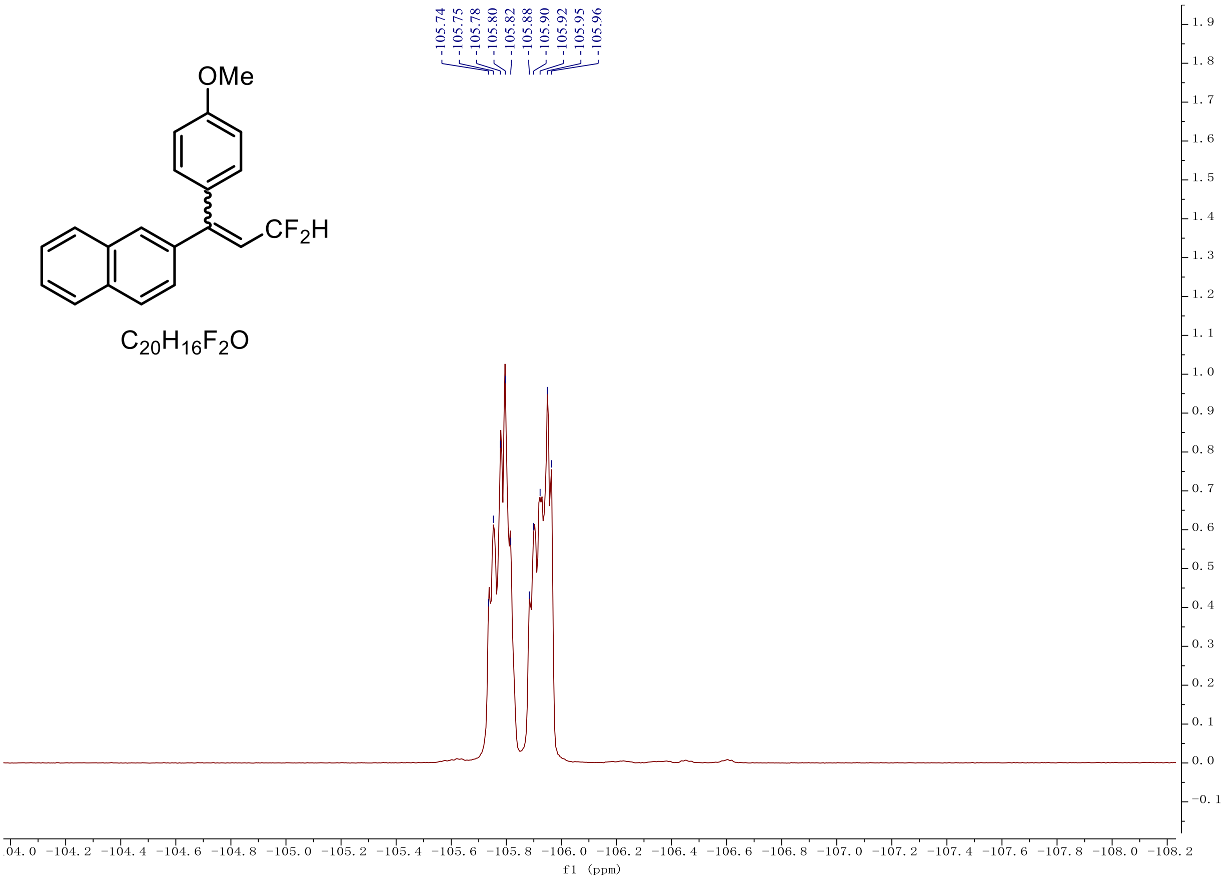

^{1}H NMR (400 MHz, CDCl3) spectrum of 5d

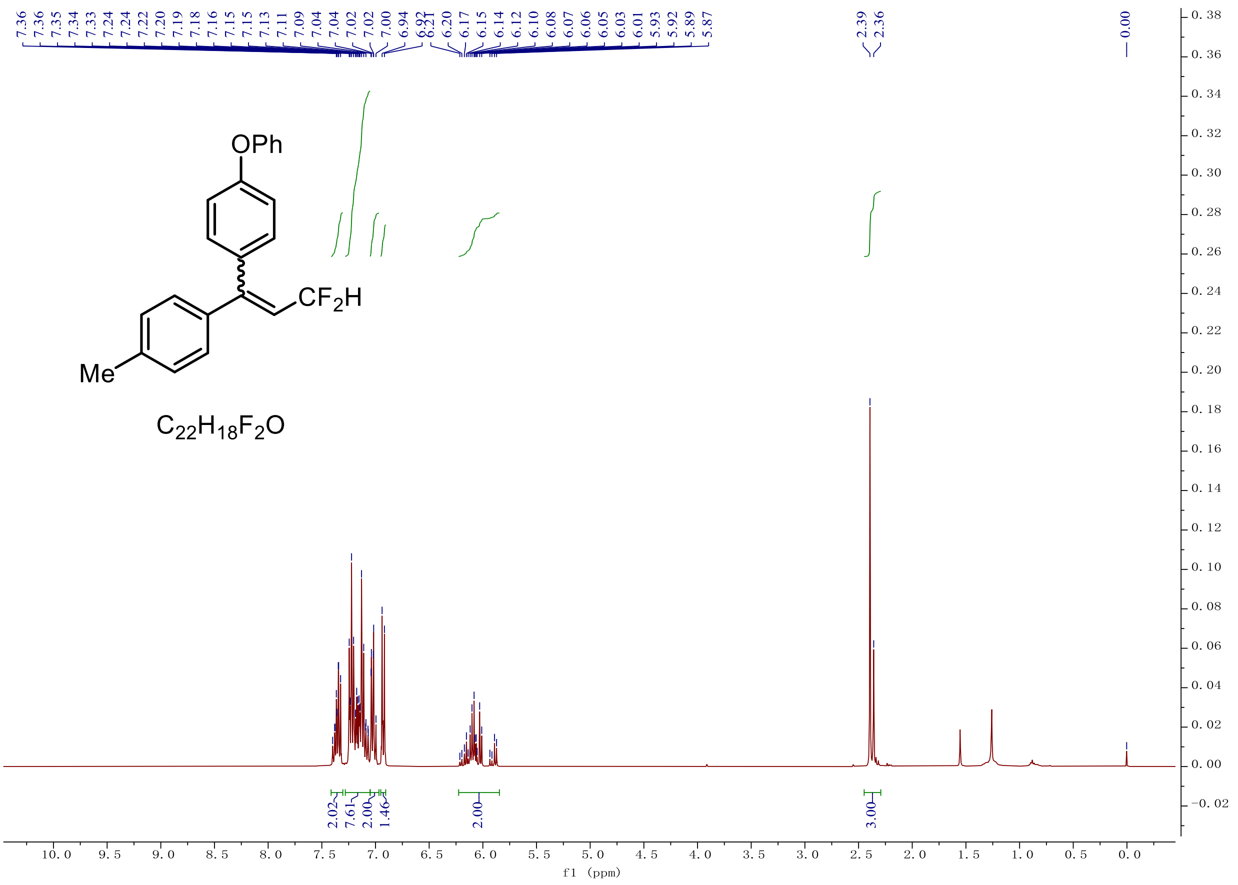

^{13}C NMR (101 MHz, CDCl3) spectrum of 5d

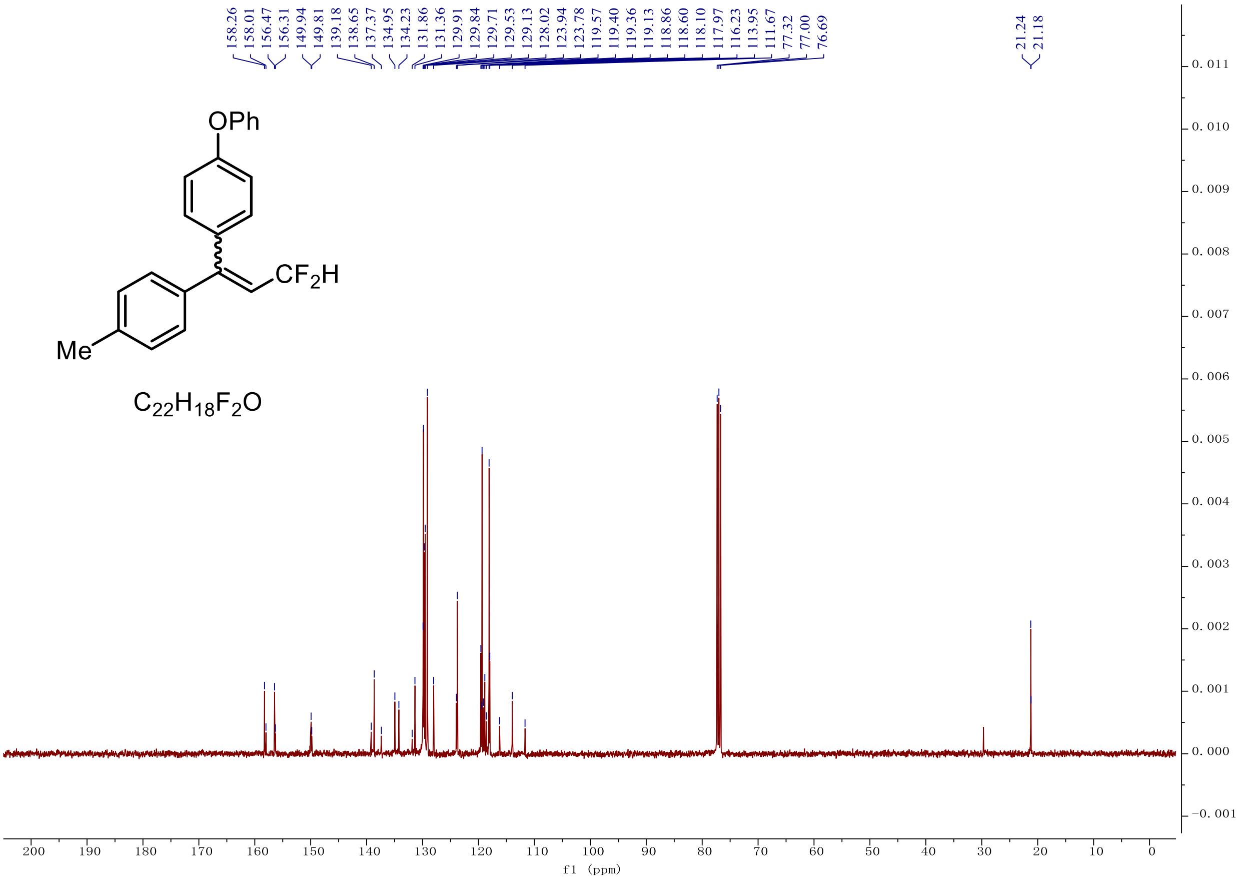

^{19}F NMR (376 MHz, $CDCl_3$) spectrum of 5d

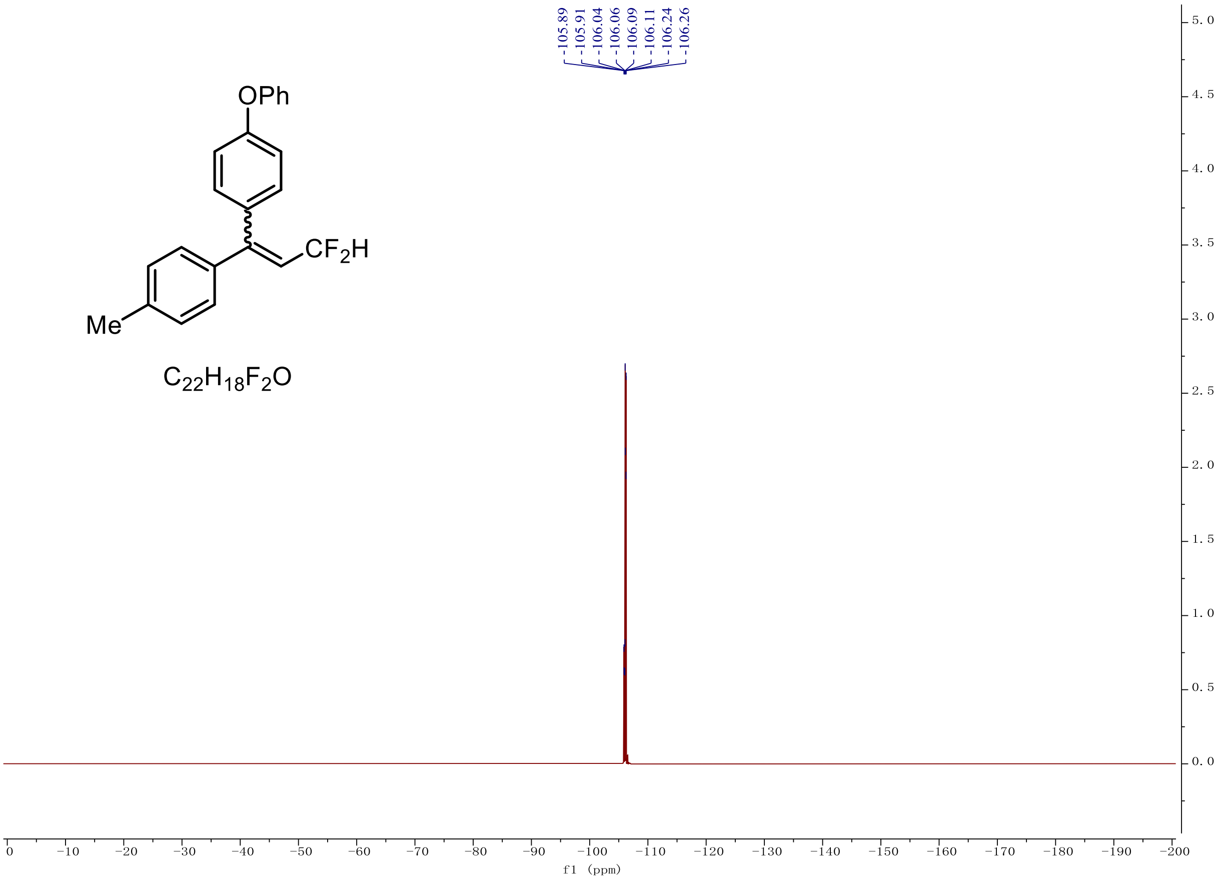

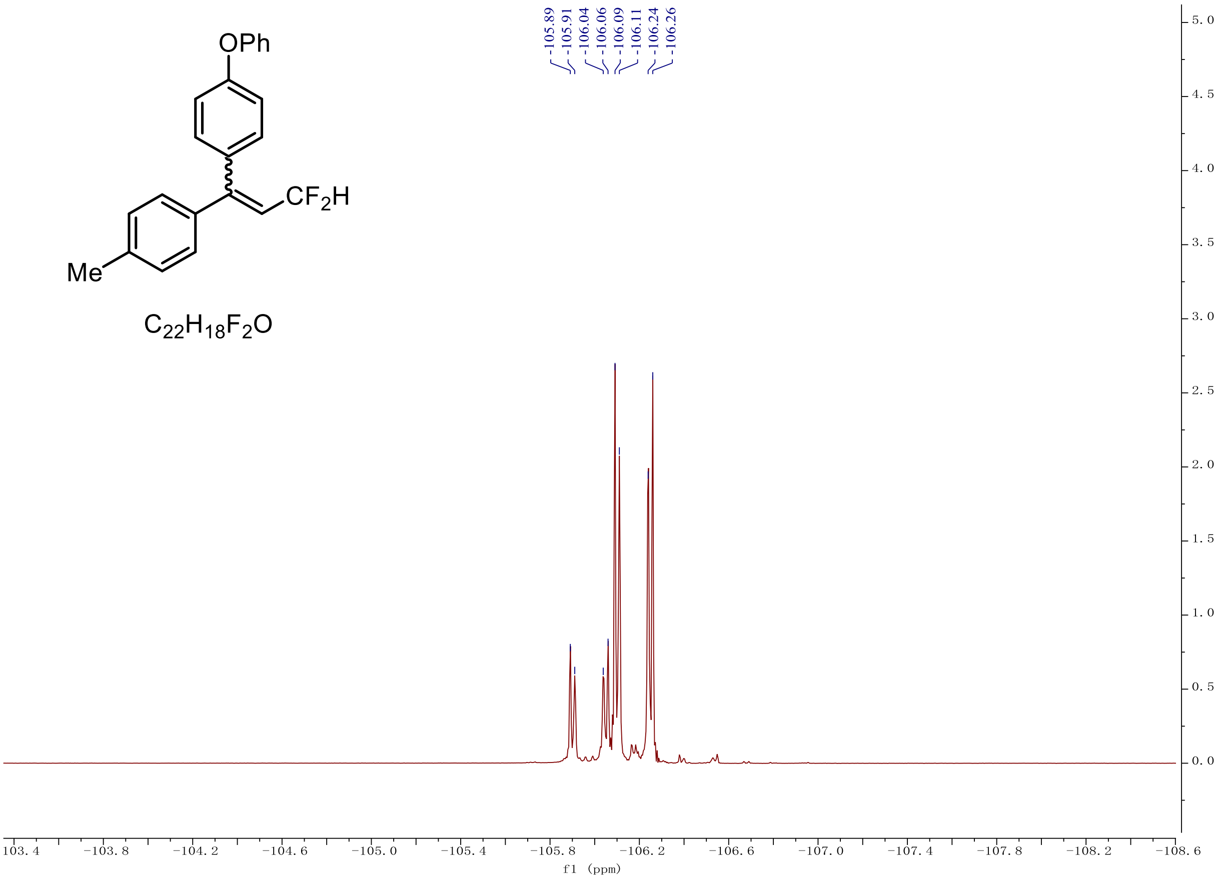

^{1}H NMR (400 MHz, CDCl3) spectrum of 6a

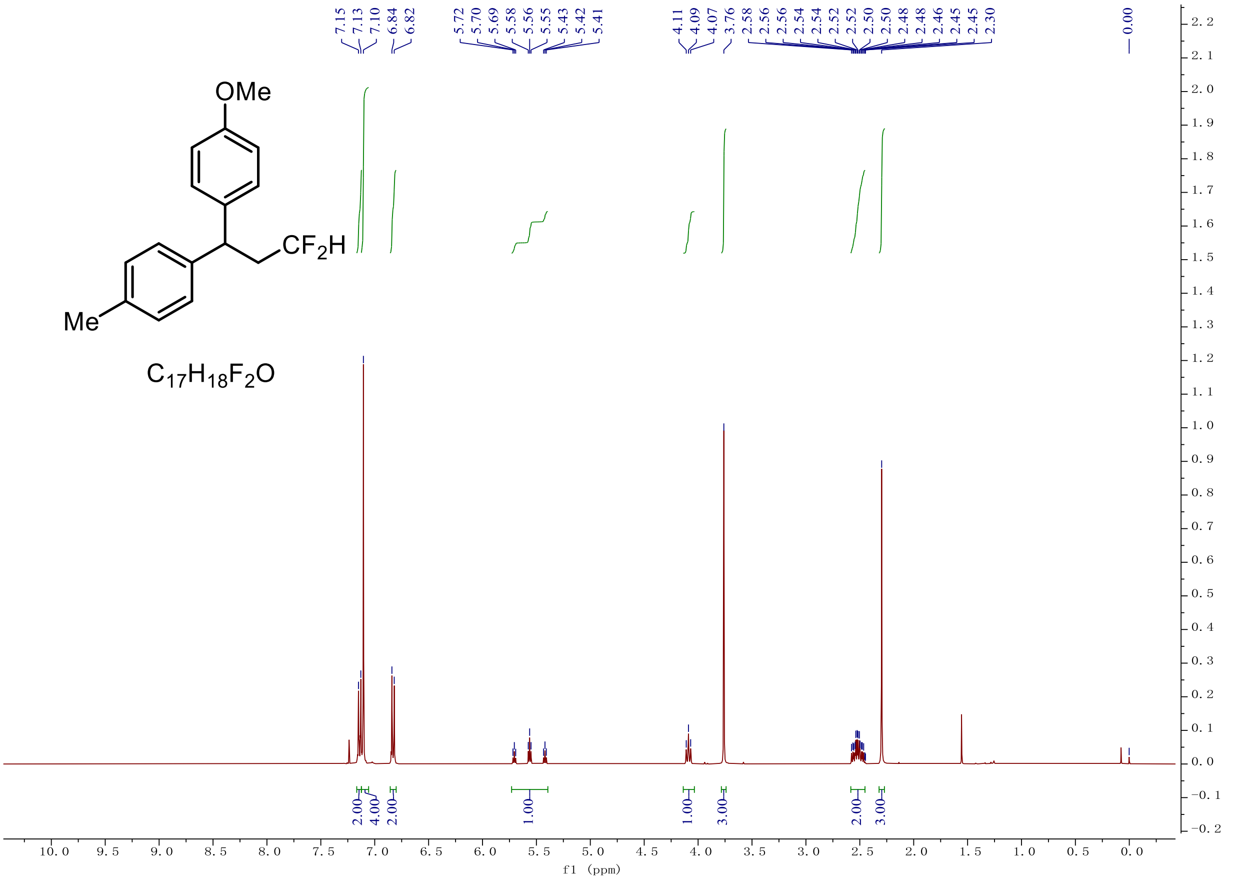

^{13}C NMR (101 MHz, CDCl3) spectrum of 6a

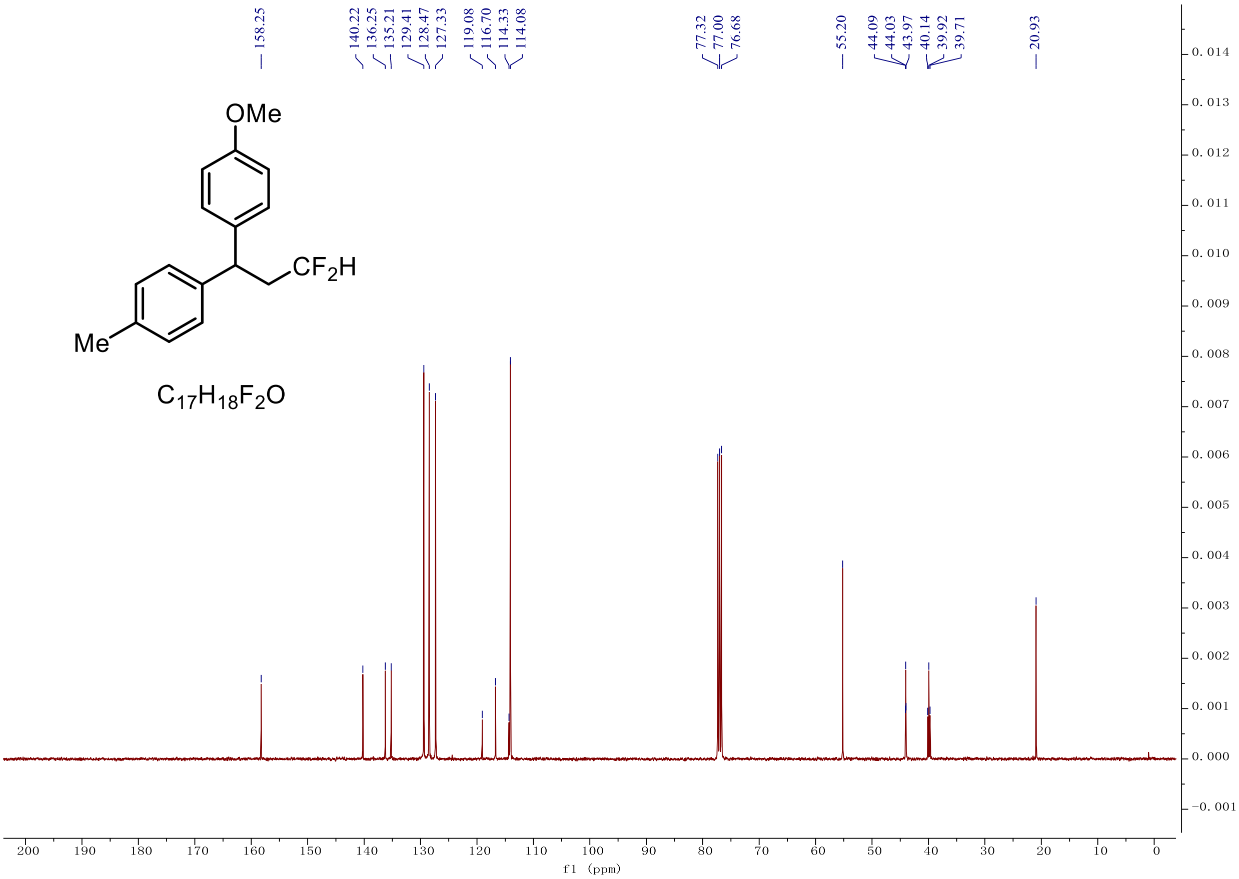

^{19}F NMR (376 MHz, $CDCl_3$) spectrum of 6a

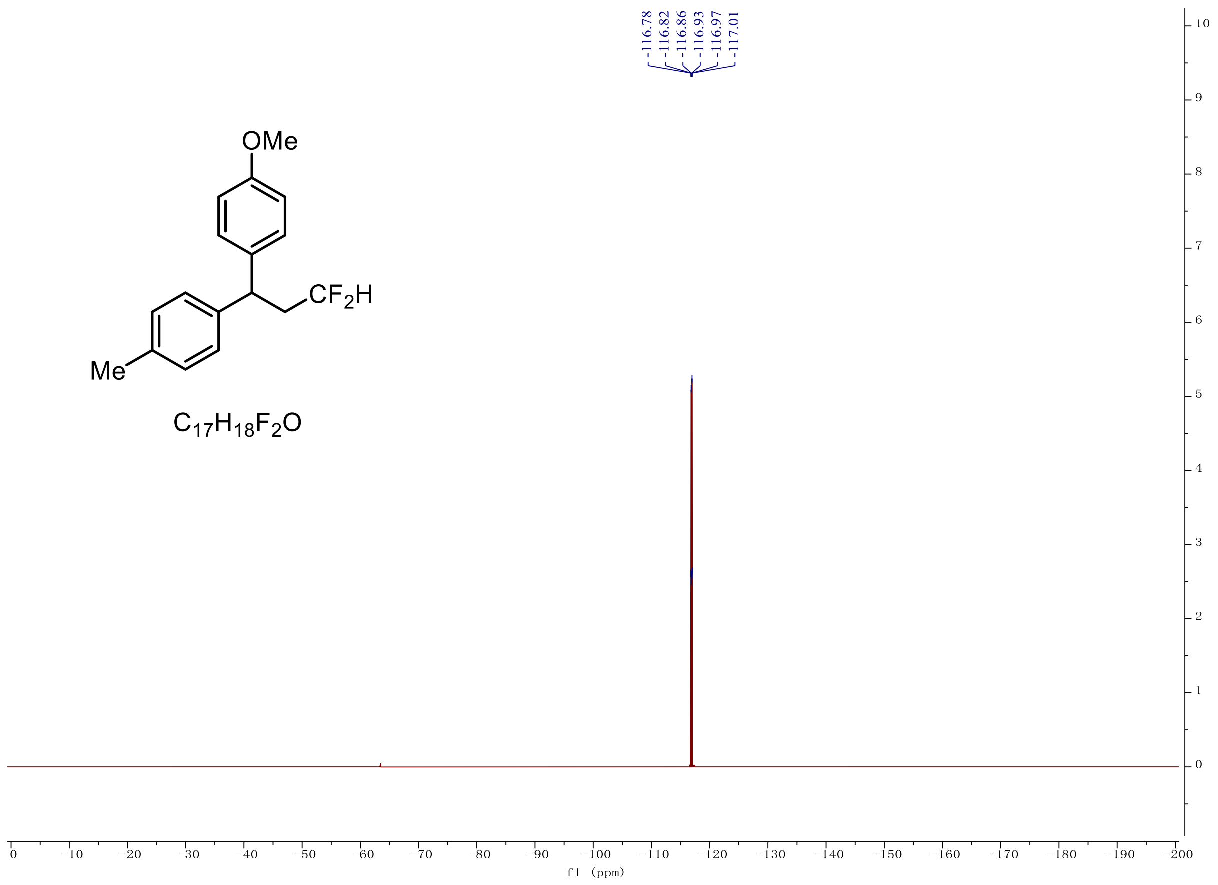

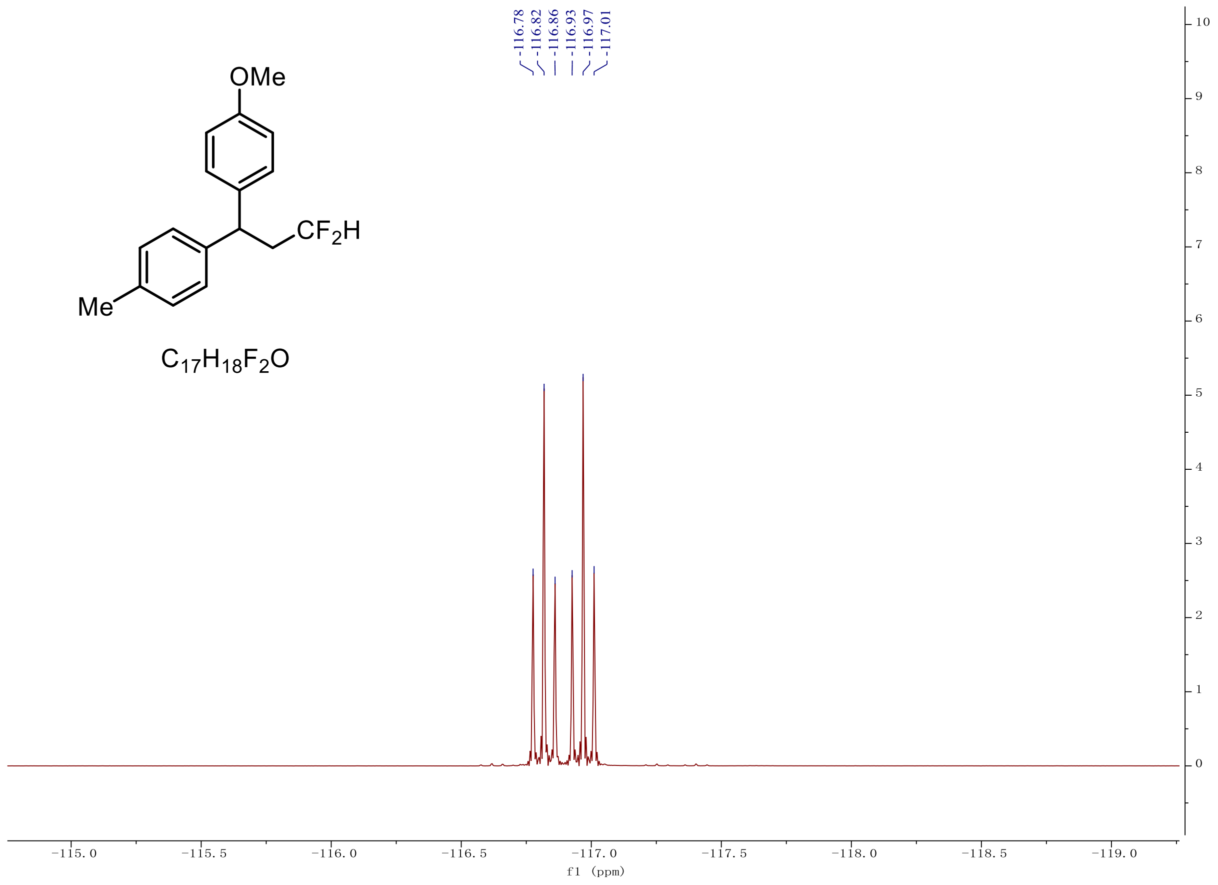

^{1}H NMR (400 MHz, $CDCl_3$) spectrum of 6b

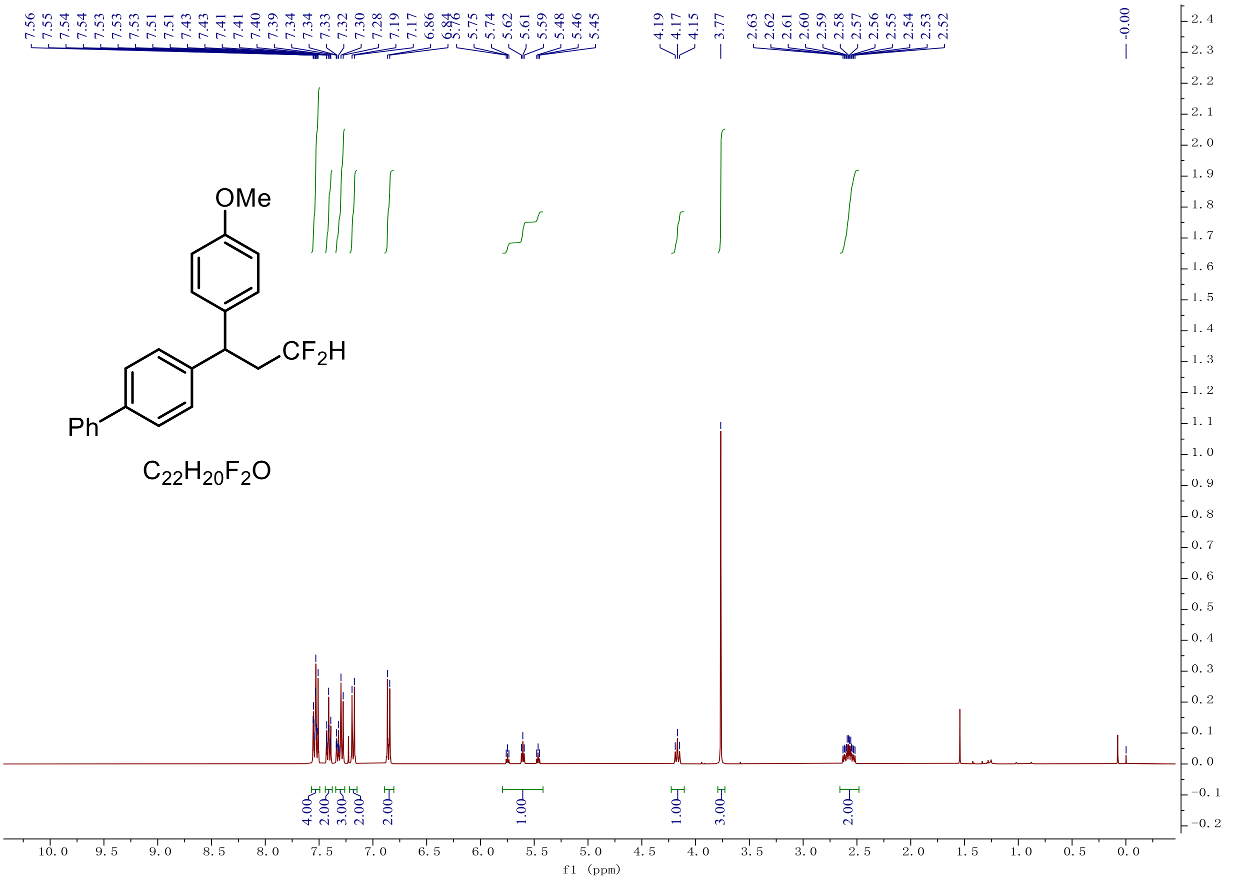

^{13}C NMR (101 MHz, $CDCl_3$) spectrum of 6b

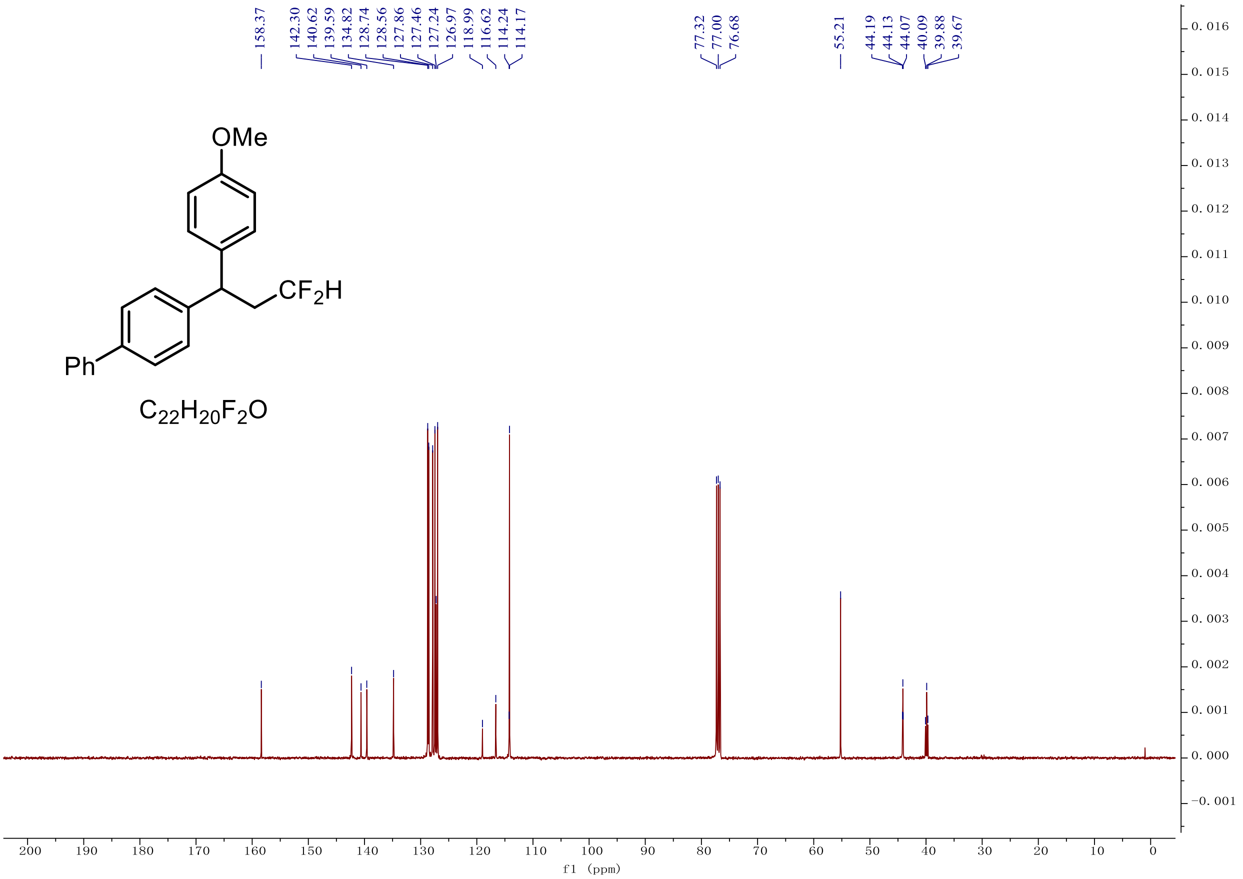

^{19}F NMR (376 MHz, CDCl3) spectrum of 6b

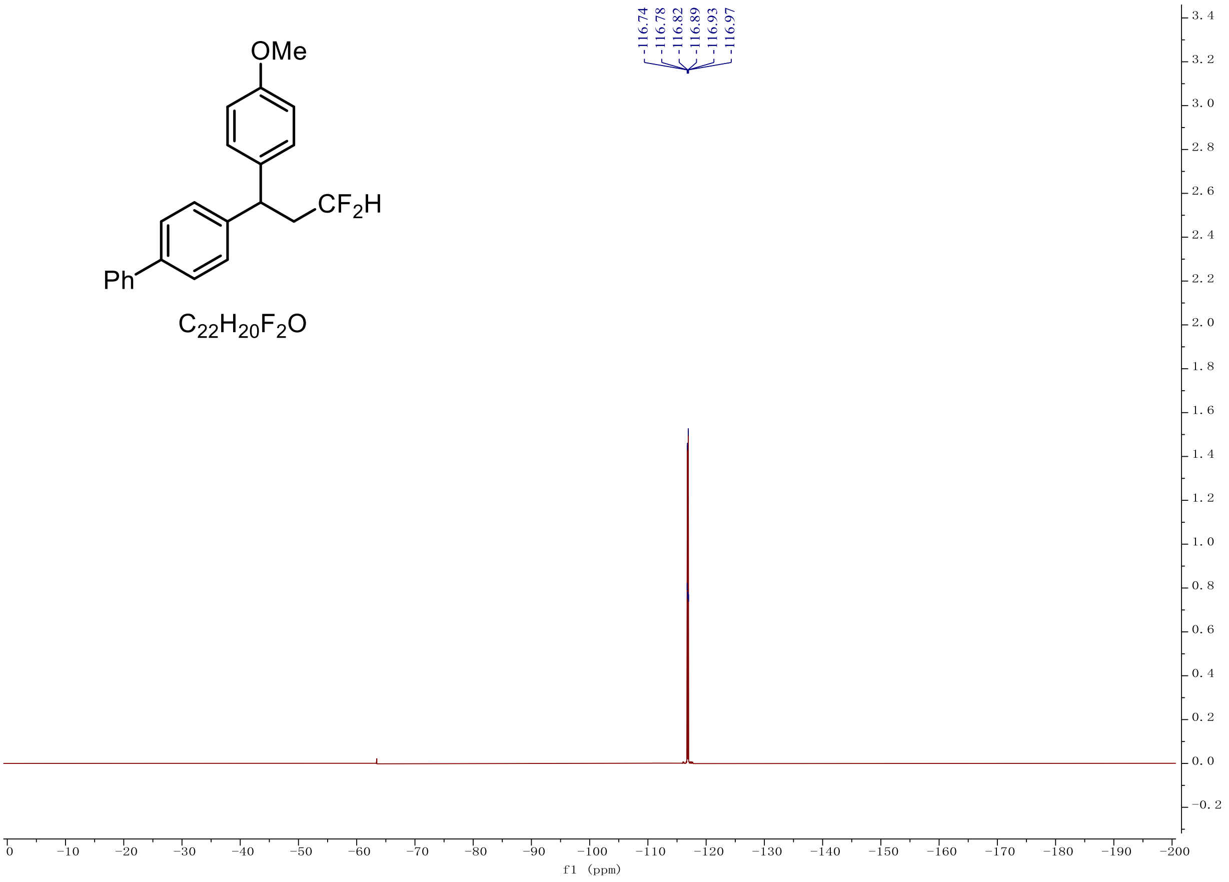

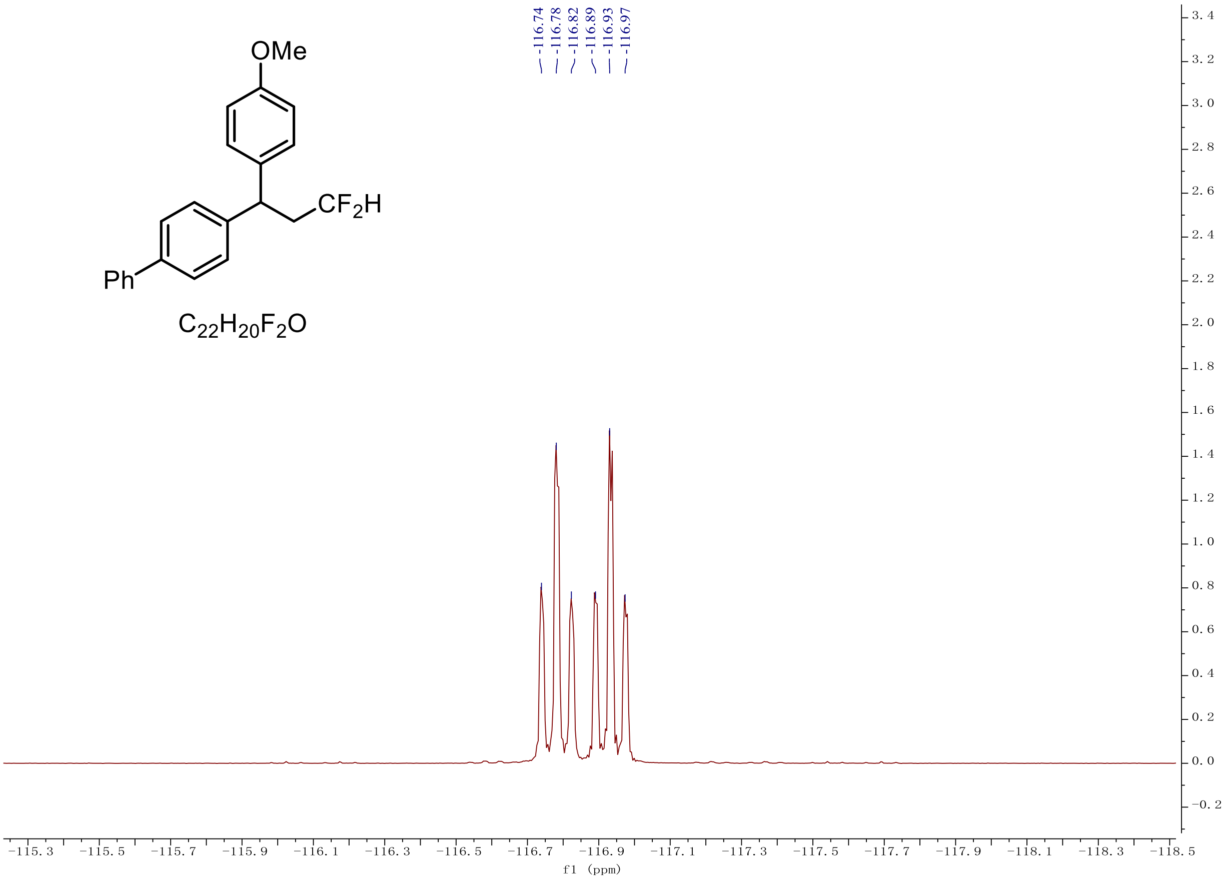

^{1}H NMR (400 MHz, $CDCl_3$) spectrum of 6c

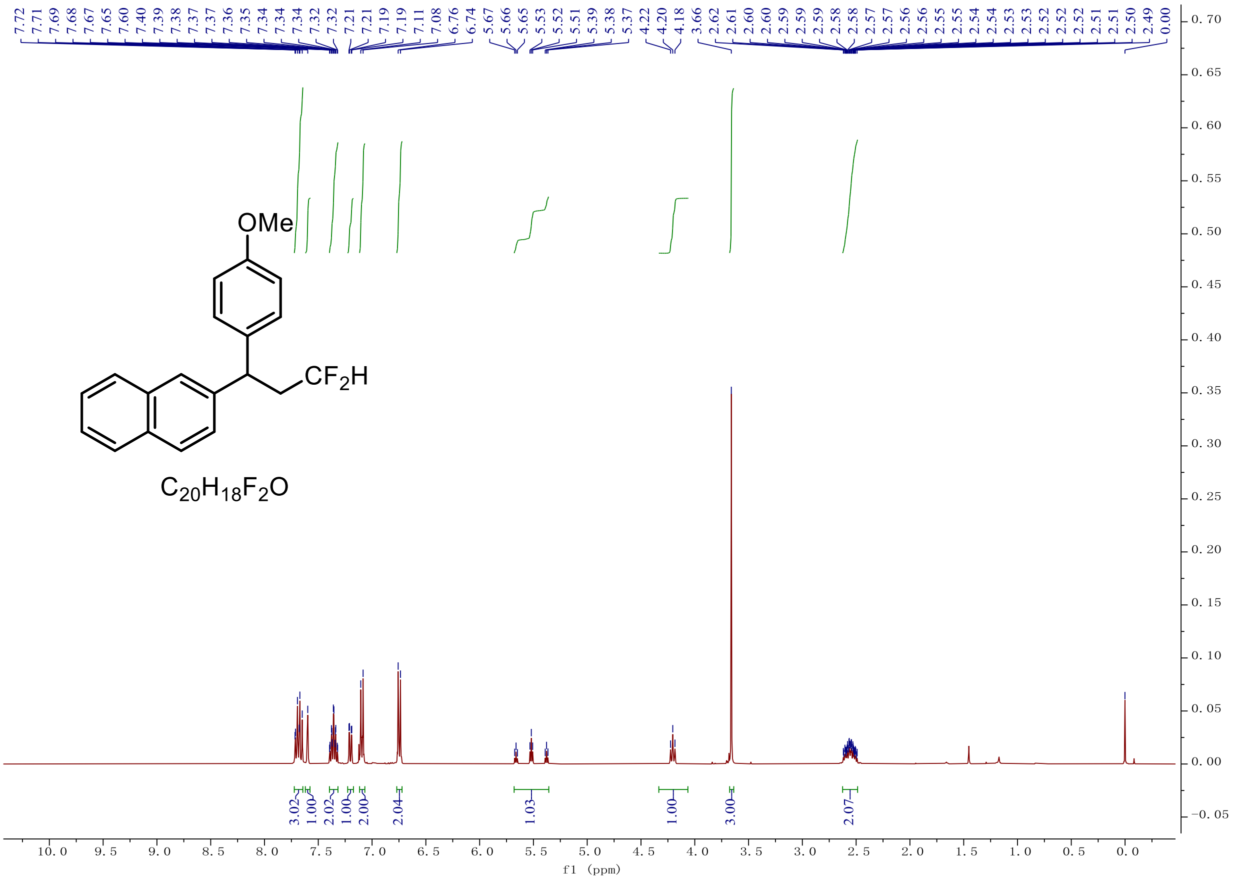

^{13}C NMR (101 MHz, $CDCl_3$) spectrum of 6c

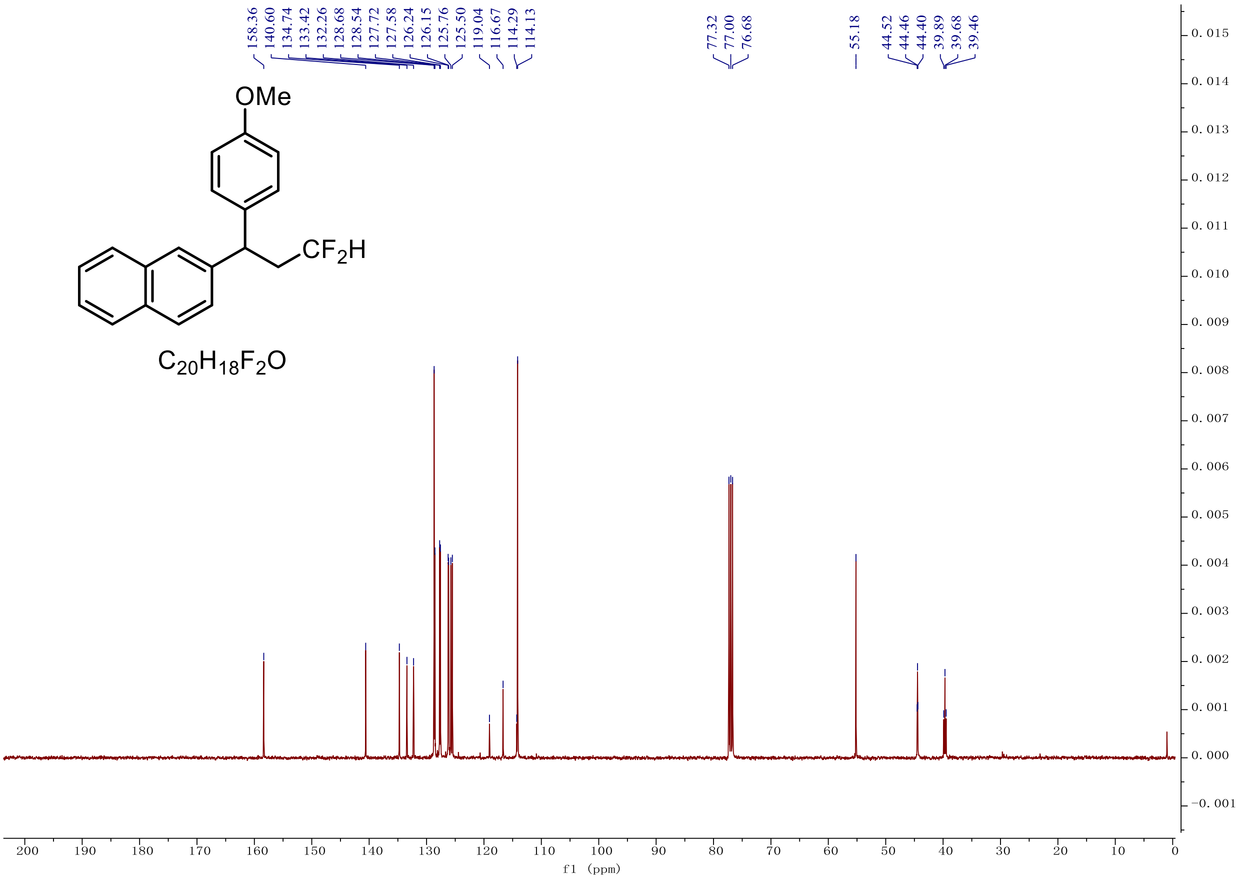

^{19}F NMR (376 MHz, $CDCl_3$) spectrum of 6c

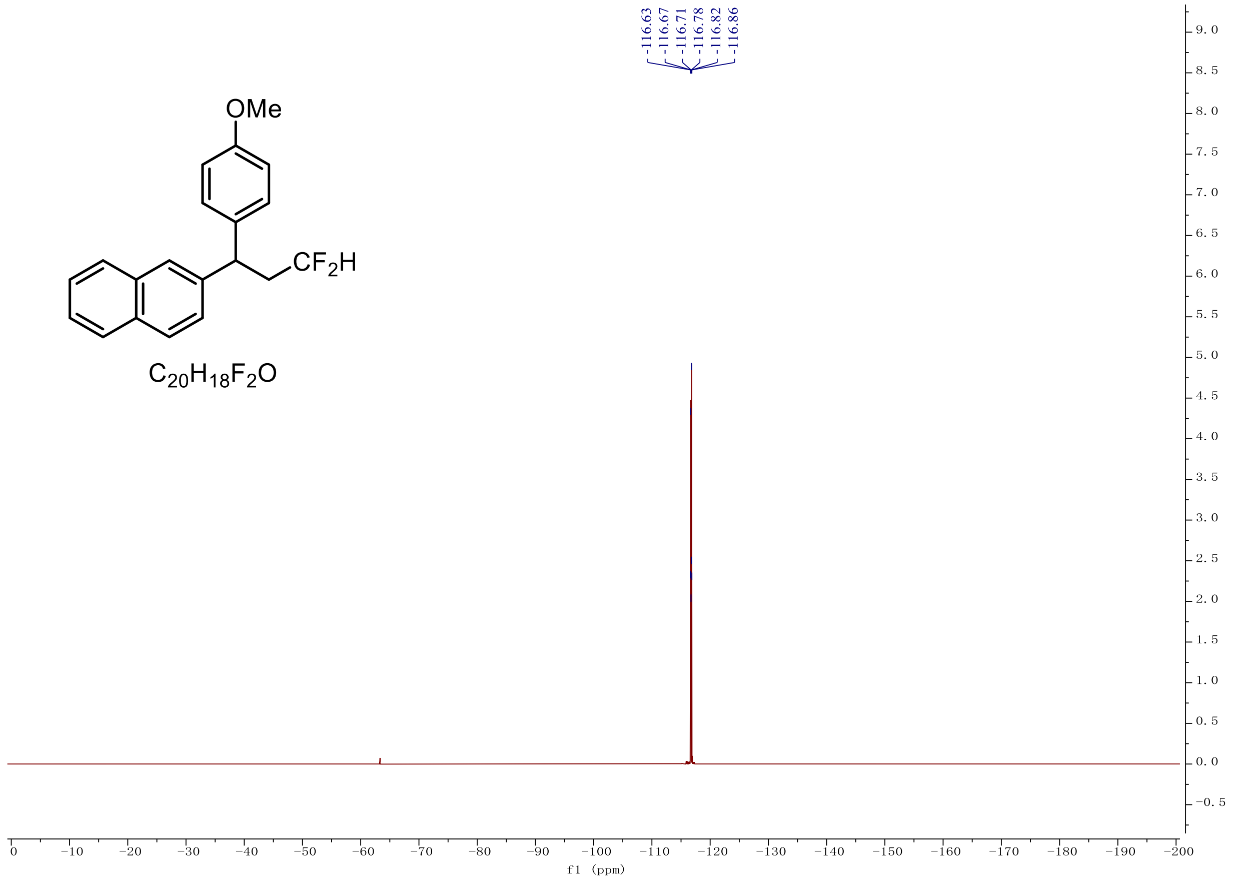

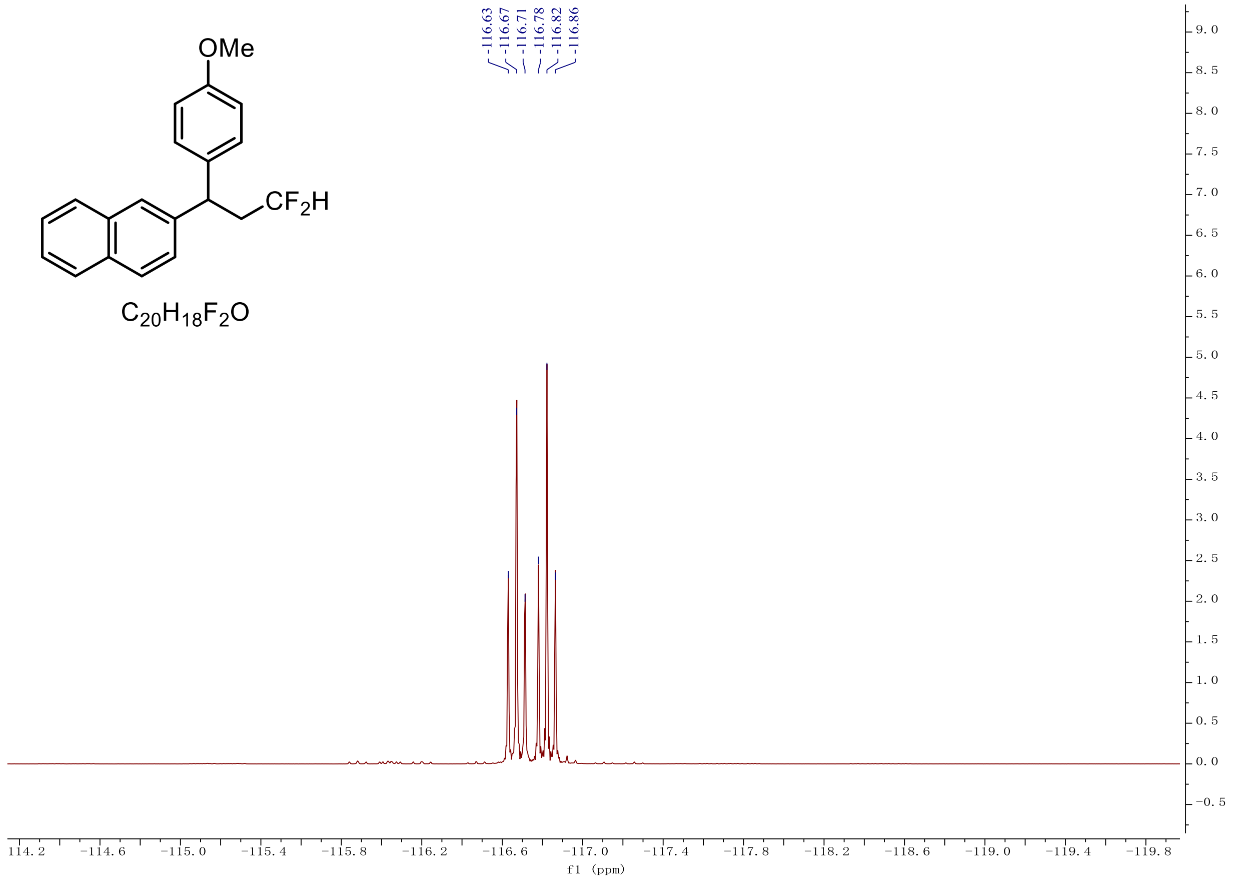

^{1}H NMR (400 MHz, CDCl3) spectrum of 6d

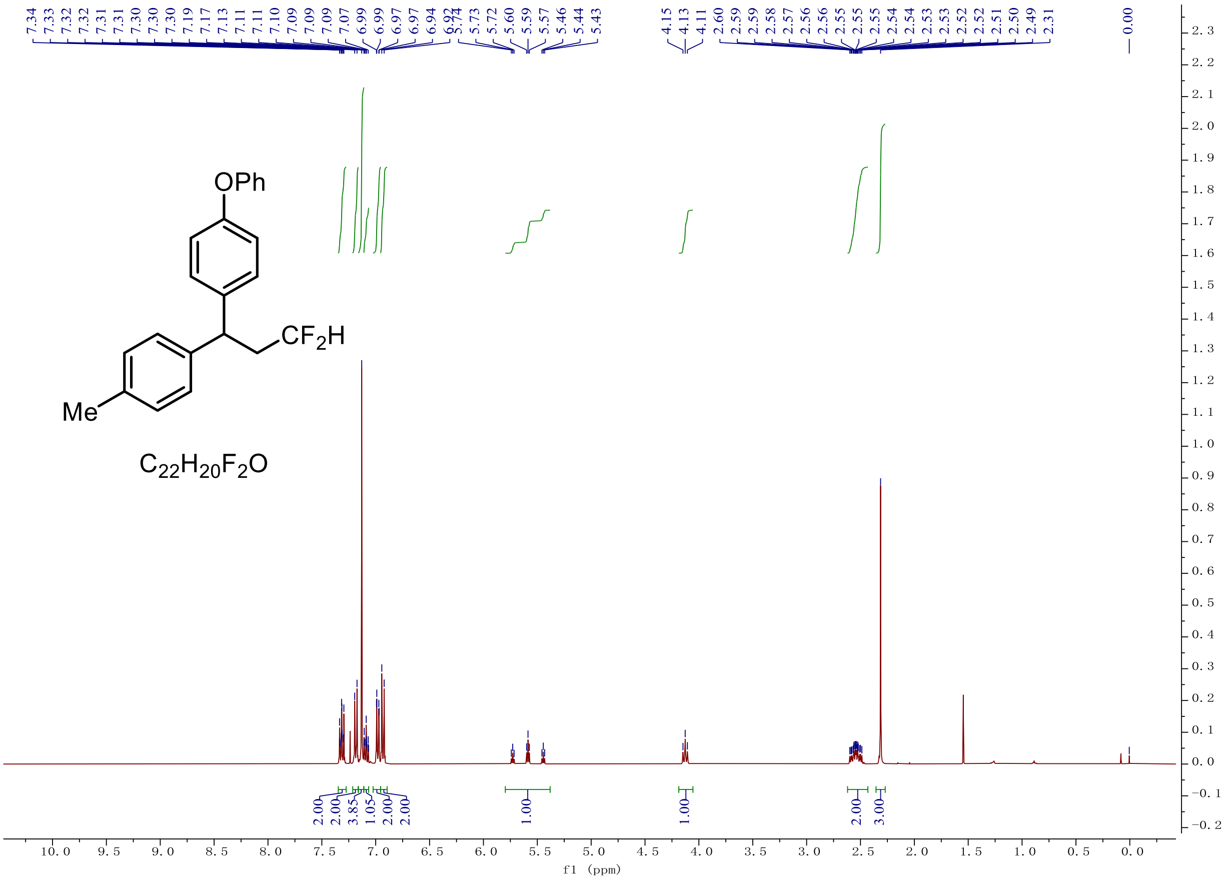

^{13}C NMR (101 MHz, CDCl3) spectrum of 6d

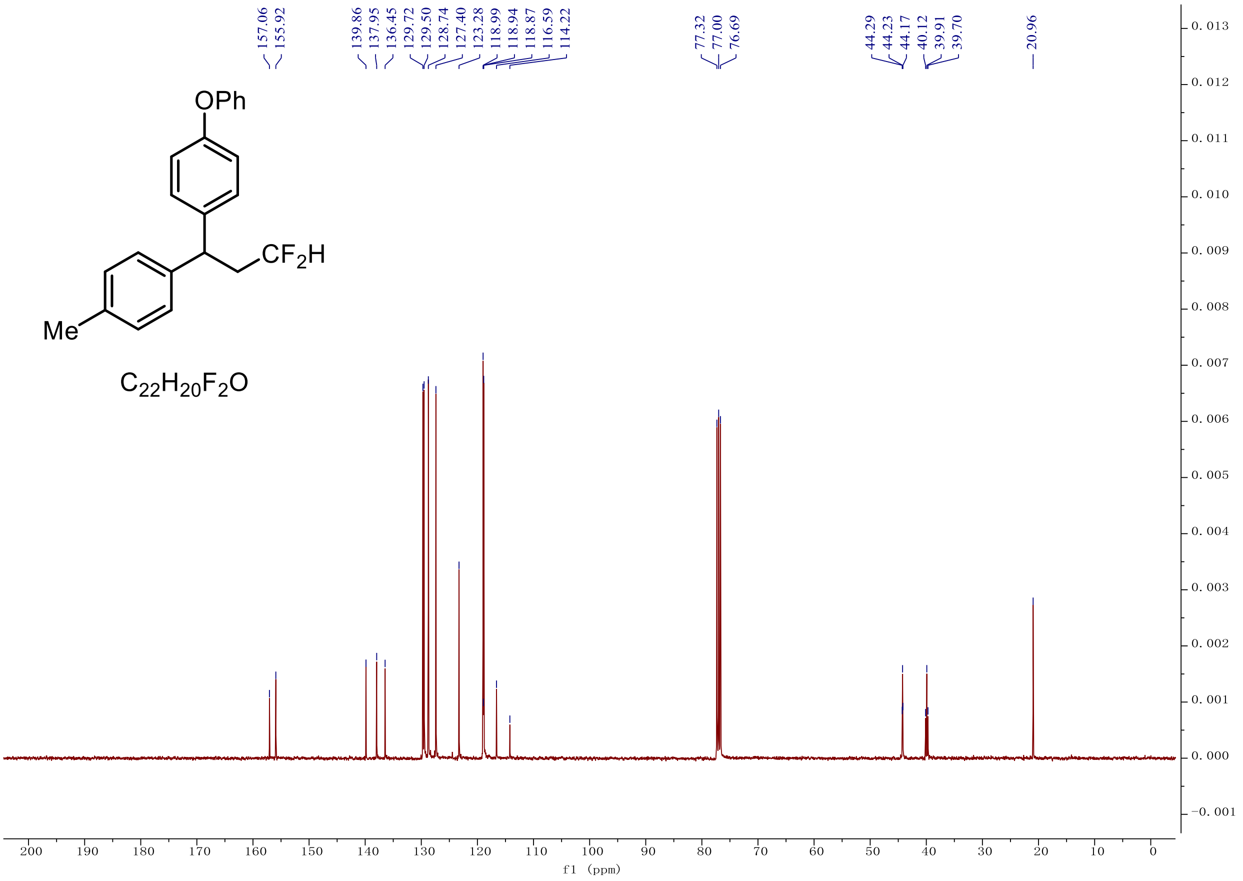

^{19}F NMR (376 MHz, $CDCl_3$) spectrum of 6d

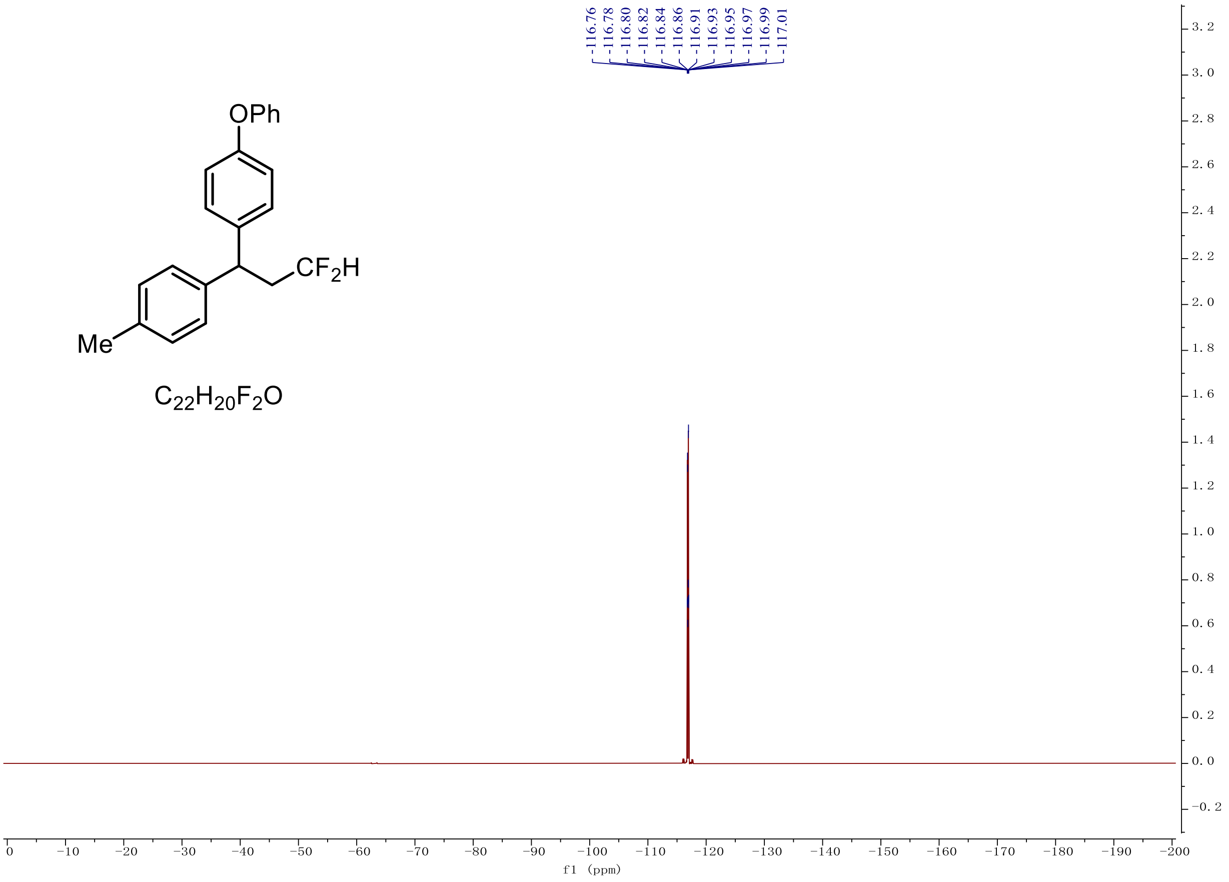

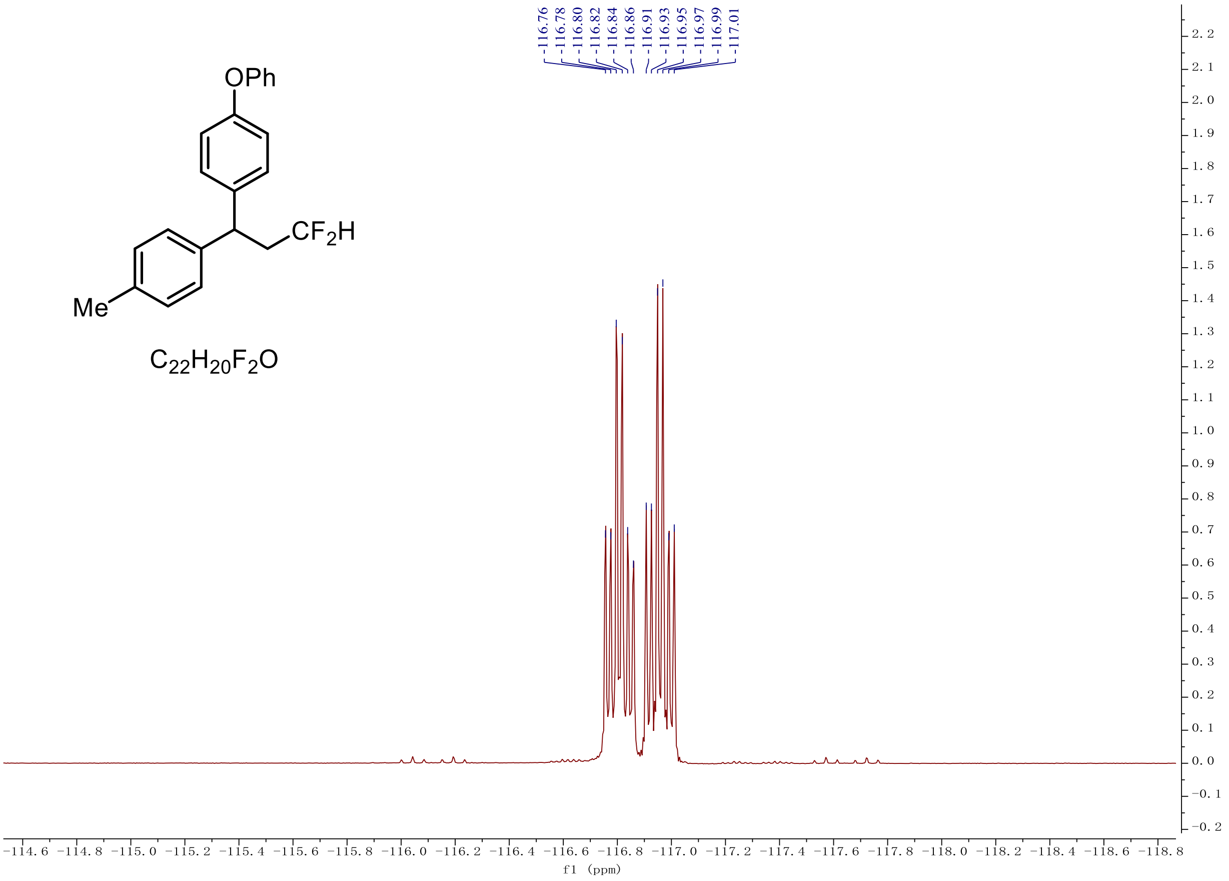

^{1}H NMR (400 MHz, $CDCl_3$) spectrum of 7a

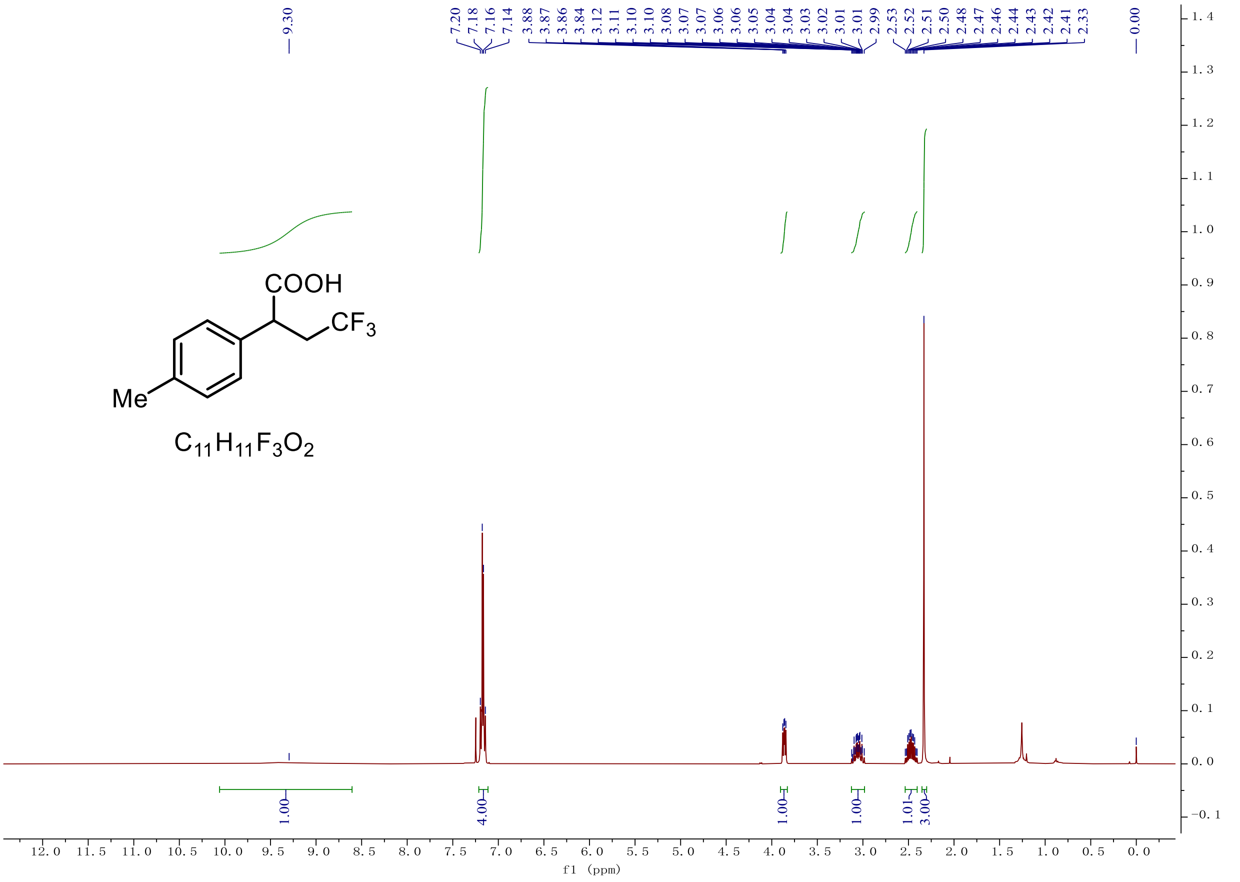

^{13}C NMR (101 MHz, $CDCl_3$) spectrum of 7a

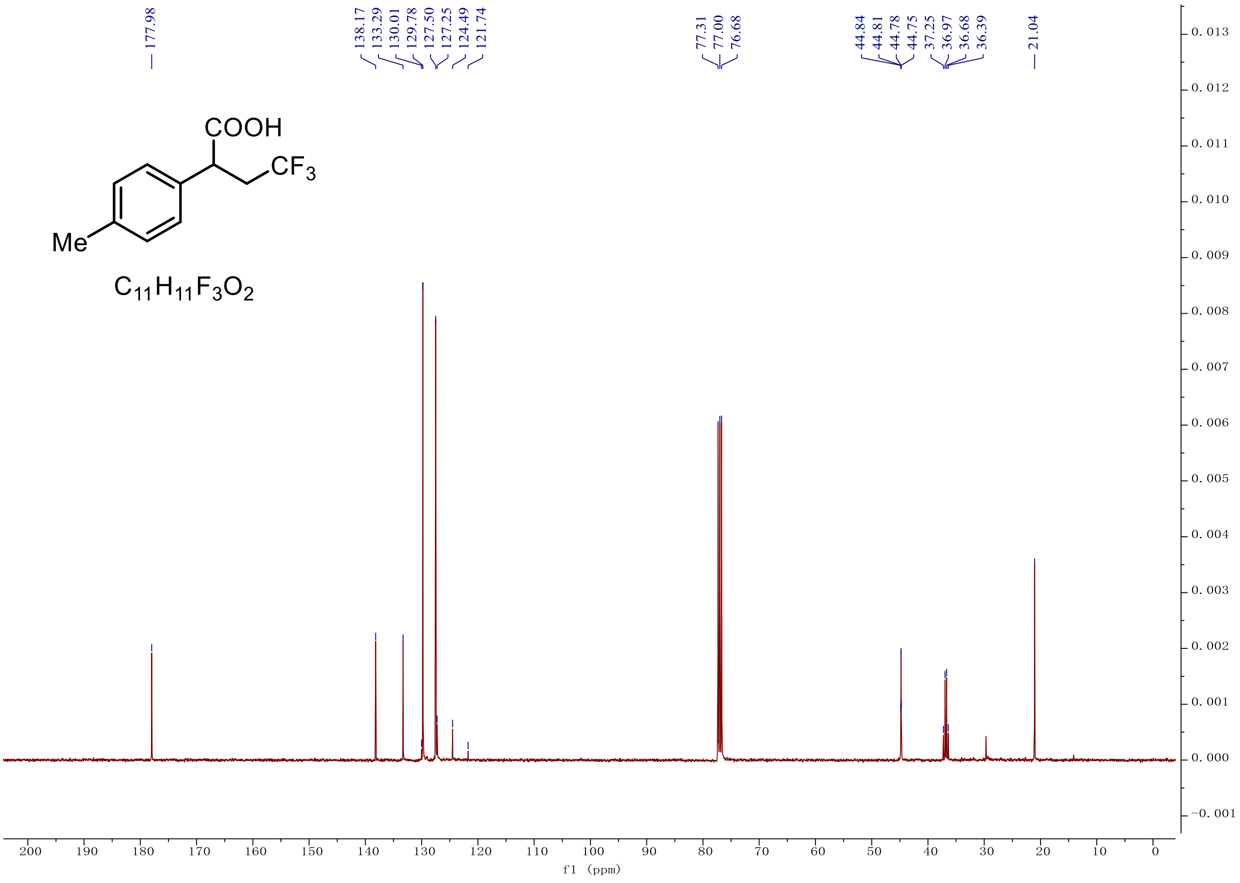

^{19}F NMR (376 MHz, CDCl3) spectrum of 7a

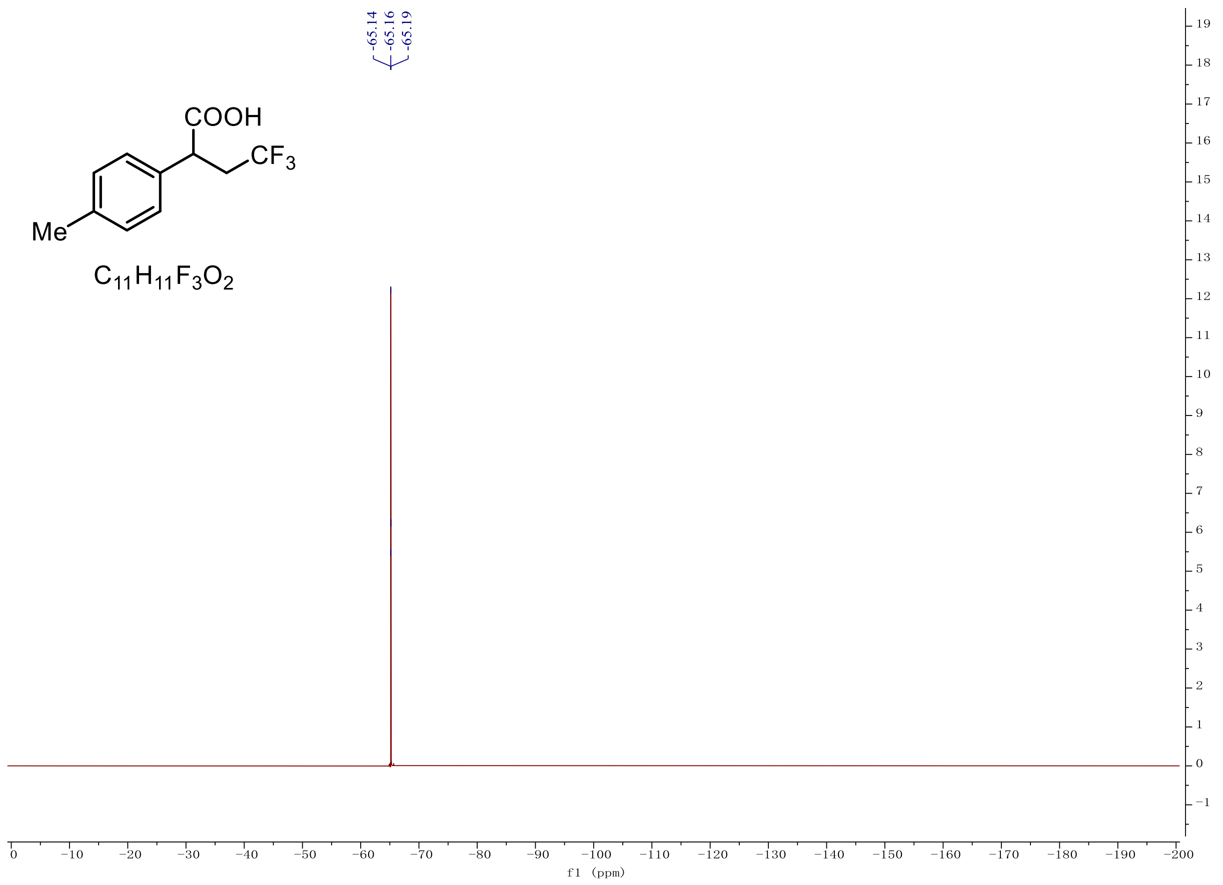

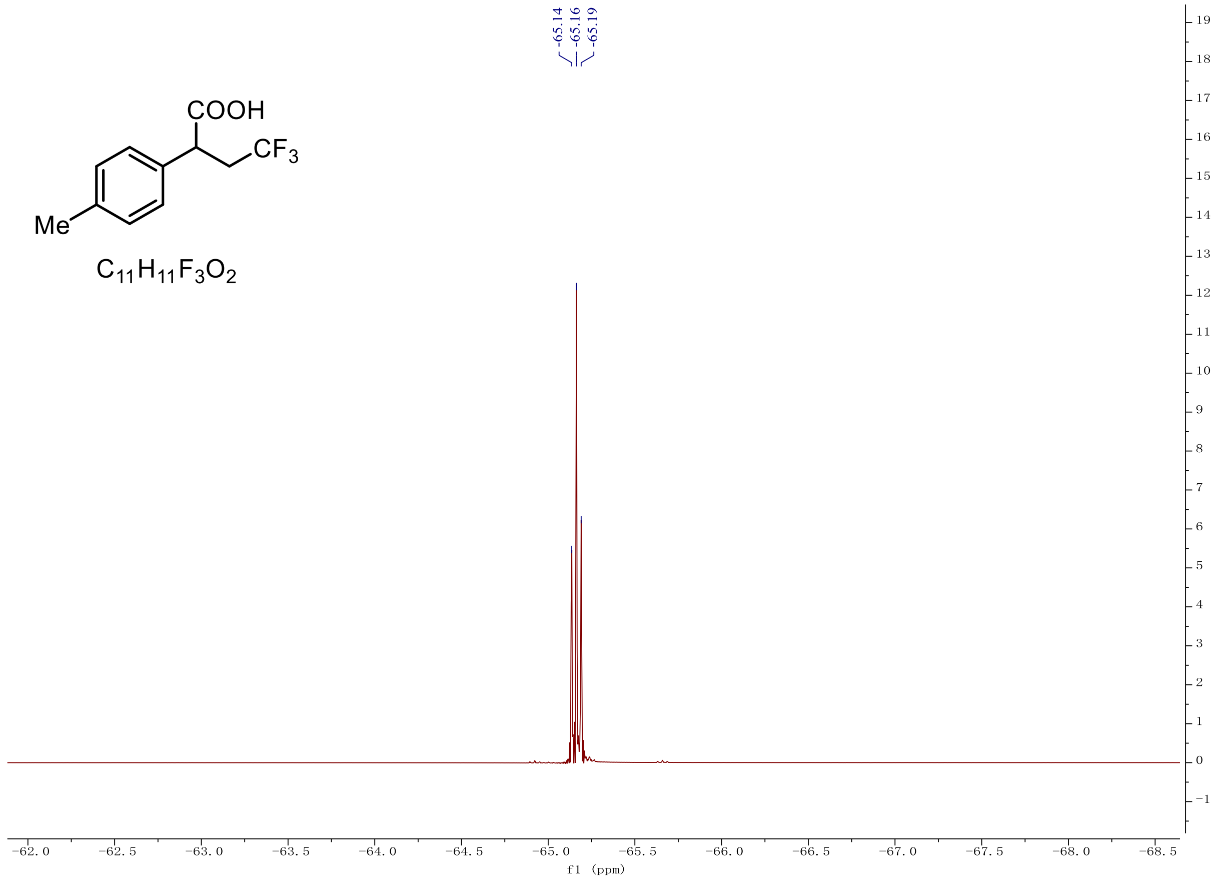

^{1}H NMR (400 MHz, $CDCl_3$) spectrum of 7w

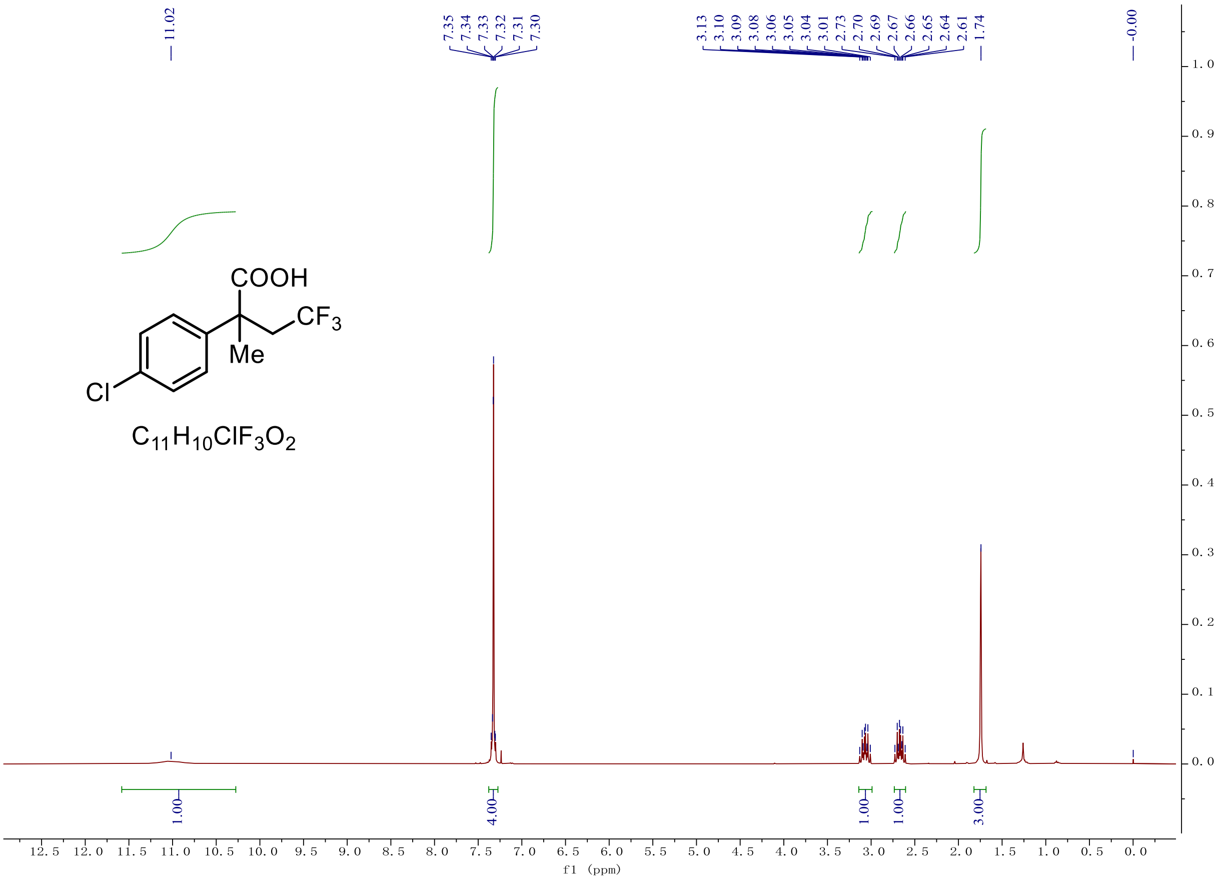

^{13}C NMR (101 MHz, $CDCl_3$) spectrum of 7w

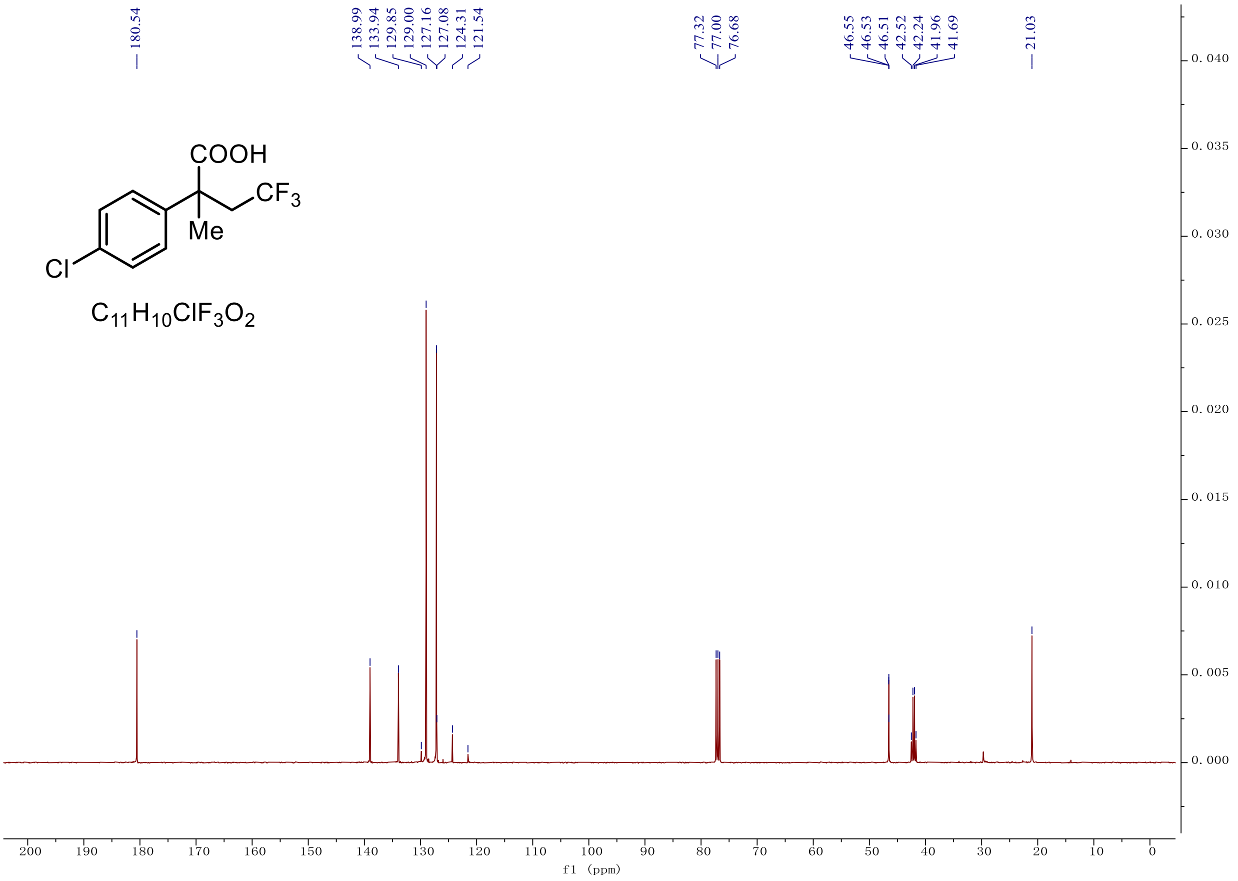

^{19}F NMR (376 MHz, $CDCl_3$) spectrum of 7w

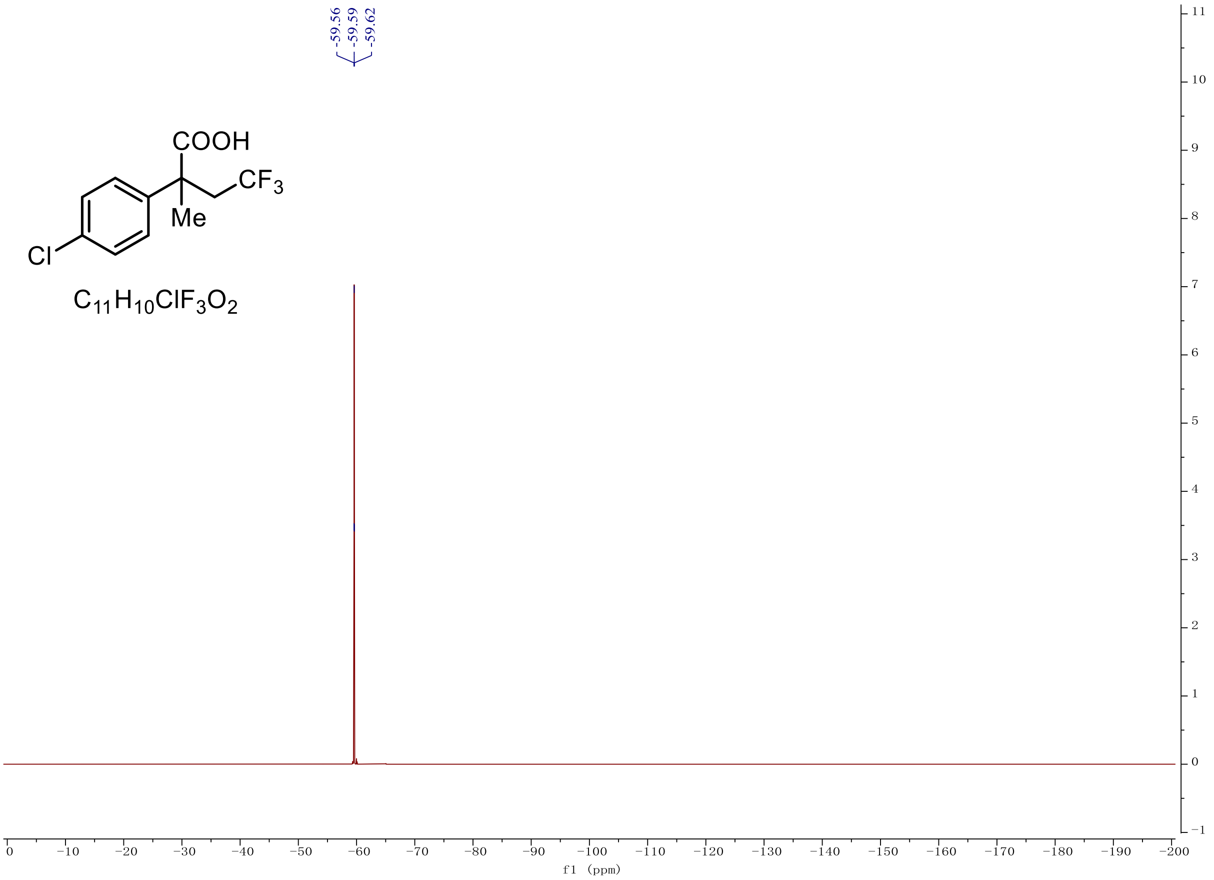

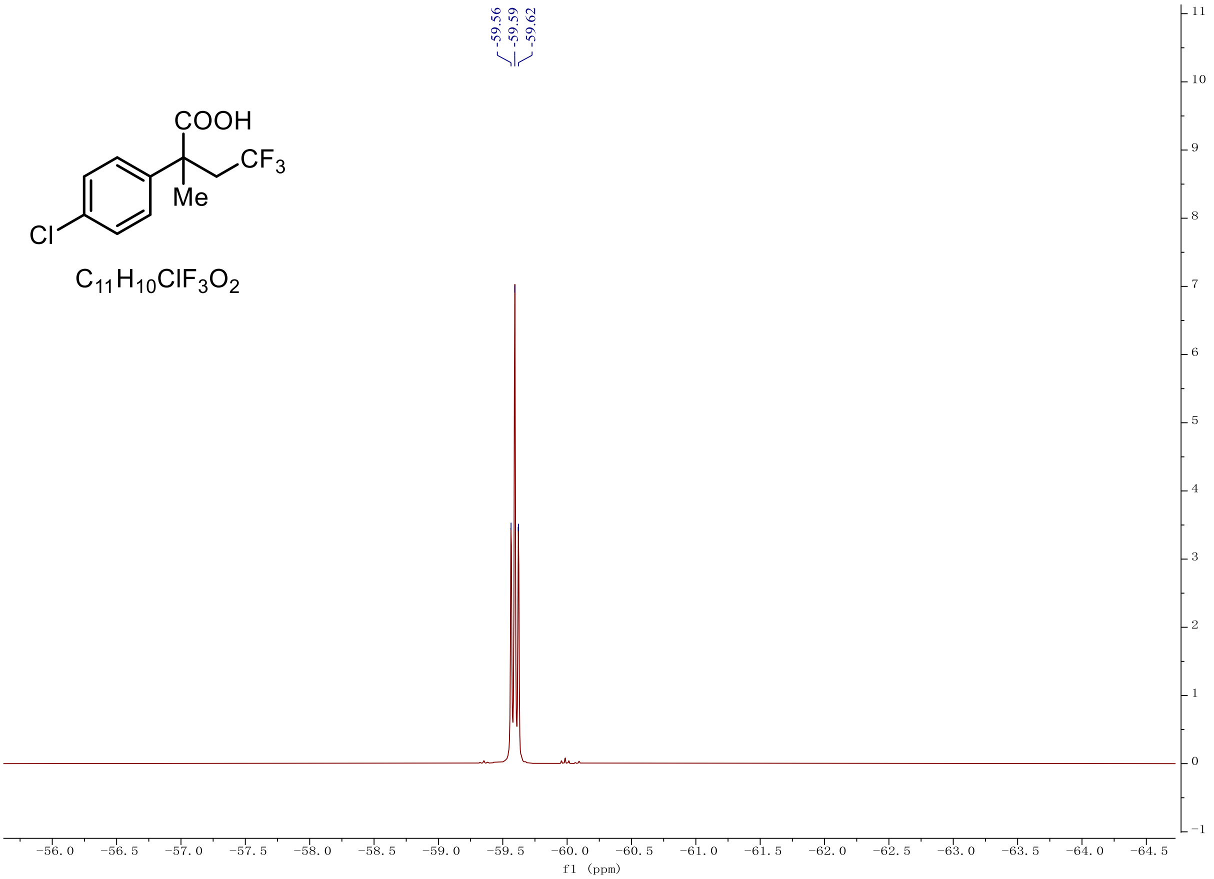

^{1}H NMR (400 MHz, $CDCl_3$) spectrum of 11b

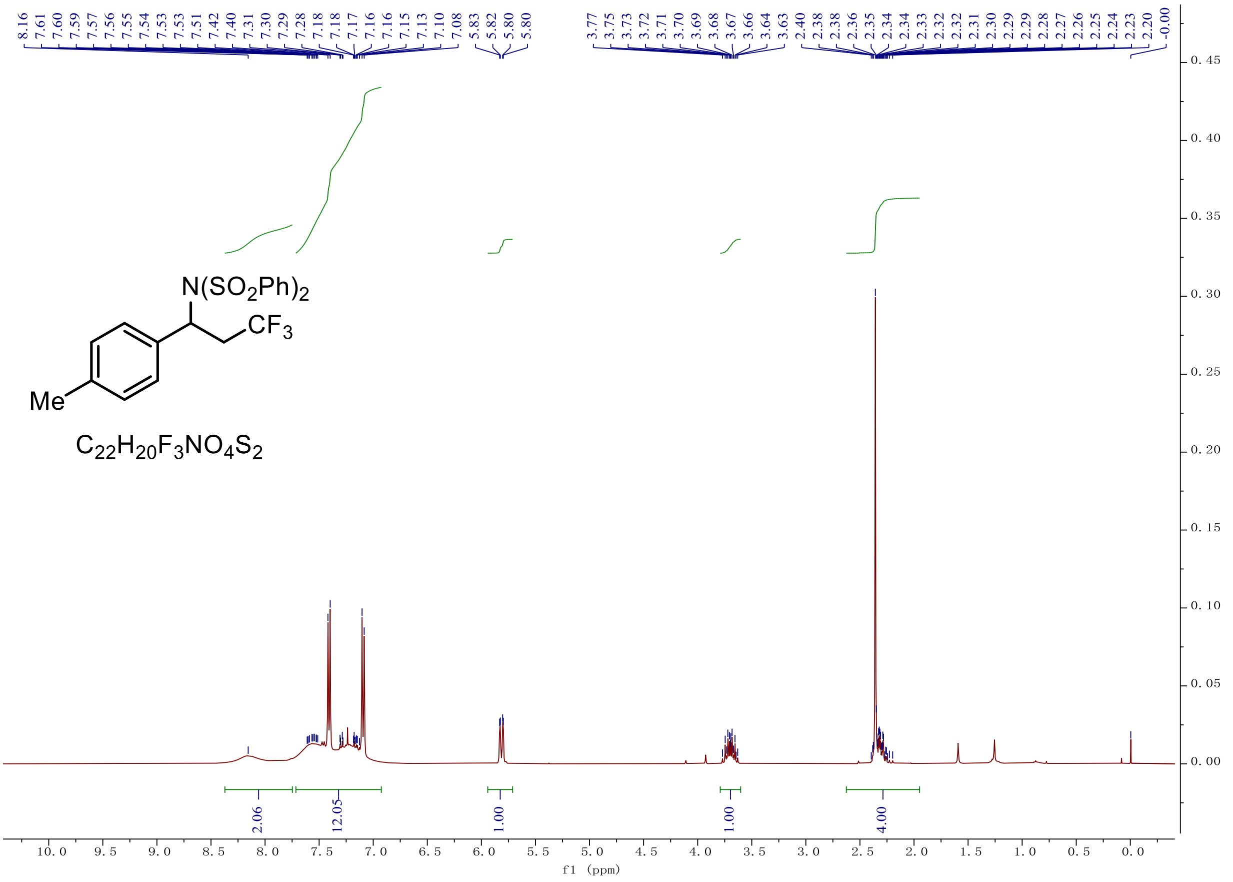

^{13}C NMR (101 MHz, $CDCl_3$) spectrum of 11b

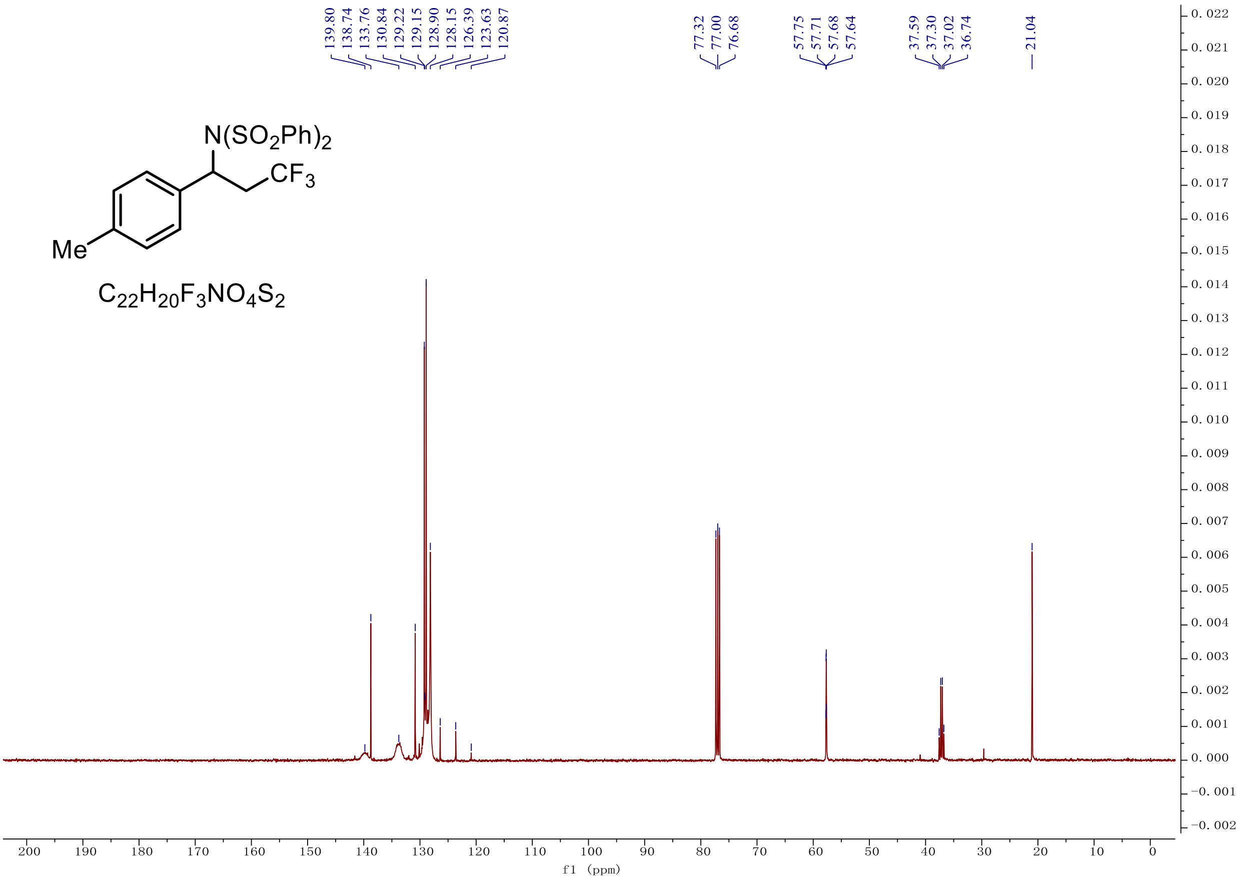

^{19}F NMR (376 MHz, $CDCl_3$) spectrum of 11b

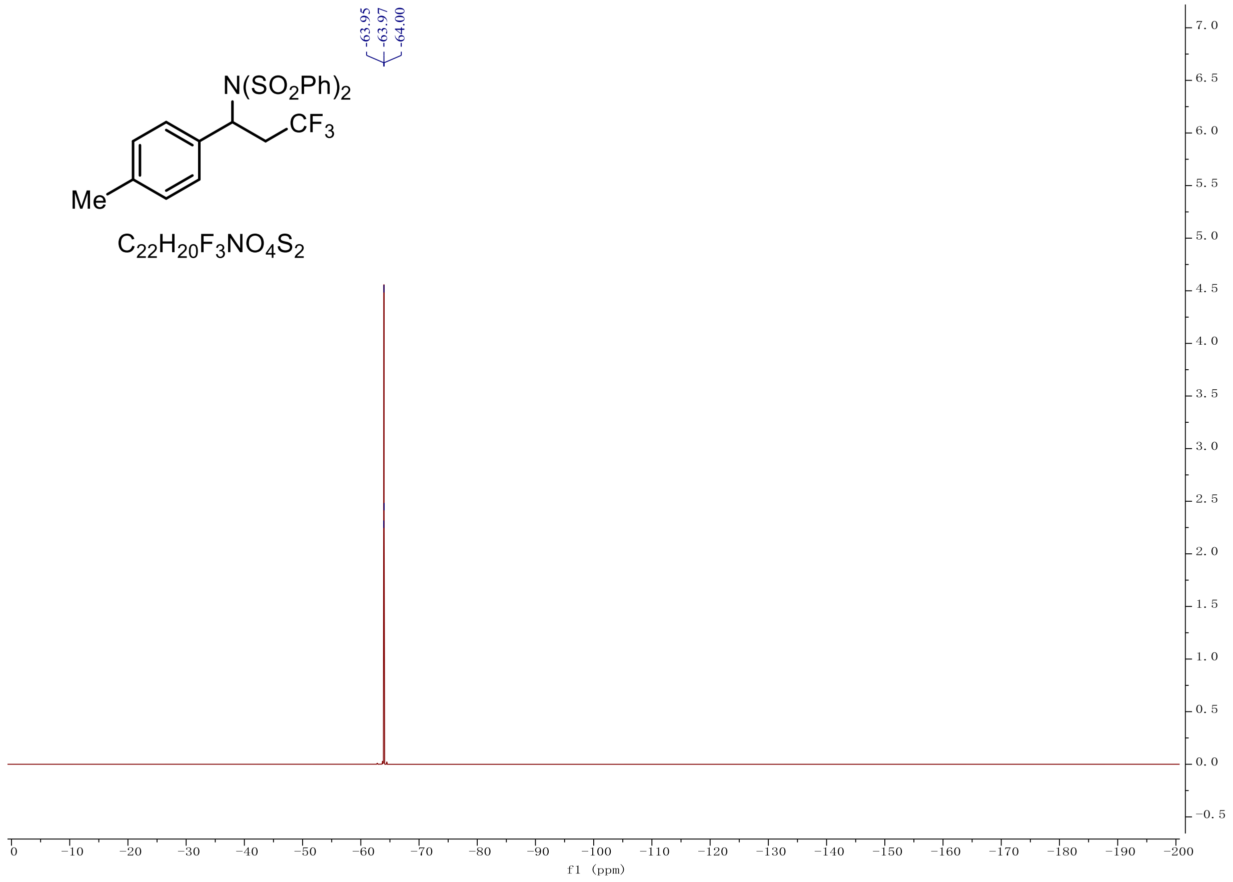

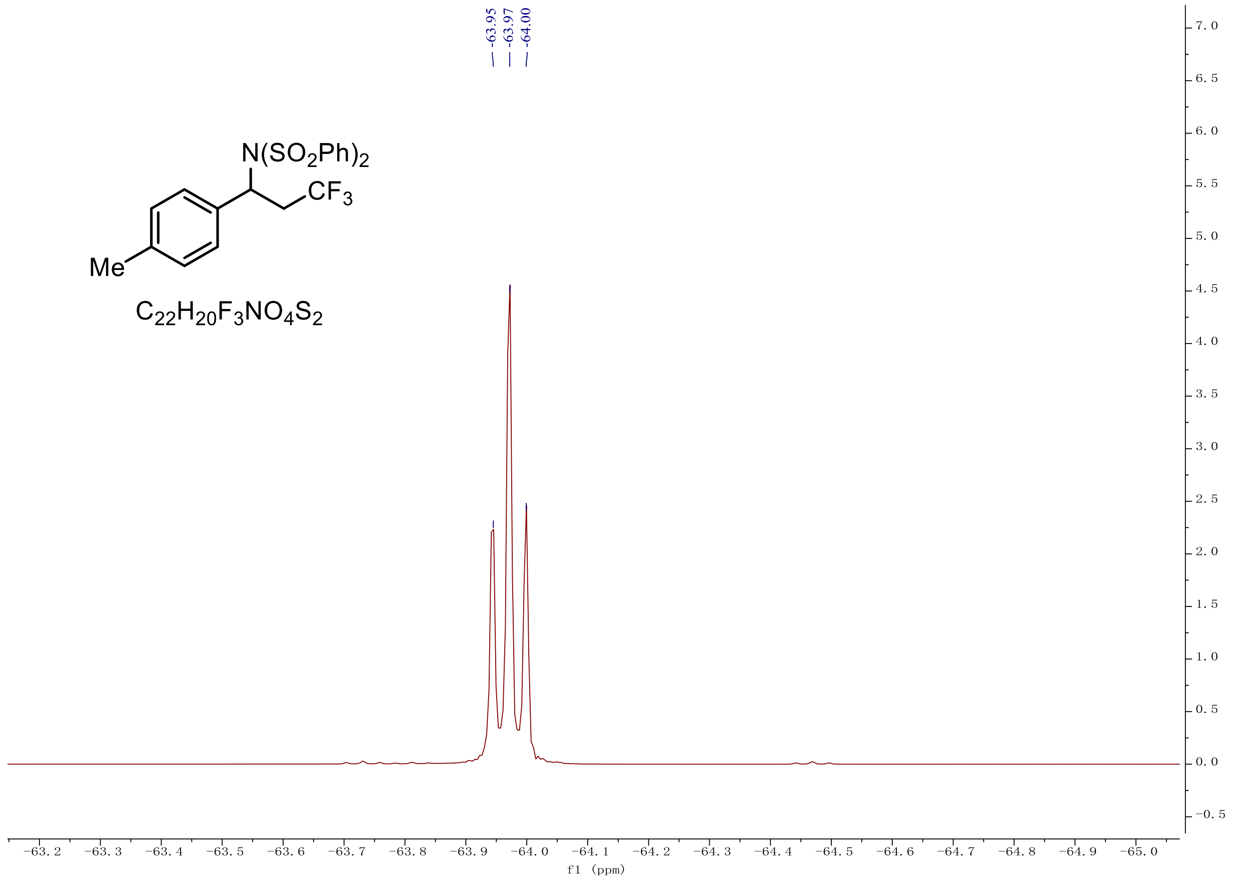

^{1}H NMR (400 MHz, $CDCl_3$) spectrum of 12

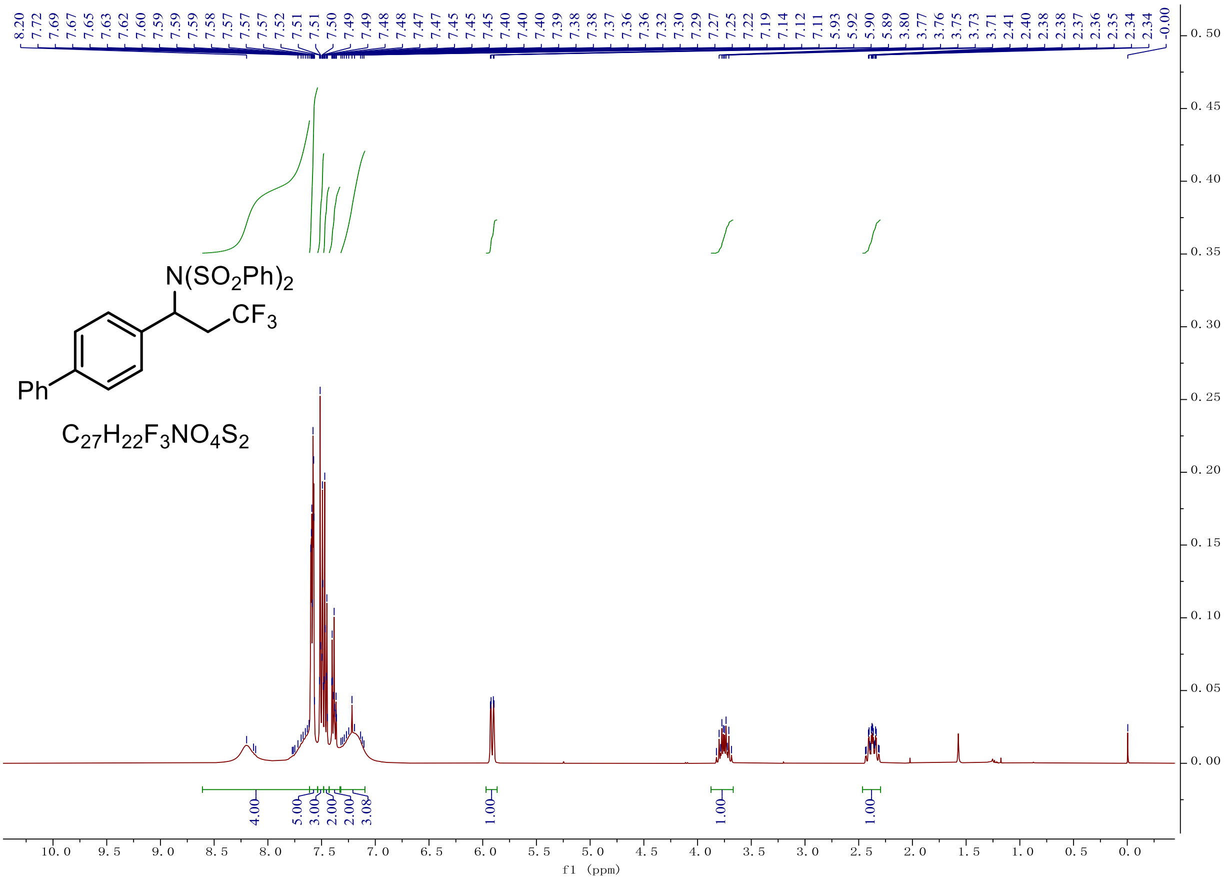

^{13}C NMR (101 MHz, $CDCl_3$) spectrum of 12

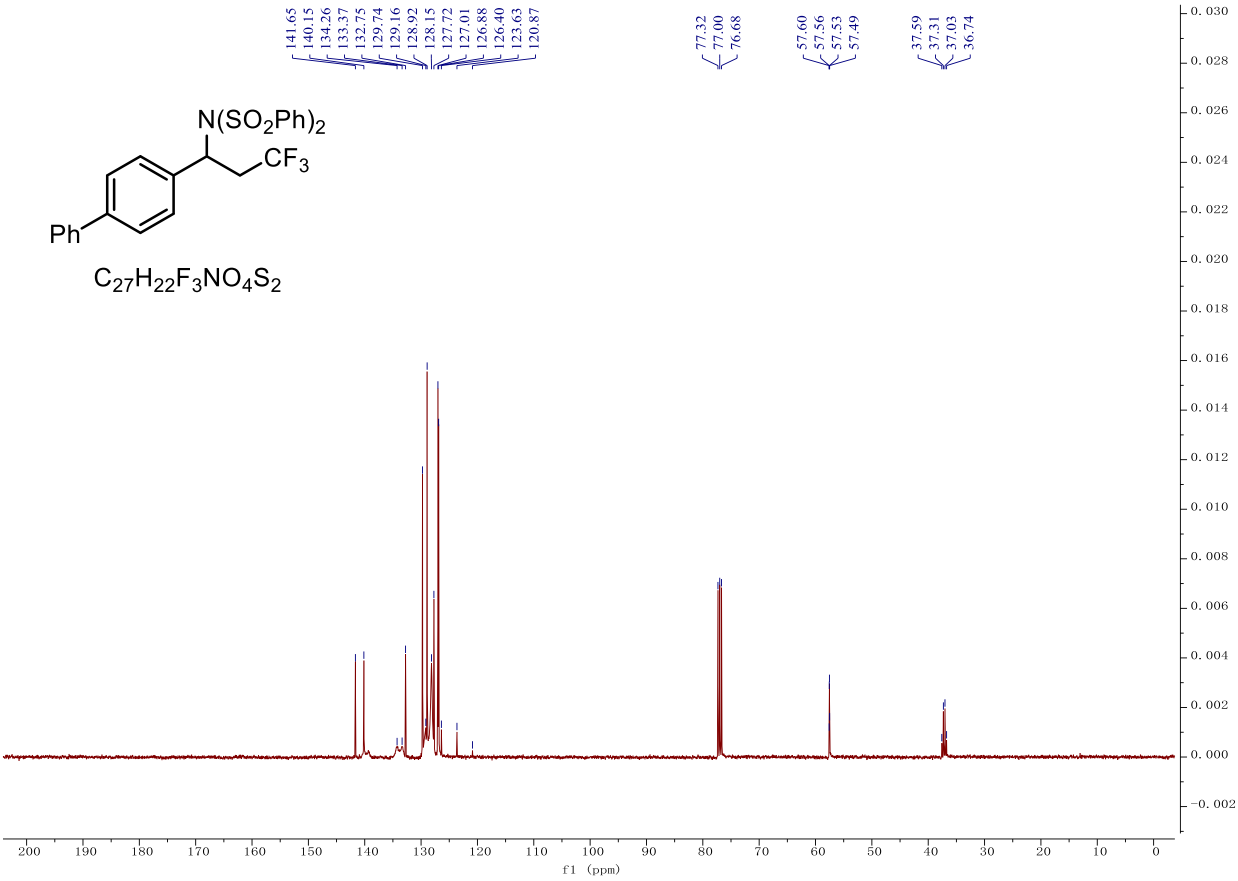

^{19}F NMR (376 MHz, $CDCl_3$) spectrum of 12

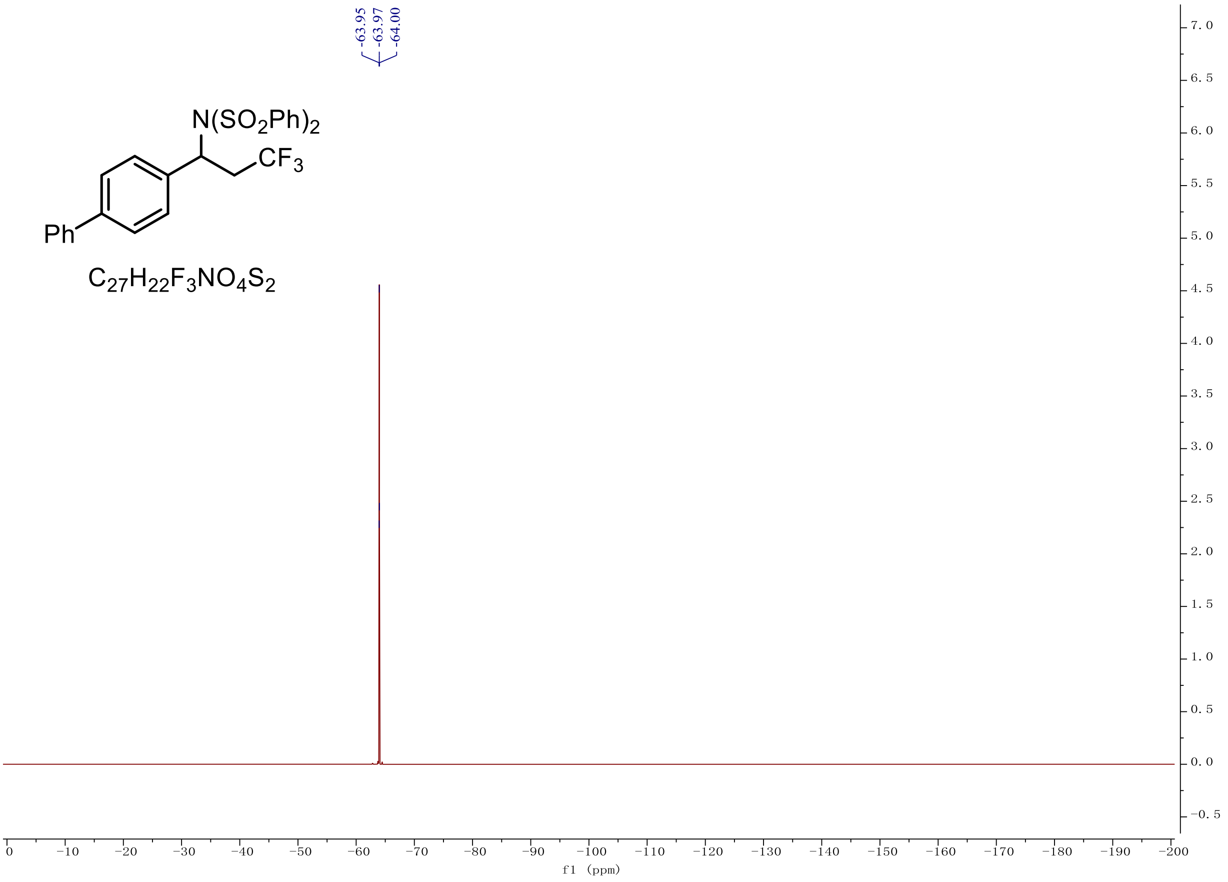

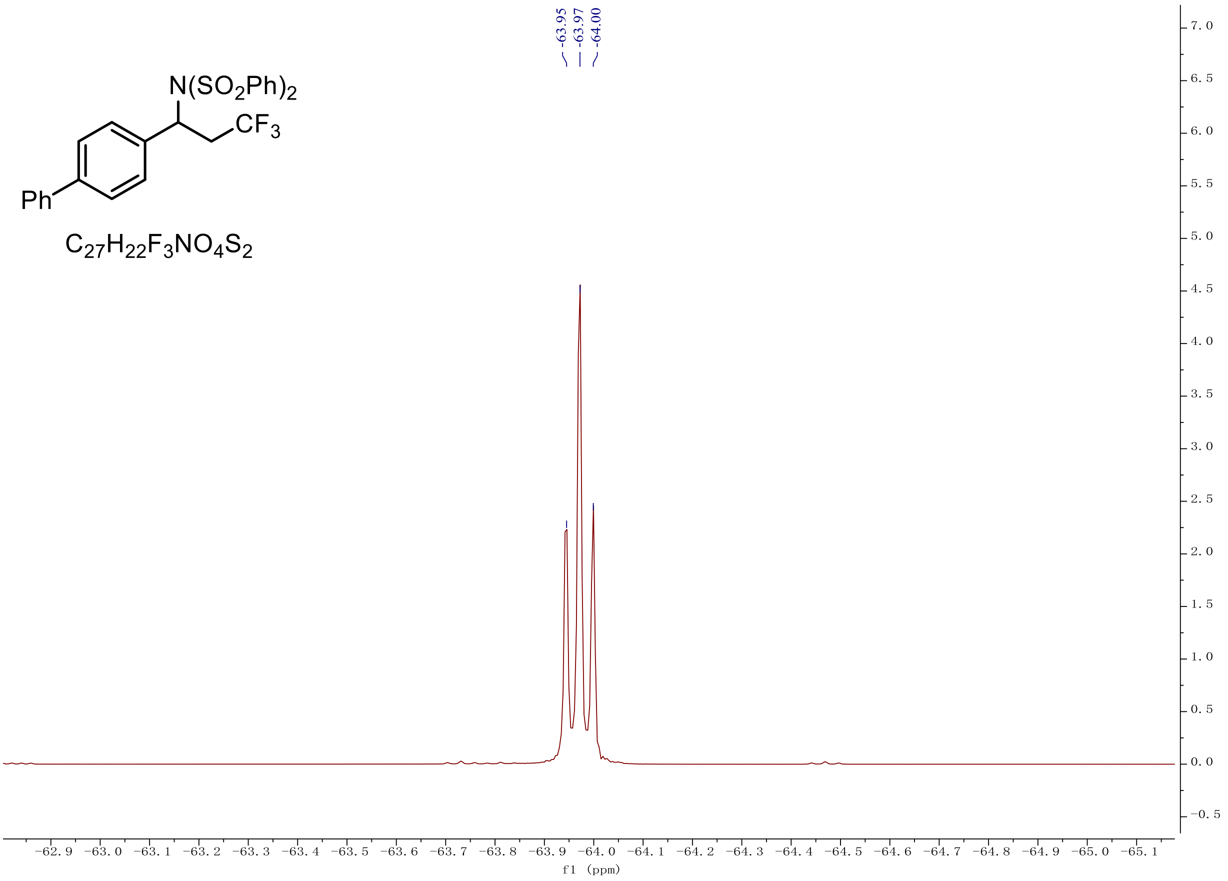

Supplement: Supplementary file 1 — Supporting Information [file ADVS-11-2401243-s001.pdf]
